# Supplementary material for: Repeated Exposure of Macrophages to Synthetic Amorphous Silica Induces Adaptive Proteome Changes and a Moderate Cell Activation
Source: Nanomaterials (Basel). 2022 Apr 22;12(9):1424. doi: 10.3390/nano12091424 (PMC9105884; doi:10.3390/nano12091424)
Supplement: Supplementary file 1 [file nanomaterials-12-01424-s001.zip › Figure S1.pdf]

Figure S1: TEM characterization in water and stability of SAS in culture medium containing 10%FBS, incubation time 2h30 at 37°C.

Methods:

Synthetic amorphous silicas were incubated at 20µg/ml in water or culture medium for 2h30 at 37°C. The SAS incubated in culture medium were then centrifuged 25 minutes at 10,000g and resuspended in water (to avoid culture medium-derived salt crystallization on grid for microscope observation).

Negative Stain On Grid Technique (SOG): 10 µL was added to a glow discharge grid coated with a carbon supporting film for 5 minutes. The excess solution was soaked off by a filter paper and the grid was air-dried. The images were taken under low dose conditions (<10 $e^-/Å^2$) with defocus values between 1.2 and 2.5 µm on a Tecnai 12 LaB6 electron microscope at 120 kV accelerating voltage using CCD Camera Gatan Orius 1000.

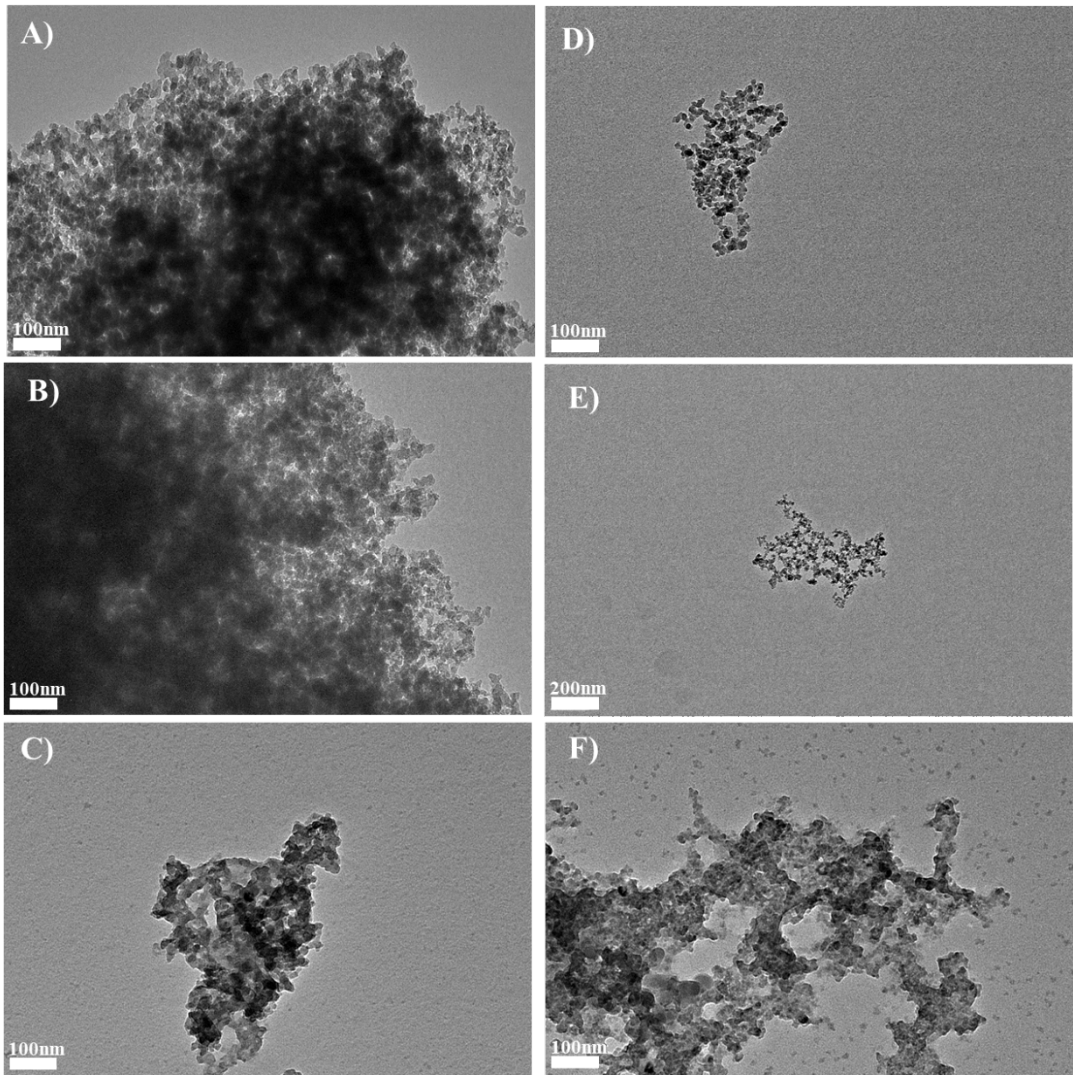

SAS characterization in water: **A-B)** Precipitated silica magnification 23,000× and **D-E)** Fumed silica in water, magnification 23,000× and 9,300×

SAS stability in culture medium DMEM 10% FBS: **C)** Precipitated and **F)** Fumed silica, magnification 23,000×

Results:

The precipitated silica in water is composed of aggregates around 3-5 µm, with a primary particle size of 20 nm. The fumed silica is slightly aggregated, with a primary particle size of 15 nm. The precipitated silica seems to be less aggregated in culture medium, with a size of 300 nm, while the fumed silica seems more aggregated with a size of 3-5 µm.
