# Supplementary material for: Repeated Exposure of Macrophages to Synthetic Amorphous Silica Induces Adaptive Proteome Changes and a Moderate Cell Activation
Source: Nanomaterials (Basel). 2022 Apr 22;12(9):1424. doi: 10.3390/nano12091424 (PMC9105884; doi:10.3390/nano12091424)
Supplement: Supplementary file 1 [file nanomaterials-12-01424-s001.zip › Figure S2.pdf]

Supplementary figure 2: SAS internalization observed by confocal microscopy.

Methods:
The precipitated and fumed silica were labelled with rhodamine B as described in Huang X. *et al* (2020) https://doi.org/10.1007/s11051-019-4720-1. The conjugation used aminopropyl triethoxysilane dans rhodamine B isothiocyanate, instead of fluorescein isothiocyanate in the original publication.
The labelled nanoparticles were collected by centrifugation, and then washed three times with ethanol. The particles were resuspended in sterilized water with care to avoid exposure to light.

The cells were cultured as described for acute exposure. The cells were seeded on glass slide at 500,000 cells/slide, and then exposed for 24 hours to 20µg/ml of rhodamineB-fluorescent silica (rhodB-silica). The cells were fixed with 4% paraformaldehyde for 30min after a wash with PBS 1X. The cells were washed with PBS1X before permeabilization with 0.1% Triton-X100 (Eurobio, GAUTTR00-01) for 5 min. Cells were stained with fluorescently labelled phalloidin, which detects polymerized actin (Sigma-Aldrich, Merck, phalloidin-Atto 390) in a final concentration of 500 nM for 20 min at room temperature, protected from light. Nuclei were stained with SytoxRed probe at 10nM in PBS for 5min. A Vectashield mounting medium was used (Vector laboratories, Vectashield H-1000). Microscope analysis was performed on a Zeiss LSM 880 confocal microscope (Zeiss, Marly le Roi, France). Fluorescence pictures were taken at the same exposure and gain conditions to allow comparison of fluorescence intensity. The raw data were treated and adjusted by using the same parameters with the ImageJ software (1.52s, Wayne Rasband National Institutes of Health, USA).

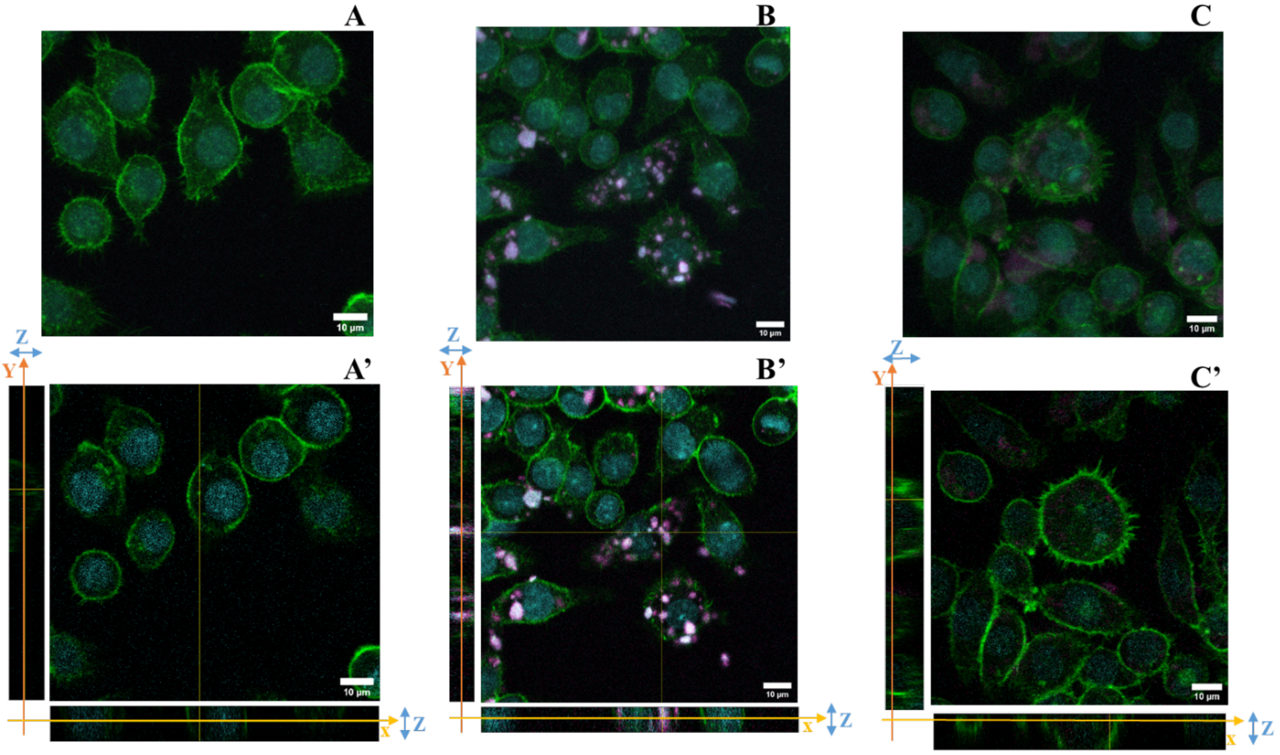

(**A**-**C**) Control and rhodB-silica internalization of cells exposed to 20 µg/mL during 24 h, obtained by confocal microscopy (artificial colors: nucleus in blue (SytoxRed), actin in green (phalloidin-atto 390), silica in magenta (rhodB)), *n* = 2. Z-project reconstitution, **B** precipitated and **C** fumed SAS. (**A'**–**C'**) Same conditions as previously, represented in orthogonal views. The stack of the cells corresponding to a slice in the thickness of the cells is illustrated. The combination of the *XY* and *YZ* confocal plans demonstrate the internalization of the silica nanoparticles.

Results:
The rhodamine B labelling of SAS was efficient for precipitated silica, but not for fumed silica. The SAS particles were mainly visible inside the cells, in the cytoplasm.
