# Supplementary material for: Repeated Exposure of Macrophages to Synthetic Amorphous Silica Induces Adaptive Proteome Changes and a Moderate Cell Activation
Source: Nanomaterials (Basel). 2022 Apr 22;12(9):1424. doi: 10.3390/nano12091424 (PMC9105884; doi:10.3390/nano12091424)
Supplement: Supplementary file 1 [file nanomaterials-12-01424-s001.zip › Figure S3.pdf]

Figure S3: cellular SAS internalization observed by TEM inclusion.

Methods:

For imaging cells *via* transmission electron microscopy (TEM), cells were seeded on Labtek chamber slides at 170,000 cells/500 µl in DMEM 10% FBS. They were then exposed or not to 20 µg/ml of precipitated or fumed silica, for 24 hours. The medium was removed and a solution of DMEM (without serum) and fixative solution (4% paraformaldehyde, 0.4% glutaraldehyde, 0.2 M PHEM) v/v was added for 30 minutes at room temperature. They were then fixed for another 30 minutes at room temperature in 2% paraformaldehyde, 0.2% glutaraldehyde in 0.1 M PHEM (30 mM PIPES, 12.5 mM HEPES, 5 mM $MgCl_2$, 1 mM EGTA, pH 7). Cells were rinsed three times in 0.1 M PHEM and post-fixed in 1% osmium tetroxide (OsO_4), 1.5% potassium ferrocyanide in 0.1 M PHEM buffer for 1h at room temperature. After 3 washes in water, they were post-stained using 0.5% uranyl acetate in 30% ethanol for 30 min at room temperature, in the dark. Cells were then dehydrated in graded ethanol series (50 to 100%), and embedded in Epon resin. After polymerization during 48 hours at 65 °C, the blocs were cut on a Leica UC7 ultra-microtome. Ultrathin sections (70 nm) were collected on Formvar carbon coated copper grids and post-stained. Images were recorded on a Tecnai G2 Spirit BioTwin (FEI) transmission electron microscope (TEM), operating at 120 kV, using an ORIUS SC1000 CCD camera (Gatan).

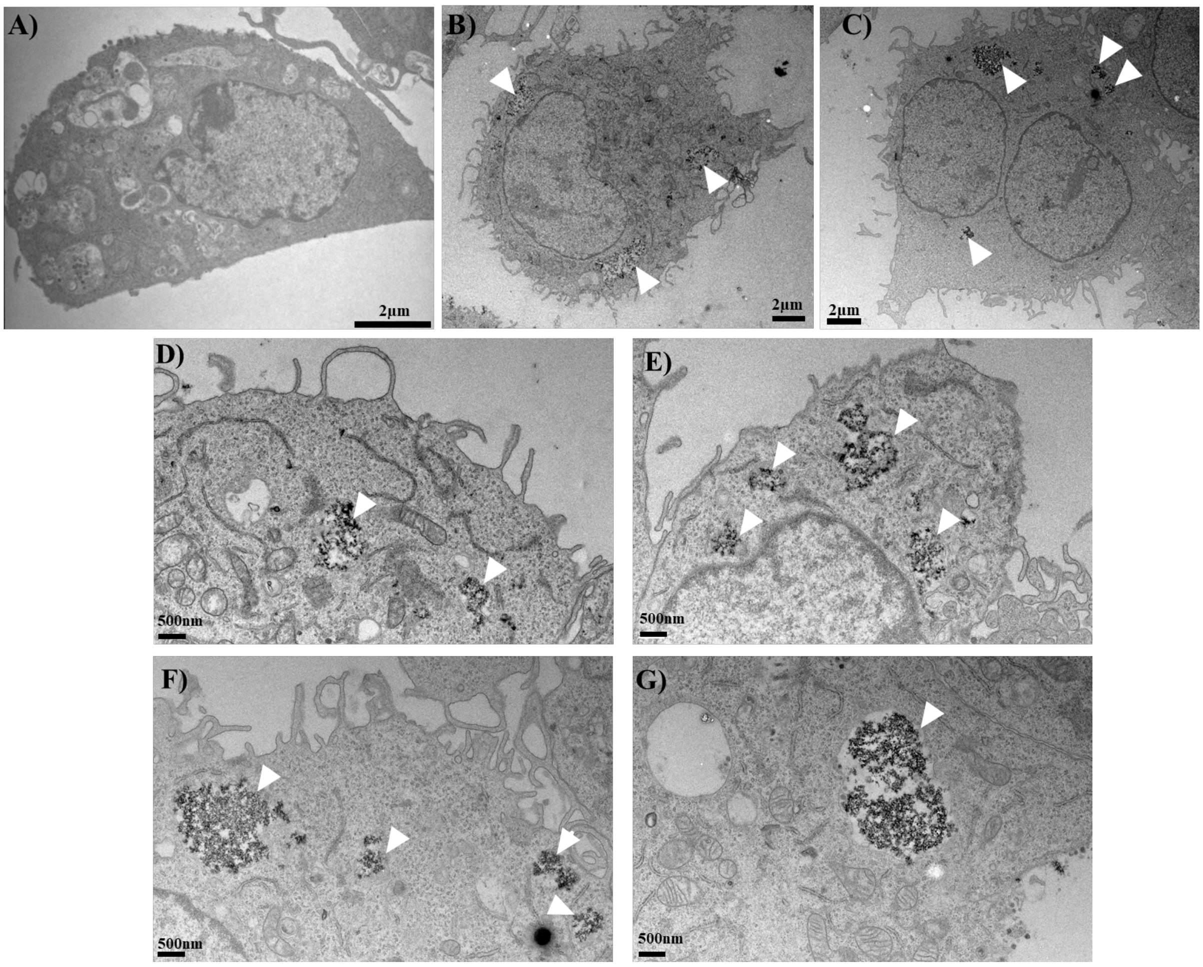

A) control cells (untreated)

B) cells exposed to precipitated SAS, magnification 690×

C) cells exposed to fumed SAS, magnification 890×

D) and **E)** Precipitated silica magnification 2900×

F) and **G)** Fumed silica magnification 2900×

The arrows indicate internalized silica in the cells.

Results:

The cells were adherent with actin filaments. The macrophages exposed to precipitated or fumed silica have internalized the SAS inside vesicles, with no visible SAS around the cells or at the cell surface.
