# Supplementary material for: Repeated Exposure of Macrophages to Synthetic Amorphous Silica Induces Adaptive Proteome Changes and a Moderate Cell Activation
Source: Nanomaterials (Basel). 2022 Apr 22;12(9):1424. doi: 10.3390/nano12091424 (PMC9105884; doi:10.3390/nano12091424)
Supplement: Supplementary file 1 [file nanomaterials-12-01424-s001.zip › Figure S4.pdf]

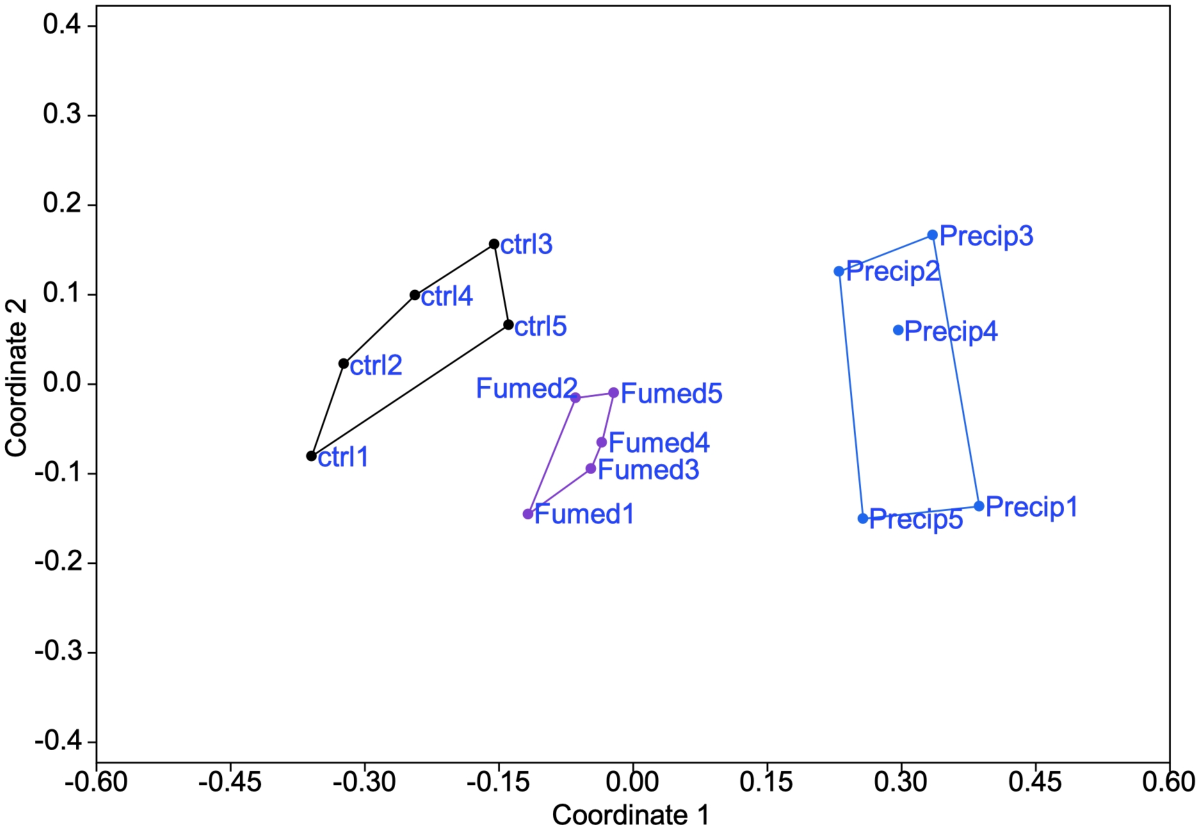

Figure S4: Analysis of the proteomic results obtained on J774A.1 cells after a 10 days exposure to silica by the non-metric multidimensional scaling approach.
CTL: control, cells without nanomaterials, Pr: precipitated silica 220 m^2/g, Fu: fumed silica 200 m^2/g
The results show that 1) the groups are consistent (no interpenetration of the groups) and 2) that precipitated silica induced more changes of the proteome than fumed silica
