# Supplementary material for: Repeated Exposure of Macrophages to Synthetic Amorphous Silica Induces Adaptive Proteome Changes and a Moderate Cell Activation
Source: Nanomaterials (Basel). 2022 Apr 22;12(9):1424. doi: 10.3390/nano12091424 (PMC9105884; doi:10.3390/nano12091424)
Supplement: Supplementary file 1 [file nanomaterials-12-01424-s001.zip › Figure S5.pdf]

Supplementary figure 5: Synthetic amorphous silica exposure and quantification, by ICP-AES dosage.

Methods:

For SAS quantification in the supernatants, the cells were grown on adherent 6-well plates in DMEM supplemented with 10% fetal bovine serum (DMEM FBS 10%). The medium, containing or not 20 µg/ml of SAS (precipitated or fumed) was applied on cells for 24 hours (T0). At the end of exposure, the medium was removed (T24h) and replaced by a fresh DMEM 10%FBS, without any SAS. The medium was changed again after 36 hours, and finally, the medium was collected for dosage (T72h).

The collected media were centrifuged to remove any non-adherent cell, for 5 minutes at 200g. Then, the supernatants were centrifuged or not 30 minutes at 10,000g, in order to collect the supernatant with small size particles only (<200nm). The day before the quantification by ICP-AES, the samples were mineralized: in each sample, an equal volume of 1N potassium hydroxide was added, and the samples were then incubated overnight at 80°C in a preheated water bath.

The standards were first prepared, in HNO_3 10%, the multi-elemental standard was purchased from Sigma-Aldrich (ref 92091). At the end of the standard measurement, samples were prepared two at a time (base-mineralized silica is not stable in HNO_3): from 250 µl to 500 µl of mineralisate were added to HNO_3 10% to reach a final volume of 6 ml. The samples were measured in an ICP-AES Shimadzu 9000 with ICPE solution Launcher software.

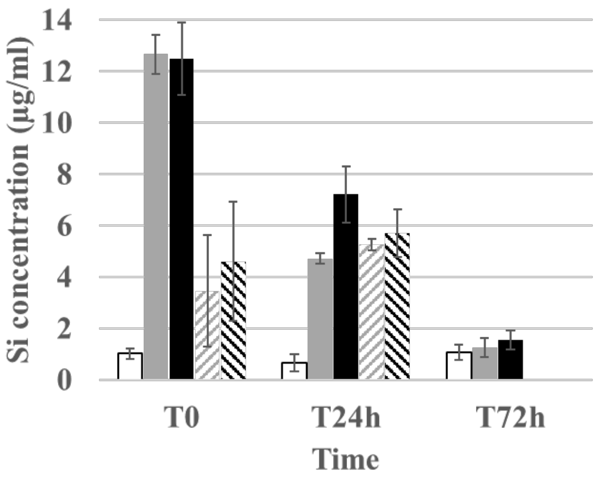

Supernatant of control cells, untreated (white bars), supernatant of cells exposed to precipitated SAS (grey bars, plain or hatched), and supernatant of cells exposed to fumed SAS (black bars, plain or hatched). Hatched bars are for centrifuged supernatants.

Results:

The mineralization yield of both silica is around 60%, determined in T0 supernatants (grey and black plain bars). A proportion of 25% is measured in the supernatant after high-speed centrifugation, and correspond to SAS smaller than 200 nm, that means nanoparticular or silicates. After 24 hours of exposure, 30% of initial amount remained in the medium, and seems to correspond to the smallest SAS particles, and can be explained by the sedimentation speed. After 72 hours of culture without any SAS, there was no detectable release of SAS by the macrophages.
