# Supplementary material for: Repeated Exposure of Macrophages to Synthetic Amorphous Silica Induces Adaptive Proteome Changes and a Moderate Cell Activation
Source: Nanomaterials (Basel). 2022 Apr 22;12(9):1424. doi: 10.3390/nano12091424 (PMC9105884; doi:10.3390/nano12091424)
Supplement: Supplementary file 1 [file nanomaterials-12-01424-s001.zip › Table S1.pdf]

Table S1: list of identified and quantified proteins, with abundances normalized in part per billion

accession number	gene_name	description	protein_set_score	coverage	MW	#peptides	abundance_Ctrl1.mgf	abundance_Ctrl2.mgf	abundance_Ctrl3.mgf	abundance_Ctrl4.mgf	abundance_Ctrl5.mgf	Abundance Precip/1.mgf	Abundance Precip/2.mgf	Abundance Precip/3.mgf	Abundance Precip/4.mgf	Abundance Precip/5.mgf	Abundance Fumed1.mgf	Abundance Fumed2.mgf	Abundance Fumed3.mgf	Abundance Fumed4.mgf	Abundance Fumed5.mgf
A0A0B4J1G0	Fcgr4	Low affinity immunoglobulin gamma Fc region receptor IV	72.487	18.07	28398	4	39199.1254	40783.24956	35556.49013	36118.01865	39123.69075	27629.59008	28880.24694	27611.01412	26285.32985	24375.01463	35482.56764	34927.08094	32841.60974	35668.44065	36295.5207
A0JPJ7	Ola1	Obg-like ATPase 1	219.668	22.73	44535	6	82274.18741	91846.7714	84096.9419	77577.22846	94372.99772	86495.08578	86345.93951	92501.26224	76991.43838	85365.98096	83510.68824	94351.35539	82115.6756	88591.99183	92528.93282
A1A5S1	Prpf6	Pre-mRNA-processing factor 6	88.778	6.38	106764	5	19284.48583	22107.45939	28889.96191	32631.19027	29398.58121	25059.85319	24831.9695	29456.51304	26291.35342	30150.27304	25093.82159	25789.38977	17666.00727	27789.91731	19229.83464
A1L134	Aup1	Ancient ubiquitous protein 1	115.993	13.17	46199	4	11288.9064	8626.344556	17931.7066	8539.454192	15158.96742	20405.69066	22803.89933	18614.40804	9700.546022	20000.48209	11639.88605	13270.35529	15527.14316	14721.39664	17689.45437
A1L314	Mpeg1	Macrophage-expressed gene 1 protein	674.945	37.87	78390	18	270985.5302	290690.2226	299066.9048	306651.2484	284129.8829	512932.7903	504834.4794	553927.0619	543097.6479	395463.1491	280364.3471	314709.092	283823.0185	286390.758	323019.339
A2A432	Cul4b	Cullin-4B	133.737	6.39	110699	5	25357.46478	27985.71566	25521.27573	26714.345	25515.08437	22013.61073	28483.06444	30003.46203	30853.48448	26782.55516	24382.70908	27362.54817	26405.27526	25725.17047	26451.34808
A2A5R2	Arfgef2	Brefeldin A-inhibited guanine nucleotide-exchange protein 2	185.217	4.41	202240	5	11966.06851	11589.80098	12760.37278	11626.13357	9736.615617	8661.744692	9599.587996	12566.264	13088.76094	8834.79459	5888.337078	10137.02645	11756.36018	11407.59027	12959.89439
A2AAY5	Sh3pxd2b	SH3 and PX domain-containing protein 2B	31.872	3.96	101517	3	29090.19323	30299.05295	28345.29327	28780.96837	27827.00585	28913.96872	32515.66241	30931.1368	31854.0312	30920.55281	29209.94605	27975.94429	31478.41193	29930.82787	28075.06625
A2ADY9	Ddi2	Protein DDI1 homolog 2	137.299	18.8	44591	4	27053.59116	31684.44214	25878.21622	28804.77197	26497.00021	18223.66116	9766.676001	17323.70043	17181.35877	19769.30858	28385.84973	23429.13251	31361.3572	28044.08008	27119.55092
A2AGT5	Ckap5	Cytoskeleton-associated protein 5	272.831	6.55	225635	12	55367.49803	56866.51885	55265.13076	55008.64188	54973.54139	56990.89208	56133.61946	61208.28856	63844.09718	61135.76176	48593.98515	59081.85269	53545.19334	57446.45932	56843.63289
A2AN08	Ubr4	E3 ubiquitin-protein ligase UBR4	402.156	3.82	572290	13	49053.91728	51485.92435	41384.20797	46255.18572	43823.30684	38990.05335	45584.1359	45343.30333	41442.83611	41372.04433	40144.68803	39800.25162	46820.54038	43991.6389	43192.97326
A2APV2	Fmnl2	Formin-like protein 2	199.494	4.88	123101	6	56055.62275	52978.95696	53287.51964	53507.80453	51585.7791	45010.26093	46782.28673	46437.69427	47934.28832	50259.34296	47918.35937	50426.32392	47982.38964	55278.6502	50880.98518
A2RRU1	Gys1	Glycogen [starch] synthase, muscle	88.101	7.05	84072	4	23643.45434	20401.76787	27989.29427	29177.0179	23427.47143	30067.73633	35033.54715	32128.5437	37237.5909	22279.59438	28079.3857	27655.24839	27275.9754	27125.9026	30906.64841
A2RSY6	Trmt1l	TRMT1-like protein	90.625	4.4	80861	2	4801.508825	3885.840982	4648.393544	4273.258544	4237.050857	3293.58488	4043.112321	3758.433466	3637.863005	823.0014014	3682.784955	3849.754951	2909.892912	3764.883504	3831.864847
A2RUW1	Tollip	Toll-interacting protein	85.043	15.69	30315	3	31414.45584	31833.20242	27983.08667	34385.0813	30164.63002	25686.10416	27763.51268	33819.37114	28870.81819	24468.88864	27129.09535	31398.41389	27675.58675	29755.71537	28221.78529
A2VCX1	Tiprl	TIP41-like protein	59.903	13.65	31225	2	1124.436964	3842.729994	1331.070623	7308.946582	6698.604554	7374.377256	893.2508298	8326.04246	1026.472359	6778.178977	3692.047914	8647.84603	7472.475779	1296.345814	6840.481923
A4L9P7	Pds5a	Sister chromatid cohesion protein PDS5 homolog A	309.96	9.38	150286	11	63429.17092	53941.52014	67850.58406	64025.41418	60121.84484	57292.46206	65361.1896	61492.69509	61924.02283	60071.48549	52052.05521	62159.51169	58086.22294	62370.19568	62781.86627
A6H5Z3	Exoc6b	Exocyst complex component 6B	77.214	5.56	94130	3	9178.157293	9090.210846	4916.481731	7375.548834	6570.796273	4153.79809	9639.28834	9393.971203	9428.50367	10577.51547	9887.769856	8554.38407	9264.532701	9418.151975	10161.20655
A6H8H2	Dennd4c	DENN domain-containing protein 4C	112.131	2.2	211458	3	5947.45996	5213.832828	4526.716528	5352.962258	5203.628412	4982.039589	5206.836719	5418.706607	5847.080292	5133.178784	5789.807166	4625.995993	4479.588196	5579.617439	5612.275251
A9UMV8	H2afj	Histone H2A.J	423.071	64.34	14045	9	15775724.09	14961418.38	15768989.1	15352083.55	16118673.57	18102989.21	16482237.44	16993907.28	16681569.96	18141460.33	15544061.94	14722481.37	16412267.93	13465380.97	17123953.9
B0BNG0	Emc2	ER membrane protein complex subunit 2	111.856	8.42	34870	2	15405.82219	16554.65356	15765.09539	15132.25107	19080.96043	22127.64274	22849.87424	19105.87408	19552.5286	18067.9977	13346.04712	16069.46249	18192.57937	18054.37691	17965.01167
B0BNM1	Naxe	NAD(P)H-hydrate epimerase	199.691	29.43	30891	4	57464.32921	60050.63995	61329.97125	68961.76989	41226.00451	18796.49291	42662.57028	32076.41431	48277.82203	26501.20784	57322.07465	53159.14517	28334.62145	29729.53786	37469.66413
B0BNM9	GLTP	Glycolipid transfer protein	119.801	32.54	23704	4	56125.1394	53064.1022	51287.7742	45652.58918	41924.71757	30814.51315	31769.41016	28372.12028	38651.05717	38375.948	47258.41607	51551.59447	49278.84411	48449.8964	44707.40457
B1AZI6	Thoc2	THO complex subunit 2	150.676	4.52	182773	6	45995.92108	49662.41796	46917.63903	58110.10736	48615.83532	53942.88849	52682.14686	56939.32309	56707.29846	49934.95978	51718.04426	22891.35687	51233.62685	48837.24225	45039.45593
B1H268	Mcmbp	Mini-chromosome maintenance complex-binding protein	64.848	6.23	73007	4	16874.80506	18280.15861	13401.83692	20335.09039	21214.36623	26470.93346	22150.56935	22792.57235	21337.82334	17897.01812	21026.28543	16458.37805	22857.72804	20533.72514	21094.65006
B2GUV7	Eif5b	Eukaryotic translation initiation factor 5B	215.548	9.7	137686	7	66780.06579	72584.40624	49682.23385	74552.54345	66804.08919	61732.94225	59383.47235	68271.52979	63718.06745	57784.85562	58419.09149	44695.55573	58270.67102	62438.53665	65132.64999
B2RQC6	Cad	CAD protein	670.669	12.18	243238	18	86332.50187	94621.20998	84688.42072	86805.81657	74219.92439	86412.15803	102778.585	98854.07731	98990.22902	96122.70456	85393.90477	88954.75562	82749.71528	84527.65707	89995.17897
B2RX12	Abcc3	Canalicular multispecific organic anion transporter 2	272.445	7.88	169124	7	17139.02056	24232.75343	15987.27203	19579.28827	18757.33346	12812.40764	15139.55339	14769.45041	16005.35522	15500.08045	15520.64466	17846.26773	23566.25976	18766.28207	15621.22641
B2RXC1	Trappc11	Trafficking protein particle complex subunit 11	68.93	2.56	128396	2	6565.583218	6296.46119	5683.375741	6497.188125	6781.489422	4510.488886	5172.005933	6977.154892	6363.053419	5645.542238	4717.439535	5440.041302	6026.939742	7282.92288	6003.433455
B2RXR6	Ankrd44	Serine/threonine-protein phosphatase 6 regulatory ankyrin repeat subunit B	292.332	9.87	107385	6	22499.2513	18788.17334	21190.11696	23429.38636	22795.344	17618.52925	13892.52112	17903.30146	14582.7373	21870.73124	20132.62177	18482.42109	19052.23901	14232.39986	18800.27882
B2RXS4	Plxnb2	Plexin-B2	833.25	21.61	206230	28	259584.866	310327.3357	250420.4627	276834.9817	275066.3025	262659.9789	276495.7578	253657.1997	255526.6456	261798.0571	274144.7856	279203.1975	285625.0544	281458.9554	284835.4018
B2RY04	Dock5	Dedicator of cytokinesis protein 5	25.042	1.28	214429	2	1056.327021	1100.59322	1086.020592	986.4857275	739.8470698	860.4103692	869.0210861	908.1529155	1004.702566	1028.147332	959.330505	1370.465242	922.557431	1095.731413	1033.997502
B2RY56	Rbm25	RNA-binding protein 25	152.921	15.04	99552	8	42490.63847	45823.95128	43314.07929	35741.19406	39447.12141	30784.12861	36274.28787	41972.69158	35050.71537	32543.36117	36284.22451	38248.40245	34810.9823	33111.34949	38753.48034
B2RYG6	Otub1	Ubiquitin thioesterase OTUB1	342.545	46.13	31270	9	92335.01197	101363.7237	81621.12998	104826.3107	97190.75464	92471.53947	97846.75261	99285.10296	90983.00957	85079.78343	90344.26405	100043.3001	100954.9737	104630.5396	94670.45166
B3GNI6	Sept11	Septin-11	222.449	23.67	49695	7	72574.68817	74438.83157	79434.99016	75105.15289	64860.327	70636.7083	77672.51949	74382.76386	69332.69172	65841.51073	76217.14683	74747.71343	66576.25079	69878.47259	64872.67605
B5DF51	Mmgt1	Membrane magnesium transporter 1	153.631	42.75	14677	3	12313.45739	9662.080722	8835.279163	9873.321287	9127.239964	11747.24643	10052.75075	12455.93951	12031.68662	11952.00804	9817.320424	9555.24321	10289.4183	11109.13755	10056.92119
B5DF89	Cul3	Cullin-3	234.173	15.36	88932	9	41767.32971	46972.16798	41273.92146	52735.05192	49025.81366	36297.86409	42389.42512	49405.54951	47472.87815	52598.50765	51685.67753	52042.30684	54092.76837	47863.34329	46421.65465
B5DF91	Elavl1	ELAV-like protein 1	298.701	33.13	36169	8	143292.3387	123840.6449	144173.6677	124160.4279	133434.3308	131075.0413	152263.8724	140035.0069	137379.1673	140371.3398	135046.984	134895.4308	150930.318	140777.2369	140966.4707
B9EJ86	Osbpl8	Oxysterol-binding protein-related protein 8	668.143	27.11	101269	18	276513.1669	271911.7461	268075.5864	249974.3599	246843.7174	299100.6129	355227.974	342382.983	340763.1951	324279.8038	268609.0251	298020.3157	279942.9663	303762.5267	304001.9239
D0QMC3	Mndal	Myeloid cell nuclear differentiation antigen-like protein	63.269	8.18	60526	4	39778.88461	45037.6491	40111.99974	42704.52874	51034.10328	47408.74988	46728.41342	46534.45789	50533.80849	41607.81496	34413.96272	45770.93038	46182.31606	53176.89834	46721.70268
D2XV59	Gtpbp1	GTP-binding protein 1	190.707	15.25	72489	6	24281.34083	23418.65371	28902.56493	28316.99307	22443.91894	23078.97818	29949.14938	25420.81758	27536.99051	27737.21462	23317.09944	21628.54883	28713.49846	22426.63846	18196.60583
D3YXK2	Safb	Scaffold attachment factor B1	69.403	3.2	105104	3	45048.74495	45197.73788	27592.87811	21827.22207	42660.34595	22099.06658	26101.10553	18789.0863	23486.68906	17507.39766	24577.60748	22073.77615	26347.34763	26008.45167	44232.34839
D3Z6Q9	Bin2	Bridging integrator 2	199.295	17.18	52554	5	18838.63697	29337.46533	20091.58836	21973.40488	15334.42681	11909.23892	21479.08248	21399.61947	21111.81265	18985.20413	14611.57914	18252.37943	22770.39017	18809.63821	24524.4116
D3Z7P3	Gls	Glutaminase kidney isoform, mitochondrial	177.63	8.75	73964	4	37964.56635	38825.81656	37000.56396	29785.31198	32379.04743	34477.69566	32678.73018	27560.1293	31875.55172	27268.04101	29111.07365	30155.88483	27415.02525	30852.87299	33907.39028
D3Z8L7	Rras	Ras-related protein R-Ras	63.982	10.55	23909	2	22399.12446	32211.83366	27376.41337	31685.8994	22208.38949	34844.12042	28414.44397	22921.19822	33146.23766	38379.88745	29000.51582	28191.52506	30091.33761	32761.19069	32008.5357
D3ZCL3	Snrpc	U1 small nuclear ribonucleoprotein C	61.28	16.98	17364	2	9798.285185	7642.577764	7562.538379	7989.89016	9486.589916	8468.165437	5599.413665	6232.081439	5897.838069	4483.519968	5341.390712	5795.137427	4698.683457	4253.414557	6480.571409
D3ZLZ7	Impdh1	Inosine-5'-monophosphate dehydrogenase 1	75.457	5.06	55293	3	55682.72297	55040.99329	57725.41297	56470.0068	59174.98656	60318.99765	59600.84871	70955.70754	56813.8256	60532.96336	59542.77416	56983.62131	54665.11277	55323.40706	57289.70581
D3ZTX0	Tmed7	Transmembrane emp24 domain-containing protein 7	93.147	14.16	25479	2	14746.41252	14787.55334	13400.5935	14356.17623	14903.85511	17661.68701	18614.91874	15762.44315	12330.57372	13648.47957	12184.16205	11442.87525	12077.75721	14023.59088	14514.96702
D4A4T9	Chordc1	Cysteine and histidine-rich domain-containing protein 1	94.393	16.31	37362	4	41981.80261	37915.45674	35845.39667	38477.8147	38557.92577	37746.11535	40179.15013	49136.0361	42281.26909	32511.10634	35989.91057	38563.75915	33691.15608	38118.33001	37540.20044

D4A631	Arfgef1	Brefeldin A-inhibited guanine nucleotide-exchange protein 1	177.655	3.25	207890	5	17438.37484	15652.28154	17254.61831	15578.3175	16015.48295	16165.7652	15076.59161	18059.53689	11593.50374	17042.46985	12704.53917	14641.91264	15290.29873	16047.2641	16869.88196
D4AE41	Rbmxl1	RNA binding motif protein, X-linked-like-1	209.356	15.98	42250	7	161433.3002	162972.5616	172382.8598	172738.1314	175003.3189	152573.7196	170435.2818	141831.4852	151609.47	152120.757	174188.5247	167344.3619	182227.0242	177162.7353	159462.7568
E9PVA8	Gcn1	eIF-2-alpha kinase activator GCN1	950.724	15.46	293021	30	201110.6101	195927.4064	202721.2273	206996.3855	200843.1709	259928.2996	256546.9955	247360.7911	268080.4388	217864.0713	206431.9654	218210.9091	192418.8724	204103.5442	211272.7761
E9PVX6	Mki67	Proliferation marker protein Ki-67	309.544	7.9	350864	12	53153.83644	63441.61202	73201.84314	69799.63733	75052.84193	47396.88177	65364.98676	48253.58573	54361.01889	54967.30309	73694.59389	58538.26462	61400.47017	67573.28177	60377.01672
E9Q1P8	Irf2bp2	Interferon regulatory factor 2-binding protein 2	130.928	11.58	59292	4	29222.02642	29130.12469	29079.3852	25268.41734	29745.12528	26522.79874	23439.16967	24055.63916	24191.26682	24490.75706	28296.70142	28520.91357	24633.77794	25203.11433	25177.65659
E9Q3L2	Pi4ka	Phosphatidylinositol 4-kinase alpha	102.128	1.62	237042	2	5458.826993	5357.480423	4704.588883	4364.419911	4546.641908	4982.128573	4742.564964	3973.061039	4879.431734	4714.122774	5210.215947	4944.907362	4333.335112	5958.950795	847.0776106
E9Q555	Rnf213	E3 ubiquitin-protein ligase RNF213	1509.702	11.59	584784	40	185282.779	192406.6481	173891.2088	206454.8135	174660.137	231588.8638	230885.9772	245923.2196	245008.3715	211914.41	182647.8074	199871.5017	163673.268	174015.2872	173996.4321
E9Q634	Myo1e	Unconventional myosin-Ie	753.556	27.55	126818	22	215521.1424	257169.21	224111.7135	239053.7398	214177.4893	236270.5047	256880.7006	255674.6101	250748.4394	241243.1439	244899.9563	222625.3143	258838.7068	240091.1576	244911.8471
E9Q7G0	Numa1	Nuclear mitotic apparatus protein 1	1707.656	34.62	235630	48	439269.3358	446280.1195	446012.6279	444480.6285	460376.3457	354781.589	339391.3523	392140.642	361472.9119	366912.6338	441828.0124	383603.1361	430440.9465	401908.4759	421287.207
F1LMZ8	Psmd11	26S proteasome non-ATPase regulatory subunit 11	596.643	43.13	47464	16	279823.4771	285585.8642	293265.9883	290393.8531	289354.9969	332846.964	342161.5596	339914.0161	339946.4065	311994.2757	271739.0024	305926.7993	307423.6998	314320.4353	309937.4481
F1LNI5	Ppm1g	Protein phosphatase 1G	85.929	4.43	58743	2	10075.60935	11628.80792	12425.30629	11562.20639	11956.03697	12817.39842	12883.62146	11210.4511	12338.8274	11253.14865	13541.18389	10597.07241	10709.27709	11317.42762	11376.96518
F1LNJ2	Snrnp200	U5 small nuclear ribonucleoprotein 200 kDa helicase	1370.18	26.18	244875	39	262417.4071	290472.9393	269195.9117	276014.3513	291651.1202	260596.4615	294304.5049	290638.6581	287971.7478	278404.7604	263713.2769	290307.7097	273160.0374	276386.4235	282437.4005
F1LP64	Trip12	E3 ubiquitin-protein ligase TRIP12	297.575	6.62	223928	10	44539.04532	50029.19134	49479.52996	44976.23863	46200.53068	36539.47557	44421.80799	38328.46301	47784.34557	46411.22755	44204.57003	32841.63828	45033.40338	39578.49996	45449.03428
F1LP90	Mink1	Misshapen-like kinase 1	27.601	2.47	150382	2	11024.30016	15081.66798	11827.71378	15966.97397	14967.15403	17661.32351	18061.42618	15712.04781	19978.49216	2421.042407	17044.25457	16134.16684	11313.0198	12750.83022	16431.016
F1LQ48	Hnrnpl	Heterogeneous nuclear ribonucleoprotein L	894.689	60.67	67903	25	508020.3411	525763.589	496055.2591	470609.2495	515460.3272	443745.0093	419379.9763	422833.3292	402523.7539	393307.5166	493381.4631	474475.7906	518757.8561	472038.4304	418205.2732
F1LSG8	Vps50	Syndetin	85	3.63	111188	3	11660.58149	8828.764901	12369.52825	14460.24762	8760.917761	9567.635117	12899.47999	8914.311141	12038.35967	8030.472619	6468.306519	12168.47146	8195.903357	12750.84085	8566.247604
F1M0Z1	Trio	Triple functional domain protein	43.187	1.23	347860	2	5516.774821	5610.4579	2691.678099	5603.847519	5824.894999	4879.668496	6686.596107	4703.810713	5498.399482	5791.564787	4616.570085	5536.200575	5591.095415	4924.295468	4683.89971
F1M775	Diaph1	Protein diaphanous homolog 1	461.72	12.65	140409	12	74301.01755	63867.21722	70912.56351	66907.67654	66772.91037	88987.74537	77230.80222	82604.72459	79106.58088	70400.35136	70435.24628	84727.62534	75094.26129	78769.18124	76150.16052
F6ZDS4	Tpr	Nucleoprotein TPR	576.672	10.08	273990	17	101036.6403	95580.01841	108815.69	114724.527	99262.96415	99328.18174	100802.8106	101070.6666	99357.55794	101480.2303	100625.176	98635.09292	95943.1153	98497.91419	100402.4765
G3V6S8	Srsf6	Serine/arginine-rich splicing factor 6	94.874	9.14	39025	3	131356.9627	123124.2799	135216.0577	130876.7389	118565.3731	121574.86	122810.6007	124721.4906	99870.77563	108388.3523	123883.8383	122067.1418	130715.4694	117437.9506	122848.4435
G3V9R8	Hnrnpc	Heterogeneous nuclear ribonucleoprotein C	583.779	37.25	32857	13	263892.2574	268958.4716	297709.8396	298336.946	241350.0622	280079.5601	290936.1622	305227.723	264340.0022	258167.6935	256957.9686	265399.688	300347.3455	321532.1236	289773.28
G5E829	Atp2b1	Plasma membrane calcium-transporting ATPase 1	383.056	12.54	134747	11	95333.44539	83266.98391	67533.03365	83602.28587	76960.26327	97361.17951	107473.8329	102404.2432	98695.21313	100208.6186	81592.63876	71434.07014	85043.95941	77302.03488	80638.33963
O08528	Hk2	Hexokinase-2	826.505	39.04	102535	30	336599.2744	346847.3301	360622.5464	344898.8776	354229.723	288574.6415	317923.3325	311154.9489	311559.7274	231115.7525	298499.8211	326225.234	288512.0167	336510.8898	334060.9116
O08529	Capn2	Calpain-2 catalytic subunit	654.542	36.43	79872	20	267259.7078	238570.0838	220517.6113	231706.9704	235493.4036	209204.1446	220068.4175	196243.0881	212122.2499	211830.706	224969.3903	232441.2282	232405.3017	229032.8006	245970.5366
O08539	Bin1	Myc box-dependent-interacting protein 1	97.106	8.5	64470	3	19987.8848	13134.53783	23685.9639	21277.56344	10255.34579	7840.56321	21354.47712	10557.50489	17050.0486	22382.96236	14379.73057	12948.22383	15306.94409	21781.43477	12108.77839
O08547	Sec22b	Vesicle-trafficking protein SEC22b	311.197	61.4	24741	9	55213.13907	57161.55137	51205.57047	53609.97062	47546.43156	41965.37976	54371.37489	46911.19483	47180.723	45216.09043	46356.60138	55638.95467	53061.16307	46807.84558	52552.28615
O08553	Dpysl2	Dihydropyrimidinase-related protein 2	1630.64	79.2	62278	33	1961265.623	1932813.829	1857964.148	1832358.196	1964268.572	1376391.05	1421111.213	1429171.027	1394428.273	1458417.048	1687081.742	1638124.239	1743962.126	1737091.316	1726232.076
O08560	Dgkz	Diacylglycerol kinase zeta	32.898	3.77	104009	2	5328.804581	6562.205385	6029.369694	6718.587188	6259.582551	5231.534768	5796.358305	3742.869912	0.012083387	7401.013001	4547.578584	4649.261571	6532.658534	5698.34826	5135.424435
O08561	Pi4kb	Phosphatidylinositol 4-kinase beta	40.23	1.96	91655	2	3428.329116	5854.973486	2252.571783	5866.317077	7248.022766	6854.059629	7117.996965	6173.434552	6462.831986	5516.452577	5814.830529	6350.811141	6724.711238	6613.25621	6816.709947
O08579	Emd	Emerin	135.864	29.73	29436	4	33130.16091	36331.52768	41143.73544	37955.13745	33112.88212	35025.58438	33631.81628	37590.44466	35220.46279	39816.97577	39681.79353	34320.46637	36431.16528	36690.17686	35851.38
O08583	Alyref	THO complex subunit 4	246.457	41.96	26940	5	46968.7675	49909.66736	43169.28129	42314.85772	35822.14964	24740.14118	29577.97894	20919.46735	21639.09087	29099.20373	39971.73424	36668.37872	41152.07567	32823.23373	31139.03515
O08585	Clta	Clathrin light chain A	53.48	6.38	25604	2	0.008226335	2573.714592	851.9605061	162.7194985	606.9259413	2019.7958	0.012348474	1698.867431	0.012083387	0.013320187	414.1805425	768.0626992	4171.10871	0.00945634	159.5264837
O08599	Stxbp1	Syntaxin-binding protein 1	53.807	5.22	67569	2	4093.520339	3320.638775	8498.689899	8860.94723	3384.511241	9974.878542	3409.083376	6329.545839	6598.244462	9138.70884	3205.40215	3076.893949	9384.895157	2459.446034	10482.36393
O08618	Prpsap2	Phosphoribosyl pyrophosphate synthase-associated protein 2	66.222	9.49	40867	3	13753.68614	13931.74595	13020.32756	5605.415778	22774.48511	8904.493913	21901.08695	16437.08589	11799.96804	15634.37624	14111.8431	13924.13049	17216.30652	10767.14785	9097.38664
O08638	Myh11	Myosin-11	625.102	7.86	227028	18	720522.0811	724313.0589	642229.0812	670649.7289	581439.1076	616965.3459	615293.1115	612514.0571	603903.9091	629362.6475	681100.2457	660945.2051	692605.0268	684751.4895	664403.2106
O08697	Arl2	ADP-ribosylation factor-like protein 2	99.388	24.46	20836	3	2090.132762	10648.02972	8599.294746	10455.08337	8253.72102	9720.300249	10883.57307	8846.464194	11806.35562	9081.126538	7701.088119	11822.12681	8922.510479	9960.102458	8918.68116
O08700	Vps45	Vacuolar protein sorting-associated protein 45	189.022	14.74	64894	7	33060.78616	30485.68581	22298.54777	31551.65278	18066.56596	26437.44466	29017.73485	29360.47845	29404.81628	29576.53929	26012.58807	23892.42659	33372.41186	29264.75167	31294.37146
O08709	Prdx6	Peroxiredoxin-6	499.923	80.36	24871	13	426853.7228	394210.2557	381962.0024	361269.4076	375503.8617	341041.246	314778.0527	298759.3456	332688.5202	320213.0175	384276.7918	364521.8387	366404.3473	345348.1796	347837.7792
O08734	Bak1	Bcl-2 homologous antagonist/killer	209.444	36.84	23295	6	59723.6264	71470.5229	72658.36562	71379.47457	69548.25103	71093.6942	68860.34584	60275.25018	68105.37607	70425.9794	63130.46	91859.27797	73307.11939	71781.4711	71030.19802
O08738	Casp6	Caspase-6	73.728	14.49	31595	4	3813.155027	9613.305031	9759.573623	8397.546267	11796.06677	11383.18352	8196.230221	9084.086676	8693.027181	9791.552047	9432.80611	10976.91949	14225.4677	10272.81297	10140.07327
O08739	Ampd3	AMP deaminase 3	178.866	10.05	88652	8	69945.53495	68914.47933	58346.46289	51829.73559	62435.39263	79201.50074	77191.50938	81301.01761	84311.00436	81159.86554	73479.78664	56663.64546	64106.09466	69544.19098	38267.42657
O08749	Dld	Dihydrolipoyl dehydrogenase, mitochondrial	580.198	41.06	54272	16	453909.1854	430088.3825	405094.6103	421200.112	383666.166	441023.0716	426247.6048	423136.3851	431933.2435	427158.1875	409252.4638	411338.5857	424372.6349	442813.1968	411420.6093
O08756	Hsd17b10	3-hydroxyacyl-CoA dehydrogenase type-2	398.753	52.49	27419	9	138440.1829	122243.0316	119729.5375	116360.1566	113932.7229	111329.4267	116451.6799	120181.026	134143.0671	128067.5365	124010.2476	118308.1399	116946.2075	118124.1404	113302.6457
O08784	Tcof1	Treacle protein	85.208	5.53	135001	5	19124.84351	23559.85725	21077.90232	23462.82421	21297.1984	15320.52122	22094.97034	16791.25401	14278.76158	16091.27364	24309.41222	20927.20543	15542.7951	19690.51515	20989.45816
O08788	Dctn1	Dynactin subunit 1	292.672	10.62	141676	9	56066.78178	60688.03437	55803.49669	58854.02144	63310.2966	46558.48786	57659.47101	49109.54787	55093.13318	45624.10107	55550.90856	56716.8925	59160.38602	56789.55105	52677.49697
O08795	Prkcsh	Glucosidase 2 subunit beta	194.348	20.73	58793	9	140378.2417	130126.4643	135947.8649	135854.0802	135015.6144	150555.9735	159535.5801	140678.7229	151963.9603	134507.8468	120047.5382	125802.8397	140752.1374	142602.3861	130934.6347
O08807	Prdx4	Peroxiredoxin-4	341.044	54.74	31053	12	509197.1019	502400.9275	452382.5228	491206.6311	563177.114	640786.5972	604602.8399	656447.3325	695078.3316	665494.134	559655.5511	578431.222	622441.2858	607429.8375	587365.1002
O08810	Eftud2	116 kDa U5 small nuclear ribonucleoprotein component	636.795	25.33	109361	17	345369.2389	369608.5074	377467.7295	373510.1897	365675.9869	361919.3511	382483.1802	381293.8677	372224.7579	364883.0369	357095.9821	380883.4523	368974.5915	363125.4551	370419.3847
O08837	Cdc5l	Cell division cycle 5-like protein	229.349	9.73	92218	6	62473.94943	63538.39582	67056.94816	59588.0995	73355.33208	55691.90277	53839.11244	61031.27914	63768.02017	55913.88885	65233.39209	55758.57257	70743.65994	65266.40772	58428.38812
O08848	Trove2	60 kDa SS-A/Ro ribonucleoprotein	31.017	4.28	60124	2	4542.296506	6278.63892	6225.491534	7356.095226	5912.517236	4705.144085	6536.706439	7401.893083	6121.492861	6177.423961	5141.105721	7075.271815	6324.320455	6271.509439	6546.068668
O08917	Flot1	Flotillin-1	122.542	15.19	47513	5	35620.08546	23690.14065	24514.63671	40246.17314	42260.65861	22774.04951	21872.15448	33891.62534	30502.13584	36692.76257	29671.72814	21805.30835	38681.90728	35338.14725	24915.43963
O08992	Sdcbp	Syntenin-1	99.91	21.07	32379	3	12906.4826	13813.96605	11963.09696	14055.46033	12776.11867	15381.51204	17539.98562	22209.25846	19813.75481	13638.00157	14585.27639	14394.6703	12654.33899	12268.01197	10824.52575
O08997	Atox1	Copper transport protein ATOX1	124.127	58.82	7338	4	97938.01833	76976.63459	66939.73367	61997.59604	67776.36498	61199.65876	49646.14484	49146.89871	49610.59846	39996.19223	61471.62999	56886.45158	51731.91336	56028.30152	51142.46656
O09043	Napsa	Napsin-A	104.986	11.22	45544	3	8075.213933	11248.0696	7861.411881	9811.686885	9328.091038	8727.879914	7842.605425	6462.663953	7514.748937	8604.252125	8699.979335	7631.735766	7631.890228	8680.404955	3853.676736
O09061	Psmb1	Proteasome subunit beta type-1	352.181	43.33	26372	9	164296.904	169118.6427	159479.6801	172517.4563	158135.4947	136519.2802	160479.695	168793.1297	157030.899	155142.4015	166254.8503	167141.1917	167391.8803	169004.0513	159095.1485
O09106	Hdac1	Histone deacetylase 1	262.786	23.24	55075	7	69482.65551	75501.20729	68604.74428	67380.29102	75052.1724	62719.99834	51614.37739	64640.39258	65967.45034	60523.55931	73357.67416	72944.56885	70687.11574	73349.34639	49412.89342

O09131	Gsto1	Glutathione S-transferase omega-1	126.438	29.17	27498	6	72297.83908	74790.95277	67611.91366	72476.04509	74649.40189	66409.62511	66618.04817	52717.10604	62418.48711	64831.29444	75354.43373	68074.62478	72814.14585	72848.83179	72914.08956
O09159	Man2b1	Lysosomal alpha-mannosidase	557.178	24.38	114648	17	247770.8613	219268.2579	223566.0724	240154.3273	217784.1645	114832.5951	141303.3051	104673.6978	123056.6201	140026.973	186737.5457	200474.3643	207592.1826	213967.0738	195561.7844
O09167	Rpl21	60S ribosomal protein L21	125.555	36.88	18562	7	383426.6024	393902.4956	383249.9495	397415.8086	392568.5291	423591.248	446812.2103	449680.328	401700.4403	385041.1151	373324.0681	402530.7517	397095.2368	411963.0992	400282.001
O09172	Gclm	Glutamate--cysteine ligase regulatory subunit	94.314	10.22	30535	2	19624.76202	11948.05719	27803.11386	10955.41866	14922.21359	26885.20324	27962.19346	29009.42781	14849.85114	12231.00855	11481.52243	21908.2158	23479.24567	13282.67249	12392.14874
O35114	Scarb2	Lysosome membrane protein 2	48.249	12.97	54044	4	30316.47658	30666.68089	24432.10013	27695.8709	26692.20054	25697.76145	21418.62898	13517.70792	23804.86125	28801.53419	31733.77234	32465.75031	36355.79265	33655.08429	29954.49763
O35129	Phb2	Prohibitin-2	503.598	58.19	33296	15	539452.609	533144.1438	545562.4059	546039.5226	533728.173	619590.8847	615948.8154	614025.0041	566651.6973	631998.2862	528597.5253	546438.7988	572753.1169	597217.4068	547113.4311
O35130	Emg1	Ribosomal RNA small subunit methyltransferase NEP1	98.513	18.85	26974	3	5524.497828	8162.521749	4395.34358	4869.467406	4134.284574	9606.060172	7809.09321	12391.32976	9578.469075	9821.355099	6326.899201	4670.56401	10637.45625	8752.020794	9784.24538
O35143	ATP5IF1	ATPase inhibitor, mitochondrial	26.206	22.64	12159	2	28871.96706	3727.046058	28828.19262	1282.188338	27243.02705	21817.29254	19272.77135	0.01388636	0.012083387	0.013320187	24744.66123	21646.99374	26769.33432	19248.2587	22935.91367
O35215	Ddt	D-dopachrome decarboxylase	43.602	19.49	13077	2	10885.33576	8554.171696	9951.440345	8989.832189	6308.390919	8427.474064	7788.752186	6461.891594	3496.355549	7471.754915	11162.6488	9106.340363	8313.999335	3254.975545	6190.474563
O35226	Psmd4	26S proteasome non-ATPase regulatory subunit 4	259.238	24.2	40704	7	115665.335	114185.981	103741.7591	100749.1957	102771.7882	116355.7845	115907.3345	104963.3465	110333.4299	97174.92607	110610.6555	108300.2051	97876.56763	99676.74149	102996.9735
O35239	Ptpn9	Tyrosine-protein phosphatase non-receptor type 9	99.563	10.29	67970	4	16032.28745	9544.6175	14581.49701	16526.96729	16113.03972	4489.361612	6487.360393	5955.213884	8027.323188	10047.08065	12526.52116	4801.850072	5086.121077	7721.337129	9842.818618
O35263	Pafah1b3	Platelet-activating factor acetylhydrolase IB subunit gamma	86.012	13.36	25863	2	19507.23443	16855.12914	22768.79417	21588.22311	21041.22335	19383.19381	21777.15767	13764.57268	14300.73522	16542.04541	16195.33303	20647.34373	19569.15528	17107.11528	19907.18185
O35264	Pafah1b2	Platelet-activating factor acetylhydrolase IB subunit beta	163.641	36.68	25581	4	60608.61967	47504.85698	47533.78183	53364.39139	40903.16899	28690.70646	36217.41697	29693.02899	42991.38255	39551.90406	45321.23623	48156.58165	44880.21993	46404.73128	40359.38118
O35286	Dhx15	Pre-mRNA-splicing factor ATP-dependent RNA helicase DHX15	433.044	24.91	91007	16	211779.5912	214119.2965	214393.0153	212886.8203	218954.6432	220254.6394	223073.3199	227012.914	220258.8643	216533.4113	200723.3999	211674.9368	215891.2573	221588.9213	222199.348
O35295	Purb	Transcriptional activator protein Pur-beta	243.206	24.38	33901	4	61228.52339	56841.44566	48897.10458	48587.20478	57210.91102	41274.09953	44499.11561	46527.98338	39763.68335	45799.87759	62941.6789	55180.07368	55258.40491	46046.40693	56111.69535
O35303	Dnm1l	Dynamin-1-like protein	215.368	14.3	83908	7	40620.0891	37092.44282	37957.04293	35192.40897	36350.79265	39129.55545	31332.81442	65052.63693	42363.708	29702.2652	39113.71622	37238.21443	37485.49424	40090.8137	44334.93206
O35309	Nmi	N-myc-interactor	89.153	13.69	35236	2	8403.700264	7865.951219	7737.733645	7167.87607	8543.252402	5613.99147	6831.476864	6779.172274	8783.932011	6900.280534	7245.617931	8116.635704	8915.63746	7450.288838	6750.89022
O35316	Slc6a6	Sodium- and chloride-dependent taurine transporter	23.147	2.9	69856	2	6444.583574	6892.853597	7670.94849	9158.332876	8548.51048	7992.771283	8110.598925	15393.91652	15171.12574	11034.25555	9854.307809	8582.82355	7540.801877	6863.673699	10187.90472
O35326	Srsf5	Serine/arginine-rich splicing factor 5	49.024	8.92	30891	2	102740.2156	93251.40693	111019.5468	111612.1112	99864.9088	100943.0082	101518.4232	103713.949	104097.8465	88348.15769	95393.369	102240.4311	108142.6601	86686.63396	101771.3155
O35343	Kpna4	Importin subunit alpha-3	134.512	13.63	57923	5	48704.66822	38664.84677	63555.2016	54716.31754	47456.23864	29522.58473	56096.70369	62959.27518	65366.10851	30854.25491	47043.31915	45071.70861	42734.17497	44266.68126	48096.42636
O35344	Kpna3	Importin subunit alpha-4	211.239	15.16	57773	5	31438.46029	43897.72081	43374.92375	47740.50098	46441.70364	54357.77439	60651.3334	65457.47986	53537.1917	50163.5908	45047.89528	49747.38566	52120.37874	45862.89248	50684.85732
O35345	Kpna6	Importin subunit alpha-7	106.416	8.58	59964	3	7460.152475	10148.98024	10058.52083	8397.742895	9362.546237	8742.646026	10036.15984	11583.20612	10640.4153	8643.890537	9999.588955	10062.2279	7577.077141	9269.609996	7812.956577
O35350	Capn1	Calpain-1 catalytic subunit	199.376	16.83	82106	8	46764.44178	48541.29361	45719.6462	49192.5022	44429.50014	31044.48189	47810.81583	43388.32956	35628.9296	42552.10299	45581.55806	47773.18303	40249.01446	33629.98716	42769.51454
O35381	Anp32a	Acidic leucine-rich nuclear phosphoprotein 32 family member A	234.908	25.51	28538	8	363948.2856	272991.0541	340428.1987	298048.1296	320601.8944	222746.0631	275804.3667	225191.3292	273727.8996	249687.6895	279477.9534	294400.0604	276290.5626	334761.5805	294519.3661
O35382	Exoc4	Exocyst complex component 4	240.907	10.15	110545	7	18305.48817	24614.41154	18322.13983	17918.55617	14207.01324	16205.62969	11944.89892	22026.24839	19776.59991	15377.47479	19491.88728	19615.66987	16461.86392	14347.72938	13369.00264
O35435	Dhodh	Dihydroorotate dehydrogenase (quinone), mitochondrial	55.499	11.9	42700	2	3613.34273	3572.200151	4966.831086	4277.690755	3742.867222	3309.769495	3869.63824	4260.174575	3216.57415	2879.550081	1023.15025	5197.343393	4189.80808	3510.688538	4173.314394
O35459	Ech1	Delta(3,5)-Delta(2,4)-dienoyl-CoA isomerase, mitochondrial	99.792	7.03	36118	2	4102.762915	4306.964953	3470.003362	3760.305832	3086.044754	3831.763779	3591.454903	3188.013686	3322.553428	4266.631561	5493.266383	3474.084355	4382.671761	3667.219611	4751.048782
O35465	Fkbp8	Peptidyl-prolyl cis-trans isomerase FKBP8	239.618	24.13	43529	6	48640.61386	55559.34017	49244.15951	52644.07457	50377.23613	54753.29329	52163.832	58841.92098	52449.52375	48111.08986	51064.09554	52309.89156	51453.05325	53590.40043	63085.01206
O35495	Cdk14	Cyclin-dependent kinase 14	62.015	3.41	52996	2	1613.716891	1531.455805	0.009509917	1820.8997	1331.9565	1907.263423	1494.565148	1899.544419	2146.174149	2075.22165	1989.844437	1123.166297	1560.91759	1664.01747	1370.712908
O35509	Rab11b	Ras-related protein Rab-11B	284.8	57.34	24489	11	503272.6597	499734.6012	483091.6059	501161.1304	502336.4907	506993.922	512924.9033	497842.2221	498533.7303	522081.8088	526637.224	479367.345	533580.1861	545834.343	514391.1326
O35522	Psmb9	Proteasome subunit beta type-9	88.912	26.03	23427	4	104274.5423	100427.7084	106939.64	105195.9887	114456.737	96459.82099	101160.8917	99964.06957	104913.0884	96858.65155	101201.9193	104077.3408	109313.6341	108666.6566	96699.99512
O35550	Rabep1	Rab GTPase-binding effector protein 1	27.948	3.25	99428	2	6288.795826	5290.666027	5181.300219	6398.813732	4562.714871	3783.984835	5807.910303	7548.003622	5939.926802	6157.021231	5939.361444	5249.570371	7019.51945	9119.932121	6027.209264
O35593	Psmd14	26S proteasome non-ATPase regulatory subunit 14	213.025	36.13	34577	5	69209.88668	70664.61139	67243.6421	66149.32715	66341.416	69671.94172	72141.37862	73351.4656	77332.43155	69739.35707	68452.82543	74127.79615	71759.52255	70362.3062	67187.44696
O35601	Fyb1	FYN-binding protein 1	254	13.92	90055	9	44146.93705	51145.92571	54788.92164	54260.37612	48076.26485	59323.01775	65730.55716	57310.01251	55452.1306	56971.58495	45898.74797	51838.51622	54700.31676	43675.54183	45595.74296
O35604	Npc1	NPC intracellular cholesterol transporter 1	62.038	2.58	142885	4	54234.16404	48165.4718	34510.52046	94778.14691	29494.07874	25409.06101	29798.75114	33881.32513	40806.69714	30159.50726	34020.27146	33982.02296	48380.99531	49270.07783	51201.71778
O35609	Scamp3	Secretory carrier-associated membrane protein 3	184.82	16.33	38458	3	28458.28522	33162.08901	28275.20043	26463.65747	29562.74943	23371.83155	25320.0275	23891.75581	23990.70374	22588.36297	29830.21849	28416.26715	31287.48329	30177.48703	29038.85437
O35638	Stag2	Cohesin subunit SA-2	177.342	8.12	141281	7	31000.87272	27637.30761	28310.88402	37024.77255	26422.90092	26416.49827	29454.67943	28111.81006	28493.6111	31034.25393	41575.26924	26514.49689	30468.56627	30180.18209	18787.1975
O35639	Anxa3	Annexin A3	1159.261	73.99	36384	27	1196052.963	1200598.235	1067847.339	1110616.698	1145943.776	1034128.825	1029181.709	1012668.448	1048517.447	1085266.398	1183991.444	1115642.143	1179338.314	1160695.265	1167628
O35643	Ap1b1	AP-1 complex subunit beta-1	982.506	39.45	103935	25	312119.6419	329410.4867	318067.5678	313881.398	290494.3298	264462.0126	294434.0404	275082.1028	288442.5649	273336.8289	306938.1348	297210.2615	303215.7835	302618.8581	294349.4346
O35654	Pold2	DNA polymerase delta subunit 2	107.401	9.59	51355	3	15092.8636	9680.313706	19250.78565	15778.01026	15860.08791	13944.39551	15956.86493	14183.80882	14508.84588	14205.15349	14037.31715	16101.56446	14785.74997	13791.25925	16744.58696
O35658	C1qbp	Complement component 1 Q subcomponent-binding protein, mitochondrial	176.214	24.82	31013	5	50626.94067	51736.30837	40539.14244	49768.98861	49192.96173	66225.08598	58858.55106	64449.76758	59144.16718	52826.28284	42060.63337	54641.05948	48618.54372	46523.14829	52209.3978
O35682	Myadm	Myeloid-associated differentiation marker	99.873	13.12	35285	3	123425.1548	132645.9436	109123.8398	122615.9682	120498.2781	159119.6682	137521.8936	144480.836	155976.8048	153719.1794	120964.0336	118196.4832	130283.2884	116469.2053	123638.1973
O35685	Nudc	Nuclear migration protein nudC	347.061	32.83	38358	9	153187.6988	165575.0913	149641.3282	160764.4302	173080.6305	169277.4919	189604.9542	212145.6221	185266.7775	181248.5692	166656.415	182348.8508	167261.6805	169881.959	188103.3205
O35691	Pnn	Pinin	55.561	3.17	82436	2	3901.400982	4326.839926	4021.222524	4477.540292	3526.526963	902.6324201	980.3028798	1066.882406	1000.561831	1933.695263	2104.66646	2317.10329	2491.747124	2072.562358	1972.708802
O35737	Hnrnph1	Heterogeneous nuclear ribonucleoprotein H	631.314	41.2	49199	14	533501.8096	540762.4609	583309.4746	590787.575	559922.8123	494711.9789	492346.1712	505183.9403	471700.3464	478029.3399	537034.7769	511608.985	477684.7343	514976.6803	499053.9033
O35841	Api5	Apoptosis inhibitor 5	434.704	30.36	56785	10	136539.1262	130623.194	123306.4267	114193.1624	121295.7765	124550.6699	133514.6779	114495.5477	118676.8515	100991.779	129248.6607	127457.311	133547.1335	110512.5435	134475.9909
O35855	Bcat2	Branched-chain-amino-acid aminotransferase, mitochondrial	337.251	36.64	44127	10	143841.0517	151448.5593	136619.17	134563.4033	136024.6609	150399.5398	135589.2957	138855.0551	147021.3959	124470.8462	118564.4621	129139.8958	134180.4619	129284.8529	130935.6707
O35857	Timm44	Mitochondrial import inner membrane translocase subunit TIM44	305.213	18.58	51091	8	41088.87504	48621.48547	48640.90265	53272.17136	41654.41158	34084.25959	53625.45297	51666.40854	53826.32298	51843.77587	44385.86394	50348.28291	46232.69858	49088.431	44538.26559

O35864	Cops5	COP9 signalosome complex subunit 5	113.598	20.06	37549	6	68889.41334	76681.79869	70412.93146	74244.94922	78083.22981	70837.10309	79089.78473	73122.75032	75154.86023	71470.16218	67816.20871	77417.98141	75125.14327	74370.59797	74309.66358
O35887	Calu	Calumenin	199.75	31.43	37064	8	75091.97969	67573.45884	61175.62054	72058.67734	71158.78752	106466.14	84362.15715	91645.17511	96126.45424	70223.53254	80796.85578	71026.99158	68694.00403	62743.00714	75583.40946
O35892	Sp100	Nuclear autoantigen Sp-100	15.562	4.56	54727	2	14486.63925	9973.78081	12686.96573	16443.70743	11300.47996	17618.86185	14288.04738	17720.54308	12075.10446	15255.5085	15689.58128	15668.22806	10130.45155	16088.18523	15092.79207
O35900	Lsm2	U6 snRNA-associated Sm-like protein LSm2	101.258	28.42	10835	2	13696.00513	12761.09534	10445.96437	11671.54085	11453.78788	9990.600645	10576.61779	7028.590454	10779.66727	11601.63558	10941.00008	11856.501	12793.63904	11963.01429	11339.66121
O35955	Psmb10	Proteasome subunit beta type-10	214.461	22.34	29063	4	58833.48756	51019.74302	47943.37873	54685.86489	53716.58942	53190.59563	55594.89875	54357.60237	56538.42709	49027.89501	56939.85357	54037.70322	55153.38165	55532.47727	51846.71588
O35964	Sh3gl1	Endophilin-A2	113.857	13.59	41492	4	48640.04213	46094.51939	41773.13506	37002.59911	44351.70188	31155.78042	36954.85858	31349.07109	31597.55528	26603.34204	41014.35325	36239.32522	41557.22567	42731.43507	34253.36344
O35972	Mrpl23	39S ribosomal protein L23, mitochondrial	53.308	25.34	17122	2	8189.40937	3799.502508	3770.26146	9168.696223	6707.670135	5034.925783	6282.281195	4756.855773	6559.492254	6494.862397	6722.013709	7250.595235	3204.027803	6833.412231	6458.998432
O35987	Nsfl1c	NSFL1 cofactor p47	378.771	31.08	40680	8	107174.9507	115927.5902	107765.7761	98696.15244	101580.9507	85149.47096	96732.94494	80584.58561	84036.49398	94139.86154	116788.5607	99167.62673	96436.00053	96365.39613	96761.80537
O54692	Zw10	Centromere/kinetochore protein zw10 homolog	68.856	4.88	88063	2	1916.158232	4397.227111	2627.358676	3664.375813	3090.507037	885.7265591	2212.265192	3154.555197	3111.579004	2602.372376	3461.283363	2501.771978	3546.011122	3420.695201	3762.952847
O54734	Ddost	Dolichyl-diphosphooligosaccharide--protein glycosyltransferase 48 kDa subunit	568.017	50.57	49028	15	347296.8667	350428.376	342539.5525	335502.1648	349227.1921	456906.3485	433003.9984	425131.0773	442426.7466	437688.7809	358741.9258	372391.4234	389216.7262	395830.3191	361921.9782
O54772	Smarcd2	SWI/SNF-related matrix-associated actin-dependent regulator of chromatin subfamily D member 2	71.472	7.91	59128	2	8875.578283	12671.86734	8466.768673	9796.081106	9268.542757	7770.165136	9935.466491	8557.76678	6235.072227	8290.674945	11194.25917	10402.55519	1871.127105	9575.563954	8083.011197
O54774	Ap3d1	AP-3 complex subunit delta-1	96.886	5.34	135081	4	19319.93517	17934.26128	17663.24044	17329.08577	15343.34567	13201.16458	17280.35895	17129.73576	18706.98756	15282.57512	12145.50302	13899.84146	15228.52496	15605.6034	16723.51129
O54782	Man2b2	Epididymis-specific alpha-mannosidase	109.642	6.39	115610	3	12811.3841	16465.61368	9684.222363	12485.30892	10772.94975	5862.544397	6227.306159	6123.892326	6862.099533	7879.618172	13130.14455	12666.16916	14841.70547	12297.44483	10926.00474
O54824	Il16	Pro-interleukin-16	73.99	2.27	141435	2	8600.384744	8577.539561	5674.170711	4634.322006	4197.396721	7236.354139	0.012348474	0.01388636	0.012083387	2959.357381	6215.360761	3749.685792	2719.528439	0.00945634	2487.664401
O54825	Bysl	Bystin	36.418	6.65	49784	2	1718.215494	2050.062906	2095.043424	2175.222446	1748.062815	1751.082787	2503.291745	2544.722249	2749.138176	1920.482903	1972.830642	1940.129868	1797.438089	1795.620309	1415.3001
O54924	Exoc8	Exocyst complex component 8	44.609	5.03	81043	2	8218.868905	7439.505652	8137.922859	7784.420754	8111.028571	5859.277014	7689.831647	7392.0555	6899.175896	6366.215566	8916.953085	9071.546198	7578.931532	8860.699526	7190.340916
O54950	Prkag1	5'-AMP-activated protein kinase subunit gamma-1	230.611	31.21	37520	6	33409.35039	10473.03259	21404.01878	11193.54522	19615.95575	29537.48802	22072.10869	7368.878263	9874.742423	20751.01302	28529.78612	9007.484321	7478.557149	21049.56846	16101.50295
O54984	Asna1	ATPase Asna1	98.575	8.05	38823	2	28603.38338	30543.50804	27492.24892	32187.43496	29046.33783	31064.09244	30830.47232	30027.2077	30961.88152	28527.77437	27641.65035	28437.72357	30399.554	30637.54504	31088.13442
O54988	Slk	STE20-like serine/threonine-protein kinase	256.181	9	141457	9	68759.82799	74734.63546	71341.88397	77192.14299	68474.66888	51236.27211	52052.10301	57633.96739	44507.10146	56808.91217	61015.237	64013.89716	69621.5378	57938.12278	60967.72106
O55023	Impa1	Inositol monophosphatase 1	249.78	32.85	30436	7	149633.2967	161967.6073	159794.9054	133249.3951	150299.229	113677.8515	128182.6191	123935.2311	122400.0209	115945.4071	147212.4321	137075.172	139437.3745	135817.7934	136951.2913
O55029	Copb2	Coatomer subunit beta'	596.274	26.52	102449	14	236755.4198	251320.8612	221556.741	229331.7647	239437.3094	237228.154	255257.4196	256966.0415	254997.8283	228459.2277	240927.1648	247228.5445	227592.0692	241631.9357	241627.5536
O55043	Arhgef7	Rho guanine nucleotide exchange factor 7	125.311	9.6	73140	5	33763.33165	35695.03318	28639.01896	26352.0566	32878.77233	29908.47646	31747.81577	27256.68804	30017.21442	29353.38953	30543.19374	29288.89277	30633.01343	28013.85998	32815.28322
O55047	Tlk2	Serine/threonine-protein kinase tousled-like 2	46.593	2.37	82261	2	2528.013517	2911.725497	3041.180357	2872.093616	3288.79619	3446.933673	2617.227226	2160.205274	2836.173483	2337.604956	2256.935561	3385.430071	2551.014378	3065.30142	2852.766934
O55098	Stk10	Serine/threonine-protein kinase 10	145.935	5.69	111906	4	32541.1409	32701.32236	29847.50599	29484.96521	30252.39897	28860.44771	32961.76338	27055.41567	28632.83588	28177.11046	27598.21175	31010.16516	30142.83374	19850.10034	29245.01112
O55125	Nipsnap1	Protein NipSnap homolog 1	182.332	33.45	33363	5	51999.89959	50731.87966	47397.39535	45965.06702	50858.75961	47672.73084	37042.29504	40184.14236	43464.64351	42662.93361	53514.81406	41376.73626	46402.55683	55180.11041	44521.86505
O55126	Nipsnap2	Protein NipSnap homolog 2	84.772	21.71	32933	4	19685.386	32179.42493	16149.77274	16816.99313	18081.08701	13547.3082	14168.76884	14337.71481	12698.90045	11793.75929	13218.6944	20345.19689	13629.79747	20793.73429	15123.46667
O55128	Sap18	Histone deacetylase complex subunit SAP18	51.533	24.18	17595	3	22867.11451	30337.29231	26324.56182	30620.68259	28823.89737	24320.79701	28535.36332	25302.46414	28989.72174	23210.62216	27439.33315	22495.02916	2841.063086	26577.77533	27351.67088
O55131	Sept7	Septin-7	479.287	43.35	50550	15	183243.7008	191115.2446	160562.4128	178921.748	184533.722	157132.601	172067.1327	179574.3326	169529.6765	166422.4019	180869.13	171386.4635	178460.7668	176075.8425	170386.7387
O55135	Eif6	Eukaryotic translation initiation factor 6	213.179	30.61	26511	5	136890.0781	137495.6452	128503.473	103037.5053	118301.1054	125305.9979	122617.5323	128117.136	120495.2079	115881.337	106870.9447	123673.6736	96407.0269	123777.5658	119767.8772
O55142	Rpl35a	60S ribosomal protein L35a	50.285	27.27	12554	5	242272.3294	264723.8213	247384.5737	278058.572	294663.3315	315193.8154	291734.0959	309158.507	305370.0124	294534.3346	274913.7982	291631.5222	291895.8772	295546.4995	292159.4083
O55143	Atp2a2	Sarcoplasmic/endoplasmic reticulum calcium ATPase 2	625.662	20.69	114858	16	149389.7478	151301.2434	144904.6475	144343.2481	151449.6381	175381.1834	170141.6845	180312.2955	194950.5971	151077.7864	126036.4088	130881.1256	135284.2265	143388.9361	141557.5739
O55166	Vps52	Vacuolar protein sorting-associated protein 52 homolog	137.297	6.5	82103	3	543491.0815	533071.4236	553398.0073	558030.0436	545203.6319	493446.2603	535991.4084	541695.6232	515583.0509	584416.5539	536135.4123	559590.4827	563521.117	568694.6657	569382.3593
O55173	Pdpk1	3-phosphoinositide-dependent protein kinase 1	52.966	4.47	63593	2	7661.606132	7609.824488	5594.506132	6131.443366	6699.040082	3958.317248	5355.454494	1496.85992	1686.194712	4136.387572	5578.849121	2523.380685	5920.507879	3846.843964	2220.956351
O55201	Supt5h	Transcription elongation factor SPT5	105.897	5.91	120664	4	8774.614413	9793.092011	9627.405931	10221.81061	9873.226501	7404.346473	9105.131199	10353.39193	9861.123661	9310.324463	9153.253333	11797.18793	8722.062144	7522.138106	7886.046105
O55234	Psmb5	Proteasome subunit beta type-5	72.572	13.26	28532	3	6192.029444	6736.991718	6935.147727	6640.872952	6529.537238	0.014539799	7180.33978	0.01388636	6390.763646	6399.598885	6531.152606	7638.573332	6127.416557	6501.49646	6785.977688
O55242	Sigmar1	Sigma non-opioid intracellular receptor 1	83.285	13.9	25250	2	6797.529884	3297.641469	7994.169187	6476.24646	3483.761971	6009.797088	6866.736388	7860.712288	8702.739203	6080.113336	6330.739878	8176.123127	7501.12626	9347.413212	6794.249859
O70133	Dhx9	ATP-dependent RNA helicase A	1082.594	27.17	149475	30	367874.1561	415823.307	401381.4821	422711.4732	419856.6423	339163.7507	375517.8505	343841.384	372313.4017	324548.9781	333447.004	388257.6828	328308.4544	385741.0477	368614.3515
O70145	Ncf2	Neutrophil cytosol factor 2	441.898	43.62	59485	17	272852.7932	293447.8121	291695.8439	271442.7579	282258.8231	253669.4394	274534.6966	207267.955	242946.1965	230416.0697	239220.2936	270720.2982	258438.6883	254785.4394	275038.5711
O70152	Dpm1	Dolichol-phosphate mannosyltransferase subunit 1	34.675	7.31	29175	2	11273.38536	10398.12308	4954.559441	2391.239446	9495.170911	10853.91389	12120.02733	2702.987935	12547.53849	11777.22561	11796.1053	12455.62191	12236.67186	11582.68622	12945.12691
O70172	Pip4k2a	Phosphatidylinositol 5-phosphate 4-kinase type-2 alpha	73.489	8.64	46152	3	18780.41508	20532.6109	16458.68934	14674.42614	37291.38953	14150.42723	15734.2559	15096.78662	12807.30261	14032.8985	13680.1144	18161.83275	19509.69459	14834.20722	14505.03398
O70194	Eif3d	Eukaryotic translation initiation factor 3 subunit D	362.658	33.76	63989	12	115647.7964	128809.9839	119471.6476	129601.3777	109931.576	124348.1014	133684.6423	123476.4257	131786.5892	114945.0211	121175.7671	126645.7352	126486.517	127262.2931	123152.874
O70200	Aif1	Allograft inflammatory factor 1	194.268	21.77	16911	2	9070.705929	11986.03754	11516.65864	10820.42689	10285.13285	9893.815525	4528.168472	9976.450175	7512.860183	8359.496221	9671.403611	8606.454812	9861.026461	9551.809014	10588.7939
O70251	Eef1b	Elongation factor 1-beta	229.088	40.89	24694	6	174953.1178	176496.7018	189952.8888	182302.1985	172101.771	219011.3411	210064.7952	236628.1627	202524.9957	187715.2269	182412.4818	197421.5219	183050.3932	171003.8591	177915.4048
O70252	Hmox2	Heme oxygenase 2	241.671	26.98	35739	5	40326.7462	45784.40741	35909.74077	41180.73612	41604.99828	42900.23797	43632.42561	42075.65547	40878.52683	46532.60775	31434.95643	45122.00302	43376.91261	40354.52737	41967.25309
O70310	Nmt1	Glycylpeptide N-tetradecanoyltransferase 1	562.146	47.98	56888	15	225123.1842	213199.2227	217649.23	210417.684	223766.5799	213381.4436	233548.9998	239457.2585	237672.209	228687.0029	230190.5518	234746.3658	216040.2365	226605.4717	227786.8451
O70311	Nmt2	Glycylpeptide N-tetradecanoyltransferase 2	138.266	12.1	60484	5	70368.9033	78040.71942	74805.35312	69014.93019	76891.90462	79119.55443	76149.11911	87205.10886	78698.43428	80026.7905	70041.00384	68534.1126	64192.80949	81383.74567	80180.32115
O70318	Epb41l2	Band 4.1-like protein 2	640.715	34.21	109940	21	228095.8199	217218.4349	208514.2506	190212.7156	182470.6297	197938.6499	183718.0415	180936.8484	183709.9539	205664.0722	208748.7553	207551.8642	212072.5079	220490.4918	222073.6347
O70370	Ctss	Cathepsin S	545.861	51.76	38475	11	389779.5379	394795.3749	369624.5202	402537.4664	357684.7859	285323.8331	299836.2508	270911.6125	264616.5668	291910.2311	297238.0779	366876.7726	358572.3435	376690.7255	382453.2159
O70378	Emc8	ER membrane protein complex subunit 8	29.896	10.14	23348	2	9701.558906	10894.39243	15229.63236	11062.69764	11003.88855	11353.7413	10064.43167	9880.349624	12825.46847	10082.34505	11770.65447	9427.624437	9667.110831	4363.041902	8352.929215

O70404	Vamp8	Vesicle-associated membrane protein 8	106.163	30.69	11451	3	73924.10331	76094.55442	67899.71704	66213.94674	77765.77949	67654.3628	66167.0078	30222.46727	66962.82538	36155.82916	73147.56601	78179.1632	85922.55787	69862.97365	71924.73711
O70435	Psma3	Proteasome subunit alpha type-3	399.733	37.25	28405	8	248671.1349	251047.0412	251386.0236	260080.6593	270301.2166	234330.372	250357.0759	246561.4922	237976.2753	236510.5348	267942.7654	263204.8122	249312.6339	265506.8859	251204.8263
O70439	Stx7	Syntaxin-7	430.805	44.83	29821	8	139418.6068	150411.1169	134815.9945	138207.6147	150125.96	115212.2147	118960.186	117464.7569	110002.3089	119595.8843	130335.0482	129243.7451	134139.2532	129713.7451	137594.87
O70454	Bud31	Protein BUD31 homolog	46.673	12.5	17000	2	8439.244737	8204.965808	7910.225717	9907.880298	8959.18472	7497.221332	8842.683645	8232.664468	8518.339167	9058.453049	8749.653914	9559.051745	8880.639518	10055.98028	8848.973014
O70456	Sfn	14-3-3 protein sigma	110.508	16.13	27706	7	1000864.467	970155.88	1005264.322	962494.2763	1094754.899	1078826.96	1116554.473	1058920.134	1064293.517	1015722.767	998809.1183	1069851.898	1161584.281	1146421.564	1105105.952
O70469	Dok2	Docking protein 2	60.094	4.85	45522	2	15861.97454	14814.71565	9007.071067	13556.33333	16696.54014	9568.303948	13665.97293	7993.830402	14224.45279	15362.47793	14901.3388	8800.374845	11011.30359	8226.91087	17061.09251
O70480	Vamp4	Vesicle-associated membrane protein 4	107.302	31.91	16353	3	8280.583901	10684.02021	9774.70685	8911.383119	8869.016598	12318.86323	9502.279735	9538.146331	13419.46511	14479.07481	8734.152485	12562.00843	13495.29038	7588.269882	8559.526284
O70492	Snx3	Sorting nexin-3	216.177	62.35	18757	8	162265.2788	150729.3887	139896.9722	133969.4551	159117.3795	127978.8492	127196.754	109408.6687	118051.0166	125767.0736	142750.0158	142169.7971	142782.6068	142140.9357	144036.6973
O70493	Snx12	Sorting nexin-12	73.96	29.7	19116	3	65089.63614	56930.47798	65128.95029	54853.53559	69145.05483	59671.38771	60454.08505	51425.51839	55533.8143	57765.78777	64894.79468	59766.93623	65483.64192	64318.10707	66822.04543
O70503	Hsd17b12	Very-long-chain 3-oxoacyl-CoA reductase	331.683	42.31	34742	10	108287.2335	110549.7296	103036.0757	103533.6803	95815.23306	115014.5025	130016.4046	122551.0804	125209.4446	126746.8133	108070.9959	114792.9386	110179.1832	111923.9968	109869.1414
O70551	Srpk1	SRSF protein kinase 1	79.132	4.17	73088	2	7204.264582	6917.599221	7309.867005	5992.198499	7677.793165	6309.574602	6330.708392	9212.945149	9362.817654	10618.77388	7899.639066	6654.244686	7446.372043	7787.502523	8467.17871
O70593	Sgta	Small glutamine-rich tetratricopeptide repeat-containing protein alpha	62.997	7.96	34157	2	9543.694503	7871.189137	8273.677549	9583.395188	9779.963848	5221.67315	5115.134665	7159.951944	6367.37468	4494.950753	9608.373984	8517.726349	7749.703534	4801.472416	6233.453456
O88339	Epn1	Epsin-1	34.239	5.74	60158	2	8999.082312	6612.240218	6658.101863	6585.254794	8051.449789	5332.413278	4794.258023	3980.085801	5135.080313	5652.527877	6919.434134	7802.488128	8704.509209	6720.215117	7438.83095
O88342	Wdr1	WD repeat-containing protein 1	1015.99	62.38	66407	24	1042079.526	990616.6915	913664.1886	969942.313	999272.7883	849554.3879	945796.4149	902285.5537	874182.5939	841086.2192	979526.1604	967300.5076	1018522.426	971628.9181	976572.3856
O88351	Ikbkb	Inhibitor of nuclear factor kappa-B kinase subunit beta	21.733	1.98	86690	2	34230.46616	33799.09019	35607.76998	32919.22942	39017.44966	43307.62861	31532.2145	35845.83188	39951.33835	33430.07269	36647.06176	33345.06011	36177.15303	40757.16413	35973.17447
O88384	Vti1b	Vesicle transport through interaction with t-SNAREs homolog 1B	104.755	15.09	26713	3	50997.29029	59333.04988	44845.23782	43282.01938	48009.96026	27326.79331	40047.04924	36050.27381	27519.50887	20360.89472	44207.41444	48131.42992	52566.33514	47157.86253	49475.64647
O88441	Mtx2	Metaxin-2	93.071	28.14	29758	4	28326.06745	30792.30207	19330.77951	23826.69692	23335.3691	22461.90364	24538.53271	16852.69074	19940.27391	17531.21082	24691.67153	18007.51234	24481.28375	20376.38819	16275.11504
O88447	Klc1	Kinesin light chain 1	63.553	9.98	61450	3	10208.32687	10654.13448	9292.836384	8715.949789	3579.096007	9640.777578	9097.481319	3911.669027	3801.592475	10663.99345	10681.49094	9721.456804	11380.52477	10536.81269	9373.598611
O88456	Capns1	Calpain small subunit 1	288.071	47.58	28463	9	88897.55549	84890.64969	67609.17956	79752.87103	79800.68518	66372.09062	60921.28339	66801.15178	79990.76459	79724.84851	89715.81444	78652.30921	79126.12823	84155.73923	76522.16682
O88487	Dync1i2	Cytoplasmic dynein 1 intermediate chain 2	368.398	23.69	68394	7	35961.15344	38408.27291	27363.04243	36327.20006	30389.15554	21942.7183	34007.55257	32552.73032	38407.07546	32386.24624	36488.94155	39101.22458	31961.70633	30657.24496	35709.05637
O88508	Dnmt3a	DNA (cytosine-5)-methyltransferase 3A	225.665	8.15	101672	5	26237.0225	27078.08398	24269.17385	27043.90173	20448.1521	23827.86873	16879.83002	28126.32998	30019.95735	26061.36861	25797.80295	28540.1057	29847.78696	26070.00535	25734.02651
O88531	Ppt1	Palmitoyl-protein thioesterase 1	424.343	35.29	34490	10	464342.7782	412613.3338	293926.0146	355745.0283	355686.8947	268546.9692	297834.9089	264191.642	266017.6113	290526.1038	392587.9399	294585.3517	397748.6495	389695.462	370713.5791
O88543	Cops3	COP9 signalosome complex subunit 3	188.945	25.3	47832	6	56521.44311	59963.29405	53188.87327	50317.48939	54262.38822	44602.87029	53803.83902	49548.19714	49507.43654	49179.38217	48990.93118	53585.90494	47847.75991	47542.91995	50801.76944
O88544	Cops4	COP9 signalosome complex subunit 4	489.518	40.15	46285	11	87980.13812	93125.44734	83114.05389	105251.1529	83711.51928	79971.5794	103585.99	109489.4526	68912.14152	94575.69805	106074.2524	73748.37816	88314.78054	67761.56695	100744.795
O88545	Cops6	COP9 signalosome complex subunit 6	44.237	13.58	35880	3	9800.203978	25100.20222	22937.59283	32022.81594	4562.387336	26246.75338	6714.492792	22559.74497	27531.59527	25412.90694	26233.05196	9904.160088	22691.24252	21930.08843	24561.42937
O88569	Hnrnpa2b1	Heterogeneous nuclear ribonucleoproteins A2/B1	1252.754	68.84	37403	26	1444429.324	1382914.526	1552060.383	1535065.646	1442598.841	1043050.213	1272160.164	1172549.881	1171723.816	1268102.8	1373371.619	1318469.403	1372729.858	1482031.747	1247509.879
O88587	Comt	Catechol O-methyltransferase	81.166	8.68	29486	2	13788.99151	10797.15962	9357.995343	11641.82647	9690.489024	7145.613068	8909.978198	9813.044176	9848.914909	8414.097332	8196.05513	7251.183362	10091.46883	7317.304334	9525.201293
O88668	Creg1	Protein CREG1	88.14	13.18	24452	2	37496.59893	39238.04006	42547.07117	46024.02805	37987.32526	34541.81618	43085.22459	35652.64485	34474.83928	33524.51282	37597.4889	39780.89194	40585.75559	39645.77708	40919.24255
O88685	Psmc3	26S proteasome regulatory subunit 6A	481.329	48.42	49549	14	128408.5207	113106.9604	101792.6825	137397.1643	111242.9959	134610.9897	121133.5174	106165.1901	139370.7753	107969.1327	136363.0566	123539.5371	99550.36605	116829.3973	110922.2881
O88696	Clpp	ATP-dependent Clp protease proteolytic subunit, mitochondrial	52.788	12.13	29800	2	18264.06446	19334.84714	16587.53921	17333.14857	15925.08649	18122.53504	17322.56295	16054.27541	19714.29645	16896.28413	21998.89454	19836.0215	20682.84681	21924.81179	14725.32751
O88712	Ctbp1	C-terminal-binding protein 1	152.517	23.13	47745	8	155295.4011	161167.6701	152572.5891	162515.3956	162535.7916	152827.3519	144557.3132	159661.8208	162459.2035	162818.279	147891.904	161040.271	157802.3067	158170.8687	159870.9883
O88736	Hsd17b7	3-keto-steroid reductase	103.985	8.68	37317	2	4175.086264	7745.688958	7740.73759	4629.957432	5303.776484	11023.07449	9465.117743	9734.436063	8451.943977	5065.11556	8393.190429	7474.097038	7120.868909	8298.281979	7661.267801
O88738	Birc6	Baculoviral IAP repeat-containing protein 6	275.402	3.17	532170	10	27508.57537	33278.24015	26585.23579	32542.22827	32637.95893	30520.0495	25682.39193	29654.1333	29049.1569	28432.85805	28388.7907	29523.10185	30577.50681	26161.50253	33108.44934
O88746	Tom1	Target of Myb protein 1	200.291	21.14	54325	6	53758.54612	50371.16756	45729.70769	45190.67737	44769.83448	38261.11141	44724.01528	42062.7481	39824.14862	42591.71723	49068.59524	50363.1774	52532.53912	46538.71815	45997.10032
O88763	Pik3c3	Phosphatidylinositol 3-kinase catalytic subunit type 3	28.945	4.4	101534	2	4091.985346	3258.698091	3605.62634	3362.409476	4143.992536	3793.035424	4484.086704	4263.715597	4983.224644	3740.535727	3923.807727	4826.250603	3765.196209	4165.427761	4475.161558
O88796	Rpp30	Ribonuclease P protein subunit p30	55.805	7.09	29473	2	6794.182794	6232.645253	6305.649439	6854.423247	4510.32999	3389.483727	6026.433531	4757.096146	5197.790433	6245.065602	5117.572957	5830.37657	5683.835792	5441.293082	6447.467961
O88829	St3gal5	Lactosylceramide alpha-2,3-sialyltransferase	33.56	8.94	47360	2	2856.977962	6418.272801	5055.114313	7701.179232	6959.29528	6658.982339	4621.450018	7566.892543	7126.653206	4957.588351	5906.041738	5902.232677	6012.329646	5635.6628	5656.181402
O88838	Spsb2	SPRY domain-containing SOCS box protein 2	11.784	6.82	28938	2	13173.18656	36502.45718	14860.73434	11992.9972	25471.34764	32178.13915	16607.16954	26583.40963	11774.07104	12170.08947	36619.65163	39554.85241	43787.31181	35970.13334	32097.97459
O88844	Idh1	Isocitrate dehydrogenase [NADP] cytoplasmic	570.313	39.13	46674	14	435753.6963	449509.8155	443980.8911	467251.3416	445680.0453	350899.3754	379597.6876	393642.4518	360151.7386	384121.6493	421457.4037	439981.1641	454785.5425	444800.9572	457500.51
O88879	Apaf1	Apoptotic protease-activating factor 1	113.035	4.08	141003	4	7384.271195	5765.625889	5448.295336	6897.456358	5230.391693	5508.676504	5575.62081	7014.386375	8623.941927	5871.595001	6431.007817	6616.132702	5490.265382	7245.845754	6731.018941
O88958	Gnpda1	Glucosamine-6-phosphate isomerase 1	167.953	39.1	32549	6	43847.41207	45012.92757	38456.12816	43594.29546	36871.65335	25253.63601	27402.28586	32850.15531	24084.52067	28038.04438	50375.44261	49156.95397	46768.80387	42877.11471	32462.67421
O88983	Stx8	Syntaxin-8	143.776	18.22	26925	4	3028.522515	5301.085145	2596.776588	2446.228237	5277.582515	14146.71958	16921.73148	6268.766146	2084.46393	2294.699835	12883.21296	13323.85593	3108.30745	2800.953607	6092.294075
O88986	Gcat	2-amino-3-ketobutyrate coenzyme A ligase, mitochondrial	71.697	8.41	44931	2	9595.12658	9887.736589	9223.119749	8512.977216	7775.673871	7954.061829	9559.881477	9523.667232	9563.240201	7259.278753	9300.478696	7275.892425	9988.06021	7448.638707	8782.909817
O88994	Marc2	Mitochondrial amidoxime reducing component 2	143.384	18.64	38248	6	96696.99338	100443.3626	81915.44291	71747.85946	109003.2953	73849.70591	73397.5395	72465.40477	74980.30967	80161.39099	54964.38483	70494.98762	107477.7777	60730.05977	86819.87185
O89001	Cpd	Carboxypeptidase D	105.263	3.49	152406	5	35212.16206	34937.55894	35570.17966	34581.01276	36712.10987	41691.16278	44941.4256	41781.46947	41528.54961	41576.69235	32706.64095	31784.25794	40270.48467	37170.39343	34002.9185
O89017	Lgmn	Legumain	154.612	13.1	49373	3	91862.67225	83171.21348	58406.08531	73768.32238	70262.37165	28559.77011	47198.53835	40895.66901	39142.58216	43220.36345	62659.02825	63428.55559	62215.00877	59023.66802	61399.45516
O89023	Tpp1	Tripeptidyl-peptidase 1	205.19	24.56	61342	5	97024.28636	91762.75296	76291.99097	81491.30875	81814.19882	53769.89395	54483.14093	51826.25095	51825.19932	60921.29343	81544.61772	75141.42815	77857.44547	82414.92167	76129.62617
O89044	Prim2	DNA primase large subunit	90.342	4.93	58603	2	9650.73455	12703.97074	11872.58275	10805.07905	9720.375271	7458.989802	8227.745812	9642.60647	9580.80872	9668.634161	10272.18962	8721.666952	5288.580787	11103.35855	10996.0813
O89053	Coro1a	Coronin-1A	873.874	54.01	50989	24	3672251.895	3699815.455	3887122.322	3756627.862	3631625.103	3473775.844	3858290.82	3885258.102	3367489.526	3561630.019	3503946.709	3318470.482	3380935.523	3740411.717	3537423.205

O89079	Cope	Coatomer subunit epsilon	352.186	48.05	34567	7	40865.39019	45602.86027	41692.20567	43608.51067	41807.96231	39929.41526	47103.76999	38362.85605	35553.7649	31967.0766	30042.22156	34151.87414	34716.94067	42001.92102	30749.69587
O89086	Rbm3	RNA-binding protein 3	157.678	43.79	16605	5	34115.66148	21466.81123	31757.82255	25418.9931	27797.7757	11194.52054	21558.06332	20206.55554	21587.29392	17419.24133	33140.03124	29755.33195	31878.68801	26481.2261	25495.12825
O89110	Casp8	Caspase-8	162.529	13.54	55357	5	20542.86629	23378.04354	22695.13273	21687.72271	20726.29619	15334.25588	17753.41973	17587.40934	22395.53053	13482.23364	21991.59845	24197.08453	25457.15677	23690.4026	17766.11757
O89112	Lancl1	LanC-like protein 1	225.478	17.54	45341	5	33944.59895	31724.16751	22426.91739	30714.90192	31143.45443	28405.44287	27413.95208	28975.63188	26136.94586	21637.91435	30223.47524	31712.89205	31053.99688	20792.29929	29855.02209
P00173	Cyb5a	Cytochrome b5	126.291	41.04	15355	4	38399.46802	39871.53606	44536.76938	43419.62262	41001.28061	49514.14919	50506.50008	49360.55076	48231.13786	43164.26548	45863.62534	47028.5532	40211.4238	42280.5568	42557.33321
P00375	Dhfr	Dihydrofolate reductase	163.832	36.9	21606	6	45017.97434	45807.45765	51379.74937	52764.80756	51303.16112	45170.11876	49220.70904	45842.64987	45866.83895	42625.30408	48580.53556	51517.00065	52598.76735	52617.45184	45287.49984
P00405	Mtco2	Cytochrome c oxidase subunit 2	186.311	40.53	25976	7	59725.16472	61000.87261	47389.47359	64785.47398	52546.83812	46857.37524	36426.22658	29939.75488	48920.048	47772.81039	57435.01422	50540.98303	63671.49027	57784.57074	55693.43497
P00493	Hprt1	Hypoxanthine-guanine phosphoribosyltransferase	409.853	59.17	24570	10	264238.4545	281765.5738	257625.4234	278590.441	253299.6839	198596.285	226359.5206	192174.5653	224206.966	212015.3771	257247.5432	250923.9561	256867.99	253840.6565	253415.8221
P00564	Ckm	Creatine kinase M-type	53.894	8.4	43045	2	14311.66201	10115.37759	14627.07685	12532.85651	11080.81993	9240.513258	22480.8509	14879.19257	16690.02409	11916.1826	11138.50149	9373.943785	14418.01648	6963.493638	11187.79822
P01896		H-2 class I histocompatibility antigen, alpha chain	107.51	27.03	20454	4	234761.1423	219046.2103	183925.0276	184713.8135	214137.3833	271808.159	238268.4878	231869.4739	245384.3098	267354.8661	244194.4944	219907.2245	221987.0805	221630.0375	215660.2053
P01899	H2-D1	H-2 class I histocompatibility antigen, D-B alpha chain	255.44	28.45	40836	9	300775.9532	284979.3282	251421.5146	261122.1298	301392.8648	409545.3946	339294.5896	348631.206	354408.9881	381423.5122	318627.9751	301800.14	328544.482	320679.3887	307593.9218
P01900	H2-D1	H-2 class I histocompatibility antigen, D-D alpha chain	393.694	33.42	41110	12	412785.8667	406011.3751	343379.5825	361209.6487	404193.9307	536442.4828	465331.6614	457999.4238	477901.8306	516405.7042	431334.6428	410374.6865	442112.1469	433472.7541	409039.2468
P01901	H2-K1	H-2 class I histocompatibility antigen, K-B alpha chain	85.959	9.21	41302	4	265491.2925	254753.8349	209802.7873	225566.2379	256081.2115	353265.3206	303017.7611	295496.9399	309536.7509	335949.6196	273590.6125	259015.568	278401.801	277092.7743	265869.4366
P01902	H2-K1	H-2 class I histocompatibility antigen, K-D alpha chain	345.838	33.15	41490	11	254578.7954	265347.2366	217114.3162	251806.3795	268494.895	377330.4916	336294.5279	351074.0111	358607.3609	373527.3853	277370.2095	274321.9863	311233.1282	295762.4634	305857.2052
P02262		Histone H2A type 1	466.815	63.85	14077	10	16091889.14	15232084.62	16100043.78	15574715.09	16314933.9	18270634.73	16674355.2	17090545.68	16795951.11	18297070.8	15805338.62	14870157.42	16601302.27	13664306.04	17301139.9
P02301	H3f3c	Histone H3.3C	244.642	55.15	15315	11	17617614.52	18038387.95	19065016.32	19836384.03	19172892.84	20973416.41	18439241	18768337.85	18742133.49	21255499.23	19356494.77	18469476.64	19522586.6	19934927.39	19107943.52
P03921	Mtnd5	NADH-ubiquinone oxidoreductase chain 5	87.885	5.44	68475	2	6429.509349	5411.68642	3126.483128	5310.962639	4674.814879	3722.082802	4775.451296	4644.501238	4745.786817	4540.858777	4421.922659	4051.729878	4623.348253	3696.442064	4240.165655
P03975	Iap	IgE-binding protein	126.577	10.95	62747	4	24203.09605	29154.53806	22579.52304	25818.56892	17660.80941	20632.44429	24046.22374	29251.66147	24081.45451	16095.39124	15345.66116	16306.43967	15077.82016	16613.39742	24385.05623
P04256	Hnrnpa1	Heterogeneous nuclear ribonucleoprotein A1	841.217	49.38	34212	18	485966.3587	500638.0376	488821.9778	470920.8469	517809.7488	403888.1041	465222.6985	483211.6092	420676.1185	395341.9353	475094.3624	488564.1296	468279.4225	433254.5774	419998.3712
P04441	Cd74	H-2 class II histocompatibility antigen gamma chain	251.246	21.51	31557	6	139862.8289	150396.7936	143464.884	147093.0201	143189.0032	156834.2298	150511.0189	152378.939	168625.1504	163172.8225	153383.8437	147949.3969	160612.9349	159372.2588	148246.1242
P04550	Ptms	Parathymosin	83.296	11.76	11559	2	25172.9563	13430.02599	13686.83488	4310.81105	22082.22769	18632.31313	11174.54406	12670.87259	13003.34801	10993.58982	19491.13494	12902.72632	26151.73641	10396.27376	9597.513789
P04692	Tpm1	Tropomyosin alpha-1 chain	138.598	21.83	32681	8	540462.6714	367698.0295	484858.2062	489067.8564	452743.7177	431545.7687	479785.501	438887.4605	505052.4276	495470.1533	486163.6949	434966.1424	398877.6103	516333.7786	493861.9256
P05063	Aldoc	Fructose-bisphosphate aldolase C	1174.329	80.17	39395	30	4006973.451	3753982.921	3749252.864	3828756.465	3827635.73	3073012.677	3405920.574	3113646.204	2907326.7	3133873.848	3521674.326	3389887.271	3434211.045	3513232.917	3346338.001
P05064	Aldoa	Fructose-bisphosphate aldolase A	1657.605	92.31	39356	40	11928239.94	11788232.62	10950522.98	11133586	11665209.7	9688244.775	10210130.93	9959441.037	9489874.479	9800521.261	10707215.97	10237847.86	10697867.2	10444974.65	10611272.01
P05132	Prkaca	cAMP-dependent protein kinase catalytic subunit alpha	253.713	25.64	40571	8	108286.2957	108371.2774	108729.6728	109683.3914	117022.0712	113733.3063	118483.2015	121446.7815	123715.2372	109288.5771	110887.9111	110309.0922	121389.2586	120975.7564	112518.5693
P05201	Got1	Aspartate aminotransferase, cytoplasmic	819.476	50.12	46248	17	637248.468	673636.3433	607158.7499	649143.4908	634328.7019	580784.7998	668473.8897	616085.4066	611535.5346	580988.9501	636615.3826	600209.6328	664549.5304	645313.4091	661377.9689
P05202	Got2	Aspartate aminotransferase, mitochondrial	943.827	66.98	47411	25	1338992.713	1429739.552	1262160.363	1294326.963	1417763.704	1461399.442	1347146.224	1395827.212	1401597.105	1367917.141	1258891.263	1262041.561	1328076.605	1329357.101	1353072.081
P05213	Tuba1b	Tubulin alpha-1B chain	1739.652	89.36	50152	39	5904946.168	6744169.108	6611794.927	6825657.136	6462916.143	5966887.102	6497842.556	5471556.717	6445999.333	6419215.471	5951809.555	6630710.209	6645788.465	6624580.167	6818078.475
P05555	Itgam	Integrin alpha-M CD11b	968.769	29.31	127481	23	483369.6008	513416.7657	492410.1077	478056.8826	514439.6259	438091.6154	445175.5435	435078.3488	435179.5178	451248.3582	455189.4161	449687.1784	476731.8719	490871.4116	455009.037
P05708	Hk1	Hexokinase-1	866.042	31.92	102408	27	648657.2763	685724.532	629981.1824	663173.0783	722764.9283	641209.8799	622903.97	638992.1231	681254.2603	639638.7454	643070.2858	650379.5576	657277.847	710833.6628	673918.5073
P05712	Rab2a	Ras-related protein Rab-2A	311.354	52.36	23536	9	102471.8067	116447.0677	115780.2019	113742.5219	112670.9065	110368.3023	106521.6544	118954.305	107415.4007	116685.5699	104543.9278	106232.6096	108317.8951	109743.5446	111327.2768
P05765	Rps21	40S ribosomal protein S21	71.433	28.92	9127	3	20568.30006	21905.69036	15481.92452	12171.64801	20845.83497	15002.73028	16759.36602	15472.96636	18744.56689	16449.0755	23507.77749	22599.46047	19204.35423	17731.26679	16554.7388
P06151	Ldha	L-lactate dehydrogenase A chain	1682.741	84.64	36499	35	8217265.576	8792004.974	8585379.425	8550950.978	8893594.46	7565543.398	8029616.434	7931280.684	7533344.073	7711121.431	7256506.959	8102216.036	8157885.436	8354161.324	8123125.622
P06339	H2-T23	H-2 class I histocompatibility antigen, D-37 alpha chain	147.539	8.68	40875	3	115638.5418	110479.8982	94251.9551	102794.9388	117511.9473	174669.4312	150703.3711	153362.0377	150847.6849	166653.2674	125052.4844	116537.7369	148204.5896	130256.0568	119216.4647
P06685	Atp1a1	Sodium/potassium-transporting ATPase subunit alpha-1	1379.998	33.24	113054	29	1070626.094	1144754.816	968789.224	1028690.275	1059259.714	1091179.973	1051726.466	1030060.946	1099545.976	1067758.664	1116839.62	1081040.388	1147860.535	1104696.865	1140510.977
P06745	Gpi	Glucose-6-phosphate isomerase	1529.065	69.53	62767	39	2059905.701	2041316.466	2029275.345	2102741.554	2079254.341	1748598.982	1868822.231	1859687.61	1824612.314	1811778.163	2014666.341	1962044.924	2025673.602	2010582.123	1850950.606
P06761	Hspa5	Endoplasmic reticulum chaperone BiP	1805.756	54.59	72347	39	4095761.539	3799424.207	4227951.981	3726789.64	3603341.846	4616002.48	5060551.693	5276284.285	4355010.024	4678664.729	4129526.965	3886069.767	4109104.964	4365993.821	3906113.136
P06800	Ptprc	Receptor-type tyrosine-protein phosphatase C	1161.594	32.25	144837	33	698054.3799	748973.4915	620899.4394	723468.9027	707437.1824	633142.9665	651798.6091	624290.1343	640504.1013	672197.0652	666874.238	640184.8914	729892.6017	645171.3371	719376.3687
P07091	S100a4	Protein S100-A4	293.402	51.49	11721	8	1380613.363	1081202.695	1319901.539	1216087.324	1013109.836	931273.3532	876619.5715	820435.7958	924493.7569	931754.4404	1393770.648	1274459.144	1100906.11	1005796.262	895379.4581
P07356	Anxa2	Annexin A2	1133.734	71.98	38676	29	3914881.141	4078368.447	3654210.901	3612007.628	3740116.287	3770708.326	3775639.421	4002185.248	3911296.07	3765452.322	3718515.272	3678448.371	3821263.992	3826925.272	3773450.736
P07901	Hsp90aa1	Heat shock protein HSP 90-alpha	1880.892	60.3	84788	51	2825160.48	3064368.048	3127936.025	3062740.694	3106838.547	3286276.407	3523880.311	3737525.899	3324731.35	3265901.409	3034398.528	3306903.113	3163521.674	3252270.625	3176229.574
P08003	Pdia4	Protein disulfide-isomerase A4	868.491	44.04	71982	24	618571.5279	644631.9381	580421.7311	618637.9056	658990.6525	777486.0692	713590.7686	765986.1581	745485.1467	689218.7189	700592.2818	691550.7235	658994.6005	651532.125	597380.3906
P08030	Aprt	Adenine phosphoribosyltransferase	527.951	78.33	19724	12	603437.8349	525012.0664	497799.7584	561265.719	513177.0986	511552.0328	565259.2677	573322.1402	564054.4941	537208.2663	512413.5015	524949.8185	515687.4171	550281.8486	518076.9544
P08101	Fcgr2	Low affinity immunoglobulin gamma Fc region receptor II	293.703	39.7	36695	9	252624.432	246868.625	217328.004	206100.4787	227141.8112	180653.053	168707.9155	163290.6598	177274.5113	199478.3106	205025.9499	171142.1013	217813.1712	193819.756	215179.3162
P08113	Hsp90b1	Endoplasmin	1785.373	54.49	92476	49	3090893.75	3169137.392	3224201.648	3137229.131	3192167.869	3738164.067	3689061.009	3795675.308	3779101.111	3579714.357	3233554.783	3259645.608	3241199.327	3342764.92	3265203.838
P08207	S100a10	Protein S100-A10	70.036	27.84	11186	2	238097.349	131875.5301	143824.7774	169672.3965	147371.7428	139814.0715	119966.389	113577.826	132635.725	128306.5938	205051.9881	127204.6525	146150.0819	141178.772	98518.96311
P08228	Sod1	Superoxide dismutase [Cu-Zn]	173.22	34.42	15943	4	176302.4836	172529.5796	162932.8738	152992.9378	177644.8725	138242.8717	128724.2479	119916.3659	129190.8844	120979.1723	162005.1975	155636.1176	141815.6549	141614.9362	142499.4601
P08249	Mdh2	Malate dehydrogenase, mitochondrial	875.389	60.36	35611	18	1161434.57	1117625.104	1065763.45	1117251.734	1035809.803	1285655.956	1077697.382	1116149.821	1192336.624	1076618.08	1102768.297	1034146.329	1023581.439	1078332.212	1063477.881
P08508	Fcgr3	Low affinity immunoglobulin gamma Fc region receptor III	55.362	8.05	30036	2	128348.0325	115726.067	108332.1487	102323.1864	115816.7767	80313.97714	80590.19225	78149.48749	76163.89554	96292.89659	99506.28898	87324.90167	113497.8653	100124.7261	102185.6596
P08644	Kras	GTPase KRas	95.413	20.11	21656	3	55161.35018	52847.91542	47594.36952	50161.56687	56248.99649	53863.00683	51873.69843	50392.20313	53937.4841	55042.22914	51741.88983	52549.97421	56323.0066	52619.99086	57690.84042
P08752	Gnai2	Guanine nucleotide-binding protein G(i) subunit alpha-2	1256.755	69.86	40489	26	705787.859	689204.7557	624922.0204	657923.9788	662126.1904	542235.6623	580393.8046	546675.0495	575677.2623	548604.2191	585022.0539	589495.6291	603313.0806	589539.4642	617825.8855
P08753	Gnai3	Guanine nucleotide-binding protein G(k) subunit alpha	420.479	29.94	40522	10	208683.0176	205340.1486	180518.3089	201183.1714	197104.1262	164842.1261	184833.5038	184604.7358	187514.6137	158874.6912	161012.9772	169159.2355	176401.5299	174750.215	190743.6519

P08775	Polr2a	DNA-directed RNA polymerase II subunit RPB1	350.364	7.41	217176	10	67225.97018	68831.88263	67329.82098	73785.90797	65737.21064	58469.41884	56376.2361	58969.61994	53815.21835	59508.84079	54754.77959	64481.17866	52162.53424	60263.34213	65129.0342
P08905	Lyz2	Lysozyme C-2	74.868	16.22	16689	2	39325.11995	13085.21103	27255.24751	31909.88297	32612.46289	38409.49006	54998.59094	43346.36846	44790.33605	34711.9209	43283.55256	46010.10732	34826.85746	38023.57749	34333.30182
P09055	Itgb1	Integrin beta-1	467.858	21.55	88231	13	162465.2775	166235.139	147856.8778	160350.7034	155741.768	158808.8364	155111.6652	146801.0939	153334.3372	154473.3551	130795.3437	130380.2828	146139.5344	148091.7534	155215.7008
P09103	P4hb	Protein disulfide-isomerase	1539.052	66.6	57058	38	3149509.482	3124158.322	3130231.795	3166294.926	3116742.237	4472751.723	4095885.341	4037445.936	4271935.096	4018740.471	3274469.996	3337629.635	3314583.428	3446869.385	3133910.671
P09330	Prps2	Ribose-phosphate pyrophosphokinase 2	365.808	30.19	34813	9	103237.7608	101587.3443	108403.4921	120495.0587	109634.3605	104403.2624	104673.9893	107501.2314	115980.2866	122539.6456	97034.46068	114130.19	105680.6476	109431.4853	104665.9226
P09405	Ncl	Nucleolin	1046.262	36.35	76723	29	2539139.093	2502691.099	2540729.357	2594511.685	2565329.035	2315674.772	2727556.458	2784384.179	2511331.298	2429433.099	2508072.225	2655998.829	2571673.211	2628877.55	2683720.564
P09411	Pgk1	Phosphoglycerate kinase 1	2028.486	82.97	44550	48	8829100.648	8700622.921	8504147.384	8855084.731	8216684.888	8527302.509	8263255.096	7769414.168	8309214.286	7817266.91	8384398.878	8054228.452	8011241.567	8315448.129	7954646.35
P09527	Rab7a	Ras-related protein Rab-7a	710.488	74.88	23504	17	762887.4449	751813.2693	739696.3013	703704.2206	756792.2294	678752.9796	686777.4401	684488.2249	720469.5392	684392.2824	710076.759	750621.8073	748692.1579	770192.7709	740368.7277
P09528	Fth1	Ferritin heavy chain	222.436	41.21	21067	6	121468.2824	139999.4249	142364.3108	136725.922	146750.2989	307375.1546	283079.816	266604.0082	279662.0026	218345.1032	169146.7018	193189.5279	167126.7122	172174.1946	182225.5453
P09581	Csf1r	Macrophage colony-stimulating factor 1 receptor	258.036	13	109179	11	128403.6754	143937.2201	126039.4629	128494.5906	141602.1567	92418.38924	96309.23175	95294.10769	91506.99355	98086.27993	124556.8812	120639.4346	134892.6852	111759.7685	126589.1444
P09671	Sod2	Superoxide dismutase [Mn], mitochondrial	338.067	51.35	24603	8	164062.3876	164225.2152	143801.2023	137839.7251	138823.3072	194138.746	208178.5658	220371.9431	206391.462	168515.5761	150325.2382	147256.7959	154223.6273	144534.1272	147195.9613
P09895	Rpl5	60S ribosomal protein L5	265.395	31.65	34459	10	269234.4396	265367.6248	285121.9042	233051.3442	263201.8853	225296.2712	368384.2111	320393.2106	318513.499	223172.0193	278873.989	295418.3206	167234.1688	272775.2176	262567.8205
P0C0A1	Vps25	Vacuolar protein-sorting-associated protein 25	70.109	18.75	20762	3	9988.27857	15778.92554	33800.50051	36361.50458	15175.51154	18430.01726	6413.609872	17345.17742	9055.648111	32308.7394	34138.64638	33329.81691	34515.10644	41182.26915	13803.79047
P0C0R5	Pik3r4	Phosphoinositide 3-kinase regulatory subunit 4	85.965	3.46	152447	3	10832.12474	12414.55615	12664.84209	11414.71326	10533.03423	10851.62481	10377.58663	11352.97896	11897.93035	10533.7853	11884.03357	11167.8512	10680.01402	11900.81049	12190.13356
P0C0S6	H2afz	Histone H2A.Z	301.957	53.91	13553	6	13696408.72	12798786.26	13878419.94	13354976.97	14412748.41	16271450.93	14491249.3	14768626.39	14491136.89	15818692.53	13653452.23	13202000.81	14276729.65	13708250.03	14755555.37
P0C5H9	Manf	Mesencephalic astrocyte-derived neurotrophic factor	71.399	24.58	20388	3	23731.92446	19659.14432	20132.06257	15725.95508	22381.18314	15787.147	25690.88613	24849.20468	23611.42586	21291.82593	16375.71165	15153.85047	21632.42232	22137.72601	21363.9147
P0C7N9	Psmg4	Proteasome assembly chaperone 4	41.763	22.76	13974	2	5934.791938	6829.138354	5706.056276	6978.448667	4855.974114	3530.214664	5834.514349	3734.005207	6270.359825	5083.782936	5431.354203	5947.876232	5240.557322	7052.160392	6192.447549
P0CC09	Hist2h2aa3	Histone H2A type 2-A	539.504	63.85	14095	11	16594916.52	15622893.81	16174435.98	15922750.99	16558365.43	18273188.15	16796799.9	17243684.01	16998064.22	18335364.74	16057336.56	15179166.25	16884687.36	13701470.51	17397126.76
P0CG49	Ubb	Polyubiquitin-B	202.537	56.07	34369	6	1610577.211	1615007.605	1710586.751	1581392.485	1435783.541	1488857.329	1697072.343	1532412.889	1509388.456	1533816.552	1715221.557	1463096.299	1580507.47	1556813.539	1500721.28
P0DOV2	Ifi204	Interferon-activable protein 204	98.075	10.99	69438	5	35404.79815	36719.13552	30485.70229	35468.67013	44162.22359	39480.25542	40990.69802	35976.71776	42355.03213	38142.07216	27184.15701	37538.29397	40129.73986	46845.15084	37565.44359
P0DP26	Calm1	Calmodulin-1	247.581	60.4	16838	6	121159.8771	114690.621	66994.51555	123069.1124	117272.0721	88270.19173	111763.4582	93131.63353	120327.6355	86240.57111	150799.9127	135815.9461	143977.8841	80046.90294	74832.79728
P10107	Anxa1	Annexin A1	1203.964	71.1	38734	27	1239134.742	1235226.355	1113844.756	1141203.282	1065915.449	1171545.865	1102419.027	1154005.148	1158600.388	1082946.021	1197693.275	1170649.026	1213033.041	1204045.547	1214181.413
P10126	Eef1a1	Elongation factor 1-alpha 1	1368.036	67.53	50114	40	14777322.33	15271049.09	15477283.86	15327406.97	14750610.17	17100977.89	16285461.64	17192873.71	16407405.06	15777184.7	15579185.02	16099263.52	15350929.54	14959460.84	15770801.93
P10404		MLV-related proviral Env polyprotein	68.924	3.28	69613	2	20497.5145	21628.00601	22870.68818	20090.12333	5563.862273	13113.2579	23645.6485	24467.08505	9583.069039	23641.22052	17271.0711	18159.74167	18897.36306	20973.33145	19989.96546
P10518	Alad	Delta-aminolevulinic acid dehydratase	122.125	25.15	36024	4	22405.08855	21118.57522	19959.60972	20980.01157	21262.93641	19477.48259	19968.43499	19119.27789	21188.48476	15671.29314	22737.06313	23361.33514	22603.82861	19927.43665	6686.24381
P10605	Ctsb	Cathepsin B	1041.968	69.32	37280	22	891880.3315	884936.409	708097.6605	789889.9238	799651.9644	602047.0464	608949.3052	599163.9332	623961.703	561251.8964	707292.8262	751564.6213	729519.4473	815184.5973	758539.5724
P10639	Txn	Thioredoxin	141.27	34.29	11675	4	390853.8644	294971.1851	352268.4643	334582.8901	319369.9508	324094.47	356393.5958	303167.2927	322863.9533	319149.0276	416396.8809	369370.7883	323578.326	339604.8907	278582.7039
P10649	Gstm1	Glutathione S-transferase Mu 1	172.837	32.57	25970	6	80912.77004	115674.151	104459.6818	82813.48842	98427.3602	77315.73237	73462.55422	73259.43375	78725.99648	87493.16819	107737.1016	110222.9767	75847.83066	81240.56723	86474.65759
P10711	Tcea1	Transcription elongation factor A protein 1	175.251	20.93	33880	4	21691.99485	23008.87854	21195.57566	19742.04097	21796.2021	6086.037908	18255.45466	5711.095988	17508.84725	15613.23378	7771.933314	19470.24811	6493.548898	6804.142495	7592.666975
P10810	Cd14	Monocyte differentiation antigen CD14	612.498	50.55	39204	14	312606.6409	297975.4001	273550.8263	288584.3026	290226.1853	313357.5551	325549.9477	303191.0662	335513.5435	354086.8123	291379.4467	273624.2408	309551.1665	296560.5028	349592.9581
P10852	Slc3a2	4F2 cell-surface antigen heavy chain	727.33	46.58	58337	19	668401.5999	661311.6021	547358.4108	620283.7152	616898.1215	604427.1534	651725.7531	625579.3439	644469.2889	641201.8427	680422.3508	645496.7343	692883.99	660976.5118	666502.6454
P10853	Hist1h2bf	Histone H2B type 1-F/J/L	656.022	61.11	13936	15	14768653.22	13656565.05	13749574.4	8090721.226	15931577.91	15402620.44	11711306.28	13749871.51	12937014.59	14303050.48	15935824.02	14420528.14	7532344.915	7166764.013	13936174.71
P10854	Hist1h2bm	Histone H2B type 1-M	661.54	61.11	13936	15	14780585.49	13643805.02	13754024.74	8094255.842	15911415.03	15406088.59	11713037.04	13759850.14	12946048.71	14309976.13	15944194.36	14408511.83	7546320.387	7169908.057	13942298.8
P11030	Dbi	Acyl-CoA-binding protein	123.494	39.08	10027	2	22916.11056	16520.02526	14984.54633	16354.18714	14107.62249	11350.81429	11633.04981	10513.01043	10879.71088	9861.848467	17696.02673	14570.01691	14306.15699	12900.59618	11507.27073
P11031	Sub1	Activated RNA polymerase II transcriptional coactivator p15	35.967	15.75	14427	2	33524.67744	37580.02201	35331.34759	36123.66805	38590.04276	36893.98859	34404.97894	32751.78087	36165.59511	37052.2178	41582.83288	40646.89677	42826.54127	42885.04858	34890.54719
P11103	Parp1	Poly [ADP-ribose] polymerase 1	499.094	19.94	113100	14	176669.3946	165246.5951	186738.0231	180237.0127	147865.975	129466.5179	129711.0268	119223.2005	144080.4083	101756.8306	165229.3822	164404.4804	148321.9166	163870.0722	139027.1872
P11157	Rrm2	Ribonucleoside-diphosphate reductase subunit M2	154.207	6.92	45096	2	7521.543559	8606.449731	9515.558651	9969.680926	8508.566791	9632.020984	9250.797627	10832.77073	10634.45819	8670.997117	10368.80199	9110.259306	8330.842156	8054.522366	8913.590259
P11167	Slc2a1	Solute carrier family 2, facilitated glucose transporter member 1	91.916	3.86	53963	3	54798.40015	53621.67155	61101.94245	60189.07275	52347.89543	54404.37445	60125.01056	64318.07629	60382.09808	58966.34954	47567.00436	55968.55376	52407.31379	58713.62774	57706.32924
P11240	Cox5a	Cytochrome c oxidase subunit 5A, mitochondrial	114.351	27.4	16130	3	36882.30556	17955.13544	29665.45051	40046.09176	37278.33774	43100.15294	27917.55372	25861.66436	36989.19876	12484.00552	28318.64603	17515.05646	33784.15033	54044.28582	29118.2825
P11352	Gpx1	Glutathione peroxidase 1	33.565	15.42	22329	2	0.008226335	0.007562416	0.009509917	8793.217484	6221.201605	2470.390727	2180.630377	2403.620798	2902.51466	3015.59894	4536.942222	8106.931546	3719.558186	8336.931221	8071.704771
P11438	Lamp1	Lysosome-associated membrane glycoprotein 1	381.546	17.49	43865	7	508967.4884	531056.796	437288.3437	481829.6179	465582.2498	455789.8664	425124.4864	437623.413	489689.7061	460843.9012	450515.6365	423909.8143	451079.5479	516339.3011	490407.0167
P11440	Cdk1	Cyclin-dependent kinase 1	192.284	22.56	34107	6	36606.62873	39226.16707	45628.95963	44112.19227	45652.55382	49540.01185	44668.54902	40409.48326	53213.62881	51217.43737	33936.92261	43502.45611	41262.41191	51754.84454	56489.63299
P11497	Acaca	Acetyl-CoA carboxylase 1	257.096	4.65	265194	7	20508.57687	21132.78311	19109.14018	21867.2035	19666.2969	22726.00274	24789.56175	24324.47561	21030.42651	22859.76345	21276.90529	18513.12939	21168.5009	22523.86382	24405.91693
P11499	Hsp90ab1	Heat shock protein HSP 90-beta	1898.325	58.01	83281	49	4637699.875	4994238.214	5042811.009	4839741.952	4992168.291	5257848.316	5564751.943	5852813.05	5359577.57	5173068.295	4660859.3	5020958.22	4991985.129	4788633.497	4933641.024
P11506	Atp2b2	Plasma membrane calcium-transporting ATPase 2	135.354	5.63	136811	6	31283.02574	30289.97049	18591.20749	33344.22524	27065.33381	45924.16366	47122.25874	45213.2298	35164.4563	41967.14699	35474.21948	26747.15721	31784.83279	34999.28878	26599.43729
P11598	Pdia3	Protein disulfide-isomerase A3	945.818	52.28	56623	26	1290578.953	1282544.329	1231548.167	1250563.924	1251417.062	1486147.227	1360048.206	1415890.113	1472479.896	1441914.615	1336224.191	1315987.017	1423812.764	1379298.678	1347591.172
P11688	Itga5	Integrin alpha-5	144.39	8.64	115043	8	60462.71738	49239.35374	46582.30983	41871.04967	66644.21508	75785.89103	70168.48144	79143.11206	70685.28188	68951.51463	53922.26872	53025.07204	42482.08578	53156.94074	57099.29689
P11730	Camk2g	Calcium/calmodulin-dependent protein kinase type II subunit gamma	105.492	8.92	59038	4	85466.26054	97617.16683	86045.89487	91304.60058	86329.79623	75880.94497	84704.13579	76670.40967	72595.65326	82434.03466	87107.8246	86015.82513	95542.4492	85909.72002	89020.47097
P11835	Itgb2	Integrin beta-2	1205.242	41.76	85026	25	623078.8357	600760.1866	561649.8007	568800.8078	601608.3324	525539.378	518278.609	484298.689	518058.4052	507818.606	481043.3067	479114.3604	527735.6966	534839.873	552677.2649
P11881	Itpr1	Inositol 1,4,5-trisphosphate receptor type 1	78	1.09	313167	3	18494.38745	19135.06135	18356.69293	21468.62963	21002.73751	20656.90386	18681.1575	19549.42176	19048.87944	19376.09667	19915.38248	17039.39709	16826.64535	20370.99572	18860.30707
P11928	Oas1a	2'-5'-oligoadenylate synthase 1A	199.408	26.43	42429	6	17173.47451	17867.60993	21361.45239	22857.80192	17510.19926	28959.63096	26208.87684	23165.80298	26616.47507	19442.47282	20499.28711	21020.35831	19956.01892	22048.27849	22540.62492
P11983	Tcp1	T-complex protein 1 subunit alpha	1156.603	60.25	60449	30	813799.1813	820543.0442	799640.6586	761656.131	864607.7951	816327.8057	859332.2017	919680.9963	952766.4656	757196.9075	811624.4572	829645.6683	737891.2455	701942.9636	818515.4441
P12001	Rpl18	60S ribosomal protein L18	125.06	22.34	21659	5	157121.7133	153318.8052	150663.5017	152793.2095	166822.0362	227215.612	261543.3857	248462.0905	267649.3277	209531.402	169206.4384	166223.4298	185307.8243	156657.9205	176501.643

P12265	Gusb	Beta-glucuronidase	922.641	47.69	74195	24	957313.4583	897881.2682	733869.3086	774917.1067	774041.8867	539496.8875	575199.5918	522805.8417	576562.4429	648992.0208	789073.6224	724976.3516	800412.1109	816334.2613	769220.3331
P12368	Prkar2a	cAMP-dependent protein kinase type II-alpha regulatory subunit	196.091	21.2	45480	6	24147.84592	21105.48657	17230.68304	23488.313	25542.0228	15083.15987	21608.28302	15583.67363	25235.01468	19372.97974	29451.62232	23066.2076	12486.53504	27202.65025	24264.26154
P12382	Pfkl	ATP-dependent 6-phosphofructokinase, liver type	814.492	35.13	85360	20	422151.3192	494340.9956	428366.7858	418021.5543	462092.5033	398424.1637	399882.3037	422020.3384	411998.4101	431679.938	472470.1349	421910.2471	469296.1387	471921.8148	431763.163
P12815	Pdcd6	Programmed cell death protein 6	164.125	42.41	21867	4	37132.13937	35198.54925	33444.65367	33814.28098	31260.70978	26589.78541	25926.4765	26757.79107	30959.22015	29178.20909	31910.10362	30695.14357	34564.0393	32178.81492	33097.44398
P12970	Rpl7a	60S ribosomal protein L7a	552.629	53.38	29977	16	940026.6372	996769.8359	948022.0748	932551.9436	954359.8801	989642.9708	1114657.451	1083516.321	1067781.942	931120.293	931965.1828	981529.8123	897174.5627	902745.5361	955964.5223
P13020	Gsn	Gelsolin	1819.541	61.03	85942	44	4273537.252	4589352.044	4147595.766	4275285.908	4468035.598	3163679.377	3279380.327	3121558.096	2931246.779	3468644.671	4065772.276	3825802.124	4114477.634	3889786.781	3822283.606
P13084	Npm1	Nucleophosmin	512.731	39.04	32560	13	924459.8442	912642.8616	908040.2516	977502.3105	964659.8016	934118.3266	936707.1477	971746.1239	689917.0821	894033.1629	982874.3083	937885.3898	921848.0432	920002.9776	851565.1732
P13379	Cd5	T-cell surface glycoprotein CD5	215.179	18.62	53849	6	132250.1288	148942.2991	117595.6547	115518.8087	141436.8064	127858.925	117968.3071	112970.5338	108294.1651	109090.4926	84557.78508	104504.4492	128064.5347	119790.9243	140431.5002
P13439	Umps	Uridine 5'-monophosphate synthase	164.849	12.68	52292	5	33067.6531	40673.41681	43304.95928	34639.34557	44716.66434	47217.70055	48545.22281	49129.65879	42718.86593	43479.07478	39377.04339	46981.03481	47236.26727	45079.93593	51577.05496
P13471	Rps14	40S ribosomal protein S14	311.078	66.89	16259	9	412032.9925	417151.9327	401519.9465	388677.2142	421716.0307	370168.7389	434378.1366	408967.6881	376770.0206	358786.8669	399914.6889	388175.4801	389631.1285	357666.0076	376847.6089
P13668	Stmn1	Stathmin	133.677	20.81	17288	3	29627.59298	26582.06514	31565.5653	28988.30868	25721.62958	33084.72835	27025.61726	25731.88939	25237.98719	28896.02093	31610.68624	30718.60614	27965.12677	28567.50029	31399.43386
P13832	Rlc-a	Myosin regulatory light chain RLC-A	323.911	62.21	19895	9	288074.8863	243261.1864	264530.5555	267275.7019	287919.4156	281607.7221	238012.1087	227580.4357	250202.5362	253733.1102	276914.3315	277683.7141	302376.0291	274221.6783	284715.6354
P13864	Dnmt1	DNA (cytosine-5)-methyltransferase 1	633.574	15	183189	19	157446.67	167368.7451	178273.8162	164405.7252	177585.3583	173946.3669	173034.5739	188698.8929	173139.5038	183148.0411	162729.4719	171190.7564	156101.6246	171052.8619	164139.6353
P14069	S100a6	Protein S100-A6	189.18	78.65	10051	6	1475254.916	1346599.079	1181803.691	1167499.991	1273583.98	780560.0736	784260.5919	732953.175	989172.5503	777370.4371	1112436.618	1044828.126	969225.3079	894718.6924	919118.3222
P14115	Rpl27a	60S ribosomal protein L27a	165.384	22.3	16605	4	251604.4487	281065.8386	259889.3924	253659.8229	273213.0804	302284.4801	319613.641	335720.03	309138.7483	287038.2129	252421.2839	267204.8342	255775.4499	239803.4012	297720.4241
P14131	Rps16	40S ribosomal protein S16	263.487	52.05	16445	10	758597.2465	764597.5028	753821.9523	834505.0686	794222.2869	844077.3618	896367.4488	908372.8894	926429.3221	891551.5056	767183.946	830588.5549	804625.1293	837723.8206	818720.0002
P14148	Rpl7	60S ribosomal protein L7	220.793	31.48	31420	8	104689.4787	76632.46149	99615.70071	112751.3365	112247.2151	188307.7049	248458.8931	209632.0105	240837.2347	222102.4083	100693.0556	148036.4906	125724.8237	93349.23042	162682.5774
P14152	Mdh1	Malate dehydrogenase, cytoplasmic	618.435	47.6	36511	13	803781.6766	833422.6852	791333.4794	816405.5654	817977.043	670679.0872	710145.4455	722568.2888	706358.7533	688691.5059	746250.828	785122.1216	723093.9546	778977.1051	743505.6133
P14206	Rpsa	40S ribosomal protein SA	721.327	57.63	32838	16	1596701.16	1868727.377	1649457.002	1766340.043	1782659.848	1214712.203	1474296.387	1363648.629	1367801.419	1251295.601	1698761.669	1677039.737	1535935.849	1477521.451	1464140.555
P14211	Calr	Calreticulin	725.785	58.41	47995	23	2262972.197	2142861.438	2083942.611	2121351.557	2198651.962	2592538.375	2412202.707	2448570.679	2479346.283	2324972.57	2177894.49	2152030.509	2197709.867	2229371.41	2183858.144
P14576	Srp54	Signal recognition particle 54 kDa protein	68.357	8.93	55721	3	18395.50073	20393.42842	21346.55273	20467.12218	18038.17687	6178.030274	8989.676794	19848.94186	19150.74239	20097.77773	17415.88656	20873.77951	17979.60654	17100.06676	19223.08483
P14685	Psmd3	26S proteasome non-ATPase regulatory subunit 3	410.713	25.66	60718	11	182469.9152	199012.7359	201565.0115	192621.3959	195571.6194	209823.8309	235282.034	222864.8502	220811.6067	204560.2549	198289.9852	193853.1822	196063.1128	194999.2263	198333.5067
P14733	Lmnb1	Lamin-B1	1089.586	57.99	66786	32	798742.0919	800963.708	827822.348	811240.4732	835253.2833	713690.0239	733323.3832	676299.3276	715340.8181	714114.3081	778236.4453	775402.4129	782313.9657	805848.618	750061.1876
P14824	Anxa6	Annexin A6	901.215	49.03	75885	27	401262.8769	419406.1981	379407.8668	411517.3709	402442.4282	414277.634	385650.6626	388219.5784	413867.9034	417435.9163	400193.7066	416034.4693	429665.0067	433299.4383	416767.3717
P14869	Rplp0	60S acidic ribosomal protein P0	485.889	50.47	34216	11	886598.4977	936993.5377	937544.3521	943630.9303	927806.2381	858277.6278	1018412.16	980428.7646	947189.2575	915159.1925	822486.6104	945797.5069	830097.3517	865893.7267	915425.3724
P14901	Hmox1	Heme oxygenase 1	113.408	17.99	32929	3	13269.84291	16814.54543	16955.96649	18077.50744	15987.73137	16864.87692	20622.00577	17603.64423	15910.79517	14089.40439	17724.92545	15745.84452	15571.38117	14102.42365	15231.84828
P15307	Rel	Proto-oncogene c-Rel	32.916	4.26	64960	2	3054.836793	4084.514112	4708.266511	4157.772293	5299.297463	6235.991367	4434.638042	4462.438914	5345.161715	5210.689351	3366.154271	4313.608314	4068.443027	4825.117662	5157.161884
P15379	Cd44	CD44 antigen	157.423	5.4	85617	4	103019.8205	116311.9424	103651.0915	93331.87304	109898.1572	116554.2963	94727.99112	93847.96138	110721.935	100615.2506	107826.5698	111941.5999	124356.3815	123498.6321	129839.9116
P15429	Eno3	Beta-enolase	440.355	19.35	47014	6	454116.1271	466351.1943	388086.2371	443157.1648	472476.7035	402459.2198	420399.4663	422363.137	391885.7093	424566.5853	377157.3186	431400.1938	467166.9844	449431.5754	410162.316
P15532	Nme1	Nucleoside diphosphate kinase A	349.309	69.08	17208	11	379268.0583	393672.7192	396925.9716	373193.0896	398998.2763	391599.589	388728.3222	403240.4535	387813.0279	332844.1245	376129.6619	402864.8522	375495.963	387569.9416	362405.3124
P15651	Acads	Short-chain specific acyl-CoA dehydrogenase, mitochondrial	152.91	14.81	44765	5	44345.86218	41180.00792	39556.73706	15632.39262	38797.19759	34888.94662	36857.85206	39699.69588	45666.57795	23515.85591	46406.36447	40489.64184	44335.18119	43205.53339	37423.77526
P15702	Spn	Leukosialin	103.556	9.62	40038	2	14853.66851	14233.34454	14701.86166	51468.43538	46698.26351	70019.23937	60215.1606	58564.54789	60177.31488	57170.44202	18221.39163	46763.53166	45257.53444	0.00945634	11923.16376
P15864	Hist1h1c	Histone H1.2	41.584	16.04	21267	3	56425.07981	43960.18636	57196.35249	53962.43447	45579.806	125604.7035	63624.26559	61026.41891	80390.37428	89961.93163	57442.56981	48214.20783	49919.85002	52731.97284	85028.51956
P16045	Lgals1	Galectin-1	739.544	94.81	14866	15	1018485.738	863511.2988	892948.0887	751039.368	745615.4655	667351.5087	650364.803	601819.4495	654489.22	610574.3107	846281.4862	811152.6153	793474.3748	781777.3164	748009.7422
P16110	Lgals3	Galectin-3	576.284	30.68	27515	12	1169621.024	1027561.694	996914.8429	984889.0842	837723.7171	662869.063	765936.6418	648957.1413	676283.5416	635885.0102	1076787.755	935526.1084	868780.9981	881177.668	837618.4777
P16330	Cnp	2',3'-cyclic-nucleotide 3'-phosphodiesterase	106.342	8.81	47123	3	4882.079117	4257.701978	3242.539498	5196.943993	3496.470977	2083.650019	2511.014357	3597.164646	3788.442367	3462.323443	4127.65346	4901.234283	4176.513081	3848.783128	4059.286353
P16332	Mut	Methylmalonyl-CoA mutase, mitochondrial	107.713	7.35	82844	4	26024.94551	29136.44498	30318.56073	30892.99559	29158.51587	33858.57646	35935.58157	30521.8433	36195.62535	34351.31014	28728.88274	30228.19266	30569.89302	27219.28395	31284.40474
P16446	Pitpna	Phosphatidylinositol transfer protein alpha isoform	125.049	40.96	31907	8	155140.8776	158746.3963	145309.3135	145705.2212	148475.4002	109878.8636	109076.6649	99940.90018	100265.1895	122419.1245	156407.1255	140785.1023	115958.1607	146264.5049	145406.5205
P16460	Ass1	Argininosuccinate synthase	229.08	26.21	46584	10	26640.81011	30540.1957	37466.49524	26158.04878	36157.73525	227123.0516	209305.4381	239170.8939	216288.1909	129783.3364	37608.05788	50082.59113	40506.4436	45401.99048	47462.7004
P16546	Sptan1	Spectrin alpha chain, non-erythrocytic 1	408.587	7.2	284597	13	82159.40535	86486.16815	68529.54461	68020.1039	74881.33363	56126.29745	55969.24274	50842.83633	60230.69324	63311.58765	74635.23658	70230.37365	73755.30301	63911.54594	70688.74459
P16675	Ctsa	Lysosomal protective protein	740.128	35.65	53844	18	735294.2102	717667.3895	655400.2408	625630.2485	634074.5764	527035.6978	560787.6383	505336.3015	561928.1564	517014.7564	716680.5532	690373.2204	680823.5833	687840.0057	608408.4325
P16858	Gapdh	Glyceraldehyde-3-phosphate dehydrogenase	2372.076	87.69	35810	49	19843809.86	19948228.72	19491468.41	20103606.22	19542746.26	21128304.12	19598461.22	21457366.27	21393272.66	19424377.19	18562183	19928616.62	18503631.77	19712435.6	19184424.28
P17047	Lamp2	Lysosome-associated membrane glycoprotein 2	207.745	17.11	45681	7	1568110.367	1626416.567	1402160.499	1461001.72	1505847.474	1551945.984	1418451.648	1430469.68	1591240.933	1636312.194	1635193.271	1529605.267	1644576.997	1765322.348	1707353.242
P17074	Rps19	40S ribosomal protein S19	335.843	55.17	16085	11	598045.9657	510024.6895	675117.7232	680493.5513	650881.7127	654238.1217	727657.0142	613001.9682	683171.2655	647796.614	632290.1417	610849.3316	573692.8318	672650.3252	585249.213
P17078	Rpl35	60S ribosomal protein L35	42.539	18.7	14553	2	36074.91954	43846.56863	42329.29881	39382.20933	41817.95987	73387.39117	93607.33005	82786.39289	88549.72961	78688.13836	41336.14973	51241.54073	48011.01496	40367.57239	63373.73616
P17136	Snrpb	Small nuclear ribonucleoprotein-associated protein B	31.968	3.9	23656	2	67863.97596	64736.88746	70964.70638	69700.96084	61535.09594	71940.43396	54034.84193	38220.8715	61061.06359	64105.98362	57523.64152	65346.06946	63014.75639	68047.95219	64531.57488
P17182	Eno1	Alpha-enolase	1915.87	79.26	47141	40	6769287.88	6997460.909	6352567.663	6473430.409	6680324.897	5769069.525	6073898.695	6088459.461	5744464.183	5915645.911	6419522.638	6249835.932	6369025.641	6273816.237	6091933.615
P17220	Psma2	Proteasome subunit alpha type-2	486.687	43.59	25927	9	220987.9206	228590.2209	229446.3397	232239.6465	228600.6857	150879.5567	219612.2155	239247.9632	220862.1153	214656.9832	225059.7677	229078.7034	180390.8636	213427.514	233544.5016
P17225	Ptbp1	Polypyrimidine tract-binding protein 1	1128.374	66.98	56478	21	808520.1115	832959.5628	782624.9817	871725.0022	736236.0962	564241.0089	723661.6901	725355.9477	709830.0687	626595.1922	765009.2881	773683.1466	730810.8095	707470.6859	677825.2132
P17426	Ap2a1	AP-2 complex subunit alpha-1	704.197	30.5	107664	21	193524.0533	238488.5458	203224.4541	211898.8906	227426.3882	205454.5194	195704.5201	228082.0665	203827.5848	182772.8115	192249.1509	210502.723	209065.7854	237261.4236	201038.8601
P17427	Ap2a2	AP-2 complex subunit alpha-2	577.944	31.24	104017	20	175317.1989	195921.9463	188250.4614	197889.2228	180933.709	167650.4157	174917.7038	190139.7693	163303.4093	174741.5646	175537.7164	187752.0623	180969.9829	192684.2387	187705.8742
P17439	Gba	Glucosylceramidase	172.323	19.61	57622	5	51552.00853	48127.64082	49093.86953	49835.54331	41842.48881	22984.65123	43429.08328	28980.33242	42498.65829	39077.71539	42096.36753	40783.9814	45310.83591	42809.77139	49906.588
P17710	Hk1	Hexokinase-1	1049.753	37.06	108303	32	818294.3799	874892.9764	808837.2052	838990.7654	920721.1406	823796.7843	801017.8664	808071.8256	850893.8473	799720.9115	792726.1825	822140.906	829908.2628	893496.2525	837421.5469
P17742	Ppia	Peptidyl-prolyl cis-trans isomerase A	1008.804	71.34	17971	20	2768392.448	2810325.814	2698214.351	2779964.607	2719647.199	2152914.396	2347837.718	2167823.817	2187000.82	2104056.951	2438440.688	2577387.612	2435659.61	2466866.396	2471415.403
P17751	Tpi1	Triosephosphate isomerase	1106.771	75.59	32192	22	8237226.219	6167738.31	7317276.21	7706068.411	6371919.219	5305717.058	6655840.195	6442636.281	4719132.545	5470570.514	6279904.233	5617785.321	6907896.383	7067899.42	5492659.268
P17918	Pcna	Proliferating cell nuclear antigen	670.762	70.11	28785	19	555300.8743	606119.4588	748860.5143	720926.9663	676951.9673	544759.3062	578347.4154	597083.9787	611229.2932	505125.6904	565817.3715	698865.7202	624173.1885	631684.5921	588867.2777

P18155	Mthfd2	Bifunctional methylenetetrahydrofolate dehydrogenase/cyclohydrolase, mitochondrial	92.016	21.71	37863	3	6528.54003	8519.781611	8914.609088	10252.59457	6465.215465	15415.33707	18768.85637	17360.16775	13248.56071	6674.025105	8614.913579	16081.49493	7569.150014	9320.430162	7732.265146
P18242	Ctsd	Cathepsin D	859.885	54.39	44954	24	3486009.506	3181916.544	2556173.311	2712380.763	2746133.921	2063456.244	2209473.362	1953448.42	2184164.801	2093347.947	3099020.171	2796425.376	2927266.118	3046832.63	2821723.634
P18572	Bsg	Basigin	59.597	9.77	42445	3	19205.6179	20426.20582	17169.73992	19827.60049	17761.75414	22546.83243	22389.35488	26367.74579	24688.91205	21363.58178	19131.22658	20975.19478	19063.47083	19338.00645	20378.58857
P18653	Rps6ka1	Ribosomal protein S6 kinase alpha-1	168.673	9.25	81595	6	36831.26527	37419.56646	31669.55149	35227.07116	35909.5611	34209.83984	34694.08759	37147.95581	36364.73839	35293.7067	32777.74315	25788.78294	39251.99746	29764.29463	38567.86311
P18654	Rps6ka3	Ribosomal protein S6 kinase alpha-3	227.92	13.51	83694	9	71039.62932	73037.93943	63383.93274	62485.12274	69019.79814	51198.28325	59854.46785	68652.43263	68600.72243	67102.17318	73607.62817	66266.13647	74477.9855	47729.02545	67351.45236
P18666	Myl12b	Myosin regulatory light chain 12B	271.774	50.58	19838	8	285538.2136	243322.3663	265505.5312	265932.0985	284653.3432	281715.1131	238044.684	229293.6097	249800.8119	252356.8419	275110.7746	277686.8116	301616.5335	272124.7539	280978.3016
P18760	Cfl1	Cofilin-1	690.857	73.49	18560	14	4079557.369	3736853.868	3271299.932	3483498.016	3558292.254	2690209.071	3090002.942	2943822.471	2968897.066	2784959.544	3630720.195	3173095.683	3566846.162	3436213.83	3297672.223
P19096	Fasn	Fatty acid synthase	3301.881	53.55	272428	87	1647409.607	1758399.96	1664420.134	1658048.091	1548736.57	1382850.444	1528066.879	1534771.681	1480953.347	1384053.748	1644986.602	1674982.385	1583010.917	1598236.392	1587597.435
P19139	Csnk2a1	Casein kinase II subunit alpha	141.339	24.55	45073	6	76034.08429	66710.62502	69416.96732	71021.0816	62903.91804	69228.56508	79121.81668	74034.87584	76625.14863	86554.97415	72686.86014	70872.29076	80583.32598	74196.59659	68658.24279
P19253	Rpl13a	60S ribosomal protein L13a	144.874	27.59	23464	6	262092.2036	276162.6103	278112.7717	281233.3329	293542.4149	384131.8608	377854.33	401350.9088	401262.8525	361044.7984	296866.0436	334145.5026	292754.8427	288498.387	328361.522
P19783	Cox4i1	Cytochrome c oxidase subunit 4 isoform 1, mitochondrial	181.422	30.77	19530	5	152733.9341	167376.3075	162399.3484	160146.8847	165220.0632	181846.6106	173956.9802	169912.3426	181957.0049	186723.6455	179583.1671	169602.6559	175008.0486	167308.5296	168891.1918
P19973	Lsp1	Lymphocyte-specific protein 1	598.308	46.06	36714	12	507845.6137	470264.5932	442871.8065	395328.5387	413217.835	339035.3352	361245.1384	300981.1631	297177.0169	341216.6612	414119.4219	377151.9388	416378.112	397496.5261	368137.6066
P20060	Hexb	Beta-hexosaminidase subunit beta	453.303	38.06	61116	16	205799.539	251540.8821	214056.1169	225602.5555	225005.9463	194354.6039	140166.307	181033.3447	193838.1401	146432.0648	224035.0238	201569.5923	196119.9416	232477.5371	224087.4925
P20108	Prdx3	Thioredoxin-dependent peroxide reductase, mitochondrial Prx3	201.247	42.8	28127	8	177021.4653	208692.991	200427.3211	199514.3974	203364.7704	244162.6497	223185.7404	218994.819	224172.335	234251.9905	200029.6536	211024.4878	211257.6554	216127.9798	219472.217
P20152	Vim	Vimentin	2334.946	84.12	53688	58	13333859.25	12695979.03	11874148.08	12236534.33	11847859.53	10401975.38	11917033.83	10465721.26	10628389.37	11971825.11	13136296.79	12479391.93	12834910.03	12357881.1	10786640.55
P20491	Fcer1g	High affinity immunoglobulin epsilon receptor subunit gamma	107.446	25.58	9652	3	219974.5513	212848.0695	200452.7506	204267.3769	216755.2635	242411.0691	218590.4781	234393.5419	215342.69	249051.1178	211674.1603	203882.1568	218374.2764	213980.0101	228415.598
P20611	Acp2	Lysosomal acid phosphatase	47.803	4.49	48320	2	12980.64918	11988.39323	12286.80623	11322.33388	9918.447982	10626.59052	12616.9716	12804.99052	14420.14778	13263.32223	10614.86456	4570.278612	14007.52653	13286.19024	13669.33039
P21107	Tpm3	Tropomyosin alpha-3 chain	198.317	29.82	32994	11	687946.2539	505291.8575	630474.2145	624577.846	577723.9016	537671.9682	597976.945	542081.8306	606217.0077	616796.6495	629390.0185	560341.611	524573.3033	638906.7012	612655.8718
P21279	Gnaq	Guanine nucleotide-binding protein G(q) subunit alpha	213.382	17.27	42158	5	29040.07839	27759.04359	29192.95263	26249.15682	21936.0811	21545.10204	23994.18881	22773.12103	23956.34765	24942.46957	25223.66266	22952.99084	24680.56451	22836.15234	25446.94713
P21300	Akr1b7	Aldose reductase-related protein 1	111.066	15.82	35988	6	201564.2596	185546.7808	181711.0237	187702.9147	183192.5864	193358.584	209095.7116	205194.2216	198545.4168	185280.0303	194219.14	200118.8889	206074.9576	204465.0979	206797.159
P21575	Dnm1	Dynamin-1	42.89	3.94	97295	4	60213.63217	63484.3548	63073.70972	61717.92324	62232.6807	70938.91803	70460.97975	66737.67029	71888.72076	69304.75933	65234.83643	72267.33786	66930.10088	66640.80158	69946.82997
P21619	Lmnb2	Lamin-B2	262.278	18.96	67318	11	301689.5371	265232.8928	298083.9408	286754.8142	305382.9272	267101.1316	275601.506	196285.8026	265088.9547	283547.6579	300674.6721	279497.7906	279861.3731	311133.8569	272060.6144
P21670	Psma4	Proteasome subunit alpha type-4	257.786	46.36	29498	7	113086.7244	102374.9019	119383.1483	119810.0507	121847.8201	105269.0275	118735.3079	115862.7431	110726.5629	103899.5958	105875.7794	116045.4441	114270.4789	115552.7631	116602.6559
P21708	Mapk3	Mitogen-activated protein kinase 3	616.377	62.37	43081	17	381849.581	368153.5591	305185.9091	335128.2406	346758.3454	303075.5033	261840.811	267978.5078	284962.8636	316531.238	342251.7518	312026.3252	342858.7287	374685.452	316166.7107
P21855	Cd72	B-cell differentiation antigen CD72	115.763	10.73	40347	3	23549.84687	26321.19961	18402.58898	14560.54052	19382.07947	24533.50518	23069.60608	24222.12446	26505.7852	25999.68282	27100.89265	15416.5647	21345.87482	21441.81979	25734.57561
P21956	Mfge8	Lactadherin	107.297	12.31	51241	4	25934.37356	28469.9844	22546.53313	35534.58743	30383.51203	37076.48125	35904.42328	41784.40991	36349.73082	30080.02238	21713.57047	33597.22049	25684.46188	28836.66554	25922.49285
P21958	Tap1	Antigen peptide transporter 1	167.833	13.26	78864	6	30447.1766	20772.79889	20747.3566	21398.04194	21123.5203	31134.77768	22319.78821	20747.03348	24656.53159	27361.44715	19962.25892	26864.07608	29774.08965	18671.96218	18639.0539
P22437	Ptgs1	Prostaglandin G/H synthase 1	696.456	49.17	69042	19	433215.7731	455019.1261	399279.2958	434137.1643	443337.2694	371643.3944	322282.739	300295.1214	347545.7452	404945.7635	397657.7966	381032.4969	431493.1021	412416.474	400772.3388
P22509	Fbl	rRNA 2'-O-methyltransferase fibrillarin	373.27	49.85	34222	12	393057.1074	363254.2027	372857.778	351674.2322	373405.3254	373061.5774	372988.1174	370621.4506	365840.1863	351718.3232	363879.0827	355937.3905	372165.87	394331.3758	339358.3169
P22682	Cbl	E3 ubiquitin-protein ligase CBL	70.203	3.61	100564	2	12235.89025	12348.5788	12000.86203	10004.00722	12262.7365	9192.402006	12916.17976	9944.72762	11642.779	10288.72216	13995.76241	11573.47382	10259.12992	11086.54045	11008.96966
P22892	Ap1g1	AP-1 complex subunit gamma-1	392.919	19.1	91350	12	120208.2875	113158.0521	117250.0453	110250.6333	116049.5267	106342.7408	118572.5303	124272.9751	121794.4378	115405.5799	116360.2658	121639.5352	125597.8224	130209.333	114576.4382
P22907	Hmbs	Porphobilinogen deaminase	130.623	18.01	39344	5	36401.87935	38947.57146	39131.48921	42968.70697	34140.98759	34746.16942	41326.81731	43043.44447	39515.42412	32784.48319	36459.67671	35000.20618	34459.5802	31780.92525	39171.54128
P23116	Eif3a	Eukaryotic translation initiation factor 3 subunit A	755.463	20.54	161936	23	284581.4908	312976.9642	329336.7439	315103.6127	305473.7194	308736.0217	334950.8163	317303.7178	332875.9335	284462.3818	317881.6534	336489.1332	321105.0596	306966.4102	300413.6144
P23198	Cbx3	Chromobox protein homolog 3	366.566	42.62	20855	7	181265.1684	164451.0139	166250.5417	151729.2973	157472.4571	135580.8525	148903.7415	145687.7828	127410.4819	117736.6392	154881.6528	140744.2726	155029.1393	140583.3252	138479.4631
P23249	Mov10	Putative helicase MOV-10	210.926	8.76	113583	5	13893.24489	21290.75252	12588.34313	17977.70635	16878.23095	17956.39057	16281.04325	22844.97426	19862.27414	18593.95194	19225.98124	16636.78145	18124.81317	18176.97126	19793.65111
P23475	Xrcc6	X-ray repair cross-complementing protein 6	172.197	15.95	69484	6	30397.69313	26603.56697	26802.89878	21422.40956	19911.2205	22968.01407	27516.50924	23531.52629	27137.02436	33487.35616	19825.92238	23689.04581	33095.76672	21198.9999	17733.57162
P23492	Pnp	Purine nucleoside phosphorylase	251.547	31.49	32277	7	135185.5732	149361.7409	169505.5962	153615.5549	158496.8119	129690.14	130946.5287	145799.9151	128537.4148	120052.0207	137573.9142	151188.0651	139668.2511	151424.0351	150059.912
P23591	Tsta3	GDP-L-fucose synthase	149.431	18.69	35878	4	20211.5856	26494.98014	18521.99669	26500.44964	21421.6781	15456.90636	23425.87036	11258.03792	18959.52279	16810.00595	23949.27454	19691.45338	18940.56349	22408.03075	22854.35647
P23780	Glb1	Beta-galactosidase	558.879	27.36	73121	14	340958.574	323585.4302	275563.0107	300544.2839	265219.3831	231928.4844	246125.9947	233130.0221	242555.4198	275885.8331	280198.8161	239567.3106	304706.7724	308360.3881	306346.9421
P24049	Rpl17	60S ribosomal protein L17	248.799	46.74	21397	8	145057.7761	160413.6367	146180.032	165907.9153	176641.3807	173487.1855	168220.7929	173795.4686	176079.718	152340.3003	153976.9776	164762.6005	159722.484	157632.037	165300.3439
P24051	Rps27l	40S ribosomal protein S27-like	146.25	25	9477	3	138192.4715	154374.8814	141062.774	142741.6616	141169.6374	119900.2095	113488.256	128932.9457	133435.2948	115162.3266	120401.7175	123504.1236	116602.0464	119345.7293	113020.9667
P24270	Cat	Catalase	342.177	24.1	59795	9	85180.3378	81910.30922	71141.5433	78800.82793	75327.86359	100015.8125	92625.8795	89382.81636	94429.07672	96200.72756	87533.955	64355.52416	79938.57695	77693.06871	67561.42174
P24369	Ppib	Peptidyl-prolyl cis-trans isomerase B	393.827	43.06	23713	11	278388.8042	286216.4789	260462.3839	286666.7533	268058.1766	297252.3136	296941.9414	290648.7952	304503.2469	325531.3686	301017.9324	282616.0076	291781.3171	303183.0989	292254.061
P24452	Capg	Macrophage-capping protein	858.851	52.56	39240	17	2290226.77	2317402.234	2208040.795	2179173.385	2105746.749	1766897.028	1958529.648	1825297.318	1815491.967	1914932.675	2506570.055	2218562.663	2295520.903	2249646.256	2074218.413
P24527	Lta4h	Leukotriene A-4 hydrolase	368.838	27	69051	12	116358.9138	120967.1614	98313.84056	122125.0206	94238.66099	83666.28783	95880.68413	97319.1555	109907.7685	83073.92978	82603.24508	106585.6758	102307.4523	101358.8825	111326.2926
P24547	Impdh2	Inosine-5'-monophosphate dehydrogenase 2	704.166	41.25	55815	17	372996.9583	410560.7732	424316.6261	397110.5575	419769.1564	411951.7605	442904.3628	461424.2444	415481.4705	395678.8828	401928.7402	410895.6544	382336.7214	378439.354	389705.2174
P24668	M6pr	Cation-dependent mannose-6-phosphate receptor	311.197	29.86	31172	8	325008.6641	316912.9293	293146.5248	323040.5059	311207.8449	339504.8254	333469.6168	343197.1958	338779.5138	324569.9441	294378.1868	309264.3625	289187.3877	334240.4227	311167.1674
P24788	Cdk11b	Cyclin-dependent kinase 11B	45.928	3.06	91513	2	8745.887022	9496.467599	6940.230207	8016.141848	8920.339316	7796.118678	6721.181096	9343.394513	7082.98004	9001.094659	8308.645679	9428.575982	9503.907293	10093.82337	9026.460575
P25113	Pgam1	Phosphoglycerate mutase 1	1015.485	74.02	28832	21	2221667.833	2073040.86	1894856.237	1892748.959	1779660.863	1518283.441	1675442.558	1545206.114	1728206.026	1720471.905	2249426.013	2001612.393	1474507.288	1828515.207	1805856.768
P25206	Mcm3	DNA replication licensing factor MCM3	880.595	42.86	91546	26	436520.7198	427357.5034	434525.2182	471309.1881	464753.4486	462946.2394	443536.7528	429745.1813	464596.8132	429400.1348	441398.0761	461769.8276	450023.3498	435207.0467	412529.5054
P25286	Atp6v0a1	V-type proton ATPase 116 kDa subunit a isoform 1	258.067	14.8	96328	9	47345.27453	56170.78794	52138.50289	49537.08349	50462.05625	45239.32457	58063.74153	54773.32179	48833.30741	45276.96368	60455.55612	55389.5839	53468.36859	49449.16199	57752.33985
P25322	Ccnd1	G1/S-specific cyclin-D1	26.402	7.46	33429	2	5642.620271	5807.014083	6996.852256	6322.674633	6442.709597	5564.448921	5582.223786	5962.143177	5477.356626	5362.331754	5838.049482	8411.099807	5823.524628	6542.845534	7983.7798
P25444	Rps2	40S ribosomal protein S2	517.136	54.61	31231	15	1351813.687	1453881.929	1480263.245	1664394.495	1430314.849	1587985.262	1604837.54	1567760.45	1614485.502	1467601.477	1275040.108	1391470.253	1392916.616	1430700.617	1412787.865
P25799	Nfkb1	Nuclear factor NF-kappa-B p105 subunit	127.435	8.34	105615	7	41542.18314	35912.62279	42304.06425	38819.46124	40036.89458	48336.6399	47699.78761	41053.67495	42881.31196	47733.97539	36888.83257	37284.79993	41286.89673	50255.53717	41155.69316
P25911	Lyn	Tyrosine-protein kinase Lyn	434.665	24.22	58812	11	70542.90674	68396.68829	66439.33608	65767.83216	55248.35227	40047.84193	46860.95886	52923.87046	69599.90978	55016.79424	66179.00747	61704.13583	76072.86439	49939.07603	60458.35635
P25976	Ubtf	Nucleolar transcription factor 1	64.217	6.54	89509	4	33937.72585	35601.48232	39128.18451	31275.64613	35612.59772	30138.79778	26837.7908	28007.49573	26861.87053	28969.38852	29831.50793	22065.34846	31493.21762	24078.15744	22354.94554
P26039	Tln1	Talin-1	5661.689	64.62	269821	120	3312361.702	3402907.996	3337683.721	3345493.302	3298767.56	2895463.813	3269043.172	3233287.967	3105393.518	2991764.93	3241703.683	3280194.492	3197914.673	3293079.042	3300147.451

P26040	Ezr	Ezrin	686.864	35.32	69407	21	2288924.771	2262365.756	2321924.872	2467061.518	2262552.077	2434898.335	2509015.93	2144583.161	2317616.239	2522809.741	2282502.081	2281179.081	2349590.771	2458283.973	2512921.719
P26041	Msn	Moesin	2283.098	75.91	67767	64	6700957.438	6455066.547	6446020.481	6664342.558	6472790.207	6331356.37	6605123.333	6131361.424	6375972.529	6589812.326	6839948	6540733.344	6839466.429	6968232.323	6588426.662
P26043	Rdx	Radixin	725.608	37.22	68543	22	2644547.536	2612303.293	2681833.252	2815841.671	2595404.33	2708650.654	2808725.053	2433873.134	2621765.527	2843350.555	2628223.957	2608346.867	2690908.835	2792952.153	2861085.924
P26151	Fcgr1	High affinity immunoglobulin gamma Fc receptor I	103.29	10.4	44888	3	12664.11419	11701.37119	13104.68528	12090.59838	11317.16977	8813.541143	9204.61279	10233.01719	11421.06251	9475.657953	11202.28949	10143.6461	13137.28753	11907.10841	10209.42393
P26284	Pdha1	Pyruvate dehydrogenase E1 component subunit alpha, somatic form, mitochondrial	124.753	11.54	43227	3	33987.57744	32313.97165	23120.63496	22969.56328	32869.22939	31397.49368	30156.15611	29876.72122	34149.15273	31510.24747	33896.28818	29168.86503	35101.94501	31079.60055	31120.92773
P26350	Ptma	Prothymosin alpha	360.673	36.04	12254	6	142795.4187	139083.2685	197963.5868	207257.285	180266.6376	191854.2818	167435.3435	180735.149	168030.7203	189576.35	157167.5605	187412.926	143538.4524	162797.4396	152136.6057
P26369	U2af2	Splicing factor U2AF 65 kDa subunit	384.06	29.68	53517	10	132941.7826	134065.9535	152999.0423	143578.2222	136445.8064	114966.9574	137348.805	154753.2565	142563.6049	107424.337	124339.837	150747.9686	127436.8676	134463.0499	133581.3171
P26443	Glud1	Glutamate dehydrogenase 1, mitochondrial	1054.563	57.71	61337	31	913737.5729	955763.393	877551.7605	787663.6201	840218.9485	917540.9779	801960.6971	824319.8661	902040.0207	868725.8202	837504.9653	909063.1173	925996.3051	913842.5128	854177.9045
P26516	Psmd7	26S proteasome non-ATPase regulatory subunit 7	536.353	57.63	36540	13	206425.0202	207311.7006	200838.8152	209144.9366	188912.6269	190314.9242	217051.8088	211564.7696	202880.2714	204604.8909	205495.1367	208899.334	195094.3557	187862.697	190127.9738
P26638	Sars	Serine--tRNA ligase, cytoplasmic	962.88	54.3	58389	23	922304.0178	973630.9008	859825.1222	882140.4412	883823.2059	761219.6977	880074.7733	881424.9645	859231.6743	756118.9847	899131.9877	917092.9775	880287.2461	875527.316	890208.6156
P26645	Marcks	Myristoylated alanine-rich C-kinase substrate	25.002	11	29661	2	26471.9111	19583.9153	21101.95052	16117.98193	19713.81939	26968.34363	20838.90826	16138.47882	18969.56107	28344.78498	24932.26152	20596.0485	27216.32093	19329.30899	14566.82868
P26817	Grk2	Beta-adrenergic receptor kinase 1	67.4	6.24	79785	4	23886.69268	26134.2151	30062.96268	28092.16575	25287.53353	27618.15089	27374.64688	25915.83852	25754.22875	23451.87572	24603.06233	28331.42874	26714.9732	26483.47907	27509.03784
P26883	Fkbp1a	Peptidyl-prolyl cis-trans isomerase FKBP1A	125.15	25	11923	2	80731.92229	75777.84802	67591.7669	67413.25197	69154.31673	52411.60406	55013.42145	51183.86796	50696.69556	54434.97847	68629.97543	62812.11626	66173.74992	68019.04416	59267.57151
P27008	Parp1	Poly [ADP-ribose] polymerase 1	679.782	24.85	112660	16	131113.603	139617.7044	144746.7639	137513.1696	135061.4982	115746.974	119658.5663	100576.9302	98007.6382	84542.04082	122749.4458	124316.9855	127622.8954	133563.7992	124541.9468
P27546	Map4	Microtubule-associated protein 4	360.388	16.8	117429	13	152503.1854	155412.944	137253.0915	151441.3338	154751.0387	135121.5111	129334.1391	132570.6304	137573.444	145037.3747	144843.1473	129930.622	141305.6092	148738.8224	152038.8241
P27601	Gna13	Guanine nucleotide-binding protein subunit alpha-13	295.529	24.93	44055	7	47104.56784	51459.54665	42397.60855	35682.22844	40034.24124	38938.21533	43904.53967	40201.13927	43630.28559	46020.45322	38725.3356	42492.01676	48712.02855	46855.62846	39782.34741
P27612	Plaa	Phospholipase A-2-activating protein	103.381	8.19	87221	4	1830.762044	11460.7909	1395.904914	3104.00446	7268.662078	5426.006839	3974.590268	10701.55595	6579.926772	2002.736323	3083.654009	7471.346469	2980.649488	4688.116909	7667.036636
P27641	Xrcc5	X-ray repair cross-complementing protein 5	78.459	3.28	83057	2	7373.605752	7184.696672	7029.969925	7383.175798	6996.046322	7088.023613	5870.572264	8563.494903	6441.798556	6151.125783	6525.117267	8476.227577	7068.611741	6542.770451	1399.698453
P27659	Rpl3	60S ribosomal protein L3	722.272	44.42	46110	23	772497.6472	776790.4159	800106.4163	779532.4462	774436.9286	912640.3094	1010706.91	998739.6515	957444.3796	923380.0922	844734.6422	877673.6111	803867.5175	767754.5483	854650.4224
P27661	H2afx	Histone H2AX	358.821	52.45	15143	7	13682382.1	12537895.74	13951678.86	13323869.76	14337292.89	16254877.88	14496492.17	14779639.49	14421095.78	15782729.31	13634208.8	13065821.7	14172149.71	13688429.54	14755760.92
P27704	Mapk6	Mitogen-activated protein kinase 6	43.662	2.5	82275	2	57331.89344	51417.91177	49422.34683	46751.39745	53781.19537	45234.5228	41639.54242	49397.37045	44867.5942	54061.9433	52452.93192	47569.72753	48767.61121	58351.30808	53773.74301
P27773	Pdia3	Protein disulfide-isomerase A3	1310.895	67.33	56678	34	1870878.994	1902343.223	1797064.463	1818613.719	1824297.691	2082046.25	1966529.681	1992033.614	2065410.821	2015997.515	1903671.952	1906018.64	1988455.807	1942544.743	1901172.104
P27867	Sord	Sorbitol dehydrogenase	47.078	7.28	38235	2	39365.22334	40272.98312	38100.45724	42193.89086	40845.44024	29678.28599	38779.27462	29983.54899	31249.29998	30262.12598	34927.42897	37422.06118	36511.1788	34664.32159	35405.87432
P27870	Vav1	Proto-oncogene vav	714.634	30.77	98137	20	251441.814	227767.3696	280648.6293	247574.6305	267598.2477	295496.2839	293335.2485	291719.7112	284236.9904	279966.6855	284813.4707	258898.948	264285.1598	279526.0985	254629.6278
P28063	Psmb8	Proteasome subunit beta type-8	321.323	23.91	30260	7	145714.1554	136934.6803	135536.58	154237.3557	164972.7983	135761.6404	155907.5984	147187.218	161677.4688	142690.4243	153865.3255	161734.8827	157545.0763	155958.5202	147108.8509
P28271	Aco1	Cytoplasmic aconitate hydratase	290.564	15.86	98126	8	54424.82992	49271.1726	51139.21027	51461.99268	46483.63635	38684.67758	47520.79648	41035.61227	41259.46467	38712.05961	44727.69601	64546.66645	46252.41988	47934.7623	46763.40336
P28352	Apex1	DNA-(apurinic or apyrimidinic site) lyase	249.51	38.8	35490	8	71974.45361	75384.21672	59284.70183	69387.44666	72215.51146	55391.69226	49640.40897	68726.54413	62552.655	49051.50837	62039.63489	54354.20113	65719.51745	67404.51921	56783.92063
P28474	Adh5	Alcohol dehydrogenase class-3	574.766	62.3	39548	15	275593.1006	288504.4424	261883.7933	275976.7131	280863.4469	278189.9968	281821.7288	275056.5241	298370.5414	297952.0015	241388.0799	261550.5945	265120.2046	275688.2241	258580.6763
P28650	Adssl1	Adenylosuccinate synthetase isozyme 1	216.936	24.07	50254	7	37206.90853	32330.61653	29438.64611	38154.03118	26164.02455	17641.39308	25055.08173	20924.85178	26352.70078	29027.04495	33919.4659	28114.55732	28022.66194	30401.84341	30488.86461
P28656	Nap1l1	Nucleosome assembly protein 1-like 1	243.648	20.2	45345	7	72953.46978	66162.81875	68892.73312	65754.52489	59881.20502	61144.32755	66725.78861	65863.10736	68442.79861	63516.11912	71851.12668	68692.74874	66010.20543	66604.79656	59367.86552
P28658	Atxn10	Ataxin-10	372.172	28.21	53707	9	79924.35117	80202.61744	77304.27889	75972.00102	69613.89022	74534.37052	82410.12271	88025.36223	82716.02146	71933.79126	77184.56801	77935.32636	72366.61469	73179.87933	74092.68019
P28667	Marcksl1	MARCKS-related protein	68.906	17.5	20165	3	18449.00482	21605.00114	25940.62981	19393.36249	29338.10405	42612.19001	34104.00959	39635.16944	33583.92512	27901.91873	28140.71552	20386.26431	30997.75193	30012.88525	23642.82562
P28740	Kif2a	Kinesin-like protein KIF2A	100.667	5.53	79756	4	20494.91704	22409.2943	17569.59153	20612.50481	20786.37245	16770.32097	19948.65891	23867.56578	15738.40602	23471.25493	20137.69101	20242.72419	15922.3442	15010.54431	16685.91204
P28798	Grn	Granulins	274.292	20.54	63458	9	164623.0782	114631.4527	122420.3782	116267.024	123277.4252	117579.9411	102307.7994	74869.93207	86591.10322	57644.12119	73503.57186	78936.20588	68454.86124	83480.74571	92731.87266
P28867	Prkcd	Protein kinase C delta type	208.842	17.8	77547	9	119460.3491	87404.82016	113772.1353	117303.799	113990.121	106155.9261	118251.2971	114622.955	112382.0836	122101.5579	108662.8504	87733.14407	119073.9489	121306.7661	95245.64127
P29066	Arrb1	Beta-arrestin-1	207.183	21.77	47020	7	78022.64456	88633.78078	79214.40762	82500.46948	86466.60446	72331.25649	72615.92431	67453.05387	71257.56921	65498.79564	72806.26903	65908.87229	75273.76433	82873.21372	74399.67488
P29067	Arrb2	Beta-arrestin-2	367.675	39.76	46340	11	164546.31	165950.7921	159366.5026	156624.1905	159255.3202	168843.4353	174107.9897	159360.0285	168270.0318	149035.5087	143867.551	143869.3549	153135.3356	167787.3987	172691.7552
P29314	Rps9	40S ribosomal protein S9	270.149	44.85	22591	13	871313.8351	788713.381	822263.1546	700696.2453	923844.974	1021702.414	971923.3164	1038348.881	1035510.696	877155.6869	895855.4208	931048.5386	1006616.729	873367.1854	877641.596
P29341	Pabpc1	Polyadenylate-binding protein 1	1065.199	46.86	70671	26	709188.626	725754.3343	739722.0161	708362.8478	691330.1313	719866.8215	756605.3939	722712.2625	716404.3978	695659.4022	769538.5218	713210.232	707218.9587	733514.5799	691595.7817
P29351	Ptpn6	Tyrosine-protein phosphatase non-receptor type 6	1259.199	70.42	67559	36	1041504.999	1125985.022	1158329.183	1078731.118	1125694.406	1315413.574	1349467.836	1465120.146	1356146.847	1206933.624	1137738.042	1190665.928	1137522.18	1130932.912	1151942.412
P29391	Ftl1	Ferritin light chain 1	365.894	55.74	20802	7	162576.0698	151912.453	166625.7269	166080.919	132450.5862	292465.6953	259242.4945	244458.3197	273510.6645	193896.1399	256148.7079	215396.3243	175080.7133	179058.599	211659.4683
P29416	Hexa	Beta-hexosaminidase subunit alpha	457.711	32.95	60613	14	275646.9831	282418.3918	241574.7749	273774.2175	243207.9628	215597.6725	212297.7204	192507.6435	217249.1343	243877.2641	253763.9097	234883.4369	261685.3631	263309.4595	260404.9592
P29418	Atp5f1e	ATP synthase subunit epsilon, mitochondrial	2.148	29.41	5767	2	38083.23535	34817.90261	23287.43654	27428.07009	36777.75732	25392.44747	17680.00033	20018.91437	17471.07307	19824.16944	19841.43185	16387.5103	27687.9518	17246.06555	20792.65299
P29452	Casp1	Caspase-1	384.371	30.85	45640	12	81292.57995	95166.68702	102453.0321	90078.91926	81927.76346	94105.11502	74505.77801	95008.9027	98734.45393	93306.09776	73047.42831	100450.3016	94277.60768	75466.19529	98463.85605
P29758	Oat	Ornithine aminotransferase, mitochondrial	625.5	51.94	48355	17	358953.7813	371667.0877	367766.4725	343532.5299	364841.3335	474357.6555	456349.4801	452170.0411	477032.31	430400.987	364466.2783	370451.2349	384187.4228	380607.6927	383616.6078
P30204	Msr1	Macrophage scavenger receptor types I and II CD204	167.547	11.79	50170	6	79911.16436	84128.74387	76070.15788	85557.94118	106242.1182	126848.816	115762.351	133815.1953	124084.9888	99345.2374	95079.39766	110993.6232	105526.4977	93649.48812	103735.1532
P30285	Cdk4	Cyclin-dependent kinase 4	59.271	8.25	33751	2	4497.289197	0.007562416	4689.573009	4179.483734	4142.293116	4520.663983	5560.90995	4034.32245	4258.620545	3056.89132	4810.001537	4184.850604	5179.392506	3967.116216	4758.875766
P30416	Fkbp4	Peptidyl-prolyl cis-trans isomerase FKBP4	472.356	42.79	51572	17	295880.6256	350266.0865	350100.964	360644.1585	368119.507	370468.5496	407572.316	430990.0934	393557.3733	347311.1795	334349.5549	376191.3003	368409.9148	373681.3022	366379.3196
P30681	Hmgb2	High mobility group protein B2	399.936	29.05	24162	10	426500.2207	436953.604	436718.5856	420680.8075	408372.5869	321769.1489	318622.2067	293303.2283	302331.4031	304808.3546	393450.6772	372920.0354	367677.5777	345896.496	335484.0805
P30993	C5ar1	C5a anaphylatoxin chemotactic receptor 1	103.201	11.68	39023	4	80478.04935	89844.00173	79017.86616	77708.34773	85848.47526	62784.04616	58637.63069	55167.11117	62050.08881	70702.93273	77445.40884	66871.27247	81688.09141	76141.05642	80317.12527
P30999	Ctnnd1	Catenin delta-1	263.815	16.1	104925	10	54413.13208	60238.48279	59511.0236	62987.45619	55207.7669	34255.00409	50910.33531	59158.68967	50003.35082	57483.97258	54389.65715	59965.89032	53814.07506	55990.22557	60185.70127
P31230	Aimp1	Aminoacyl tRNA synthase complex-interacting multifunctional protein 1	84.573	19.68	33997	4	35013.63179	33512.14945	40687.09298	33584.23761	37731.02577	30339.95409	45582.80844	35346.00279	39571.92302	30655.85073	35183.1402	35033.78107	24076.24659	30674.81011	34387.22261

P31266	Rbpj	Recombining binding protein suppressor of hairless	94.601	7.22	58537	3	87533.37408	87023.36435	93910.61563	86717.39796	97345.20222	95254.12267	98804.50643	99780.46412	103599.8901	88116.71278	90378.46938	88831.86808	94150.76344	91268.42909	98182.40498
P31750	Akt1	RAC-alpha serine/threonine-protein kinase	63.158	5.21	55707	2	10429.78085	11377.05889	10694.76046	11704.50065	11061.42203	9216.319031	10513.967	10550.53047	9865.373992	11867.97937	10457.28144	12780.02785	10986.71329	11349.78485	10900.00786
P31938	Map2k1	Dual specificity mitogen-activated protein kinase kinase 1	480.979	39.95	43474	11	151520.8787	157152.7987	152128.7422	141490.613	173557.6202	127616.9827	134377.9103	132987.3739	134454.3956	127764.1692	167020.3063	149778.0012	149904.7812	148689.3374	152200.861
P31996	Cd68	Macrosialin CD68	137.842	16.87	34818	5	847693.2623	821643.5875	716359.4203	729747.7338	742959.8765	859681.0093	795964.5744	786611.4012	834496.0146	860294.0886	772058.7252	718728.7955	838701.1744	802406.813	905626.4074
P32020	Scp2	Non-specific lipid-transfer protein	78.683	5.3	59126	3	79426.95404	66278.01703	65416.77269	67246.76892	76792.44981	64886.443	43302.8047	39982.55408	56233.53303	50995.69955	63970.14425	49543.4773	58031.74408	56308.97514	52139.19562
P32067	Ssb	Lupus La protein homolog	467.172	32.29	47756	13	321847.5466	305189.249	307609.1502	349324.7055	320476.6832	281428.6499	300963.6665	298532.5258	306603.3395	302003.8958	308330.6162	333728.982	307832.2152	325468.3817	303468.6526
P32233	Drg1	Developmentally-regulated GTP-binding protein 1	186.528	19.89	40512	5	22331.74255	25073.43316	24928.9743	25902.48451	21178.86643	18578.8939	27809.86885	30941.71127	29382.84567	23236.70309	23879.36724	22990.70485	13881.34948	18546.56639	24332.59885
P32577	Csk	Tyrosine-protein kinase CSK	379.565	33.56	50746	12	111256.1426	102382.8802	101167.7097	104318.6626	104923.6806	92764.14567	91678.70832	96397.42062	97598.95389	96985.14004	96891.86201	107272.5255	107049.0308	100957.6783	99924.74732
P32921	Wars	Tryptophan--tRNA ligase, cytoplasmic	342.969	29.73	54358	10	87427.28726	98066.28357	89703.97972	89321.5787	70348.04728	80172.54124	99711.21784	95246.19281	89510.49179	94386.15845	91881.05801	110293.7403	99435.41346	99670.11259	101995.805
P33609	Pola1	DNA polymerase alpha catalytic subunit	28.801	2.87	167340	2	5135.273379	4867.209505	4306.025865	4845.784728	4536.868026	2892.219897	3882.534678	3425.155214	4884.578109	3571.840155	3206.630235	4804.483985	3739.917579	4455.37658	3995.176095
P33611	Pola2	DNA polymerase alpha subunit B	42.166	6.83	66214	2	8140.260636	8310.837737	8082.567389	5981.989364	7591.734249	6737.163277	6812.554371	8356.532601	6431.620598	7879.493296	7864.791506	8268.7156	7188.004714	7986.404806	7459.864565
P34022	Ranbp1	Ran-specific GTPase-activating protein	104.906	35.47	23596	5	114958.1663	109654.2866	116325.7954	118573.2081	117619.1555	102447.7611	129762.4582	122030.9946	116113.0226	111888.8374	120521.6977	123600.4102	113581.4894	115935.5274	116650.4278
P34884	Mif	Macrophage migration inhibitory factor	364.546	71.3	12504	8	401394.4982	341766.627	343561.3741	337389.9815	305590.9954	278252.6925	245459.5229	230377.1346	263141.5236	220172.3931	292495.3887	278395.2264	245761.2307	246844.516	184719.8363
P34960	Mmp12	Macrophage metalloelastase	124.013	12.26	54971	5	32006.70263	32104.78011	20548.53989	25036.66584	20757.08031	52248.96914	41840.64967	46870.33421	48401.14206	38066.06052	44183.64129	40111.12956	37870.80765	42418.13708	36213.88839
P35123	Usp4	Ubiquitin carboxyl-terminal hydrolase 4	358.208	16.22	108343	12	51568.34604	54013.06059	42174.91957	49647.52651	52892.09892	47942.3896	43384.02061	52238.54391	55871.15225	46456.11658	42005.1533	46286.22841	36455.74822	54630.63562	43320.61878
P35276	Rab3d	Ras-related protein Rab-3D	80.876	11.87	24416	3	58983.41663	54893.74549	48042.02511	55518.81203	67863.61112	65666.14701	61902.71542	66175.23107	71341.04124	59381.31332	48205.29474	54083.62302	58529.697	57447.09289	55373.08571
P35278	Rab5c	Ras-related protein Rab-5C	510.872	58.8	23413	10	413151.0831	395009.361	387205.885	400086.3239	393951.9694	346599.695	387108.4247	377406.9646	396771.0951	387021.1876	404274.5294	378123.6271	380486.4517	405204.502	402500.3734
P35279	Rab6a	Ras-related protein Rab-6A	196.33	28.85	23590	5	57130.65671	63832.43011	54489.12625	57769.87776	56104.75312	60565.80347	60268.49983	60789.30932	63712.27951	64093.61583	68729.67059	60488.00386	63477.95175	63047.51546	61853.39682
P35280	Rab8a	Ras-related protein Rab-8A	324.08	44.93	23668	10	114906.1923	92005.67288	95754.33186	77769.10629	109114.8596	105326.7287	103678.9616	114871.5001	103175.1228	93134.17411	104644.1942	96412.39354	121976.6588	88589.19276	94131.57531
P35282	Rab21	Ras-related protein Rab-21	103.17	22.97	24106	4	21632.16677	23314.8709	25835.30747	25374.44447	22206.6454	14715.94727	24233.86652	26283.89301	22442.72673	21182.9667	23312.18509	23956.81394	22137.74129	24909.70539	19621.91813
P35285	Rab22a	Ras-related protein Rab-22A	73.206	22.16	21802	3	34711.13299	33617.97033	29779.92376	30305.75377	36030.89564	28900.58846	26219.20634	29841.32142	29343.13966	28837.80505	26251.15037	29749.83425	30037.30764	31197.40527	33845.49197
P35286	Rab13	Ras-related protein Rab-13	40.06	12.81	22901	3	63762.85987	52372.45878	51522.92593	54181.00022	57519.40114	59346.69218	57830.31215	63593.736	59164.73914	62578.75759	56782.58627	54068.75116	57992.6498	55520.49609	51529.15875
P35293	Rab18	Ras-related protein Rab-18	80.318	18.93	23035	3	8883.651403	10849.1569	8617.629248	8225.590366	10949.84739	15499.80422	13246.80861	14203.1178	14683.58827	13584.23377	9531.331516	10850.10114	11772.55421	12140.42704	12691.83156
P35434	Atp5f1d	ATP synthase subunit delta, mitochondrial	85.494	28.57	17595	3	109105.2932	32361.43337	110720.2982	87468.6214	71595.68776	104718.6379	92556.24646	33360.32628	101582.5445	89537.80356	108712.867	87077.46913	87467.8637	84520.20547	79825.30075
P35505	Fah	Fumarylacetoacetase	102.778	9.31	46176	3	11898.57245	22341.43108	22426.76523	21300.9658	14628.19905	18810.40023	12282.58498	20307.40175	14896.83588	19848.36223	21514.09366	16882.20782	11615.80425	16370.30567	25172.82864
P35564	Canx	Calnexin	771.865	29.1	67278	17	388870.5937	386615.1703	367497.7603	367987.241	374049.335	472574.3201	444755.0533	454753.8483	473423.1474	473101.1905	383442.8286	408923.005	429925.21	401461.0398	401837.0581
P35585	Ap1m1	AP-1 complex subunit mu-1	127.778	15.37	48543	5	43590.49539	41221.90748	39386.87804	17325.43109	40687.11184	34727.80202	40470.61734	29503.28924	42208.61169	42473.90018	42541.74095	41478.0904	46030.82021	51046.83894	36406.08966
P35700	Prdx1	Peroxiredoxin-1	593.265	64.82	22176	20	2205034.841	2258420.958	2224902.868	2187989.53	2232499.53	2630003.367	2578725.535	2547737.283	2620900.357	2499457.002	2555874.66	2496023.888	2537582.409	2467131.324	2306640.188
P35922	Fmr1	Synaptic functional regulator FMR1	106.209	6.51	68989	3	39571.95937	33354.41258	37374.44399	32652.90515	34219.26734	36515.42674	37845.68676	37600.40465	39595.54302	38436.35838	34380.73905	32372.26458	37973.85142	38345.48074	37661.56754
P35979	Rpl12	60S ribosomal protein L12	358.578	58.79	17805	7	620494.5819	646830.7255	603865.047	599613.9552	655884.3428	650830.7488	619855.9714	605742.235	564828.7492	569881.8323	584131.6691	592725.6658	576467.7457	577517.0523	621040.1011
P35991	Btk	Tyrosine-protein kinase BTK	95.635	3.03	76437	3	5849.289465	43782.92334	14504.6117	38088.04738	33941.10259	26489.01006	32105.30409	34367.57684	17240.56585	32933.53276	5628.974782	38590.8627	22816.30596	4921.054592	4251.823714
P36371	Tap2	Antigen peptide transporter 2	125.645	7.69	77445	4	16575.79217	21245.61814	10744.66018	28752.57922	23799.3049	33245.68757	28645.8729	21558.13574	14509.24312	30126.91609	10779.59391	30984.06696	28724.65669	26503.60453	19556.55168
P36506	Map2k2	Dual specificity mitogen-activated protein kinase kinase 2	178.952	24.75	44282	8	97626.55282	96717.5343	98153.60796	88827.32958	119230.0722	89609.66348	91819.78346	86900.34493	93266.50385	86654.05636	113910.0019	93913.96705	95078.39042	91750.74023	100609.861
P36536	Sar1a	GTP-binding protein SAR1a	121.468	27.27	22371	3	7633.222725	7251.516778	6539.004983	6785.529916	5861.205884	4614.056749	5644.679652	7437.40224	6863.69303	1106.544357	5038.667743	5950.117879	1144.738928	5009.375338	4591.781982
P36552	Cpox	Oxygen-dependent coproporphyrinogen-III oxidase, mitochondrial	58.206	7.45	49715	2	4976.339017	5981.715792	4596.213245	6193.350012	4395.232037	3498.577005	2688.871529	4106.340998	3789.934665	3594.717576	5659.808184	4847.00282	5942.342985	4223.296399	4645.626984
P36860	Ralb	Ras-related protein Ral-B	252.646	23.3	23317	7	64769.98543	78384.03797	45275.82786	78059.86696	77771.55733	57192.83536	58204.71171	53282.19756	62073.86891	67375.64994	85848.47927	73596.7749	78199.20768	79063.01808	66368.60107
P36876	Ppp2r2a	Serine/threonine-protein phosphatase 2A 55 kDa regulatory subunit B alpha isoform	291.297	21.92	51678	8	99527.83167	106189.9818	114684.3837	113471.6441	106068.4029	117733.7285	123081.2422	130067.3641	124616.537	114441.2916	108078.1975	112476.4251	110744.4485	111513.2796	108121.2045
P36916	Gnl1	Guanine nucleotide-binding protein-like 1	100.222	6.43	68771	3	14485.54206	13608.89508	12087.95148	12983.09121	13244.18843	12250.16719	14195.17296	12793.44575	14803.49927	12266.60928	10597.00855	11230.3207	16185.21878	12942.77854	13369.25129
P37040	Por	NADPH--cytochrome P450 reductase	191.7	19.17	77044	9	80717.92107	87521.09231	83672.94223	88574.4364	85550.42492	89831.21368	94789.02962	92441.04404	91910.93514	79656.34278	93107.64637	90030.99071	88121.34504	90170.49132	85862.69886
P37913	Lig1	DNA ligase 1	423.733	18.56	102290	11	77901.3637	85607.03733	94133.5281	78295.53681	86856.67861	87359.62952	95980.44745	95888.86741	93735.59906	82580.7765	77987.04561	86661.47379	83896.70979	74662.48208	84658.86507
P38060	Hmgcl	Hydroxymethylglutaryl-CoA lyase, mitochondrial	443.427	42.46	34239	10	144571.8136	132691.3937	128712.7387	130968.6242	121069.0522	118617.2394	94448.86003	100505.5334	109072.4681	116804.5192	136288.2249	116758.9399	130041.3935	135254.7157	112934.8094
P38647	Hspa9	Stress-70 protein, mitochondrial	1481.185	53.31	73461	32	1360334.854	1104318.398	1458940.336	1284415.862	1138226.903	1595825.354	1627915.356	1684973.433	1589337.171	1521921.519	1402795.385	1275326.747	1452655.857	1475184.752	1207720.492
P38718	Mpc2	Mitochondrial pyruvate carrier 2	76.703	16.54	14258	3	10739.83657	11813.17017	6021.981107	10782.196	8000.484032	9147.90564	7159.253958	7450.89631	12696.61878	9524.478969	12279.61875	11080.56962	12712.04831	12522.1511	8009.099363
P39052	Dnm2	Dynamin-2	613.186	30.46	98230	20	248130.8292	257337.3376	236747.6549	242664.7702	223086.4981	219027.7711	236144.7231	221019.7973	244828.184	235624.3694	241631.13	248592.3172	242329.5682	228772.6378	249073.2949
P40124	Cap1	Adenylyl cyclase-associated protein 1	1729.781	73.42	51565	40	3737957.281	3716568.202	3499529.43	3671683.486	3622441.138	2768390.387	3024615.5	2987648.043	3104018.912	2788756.969	3444171.458	3515046.781	3361669.328	3541279.966	3382124.704
P40142	Tkt	Transketolase	1581.91	65.17	67630	37	3111043.928	3417445.016	3341239.365	3308876.681	3384224.109	2734597.799	3116461.277	3121888.925	2982078.785	2839425.469	3324227.26	3384527.462	3245756.614	3181910.314	3286665.317
P40240	Cd9	CD9 antigen	166.588	26.11	25258	5	73761.65787	85775.24058	55502.48879	79521.83259	76207.02861	80939.81373	85242.15263	90737.06969	92912.89562	81437.09859	59775.39217	66807.89499	72826.28279	73632.25643	82659.72426
P40336	Vps26a	Vacuolar protein sorting-associated protein 26A	85.835	19.57	38114	4	19705.82227	21533.38318	20724.37827	25762.73524	24108.67946	14245.55932	23464.5643	26182.27957	23316.42659	25044.88516	20320.59622	22843.35393	24920.78167	23243.07518	26308.29404
P41105	Rpl28	60S ribosomal protein L28	142.453	39.42	15733	6	165760.5007	188442.5357	168338.444	180391.5458	192587.479	205060.5344	234811.9276	229324.1875	212804.8646	199990.4718	179680.0299	191684.3548	181215.3119	167108.1687	188810.0667
P41123	Rpl13	60S ribosomal protein L13	166.598	28.44	24309	6	166051.8281	174917.8207	170590.6778	160601.8449	165662.8156	239508.6343	256512.1975	281792.6027	237940.9677	223293.3262	170949.4541	184039.1703	188305.2455	170753.7011	155112.6872
P41216	Acsl1	Long-chain-fatty-acid--CoA ligase 1	279.735	22.46	77951	14	155258.2345	161589.2899	157425.1956	151289.5796	162003.1893	196997.983	188014.0509	165528.891	188566.3034	204169.9868	147606.8193	149437.5426	166639.0958	175625.4938	158689.1445
P41245	Mmp9	Matrix metalloproteinase-9	146.64	9.73	80535	5	27823.55147	35569.68614	26796.28939	27944.97642	29263.06658	25266.01301	25840.74104	30556.29536	26842.44649	25573.18042	28002.35329	26371.01111	27702.54409	26166.82409	28362.18966

P41498	Acp1	Low molecular weight phosphotyrosine protein phosphatase	157.634	24.05	18152	3	71540.28409	66287.49274	65201.19237	44585.71469	69908.94005	43708.98523	64456.13289	58783.43163	58791.15103	39596.30357	50054.93355	69703.08394	71803.09092	70231.87491	66680.01448
P41565	Idh3g	Isocitrate dehydrogenase [NAD] subunit gamma 1, mitochondrial	188.881	21.63	42851	6	66681.67882	71470.75734	62607.10562	64038.24914	70945.32171	77309.50934	74037.16576	73613.54286	77873.97875	65270.78068	56739.03904	58345.60072	55552.37978	61266.89617	64897.06966
P42082	Cd86	T-lymphocyte activation antigen CD86	79.02	10.68	34666	3	60447.65496	53914.11016	54687.56969	48781.6551	52439.08852	67337.94769	69593.14134	64194.54323	61205.49027	69438.48735	56407.34144	50082.47792	68320.79077	60669.92691	59274.2488
P42125	Eci1	Enoyl-CoA delta isomerase 1, mitochondrial	293.464	37.72	32250	8	153728.0868	158000.3035	126227.8733	111838.3531	140363.2367	126925.6443	141385.7435	127342.3743	139625.3844	149899.7757	160238.5897	141118.1884	165534.2126	149997.6976	144097.0985
P42208	Sept2	Septin-2	454.811	50.14	41526	11	240634.5483	302625.3178	263036.167	249265.5619	260506.7359	255244.7975	264207.322	288498.3535	259598.4328	272844.9677	269763.429	284565.217	293633.6275	258491.0384	274684.1205
P42225	Stat1	Signal transducer and activator of transcription 1	225.904	10.55	87197	6	28022.80977	16771.50394	25780.99634	20948.58907	22077.08219	33751.3309	23088.20288	34298.17628	32674.2938	20948.73787	13561.77166	27055.0893	16728.47426	30040.16916	23011.69753
P42227	Stat3	Signal transducer and activator of transcription 3	196.162	14.29	88054	6	26189.88148	32764.59331	18233.21735	29357.19798	28092.38951	12967.53821	21135.29633	34656.92691	16083.52265	18307.48634	25167.57106	24248.34805	23458.9087	28040.4583	18408.29504
P42346	Mtor	Serine/threonine-protein kinase mTOR	86.655	2.28	288794	4	7498.400287	5905.048476	4482.734396	4890.135635	5027.794457	4121.690578	4500.043279	4585.1592	4471.828523	4320.682748	6277.872882	5173.701943	16093.0119	5426.029935	5086.676758
P42667	Sec11a	Signal peptidase complex catalytic subunit SEC11A	70.665	16.2	20599	4	44711.07034	41038.39412	41960.47568	45155.3136	43660.67607	57441.27327	51844.56838	55117.32857	55075.44914	54277.0843	45703.84756	48485.17087	50363.41238	52443.84763	49475.16989
P42669	Pura	Transcriptional activator protein Pur-alpha	180.048	23.68	34884	5	49930.04256	68913.73065	73285.71586	65490.63097	64080.26136	56254.7275	74610.6536	61456.2434	56183.01843	76346.52942	70276.40993	61120.84118	50962.55714	48711.5344	60989.75766
P42932	Cct8	T-complex protein 1 subunit theta	1318.426	61.86	59555	32	1006932.541	1079858.884	1130846.054	1045364.662	1062158.121	1180823.071	1235372.183	1262936.64	1144391.002	1080583.34	1050088.637	1119871.257	1067529.514	1127274.67	1087329.301
P43247	Msh2	DNA mismatch repair protein Msh2	478.624	18.93	104151	13	59481.13871	84647.54071	62844.96767	71285.83302	72835.60793	65670.22542	63988.69375	69138.92041	75661.49248	57489.18743	55553.59607	70811.88857	72497.28545	64809.43011	74328.97022
P43883	Plin2	Perilipin-2	41.644	6.35	46646	2	2862.46571	3099.207689	3201.594263	3710.156495	2360.914013	10370.13754	5882.534046	14181.61047	15895.60786	5402.315625	4689.272033	8590.821611	3705.078732	7312.421931	4161.43457
P45376	Akr1b1	Aldose reductase	575.159	56.65	35732	20	1388197.913	1424876.979	1389402.603	1378776.1	1440627.996	1232236.035	1275814.028	1257179.355	1274101.423	1152637.878	1363924.349	1390569.754	1265539.02	1416766.965	1337986.652
P45377	Akr1b8	Aldose reductase-related protein 2	465.668	55.7	36121	16	568552.4476	560424.4997	537070.43	501679.7013	527493.434	567008.6307	559515.4491	577958.7401	561341.0971	512283.5326	509674.0814	514662.081	510380.7989	526913.7961	554295.6449
P45878	Fkbp2	Peptidyl-prolyl cis-trans isomerase FKBP2	130.253	30.71	15344	2	12292.46687	9824.060104	7654.866602	9439.827292	8929.925646	7658.324999	10184.0953	9759.647652	11880.68657	27300.6788	10102.43428	10013.20602	9906.760147	8979.826379	10742.37153
P45952	Acadm	Medium-chain specific acyl-CoA dehydrogenase, mitochondrial	283.311	25.89	46481	8	157402.2314	166453.4508	139334.7744	152510.7487	148988.8919	159572.6847	153390.7077	153999.6299	151207.0932	156393.3402	172410.4427	160332.2185	161364.3064	161079.3929	155783.3377
P46061	Rangap1	Ran GTPase-activating protein 1	236.691	19.02	63531	6	25548.81551	38497.69091	24143.76181	29266.60353	26883.80935	29917.82918	21593.53586	27558.56708	35254.79472	26625.58175	25436.39192	29450.117	28000.20566	32194.09164	24551.1622
P46062	Sipa1	Signal-induced proliferation-associated protein 1	62.107	4.92	112066	3	9620.672436	11297.47391	9173.998648	10755.78666	10503.85471	9334.176828	11475.45476	9768.066257	8593.697995	9543.409419	9978.323271	10067.69616	10524.53466	10233.71928	11056.84644
P46413	Gss	Glutathione synthetase	90.551	11.6	52345	4	19642.55559	21252.37326	20333.9914	20354.51708	27530.47436	13867.27641	9100.353821	17553.50753	16363.89348	19654.30043	17179.22925	16726.26845	20690.9659	20661.32189	20143.63893
P46460	Nsf	Vesicle-fusing ATPase	751.067	32.53	82613	21	302632.0119	338094.8927	316006.2361	357751.0421	292728.8229	336766.1378	328110.9965	350709.8552	334193.2885	333711.4285	316026.7925	325509.3924	328839.4773	309748.7868	306503.5916
P46462	Vcp	Transitional endoplasmic reticulum ATPase	1627.367	63.28	89349	43	1236682.899	1298652.033	1178802.97	1256048.831	1199860.586	1033930.386	1225597.921	1094622.52	1149565.108	1089240.715	1192705.267	1271033.364	1086801.282	1185399.612	1207292.814
P46467	Vps4b	Vacuolar protein sorting-associated protein 4B	260.717	23.65	49419	9	105067.5775	98706.16982	103746.0005	122403.7672	118518.6214	119387.6089	92726.79123	95351.9791	116820.372	111622.9931	99632.11891	111504.6915	108985.6523	119710.5549	94531.35271
P46471	Psmc2	26S proteasome regulatory subunit 7	451.861	44.11	48648	15	153468.7762	184504.6799	162572.7333	159442.7091	166891.238	170862.5918	180132.7361	175056.7527	137702.3254	141510.3623	168579.2521	170437.1464	154267.7108	177385.0916	163093.0468
P46664	Adss	Adenylosuccinate synthetase isozyme 2	471.809	35.53	50021	12	106395.1353	122381.386	108875.1935	122969.184	109948.5293	105169.03	119375.354	130125.6452	119961.3397	109755.7627	105612.4201	113340.0977	105732.2762	107538.5342	110316.1312
P46737	Brcc3	Lys-63-specific deubiquitinase BRCC36	120.438	14.78	33340	3	19565.68048	20884.62811	20622.24413	21674.90838	22162.11928	18028.97506	22806.85216	17098.82473	16864.60637	20298.41471	18958.08772	20900.93287	19934.32305	18112.3821	21536.09965
P46978	Stt3a	Dolichyl-diphosphooligosaccharide--protein glycosyltransferase subunit STT3A	246.339	18.44	80598	11	103439.3636	117572.2039	102742.7803	102923.1141	100679.2695	134838.1886	124961.0875	117650.3619	132947.6577	125738.1954	107043.8059	109389.2199	120317.8035	99964.26149	118034.2583
P47197	Akt2	RAC-beta serine/threonine-protein kinase	39.141	3.33	55543	2	3958.240599	3742.844171	5167.034772	4136.57989	4842.857229	3487.86728	4111.923391	4214.378611	2476.574185	3433.91941	4755.710807	4216.690187	4613.666666	4530.712874	5239.064242
P47199	Cryz	Quinone oxidoreductase	56.587	9.97	35269	2	22682.62043	26512.87659	22463.61141	19147.65487	19328.81221	20600.82749	18598.24985	20840.27691	17821.06535	18603.69732	21847.02036	18135.51943	21316.89875	20961.09968	21254.91405
P47226	Tes	Testin	241.991	30.5	47983	8	86354.12068	71274.56559	75882.50819	101849.7832	70489.97208	66638.15441	89765.12086	72145.10895	73039.38544	76682.15151	78085.96831	63521.05636	63597.11351	75234.70883	70956.78411
P47713	Pla2g4a	Cytosolic phospholipase A2	413.173	26.07	85222	13	130722.2104	138157.0389	120746.0716	139694.4249	142948.4667	122217.3519	146811.5889	145472.4887	148507.3141	125814.4801	126174.0187	146445.9175	123053.2454	134094.1108	134205.0038
P47738	Aldh2	Aldehyde dehydrogenase, mitochondrial	996.969	62.62	56538	25	793715.0113	785429.5986	734327.8388	786447.2383	756560.8514	814239.1926	738122.1978	719269.7785	811527.6228	800100.9631	749229.0337	740667.3369	794393.603	763123.1357	780932.9913
P47740	Aldh3a2	Fatty aldehyde dehydrogenase	180.153	17.56	53971	7	36681.26215	34287.05884	33946.44922	30589.7668	36787.15147	40681.70812	35458.50136	30796.6127	40309.65306	39272.19012	43257.08386	43119.12542	36883.61695	41132.3775	33176.49396
P47753	Capza1	F-actin-capping protein subunit alpha-1	366.653	55.94	32940	11	504879.5592	519825.1567	499250.3251	503365.0934	510819.2458	437325.8333	485327.5455	454978.6962	451747.823	466040.0528	477555.7947	494522.1465	524923.3024	541378.6671	533864.0744
P47754	Capza2	F-actin-capping protein subunit alpha-2	660.331	70.28	32967	14	540284.7193	549555.8563	523091.8023	529975.0139	540276.4526	446637.3244	513903.594	501390.4646	462481.4472	489341.3755	502095.1898	487402.9293	553094.6541	567568.9452	563398.4313
P47757	Capzb	F-actin-capping protein subunit beta	867.592	66.79	31345	21	489360.7092	511178.2301	434624.5778	471727.6472	479991.1297	366934.9421	362326.1238	362791.4046	374227.966	355511.5128	432139.993	491508.6122	461482.6985	439731.6026	414627.6555
P47758	Srprb	Signal recognition particle receptor subunit beta	192.735	18.22	29579	3	20321.01437	26198.74708	20597.56589	22589.90252	21876.90788	24747.92906	25893.68512	28607.5826	25483.24067	22456.96266	23228.11407	24136.7412	22381.02702	21882.76891	24559.25109
P47791	Gsr	Glutathione reductase, mitochondrial	264.542	25.8	53663	7	158738.8463	154395.3302	148312.0414	149466.0524	151390.2727	142899.6205	156838.2288	150124.5024	150301.5279	139363.8275	145803.3267	143803.9894	136273.3918	143589.6847	142754.9273
P47802	Mtx1	Metaxin-1	109.481	12.62	35624	3	12270.58173	12729.06568	10633.04466	13900.44231	6781.015168	9693.552543	10755.39745	13655.29918	13850.32999	13173.23528	10948.09602	10362.10139	10437.40406	13010.83464	11886.0644
P47811	Mapk14	Mitogen-activated protein kinase 14	242.495	36.94	41287	8	61429.45986	68283.29743	69988.02835	69071.82772	69952.30275	64519.96008	69128.95602	75964.75349	67111.34229	71759.07037	68174.75722	67658.64756	65782.24785	70483.9762	66782.86751
P47856	Gfpt1	Glutamine--fructose-6-phosphate aminotransferase [isomerizing] 1	204.461	14.63	78539	7	43193.81739	23129.99252	39423.70282	24747.08366	38356.42893	47827.58334	48774.20365	45836.30728	47513.70187	29547.17161	21908.04643	28091.39364	38508.20043	43947.4116	44281.19775
P47857	Pfkm	ATP-dependent 6-phosphofructokinase, muscle type	183.95	8.08	85269	6	216488.8885	266807.3273	241661.6956	227468.0497	244969.3016	244659.5037	236935.5935	257851.0747	232396.2881	239369.9527	252689.5998	245816.4246	249454.5095	257363.1808	228879.7032
P47911	Rpl6	60S ribosomal protein L6	224.616	24.32	33510	7	66824.10548	61000.07477	64154.57365	64216.08143	58857.27588	129651.1733	163292.7886	144273.3737	154531.6679	147400.7887	68700.38966	69862.60769	75658.00583	64603.02659	93782.13918
P47941	Crkl	Crk-like protein	58.6	12.54	33830	3	16192.77502	15253.97478	14441.91638	16691.37767	7951.637476	12637.03766	14076.78814	16073.84129	15438.85734	15932.62522	9921.326449	16279.4516	17040.86548	14494.2589	14193.61928
P47955	Rplp1	60S acidic ribosomal protein P1	140.262	79.82	11475	4	178336.955	167098.7215	152590.5818	157202.7954	117283.14	109545.6041	128816.9973	146392.5988	153221.273	116288.0689	144187.4606	134216.7778	130874.1326	134880.169	140547.5162

P48025	Syk	Tyrosine-protein kinase SYK	114.19	10.81	71376	4	8072.583069	14322.66707	11619.32177	11646.26987	12844.79349	8903.898726	11750.52394	8828.337218	5409.943048	6959.531257	8379.419986	14170.38107	13385.06453	6476.846761	7970.551465
P48036	Anxa5	Annexin A5	1571.408	83.39	35752	38	5574873.479	5496021.557	5094108.873	5311873.889	5111634.684	5718685.048	5792282.358	5924580.422	5822760.664	5865091.859	5857922.629	5982034.421	5998937.483	6156422.665	6043348.731
P48193	Epb41	Protein 4.1	155.158	8.62	95911	5	47369.69029	50306.86056	54455.06172	54377.95884	51153.89624	40423.99745	48019.19325	52396.62969	40119.16149	43485.72822	49833.52824	54134.39293	50129.11161	50003.45479	46439.70768
P48410	Abcd1	ATP-binding cassette sub-family D member 1	41.003	5.03	81859	2	5718.81156	2294.911012	1841.544829	7240.448756	5908.156996	7167.819413	6000.744074	6159.75128	5477.431785	5979.255411	4999.588288	5410.124372	6201.839922	6968.520865	1949.930286
P48441	Idua	Alpha-L-iduronidase	73.994	3.31	71254	2	5524.020701	5406.639453	4309.623276	5019.397755	4517.101795	3841.350959	3819.495718	3641.933436	3254.537554	4192.027658	3493.898283	4828.131141	4978.824618	5244.102396	5788.491692
P48678	Lmna	Prelamin-A/C	1821.487	59.85	74238	47	2832693.434	2705449.507	2389153.598	2414607.941	2577206.215	2593459.384	2511151.524	2475488.443	2446144.036	2711995.396	2883235.762	2456646.257	2712312.23	2692986.691	2532066.94
P48758	Cbr1	Carbonyl reductase [NADPH] 1	110.481	25.99	30641	4	11401.85707	10400.08836	11687.97148	8401.446829	8803.017847	5913.621768	7898.86353	7297.277625	3506.823145	8387.839914	9456.602394	4990.207289	9802.556742	8656.726895	8142.443106
P48771	Cox7a2	Cytochrome c oxidase subunit 7A2, mitochondrial	84.488	27.71	9291	2	33067.09782	36109.87328	37436.9931	32763.84341	36716.66436	36188.23763	30028.55007	27957.92316	35702.83462	39486.56188	44563.15475	35637.48377	30998.42403	37299.9642	30417.35442
P48774	Gstm5	Glutathione S-transferase Mu 5	104.255	27.23	26635	5	76102.54385	74671.26998	69671.02952	76640.37729	64013.9981	66020.67821	61851.11115	64700.03449	70517.64905	69624.05753	67825.446	68877.8318	68584.09951	69293.30957	74954.91033
P48962	Slc25a4	ADP/ATP translocase 1	705.604	58.39	32904	22	2888003.101	2578678.978	2855141.379	2655645.528	2532936.655	3282575.621	3178345.704	3256407.534	3083208.806	3097823.242	2785614.524	2687891.044	2783705.442	2965831.895	2749786.04
P48999	Alox5	Arachidonate 5-lipoxygenase	576.016	30.27	77967	16	225251.2847	192218.5708	203566.9823	228473.8813	262766.4399	175337.6658	160150.9046	160745.2484	216559.8496	212151.8157	180209.8063	167536.6816	190734.3597	226055.3964	212074.4858
P49186	Mapk9	Mitogen-activated protein kinase 9	56.326	4.96	48017	2	26393.76503	25266.57536	31203.50848	28916.60441	31430.95134	30293.67391	31336.28434	31996.2796	33252.15459	30582.91604	28963.3836	28523.22994	28270.28195	32994.56842	29839.49701
P49242	Rps3a	40S ribosomal protein S3a	727.985	57.58	29945	20	1083636.669	1110727.697	937474.4352	1086150.614	1212456.035	1147364.55	1212069.921	1181991.384	1054458.703	1103664.666	904945.8165	1047413.764	1048886.786	895036.4254	924118.5079
P49300	Clec10a	C-type lectin domain family 10 member A	363.302	46.05	34596	10	327689.2319	325165.3398	271385.9887	292427.0501	329697.0835	282326.2208	267192.0964	248712.6837	258818.7644	273859.3532	256460.9251	266585.5056	299397.8779	307867.5994	332068.6242
P49442	Inpp1	Inositol polyphosphate 1-phosphatase	154.202	11.87	43346	3	9735.588171	13744.87771	9216.002765	15250.96642	13036.41883	8776.548258	13686.17658	10131.34037	11352.47491	11713.62838	12059.51997	13152.22389	12531.53958	12548.63122	9830.333694
P49615	Cdk5	Cyclin-dependent-like kinase 5	54.739	9.25	33288	3	10638.17346	9339.403787	8597.538359	7903.976361	8352.762541	8123.937576	9236.323672	7887.291683	8795.221701	7760.500736	9059.769009	8447.736017	8966.105827	10252.07404	8909.387193
P49710	Hcls1	Hematopoietic lineage cell-specific protein	322.005	24.69	54240	10	139127.0161	142703.896	128709.7906	129538.4181	135306.3991	175869.3717	170139.6594	128732.9405	123613.4105	122480.5572	136168.8482	125793.8097	174996.3532	129176.6629	123167.9898
P49717	Mcm4	DNA replication licensing factor MCM4	823.128	32.48	96736	22	255697.5112	270464.7838	270443.0804	282847.759	271316.446	205171.0951	217216.0435	229738.3065	220709.5021	212721.2404	265645.4639	254713.6527	225763.5616	237138.9262	256873.5283
P49718	Mcm5	DNA replication licensing factor MCM5	742.669	38.74	82343	21	417834.8294	411009.1639	432411.2396	475239.3362	449111.2694	442170.0583	415588.5008	442770.2255	473600.7248	454056.4668	440687.5449	434464.2671	416989.2563	456281.6721	433756.5486
P49793	Nup98	Nuclear pore complex protein Nup98-Nup96	83.503	3.85	197283	5	17402.08848	16104.62177	15322.86402	15683.44013	15042.50457	14675.77562	16399.29069	16162.26615	16210.63686	16700.82371	15367.66711	16499.96626	16453.24444	14968.09481	15396.48696
P49813	Tmod1	Tropomodulin-1	39.829	6.96	40466	2	9628.270485	6550.701552	5635.174724	8585.707507	7231.177979	6876.303269	6157.974478	7068.403897	6911.931421	7242.516097	10647.57025	8844.017311	9152.434694	9061.314636	8006.026205
P50171	Hsd17b8	Estradiol 17-beta-dehydrogenase 8	113.905	14.67	26588	3	42637.77059	40519.6729	39736.49827	40361.89872	38956.25751	39121.853	38162.07937	36186.87393	40605.29729	41523.82453	42914.78107	37781.83873	41286.56314	40455.69602	37594.13677
P50247	Ahcy	Adenosylhomocysteinase	813.817	58.33	47688	23	1009228.742	1035375.182	958438.1733	995632.7754	1001827.203	846790.0813	905941.2702	885730.2359	879678.9882	847177.9136	989722.9711	993483.3789	986184.7593	1024472.911	1012521.622
P50396	Gdi1	Rab GDP dissociation inhibitor alpha	659.795	46.31	50522	16	273039.0868	291239.4355	265625.2801	275107.4025	276283.7808	127826.3122	138665.1277	248771.1175	240217.6469	238922.6341	248874.1158	275518.0204	269222.1165	263091.1694	262602.4657
P50399	Gdi2	Rab GDP dissociation inhibitor beta	1482.039	80	50537	37	1086323.916	1133474.233	994410.9197	1058344.342	1046434.936	767213.3519	855547.7401	957196.2185	937096.8261	905707.7207	996367.0618	1051231.755	1041277.545	1059553.056	1052613.783
P50408	Atp6v1f	V-type proton ATPase subunit F	178.96	57.98	13370	5	10747.93848	9334.911712	9017.439255	11069.74449	8454.139706	8434.931091	2148.453896	6223.886335	8735.095492	7350.561994	11418.64669	9779.881143	9474.550377	2217.26147	8541.304901
P50428	Arsa	Arylsulfatase A	16.653	8.89	53748	2	1177.976751	963.1616286	983.4723463	819.5440822	614.5203232	336.8241952	533.2008755	574.3375386	0.012083387	737.5985568	790.0857898	800.401921	668.3075853	680.290868	773.2942976
P50429	Arsb	Arylsulfatase B	484.17	37.83	59647	14	1522174.642	1280422.86	1175037.347	1294895.718	1302751.882	670399.8649	648891.4819	583370.5877	692618.734	898932.4343	1229972.169	972948.2624	1082473.429	1165131.121	1104767.705
P50431	Shmt1	Serine hydroxymethyltransferase, cytosolic	94.741	7.74	52601	2	19746.80176	20871.893	18324.66828	24120.94031	17169.52498	14464.2624	16311.99779	13647.06283	19177.52522	19627.31539	18737.23761	25969.00682	20292.09042	21703.10791	16239.44801
P50516	Atp6v1a	V-type proton ATPase catalytic subunit A	1366.171	59.48	68326	30	752596.1677	816061.0122	712038.6464	772261.155	712309.7409	720185.5339	752553.0692	792242.5978	811900.8545	785177.5052	772394.8725	793118.3617	771611.3261	784640.5448	780204.7784
P50518	Atp6v1e1	V-type proton ATPase subunit E 1	214.308	36.73	26157	7	115809.518	98654.51852	89228.17953	107446.3574	88555.20517	66333.76371	45585.4294	52322.21964	93774.42902	52501.62993	100251.2427	65899.07244	63694.55241	71097.6359	71079.64829
P50543	S100a11	Protein S100-A11	164.439	71.43	11083	4	58592.71095	49915.3732	43285.05027	50926.99176	47468.49691	36542.32537	33193.02252	31213.27986	37520.34216	35247.86195	42463.66653	40317.85469	36990.68348	38416.80755	35216.23725
P50544	Acadvl	Very long-chain specific acyl-CoA dehydrogenase, mitochondrial	264.315	15.09	70875	5	21895.24291	24494.54347	22153.72362	26088.90325	23531.05711	31382.93934	27511.80447	22720.39973	28228.54992	27646.45753	24567.24771	21602.97594	24953.90069	24792.13472	27998.09094
P50580	Pa2g4	Proliferation-associated protein 2G4	398.528	45.43	43699	14	359090.503	411904.8262	400449.6243	420092.793	406725.9286	395329.3674	447617.3308	499268.4067	444653.6665	381510.9459	381774.4837	426007.9234	398774.8241	388676.7873	397119.919
P50637	Tspo	Translocator protein	244.825	42.6	18841	5	269762.2577	212246.7365	220294.9842	242211.0757	206165.3426	294165.427	232751.9057	267193.4008	230703.2848	234550.4959	250478.2985	246872.0547	267862.6421	260684.5499	204310.8662
P51125	Cast	Calpastatin	88.948	7.23	84922	3	5700.222634	2771.230938	8753.947401	10560.45247	5681.806781	3506.331953	5522.249964	9316.431507	9125.730245	9869.179565	8792.09979	5143.586299	2781.782979	4994.429593	10359.8248
P51174	Acadl	Long-chain specific acyl-CoA dehydrogenase, mitochondrial	859.062	52.09	47908	20	502445.9459	510079.3809	459195.1614	480346.2102	511499.1623	571867.8317	521657.2503	544519.9976	535275.8299	576587.9071	479804.9303	509294.5909	543231.1802	553211.6874	503663.1698
P51175	Ppox	Protoporphyrinogen oxidase	80.073	15.3	50871	5	29871.88223	33018.97785	33385.68267	30484.19823	27652.91653	36697.45049	31429.69129	27549.0341	32032.77168	38753.69185	29813.01122	29224.04577	30894.08267	33328.39376	34663.45722
P51410	Rpl9	60S ribosomal protein L9	476.129	59.38	21881	11	477190.5042	480333.0119	484492.9493	489914.8628	462392.1572	479041.8555	557509.4642	546645.3882	565826.982	499941.6868	444965.3119	525617.4761	468140.5296	503339.5197	493647.8794
P51432	Plcb3	1-phosphatidylinositol 4,5-bisphosphate phosphodiesterase beta-3	233.802	8.35	139487	7	22575.27909	19789.7548	21447.4268	18672.55501	14824.96472	28736.46684	20128.02358	15495.33555	13627.319	16139.1031	25038.47629	19921.65015	17668.85386	18247.09829	22588.71539
P51569	Gla	Alpha-galactosidase A	333.553	26.25	47643	9	100788.9371	96267.15463	83366.352	84231.83628	83749.87287	55050.38864	62682.2005	51609.99173	66753.60153	70396.1888	82643.29212	90970.17286	88694.36153	88185.21793	83649.25801
P51660	Hsd17b4	Peroxisomal multifunctional enzyme type 2	224.389	12.11	79482	6	34072.95035	34544.99772	32712.19269	32810.33861	44294.89889	58237.33456	47536.52535	42946.64612	51947.78528	36566.20414	28295.20278	30526.74607	35933.82559	35195.71114	42911.63874
P51829	Adcy7	Adenylate cyclase type 7	123.929	4.37	122708	3	8428.751512	5817.237032	4911.363304	6376.685555	6029.580397	4984.158765	5940.469999	4074.369808	6832.008879	6892.384194	5680.163135	6965.626974	7179.797908	7017.571466	5922.45706
P51859	Hdgf	Hepatoma-derived growth factor	165.045	31.22	26269	7	96311.06308	80029.99774	85807.07231	80442.44631	76097.75396	49762.07458	68859.79633	58888.2181	57633.27256	63999.3089	71796.70874	71376.63869	70930.45299	66592.29056	73820.20124
P51863	Atp6v0d1	V-type proton ATPase subunit d 1	379.805	36.75	40301	11	182585.5446	172510.8853	166509.5728	170661.2249	169368.9127	177639.3742	176083.9678	190490.1638	182696.0369	184279.3379	164783.1599	174044.7579	184608.9643	179116.642	190606.708
P51881	Slc25a5	ADP/ATP translocase 2	804.91	58.72	32931	23	3204701.217	2914436.925	3243824.031	3074880.007	2890109.049	3940225.172	3656280.453	3861158.491	3639894.694	3581912.934	3113248.303	2959173.112	3147397.071	3376576.252	3145181.478
P51908	Apobec1	C->U-editing enzyme APOBEC-1	111.192	30.13	27522	5	41221.31081	38599.93477	45277.1545	48454.46222	37666.43222	45611.65612	44687.42058	38409.56976	36404.08492	41721.06985	40566.54981	38242.90928	38490.08824	42802.5231	41076.49037
P51912	Slc1a5	Neutral amino acid transporter B(0)	161.95	8.68	58483	3	15451.17809	15471.40747	12593.4024	13910.40786	12248.66388	14136.68712	18835.87153	14545.59361	19270.84673	15986.22565	14414.27285	14764.99038	16168.44586	11267.01469	15461.14891
P52019	Sqle	Squalene monooxygenase	95.834	4.9	63770	2	3600.05436	3233.082298	3289.023071	2954.140327	2589.336603	3346.571108	4101.709288	4062.3638	4431.345008	4038.405942	2131.488842	3824.094025	2771.056752	3370.416458	3126.261523
P52293	Kpna2	Importin subunit alpha-1	562.347	37.05	57928	10	51417.42158	62437.24759	63907.95296	62782.97268	55078.29462	75452.93689	66661.79264	73568.73158	77398.41893	70602.92477	59587.92263	61803.80081	61305.78833	55628.45912	59838.05749
P52431	Pold1	DNA polymerase delta catalytic subunit	339.844	14.03	123790	12	65145.69039	75264.59442	74041.8066	69693.88075	74682.89515	65466.11572	63257.47886	62275.58027	58527.2559	63939.21488	56791.33273	68279.02676	69342.05466	70097.88804	78445.81127
P52480	Pkm	Pyruvate kinase PKM	3430.515	82.86	57845	65	21604741.57	22806575.02	23079856.98	23863804.5	23084332.21	20763827.41	22420842.69	21755109.37	21464302.09	20832596.73	20982270.37	22805671.28	21909378.2	22584118.66	22167912.63
P52590	Nup107	Nuclear pore complex protein Nup107	66.466	2.7	107209	2	3970.07007	0.007562416	7326.351258	945.7402566	1152.426425	7858.438076	1074.04132	8826.913033	5714.001421	7648.782663	2919.253803	2083.350187	1681.649992	3233.995426	2045.215038

P52633	Stat6	Signal transducer and transcription activator 6	87.955	6.69	93497	4	21323.854	18977.09572	19746.09306	22479.27837	18461.20493	21738.89394	20481.77804	22375.08763	19049.84007	22856.22361	17212.75265	20429.28129	19683.75588	15376.13822	21162.80218
P52825	Cpt2	Carnitine O-palmitoyltransferase 2, mitochondrial	46.797	4.56	73981	2	12408.84689	14091.5275	12489.77283	14314.34568	12627.40037	12426.82124	13214.83378	11804.47054	8455.963938	13419.22083	13124.23747	8262.873732	12037.77866	13283.37225	14758.57668
P52875	Tmem165	Transmembrane protein 165	69.156	13.62	34791	2	9960.508518	12160.92596	9554.755535	8535.347831	11581.79917	14828.46446	13428.72787	13836.68454	13178.50425	12469.11188	10024.49866	10351.90756	10450.51847	9828.881561	9539.431325
P53042	Ppp5c	Serine/threonine-protein phosphatase 5	90.421	7.01	56917	3	25372.75137	23834.562	20423.39889	16853.25109	22381.18107	23290.74491	24189.62348	22713.71866	23934.75464	21451.5383	24047.25983	25525.8151	23283.32784	24415.37997	21324.28611
P53395	Dbt	Lipoamide acyltransferase component of branched-chain alpha-keto acid dehydrogenase complex, mitochondrial	118.196	6.43	53247	2	10116.58061	8488.310618	5546.879895	8068.922695	8174.572858	6870.990281	7808.367737	6519.340296	10402.97294	8600.196994	9160.552442	6827.297567	8296.852355	7036.347643	8746.129043
P53569	Cebpz	CCAAT/enhancer-binding protein zeta	47.08	3.14	120262	2	4815.777939	3408.595833	3133.401878	7336.047194	3158.825642	5534.984017	4375.199835	6805.714384	4879.082041	6056.536205	4368.698602	5233.380699	5334.235343	5089.640748	5214.034097
P53676	Ap3m1	AP-3 complex subunit mu-1	157.049	25.36	46981	6	37250.62327	37931.22438	30883.57346	34448.88238	34181.4841	27373.46243	36308.53327	32936.35489	30077.00301	31204.31608	35160.13951	31589.52901	35122.25255	30106.53139	33278.91963
P53811	Pitpnb	Phosphatidylinositol transfer protein beta isoform	114.69	34.69	31487	7	89439.07869	88773.67034	88995.73813	91973.81601	89147.3465	74110.78254	69762.11168	71274.13565	70237.54406	76374.12219	82452.26204	84070.48313	72463.07244	91352.82692	87217.75848
P53986	Slc16a1	Monocarboxylate transporter 1	98.015	6.49	53267	3	8955.279133	10618.9598	7967.785681	9482.408726	8788.707579	22321.12749	12522.49034	13996.07995	11924.81963	11405.67391	11231.52415	9977.673655	19293.69244	9443.363144	10221.46076
P54001	P4ha1	Prolyl 4-hydroxylase subunit alpha-1	376.958	26.03	60898	11	64288.56795	70455.62403	59467.14485	55151.9312	68341.48522	59154.88314	63156.99933	63695.80769	58437.94759	51109.99674	53881.9642	50311.50178	57113.6	59608.69392	56897.03048
P54071	Idh2	Isocitrate dehydrogenase [NADP], mitochondrial	817.561	48.67	50906	23	605879.8719	602116.279	564916.0769	574929.086	566185.2915	567399.5187	479365.1589	514710.6495	548026.5581	577065.9952	584121.7559	546511.4735	596883.781	583467.1325	580585.8237
P54116	Stom	Erythrocyte band 7 integral membrane protein	430.834	47.18	31375	11	349521.5968	177863.8656	162653.8528	349514.5106	158993.6396	149632.4491	150789.1189	136413.4443	149180.25	151518.618	177450.2862	148140.0954	161833.2318	315579.7739	158747.1939
P54276	Msh6	DNA mismatch repair protein Msh6	449.95	15.32	151084	15	92323.40462	81445.59878	92367.79319	89731.26107	112736.3638	135488.3939	116072.7917	90661.37514	93752.8783	115658.2772	111406.3822	73542.64025	79770.2346	105656.4673	103503.6911
P54311	Gnb1	Guanine nucleotide-binding protein G(I)/G(S)/G(T) subunit beta-1	489.887	49.71	37377	12	654701.8559	586807.1666	610086.8154	578892.9837	605237.7719	543759.4914	594791.0881	629708.0919	547445.7822	589434.1617	533140.2448	525431.4783	590178.658	615836.1073	560951.218
P54313	Gnb2	Guanine nucleotide-binding protein G(I)/G(S)/G(T) subunit beta-2	730.278	61.18	37331	17	783443.8723	726351.8862	735588.3213	718080.6226	726875.8376	642697.2432	691719.6946	732980.0589	675088.688	679624.4547	637549.7974	648451.6144	707337.3257	729906.9485	684640.834
P54645	Prkaa1	5'-AMP-activated protein kinase catalytic subunit alpha-1	53.041	6.8	63973	2	6152.480308	9085.613843	8561.206908	9100.808275	7588.373103	7990.889251	8524.774526	6757.408044	7307.603435	7599.70197	8409.658918	7396.733046	6015.013754	7851.370641	7423.990275
P54726	Rad23a	UV excision repair protein RAD23 homolog A	120.914	17.36	39706	4	54206.91842	15796.11396	48290.16263	32623.19536	29475.67687	44270.48684	27422.78432	29432.21538	42710.67037	28805.52691	50223.6147	46343.85459	45335.68867	38668.41238	45148.1367
P54728	Rad23b	UV excision repair protein RAD23 homolog B	113.113	13.46	43513	5	68181.09296	30303.91559	55569.64782	42914.69375	34799.41078	48213.84038	28612.53202	34202.72145	45719.66329	29242.69545	63843.05908	53726.04636	52004.028	42243.91821	48388.84394
P54729	Nub1	NEDD8 ultimate buster 1	69.033	8.79	70307	3	9845.290466	0.007562416	9106.775419	12919.00124	9411.219748	10053.49429	9390.771409	12015.76275	9755.744444	0.013320187	9342.388479	13061.29114	10641.74391	10817.10451	10218.12409
P54775	Psmc4	26S proteasome regulatory subunit 6B	495.645	55.02	47408	14	165639.2116	166665.879	200300.1355	179044.2373	158465.8645	135524.5253	157622.2952	172310.0864	183989.4306	143071.8878	163252.1996	164804.354	169244.5636	170219.0586	164633.3403
P54818	Galc	Galactocerebrosidase	55.234	6.29	77256	2	8603.677334	8848.722116	4550.06756	4808.81202	5195.280886	2915.334761	4616.51952	5831.071982	5925.642305	5279.652954	6853.251316	4914.172435	6474.646869	4799.581999	7759.465125
P54822	Adsl	Adenylosuccinate lyase	453.506	35.12	54866	11	149101.5299	154898.367	147267.2248	148124.1548	147064.6	110610.9861	129269.1984	133519.3881	121130.3228	124715.3116	122584.5425	143841.9842	121682.4289	144200.2144	137251.2253
P54823	Ddx6	Probable ATP-dependent RNA helicase DDX6	306.321	26.5	54192	8	73959.48478	76089.72204	83192.27296	86608.06006	80950.12726	74499.62767	91355.88833	80547.36322	80157.57574	74520.18527	77049.79851	76585.33863	74481.70409	73242.1115	75540.11328
P54921	Napa	Alpha-soluble NSF attachment protein	261.977	27.12	33193	6	52717.53629	55693.00965	52301.0464	54465.86387	49956.91307	48893.99039	52513.60253	48135.267	51794.56793	46818.52556	50998.72642	53520.73867	52193.9951	52590.83697	49614.47605
P55062	Tmbim6	Bax inhibitor 1	43.975	3.8	26464	2	10621.95935	6512.506172	11798.75608	10761.12768	7365.071461	14614.12142	13486.25633	14699.4024	6490.81711	12803.73915	8062.828005	10538.29597	7844.034615	13674.23941	12937.50294
P55096	Abcd3	ATP-binding cassette sub-family D member 3	43.503	5.61	75475	2	8240.879493	9862.968733	6415.1074	8436.36067	6160.143772	7161.929413	8451.731696	9319.953435	10993.70556	10677.66496	7855.013616	4114.698103	8381.618362	7373.756932	8700.360607
P55194	Sh3bp1	SH3 domain-binding protein 1	151.519	7.21	74172	3	10800.23329	15587.18238	9380.684817	9877.088318	11045.59704	11034.47915	8861.102938	9233.859742	11491.25563	8135.368225	13584.39015	10086.5543	10174.7392	11164.59899	10401.57858
P55264	Adk	Adenosine kinase	112.439	13.57	40149	4	39938.84159	36282.5686	40323.19599	49093.037	51696.48789	31813.90263	36222.29153	32061.75725	36128.3964	31565.45299	40717.22306	33157.50898	35369.0214	40189.16909	31358.52057
P55302	Lrpap1	Alpha-2-macroglobulin receptor-associated protein	230.143	22.5	42215	7	135002.1917	128852.2124	114861.0684	117639.7548	127526.8785	161477.3694	165117.0657	165127.3808	158234.4285	164743.7121	157079.2752	148582.3203	150781.7313	141124.6345	140216.2419
P56135	Atp5mf	ATP synthase subunit f, mitochondrial	50.03	34.09	10344	4	43182.30052	45446.53758	47877.32284	50747.33443	43750.62928	59527.58909	55852.58671	55039.89476	56450.33315	55276.42466	49688.41908	50044.89976	52154.33666	53427.23602	50937.12828
P56391	Cox6b1	Cytochrome c oxidase subunit 6B1	60.841	29.07	10071	2	27084.02449	25527.30855	7085.805646	25683.83623	24783.72323	19983.84928	23341.5272	17356.20319	18879.32082	22335.72565	18727.21579	21007.58998	20855.364	22083.22912	6199.090545
P56399	Usp5	Ubiquitin carboxyl-terminal hydrolase 5	937.149	46.39	95833	25	344270.2992	347272.8521	322634.116	334898.1926	298043.1751	267772.6086	313362.4236	277486.276	298477.9144	293076.3336	314717.8329	319801.9328	320555.0807	316721.6269	321088.1366
P56477	Irf5	Interferon regulatory factor 5	129.278	6.24	56005	3	28334.61255	31109.54162	28770.17737	28664.88054	25286.62636	24589.03268	30243.43204	27868.09924	33802.63745	32545.67222	29079.16959	29726.57094	30147.38632	31243.32998	30006.7737
P56480	Atp5f1b	ATP synthase subunit beta, mitochondrial	1842.881	74.86	56300	37	2131751.224	2215010.756	2049437.283	2108782.009	1936299.815	2137911.184	2183946.794	2415931.069	2469969.188	2319588.444	2191319.783	2251492.176	2282052.337	2198134.624	2098948.645
P56546	Ctbp2	C-terminal-binding protein 2	223.338	30.56	48957	9	143470.8831	149839.0201	156073.3894	164985.1555	160576.6673	161467.8603	153944.2034	170450.3557	164892.423	163726.3162	142241.9809	164969.8464	163142.9809	159984.0083	161945.7032
P56959	Fus	RNA-binding protein FUS	350.781	22.2	52673	10	133512.9371	140598.0204	138842.0846	132181.8695	132477.971	117272.497	124195.9143	115727.3233	121594.5786	111712.8977	147087.9539	138036.7163	129124.1177	131459.915	121680.5966
P57080	Usp25	Ubiquitin carboxyl-terminal hydrolase 25	42.54	3.03	121420	2	5786.033594	6111.011851	7169.444752	6907.047039	6366.820209	6586.371962	6110.624254	1432.001873	6617.680589	4922.26488	7618.715863	7052.690052	6106.420981	5530.585136	1503.083082
P57716	Ncstn	Nicastrin	174.822	12.01	78492	6	103288.032	95408.41208	102771.3481	89741.80783	108208.9793	126883.5371	135825.1886	126655.7911	128040.848	116091.5296	108435.4344	106343.0254	108106.8576	117422.5462	115364.7519
P57746	Atp6v1d	V-type proton ATPase subunit D	155.739	26.32	28369	4	41314.94296	44832.88157	37957.36865	39198.81935	40162.44058	39648.49543	41607.76362	47886.67339	42409.65206	39670.8067	41204.61891	41969.62698	42896.92962	36744.04017	42252.31218
P57759	Erp29	Endoplasmic reticulum resident protein 29	193.219	25.95	28823	6	156115.3857	178968.8102	166763.906	185387.2293	174709.137	191249.2081	184128.6529	180149.2835	169935.7266	197583.8603	173515.1519	170755.5055	185325.7599	184139.3768	187541.8273
P57776	Eef1d	Elongation factor 1-delta	485.996	49.82	31293	12	445591.7681	458086.5024	461952.2006	424508.7528	429518.6709	400890.0265	452925.6434	390111.1228	364372.8523	346627.5076	437947.2558	443378.9756	412051.2792	418521.4112	411581.8174
P57780	Actn4	Alpha-actinin-4	2225.24	66.01	104977	51	1387003.844	1360965.854	1217927.227	1277378.47	1246460.985	1071016.761	1170689.789	1228005.002	1154672.901	1220330.855	1317787.587	1257577.034	1251882.15	1256731.808	1320877.874
P57784	Snrpa1	U2 small nuclear ribonucleoprotein A'	114.005	21.57	28357	4	48095.28597	43912.19906	42915.70885	52048.76906	48847.22565	52833.74533	34807.7059	37066.06101	43681.34093	33853.58138	49380.14461	43557.3425	42690.85679	41469.62367	44779.59596
P58021	Tm9sf2	Transmembrane 9 superfamily member 2	163.342	11.18	75330	5	42519.09336	46105.03493	45028.32751	48473.79949	48513.7561	57454.11919	54870.57431	51618.11872	58867.34283	46648.36017	39051.46508	40746.76554	47570.41078	46853.13199	50729.27242

P58044	Idi1	Isopentenyl-diphosphate Delta-isomerase 1	119.261	24.67	26289	4	52495.40056	60054.03925	56430.33815	65043.95237	56428.18378	40246.45196	54079.41992	47429.61776	45961.1377	47811.75862	58537.78827	61095.46755	58156.86148	56144.69961	57708.94007
P58242	Smpdl3b	Acid sphingomyelinase-like phosphodiesterase 3b	120.72	14.69	51600	4	18755.86152	15614.63395	12454.74899	12872.10939	13922.99006	8642.674872	12434.09687	13568.49117	12424.95857	10409.72527	14009.63746	10768.97616	14587.27744	11169.58248	11610.13931
P58252	Eef2	Elongation factor 2	2023.466	55.94	95314	51	2684523.578	2927014.493	2724852.467	2742561.471	2821740.741	2744936.411	2937047.827	3062910.335	3061926.385	2551176.198	2646118.939	2731526.554	2707608.318	2748597.835	2750391.917
P58281	Opa1	Dynamin-like 120 kDa protein, mitochondrial	67	3.96	111339	3	2925.628294	2657.632829	6194.552206	2160.089253	6020.267874	5469.862509	3079.06096	6508.804854	7623.201866	6330.776809	6354.668928	3758.893527	2581.262578	7421.815232	6323.464756
P58321	Uchl4	Ubiquitin carboxyl-terminal hydrolase isozyme L4	94.532	11.59	26450	2	6495.129797	6574.583169	3196.972728	5394.650656	2839.580068	5266.931474	2227.902991	2865.180337	6023.827799	4022.197739	6701.616032	6136.798389	5630.767353	6103.482105	2172.154903
P58389	Ptpa	Serine/threonine-protein phosphatase 2A activator	250.352	36.53	36710	8	75035.01232	84937.02242	69307.55572	87612.07581	66054.07408	51445.81606	64206.67519	53242.60407	52912.30539	53516.11201	78837.60058	61955.32709	64321.3266	82680.48406	68945.65629
P58681	Tlr7	Toll-like receptor 7	224.646	8.76	121837	7	56157.11105	43940.63374	55181.06784	52139.1136	48085.90284	45164.62271	47124.24067	44369.47724	51040.31688	43919.73321	53735.44692	46962.46311	52646.09846	48519.87331	49932.12643
P58742	Aaas	Aladin	78.481	7.14	59431	3	18108.60112	10338.41025	17080.48934	15880.35061	17773.59705	19122.01721	19575.54513	20213.96391	17963.35327	12099.66484	17321.4417	18119.19843	16516.76958	17227.8751	19610.40383
P59017	Bcl2l13	Bcl-2-like protein 13	170.758	15.9	46719	4	29123.32479	31652.20545	13879.68055	34222.30338	13235.86677	7924.706847	9920.660671	2293.112038	11063.52035	10278.81447	28622.75045	24557.51872	24482.3802	11497.79311	18817.69209
P59325	Eif5	Eukaryotic translation initiation factor 5	111.347	7.46	48968	4	30063.67718	26600.97496	26273.75746	27897.4403	26372.51844	19869.05574	23778.49647	22692.18091	22550.54528	23526.14909	24206.97726	24477.61419	23234.71386	24943.36287	25066.01274
P59708	Sf3b6	Splicing factor 3B subunit 6	36.341	18.4	14585	2	10833.56949	6283.345579	10636.62514	5916.039362	9873.374253	9398.350995	11611.1004	10300.61946	9553.65584	9632.308346	10847.06415	10802.96995	7057.064726	10836.14958	9460.781517
P59729	Rin3	Ras and Rab interactor 3	28.734	3.16	107220	2	2304.43945	1704.675546	0.009509917	2126.788105	1714.918415	1882.965674	1413.940293	1247.389249	1353.892872	1928.005279	1679.000014	1753.844069	1595.276553	1595.134514	1655.392085
P59999	Arpc4	Actin-related protein 2/3 complex subunit 4	381.212	66.07	19667	9	1025049.038	1015125.694	1026093.02	996486.8619	1037499.542	976552.7313	1020983.31	1031669.653	1055626.345	1014966.82	991635.3801	1018662.355	1042732.957	1069544.322	1083827.066
P60122	Ruvbl1	RuvB-like 1	510.864	37.06	50214	12	228691.5711	258943.4737	252282.8088	249303.0534	250190.1835	259093.7732	259056.403	251744.4926	240022.8627	247297.8881	232234.6163	244991.2812	240663.0892	255271.6654	243038.4766
P60229	Eif3e	Eukaryotic translation initiation factor 3 subunit E	401.029	35.28	52221	11	182526.3808	207289.3763	201089.1353	204739.9822	196286.021	191581.0354	206508.3729	206673.0495	194423.2539	180935.3849	204212.9717	208197.531	180285.9777	184316.7777	207494.2907
P60335	Pcbp1	Poly(rC)-binding protein 1	636.305	63.48	37498	14	618598.6419	675767.9765	616316.9145	616532.4403	657522.4376	572041.9021	557495.4363	593211.5174	574596.1859	481587.002	515532.777	560657.8146	453741.6678	552196.6439	572379.5449
P60517	Gabarap	Gamma-aminobutyric acid receptor-associated protein	31.286	20.51	13918	2	14520.9297	12526.23034	12150.54932	14440.79571	15719.66646	16935.87113	15844.199	19203.549	20297.98295	0.013320187	14198.08162	15130.48979	13728.15744	12590.45306	16116.80011
P60670	Nploc4	Nuclear protein localization protein 4 homolog	85.95	4.11	68017	2	8899.169356	7362.366176	7585.725935	5965.264664	7193.035172	6686.955532	7546.598609	9501.401288	8734.743564	6905.228185	8573.18987	7792.787322	8495.687909	7533.31375	8139.704071
P60710	Actb	Actin, cytoplasmic 1	3245.62	90.93	41737	62	45860522.75	46775046.85	49577061.56	49137577.1	46860692.17	50881946.66	49784024.41	50750695.46	50961228.37	50130806.9	49520841.11	50411603.44	53038066.87	51572301.67	49771804.91
P60766	Cdc42	Cell division control protein 42 homolog	384.278	58.12	21259	9	1000275.725	827285.9966	825715.1024	924889.5965	821094.1976	741195.4277	745858.5663	738781.9466	759271.8078	646765.3651	726321.2597	713544.26	664239.7196	737464.3038	770341.9104
P60843	Eif4a1	Eukaryotic initiation factor 4A-I	1018.101	54.93	46154	24	858907.6659	1056380.426	1011596.177	1044845.945	1029389.082	1250689.366	1228409.619	1290619.486	1344792.233	1179764.919	1072385.462	1124799.004	1089265.483	1074704.76	1068442.261
P60867	Rps20	40S ribosomal protein S20	130.94	28.57	13373	4	338399.7889	341661.7817	329740.8393	345589.0582	356862.895	294477.7419	297999.8845	303148.1295	283098.4212	276844.114	275854.1029	271374.6236	242026.3704	258729.7732	274045.4009
P60892	Prps1	Ribose-phosphate pyrophosphokinase 1	338.338	27.04	34834	8	74650.46405	78576.72076	77230.62933	81117.72384	84690.26311	77119.57594	91183.61477	77283.15918	86336.37938	95882.99448	90117.44349	97172.66056	87831.14764	89880.45593	80228.49708
P60901	Psma6	Proteasome subunit alpha type-6	260.953	38.21	27399	8	266314.1892	263316.123	273623.7674	258692.466	290374.3757	261116.9863	330706.3988	325201.1126	263376.7871	273546.0624	243831.8583	269002.0101	264331.2154	264676.1654	273641.1581
P61021	Rab5b	Ras-related protein Rab-5B	133.178	21.4	23707	4	178610.7768	179401.0325	174891.0815	167226.3164	185258.2907	183095.3758	189889.9941	197277.0109	193498.2465	183513.2407	186724.0277	177655.5394	185938.4544	196684.9323	196392.1304
P61027	Rab10	Ras-related protein Rab-10	292.933	43	22541	9	158433.7315	155073.21	138192.2434	145562.3996	148610.795	149234.8728	140375.5519	151230.787	160485.4064	165091.4089	154612.4839	137956.2797	161400.4327	153605.6127	154045.0463
P61028	Rab8b	Ras-related protein Rab-8B	303.135	44.44	23603	9	111523.8605	100330.5162	102611.1916	103239.1503	117594.5976	128991.0664	117527.4296	124123.2663	124922.9958	132469.6584	122970.7063	111754.1619	125140.4846	121845.8247	114993.072
P61078	Ube2d3	Ubiquitin-conjugating enzyme E2 D3	106.186	40.82	16687	4	124230.0277	100973.503	128696.6955	122011.1889	100597.5615	112085.8816	88804.22434	96607.23656	91784.17437	77085.57335	105547.582	93309.22957	85363.778	85338.35852	100702.1826
P61087	Ube2k	Ubiquitin-conjugating enzyme E2 K	130.606	31	22407	4	39753.16086	42695.61434	44187.71737	37692.56417	39875.73513	39200.64417	44693.46207	48672.91907	46260.04444	35651.42698	38656.69561	43774.50601	37187.477	40959.53925	41732.48864
P61089	Ube2n	Ubiquitin-conjugating enzyme E2 N	157.983	45.39	17138	4	129087.5224	109186.7781	117337.3939	109415.2375	94863.66738	113460.2852	84016.369	74105.91151	102336.4511	72708.67981	128462.646	94844.78944	81943.63428	74272.12438	90516.38045
P61107	Rab14	Ras-related protein Rab-14	457.453	73.02	23927	13	486521.2744	419554.9357	423288.1856	444672.598	401962.281	370409.0527	403344.0255	315122.5595	361260.4135	377965.9114	450510.7121	393026.3497	453007.0152	423574.7277	428572.5277
P61161	Actr2	Actin-related protein 2	1171.859	58.38	44761	26	991801.6123	1000778.219	963013.3566	993676.7624	1022532.453	853158.0481	858351.0413	882853.7598	914788.6705	810012.3544	946264.9601	1018215.003	979151.419	970028.8298	934710.0862
P61164	Actr1a	Alpha-centractin	328.094	30.05	42614	8	111824.5166	112354.4093	117402.2325	116641.5719	111501.1634	101004.0463	118294.7884	111895.6879	110392.4452	109885.175	111032.6018	101884.5753	112444.8756	126415.9602	98508.89279
P61202	Cops2	COP9 signalosome complex subunit 2	226.863	26.19	51597	8	56795.83252	56101.65612	54266.07589	48874.38872	54309.40029	45686.33253	51895.78368	49772.98953	49065.04562	36107.0906	50319.16594	45657.86986	52592.60567	46004.2222	52990.77007
P61205	Arf3	ADP-ribosylation factor 3	661.188	58.56	20601	14	636176.2146	570414.5718	580235.4888	619963.9738	592545.2479	597417.6325	599179.2418	616596.1469	635487.2724	605385.2454	639038.1921	634327.2279	643690.7961	695162.3899	664837.9337
P61211	Arl1	ADP-ribosylation factor-like protein 1	127.795	19.89	20412	3	36839.83299	36353.16375	38370.13948	42340.4772	41242.80901	52880.48715	46882.73848	51912.56856	54252.17174	46558.0926	41890.2303	43696.92691	39442.65628	46749.82621	41784.02912
P61222	Abce1	ATP-binding cassette sub-family E member 1	780.715	45.41	67314	20	164881.418	172977.3502	187281.6861	192256.4593	197688.6216	163074.6681	223505.4918	187857.6572	222887.7018	158896.0568	181997.8658	200056.0503	164245.4308	172932.0824	176334.5295
P61255	Rpl26	60S ribosomal protein L26	205.138	39.31	17258	8	353641.872	382674.456	373068.6319	391458.6632	400130.9965	411119.8222	447553.8102	451017.8621	429713.3319	396901.0899	395163.2452	391012.8707	405494.1543	387755.702	350228.0931
P61290	Psme3	Proteasome activator complex subunit 3	136.902	16.93	29506	4	28537.86679	30644.79526	30693.80068	29535.03027	33729.20109	32946.96012	5862.48673	38124.02802	32176.86305	31250.42724	28395.21376	31690.29444	31942.74543	31957.15596	31345.61149
P61314	Rpl15	60S ribosomal protein L15	216.757	38.73	24146	8	287968.2072	262589.9496	238772.0501	291332.5706	283270.2005	390115.0173	418774.4071	453589.2548	438805.7906	391551.1434	284299.24	307888.9423	290553.2984	255638.2688	307671.6457
P61327	Magoh	Protein mago nashi homolog	95.197	44.52	17164	4	37607.25959	31067.74037	38256.57443	41296.70472	35919.68677	33285.75926	40102.86434	30743.10855	37040.81295	29276.30894	34326.80791	24617.83261	37810.64543	29570.50823	38824.68748
P61354	Rpl27	60S ribosomal protein L27	150.104	36.03	15798	5	327115.8234	374997.6058	332608.6881	326377.3936	356704.8187	431888.5008	434731.5499	439431.4448	444626.5997	388844.4547	353606.4747	377608.2748	375769.0784	388229.4646	404282.2822
P61514	Rpl37a	60S ribosomal protein L37a	113.284	28.26	10275	2	26256.90555	51847.34732	44647.97836	45160.40815	35163.65825	30506.51658	33262.26967	30706.6638	46434.9273	33529.48125	39222.58429	32576.8092	26296.1066	29280.56977	32425.55542
P61589	Rhoa	Transforming protein RhoA	508.556	64.77	21782	11	953510.1931	978185.532	764633.4351	924056.2734	903131.3928	792593.9095	722052.9803	632132.6503	798846.8333	640194.7833	681366.55	723544.306	749249.9272	784645.1595	805565.7522
P61620	Sec61a1	Protein transport protein Sec61 subunit alpha isoform 1	115.437	10.92	52265	3	30016.72949	36010.11745	29022.01662	29395.53091	31029.57156	36047.79771	33945.60336	36852.56865	35841.86003	32047.52387	25984.25461	28286.92639	31314.04326	31520.16432	31428.48447
P61750	Arf4	ADP-ribosylation factor 4	388.937	50.56	20397	8	298158.6953	254405.4193	248669.2875	268485.1137	260957.3409	264099.4191	262806.684	269922.6259	288867.2959	247019.5762	295582.9613	280329.6183	280463.1572	316439.4875	288690.6579
P61804	Dad1	Dolichyl-diphosphooligosaccharide--protein glycosyltransferase subunit DAD1	80.636	19.47	12497	3	18627.24894	22997.25511	21489.50819	22303.77555	20291.30743	26591.79009	22631.01606	26112.00765	28083.40428	24774.58194	21902.12426	22511.86416	22180.96379	21718.39172	22734.2378
P61924	Copz1	Coatomer subunit zeta-1	149.625	21.47	20198	3	34177.97186	47119.29478	47426.063	40743.33867	34198.94779	30127.50399	40104.553	26575.24098	24306.46832	33796.9173	34340.50292	30342.44193	34850.05206	35067.92526	37348.86685
P61965	Wdr5	WD repeat-containing protein 5	103.925	12.87	36588	3	8570.31646	3634.249772	5015.355107	7165.871083	8406.377506	3509.38531	5507.222612	4452.17127	5371.034119	3382.676851	10484.1937	10902.68022	8243.660045	5434.604471	5650.992801
P61971	Nutf2	Nuclear transport factor 2	149.849	71.65	14478	4	45794.31828	51349.69878	38876.49028	43458.74196	32570.45992	19537.85184	25481.55943	21950.65799	26090.51847	20364.97236	37970.54511	39007.58714	35515.90336	28051.13923	27410.44293
P61979	Hnrnpk	Heterogeneous nuclear ribonucleoprotein K	846.419	50.76	50976	21	1535868.134	1577296.197	1568674.437	1561613.423	1484281.518	1199676.771	1431659.18	1407769.592	1380911.12	1380433.108	1448731.693	1473029.612	1510532.308	1516303.942	1539952.448
P61982	Ywhag	14-3-3 protein gamma	642.297	72.47	28303	22	1551655.327	1554505.012	1470110.609	1527891.642	1605812.524	1526055.952	1582189.154	1544450.029	1535361.358	1453665.578	1554415.059	1582171.137	1684279.557	1619345.211	1568752.914

P62082	Rps7	40S ribosomal protein S7	294.7	52.06	22127	12	670101.1608	660123.8189	617347.1049	684662.1481	603917.9456	632192.8779	724614.4984	765313.8361	784936.9216	720664.4833	658809.5356	652098.3891	656267.6849	674165.7604	647968.4402
P62137	Ppp1ca	Serine/threonine-protein phosphatase PP1-alpha catalytic subunit	495.497	46.06	37540	12	224169.39	241595.6853	206799.0228	194517.3748	219491.2628	193991.3415	203079.8561	214961.137	211973.3826	175860.447	161803.4093	219849.6209	206550.5057	209242.6109	222558.0257
P62141	Ppp1cb	Serine/threonine-protein phosphatase PP1-beta catalytic subunit	490.999	33.94	37187	9	179531.5834	176341.8689	149148.4862	166920.185	156368.6839	142631.4629	148671.5655	138453.6421	150483.6971	134940.9394	143948.9647	162383.5746	149999.3355	154819.627	164650.953
P62192	Psmc1	26S proteasome regulatory subunit 4	440.189	38.86	49185	13	232528.9262	224853.0414	245887.3893	236960.949	238847.4577	239766.7885	247762.6862	244242.4146	260534.122	241741.5587	216468.7972	233772.0147	228427.5261	222511.3684	234164.8833
P62196	Psmc5	26S proteasome regulatory subunit 8	529.433	48.52	45626	15	199633.7526	187847.1618	186717.8241	195997.646	165279.3955	151892.341	207089.4304	198189.1087	200907.8639	193612.3933	190605.3475	191729.0791	135823.7659	195695.9505	182125.7123
P62242	Rps8	40S ribosomal protein S8	343.425	45.67	24205	8	125505.7842	136384.5448	135554.9626	139121.4235	130290.8164	189493.9344	234449.3517	221373.8579	214677.5962	190923.6603	122454.5606	140072.005	137175.8324	130764.335	170778.611
P62245	Rps15a	40S ribosomal protein S15a	335.051	71.54	14840	13	751961.4237	734066.0947	744840.8623	754946.6983	769275.1973	765855.2184	771208.0078	743225.8594	783824.38	692426.7527	707548.3161	709913.0637	669357.8375	741701.8031	678303.8645
P62259	Ywhae	14-3-3 protein epsilon	958.279	78.04	29174	26	2053060.469	2149441.102	1884839.327	1948934.662	2201661.396	1895656.258	1973548.158	2025739.253	1996666.596	1901983.961	1978362.307	2164183.478	2167877.609	2119649.88	2123375.689
P62267	Rps23	40S ribosomal protein S23	229.807	42.66	15808	5	308054.8122	345406.4479	334920.6251	324917.3539	356610.6541	412170.0029	390676.541	404716.1292	382559.4854	371444.1347	341623.4872	349219.7316	359420.6929	350811.4096	354795.5864
P62270	Rps18	40S ribosomal protein S18	304.374	54.61	17719	13	825561.5904	807873.5175	851079.1935	869663.3381	881663.6346	881699.7326	988468.1971	813785.2849	976841.1156	922521.2065	877939.067	933087.4139	878843.451	953231.4959	899719.5609
P62278	Rps13	40S ribosomal protein S13	301.8	50.33	17222	10	461662.8684	523446.2834	475483.6719	476041.7344	479718.1911	500540.5774	527755.964	524405.7725	518590.6543	485829.9611	504892.705	506303.7719	484166.938	465275.1428	481592.167
P62281	Rps11	40S ribosomal protein S11	261.042	61.39	18431	12	879060.4105	885825.4466	912834.0108	931056.1733	903588.9246	953937.6436	1012392.822	979491.8241	970621.9414	899170.7058	847226.7153	865174.2964	791656.5618	925467.9098	920844.4505
P62305	Snrpe	Small nuclear ribonucleoprotein E	145.962	39.13	10804	3	46586.18944	47119.79011	33965.43577	46327.97676	39286.96213	27776.33119	30087.1868	29031.44116	38284.45325	35493.83252	49867.00537	51522.26738	51787.68734	50802.00012	38945.2387
P62307	Snrpf	Small nuclear ribonucleoprotein F	87.878	33.72	9725	2	10935.30252	10825.76445	10583.09976	11895.91534	7560.558488	3173.108102	2671.799763	1845.593066	10429.58358	9672.020819	14361.92787	12902.28591	12659.41158	13080.63779	10523.44427
P62309	Snrpg	Small nuclear ribonucleoprotein G	56.483	26.32	8496	2	20061.51256	16845.67234	14112.01616	17186.26188	10102.18563	7167.401394	9118.992361	5969.919122	10281.82188	10090.33962	17502.48818	15211.19927	13405.39659	12546.36288	11582.80594
P62315	Snrpd1	Small nuclear ribonucleoprotein Sm D1	165.246	54.62	13282	5	148633.6983	152761.6669	137326.9646	138629.7331	143161.1142	136847.6325	150099.2714	148874.3135	151178.6006	135668.0351	149066.8424	156297.7068	149397.5514	159047.9631	150793.243
P62317	Snrpd2	Small nuclear ribonucleoprotein Sm D2	180.707	52.54	13527	7	259496.9922	264552.6384	244674.057	272814.0764	282329.7442	244995.1695	166500.9962	230348.251	224886.9948	240582.4094	224238.0993	228837.6561	218643.4672	236050.0287	248446.7798
P62320	Snrpd3	Small nuclear ribonucleoprotein Sm D3	137.598	37.3	13916	5	62061.48509	63663.89411	53410.76817	61343.48542	64540.52092	49016.26647	54549.37197	44544.73698	56770.95978	55513.85699	62221.66504	67048.46673	66931.54318	69389.96754	60298.30864
P62329	Tmsb4x	Thymosin beta-4	113.415	79.55	5053	5	154325.039	103576.2597	138677.6772	123933.5714	92713.45296	119525.2717	79054.99908	46460.34292	76927.52934	89923.03669	131697.0739	96150.26123	95027.01693	93259.8491	77237.52768
P62331	Arf6	ADP-ribosylation factor 6	304.954	65.71	20082	7	68416.76513	67540.32034	72576.02876	75803.56453	72391.88009	60357.69532	60945.08508	55923.82056	68420.66184	62270.2221	74337.23739	72806.70982	75068.62851	76749.31656	74632.68583
P62334	Psmc6	26S proteasome regulatory subunit 10B	454.088	41.39	44173	12	149368.31	152000.6913	143743.7719	146045.0289	149365.3853	154762.9918	158630.1282	167130.2937	155700.1435	147762.7781	149111.6449	156385.67	155245.4652	147259.6145	143041.6518
P62482	Kcnab2	Voltage-gated potassium channel subunit beta-2	87.277	15.8	41021	4	33806.04073	40744.4468	34765.35772	36118.21354	31258.25068	28124.42489	30962.86031	33840.12777	28491.80161	29637.34594	28923.84349	33287.1758	29836.6042	34648.18671	31301.9527
P62634	Cnbp	Cellular nucleic acid-binding protein	211.785	28.25	19463	4	75223.4283	47453.96948	70879.19796	54270.04246	58154.60761	54360.39156	50087.13355	46249.0689	49998.01903	28393.55018	55789.86715	68821.93101	50144.46184	50605.6014	48746.65129
P62700	Ypel5	Protein yippee-like 5	56.819	18.18	13842	2	6532.593557	6225.863657	5697.285754	7339.633691	5814.392192	8678.213777	10246.17034	6195.544275	6638.650522	6679.563839	8104.657846	6954.467965	7248.764164	6852.226233	7237.831097
P62702	Rps4x	40S ribosomal protein S4, X isoform	648.212	53.23	29598	17	1225627.231	1244866.075	1290529.893	1208005.675	1284462.644	1349253.969	1431170.28	1430294.711	1376513.541	1351027.677	1247539.243	1301904.801	1284696.582	1247443.186	1303024.307
P62717	Rpl18a	60S ribosomal protein L18a	295.801	50.57	20732	10	769774.6644	760279.182	747896.8933	819164.2334	817124.7999	799732.7184	809081.0742	809350.9815	822493.1514	790360.4965	703990.2532	765905.5102	672843.3068	768021.5196	708777.4138
P62748	Hpcal1	Hippocalcin-like protein 1	103.422	23.83	22338	4	48342.59841	49431.44666	50538.7731	51290.30962	54481.57813	52322.79497	50357.34286	53216.77545	51246.393	51969.40188	50744.11352	53440.58281	55203.72492	53953.55224	60106.06593
P62751	Rpl23a	60S ribosomal protein L23a	84.407	24.36	17695	3	37601.20089	35371.27104	44287.03419	39466.95032	35562.62647	49611.25687	64390.95147	47442.48347	57551.31095	55749.22137	50485.75502	27897.68138	41974.71864	39584.96573	46464.74881
P62774	Mtpn	Myotrophin	279.736	75.42	12861	5	135770.9016	123743.2334	96629.35839	99225.31595	102042.1526	85304.74875	78699.47416	80455.93543	79768.00735	65195.60821	83975.51388	88062.14539	82409.20404	79580.19475	83686.82488
P62804	Hist1h4b	Histone H4	791.118	58.25	11367	16	22229269.15	22419425.8	23545849.63	23641932.46	24826896.95	28224593.51	25272783.04	24773839.52	24664354.15	27296120.88	22794461.34	24474542.03	20954720.84	25166536.37	24789312.22
P62814	Atp6v1b2	V-type proton ATPase subunit B, brain isoform	941.299	57.93	56551	22	533283.7788	587463.9473	553900.0548	569842.0949	509078.3567	508842.1631	545589.1858	505400.9008	542635.0958	507066.4416	545156.9307	576853.3199	523111.3372	553752.0983	562888.906
P62821	Rab1A	Ras-related protein Rab-1A	545.308	71.71	22678	14	511701.4616	336341.9247	474831.7481	325181.4083	405211.7886	478446.7415	540744.4348	613809.9322	451445.9799	392539.8742	413382.4663	352982.8726	474173.1269	493996.5805	342602.8679
P62827	Ran	GTP-binding nuclear protein Ran	469.364	47.22	24423	12	1023432.332	1090310.747	1161477.27	1147755.779	1089697.516	1023344.946	1072256.644	1129321.866	1100272.333	1080565.331	1126908.589	1127337.979	1106438.199	1136915.748	1108854.848
P62830	Rpl23	60S ribosomal protein L23	191.96	50	14865	7	469075.5205	495293.8599	480945.4838	459242.8771	474210.0872	435420.1599	441547.5124	452447.3795	425135.6617	440579.741	458009.4292	433576.5206	438160.2739	422294.9824	425341.7353
P62835	Rap1a	Ras-related protein Rap-1A	541.167	66.3	20987	12	485201.0134	491143.2734	434488.5099	459457.9211	471983.1656	459317.9778	477310.8174	484866.4744	500639.8164	485000.5397	447755.2313	441444.9097	472749.1512	479505.3831	490556.2482
P62843	Rps15	40S ribosomal protein S15	111.328	28.28	17040	3	3620.999391	2567.751627	1253.381255	1850.343665	1927.037135	399.1711459	1180.700524	727.442151	1650.806459	669.5563781	2365.674892	3276.48064	1857.534569	1247.943823	1168.033994
P62849	Rps24	40S ribosomal protein S24	122.541	20.3	15423	3	38290.68708	44866.68557	36593.03547	39409.73639	34762.10283	44337.59892	50058.47583	57344.84644	60885.05094	44282.78822	26031.85322	44962.31171	40686.44949	36949.134	45603.43557
P62852	Rps25	40S ribosomal protein S25	129.882	24	13742	3	322585.0212	324080.9196	335990.8331	305364.1757	326385.6481	349421.4629	390537.0032	359290.6255	322215.2204	329644.0562	308481.374	310168.738	317018.1399	306699.5145	332408.2593
P62855	Rps26	40S ribosomal protein S26	110.364	31.3	13015	3	154537.7062	158849.1695	155097.1013	160045.7275	166842.1223	167500.1614	179910.3895	181063.1309	174800.4619	175542.1212	156897.9893	172213.2372	161208.881	150135.4576	167030.3056
P62869	Elob	Elongin-B	274.104	59.32	13170	7	116579.8073	125218.3204	108733.9998	115319.1263	129852.2713	105518.2978	98329.80639	111000.3024	105206.3159	93825.42521	102588.9131	106365.1883	102506.1861	93716.03238	102082.8117
P62889	Rpl30	60S ribosomal protein L30	316.786	66.96	12784	8	298043.3621	407051.6005	304722.0343	307769.4077	332215.0956	409192.9789	416422.5661	401641.0782	395130.002	353856.453	291162.2899	366128.144	292051.5086	352706.3466	395114.4324
P62897	Cycs	Cytochrome c, somatic	314.881	62.86	11605	10	414830.8679	346820.3172	379721.9604	353575.1452	390329.4071	383694.6199	374931.1992	371274.4983	375994.0738	398460.9637	390818.017	381800.9788	409884.0952	420217.992	384666.6982
P62900	Rpl31	60S ribosomal protein L31	61.731	16.8	14463	2	111707.9001	118137.6457	102698.5782	118577.2434	112320.2357	100437.0086	135294.1918	120111.0665	130167.1858	127417.2849	115843.7339	125758.3057	129775.6564	110957.748	124436.426
P62907	Rpl10a	60S ribosomal protein L10a	399.367	57.6	24831	13	600349.6028	630064.3054	648112.6008	613283.962	610496.5775	576241.0544	645298.9156	643323.1675	620871.3527	535441.8497	608746.3786	625285.7917	563351.0625	615442.2319	614167.7881
P62908	Rps3	40S ribosomal protein S3	525.947	70.78	26674	19	1251761.969	1331670.75	1293816.825	1321173.792	1378476.936	1472822.839	1461427.301	1545967.531	1528356.764	1379442.299	1243547.189	1381488.539	1278645.186	1350506.469	1381403.925
P62911	Rpl32	60S ribosomal protein L32	102.069	28.89	15860	4	30510.47414	35270.97072	18784.18393	21457.00984	35047.32455	30801.88534	37666.93951	36782.21341	19595.08176	29085.60382	21974.02305	25603.40779	24700.27354	28539.72939	34168.55048
P62914	Rpl11	60S ribosomal protein L11	448.019	71.35	20252	15	588247.9397	596173.7932	576999.7204	575417.0621	627535.6207	579247.1288	604507.2627	608192.9553	615952.5442	575086.2425	589118.5189	566754.3697	552563.6901	567123.2868	543238.3866
P62915	Gtf2b	Transcription initiation factor IIB	33.448	10.76	34819	2	4901.303569	2065.197417	2418.874848	6706.090367	1580.561135	6428.22569	5188.246893	1916.827313	10126.35177	6296.387017	6705.378217	6198.233645	1941.753874	6494.012382	1710.97054
P62918	Rpl8	60S ribosomal protein L8	246.047	45.14	28025	11	456042.6032	486462.4104	471160.3113	485367.5362	488818.0421	574331.1063	634083.1424	622327.103	567619.7699	584958.7388	473733.6805	496514.7578	480468.7141	488184.5251	505766.3758
P62944	Ap2b1	AP-2 complex subunit beta	860.326	35.22	104553	27	293330.2313	304575.4833	299711.1827	310052.022	275897.137	272027.6227	301169.318	309933.227	302806.4494	268543.16	279078.5934	296372.7097	282129.4399	282931.6479	299382.6286
P62959	Hint1	Histidine triad nucleotide-binding protein 1	96.715	58.73	13777	5	192686.7111	183735.673	185757.7408	159238.2118	168914.6211	160828.5017	162717.3867	134907.3853	133290.3425	127484.1123	177085.7512	164819.0628	154111.9714	164370.1423	159392.0366
P62960	Ybx1	Nuclease-sensitive element-binding protein 1	198.426	18.32	35730	3	48110.5047	44362.31782	42780.27812	40718.42995	44291.24125	40114.29245	104576.2017	97394.63481	42194.00892	33933.3693	42066.61589	44393.27756	49821.87638	37190.70801	37320.35758
P62962	Pfn1	Profilin-1	700.956	91.43	14957	16	2983507.827	2788240.172	2985663.095	3181878.172	2773412.049	2436287.41	2755563.192	2572910.92	2778285.601	2704771.592	2875136.556	3037521.744	3003556.333	2964367.297	2729022.107
P62996	Tra2b	Transformer-2 protein homolog beta	180.989	27.78	33666	6	68422.90609	74519.88554	79378.14463	62234.56819	71444.42254	57258.40985	78422.41145	62638.78494	68850.4377	73056.44991	76860.67543	59131.68065	56556.5715	77085.35231	75549.12166
P63001	Rac1	Ras-related C3 botulinum toxin substrate 1	404.337	54.17	21450	9	557827.4781	533716.5279	459888.6245	511507.1992	545653.493	441682.0735	409925.2436	406541.8522	411973.2283	336990.5922	355277.0726	403118.4165	355149.3937	427018.8779	497424.2098
P63004	Pafah1b1	Platelet-activating factor acetylhydrolase IB subunit alpha	323.705	30.49	46670	12	193928.1638	186298.2581	191948.2977	201481.0122	194290.4443	185758.9943	202670.9858	190444.9915	187686.657	169683.0904	187982.6488	192009.0721	183446.8011	197571.1237	195103.8747

P63017	Hspa8	Heat shock cognate 71 kDa protein	2230.44	77.24	70871	50	7254732.22	6641433.994	7507439.867	6938701.48	6574808.099	7183537.038	7948781.94	8114026.064	7127088.619	7552524.679	7255460.024	6756531.979	7262750.674	7714288.799	6610381.462
P63024	Vamp3	Vesicle-associated membrane protein 3	185.691	54.37	11480	3	25914.45349	28662.68232	25516.53504	24181.96025	21051.11345	17188.07849	18887.24736	15102.97125	19587.1611	16433.43594	26133.96028	15206.47143	24245.39595	23548.0445	23195.051
P63028	Tpt1	Translationally-controlled tumor protein	244.371	58.14	19462	7	163610.7453	160094.6238	152558.4478	157371.4703	133325.3538	142195.2108	151725.8124	132550.9117	151065.5605	154318.8276	173381.694	173422.2593	167758.0096	156159.231	146918.7891
P63036	Dnaja1	DnaJ homolog subfamily A member 1	114.421	14.36	44868	3	9523.455662	9957.663411	11067.20449	12885.50608	7344.760717	12452.40947	15262.56889	15357.17322	17614.24308	12779.5072	11151.09213	12345.9114	13412.75283	12351.47836	12908.02367
P63038	Hspd1	60 kDa heat shock protein, mitochondrial	2093.763	71.38	60955	46	2070205.336	2161308.408	2081075.181	2102953.883	2031791.779	2375451.726	2331564.998	2351947.835	2450561.528	2246277.332	2240080.836	2197287.033	2100863.821	2189367.462	2120341.994
P63073	Eif4e	Eukaryotic translation initiation factor 4E	71.205	15.21	25053	4	67519.99997	70726.27532	59693.71863	69419.75188	77233.52431	72081.84077	59021.53857	64906.51077	72837.36934	51771.15088	69842.13655	68855.43344	69238.61597	69965.79716	66146.41676
P63081	Atp6v0c	V-type proton ATPase 16 kDa proteolipid subunit c	144.756	31.61	15808	2	6432.16699	10655.03347	4981.106376	8374.402436	5357.715884	1005.507099	2135.049072	1788.902697	5312.990423	1289.469951	8664.397056	7326.98839	7402.821485	5575.846109	1733.940361
P63085	Mapk1	Mitogen-activated protein kinase 1	445.944	63.69	41276	15	250293.1873	243151.123	201776.7023	219707.1683	236194.084	192271.2251	187334.6626	196261.4459	206277.8782	189775.0606	237893.9216	236277.7322	232088.1323	245771.0512	226771.3585
P63087	Ppp1cc	Serine/threonine-protein phosphatase PP1-gamma catalytic subunit	538.526	44.58	36984	11	226765.6215	228326.943	198744.8265	214954.7391	202651.6913	174058.0617	189051.1499	188517.662	193034.7278	156147.676	179672.2249	210768.9853	189353.7905	195029.1462	205375.6891
P63094	Gnas	Guanine nucleotide-binding protein G(s) subunit alpha isoforms short	242.821	27.41	45664	7	37262.35403	41092.20071	38850.84679	36840.17658	35533.31573	30040.85224	37032.56444	35931.72943	24406.07168	34026.02119	27367.92117	38266.34921	38070.74823	36430.78001	35783.39236
P63101	Ywhaz	14-3-3 protein zeta/delta	843.291	74.69	27771	26	2635142.4	2617051.28	2691206.227	2734494.128	2826770.615	2524523.985	2693128.715	2607072.92	2628293.843	2590161.934	2711388.158	2745507.91	2920914.801	2930596.817	2669797.49
P63158	Hmgb1	High mobility group protein B1	259.891	35.81	24894	9	410421.6838	361048.3066	346434.0539	366481.3825	377842.6038	281231.1995	282799.7279	260918.2383	275377.4028	272192.5183	364402.8403	316967.7665	315915.2386	336998.6857	263188.1193
P63174	Rpl38	60S ribosomal protein L38	79.745	48.57	8218	3	128930.9341	131755.1062	140042.1601	133520.4929	133629.9424	129255.6908	144518.7983	146154.1006	140497.4061	128796.0441	130224.6996	128649.3509	129375.9911	128287.5589	140980.4469
P63213	Gng2	Guanine nucleotide-binding protein G(I)/G(S)/G(O) subunit gamma-2	99.828	49.3	7850	3	38632.1129	38399.98828	31224.01186	33132.1343	36966.9835	27150.92534	25445.12372	23309.28552	27286.59554	25706.66868	27979.18161	30571.99337	29572.63318	29276.98818	32256.22477
P63242	Eif5a	Eukaryotic translation initiation factor 5A-1	567.836	85.06	16832	15	725526.0622	701617.4024	703087.6078	680876.7567	673957.7425	731552.2256	696369.9818	731047.7442	730547.9538	646566.2549	815632.8622	826157.7925	758882.6324	755439.3681	676003.1475
P63245	Rack1	Receptor of activated protein C kinase 1	898.447	82.02	35077	20	863206.0248	936533.3193	905098.1114	896062.7635	901699.0186	834006.3988	924300.3888	959908.8355	891373.8701	716553.9802	739927.8218	874522.2993	783474.9515	838346.9556	841213.8738
P63259	Actg1	Actin, cytoplasmic 2	3229.127	90.93	41793	61	45683648.91	46558465.07	49444768.71	48934905.39	46675426.9	50742744.25	49683178.67	50682547.87	50831382.03	49981579.89	49403852.07	50235340.97	52915281.65	51403618.73	49621394.87
P63276	Rps17	40S ribosomal protein S17	395.75	59.26	15524	10	240727.9007	216026.7041	194152.0689	227061.6964	214567.3072	173922.0128	172996.2072	172724.6081	194175.5324	182088.6734	217477.4643	190480.767	187892.1187	189032.4084	161410.1007
P63280	Ube2i	SUMO-conjugating enzyme UBC9	104.874	34.18	18007	4	70289.03381	65907.2696	57579.15044	62805.32724	69653.89698	54973.10234	62407.34816	55485.94198	49986.20148	52990.94366	59092.77405	60737.80938	60725.92335	59192.90813	60199.82252
P63321	Rala	Ras-related protein Ral-A	134.332	17.96	23553	5	38485.42911	46972.86372	13379.35666	44371.50981	47295.41809	29329.03637	29129.08413	24215.95544	36988.99334	38165.15938	55356.33001	44590.56966	46216.96079	48246.39597	40411.22471
P63323	Rps12	40S ribosomal protein S12	225.662	45.45	14525	6	170966.6192	177269.1119	166223.4574	151511.7221	170665.0324	158201.7997	151608.6377	149227.027	137868.8466	131502.1733	127539.9791	139482.7462	126840.058	140438.6054	140306.3671
P63325	Rps10	40S ribosomal protein S10	282.041	42.42	18916	7	322339.317	300215.1759	288927.7542	321891.8802	218811.5777	242119.0954	353808.1464	242526.9771	253002.1844	351993.0654	298399.9489	299124.2929	242850.9953	345696.816	243712.4631
P63328	Ppp3ca	Serine/threonine-protein phosphatase 2B catalytic subunit alpha isoform	225.344	23.22	58644	9	88463.34483	81359.36455	90622.69481	94126.77845	83962.29718	70327.11235	79605.15447	69922.854	74618.06785	77171.40864	83281.40548	77713.20858	79519.11676	91364.288	84783.64075
P63330	Ppp2ca	Serine/threonine-protein phosphatase 2A catalytic subunit alpha isoform	466.987	58.25	35608	12	225674.5297	205217.6828	217273.8356	230094.6171	203527.5086	219970.9097	238447.1208	218796.2579	210372.1646	200391.4493	200652.3178	230119.9688	200822.6956	228281.8916	213660.4791
P67778	Phb	Prohibitin	743.077	79.04	29820	18	789840.8022	776765.3692	744246.6827	782035.2771	811978.2777	814216.5686	891990.2605	942081.8605	836796.6915	903823.5003	758999.1267	819240.1514	836938.4634	782363.9121	718747.1184
P67871	Csnk2b	Casein kinase II subunit beta	41.806	9.3	24942	2	28388.9475	32008.42359	29765.57805	26326.22848	28205.87356	28814.26204	31231.21735	34286.78253	31370.76823	25682.40929	24495.81159	29738.92264	27296.14803	30199.58649	30436.73876
P67984	Rpl22	60S ribosomal protein L22	150.378	39.06	14759	3	208956.4445	220124.641	202773.8742	204709.4241	209657.8994	222070.2241	241994.9856	228190.8384	234472.504	204674.5421	207465.335	221896.7563	221166.8905	218195.4382	211797.3032
P68033	Actc1	Actin, alpha cardiac muscle 1	1232.137	40.58	42019	30	30883565.57	31568155.12	34101114.26	34015998.46	31879220.93	34460454.32	33854861.1	35023828.51	35554584.5	34391247.8	35558471.21	35415555.86	37227043.15	36470978.2	34639316.05
P68037	Ube2l3	Ubiquitin-conjugating enzyme E2 L3	192.778	51.95	17862	4	75302.31886	72626.58939	66223.80806	70560.45833	67274.68934	57465.96912	60491.80347	55434.63189	62241.21174	58962.3668	56488.31257	69360.61462	65121.5648	56315.75533	66003.60359
P68101	Eif2s1	Eukaryotic translation initiation factor 2 subunit 1	535.04	54.6	36108	15	502383.9523	512552.2303	497302.2375	500336.6115	494055.8194	402204.9769	486557.1572	481065.0002	437575.9435	402386.7691	484280.2852	505987.6408	471109.713	516422.9708	460242.5394
P68181	Prkacb	cAMP-dependent protein kinase catalytic subunit beta	264.71	23.65	40708	8	106543.6371	107281.5938	105972.7762	109862.1179	116281.1278	115820.3782	117325.5814	123217.4451	121322.787	113892.3401	109404.2154	105648.3051	117765.1024	118648.0784	108901.9655
P68254	Ywhaq	14-3-3 protein theta	725.598	59.18	27778	20	1321709.38	1271993.005	1343918.874	1308244.003	1409688.82	1381496.73	1421678.65	1379952.992	1412103.852	1339772.438	1296012.17	1361749.293	1490394.215	1517661.267	1414328.169
P68368	Tuba4a	Tubulin alpha-4A chain	1509.436	79.02	49924	34	3926805.247	4120369.015	3993224.395	4099905.94	4004753.646	3698265.229	3817009.377	3202968.601	4126838.559	3869220.201	3682157.294	3935444.763	3958857.337	3951018.471	4126241.323
P68369	Tuba1a	Tubulin alpha-1A chain	1741.793	89.36	50136	39	5484605.441	6266720.53	6188243.593	6316326.615	6043157.046	5576771.023	6105751.04	5038005.02	5955168.291	5989859.599	5603677.873	6217242.911	6213655.264	6232254.944	6327352.119
P68372	Tubb4b	Tubulin beta-4B chain	2065.138	82.92	49831	45	6047252.029	6055366.862	6283495.535	6323338.915	5821995.459	5172406.406	5346120.332	4793537.105	5462352.093	5523727.224	6259144.724	6263497.317	5828558.042	6207572.838	5320552.69
P68373	Tuba1c	Tubulin alpha-1C chain	1691.767	89.76	49909	39	5700044.344	6511318.82	6402573.091	6572348.025	6247599.114	5829210.322	6376156.938	5350136.167	6255072.153	6260387.693	5889688.611	6459089.066	6573138.113	6439373.897	6621564.081
P68433	Hist1h3a	Histone H3.1	348.982	55.15	15404	12	17584898.72	18020336.88	19053631.2	19805848.51	19148594.13	20962015.35	18427241.05	18760645.03	18726408.46	21243335.88	19345845.17	18462912.59	19513431.21	19939114.2	19106214.24
P68510	Ywhah	14-3-3 protein eta	683.02	71.95	28212	23	1512462.828	1499901.467	1504783.616	1458079.88	1586088.73	1524223.24	1598724.946	1549197.498	1544060.623	1485207.567	1504442.665	1573218.75	1658681.354	1623532.327	1586296.829
P69897	Tubb5	Tubulin beta-5 chain	2252.156	83.11	49671	46	7106101.689	7055790.971	7301463.88	7471537.025	6840413.291	6127160.823	6236331.116	5651002.253	6384682.234	6503893.924	7338270.44	7331243.96	6915321.167	7251816.601	6314165.712
P70122	Sbds	Ribosome maturation protein SBDS	45.375	7.2	28781	2	8762.441493	8542.040636	9207.478883	8325.622437	8401.903073	6872.775986	8592.689528	8312.01807	7994.839298	7713.41887	7880.684716	7934.168494	7705.774592	7265.682176	8604.019093
P70158	Smpdl3a	Acid sphingomyelinase-like phosphodiesterase 3a	75.152	8.99	49858	2	5701.303328	6067.006607	4887.043449	5304.167401	3692.508221	1184.118648	3313.835335	2825.511382	2339.156048	3653.726004	4887.573057	4318.57363	4328.511145	4744.364068	4182.723258
P70168	Kpnb1	Importin subunit beta-1	1005.834	39.84	97184	26	517780.2298	551091.6922	496636.0868	506576.7038	515000.4148	410550.7927	466293.6569	489724.3118	461263.4418	423869.8862	492730.7652	520443.447	501287.1424	455407.6416	487858.893
P70188	Kifap3	Kinesin-associated protein 3	55.375	4.29	91291	2	1588.484663	1528.361378	1439.691569	1273.707223	985.8063403	918.6900292	1132.269129	1327.190547	1361.484683	1022.777631	1196.767309	1214.799028	1215.204248	1280.120485	1185.76595
P70195	Psmb7	Proteasome subunit beta type-7	53.644	29.96	29891	3	34182.8254	39597.35312	29922.61294	36592.55448	31263.76814	20434.37587	28674.21882	31408.36585	23535.24464	13472.71384	36484.7212	35340.06251	30664.46693	30242.90126	29996.57646
P70202	Lxn	Latexin	97.227	17.57	25492	3	83840.02098	82788.18469	87221.36822	85326.27911	86730.2275	86674.73954	83211.53868	44782.62768	84850.2678	81830.95654	83680.87004	83213.02374	85598.12783	88983.79635	89500.5416
P70206	Plxna1	Plexin-A1	150.334	3.59	211099	6	40420.60869	40463.77909	35348.45355	38054.07761	30809.81544	44962.23598	41206.71914	50147.71294	48836.07753	47880.54739	39116.31521	41842.5822	34210.84167	41258.89387	44121.86491
P70227	Itpr3	Inositol 1,4,5-trisphosphate receptor type 3	347.932	6.4	304275	15	89897.16218	100838.408	94136.53323	84513.08652	80648.37381	84948.18925	78090.94754	70345.4923	79040.42433	95828.48827	88859.97297	87423.19902	87231.89496	92512.17415	80087.59024
P70248	Myo1f	Unconventional myosin-If	463.556	18.84	125947	16	117017.5717	137866.2943	112632.8757	131400.8124	125448.668	124785.3131	120231.0292	126186.3627	128465.277	121345.9304	120125.8942	105301.6151	125603.3267	117820.6203	125146.3632
P70268	Pkn1	Serine/threonine-protein kinase N1	87.808	4.33	104411	4	53976.39593	54971.92196	54758.03818	49668.45955	62558.62832	55024.03162	59157.10629	56931.6474	59723.58975	56441.16845	61961.77772	61697.08483	61200.27939	62167.14443	59917.71873
P70288	Hdac2	Histone deacetylase 2	177.577	14.75	55302	5	46142.30461	53329.24427	46321.1152	47490.28117	49667.34764	49218.67502	47593.14619	44358.35079	44067.78576	46678.10415	48393.33155	45803.86674	47799.57519	49773.36787	44319.31373

P70290	Mpp1	55 kDa erythrocyte membrane protein	243.144	25.54	52227	6	52723.94872	37963.87889	33982.5346	38336.12115	40527.09308	32214.0161	38090.54157	43839.98341	31985.17824	33574.04393	43701.19399	35311.59585	48884.60951	40867.87423	38369.23321
P70296	Pebp1	Phosphatidylethanolamine-binding protein 1	253.167	62.03	20830	6	118241.2227	119339.3287	105050.8753	97339.04004	90461.84125	91566.42242	91110.90696	84599.80563	82638.07757	87317.44829	107795.0278	113260.748	102393.7453	98711.4478	89405.18432
P70303	Ctps2	CTP synthase 2	67.172	7.68	65514	3	11237.50723	8873.630823	10786.39327	12941.47274	10832.44533	9004.653211	12534.76781	11245.4725	10845.79433	8298.652937	8993.241202	10341.04576	10562.96175	7605.008171	10492.98138
P70315	Was	Wiskott-Aldrich syndrome protein homolog	167.146	14.04	54192	4	31204.47246	33508.76905	30477.68305	30708.87651	29905.37135	25117.48896	26853.5351	25062.49049	24036.03608	17197.45356	30410.95081	29499.85212	29654.40052	27775.93611	30642.83594
P70333	Hnrnph2	Heterogeneous nuclear ribonucleoprotein H2	330.103	25.17	49280	8	361028.5947	370196.5306	411542.2583	416449.3703	395860.6554	373533.074	352764.3299	360258.7548	335271.32	357056.548	383423.9356	351436.5982	327715.854	351698.7925	346406.8399
P70336	Rock2	Rho-associated protein kinase 2	112.459	4.68	160586	5	30565.751	28387.61835	30600.11372	19485.00016	25598.30297	17503.21046	19847.61443	29864.43874	22591.95504	22481.31862	26720.86019	21755.91287	27019.61572	27919.39587	27334.48813
P70340	Smad1	Mothers against decapentaplegic homolog 1	42.288	8.17	52157	2	7173.510427	7732.049196	6532.041345	7327.372476	7182.083405	4834.174773	4585.760953	5963.422041	4304.779807	4065.751886	5346.848679	1137.233016	5086.31883	5430.00278	5848.417637
P70388	Rad50	DNA repair protein RAD50	122.379	5.11	153488	5	17960.07875	20625.83468	15972.87758	17994.65913	18814.86384	19146.92207	17246.34199	18747.42201	19036.79907	18633.96245	17306.3969	19711.28856	22165.72149	18941.61206	17815.01615
P70398	Usp9x	Probable ubiquitin carboxyl-terminal hydrolase FAF-X	209.335	5.47	290711	10	28095.68481	27100.87899	22788.05175	29400.11646	28335.70747	26559.09008	28445.47877	27525.62516	28885.68982	22645.01706	24971.06539	31031.48798	27723.86468	19456.26862	25287.31383
P70452	Stx4	Syntaxin-4	115.23	17.45	34165	4	21587.2119	33300.64381	19280.371	28259.71953	22898.86769	28162.72818	29068.81431	77558.15075	20665.79218	60716.27578	21036.58284	18374.5107	22095.09276	19903.30171	23669.54254
P70460	Vasp	Vasodilator-stimulated phosphoprotein	226.347	28.8	39667	8	132098.3529	138125.0953	141309.4232	142985.4295	150407.3795	140121.9226	152372.9218	150786.4374	138841.668	134570.9046	132738.329	132681.1545	139804.5929	130791.0586	137888.94
P70665	Siae	Sialate O-acetylesterase	82.931	7.39	60775	3	21091.29139	20012.54735	18556.38099	19555.63829	19046.91543	15332.83643	12324.60295	12466.74657	13192.93182	13005.54578	19471.72899	17693.5142	17612.09749	17369.31475	18635.88491
P70670	Naca	Nascent polypeptide-associated complex subunit alpha, muscle-specific form	297.931	3.2	220499	6	152363.8971	169354.3481	172182.2195	179001.2064	159274.2821	181486.8814	196205.6829	213466.6315	210141.7586	181635.7604	170666.1396	194707.2905	179897.3995	179346.3743	177397.2596
P70677	Casp3	Caspase-3	121.249	19.13	31475	4	37393.46947	36288.73575	30132.49444	33998.89071	32775.74253	29055.3719	33330.27581	30609.44887	30830.7949	40399.69732	35507.10118	29643.96965	31208.32338	33737.38518	32041.60097
P70697	Urod	Uroporphyrinogen decarboxylase	260.78	23.16	40692	5	26983.95935	29225.1937	22695.02574	28896.96908	27783.53156	22321.28379	25924.81871	25877.87669	27830.62587	19320.66637	22412.05814	22082.89218	21657.60867	28411.17045	24993.6271
P70698	Ctps1	CTP synthase 1	167.691	10.15	66682	4	6960.98873	11653.9851	18044.75931	14901.09226	11135.50065	18625.99013	23276.83817	21945.02534	13788.97206	6313.33822	13036.51438	15180.69589	13595.01293	5737.002845	18084.37144
P70699	Gaa	Lysosomal alpha-glucosidase	332.592	16.68	106248	10	49665.26151	45476.47718	42613.9354	40474.72163	37530.26872	26548.60325	40694.12163	34588.84209	46934.5391	22486.90977	46567.90442	45357.42577	43216.59123	34481.85915	36134.80733
P80313	Cct7	T-complex protein 1 subunit eta	989.804	56.43	59652	27	905863.0614	954641.796	969161.4041	977095.7005	955955.257	968661.1097	1004941.753	996775.1204	1027885.112	896203.9272	887631.0775	968323.0972	892529.6774	938583.2476	912720.1433
P80314	Cct2	T-complex protein 1 subunit beta	1589.566	77.2	57477	38	792356.3498	804696.1534	783553.3018	844158.3522	749181.1587	765144.2222	833067.9852	805718.8098	850628.3028	737766.0582	774483.8521	808233.6015	790552.3262	781655.8214	772583.8217
P80315	Cct4	T-complex protein 1 subunit delta	953.883	60.48	58066	27	665821.887	726370.0359	707736.6641	683549.8032	703731.697	724996.3464	787840.8591	812525.4588	777499.307	711911.5221	644777.9938	753533.2855	677002.1652	701110.4275	683203.1812
P80316	Cct5	T-complex protein 1 subunit epsilon	1043.203	59.15	59624	28	511161.3862	624896.5135	565050.5091	558116.5087	590470.7157	502096.8594	570326.2912	602835.7644	558241.66	536232.909	575545.6231	599769.4911	586807.3172	576167.33	549697.0289
P80317	Cct6a	T-complex protein 1 subunit zeta	846.636	56.12	58004	24	775010.3649	787014.439	822974.3442	815326.0165	802754.4388	837942.2644	903776.7309	921703.3501	893413.1591	850277.308	784573.742	876302.5021	823655.2186	858617.3382	839775.1849
P80318	Cct3	T-complex protein 1 subunit gamma	682.785	46.61	60630	25	971476.7727	884346.1775	952331.9694	943855.2188	917278.245	819272.2314	995524.3128	974227.6718	950871.1138	848005.044	841023.5042	949664.4068	837871.0354	983414.3919	882196.7811
P80349		Fuctinin-3	52.309	84.62	4480	2	10451.15389	11071.57321	11282.35329	6233.705757	15600.92326	12998.75152	9351.34199	7060.751679	12720.37321	4959.346615	11793.45098	11474.44964	11847.74165	10150.33564	5072.793489
P81117	Nucb2	Nucleobindin-2	156.708	12.38	50304	4	21607.62966	16711.46593	21507.46053	30342.9082	19131.59295	31188.68398	23419.62666	29049.51252	32147.8448	31150.20282	25600.18285	25174.54223	23753.44282	28949.93122	21703.55377
P81155	Vdac2	Voltage-dependent anion-selective channel protein 2	562.666	50.51	31746	12	918795.6503	921362.6294	1014538.089	923867.2019	1020008.985	1318468.91	1192124.27	1306582.912	1302524.546	1168293.893	939823.7036	1077399.338	985408.7803	1017113.836	1118894.507
P81795	Eif2s3	Eukaryotic translation initiation factor 2 subunit 3, X-linked	382.992	37.29	51079	12	394804.3152	418080.6881	414129.9449	448677.6923	401271.3213	359588.8248	427277.0724	438516.167	399289.8529	350660.8336	405488.4768	423136.3329	388784.506	403322.1609	394924.9197
P82343	Renbp	N-acylglucosamine 2-epimerase	160.661	19.77	49771	5	45728.96416	51993.26414	44512.80439	44143.78214	41288.32496	31957.42499	44946.09024	40846.62933	38983.07843	32336.67849	41307.83035	41335.64846	42607.61526	42226.16866	44274.83646
P83732	Rpl24	60S ribosomal protein L24	113.715	24.2	17779	4	92218.46126	96912.19088	99131.23698	77664.45482	93150.93181	117512.9853	145749.6325	151328.6303	152350.2662	134489.385	79804.08033	87430.73457	103662.5016	90636.49974	118050.1616
P83741	Wnk1	Serine/threonine-protein kinase WNK1	164.924	3.32	250934	7	70002.15681	72943.45461	69934.14516	70483.2835	77278.01116	72225.8066	70827.75412	73550.10303	76865.94032	73712.28264	69967.74923	65784.43598	72552.353	73357.02494	75055.58768
P83868	Ptges3	Prostaglandin E synthase 3	136.366	26.25	18721	3	59109.06979	60019.57354	56303.57095	57447.97661	62246.44336	52066.34562	29496.86799	57659.47316	53094.54703	39416.49104	21392.14009	27222.91621	47470.30313	54083.69039	56829.73439
P83870	Phf5a	PHD finger-like domain-containing protein 5A	32.466	21.82	12405	2	4779.614969	4215.844914	3361.272006	3514.456607	3622.616788	0.014539799	3108.397044	3006.170767	0.012083387	2458.462342	3449.949698	2598.821978	2667.639501	2340.442445	0.010360417
P83882	Rpl36a	60S ribosomal protein L36a	35.662	16.04	12441	2	44463.14504	54483.88525	45922.18842	51473.08054	69145.66237	53452.98812	51399.51394	59185.89305	50571.07667	48277.30914	37467.6599	47481.10323	39068.17241	43409.54446	51977.72335
P83917	Cbx1	Chromobox protein homolog 1	135.822	18.92	21418	3	44223.61883	42407.08927	41268.62919	43362.36284	37914.54437	34755.6312	30585.12975	31116.7766	34411.09035	35818.58201	45787.98496	45229.2896	43173.224	41345.2728	35178.31295
P83940	Eloc	Elongin-C	181.167	37.5	12473	3	36856.99313	36969.23217	37868.7172	35794.98946	31729.21866	28774.41209	28831.90883	24899.92114	25001.46697	26899.41982	41030.07193	32836.64099	30213.96777	30856.96286	28656.10432
P83953	Kpna1	Importin subunit alpha-5	161.023	17.29	60137	5	14014.76328	15667.70982	15343.05952	15883.3679	13008.32524	14519.27286	14888.07054	16381.4606	17630.2294	10866.60349	13632.72198	16325.65443	14209.0664	13361.39297	14429.96499
P84082	Arf2	ADP-ribosylation factor 2	569.006	58.56	20746	13	508497.2053	458649.5394	460406.6488	488433.913	485709.4179	475932.9556	462411.5931	464632.1601	513768.2712	473090.481	505969.3161	505061.2021	513725.5308	562818.988	530385.7096
P84083	Arf5	ADP-ribosylation factor 5	418.744	67.78	20530	10	382333.6844	329739.6636	350190.6615	361351.7641	335473.6955	363085.2976	363080.7885	408613.4193	384406.4794	369091.5766	379300.9495	364960.6399	377247.2141	405477.1094	394467.2379
P84091	Ap2m1	AP-2 complex subunit mu	228.23	30.8	49655	11	138327.1531	150604.2458	157192.4404	139839.0532	135499.5226	123992.9377	150225.7568	155375.4626	135265.4204	138171.5509	130640.1202	152130.3085	148922.1701	148650.538	146892.7119
P84096	Rhog	Rho-related GTP-binding protein RhoG	411.779	51.83	21309	9	435890.1218	425306.1528	397003.002	411858.3525	425381.7408	398845.5271	383182.6966	362589.0803	357113.1051	319244.267	330603.4632	342139.8225	323787.7446	357981.925	417486.3017
P84099	Rpl19	60S ribosomal protein L19	89.065	13.27	23466	2	17734.06018	27637.72543	27272.64591	26351.27706	20428.66534	42118.13489	58441.51222	51013.8885	52894.69989	46072.38863	18930.97732	26463.62962	26465.96731	19957.5811	37545.03616
P84104	Srsf3	Serine/arginine-rich splicing factor 3	146.829	30.49	19330	5	154020.2368	161286.158	139703.2552	128821.8614	143488.0785	143047.1413	157153.7694	140932.6628	136481.6254	117345.9049	125016.4927	118094.5902	151760.0548	141837.0562	119307.3152
P84228	Hist1h3b	Histone H3.2	233.198	55.15	15388	11	17721016.27	18150117.13	19087614.93	19897208.55	19238935.07	20953063.48	18441610.33	18781999.36	18754794.56	21251054.34	19396452.4	18518751.06	19554189.65	19948436.79	19113626
P84817	Fis1	Mitochondrial fission 1 protein	121.036	15.79	16995	2	15769.71928	11940.09586	5596.686852	12383.15429	5223.885429	5411.617018	10051.08988	6009.42144	11972.54901	9714.693702	15977.99316	11246.87492	11396.9739	12891.75923	9701.201461
P85094	Isoc2a	Isochorismatase domain-containing protein 2A	180.081	40.78	22417	4	27075.18529	25277.34235	22819.17771	26295.16366	20415.55568	20148.08904	19906.60927	18422.88911	23701.5785	21179.98964	20791.04027	21083.70408	18855.36087	21590.21813	23597.50657
P85108	Tubb2a	Tubulin beta-2A chain	1611.259	58.2	49907	36	5060892.047	4874349.363	5073023.332	5153018.048	4718913.013	4223185.023	4366870.135	3826842.075	4375173.62	4499084.74	5257559.213	5079329.222	4736744.695	5082350.733	4262876.818
P85972	Vcl	Vinculin	499.007	20.36	116615	15	176284.0566	182554.4388	152920.5379	146873.3172	155626.9057	129233.2413	145171.5634	150345.976	135811.4324	153736.7088	185569.9939	171023.0903	185097.817	174008.8947	186562.9959
P97287	Mcl1	Induced myeloid leukemia cell differentiation protein Mcl-1 homolog	117.533	16.62	35217	3	10566.18891	13541.86183	10586.44725	11090.89191	10911.8978	13962.20225	12130.6848	13998.02842	12108.12461	11533.77415	11086.1897	11415.72981	11933.62495	11600.25492	12534.22631
P97310	Mcm2	DNA replication licensing factor MCM2	948.126	38.61	102078	25	347752.6057	374341.9746	408708.9496	410936.033	312378.4558	368598.3824	379856.4128	362557.0861	350286.4265	356473.2303	378734.8999	374694.4417	382802.6149	389733.0982	368469.3886
P97311	Mcm6	DNA replication licensing factor MCM6	844.462	41.9	92867	25	438588.0636	451255.5841	570340.3436	488462.4168	474851.9807	466893.7949	469218.9117	464710.868	487221.0219	439744.7518	410889.8782	411958.9649	487715.2799	481636.0477	397940.8369
P97315	Csrp1	Cysteine and glycine-rich protein 1	254.274	50.78	20583	6	94315.60092	80518.03068	82118.97416	84425.2732	94210.76371	80449.21908	75625.85252	81272.59223	70139.19133	75475.6889	75302.63892	70824.46896	80303.78887	87290.53472	81003.01075
P97333	Nrp1	Neuropilin-1	120.817	6.07	103000	4	5829.875848	5976.846542	5375.686498	4422.910046	4083.871348	4557.113442	5624.603379	5094.404567	5463.717141	5123.104993	5594.572833	5095.699421	5772.834273	3610.380816	6002.815041
P97363	Sptlc2	Serine palmitoyltransferase 2	102.438	6.79	62982	2	6990.823057	6075.646667	5877.184863	6208.751184	5162.655045	3613.565881	6556.312729	5410.441168	6689.085068	3980.739123	5929.441736	6685.400726	5751.167829	4748.797011	5209.30783

P97369	Ncf4	Neutrophil cytosol factor 4	586.392	54.87	38707	15	208428.429	192084.4438	204249.6612	210970.5182	184473.3316	225097.0323	199104.3513	216522.3665	205133.9197	233302.2612	204119.6011	204240.8203	205310.9487	242005.7827	202090.7118
P97370	Atp1b3	Sodium/potassium-transporting ATPase subunit beta-3	164.942	28.42	31776	6	139790.8649	142871.7816	123990.8459	136110.0916	142118.0124	143466.9634	136554.9463	131026.6755	137389.8007	123199.3012	122229.0999	145829.5869	141453.4699	138599.8107	153282.6232
P97371	Psme1	Proteasome activator complex subunit 1	446.188	56.22	28673	12	187026.7456	210760.7067	223906.1471	210592.2835	216636.979	169490.1656	188330.9992	204409.0174	195933.4719	191303.3922	191885.3883	202653.7705	195265.6268	203233.8636	182892.0723
P97372	Psme2	Proteasome activator complex subunit 2	458.769	53.56	27057	11	170408.5446	182831.5408	175827.8844	184516.0112	182170.9097	154440.8916	173991.7041	181554.4581	191524.1715	145543.0223	169498.1051	177011.8471	167007.7761	168746.7821	175222.173
P97377	Cdk2	Cyclin-dependent kinase 2	161.234	32.95	38978	8	32985.21082	35014.63886	31041.55934	33521.86034	28495.7655	27143.27196	24455.2299	30142.78387	38543.58753	33484.43571	33101.61964	21871.12447	30317.02381	37033.38525	44480.12293
P97384	Anxa11	Annexin A11	133.63	14.71	54079	6	43233.57938	47030.41749	43935.28612	43473.53954	41871.2044	37239.38153	42960.60996	26564.07288	35804.28069	43573.30511	44213.17568	48150.71714	45476.45064	48140.4897	46221.84884
P97429	Anxa4	Annexin A4	866.33	67.71	35916	20	652715.0314	657355.1882	547852.4701	560552.4272	583311.2254	836716.5593	780107.0592	847788.5913	871006.3065	797913.522	664489.2788	692383.6199	704343.7703	742459.2937	753817.1287
P97449	Anpep	Aminopeptidase N	345.655	17.39	109651	11	77306.6161	59492.78701	37624.52391	44360.2477	49213.85782	35294.84693	58828.95177	54257.07901	61457.79139	53264.36714	74943.87315	50300.61055	57090.51824	66642.65029	48787.44543
P97452	Bop1	Ribosome biogenesis protein BOP1	64.636	5.33	82546	2	4528.595544	4628.543446	8175.114387	7808.034603	6150.551243	4681.97128	7766.310378	5634.254984	6824.350247	5138.923115	5231.802483	5253.459878	5307.078088	5222.465062	5148.864848
P97461	Rps5	40S ribosomal protein S5	431.295	53.43	22889	11	622368.8701	641347.0062	630737.9436	643881.7719	655962.0418	572926.2127	644267.6205	673597.9308	610511.5401	514964.46	624738.0286	646878.7119	606210.7179	479330.3273	554077.7031
P97470	Ppp4c	Serine/threonine-protein phosphatase 4 catalytic subunit	80.328	14.33	35080	3	21056.23897	29446.32252	12001.36368	20684.53924	96902.2431	21329.06062	31287.39673	23771.12848	9928.910676	0.013320187	17317.13686	28065.19128	0.00981159	26089.01023	18085.08113
P97576	Grpel1	GrpE protein homolog 1, mitochondrial	38.348	9.68	24297	2	15317.13818	6526.93477	11470.38338	13722.05403	7506.468773	14338.25599	16482.92845	16483.98186	9319.238919	14228.63198	13521.54358	14541.13363	15116.40056	13437.33801	14354.93614
P97760	Polr2c	DNA-directed RNA polymerase II subunit RPB3	40.825	17.82	31443	2	2139.691291	6276.411789	4184.852256	4761.048041	4426.196978	2079.868072	3692.130469	3340.805092	3267.015161	3278.800638	4506.769672	4224.204822	3474.202203	3862.817234	3017.654622
P97789	Xrn1	5'-3' exoribonuclease 1	25.722	1.16	194307	2	11320.79682	13880.74029	5009.453633	8206.689272	10995.8748	12789.27751	4554.853895	10585.51111	15839.60891	8801.832988	14389.17707	3900.939495	7688.909058	14582.76433	15016.801
P97797	Sirpa	Tyrosine-protein phosphatase non-receptor type substrate 1	278.579	21.64	56425	7	139491.2782	151064.2524	142133.6382	151812.1307	148772.8058	144090.7499	114048.1482	129607.3229	127928.0133	148408.8205	130824.1901	130210.9864	152640.802	140299.7769	132123.161
P97807	Fh	Fumarate hydratase, mitochondrial	1056.408	54.83	54357	21	427968.3912	427167.6263	431302.155	420333.1126	404983.3533	437200.413	409594.3292	434219.5885	470556.0497	445963.0413	426946.3823	432193.6735	453917.57	448997.7185	419719.5517
P97808	Fxyd5	FXYD domain-containing ion transport regulator 5	240.414	32.02	19454	3	28947.13109	18792.93766	17953.18475	11283.26154	21352.70571	19382.14149	25804.8502	20462.49329	17979.1946	20800.96372	24006.01187	25695.49872	26052.70803	22823.57068	18958.69347
P97814	Pstpip1	Proline-serine-threonine phosphatase-interacting protein 1	160.97	14.46	47590	5	25494.46205	24847.55703	9593.192244	16342.88032	25094.15583	24558.33189	22120.22915	24208.7328	25046.48061	18848.02452	22292.16848	22314.37506	21556.84609	20644.45887	24805.97405
P97821	Ctsc	Dipeptidyl peptidase 1	238.605	18.18	52376	7	103944.0904	89325.583	87395.66599	88710.69148	89076.33451	68165.24046	61361.40153	55303.68352	56754.07929	52971.01	69747.92101	73140.43898	75319.83956	71641.75368	75995.77995
P97822	Anp32e	Acidic leucine-rich nuclear phosphoprotein 32 family member E	298.797	23.85	29622	6	164385.4687	165934.4422	185721.5841	162364.2008	168140.1597	153657.9525	184719.5274	170475.0456	169915.209	167385.0918	141553.8509	156644.1975	168111.0502	159344.8354	170879.4593
P97834	Gps1	COP9 signalosome complex subunit 1	189.463	17.62	53428	6	59382.04016	59341.44038	60292.95279	63236.24989	64113.39092	64470.72832	50946.68305	69836.86272	61811.00087	66976.55716	43631.78556	66593.96489	66677.20714	50414.68263	61896.64119
P97855	G3bp1	Ras GTPase-activating protein-binding protein 1	387.894	28.17	51829	9	85631.18211	90148.61583	101618.2706	99810.02883	107825.7741	99976.28602	104646.7486	111028.8805	98329.74505	92508.76469	102342.5236	100141.0721	107238.4141	96049.77189	94549.66993
P97874	Gak	Cyclin-G-associated kinase	63.416	2.91	143703	2	3468.025544	3437.266123	2730.450936	3149.463878	2857.193332	3500.985159	2715.283804	1745.389234	2554.09322	4064.884409	4502.194236	3609.037752	3711.922954	4248.156832	3758.388772
P97930	Dtymk	Thymidylate kinase	71.615	17.45	23915	2	13137.73105	4714.56498	12068.88053	3264.430862	11864.47502	368.8433053	10156.00326	1929.561938	10497.42799	11567.82808	2650.170948	13007.27311	10088.69033	1150.459553	8711.570578
P98078	Dab2	Disabled homolog 2	586.553	34.6	82312	19	478910.4334	441583.1939	437676.5005	436588.5269	447204.0713	431845.4339	425123.6467	437791.3268	423535.7246	411113.2231	463573.2395	454204.9982	437860.3924	442806.6152	449715.2786
P99026	Psmb4	Proteasome subunit beta type-4	360.035	40.91	29116	9	246471.5939	256824.4848	245045.115	243080.9915	247719.1545	206045.6786	237123.9077	224705.8065	217981.8709	209199.2231	222435.3953	243682.6122	237336.9209	235148.2532	257978.4046
P99027	Rplp2	60S acidic ribosomal protein P2	231.612	76.52	11651	5	54188.72176	23269.9804	49295.49404	39471.5588	9153.800236	42120.43945	18757.82764	17058.34252	38719.91133	11873.02519	60591.1705	20589.33489	8042.303501	16022.68353	8587.208644
P99029	Prdx5	Peroxiredoxin-5, mitochondrial	406.2	56.67	21897	11	400544.5862	344326.0812	320985.0963	318195.3384	313849.8449	292274.5842	256526.1266	228139.0006	263564.1038	248015.3134	351938.5804	276790.3521	274332.444	276841.8408	275984.5808
Q00422	Gabpa	GA-binding protein alpha chain	52.183	4.41	51344	2	11075.07207	13166.44177	13810.37584	14406.85461	14306.97434	14143.18095	12990.46972	11921.19664	14176.28388	13698.64592	11452.99686	13155.3588	13328.79996	14318.72678	12722.71337
Q00519	Xdh	Xanthine dehydrogenase/oxidase	800.625	23.52	146562	19	231655.8488	201641.2047	196695.6485	218043.4384	197116.525	166252.6902	195577.7259	175559.8139	196627.8075	185048.7119	207743.2262	210467.5631	209729.7945	209852.488	221556.4013
Q00612	G6pdx	Glucose-6-phosphate 1-dehydrogenase X	931.64	55.92	59263	28	621051.0112	668107.4309	620669.832	654996.2691	620686.2655	687881.0657	779595.7335	757070.2821	770692.4451	680039.565	639238.8377	714884.7921	647798.752	658546.0056	654207.5246
Q00PI9	Hnrnpul2	Heterogeneous nuclear ribonucleoprotein U-like protein 2	410.491	21.48	84940	12	231631.3343	237469.9187	209097.8362	237113.6662	236223.3451	225547.3445	205226.91	226079.5285	225292.8999	226994.1404	202728.6002	223869.7136	232121.6094	231437.9449	210979.6177
Q01205	Dlst	Dihydrolipoyllysine-residue succinyltransferase component of 2-oxoglutarate dehydrogenase complex, mitochondrial	264.324	19.16	48925	8	252092.9614	271291.0231	239979.5623	265062.146	262402.4776	345887.3902	269014.4842	306548.3713	290603.2678	335251.3754	285471.4426	275763.56	304474.375	326534.792	296498.4751
Q01320	Top2a	DNA topoisomerase 2-alpha	1042.27	28.01	172790	33	260425.9429	274994.7615	314367.4681	333114.7798	318084.9403	304681.6277	273373.0016	277584.9525	300146.4126	302401.823	276036.467	290840.7597	297485.2654	318697.9829	280899.0029
Q01405	Sec23a	Protein transport protein Sec23A	135.088	7.32	86162	4	13316.3361	13959.47166	9692.234468	21665.7946	9749.833759	7824.093252	8846.286313	7735.939876	5698.16578	8853.2248	14854.10128	10118.42757	11768.82335	16099.93828	9688.339004
Q01730	Rsu1	Ras suppressor protein 1	225.544	37.18	31550	7	85237.88924	79958.21529	75494.19448	74091.82389	77025.63795	61550.90396	65927.16339	68686.57919	71508.33574	60198.40689	77676.17214	70354.49296	70075.04422	68230.87563	73658.29383
Q01768	Nme2	Nucleoside diphosphate kinase B	450.316	79.61	17363	13	473147.0958	506391.2815	511481.8668	460087.0955	504037.2739	494635.4414	524920.0629	530553.5971	501363.7562	458873.2595	463022.4137	518483.9486	463521.1544	505834.0264	481886.9416
Q02053	Uba1	Ubiquitin-like modifier-activating enzyme 1	1435.103	44.33	117809	31	463493.6229	491570.9129	474039.1915	482139.4178	452457.9173	450857.1231	480763.5001	480625.8024	466577.3287	430682.1228	467438.4826	485597.0766	496652.1472	479926.1524	474529.1809
Q02105	C1qc	Complement C1q subcomponent subunit C	81.145	16.26	25992	3	13522.68858	12584.26054	13310.99819	12836.53468	11855.58095	12098.5671	10953.2339	9272.74502	12740.33799	14182.90378	13566.41444	11099.84439	11170.95656	14014.73147	11966.34338
Q02356	Ampd2	AMP deaminase 2	29.007	2.55	94787	2	8577.317606	12078.92669	4293.392795	10507.98803	18459.4319	9913.386095	10545.97425	9712.298637	7012.890354	8172.525753	9633.97669	8717.132168	15297.95422	13636.36323	14621.93056
Q02765	Ctss	Cathepsin S	37.975	6.97	36833	2	63886.39475	46208.39424	60687.0723	75345.0873	53029.28738	39658.71691	49321.80908	36012.22518	45752.29448	44646.36938	48970.28004	46875.51437	35016.78531	59682.52429	49552.614
Q02819	Nucb1	Nucleobindin-1	150.329	13.94	53409	4	26975.73096	23553.99259	19513.91912	24703.81668	18705.27094	17663.52993	19341.63571	18496.56492	21576.68471	21393.79695	6631.824878	25958.2673	21070.80199	20738.85206	24918.81712
Q03265	Atp5f1a	ATP synthase subunit alpha, mitochondrial	1612.934	62.39	59753	35	3218048.939	3203972.904	3209253.579	3193367.437	3138652.45	3601639.771	3379858.774	3436610.266	3675199.45	3484058.152	3227334.191	3202588.112	3343364.825	3428461.144	3284222.248
Q04207	Rela	Transcription factor p65	124.362	9.29	60212	4	36478.71332	70289.57607	66691.91473	36546.01801	61257.72264	53247.8788	66460.08649	34859.78926	35577.06469	66192.15799	67386.51776	35502.13132	66975.52854	34405.27699	62546.16161
Q04447	Ckb	Creatine kinase B-type	1299.978	64.04	42713	23	1414755.287	1444498.967	1465442.229	1411156.112	1331526.461	1211756.785	1225359.25	1213201.255	1298637.079	1226684.052	1424251.777	1469016.493	1409432.741	1341832.358	1417505.585
Q04692	Smarcad1	SWI/SNF-related matrix-associated actin-dependent regulator of chromatin subfamily A containing DEAD/H box 1	102	4.51	116451	3	8174.113528	8467.074409	9051.84257	10291.28746	7176.443527	6871.194347	8589.110817	6570.039395	10093.99469	7994.90106	7599.083119	6547.104105	5979.285486	8029.948886	6514.536118
Q04750	Top1	DNA topoisomerase 1	324.79	22.69	90876	14	170834.1917	181964.0259	167264.1277	166546.4075	194882.379	118440.4645	99680.41457	125733.1258	102520.759	123327.0018	151652.9942	150348.7834	158319.7896	160151.8016	116495.7986
Q04899	Cdk18	Cyclin-dependent kinase 18	112.94	10.64	51848	4	19159.42908	17729.94749	16135.02999	14285.00274	14844.19111	31412.72048	31805.96782	31341.00659	27089.45508	27137.09358	22596.83413	23095.38071	22696.45984	22896.51216	24083.16405
Q04931	Ssrp1	FACT complex subunit SSRP1	364.482	25.81	80914	12	77361.18961	84626.60038	82289.67718	85370.26372	78057.3742	75983.51598	80792.65783	83208.80206	76558.48458	69886.29205	76302.86535	72906.81408	64790.94365	74073.04479	77152.30808

Q05144	Rac2	Ras-related C3 botulinum toxin substrate 2	414.649	58.33	21441	9	829491.6727	788134.6445	777909.2799	800841.9824	834912.3332	859531.482	799008.3251	832625.631	778551.5766	660793.3335	587421.2391	668578.9414	623321.464	681945.4532	811889.6676
Q05816	Fabp5	Fatty acid-binding protein 5	300.599	63.7	15137	8	145671.2797	145778.0482	136373.3291	150871.4965	134352.4942	108265.7528	161616.4956	170531.1187	142420.5497	109814.9376	136215.4691	203057.9553	165926.7744	166553.8759	156572.7704
Q05921	Rnasel	2-5A-dependent ribonuclease	45.813	4.63	83275	2	5290.115139	4826.47217	3500.774174	5261.910395	4654.601796	2736.99278	2863.337933	2496.704954	3993.004682	4000.572882	2761.206768	3680.903459	2716.802043	5129.369195	3374.082378
Q06138	Cab39	Calcium-binding protein 39	276.569	24.63	39843	9	75388.41568	78650.80218	75264.13056	90058.77952	84552.868	93480.856	99137.11258	100744.2955	103868.0808	95001.65765	78308.54841	96964.60855	102121.248	101746.0723	104487.8789
Q06185	Atp5me	ATP synthase subunit e, mitochondrial	67.927	32.39	8236	2	79333.19027	74358.85902	71872.7133	65312.15212	75493.66846	80042.55543	27137.94207	74073.38272	75891.86225	73199.5953	70741.75859	63455.97161	74290.36827	68065.82467	69231.19973
Q07076	Anxa7	Annexin A7	327.888	20.52	49925	9	157729.0472	149810.782	143212.9949	158347.4592	138166.7004	134439.7254	147287.2892	145266.7345	149860.8468	129553.5365	139327.5833	147996.9561	140173.3517	155261.2854	140680.6372
Q07797	Lgals3bp	Galectin-3-binding protein	454.935	26.52	64491	9	107647.1177	104726.0267	113014.2329	111162.507	108731.2989	119262.2976	126940.1774	121436.6028	128592.0075	103369.5522	107990.3627	106219.83	95656.14591	101868.078	99366.5488
Q07803	Gfm1	Elongation factor G, mitochondrial	48.631	3.73	83457	2	13003.75078	9310.466204	13308.9488	14729.99615	6006.32804	15251.42809	9964.465339	16458.62537	14777.70275	18534.21423	7657.363345	12004.08288	13763.01189	13888.44087	14929.37851
Q07813	Bax	Apoptosis regulator BAX	202.847	29.17	21395	4	52246.19195	57867.74109	50881.58661	56277.78506	45596.94526	42930.53164	50870.86959	50804.7877	51259.49743	49037.52551	49870.90386	55291.2322	53372.62709	52201.31616	49033.49497
Q07984	Ssr4	Translocon-associated protein subunit delta	153.742	30.64	18980	4	51196.26889	66571.31776	43777.06012	60292.33021	54071.38901	61495.3692	73639.71777	65925.17939	67782.2663	75390.88593	73216.725	70703.61928	71921.507	66172.28723	68069.25828
Q08509	Eps8	Epidermal growth factor receptor kinase substrate 8	147.989	9.01	91737	4	17319.69762	10858.19209	14205.40236	13198.78181	8465.330645	11690.18957	11871.78749	10360.29693	14356.68734	10579.01066	14487.69242	11802.99357	9933.443993	9075.705388	15712.55737
Q08877	Dnm3	Dynamin-3	64.986	4.72	97914	4	103394.4643	95988.19224	86103.44889	89193.33915	84859.81241	84521.32255	88010.82253	93049.92619	96226.69198	91126.28913	93860.50169	93790.30965	96587.68771	90200.32607	96081.87194
Q09014	Ncf1	Neutrophil cytosol factor 1	176.654	15.9	44667	6	99899.20958	101272.5966	101000.6775	100527.365	107410.7866	95363.25113	94758.12757	89169.27887	97667.26732	96239.21624	102160.8917	103423.1421	97698.33029	96587.46881	96517.27881
Q0VBL3	Rbm15	RNA-binding protein 15	80.043	5.61	105722	3	11492.20074	7997.189381	7928.972712	5374.610648	12092.39265	7467.770023	12708.17743	10776.91118	6576.377277	4346.792445	11943.97739	5464.622433	12415.34582	6363.531444	9534.793744
Q0VGY8	Tanc1	Protein TANC1	231.505	6.68	200805	8	14813.69366	17542.28426	6913.583989	14499.96194	14299.44414	5833.170368	7852.753525	6674.049061	7972.864874	7624.164494	17251.85222	8318.287698	16674.43938	12775.88597	6123.08974
Q11011	Npepps	Puromycin-sensitive aminopeptidase	550.395	29.02	103325	16	127072.8847	141954.627	126712.2705	115722.5173	127971.1767	119190.6455	142479.843	140238.2476	128353.6869	131939.5283	128693.6347	134333.0174	133132.0542	135559.2571	136875.8053
Q11136	Pepd	Xaa-Pro dipeptidase	322.341	29.21	55029	11	153772.5748	158783.1345	168085.8036	157432.3848	174854.2359	103578.7685	131110.8992	123907.3473	131628.5264	147636.1165	160823.0495	158705.9331	132172.1765	148768.1371	170126.858
Q14C51	Ptcd3	Pentatricopeptide repeat domain-containing protein 3, mitochondrial	73.369	6.13	77796	4	15031.25247	15308.92802	14088.42562	15728.60667	16772.84951	27631.55841	18410.91115	18197.00143	19077.38566	20846.52376	16072.60398	16800.19185	14714.28603	17755.87337	18956.23676
Q1AAU6	Asap1	Arf-GAP with SH3 domain, ANK repeat and PH domain-containing protein 1	376.54	17.92	127088	13	93709.17193	102406.2481	104670.6022	104516.1165	96529.92402	83132.32097	91156.10855	84036.35271	79796.27643	88693.94974	85437.18646	96213.19943	99079.99842	96435.74184	111818.7232
Q1HFZ0	Nsun2	tRNA (cytosine(34)-C(5))-methyltransferase	234.526	14.93	85452	8	28204.4637	32671.44704	22502.23118	29052.6371	26115.25805	22718.49475	29612.35709	21558.38917	22948.22011	26687.27087	27494.83133	29142.99551	28022.72571	19695.18304	24674.1507
Q27W01	Rbm8a	RNA-binding protein 8A	50.009	15.52	19889	2	13980.6898	18892.54602	16616.28888	16275.6481	18151.95848	16094.38932	15876.90547	17239.26616	17944.7358	16664.22982	21047.69494	19159.5649	16542.30669	18660.23986	16478.52628
Q2PQA9	Kif5b	Kinesin-1 heavy chain	506.963	21.39	109531	16	118309.0077	95994.55979	105399.5564	94992.33804	101908.3779	90520.55284	94180.36716	88147.74272	90022.38031	99578.84684	104794.0588	98325.8181	91038.09532	102073.186	96787.57172
Q2TBE6	Pi4k2a	Phosphatidylinositol 4-kinase type 2-alpha	55.402	3.34	54258	2	7868.933968	6907.76808	6767.286985	7138.024128	5931.832482	5277.648251	6404.583138	6165.239864	7386.294982	7575.102049	7954.559912	7355.324845	9110.815744	9090.808959	7742.218916
Q32PX7	Fubp1	Far upstream element-binding protein 1	151.872	7.2	67197	4	78776.56351	79905.42207	87611.55062	82554.74407	83401.49998	78601.36325	86531.66056	82110.62014	80445.35973	84233.87164	86558.1414	83030.4948	74707.67973	82298.64652	83600.7868
Q3B7Z2	Osbp	Oxysterol-binding protein 1	104.934	9.19	88797	4	18911.88425	24536.28044	18414.16968	18842.5162	22957.76193	17601.94661	19579.93656	18538.04869	23024.47232	18793.87296	21993.7046	20162.9057	21703.53917	21956.1501	22662.80532
Q3B8Q2	Eif4a3	Eukaryotic initiation factor 4A-III	588.952	54.01	46841	19	323357.8689	344574.1587	355559.4664	366175.5079	376961.6617	398968.7374	380465.3902	395534.8571	403175.5318	355469.7141	338951.5283	376300.3847	365873.5403	377454.7599	326849.4192
Q3KRD0	Dars2	Aspartate--tRNA ligase, mitochondrial	59.564	4.75	73953	3	12632.40373	14043.87294	14645.1136	12392.5604	14576.72858	15683.12201	12758.418	6812.241389	6368.806561	14210.33338	12734.90335	5639.480542	6411.160975	8237.85124	14608.56691
Q3KRE0	Atad3	ATPase family AAA domain-containing protein 3	71.891	4.74	66759	2	8408.488526	8932.377559	6579.733011	10027.90024	8882.255404	9455.348754	9805.491824	7914.469857	8158.412107	8639.47989	8868.266204	9112.51567	8997.092055	7897.415308	9000.92795
Q3KRE8	Tubb2b	Tubulin beta-2B chain	1629.306	64.27	49953	36	5058065.347	4871017.06	5064686.862	5155844.215	4715508.273	4223185.023	4362681.137	3821735.894	4372411.261	4499084.74	5242847.008	5076347.46	4732101.066	5084064.676	4264887.568
Q3SWU3	Hnrnpdl	Heterogeneous nuclear ribonucleoprotein D-like	55.513	5.59	35294	2	85786.93131	73434.42933	86100.32964	93318.66665	64397.21572	57028.41567	68399.66131	58806.7746	72179.19934	79687.5653	80531.52505	90509.9333	83594.02224	91022.50752	80908.98997
Q3T1I4	Prrc1	Protein PRRC1	118.43	9.71	46295	2	8345.739027	8657.854306	6037.383559	7924.796509	6636.341972	6755.221271	7812.453538	8982.749231	8358.542417	7255.464184	7291.310538	4973.295308	8051.715907	7867.122538	8362.793627
Q3T1J9	Mob1a	MOB kinase activator 1A	118.443	33.8	25080	6	88582.79122	90892.01642	88954.95009	93022.64177	92340.15024	92013.18684	97459.85022	82818.84531	88293.28389	93580.36707	91563.48992	97031.52284	96547.09716	93883.4947	102933.9511
Q3TBD2	Arhgap45	Rho GTPase-activating protein 45	339.323	15.77	122902	11	92283.99224	107491.4281	99886.32443	91942.59757	96745.45629	74396.31513	82787.22042	55741.60722	79135.36955	80866.83475	89165.87376	84251.33649	89771.82131	86510.21649	93582.45251
Q3TBW2	Mrpl10	39S ribosomal protein L10, mitochondrial	110.241	23.28	29396	3	8456.726687	7529.065451	7364.631671	7766.932009	5238.74641	6461.145177	2697.636846	7383.189892	8433.732802	6650.853841	8288.291252	6447.39669	7413.77431	6319.601403	7777.560214
Q3TC93	Hs1bp3	HCLS1-binding protein 3	120.248	17.72	43691	4	24495.81168	17341.27336	27101.13692	21358.2233	20655.84007	19227.34715	21766.66455	21118.09784	18039.65533	18567.33155	22113.57414	23708.5187	20345.51575	20676.94376	21674.9888
Q3TCH7	Cul4a	Cullin-4A	110.431	6.46	87753	4	29605.91042	31193.04771	23307.73308	29581.47045	26794.12891	22433.72006	29224.86198	32251.313	31210.89294	28287.63471	26505.27243	27526.75673	29165.13331	27713.89307	27532.64146
Q3TCJ1	Abraxas2	BRISC complex subunit Abraxas 2	87.53	10.12	46943	3	12159.33494	13591.90517	13370.26637	13861.43761	10694.2567	11848.33439	12488.18473	13050.61071	11092.34073	12038.95476	12174.57977	12428.04739	12893.34197	12462.77238	12950.88601
Q3TCN2	Plbd2	Putative phospholipase B-like 2	263.875	17	66289	8	74606.02538	65535.72056	51452.45269	68707.47811	50048.32934	34415.58527	39110.59036	32721.77592	29517.70231	42002.0259	49164.9994	49652.96025	46173.24034	52573.36638	47049.26279
Q3TDN2	Faf2	FAS-associated factor 2	70.767	10.11	52471	3	17654.20509	5017.585759	10383.6756	10181.32363	8874.899823	10888.27143	11075.15736	11491.22204	5593.469274	11547.87943	12054.672	3553.687458	13612.69097	13582.46918	12208.1568
Q3TDQ1	Stt3b	Dolichyl-diphosphooligosaccharide--protein glycosyltransferase subunit STT3B	15.104	2.19	93246	2	4022.714749	4186.425718	3758.300361	4198.799225	3400.624458	4596.526767	5041.263006	0.01388636	4673.864625	5268.640889	4194.399568	3792.221066	5976.689682	4775.354574	4439.486137
Q3TFD2	Lpcat1	Lysophosphatidylcholine acyltransferase 1	74.567	6.74	59744	2	7897.71483	6467.021531	5402.759474	1411.937811	4589.715299	6762.159009	1856.09661	4541.435358	5410.488251	6272.598428	5921.047308	4540.828987	5139.512071	6050.394829	6328.361348
Q3THG9	Aarsd1	Alanyl-tRNA editing protein Aarsd1	47.018	6.55	44971	2	8258.975375	6317.697852	9489.918106	9010.749184	7638.537072	9745.744025	10854.11579	9861.18992	10440.66555	8346.727157	7361.991828	7859.538587	7470.437421	10065.71086	8666.575962
Q3THK7	Gmps	GMP synthase [glutamine-hydrolyzing]	511.269	31.17	76723	17	142910.9823	142874.5344	136821.3983	140441.0536	134268.7197	125141.9308	135342.5978	137402.9446	140719.1484	105372.3822	134581.4985	136576.349	121396.8331	122869.9747	130172.5328
Q3THS6	Mat2a	S-adenosylmethionine synthase isoform type-2	243.066	23.54	43689	6	80088.21977	78595.99735	86940.03633	84347.24545	80229.80725	82924.63448	89743.42459	88475.58578	83197.63505	83019.8698	74506.92836	83031.45486	75597.52718	80121.59856	80197.96494
Q3TIU4	Pde12	2',5'-phosphodiesterase 12	51.325	5.1	67514	2	11363.77017	11311.2791	10607.18125	8607.502085	11077.24598	11684.92885	10139.21786	12529.05988	12462.56056	555.8881635	11170.5011	9371.440613	13370.64517	10386.55146	10828.29306
Q3TIX9	Usp39	U4/U6.U5 tri-snRNP-associated protein 2	251.817	23.23	65146	9	27906.10686	35131.89033	23342.09003	30199.47444	29073.85281	33164.02478	32037.64371	31676.03237	35055.70581	27828.04498	25444.57312	34290.9038	34454.70875	25095.41608	26717.5305
Q3TKT4	Smarca4	Transcription activator BRG1	288.95	5.27	181427	7	29545.32551	39967.35187	49562.5135	53255.64045	30757.90791	56484.41271	28228.513	51205.50291	56870.4121	24022.21449	37285.07857	56516.5119	39172.79585	28985.40904	39817.59096
Q3TL44	Nlrx1	NLR family member X1	96.774	7.18	107831	4	15960.24532	14701.90426	15163.2674	15249.40848	13321.43805	13175.7163	8538.908836	12743.6753	14451.87875	13688.21288	12916.80979	15509.03943	14418.97556	14268.8505	13831.51754
Q3TLH4	Prrc2c	Protein PRRC2C	39.129	0.91	310892	2	10175.79377	13745.55549	10436.26664	11132.80729	10728.89272	10058.19886	10566.34386	9165.734998	9408.073684	4919.089214	9549.701495	10822.07376	9775.910328	9666.494366	10982.97537
Q3TRM8	Hk3	Hexokinase-3	1391.424	50.65	100101	33	645459.9301	585954.3076	584858.526	607566.4392	588202.564	472745.0464	850609.536	792837.4338	858856.4126	785490.2632	592950.6107	651295.2002	818830.899	856107.0961	794688.0607
Q3TUH1	Tamm41	Phosphatidate cytidylyltransferase, mitochondrial	48.228	10.09	37828	2	12584.13365	13590.31328	13184.01464	13940.82612	13102.55913	12176.36411	13290.37094	14046.54298	13131.70076	12292.06162	13266.22461	11952.13102	15441.32363	12625.12827	13778.94749
Q3TVI8	Pbxip1	Pre-B-cell leukemia transcription factor-interacting protein 1	176.098	12.52	81164	5	21073.4135	11167.6036	22388.22629	22197.48933	20891.19594	19954.45707	21763.50025	21517.44176	21219.10406	19433.78973	17272.0065	13881.87884	21760.67942	17975.96849	20494.1655

Q3TW96	Uap1l1	UDP-N-acetylhexosamine pyrophosphorylase-like protein 1	543.298	39.64	56614	16	257413.2615	243185.8647	234943.1861	244195.3356	233672.0381	214732.4817	247052.5501	245407.1747	226803.855	207101.4402	227281.6259	256900.8662	240882.6334	237365.9162	253662.1099
Q3TXS7	Psmd1	26S proteasome non-ATPase regulatory subunit 1	788.732	34.31	105730	24	379795.7941	429504.0498	400540.5011	408516.9893	404553.9254	409963.6174	420370.6203	436506.6164	443327.2973	395164.4305	402337.0156	402643.8575	394744.7723	409125.7813	385547.375
Q3TYS2	Cybc1	Cytochrome b-245 chaperone 1	41.296	11.23	20921	2	31331.49325	33598.19462	34809.29829	30156.77836	31400.05563	29157.0887	33235.12773	33496.3397	31823.12794	31951.76838	29797.22012	27586.24868	32326.21672	34178.87094	34713.80626
Q3U0V1	Khsrp	Far upstream element-binding protein 2	709.8	30.48	76775	19	438797.6707	409136.4073	425427.1182	400814.2894	425279.8723	363228.9217	400603.6275	367577.6439	349923.3207	366679.9568	438692.7084	426058.2088	418383.9935	412494.9994	423917.924
Q3U186	Rars2	Probable arginine--tRNA ligase, mitochondrial	75.549	4.84	65336	2	1616.415499	2784.702663	0.009509917	2292.547916	2100.316278	2298.565993	1249.974599	1316.937762	2923.97838	2573.885957	2626.293918	2786.479845	2090.001164	2465.068396	2605.704047
Q3U1J4	Ddb1	DNA damage-binding protein 1	492.801	19.56	126853	16	231301.3924	250783.4457	242277.3486	223165.0962	238724.8254	216614.1353	238512.6665	240186.6534	210626.7858	213005.733	223128.1439	205891.2159	205434.1234	211583.9438	223816.6505
Q3U1Y4	Dennd4b	DENN domain-containing protein 4B	42.609	2.54	164740	2	6035.975863	7837.621011	7026.524339	5502.927594	0.008265859	8836.979299	7734.244478	7126.319181	4666.444277	5459.773517	4943.593245	8243.773315	5697.559754	7734.008624	5387.576929
Q3U4I7	Pyroxd2	Pyridine nucleotide-disulfide oxidoreductase domain-containing protein 2	48.518	3.79	62940	2	7965.897782	7438.244166	7246.951198	7758.770873	7768.873136	6675.156048	4129.194646	6336.821458	6780.217972	7001.148516	8960.21165	5862.982344	7070.377828	8274.266989	7969.387887
Q3U7R1	Esyt1	Extended synaptotagmin-1	422.369	14.65	121554	11	81685.78232	89021.98226	76220.74742	85172.36965	81828.23425	96515.0795	92956.40493	94107.93486	103491.2846	87526.86826	90390.08841	83184.54803	89233.88125	79531.89177	91323.29114
Q3U821	Wdr75	WD repeat-containing protein 75	86.753	3.73	94037	2	4389.523175	3247.049399	2863.31309	4428.651432	2700.416852	2345.26354	3944.272788	1107.436061	3534.327592	3005.127208	3628.467108	4046.373157	3062.73752	2933.026141	3424.947155
Q3U9G9	Lbr	Lamin-B receptor	489.882	25.56	71440	14	191247.5302	184460.7726	196798.1654	196845.6612	199557.4992	248983.4365	268677.4943	294598.5479	270277.1985	242365.2897	187216.4526	216508.2174	207695.7341	194677.7107	184961.2548
Q3UCV8	Otulin	Ubiquitin thioesterase otulin	87.393	6.53	40320	3	19743.80943	18868.84352	13978.49216	17940.50491	16884.47167	14895.37748	14930.41037	18955.03003	17038.54673	14272.87165	17194.97208	17450.44269	15814.6436	17469.55313	16758.17206
Q3UDE2	Ttll12	Tubulin--tyrosine ligase-like protein 12	302.869	20.81	74043	10	81431.10321	87278.96644	81426.45246	80247.7713	85211.50817	81332.37377	89005.02905	96070.12606	85927.82195	88703.78004	78676.86527	82775.41802	84112.27043	97115.24604	89526.38048
Q3UEB3	Puf60	Poly(U)-binding-splicing factor PUF60	441.811	26.24	60249	11	94320.25703	97412.5808	88727.14953	90080.43249	82946.49751	87668.36035	98509.85949	100545.2276	88793.98924	89961.57199	95206.20121	96350.17085	97992.72705	94085.89819	88656.6753
Q3UFY7	Nt5c3b	7-methylguanosine phosphate-specific 5'-nucleotidase	19.393	6.67	34425	2	9155.407362	10028.27463	8906.717663	10578.58603	9694.345048	6937.114162	8745.46485	8223.724291	6190.731091	8556.039708	8519.145078	8171.109989	9473.705992	8983.759601	10695.27049
Q3UGP9	Lrrc58	Leucine-rich repeat-containing protein 58	61.816	12.84	40139	2	2417.496815	2770.421041	2570.721459	2734.999924	2411.743094	1434.352231	2319.872697	1710.772137	2345.47717	2031.327238	2555.167581	2539.262414	2274.623698	1664.506694	1994.513283
Q3UGR5	Hdhd2	Haloacid dehalogenase-like hydrolase domain-containing protein 2	59.041	21.62	28730	3	13541.11352	13768.08109	8660.844073	14270.29687	10106.18424	12662.78314	8465.377563	8925.014956	8880.849836	19056.83843	13296.46507	13166.80139	13795.07616	12869.03091	13353.13954
Q3UH60	Dip2b	Disco-interacting protein 2 homolog B	311.056	7.43	171126	8	34899.37211	37130.46287	27158.06091	27595.94485	32555.26107	33960.01337	37733.30947	34211.59136	34269.5667	32368.42716	32820.95645	33433.59611	35588.39139	37436.02674	28474.92135
Q3UH66	Wnk2	Serine/threonine-protein kinase WNK2	43.677	1.44	227527	3	24520.95342	24735.88284	21338.78313	22948.04787	25136.68987	21107.31777	22961.85792	23792.40933	25202.02099	23435.50355	21850.00357	22218.95109	25662.56732	25954.07299	27533.12063
Q3UHJ0	Aak1	AP2-associated protein kinase 1	165.006	7.92	103346	5	5565.452639	14104.42804	8569.982184	13249.4533	13041.18513	9428.351981	11070.62004	14967.8292	10972.67442	8906.347304	10781.06339	10028.21487	15762.77095	11014.99733	9776.22963
Q3UHQ6	Dop1b	Protein dopey-2	36.014	0.96	257483	2	1259.065548	1155.280451	1096.222593	1048.267372	618.1206834	776.9173158	791.402159	1193.153989	808.6372283	756.8257138	930.9113511	893.8811353	676.7477605	918.9215033	861.40156
Q3UIA2	Arhgap17	Rho GTPase-activating protein 17	328.307	10.05	92202	5	28417.19879	30669.02713	26476.09998	30485.09241	24234.21513	23000.9249	27044.23259	25587.532	19043.37848	23183.26583	24259.30314	27142.66298	27355.93495	25143.35972	29281.35546
Q3UJB9	Edc4	Enhancer of mRNA-decapping protein 4	224.499	7.89	152484	7	36933.04971	43652.26748	43546.29246	40812.20449	36849.6703	39749.42709	43541.93599	52246.15363	43058.70816	46951.92057	36421.99532	42327.99613	38449.21806	39699.77042	37995.68839
Q3UJD6	Usp19	Ubiquitin carboxyl-terminal hydrolase 19	105.179	4.19	150549	3	2796.665598	4158.834736	1158.381506	3390.131109	2644.76799	1352.557717	2678.635261	2830.126444	3264.781245	1709.964209	2444.299729	2842.744007	2251.202549	2531.94675	1926.966423
Q3UKJ7	Smu1	WD40 repeat-containing protein SMU1	144.893	16.57	57544	5	18228.20586	36916.15535	32265.46194	29143.46083	34644.34533	28736.07063	31072.44067	36897.12303	36072.77053	35273.79635	33364.03152	21107.98633	20818.31789	36901.64189	32094.33808
Q3ULJ0	Gpd1l	Glycerol-3-phosphate dehydrogenase 1-like protein	60.258	15.38	38226	3	25613.97837	27904.21173	24691.50691	26163.94809	20408.5731	22745.33522	24849.64634	25016.18295	26272.83261	23732.02423	24384.12123	26037.76425	26043.2423	27509.05929	25588.13371
Q3UM45	Ppp1r7	Protein phosphatase 1 regulatory subunit 7	447.807	57.34	41292	14	121238.8252	125984.3856	114240.3276	129939.2687	128826.9659	112986.3968	130122.5027	124540.8569	119232.651	113831.0672	121188.9873	129609.8899	133261.7831	129019.6404	130032.0352
Q3UMB9	Washc4	WASH complex subunit 4	241.169	8.95	136370	6	21967.68402	22315.09685	13712.12532	16420.82896	14014.0292	11971.85456	14007.93922	14334.07922	12723.80791	12969.16335	19551.51529	10693.367	10403.36029	14123.55857	14999.96921
Q3UND0	Skap2	Src kinase-associated phosphoprotein 2	185.729	23.46	40712	4	19623.77898	15305.91534	19497.01168	16172.48465	14081.72349	12922.45938	14380.52973	17573.85279	18684.84173	11401.04355	11229.54172	19264.00074	17725.89108	10345.93293	14303.63266
Q3UPL0	Sec31a	Protein transport protein Sec31A	396.156	14.47	133569	11	58404.15923	71331.94164	63571.08316	73461.20504	70650.90835	66713.77591	70857.2855	60291.33753	68155.1415	61752.9726	67899.69032	62666.46808	62033.18528	63735.79041	60258.05324
Q3UQ44	Iqgap2	Ras GTPase-activating-like protein IQGAP2	68.925	2.16	180528	4	135983.9637	148751.5447	127043.3963	135885.3354	134403.9161	131481.4869	126738.9467	124796.1437	139765.2254	129653.3846	166915.4132	158133.6293	157542.4664	159579.5607	150213.93
Q3UQ84	Tars2	Threonine--tRNA ligase, mitochondrial	44.498	4.98	81700	3	694.3082137	486.7147304	3295.521911	3742.656043	2678.378791	3501.120525	2777.837284	2432.156155	3228.027751	3402.464654	2630.710438	2951.044176	3428.657145	4495.912646	3271.821544
Q3URQ0	Tex10	Testis-expressed protein 10	104.337	5.71	105209	4	9075.489543	10935.53292	10659.98744	10134.23345	9915.97649	11284.43647	13472.75173	11713.7422	11298.48482	12273.82296	10373.31905	10065.03109	11818.18056	10223.0218	12073.55815
Q3USZ8		Deleted in autism protein 1 homolog	77.072	5.35	49463	2	9474.311534	8992.241641	10158.42845	9588.124038	8843.666242	15965.58487	14540.62616	13720.49737	12651.42238	14435.08655	9307.173318	12852.8293	12500.58524	11317.81533	12544.36527
Q3UVK0	Ermp1	Endoplasmic reticulum metallopeptidase 1	73.349	6.57	100148	3	4837.704207	1133.407147	4460.627975	4813.546006	3502.581762	3373.234847	6347.240714	4414.86987	6167.229266	3091.132325	3461.096002	1792.780526	5929.166019	5373.815007	3773.755239
Q3UVL4	Vps51	Vacuolar protein sorting-associated protein 51 homolog	179.751	7.67	86187	4	10200.63319	9200.805504	8030.836434	10137.42958	9320.024592	7559.184941	9698.540208	10572.42926	9419.717538	10158.41157	9131.610472	8989.934942	9349.649472	8406.551157	9029.056481
Q3UW53	Fam129a	Protein Niban	737.731	34.99	102649	25	465194.4013	463128.0927	453848.6477	461995.6389	483738.9033	386317.7742	408348.2941	368369.4996	412363.57	392319.5849	442957.923	426941.8977	437108.7854	441667.2019	432340.3626
Q3UYV9	Ncbp1	Nuclear cap-binding protein subunit 1	257.185	10.38	91927	7	21032.63145	21798.52714	22245.80101	24963.58361	19146.26279	19724.42836	21838.8136	26609.52988	26803.32954	23060.12403	21344.90157	20132.95141	22093.93989	21311.50434	23268.06086
Q3UZ39	Lrrfip1	Leucine-rich repeat flightless-interacting protein 1	164.582	14.81	79249	5	18336.75442	19075.68693	17941.33658	12466.43479	16034.76204	15365.1166	15387.9769	14728.81198	12290.00224	11259.85703	12844.392	14010.3354	14890.39058	13095.16391	12041.0135
Q3UZA1	Rcsd1	CapZ-interacting protein	48.015	7.28	44114	2	2597.617815	4558.486626	2633.378454	3005.35663	3189.924903	1345.332963	0.012348474	1686.902457	1596.967056	1524.058357	2533.608966	3048.640412	4941.111318	2645.436111	3180.660548
Q3V0K9	Pls1	Plastin-1	323.882	11.59	70408	9	532179.1136	596721.9776	560918.7543	617461.1429	582776.1268	625176.3778	648070.7529	677123.733	635444.3523	678851.2445	609378.6986	623367.9925	635535.5986	634926.717	646725.8533
Q3V1L4	Nt5c2	Cytosolic purine 5'-nucleotidase	176.015	15.71	64809	7	38323.44434	36836.04668	35195.2892	33877.30481	32020.95595	26584.19304	29251.41737	28820.87535	35455.0829	35368.00005	38974.49302	32372.38911	32084.66763	35863.1254	34580.50395
Q3V3R1	Mthfd1l	Monofunctional C1-tetrahydrofolate synthase, mitochondrial	942.314	35.31	105729	23	184715.8693	193130.2957	254998.4418	188052.5355	183000.7193	256533.9252	262342.8012	232896.8562	245723.9738	250216.6075	188094.9769	185705.6152	204575.4326	226870.6085	229611.1279
Q497V5	Srbd1	S1 RNA-binding domain-containing protein 1	103.445	7.84	110281	4	8289.051885	7611.313339	4870.257874	8086.473895	7595.085518	5187.80494	3146.391941	5914.535529	6380.791046	6874.327882	6709.370247	8260.321878	6433.861364	7404.732354	8088.799459

Q498C8	Rer1	Protein RER1	53.922	15.31	22988	2	20058.52229	22113.3127	19775.96846	22132.81471	21884.36782	23504.95977	24966.33633	20096.72138	20600.23528	21620.67803	20620.9671	18683.59532	22228.80265	19406.89116	25401.38979
Q4FZY0	Efhd2	EF-hand domain-containing protein D2	624.41	58.16	26759	16	979807.8787	955941.503	908318.1694	949774.7626	925989.9313	793042.4332	837175.2852	765157.8645	806591.4143	881720.8347	1053726.134	967617.7256	975420.032	997686.5805	980495.1709
Q4G009	Mcts1	Malignant T-cell-amplified sequence 1	100.927	20.33	20550	2	4323.489888	3933.720035	3915.759631	3970.723599	2972.871961	1809.77574	2688.13698	2386.314019	2918.989089	2325.486516	4490.884026	3595.547113	3535.841409	3445.370621	2915.982
Q4KLL4	Tm9sf4	Transmembrane 9 superfamily member 4	85.689	4.35	74675	2	25782.46194	28910.32118	24909.3601	25784.38821	24244.55365	35267.04683	33897.29922	41444.88147	36511.07122	35884.66346	33357.34696	28624.52295	27852.6418	32142.19761	27518.77404
Q4KM65	Nudt21	Cleavage and polyadenylation specificity factor subunit 5	267.979	46.26	26240	7	104451.6553	95041.38535	104783.5325	98500.77125	94630.90079	78933.6122	78627.83449	72762.79505	84260.20581	70316.93369	98960.7336	84890.40967	81482.98012	87370.32732	96514.60583
Q4KML4	Abracl	Costars family protein ABRACL	125.207	66.67	9030	3	7292.134183	6478.885071	2512.220917	5587.426658	4564.38974	1341.723458	2940.696493	2769.109364	3150.99048	1961.165485	4099.475418	2771.99627	2637.958263	2659.419674	2407.980675
Q4LDD4	Arap1	Arf-GAP with Rho-GAP domain, ANK repeat and PH domain-containing protein 1	92.416	3.17	162276	3	12614.59577	11862.19353	10728.91295	12125.68931	11299.20599	8948.316868	11218.93139	10493.41934	7543.832139	12521.92309	12363.73402	12575.77532	12072.85878	11945.41604	11409.40753
Q4QR75	Exosc9	Exosome complex component RRP45	79.099	11.67	48882	3	14558.78112	16593.1387	16890.44553	15589.14858	17018.18227	16222.95205	15987.66666	16845.39172	16549.63175	13849.59108	13804.31694	16058.49653	13351.78361	14850.96149	14956.21199
Q4QRB4	Tubb3	Tubulin beta-3 chain	1113.19	33.11	50419	24	2812583.795	3095735.254	2848693.503	2972300.699	2714826.815	2349292.416	2414528.962	2219572.391	2497747.444	2575871.185	3106095.614	3007330.246	3003442.676	3078923.967	2628976.122
Q4QRL3	Ccdc88b	Coiled-coil domain-containing protein 88B	95.491	2.7	166608	4	15073.94098	18624.49052	19260.29557	12667.17191	14528.46423	15039.87765	16639.94856	11338.36183	16508.77329	16124.08459	15988.69993	17056.951	18117.94506	20750.88525	16787.83393
Q4V7A0	Wdr61	WD repeat-containing protein 61	147.46	10.49	33747	2	11041.72148	9794.595987	9849.262225	11824.33143	11084.75655	7343.756003	9541.824921	10350.92537	10852.69092	8320.535474	8837.064196	11981.50339	11768.35117	10390.5444	9117.044909
Q4V7C7	Actr3	Actin-related protein 3	974.506	76.56	47357	29	1766075.745	1704942.151	1628515.25	1569365.28	1659848.89	1366354.518	1448919.285	1445146.229	1429707.8	1361007.267	1473985.096	1628892.312	1484060.951	1519311.815	1552936.122
Q4V898	Rbmx	RNA-binding motif protein, X chromosome	185.454	15.9	42258	6	150907.4244	147283.9067	158125.8388	155359.6604	158738.423	141816.0855	158893.0026	131157.1101	138687.6531	140791.7249	154549.2873	151696.5249	167470.4711	159073.2872	148514.3243
Q4V8C8	Cdc73	Parafibromin	102.814	8.47	60603	4	9818.129163	12017.77321	11446.96283	10646.21146	10805.84683	8883.517562	8116.46445	11068.06648	11910.72134	7611.666828	10196.52511	11885.51674	11190.86182	12903.26641	9373.042412
Q4V8H8	Ehd2	EH domain-containing protein 2	30.884	3.5	61237	2	33083.74998	31449.08842	32558.5885	30252.57054	30549.13918	32479.19296	32722.06407	29510.36087	33311.23027	32931.7312	34713.60338	32294.887	35146.98021	36140.49403	36037.12414
Q4V8K2	Ctnnbl1	Beta-catenin-like protein 1	104.16	9.06	64948	5	26626.91994	26853.9094	21826.15451	30039.85825	30417.02182	23773.74978	27030.31586	17774.27288	24726.19232	23727.12407	25906.52418	27387.56857	26301.36562	28088.79911	28337.33761
Q4V8K5	Brox	BRO1 domain-containing protein BROX	159.807	18.98	46192	6	89511.90644	76706.43704	73721.89773	92703.96426	92597.77226	60909.21536	73999.79928	68531.71156	66268.85834	59426.895	85084.367	90901.61016	71812.74552	55224.17223	91838.39033
Q4VA53	Pds5b	Sister chromatid cohesion protein PDS5 homolog B	468.027	10.44	164419	11	58322.05629	54437.25539	62909.79678	60928.57552	57284.43997	46908.42811	54985.73	51596.09495	50658.09821	47993.31943	53761.32019	56179.63991	57167.93168	59508.21558	57484.31587
Q4VC33	Maea	E3 ubiquitin-protein transferase MAEA	39.391	10.86	45336	2	1142.476001	1356.791908	1185.314258	1268.694664	1160.387894	945.4407156	1226.777003	1350.701126	1616.177346	1844.903963	1363.353849	1174.021251	1981.239981	1201.496364	1394.359575
Q4VSI4	Usp7	Ubiquitin carboxyl-terminal hydrolase 7	111.74	7.62	128431	5	18074.14717	23545.41114	23011.13064	21631.46257	20414.89648	15901.01908	19068.99838	17340.63485	17218.91091	19288.84944	20475.70506	19591.35139	20354.15486	19806.57281	20735.91934
Q501J6	Ddx17	Probable ATP-dependent RNA helicase DDX17	518.414	21.23	72399	11	382571.0964	394779.6148	400908.9914	385169.7072	408305.7987	344493.4887	359586.7877	358662.4066	346908.6849	317371.5553	349438.3321	356185.7016	341548.7244	337746.3795	362462.7091
Q505F5	Lrrc47	Leucine-rich repeat-containing protein 47	52.9	5.51	63590	2	11613.5433	11433.05668	9143.527066	9642.712739	9745.668799	8735.835002	10787.65167	9252.686174	5131.81369	9425.62147	11703.48697	10777.29064	10628.70676	10073.08917	11139.93957
Q52KI8	Srrm1	Serine/arginine repetitive matrix protein 1	188.869	7.29	106862	4	20975.69492	26786.63415	27189.71943	31318.00516	27441.88088	24579.28738	26243.64705	30000.20915	28620.55614	26987.63276	29875.85376	35379.45436	26794.70218	27659.96593	27913.14848
Q53UA7	Taok3	Serine/threonine-protein kinase TAO3	199.228	8.46	105474	7	60489.16505	64976.51773	63679.88612	62499.37005	68311.91825	62915.84217	62957.58382	63263.10873	63137.11634	66214.2362	70226.7957	67782.90726	69352.54815	65416.17723	68031.78984
Q568Z6	Ist1	IST1 homolog	30.577	4.37	39942	2	29229.27588	28229.80586	30006.92311	26951.49598	28852.98906	21458.1813	23527.56004	23585.13805	21060.87815	22350.27129	28124.49394	29527.47193	28100.6788	30162.50346	29263.49928
Q569Z5	Ddx46	Probable ATP-dependent RNA helicase DDX46	110.456	7.95	117448	8	95368.04536	86651.61868	84860.4266	96289.01287	95277.03472	107070.2033	89382.00945	94456.57969	95170.49521	89994.63269	85468.40658	95596.85973	94909.37597	87231.60282	87655.22707
Q569Z6	Thrap3	Thyroid hormone receptor-associated protein 3	155.525	9.57	108178	6	38941.18454	25087.79985	32845.90689	36303.03421	31278.42971	22169.063	25250.42224	24093.65307	32559.87017	30315.11701	26503.04156	30454.76656	32289.80836	22043.90257	29231.74461
Q571E4	Galns	N-acetylgalactosamine-6-sulfatase	163.699	25.77	57673	7	51617.48605	44505.06976	23999.49399	37197.71664	30920.64407	20834.83102	21456.83516	22093.23098	17908.89949	24035.33821	30718.95774	31565.5298	35378.47977	31256.81472	26642.8837
Q571I9	Aldh16a1	Aldehyde dehydrogenase family 16 member A1	241.958	13.47	84756	7	53071.13709	54356.22411	54163.51618	54131.22864	50558.61801	43078.79398	43293.42912	45555.16405	42659.22535	41081.78746	46434.34188	49377.74487	43997.8391	43720.82352	51199.78038
Q58A65	Spag9	C-Jun-amino-terminal kinase-interacting protein 4	485.956	14.91	146219	15	123751.4524	123351.1902	110301.12	118169.0009	112569.005	119174.1283	128603.2823	121638.941	122629.5207	128927.7941	114450.5766	119365.5361	124725.6407	121667.6011	133728.7562
Q5BJP6	Gfm2	Ribosome-releasing factor 2, mitochondrial	53.038	5.26	85914	2	5413.826882	4906.565261	5197.015928	5762.704241	6324.681398	5206.972031	7404.351164	7395.222423	4748.316413	5723.457472	5859.740098	5863.410839	5415.06077	4937.131267	5675.570921
Q5BJS0	Dhx30	ATP-dependent RNA helicase DHX30	86.006	3.02	133997	3	8205.62142	9570.055576	3245.592181	8772.03737	8588.041454	9905.753645	9795.961149	9753.053367	11058.86522	9310.624167	6650.393	9318.131304	8498.984604	10169.32752	9954.627612
Q5F285	Tmem256	Transmembrane protein 256	76.311	31.86	11656	2	33112.33238	31414.20299	32556.46779	32373.0902	28818.59895	34652.99475	30899.20393	27871.91626	32020.64298	36668.60308	31753.63091	27551.55524	38639.33479	29628.43304	36389.20218
Q5FVM4	Nono	Non-POU domain-containing octamer-binding protein	433.828	26.47	54925	12	276780.9835	269520.0566	238543.1654	341736.6444	328513.3133	197334.623	207591.8157	260489.5108	275234.3355	196783.4636	290609.1145	306539.6429	314707.9225	233772.3936	236538.6827
Q5FWK3	Arhgap1	Rho GTPase-activating protein 1	222.831	31.89	50411	9	48830.07047	42593.1852	36523.37057	34618.40106	32952.90055	18463.88409	28107.74492	27820.39439	31418.68188	28058.37765	27186.56088	24534.25087	29733.3225	29983.6037	25062.1509
Q5FWT7	Ublcp1	Ubiquitin-like domain-containing CTD phosphatase 1	73.499	8.81	36867	2	2971.733572	4958.760601	5241.04152	5021.189559	4234.776754	3359.821373	5096.500015	5756.237974	5586.485077	4435.546915	5163.797634	5576.916492	4636.318537	3856.204274	5757.159202
Q5HZY2	Sar1b	GTP-binding protein SAR1b	136.593	33.84	22410	4	11630.89059	10201.30365	10047.66407	9011.973526	10085.90498	10720.51581	10692.56224	11553.74447	12144.73274	8754.489714	11074.74467	8918.531105	10479.44059	10368.55604	9414.259983
Q5I0E7	Tmed9	Transmembrane emp24 domain-containing protein 9	65.897	8.94	27028	2	45228.27238	50973.32113	48133.73875	49926.57109	52561.96465	57093.55397	54511.64738	49968.07899	52711.53387	52305.5368	52263.76862	48744.96643	51773.41638	49642.44957	47800.07701
Q5I0H4	Tmco1	Calcium load-activated calcium channel	107.898	19.68	21175	3	57800.30508	59119.51373	54312.47477	56455.67237	50699.34013	59126.26518	71282.64104	61628.98277	61824.91489	61044.49184	52676.82761	57165.82872	49651.42453	57343.70673	57460.98422
Q5M7W1	Txnip	Thioredoxin-interacting protein	103.107	7.11	44018	3	20962.99141	18833.04871	20046.88937	20832.6732	18015.2887	13029.57045	19793.57751	9094.955321	11000.71616	9498.805108	19373.51639	20911.0384	19464.68638	18140.36814	23130.33466
Q5M818	Mrpl16	39S ribosomal protein L16, mitochondrial	73.028	10.36	28895	2	20240.15566	22709.2821	15678.00238	17320.30559	16633.60595	21259.82573	21749.26864	18010.30801	19569.98758	18990.67872	18327.94837	18326.06622	18586.85321	21112.8952	20197.00544
Q5M823	Nudcd2	NudC domain-containing protein 2	100.62	28.66	17674	3	36405.84033	33874.49882	30885.19014	35591.04016	32689.22585	33808.61408	28830.49802	35071.5493	33869.32868	33557.53356	39741.88018	31272.69372	28659.55679	29997.644	29771.33842
Q5M9G3	Caprin1	Caprin-1	245.688	11.6	78121	6	69811.90636	67894.14308	69181.10234	74174.97828	59689.50735	65413.60523	75723.66479	75978.38989	64523.39498	64491.32997	64820.2285	76082.21342	63136.59671	69697.0338	61541.66224
Q5ND34	Wdr81	WD repeat-containing protein 81	58.923	0.88	211945	2	2263.018824	2177.631633	1816.538698	2392.686463	2242.215742	3014.413025	2428.037983	2780.153532	1723.471054	0.013320187	2363.321556	2189.214659	0.00981159	2217.534711	0.010360417
Q5PPI1	Srsf9	Serine/arginine-rich splicing factor 9	31.498	7.69	25498	2	42183.87431	34671.71356	41685.47264	39730.69751	36487.47276	39069.06989	40024.83742	30831.42927	32360.43387	33268.50549	34045.88724	36150.32958	38139.09086	37583.42507	32711.49256
Q5PPJ9	Sh3glb2	Endophilin-B2	17.669	5.69	44853	2	19048.99053	16738.34464	16662.08745	16862.34538	17873.94871	12172.2375	14580.19067	13465.44487	14820.67278	16787.82784	17969.30531	16573.62587	15722.51767	13094.67454	18537.94789

Q5PPK9	Eipr1	EARP and GARP complex-interacting protein 1	20.754	5.7	43157	2	6511.516164	11857.29875	11009.34021	6682.294242	13897.05903	11930.60057	5796.224016	12093.61746	13125.40985	5272.338506	6046.279308	14149.83717	13437.46823	13172.54868	12529.17302
Q5PQL2	Cnot9	CCR4-NOT transcription complex subunit 9	87.826	13.71	33601	3	4082.696539	5422.856108	4709.99879	5469.990126	3443.493021	4919.885582	4004.460795	3964.181129	5213.715249	5779.042213	4520.258451	4974.762568	5187.538579	5161.063914	4409.171195
Q5PQL7	Itm2c	Integral membrane protein 2C	100.581	16.36	30480	2	9027.525895	8825.450672	7606.42546	8463.814369	7256.727749	6033.284753	9490.328697	9015.212901	10420.62755	10267.21173	8712.973299	8259.913762	9413.955272	5067.13466	8361.201335
Q5RJQ8	Calhm2	Calcium homeostasis modulator protein 2	80.793	9.6	35931	3	3561.209358	4117.119468	5068.17975	4516.991491	5155.971065	2909.925956	1232.810405	4249.972822	1966.383563	2709.763053	4384.126608	3866.522877	4474.632116	5079.767148	2747.265516
Q5RK09	Eif3g	Eukaryotic translation initiation factor 3 subunit G	45.846	9.69	35651	3	43589.02287	44396.205	18825.62933	45899.9155	17640.64898	50197.99594	50920.32831	57163.1226	54529.81172	52158.22552	47268.3534	17140.44442	48110.06296	18246.07819	44371.64419
Q5RK19	Snf8	Vacuolar-sorting protein SNF8	54.292	10.85	28884	2	10759.76383	4664.18462	12126.93025	10099.61712	10363.51076	10463.54121	12714.32079	11718.12425	10556.49086	10188.85486	10397.48547	10658.24333	10866.03932	9659.239557	11013.56321
Q5RKH0	Glyr1	Putative oxidoreductase GLYR1	137.288	15.58	60421	5	24412.86348	18939.52564	23586.49491	21317.63887	19651.98663	20331.66309	24171.47277	12669.61934	20096.03783	22052.12721	23531.53857	17923.69059	16447.62976	22416.10646	25802.74974
Q5SF07	Igf2bp2	Insulin-like growth factor 2 mRNA-binding protein 2	92.866	4.56	65584	2	7870.146324	9008.782535	7396.803104	8628.695909	6502.567311	6370.768983	6693.734266	6495.010978	6399.67889	6444.060403	10133.34662	7346.271626	6493.504157	6490.099207	6056.516966
Q5SQX6	Cyfip2	Cytoplasmic FMR1-interacting protein 2	405.784	12.13	145659	12	119701.2327	131291.2276	107643.3264	114642.3722	111740.1708	92649.23763	115526.3223	114284.3848	117739.1686	113238.5853	104040.6805	113586.226	120343.9906	116928.1499	115562.7084
Q5SSL4	Abr	Active breakpoint cluster region-related protein	138.765	8.5	97667	5	35951.80832	36135.44558	32211.71189	32519.26612	36357.83516	21597.37262	26624.06958	26189.14291	27570.90555	12617.71022	34525.83613	36126.14696	16494.93879	34093.43055	16220.41075
Q5SSZ5	Tns3	Tensin-3	99.934	2.92	155589	2	1672.916438	1931.002578	1366.526116	1185.949528	1931.066617	6749.083659	6626.062282	2523.573115	5631.795482	1432.960599	1697.357521	1711.026619	5526.015726	1598.026404	1658.302533
Q5SUA5	Myo1g	Unconventional myosin-Ig	451.415	19.82	117227	15	113588.1195	94621.11166	92960.47978	94553.04217	97848.70048	101606.1466	92949.42187	100502.5965	99749.31342	100910.5525	92467.35242	80666.69393	107930.8867	99297.6652	108584.3462
Q5SUF2	Luc7l3	Luc7-like protein 3	234.75	15.74	51450	4	8953.687337	12545.71491	15053.82489	14363.08781	17011.12013	14628.05877	17613.93291	19339.41314	9599.968441	13423.11786	9047.336283	15306.98892	17198.30471	14386.37625	16259.53427
Q5SUQ9	Ctc1	CST complex subunit CTC1	84.872	4.04	134028	3	9655.508909	8157.560805	7264.729988	7323.410559	3928.298046	7276.517136	7341.562261	6249.993524	7177.243326	5930.840661	6529.396475	8070.266444	7257.031704	7128.958305	7983.002769
Q5SUR0	Pfas	Phosphoribosylformylglycinamidine synthase	587.731	26.78	144629	19	162586.0237	190841.2583	180979.3116	156914.8044	170403.0378	110112.838	122482.193	147360.8808	122632.8557	120926.491	147711.0848	168394.5214	161639.4038	167991.8069	156027.5431
Q5SVR0	Tbc1d9b	TBC1 domain family member 9B	117.917	5.38	141779	3	1231.576879	7695.843186	7246.070912	7653.695246	6092.166919	4168.352429	6261.519891	7015.096384	6515.44982	4673.802168	6280.13292	7706.476578	6808.442073	5276.022271	6556.920583
Q5U216	Ddx39a	ATP-dependent RNA helicase DDX39A	493.935	43.09	49109	14	561718.7321	608585.1089	602266.4299	603949.9429	627965.6437	615932.3223	614283.1545	648608.1493	597712.1883	603089.5378	566968.7821	646399.2621	610795.5955	653125.6317	644660.3592
Q5U2M6	Dcaf8	DDB1- and CUL4-associated factor 8	33.859	7.61	66156	3	4148.764582	13672.37116	10517.58118	11579.50194	11011.5231	10802.03501	10802.78089	10295.9215	10666.62187	11919.45191	10411.62605	11873.98471	11313.96662	11778.14048	10590.95404
Q5U2Q7	Etf1	Eukaryotic peptide chain release factor subunit 1	237.92	24.94	49031	9	67121.51218	78506.33735	53044.30351	73661.70371	68082.90023	69766.30502	84668.63393	90579.91776	53083.09198	72745.0306	67879.72715	53644.07907	46773.1455	50031.35099	54143.19547
Q5U2R0	Mat2b	Methionine adenosyltransferase 2 subunit beta	219.535	23.65	37375	5	46696.22901	48949.34644	48121.23796	51436.79507	48538.74792	41249.09108	44280.90572	45267.08457	39959.87828	43664.99795	45516.51884	42744.55754	46525.38813	49719.73151	46176.28891
Q5U2U0	Clpx	ATP-dependent Clp protease ATP-binding subunit clpX-like, mitochondrial	66.44	3.79	69208	2	3507.794199	4361.505095	4948.785685	3993.649937	3744.060565	4187.704393	5626.992438	3971.194157	4664.947871	4644.066916	3576.311214	4607.193602	3707.566608	3873.014383	3913.498455
Q5U2X6	Ccdc47	Coiled-coil domain-containing protein 47	32.37	5.59	55733	2	13682.20957	15981.18046	14169.39306	15361.51376	14531.27256	15199.87178	17711.11232	16179.08774	15712.99616	15690.7839	15198.26481	15401.89436	14200.31814	13891.89007	15903.98031
Q5U316	Rab35	Ras-related protein Rab-35	177.192	30.35	23025	5	11840.5737	12677.6819	17654.15372	17237.97201	11600.16178	12562.02139	20313.16734	13434.56855	9121.249604	17126.52023	13486.1514	12258.27433	10914.96972	12734.33718	9268.387285
Q5U317	Fip1l1	Pre-mRNA 3'-end-processing factor FIP1	51.334	10.45	60188	2	4937.598654	5018.151049	3463.421025	3971.633006	2266.918798	2150.853699	3486.431872	2058.418259	3278.717922	2798.18312	3719.352961	3728.453546	3158.012966	3103.690045	2734.156015
Q5XFW8	Sec13	Protein SEC13 homolog	117.126	22.67	35548	4	32993.26852	28381.51548	27039.44847	31861.55125	30902.84148	38513.77677	18400.97217	34724.70276	35087.96544	18866.91254	17082.58772	21145.98338	25797.54537	34886.58812	29603.65766
Q5XG71	Utp20	Small subunit processome component 20 homolog	39.868	0.93	317744	2	4147.137824	3990.909858	3863.092805	3836.485009	4516.362	4227.408078	3564.407917	4444.05511	4951.234965	1826.893605	4389.879799	3845.888993	5126.610419	4325.944398	4553.082562
Q5XI28	Raver1	Ribonucleoprotein PTB-binding 1	121.315	9.22	79289	4	34441.20019	29722.93678	26301.93535	31779.1466	27131.35736	21547.8228	29303.67611	23615.35477	24188.34415	28319.67309	32882.7771	30369.21263	28927.54075	29047.6483	30439.6345
Q5XI31	Pigs	GPI transamidase component PIG-S	251.113	17.84	61724	6	36620.83149	37715.98669	32315.63389	31443.36269	30773.09849	38533.83806	39281.11352	39038.13236	40020.20459	36346.46102	33901.77182	36226.06328	29191.91159	29797.46038	34538.20237
Q5XI42	Aldh3b1	Aldehyde dehydrogenase family 3 member B1	162.562	9.83	52147	4	36639.12686	43993.51771	37735.69485	36338.2352	36729.32352	42694.32533	41452.47538	34753.86064	38974.24245	43570.21483	44614.71219	41793.06218	40722.66162	39899.1337	33877.38392
Q5XI72	Eif4h	Eukaryotic translation initiation factor 4H	172.757	27.02	27324	5	103195.7983	94162.35284	106601.3722	86008.24237	91348.84231	75900.61004	87672.52991	75426.85841	80600.29896	79030.70027	94454.56089	85413.02588	94123.35967	85847.76208	84555.56136
Q5XI73	Arhgdia	Rho GDP-dissociation inhibitor 1	574.961	77.94	23407	15	1329253.127	1394223.181	1243687.006	1284243.592	1212135.717	930104.4115	1040009.049	1015409.171	1056539.656	1019467.658	1248651.194	1187474.257	1209901.182	1182092.692	1102206.113
Q5XI81	Fxr1	Fragile X mental retardation syndrome-related protein 1	163.162	6.16	63947	3	40668.46406	32185.69417	38930.63975	37208.34824	34220.11252	41174.32743	42280.74311	41196.12123	42642.48684	40146.18419	33090.61012	37635.42748	39739.63349	39230.18514	38292.16984
Q5XI83		UPF0505 protein C16orf62 homolog	143.199	4.49	106151	3	10663.46018	7313.718101	7138.054892	7810.423125	7333.176611	4603.796667	6541.331745	7416.326218	6707.988802	5168.332822	7213.326638	7817.758499	6061.830983	6140.296817	6298.19052
Q5XIA1	Ncln	Nicalin	121.531	8.7	62992	4	11330.55737	11911.85496	11476.20702	13136.71636	10633.2309	12807.71397	11187.67431	10865.0956	12389.66451	15188.6728	12824.72454	11844.63499	9823.405783	13447.73408	11507.51679
Q5XIA4	Cnot10	CCR4-NOT transcription complex subunit 10	50.369	2.96	81819	2	0.008226335	1612.957509	5565.029573	2032.049733	1971.848299	2088.296212	1638.023175	5284.673694	4739.509135	2773.494753	5295.00559	5243.650385	5619.373644	2466.485523	6992.857751
Q5XIG8	Strap	Serine-threonine kinase receptor-associated protein	433.385	52.86	38456	12	98474.02988	110669.5258	98729.17669	107124.2866	99949.55119	116158.3558	113822.6033	115102.451	113179.2125	106028.2616	90798.92706	96434.08543	97477.51064	96939.76475	107360.8742
Q5XIP6	Fen1	Flap endonuclease 1	128.983	21.32	42609	5	110674.3433	124623.4381	115295.0725	112882.8868	127345.3272	97988.9717	104580.3014	102051.7032	99004.45116	89945.81421	116772.7012	122345.6676	119407.8472	112500.1621	122774.7498
Q5XIW8	Sart1	U4/U6.U5 tri-snRNP-associated protein 1	78.484	3.47	91011	2	9079.414533	7412.986151	9434.138256	5143.340635	12467.98811	8166.336504	6798.123636	9086.38834	9170.364766	5638.188296	10646.11585	9756.08006	8721.314011	6186.183705	11098.7608
Q5XJY5	Arcn1	Coatomer subunit delta	358.462	29.16	57229	13	196192.4791	206852.5107	199564.4102	206922.9433	196325.631	193691.7344	213561.9078	226343.7165	216162.3061	204897.2557	191096.2597	205283.4261	180052.3834	208418.7746	191726.8348
Q5XXR3	Arhgef6	Rho guanine nucleotide exchange factor 6	58.264	5.57	87013	3	24628.58679	27403.92957	26247.47443	22614.26326	23640.91864	19923.79264	23049.29129	24236.08199	21786.48251	22044.03853	21626.16421	24313.96717	22059.19705	22483.88714	23125.80975
Q60520	Sin3a	Paired amphipathic helix protein Sin3a	33.77	2.04	145088	2	10602.4506	10149.019	10657.20104	9506.475399	10954.40602	9693.604305	11400.70247	6558.493303	8818.654409	8999.984088	8060.783731	10314.27166	11074.37617	11343.69733	10123.99908
Q60591	Nfatc2	Nuclear factor of activated T-cells, cytoplasmic 2	116.59	6.15	100020	3	16110.44895	16685.56654	17786.93594	16742.73585	16268.94829	9936.259017	14700.47561	11118.50174	13395.46448	10343.4315	16103.67423	11464.32147	16626.7342	16732.94565	14815.32327
Q60597	Ogdh	2-oxoglutarate dehydrogenase, mitochondrial	708.109	30.6	116449	22	198499.7523	207267.0672	208339.5344	209391.7494	205978.2862	235146.2729	231059.9919	225411.2475	240708.4016	242221.4584	213400.9061	204816.5296	221269.4412	223237.4639	229031.6698
Q60605	Myl6	Myosin light polypeptide 6	651.916	66.23	16930	13	684405.2443	623847.6972	611447.8748	610863.5244	563355.5904	542839.5292	486386.847	437479.3559	504853.1484	460677.1857	641871.9484	568688.9428	555644.0977	519630.599	519206.7786
Q60631	Grb2	Growth factor receptor-bound protein 2	219.982	46.54	25238	8	87459.52627	90074.55709	84878.01044	86045.3028	83705.42735	88471.45891	97293.19521	95259.16961	92062.30977	88020.9806	92660.0642	90645.18386	98467.77481	96050.6135	94768.48193

Q60634	Flot2	Flotillin-2	156.587	19.63	47038	6	22616.62054	27558.67361	26241.90875	28586.66937	20491.30815	18240.48371	20413.41997	23181.58309	19652.71498	22727.38077	25309.3329	25503.02956	26659.09129	26945.29597	29121.41135
Q60668	Hnrnpd	Heterogeneous nuclear ribonucleoprotein D0	240.764	22.54	38354	7	220214.6122	204550.1938	216852.1848	212401.4121	196266.3814	146643.4007	174920.7416	157429.2552	173467.1085	184576.8443	207717.6869	207856.7644	207018.1261	218611.1389	194459.6019
Q60692	Psmb6	Proteasome subunit beta type-6	88.712	25.21	25379	3	66424.88554	64351.03789	64294.13645	64340.04724	63792.60533	52010.3056	65632.9363	75454.1451	61847.97	55045.56917	60690.90991	67164.72896	60813.39858	57901.31398	56107.29218
Q60710	Samhd1	Deoxynucleoside triphosphate triphosphohydrolase SAMHD1	668.224	44.38	75893	21	505010.4566	373497.1621	485182.0759	475224.7725	434538.6262	295141.222	323234.7323	294107.2208	318118.0581	338241.5975	465358.6438	330105.6472	354694.4106	390086.9545	370168.6212
Q60749	Khdrbs1	KH domain-containing, RNA-binding, signal transduction-associated protein 1	133.223	14.22	48371	5	92468.27038	81439.19341	98077.40499	109757.8883	85495.55615	79833.03693	92202.16013	73191.72386	94229.18125	99886.07695	87228.7623	81787.84347	72634.71149	100000.5927	88510.5209
Q60759	Gcdh	Glutaryl-CoA dehydrogenase, mitochondrial	34.53	5.94	48606	2	7488.497836	6194.620028	6689.850723	5992.205974	6796.957943	6873.254854	6858.070847	7284.632289	6683.63093	8938.410725	6917.087596	6679.402033	6701.003983	6948.730543	6913.006134
Q60766	Irgm1	Immunity-related GTPase family M protein 1	63.557	5.13	46552	2	3109.406682	2624.338575	2939.70212	3009.784256	2047.555549	2438.742382	2559.855659	2966.466193	2679.376071	2288.623299	2781.984115	2484.923126	2584.757025	2439.600582	2423.916757
Q60770	Stxbp3	Syntaxin-binding protein 3	185.306	17.74	67942	8	41791.46578	40750.80301	38270.44226	38075.65951	33812.65734	29742.05391	32159.93991	31519.31786	29386.17162	34653.77828	36757.44659	37599.55434	36363.08757	40730.52562	37514.95987
Q60787	Lcp2	Lymphocyte cytosolic protein 2	55.921	5.82	60238	2	5270.589397	9242.549093	5658.393758	8365.674389	3943.244042	8111.194314	5934.274955	6261.067062	6674.353124	5198.182162	7612.479887	5001.687782	5050.993131	4782.572692	4928.395804
Q60790	Rasa3	Ras GTPase-activating protein 3	74.747	6.24	95987	3	5867.73805	10570.88269	6514.075305	9149.384172	6059.950733	6244.469451	7473.672411	6540.599479	8501.081855	6240.276529	3483.549298	8861.669715	6191.038587	8929.89336	9701.871003
Q60854	Serpinb6	Serpin B6	1109.272	64.81	42599	24	660602.5734	723713.6316	579163.4549	715942.4348	683350.5356	415486.4148	463123.1615	365765.8905	417954.4565	495858.9962	604772.3479	624981.1813	659147.5828	613749.7362	633045.2546
Q60855	Ripk1	Receptor-interacting serine/threonine-protein kinase 1	56.794	3.2	74854	2	6949.186489	7435.507025	6817.591453	6675.169441	6849.478715	4278.405916	4973.819531	6544.64902	5172.007929	6142.997139	5734.576533	7225.109475	6610.561922	6349.848735	6881.857542
Q60864	Stip1	Stress-induced-phosphoprotein 1	602.769	40.33	62582	21	225011.8655	231613.9167	239814.8125	211331.5845	207610.595	196726.0452	224409.6718	223671.3005	228233.368	203628.3214	238859.3151	209478.0684	258236.2948	259641.4778	235451.129
Q60872	Eif1a	Eukaryotic translation initiation factor 1A	127.34	18.06	16502	2	9925.427311	11980.1086	9952.081646	10272.49816	9087.118518	5927.535411	6892.16344	6289.517158	4586.530798	6122.859081	10010.16497	7215.100771	8125.182003	7736.860846	8111.428577
Q60875	Arhgef2	Rho guanine nucleotide exchange factor 2	554.589	18.78	111974	13	155024.113	156775.7972	137978.4129	145334.8187	154501.8892	140079.9026	156975.7661	148216.1833	143139.3058	136948.0784	147175.5312	144008.2917	148200.5767	145097.2428	149974.5007
Q60902	Eps15l1	Epidermal growth factor receptor substrate 15-like 1	75.536	5.51	99309	4	37133.82577	34636.58992	28899.51462	31580.35834	27206.35556	15783.43027	22943.99894	20570.67845	23649.24384	33461.71813	36160.36595	38122.16376	27521.35836	31958.24817	31656.24269
Q60931	Vdac3	Voltage-dependent anion-selective channel protein 3	444.29	43.11	30753	9	302487.3271	315348.568	301203.9735	293642.1846	314706.3862	372556.7556	360616.6999	329930.1402	356257.4805	341221.8028	280655.0482	316139.7247	308659.3126	328859.5007	321541.5499
Q60953	Pml	Protein PML	152.894	7.01	98242	5	17947.97986	28717.65352	25147.95204	20146.85578	18592.04521	21456.49832	19371.12386	21257.94736	20047.53059	15926.83427	13219.76256	23775.5281	26265.64407	19496.41669	19847.20151
Q60967	Papss1	Bifunctional 3'-phosphoadenosine 5'-phosphosulfate synthase 1	131.349	7.21	70794	3	17895.29019	15318.90568	13330.93335	14664.39868	13521.63715	14515.63064	14586.0423	16711.67823	13125.12287	11152.31237	15879.34203	16023.10908	14627.8962	12422.37726	15320.71864
Q60972	Rbbp4	Histone-binding protein RBBP4	374.339	49.18	47656	13	241303.5961	275081.5176	268106.9311	251629.0471	285202.7584	255843.6628	254290.5341	262437.0172	241991.3431	252610.3783	248416.4837	243552.1529	263709.9847	247768.268	246328.5719
Q60973	Rbbp7	Histone-binding protein RBBP7	381.971	45.18	47790	13	231723.1237	245471.0755	246474.2844	246962.1476	254979.9842	234363.2029	232192.6928	246248.7159	227513.899	224876.0175	230471.6616	229125.076	245432.5425	225460.6116	223609.753
Q60996	Ppp2r5c	Serine/threonine-protein phosphatase 2A 56 kDa regulatory subunit gamma isoform	130.812	13.36	60824	5	20521.84183	26493.64537	22699.07934	25405.26396	25340.0982	23136.67392	27315.33716	24159.65146	23001.5154	22865.05322	18620.18029	22585.81133	21760.4464	31346.81543	25019.40123
Q61024	Asns	Asparagine synthetase [glutamine-hydrolyzing]	443.18	32.8	64283	14	153758.409	234647.3831	232282.8818	252629.5044	221285.4997	177159.3427	278507.2502	294488.2625	280276.8778	237510.2681	241823.8418	262651.0031	212740.2455	233356.5795	247868.8965
Q61029	Tmpo	Lamina-associated polypeptide 2, isoforms beta/delta/epsilon/gamma	868.001	53.98	50373	17	421957.8687	430376.5105	441895.5944	440749.8969	465645.2025	376990.9873	432734.2089	394799.3799	392833.216	395620.1408	388783.1737	430285.9096	444020.2657	412381.2964	439831.9384
Q61035	Hars	Histidine--tRNA ligase, cytoplasmic	240.471	21.61	57432	9	101262.1488	117547.2555	118477.671	111212.3978	106370.3547	108947.5967	121064.8475	118674.9809	117460.9365	109397.8093	109342.4833	116188.6559	110699.3348	112779.2376	108293.2185
Q61036	Pak3	Serine/threonine-protein kinase PAK 3	120.297	10.38	62398	5	59927.29812	55826.62997	58585.74192	60343.33988	58292.4904	49720.40715	52724.44655	55581.50097	52511.12486	52183.68373	54607.89218	53365.78879	57165.12066	59227.80675	60735.49232
Q61072	Adam9	Disintegrin and metalloproteinase domain-containing protein 9	130.389	9.59	92079	4	15993.91674	18652.44499	9534.500599	15956.40886	15878.46084	15277.23987	14523.91558	5170.492235	6507.47853	13260.49915	9889.019063	9249.350647	12219.79225	13658.93787	16363.64221
Q61081	Cdc37	Hsp90 co-chaperone Cdc37	260.866	21.37	44593	6	57375.82207	49135.38187	55387.02412	51341.48896	58436.399	50207.80303	55338.72348	59501.5786	50610.45643	45810.15078	56345.82253	51495.74349	55636.15113	56052.77453	47394.66354
Q61093	Cybb	Cytochrome b-245 heavy chain	329.147	37.89	65305	13	92345.41829	95114.95253	72173.37885	95977.42462	89216.13498	126171.5812	125386.5542	120332.9705	136941.4587	105463.0993	86907.01007	87251.15827	95345.81512	92847.84584	99382.32771
Q61102	Abcb7	ATP-binding cassette sub-family B member 7, mitochondrial	70.283	3.32	82581	2	17254.34555	17118.20478	16326.55973	18841.1027	16754.46418	14884.72345	17377.92116	12107.70864	14345.12807	20679.38205	18146.90792	15709.00226	13639.85313	18631.1037	18059.35233
Q61151	Ppp2r5e	Serine/threonine-protein phosphatase 2A 56 kDa regulatory subunit epsilon isoform	111.011	12.85	54713	4	8558.428377	10334.68765	10869.55393	14149.44007	12286.82528	10265.77631	12713.49882	11106.9578	9623.209325	10129.58089	8641.444641	9977.279668	9654.96472	12867.81341	14316.13379
Q61160	Fadd	FAS-associated death domain protein	65.041	17.56	22960	2	4142.443137	3545.696266	2749.624737	3774.687544	2957.885463	2708.094929	2483.402799	2657.547601	3017.079003	2782.174919	3731.058974	2760.612009	2966.28012	3212.017143	1667.557442
Q61164	Ctcf	Transcriptional repressor CTCF	76.872	4.76	83745	3	32367.7606	29828.61587	25708.14323	34608.95711	30349.24798	23403.38837	25626.60615	27212.90435	27312.38149	24001.37672	27806.21776	31225.56253	28198.34582	30810.12324	28461.142
Q61166	Mapre1	Microtubule-associated protein RP/EB family member 1	342.639	41.42	30016	9	193075.6687	197740.677	167019.3995	161763.0166	183168.1194	172277.1253	161482.4529	167239.1906	170573.9107	169183.7966	168219.046	166850.2033	171962.1582	175295.581	181688.337
Q61171	Prdx2	Peroxiredoxin-2	483.201	58.08	21779	12	705357.852	655334.5713	657060.5963	639043.0976	634092.629	621645.0094	674138.6781	620226.2079	651620.769	662318.6547	692949.2119	687285.0188	702919.4414	664686.3473	674906.3109
Q61183	Papola	Poly(A) polymerase alpha	33.647	3.38	82309	2	11179.77995	10965.59352	10779.80408	7178.608554	12738.15151	9066.150165	10626.93782	9354.735294	0.012083387	8688.962719	6010.929852	8443.472948	6330.382153	9931.850461	6471.171248
Q61187	Tsg101	Tumor susceptibility gene 101 protein	84.606	12.28	44124	4	29500.6853	32339.5969	31138.97656	27858.18797	29654.06858	20025.10232	27882.58284	24539.80271	25906.63047	25129.81967	28555.53668	28902.22678	31342.51895	30279.98429	31758.94291
Q61191	Hcfc1	Host cell factor 1	498.608	10.12	210437	13	125159.5131	130614.5124	124347.8673	124767.1514	124000.0431	99320.84641	107680.534	113017.6502	99736.83733	104117.4075	107000.6771	115765.4239	114180.8501	101513.295	120377.5463
Q61194	Pik3c2a	Phosphatidylinositol 4-phosphate 3-kinase C2 domain-containing subunit alpha	104.732	3.8	190758	4	13621.62158	12833.81081	11459.17	14013.89689	11360.32375	9146.650637	12543.73743	12253.31406	13316.22767	12052.17758	11676.46407	10881.82497	11827.31638	12334.35056	11416.43707
Q61207	Psap	Prosaposin	294.177	14.54	61422	7	589201.4706	461611.7679	409299.1351	430921.1517	408274.9506	316180.7189	175995.0958	177756.025	281808.5921	161118.3967	383491.1715	346564.3173	351911.0195	374321.4207	353548.4823
Q61210	Arhgef1	Rho guanine nucleotide exchange factor 1	557.637	26.09	102805	17	109524.6718	109387.356	107089.212	118674.9707	108602.1035	89267.75283	103829.0203	103426.8347	99877.94108	93913.30514	97443.64532	99114.10797	104503.5707	102366.8526	103333.3451
Q61216	Mre11	Double-strand break repair protein MRE11	66.562	10.76	80223	3	6131.946347	9861.110269	5276.349466	8868.616566	5338.042107	4257.61313	7980.757066	5796.633394	2215.151015	5368.807496	6641.796405	2835.720768	7594.853928	6017.318302	7231.39531
Q61233	Lcp1	Plastin-2	3330.826	89.31	70149	72	9896911.616	10154292.97	9734325.353	9910798.791	10046963.48	9153070.351	9685743.201	10146162.58	9923397.014	9146990.693	9312125.713	10149656.34	9436805.023	9963659.98	9924556.41
Q61235	Sntb2	Beta-2-syntrophin	61.084	3.85	56382	2	5140.580393	8653.872694	5714.992032	8201.490448	3101.152389	5461.374029	5818.792766	4029.016125	5253.135366	9008.420762	10272.56077	6115.042548	5781.885465	9061.810479	9429.75207
Q61239	Fnta	Protein farnesyltransferase/geranylgeranyl transferase type-1 subunit alpha	101.808	6.63	44013	3	5501.765338	8204.971479	4540.633151	5280.142549	5245.278009	4501.945808	5751.238525	6742.32829	6834.764677	5061.735962	6421.216369	4807.231157	6253.768989	5206.068998	5004.814237

Q61249	Igbp1	Immunoglobulin-binding protein 1	209.692	19.71	38971	5	18888.50912	14228.83734	17294.8619	16565.50542	18052.66072	17208.75226	14415.20888	13984.36862	18532.37356	20762.1487	12534.22662	18031.14246	20351.25921	16518.38485	18557.50576
Q61263	Soat1	Sterol O-acyltransferase 1	202.63	14.26	63799	6	90734.74296	103792.5826	89639.83533	91594.48082	94171.49262	109550.1842	115940.132	119653.9414	119443.4457	104679.2063	87121.60007	107207.957	109591.5082	99097.03004	100217.6777
Q61316	Hspa4	Heat shock 70 kDa protein 4	1248.721	50.3	94133	30	570058.9199	606902.5621	582024.6847	604726.3685	582684.3427	560357.7775	620476.1612	615575.6105	621034.0917	525476.1654	542667.3037	576275.9079	522053.9617	568940.4549	576141.1221
Q61390	Cct6b	T-complex protein 1 subunit zeta-2	159.704	11.11	58185	5	126565.3445	136008.7533	140530.8558	143298.7328	158177.7001	152990.6338	159255.0104	172848.7244	163772.861	152871.3229	149721.9015	138786.0332	148133.8088	161146.8355	156187.1142
Q61425	Hadh	Hydroxyacyl-coenzyme A dehydrogenase, mitochondrial	342.335	54.14	34464	9	120701.843	123153.0776	110002.9271	119194.1652	110554.6978	114833.6129	119599.9975	103706.0755	122101.7304	125028.8555	118938.6448	109418.23	125389.8952	119961.5829	119554.3283
Q61490	Alcam	CD166 antigen	79.013	7.89	65092	3	5563.281873	8138.148084	9526.122362	7819.704832	8250.711751	8068.490923	9373.370124	8890.988167	9422.446873	8800.381088	9591.129499	8821.708984	9115.514269	7234.643538	8502.931213
Q61510	Trim25	E3 ubiquitin/ISG15 ligase TRIM25	188.355	15.93	71726	7	21222.86751	18863.78993	15262.47486	18079.95842	14700.52428	11692.11791	18328.33381	21829.5186	17824.99245	20407.26895	13344.21756	23122.78767	22415.5074	20597.30484	18106.09594
Q61543	Glg1	Golgi apparatus protein 1	509.839	17.28	133734	18	175279.7198	165377.3039	179032.2702	185238.4007	195213.9226	173062.1436	167739.4864	195825.442	200175.1273	131653.5572	115186.5666	178598.3897	145689.0353	154576.079	180163.9467
Q61545	Ewsr1	RNA-binding protein EWS	59.664	4.43	68462	2	46720.73526	47904.92389	51072.16535	46099.14397	46196.12497	43018.08305	43693.2511	41130.42833	35057.89593	39678.96532	47289.06489	44257.62848	44011.62438	46609.73527	43485.22248
Q61550	Rad21	Double-strand-break repair protein rad21 homolog	168.295	9.45	72083	5	33811.17191	48118.18402	42196.26458	45007.72303	49995.52703	46143.58014	43552.66064	38572.12349	36938.31562	43005.33567	33981.83371	37713.97117	45114.30485	48059.78929	49482.417
Q61599	Arhgdib	Rho GDP-dissociation inhibitor 2	626.533	76.5	22851	15	906329.0669	954839.4473	749701.4953	953365.0663	831646.6576	554031.0455	712330.3845	756845.4341	710887.4133	753084.4863	705947.9152	900277.2379	853702.2324	813180.4586	790173.4884
Q61655	Ddx19a	ATP-dependent RNA helicase DDX19A	287.911	24.27	53933	7	64954.63375	80890.10909	69627.2839	72070.59519	71097.79375	66886.85768	71711.80608	72554.31913	68957.59922	73299.42344	72122.62097	73051.26218	64078.46031	58378.97261	70295.60304
Q61656	Ddx5	Probable ATP-dependent RNA helicase DDX5	720.907	37.62	69290	17	632698.3832	663860.2572	688241.1811	668341.1787	674778.5754	662589.957	679218.1982	664931.8982	647459.9272	612366.5152	633056.9334	636242.7084	637120.5629	618615.0229	655781.3962
Q61686	Cbx5	Chromobox protein homolog 5	139.648	18.85	22186	3	49362.02233	54164.80424	46912.4799	51829.07069	50157.42215	41020.97253	33184.07296	40552.74536	23389.6791	35131.1205	40249.44036	45330.81583	47204.99283	42488.27948	45513.78688
Q61687	Atrx	Transcriptional regulator ATRX	65.785	0.85	278587	2	4207.143018	5079.447756	2702.399057	4135.511732	4225.44898	3426.815825	3321.819056	3960.359325	3953.570126	3935.683125	4005.034053	4885.841097	4449.90632	3243.400654	3709.417362
Q61699	Hsph1	Heat shock protein 105 kDa	136.096	5.48	96407	3	35949.93272	55413.7372	45238.02594	49653.36392	40580.3996	41349.76829	44655.95049	50644.8099	47292.27381	29853.22288	25737.93557	36814.07445	28071.03798	35759.1955	39120.43853
Q61739	Itga6	Integrin alpha-6	70.801	3.94	122159	3	9366.790247	12869.0838	14258.65671	12244.24417	9774.947505	10358.8074	6331.597482	6447.878452	6416.788241	10146.76893	5919.50843	4888.987871	12301.49213	8362.861071	6879.218226
Q61749	Eif2b4	Translation initiation factor eIF-2B subunit delta	64.619	7.25	57624	2	3349.657587	2867.118096	9135.639207	5996.217735	5349.204116	2448.489718	7725.634504	3028.341867	2729.090689	3521.224311	2246.042477	2859.841116	1759.321925	1916.396536	2746.149855
Q61792	Lasp1	LIM and SH3 domain protein 1	265.161	40.68	29994	12	230908.6343	230513.1617	246331.2552	224309.3932	212244.0552	210817.7861	233793.7465	188793.6673	210439.4449	204744.3133	249224.1512	254737.4006	207214.9466	242863.189	218713.503
Q61820	Rasl2-9	GTP-binding nuclear protein Ran, testis-specific isoform	154.659	25	24452	6	495904.6931	507244.5035	499892.1684	478534.2936	544403.1662	473682.2528	488822.7564	489625.8853	472539.5618	447947.6692	489311.2938	494143.05	447044.4333	465193.2888	463605.2405
Q61879	Myh10	Myosin-10	1972.797	32.44	228996	54	1591061.053	1582775.863	1407987.264	1458064.326	1430319.478	1374298.948	1381853.141	1362828	1366338.266	1374689.976	1447137.472	1421059.631	1489701.438	1512569.369	1487884.309
Q61881	Mcm7	DNA replication licensing factor MCM7	769.311	47.15	81211	25	303139.2148	323951.0881	330091.1086	347206.8042	309513.4761	294407.9218	313732.3839	324501.74	340314.974	337610.8067	335809.7872	284644.2317	331732.9741	345500.5024	315354.5991
Q61941	Nnt	NAD(P) transhydrogenase, mitochondrial	101.854	5.06	113838	4	40428.92551	41623.89147	37844.56676	37491.4304	40652.28778	34424.59631	40024.19221	33712.55379	39423.53903	34323.85058	36355.94225	38075.84544	33676.62757	38461.22398	40039.0112
Q61990	Pcbp2	Poly(rC)-binding protein 2	555.288	49.17	38222	13	468765.5193	496582.2233	490541.1046	499343.271	495485.1186	455836.9172	457172.3825	433708.265	451750.868	404804.1699	419994.5322	441432.592	422240.8118	432624.5204	484177.1324
Q62048	Pea15	Astrocytic phosphoprotein PEA-15	224.718	70.77	15054	6	106979.8303	95154.29978	65458.23118	93713.62023	87135.43644	54641.25323	53913.95037	46153.38147	74721.83394	48598.3756	93353.95535	78064.08294	82858.54544	62368.67794	69663.16694
Q62086	Pon2	Serum paraoxonase/arylesterase 2	98.867	14.12	39617	3	10041.74666	7229.991761	4169.749889	6361.043642	6544.073288	4013.55351	6598.56612	7274.436229	7652.013916	5025.68694	6924.513437	6757.4459	6952.508104	5127.242086	5941.025517
Q62087	Pon3	Serum paraoxonase/lactonase 3	320.242	38.42	39351	10	86186.08133	84739.74168	83319.83899	83342.7253	82974.72542	92292.32191	98278.49848	80292.45826	88689.0269	89510.13753	89190.93031	83239.95973	98627.28183	83119.66484	79193.77119
Q62093	Srsf2	Serine/arginine-rich splicing factor 2	190.309	24.89	25476	5	54513.89646	44491.09063	38182.92963	51302.89237	43549.61599	45548.54612	47928.92281	40913.44008	35288.4983	54432.36771	45839.3171	47314.34734	39891.512	50805.13489	30918.56548
Q62159	Rhoc	Rho-related GTP-binding protein RhoC	544.859	65.8	22006	11	995975.3258	1003862.837	791622.2005	925992.0366	931960.8572	823927.2936	751904.0334	657663.2222	834003.3024	656075.4831	705463.0701	744487.0671	741883.2283	809267.8035	845635.2023
Q62167	Ddx3x	ATP-dependent RNA helicase DDX3X	692.773	30.21	73101	16	241452.2625	233008.653	272357.9973	289532.0262	255869.6551	250992.3424	251106.727	264955.5029	292100.9311	274388.6175	241225.0111	258735.6474	244843.4938	268267.4184	228284.9324
Q62188	Dpysl3	Dihydropyrimidinase-related protein 3	260.334	8.95	61936	6	251059.7172	247082.354	226037.8902	216506.5081	261508.2935	166790.6192	187431.808	194485.325	179221.2899	191924.5658	229550.797	225627.3641	230858.9559	226327.8525	227989.9921
Q62189	Snrpa	U1 small nuclear ribonucleoprotein A	144.668	16.72	31835	5	125283.3853	87220.50141	138008.9778	130971.3663	130021.928	132334.4497	126491.6438	142332.2134	127836.1433	139219.6498	98877.46924	99402.72341	147895.24	139187.2102	136487.6212
Q62192	Cd180	CD180 antigen	558.941	25.11	74302	13	98727.69716	88824.73177	71470.99537	106245.3189	101675.0823	87278.80278	82617.10784	101671.5225	97297.46131	66863.20237	85931.16429	97823.95184	90473.99757	91917.39876	104910.6668
Q62193	Rpa2	Replication protein A 32 kDa subunit	79.469	13.7	29718	2	39285.432	34457.33798	37094.03219	40613.69595	37482.4466	25509.57828	27061.69333	27655.17448	27415.79413	25298.40994	23859.12043	27450.20789	21595.17997	24904.8401	26136.56236
Q62261	Sptbn1	Spectrin beta chain, non-erythrocytic 1	232.782	5.46	274223	9	41375.05279	38517.85987	32999.27095	38181.67288	38170.35618	31028.50265	31269.79399	25389.27471	32683.42582	34238.648	38056.12685	34064.15102	33564.61355	30688.03717	37221.71864
Q62318	Trim28	Transcription intermediary factor 1-beta	935.768	42.21	88847	22	493681.2792	477324.199	517981.1726	533081.7436	503062.5638	437681.6222	469734.8803	449895.7609	441294.7266	460561.5665	496660.1221	501866.0899	487467.7924	505862.055	458736.7978
Q62348	Tsn	Translin	405.978	41.67	26201	9	102633.0018	119307.6951	105941.403	99471.58759	96941.59686	96429.66545	103809.8678	106827.3542	95963.28018	90748.84166	92799.05808	85756.95339	100526.9823	106186.1925	114972.8381
Q62376	Snrnp70	U1 small nuclear ribonucleoprotein 70 kDa	266.861	19.42	51992	7	89990.88482	93363.89787	45126.0847	85021.38577	79577.44086	69445.2299	69783.78943	74105.32134	93569.36186	69646.52869	102533.441	82397.55333	77881.53292	80197.14526	68729.18056
Q62383	Supt6h	Transcription elongation factor SPT6	29.284	1.45	199086	2	6935.366246	7240.205749	9182.949287	8537.937521	6686.511603	7030.66185	7458.558619	6990.147734	7192.593759	8306.318705	7453.957872	6382.936297	6804.643172	7321.883566	7018.120953
Q62393	Tpd52	Tumor protein D52	285.218	43.3	24313	7	278521.2811	200706.7926	235593.0368	206765.7139	186808.1062	231159.5035	139847.2347	151412.615	215446.873	134880.3325	252491.6901	195185.907	212566.757	220449.2244	154988.3415
Q62418	Dbnl	Drebrin-like protein	465.119	31.19	48700	10	68032.00709	90873.82125	67176.14071	79257.04448	70588.72429	60335.67843	66542.7163	57285.5378	61057.40837	63024.52431	82297.06267	72192.21778	82217.31387	70696.64455	72171.55332
Q62422	Ostf1	Osteoclast-stimulating factor 1	159.747	29.77	23783	4	101318.8941	100946.0817	82444.12775	88449.64519	83408.0796	74592.66785	88789.90629	75783.82116	83519.24648	83133.7241	96213.99933	93732.06306	102038.7227	84938.11895	90756.8768
Q62425	Ndufa4	Cytochrome c oxidase subunit NDUFA4	115.803	43.9	9327	2	18425.9135	20137.32721	18258.38059	23383.164	16931.45171	26802.81892	23185.22947	20235.77244	19736.34863	29581.84405	19284.43245	22417.75121	20256.95021	22594.71544	22362.2833
Q62426	Cstb	Cystatin-B	99.577	48.98	11046	4	172132.439	156489.2572	140457.5723	137756.7633	134216.0992	131107.8286	160286.2191	135408.1135	135991.5716	127950.3188	164208.1144	157440.2856	142957.1255	143785.7903	133937.7983
Q62433	Ndrg1	Protein NDRG1	888.864	60.91	43009	18	608196.6054	636964.9795	541317.1026	529106.0335	554424.1645	324178.4519	346213.9089	351830.1233	344264.864	382789.1244	563854.8816	503472.3952	514262.4602	494222.2466	488734.0166
Q62446	Fkbp3	Peptidyl-prolyl cis-trans isomerase FKBP3	119.767	29.02	25148	6	71743.57329	61888.26537	66736.11683	58010.92646	71518.86487	55738.96083	59254.02947	58913.769	53916.12067	68958.48775	68795.82826	76848.23174	79021.16874	66514.63509	68670.8825
Q62448	Eif4g2	Eukaryotic translation initiation factor 4 gamma 2	292.198	18.21	102106	11	53911.10351	49223.52938	55711.99702	106026.3221	49805.87103	56314.26071	59316.8585	70008.09542	56558.39488	53566.45899	101821.1558	54184.79149	57309.97408	59325.06993	101326.4806
Q62465	Vat1	Synaptic vesicle membrane protein VAT-1 homolog	373.432	35.47	43097	9	123415.1763	114876.3313	106926.2405	96393.83858	100067.8026	72146.53561	78832.82534	79335.95193	78061.89354	88503.15804	108801.1201	111441.3186	106380.8125	98849.15101	110589.3251
Q62523	Zyx	Zyxin	273.581	18.62	60546	8	104589.323	85627.71297	94154.22168	91241.8794	85553.21052	71293.24567	80475.02421	75264.83931	74590.48148	83313.63986	97274.09918	85151.47323	90773.29032	79696.18621	88271.52681
Q62825	Exoc3	Exocyst complex component 3	54.781	3.44	86497	2	4536.965347	1956.255374	1518.567538	1427.779196	2566.361152	3796.019717	3036.609005	0.01388636	2053.614439	1487.27799	4063.706542	3605.234017	1061.800477	2182.995431	1208.797571
Q62991	Scfd1	Sec1 family domain-containing protein 1	85.027	6.28	72263	3	12992.23803	12632.92658	10807.01315	14095.75472	10493.47692	10322.88043	12507.2593	10844.8354	9695.279176	12466.02326	11438.78688	12188.3791	9228.754737	12601.5418	13546.94927

Q63009	Prmt1	Protein arginine N-methyltransferase 1	314.173	31.44	40522	9	71627.79584	78610.434	74711.71372	84249.05962	84772.52494	70146.54985	86731.59471	88115.38056	83861.883	61181.53658	68124.3303	67724.96861	78556.6341	81365.32472	77937.39455
Q63016	Slc7a5	Large neutral amino acids transporter small subunit 1	59.457	5.86	55903	2	20854.17755	21500.40537	19315.68608	22876.83196	19904.7256	19639.83399	23974.23214	21599.44419	23270.27107	20571.35533	24493.46807	23139.36228	19832.87366	20545.59284	23312.4397
Q63060	Gk	Glycerol kinase	23.058	3.82	57477	2	6728.615817	5371.332878	6321.485211	5442.571965	4638.022012	9941.046246	8331.49232	7615.499106	9541.515057	6588.192351	5095.873772	5901.478668	5675.382469	6932.859211	5439.405224
Q63065	Pdk1	[Pyruvate dehydrogenase (acetyl-transferring)] kinase isozyme 1, mitochondrial	95.595	8.99	49081	2	10649.61835	9585.664402	8232.235088	8248.498637	7666.115573	4158.382634	8023.813108	8180.736427	7322.89043	8150.63982	7724.684731	9671.629973	2469.488501	6668.46232	8714.327744
Q63413	Ddx39b	Spliceosome RNA helicase Ddx39b	694.132	65.65	49035	21	696227.2779	756146.7755	739869.1536	773748.7081	764758.6658	721478.0639	725018.4911	760274.1429	718099.117	721519.7992	694477.1951	778614.7337	749637.9952	811559.2637	772628.4958
Q63450	Camk1	Calcium/calmodulin-dependent protein kinase type 1	124.792	9.89	41638	3	28301.8594	35319.35884	32375.20639	34228.59476	31081.24558	27587.3974	32259.88538	26920.46456	30750.64277	34214.01897	31269.50191	35823.02195	33172.92997	34957.18443	35438.27652
Q63486	Rraga	Ras-related GTP-binding protein A	293.389	29.39	36566	8	59846.37155	53215.5547	51729.68104	56236.7031	58168.15535	63568.46834	62588.4694	58900.89635	64699.84868	57938.47068	57831.00675	60274.92966	49323.69289	60180.94904	54134.57561
Q63584	Tmed10	Transmembrane emp24 domain-containing protein 10	373.401	35.16	24858	7	188908.0921	206503.6716	182630.3664	184568.8367	196630.2775	236979.6543	202671.0722	206906.4097	227937.7842	218246.1476	206611.4971	198816.2158	218110.9726	210004.9431	206951.0112
Q63623	Scaf8	Protein SCAF8	53.926	1.5	139504	2	15230.00278	17942.84084	15293.64242	16166.03507	15541.51964	17444.34063	17169.08531	13756.4318	16031.41304	13742.01891	14873.13911	18627.15111	16790.79012	17605.60031	17850.50834
Q63635	Stx6	Syntaxin-6	49.479	14.12	29057	2	4634.804196	2690.95495	10050.26265	12254.43097	11126.06621	10262.56032	10732.41003	9798.833222	11341.17622	9337.344462	8884.667828	11023.32951	10605.67283	11173.87802	11023.36157
Q63799	Tcea2	Transcription elongation factor A protein 2	53.729	7.69	33528	2	7685.06509	7746.647041	7987.79217	6502.063713	7718.017976	6086.037908	5556.374973	5146.444116	5968.73849	5293.313672	6585.29142	6419.584417	5870.739226	6804.142495	7197.578288
Q63862	Myh11	Myosin-11	645.565	10.78	152492	15	688474.6479	682263.3656	609168.3476	639019.4728	545255.8722	589882.9339	595148.8975	587855.7152	575558.1201	603917.7871	623061.0901	622299.61	651093.5624	645223.6117	639132.9111
Q63921	Ptgs1	Prostaglandin G/H synthase 1	208.36	22.43	69032	9	263099.6006	274859.9993	236614.5161	260442.1842	276725.5249	225541.7176	183410.5398	162947.1113	199945.4826	233370.5671	240157.6212	229092.1261	249878.5272	253413.9675	242860.3603
Q63945	Set	Protein SET	264.432	39.79	33406	8	248645.0081	257444.7239	246451.7269	254163.2798	251661.9031	195697.7033	236385.3208	237693.1353	225784.8871	206978.3217	249730.5929	258510.1241	237863.5286	232753.2083	250260.1428
Q64010	Crk	Adapter molecule crk	160.26	28.29	33815	6	45661.71387	37763.71309	39305.07373	27017.40183	32563.13224	28287.46693	36554.19073	28270.41485	31515.17981	27368.91645	43068.16999	30235.49952	37553.14024	20322.25774	38960.15511
Q64012	Raly	RNA-binding protein Raly	231.157	32.05	33188	10	253990.5137	256020.8118	265231.1511	254973.7486	271011.568	239767.7772	224569.387	206682.145	233665.3096	226204.3598	253711.5436	227017.6924	252606.4436	265403.7361	246405.9228
Q640N3	Arhgap30	Rho GTPase-activating protein 30	48.964	5.18	120114	3	11317.62351	12307.44209	13121.87921	12628.7874	11712.91326	7789.038741	9942.080643	8816.06001	9077.034196	8336.601351	9761.987199	9067.679335	10209.34164	9831.500967	11989.71907
Q64105	Spr	Sepiapterin reductase	408.648	41.76	27883	8	162605.2404	146218.2262	155962.5228	157389.0789	132829.2782	134055.8165	143211.7	123104.5213	123884.3321	124676.5099	159719.499	135106.9607	149801.367	139574.4851	145522.1738
Q64152	Btf3	Transcription factor BTF3	173.982	33.82	22031	3	36421.8241	38136.17341	39040.43175	33368.65477	28589.15937	30859.04129	39926.47256	37716.0329	36798.51083	35631.28353	35990.32898	40692.74864	35074.77181	34215.91142	32769.44355
Q641W4	Rfc2	Replication factor C subunit 2	89.044	8.6	38657	2	6150.815504	4901.591082	8071.843198	7782.7086	7336.150254	7684.373995	7741.894357	8360.701077	8132.133761	8933.609597	5348.080265	5720.919666	1028.887204	7168.202729	7854.148351
Q641Z4	Cdk9	Cyclin-dependent kinase 9	44.543	5.38	42762	2	4836.018302	0.007562416	4389.680424	0.009171104	0.008265859	4221.658024	3949.897889	0.01388636	4501.78475	3698.937249	0.008046421	4380.844726	3723.534971	0.00945634	4459.947597
Q641Z6	Ehd1	EH domain-containing protein 1	626.153	65.73	60603	27	355666.439	384572.8039	341315.512	351856.6638	341470.3448	416877.0012	396332.9324	404625.7012	415517.4307	416172.2568	367587.2919	366422.6466	388908.1713	397405.8587	377626.2563
Q64213	Sf1	Splicing factor 1	165.52	11.64	70403	6	29665.71793	32914.99464	25388.99754	32936.58573	33227.96767	31124.85063	37700.48414	35436.79875	19771.77259	33437.7984	41452.54121	40274.52524	42994.72174	29071.09293	36333.70661
Q64232	Tecr	Very-long-chain enoyl-CoA reductase	127.618	13.96	36123	6	78218.76039	88605.20241	83298.80305	78844.06052	87981.43057	104653.2742	105787.8834	104658.7978	117978.8909	97728.05349	82387.99528	84532.28052	91290.51811	95491.91405	94169.51516
Q64261	Cdk6	Cyclin-dependent kinase 6	117.992	20.25	37028	5	31105.36363	19578.09035	29928.91088	24506.91997	18398.3658	13938.58875	17333.74758	27937.38523	18361.66098	28546.60246	32959.57214	30947.71378	21284.75107	28659.96675	14944.64329
Q642C0	Dnajc8	DnaJ homolog subfamily C member 8	90.595	27.67	29813	4	39552.89073	38291.37308	32628.03468	38360.82122	38606.62821	29574.46455	33114.91533	30638.2839	32610.8681	32214.10946	36249.09383	37527.14235	38263.63183	37272.42733	31840.01835
Q64310	Surf4	Surfeit locus protein 4	211.051	17.47	30381	3	24714.39158	21453.11759	13557.05779	20079.84253	16822.68644	20245.70561	18109.27806	25297.72368	25186.9953	21424.66982	18287.57947	14802.77459	18724.27189	19010.11497	18440.47709
Q64324	Stxbp2	Syntaxin-binding protein 2	691.24	39.8	66357	14	92792.34686	97136.28038	84808.96844	90742.77877	78115.14429	62487.60146	83848.899	82458.11935	81647.81381	74028.8502	82316.70399	82126.01063	75676.99616	82724.03996	79202.60345
Q64337	Sqstm1	Sequestosome-1	89.348	10.41	48163	3	5742.817529	3190.638732	3388.620151	3827.488156	2423.206559	7753.686964	2213.587467	2311.426202	10244.96531	8206.58547	4584.641405	4700.193058	3897.939665	3887.601686	4134.078201
Q64378	Fkbp5	Peptidyl-prolyl cis-trans isomerase FKBP5	145.766	17.11	50966	6	75631.62729	70738.14832	77010.20371	78313.75979	89361.10988	95330.07858	89037.40675	93830.08964	88277.18882	96701.14034	73044.81322	77768.71085	83317.65918	74613.77247	76638.73186
Q64430	Atp7a	Copper-transporting ATPase 1	60.924	2.88	161959	3	7847.000501	9103.853444	6772.947763	8462.21745	9339.741766	11909.06989	10081.85456	10439.8684	10677.47426	9967.13289	8344.129913	9226.269064	9266.825571	11146.22768	8409.601368
Q64433	Hspe1	10 kDa heat shock protein, mitochondrial	142.008	52.94	10963	6	166287.7265	145896.7479	151341.1879	131598.6148	118312.1899	200074.939	160701.7453	142736.4037	149559.4871	137140.635	169317.2054	143354.0116	131412.3081	129106.4968	127529.3419
Q64511	Top2b	DNA topoisomerase 2-beta	299.406	7.38	181909	10	102697.5127	115526.5553	114637.281	109581.8673	118834.9641	110370.3234	107750.5128	110835.4019	109756.412	104965.4039	100611.4086	110820.1696	121098.443	123427.5961	108883.2131
Q64514	Tpp2	Tripeptidyl-peptidase 2	740.218	25.91	139879	24	322756.5567	347724.5401	341243.9214	336484.0965	358331.6059	306846.4294	320568.1534	305625.5672	300229.4255	301009.3041	319326.8872	320580.9934	317114.8233	313950.352	319465.0737
Q64518	Atp2a3	Sarcoplasmic/endoplasmic reticulum calcium ATPase 3	552.793	17.44	113638	15	131242.4439	112715.0534	122867.525	128450.8994	146868.1866	168283.4349	138342.8448	145436.7174	165068.4901	129695.916	116732.3645	120054.2606	127832.5396	143759.8043	118181.7388
Q64521	Gpd2	Glycerol-3-phosphate dehydrogenase, mitochondrial	442.911	29.99	80954	16	134293.1392	116542.2331	110080.5185	136704.5075	102245.3439	86808.22217	116427.3534	92013.72604	98204.01137	110673.377	132626.0815	100878.814	131390.6539	142057.1809	115372.17
Q64522	Hist2h2ab	Histone H2A type 2-B	380.051	57.69	14013	7	9283696.099	8303301.101	9099625.245	8061387.194	9204061.708	10390782.76	9578281.049	9581384.566	9368670.182	10082234.05	8795115.492	8365718.222	9226611.562	8708137.685	9691603.188
Q64559	Acot7	Cytosolic acyl coenzyme A thioester hydrolase	85.904	5.77	42735	2	33934.89393	30539.40165	31983.58799	28592.38985	26293.33358	27429.78234	36593.31378	39618.95017	39546.62947	33071.99277	30909.81482	33174.93496	31661.09638	29432.86884	28691.941
Q64578	Atp2a1	Sarcoplasmic/endoplasmic reticulum calcium ATPase 1	254.77	9.76	109409	6	44702.04605	42300.22856	48502.60467	43868.91499	43248.17664	50782.5613	50868.29184	52942.87742	56744.66633	45355.30303	40699.51691	41603.49131	41074.67694	46479.55457	43612.13241
Q64620	Ppp6c	Serine/threonine-protein phosphatase 6 catalytic subunit	117.876	19.02	35159	5	35071.28195	35863.48599	36972.88059	37453.48037	37558.71568	38644.60589	35414.17034	38038.03327	37933.50638	34991.30516	29963.02869	33776.6247	34227.18533	33641.9778	30218.2065
Q64674	Srm	Spermidine synthase	162.906	29.8	33995	6	52698.64451	48604.19401	53591.79421	51065.70012	47290.33872	51064.22266	55646.22518	44896.35002	51285.61568	47384.5003	50893.18554	53892.36298	49412.49269	52621.43768	51640.2276
Q64737	Gart	Trifunctional purine biosynthetic protein adenosine-3	506.588	17.62	107503	11	128269.6108	128855.0105	134720.2296	221632.5681	128423.6333	110578.3806	210283.0668	154464.2397	202948.8809	107092.9707	192440.6879	122600.4093	119702.9307	197234.3078	122152.7001
Q65Z40	Wapl	Wings apart-like protein homolog	138.02	2.83	134070	2	3509.303238	2835.047101	2804.926951	3219.959309	2602.958738	2096.463218	2893.842739	3062.069404	2719.666614	2509.267599	2678.542386	3040.839119	2719.928556	2174.261745	2048.1302
Q66H50	Far1	Fatty acyl-CoA reductase 1	225.411	18.45	59268	6	47447.95565	43618.08537	47603.67972	48082.64734	47935.62539	57806.40399	62141.34968	59432.0496	63036.46172	51369.52393	44113.58513	50875.89414	50977.2549	50212.81342	50591.90884
Q66HA6	Arl8b	ADP-ribosylation factor-like protein 8B	360.396	53.76	21539	11	287347.7441	290888.3579	239737.1165	264059.0474	249812.4337	208883.7747	221255.8962	214764.5062	232531.477	225776.5687	263929.1945	277541.3174	277312.4397	281164.1256	266717.4367
Q68FD5	Cltc	Clathrin heavy chain 1	4079.117	65.73	191557	96	3165989.004	3160175.022	3002172.92	2980610.452	2990805.726	2544882.961	2798147.449	2741860.813	2806487.646	2575927.875	2725550.631	2888478.867	2646578.03	2955599.076	2880798.896
Q68FF6	Git1	ARF GTPase-activating protein GIT1	102.24	5.32	85300	3	9260.807284	5614.367668	8328.352442	8654.968828	4499.371527	5468.989685	8006.035936	6234.19043	3859.694716	6551.345384	7489.546668	5680.779468	4057.018018	6713.665988	8535.919401
Q68FH4	Galk2	N-acetylgalactosamine kinase	137.369	16.38	50503	4	25609.91044	20144.05965	18545.62646	24265.49754	22734.13738	18765.91207	14076.97954	18385.40114	13204.73577	16598.16669	22415.10371	17198.61516	21158.12023	20303.7375	19457.42709
Q68FL6	Mars	Methionine--tRNA ligase, cytoplasmic	594.832	28.49	101431	15	149736.7347	152822.2116	127426.0278	139748.9287	131852.0719	108310.3173	133260.3487	136450.0349	119624.4669	103385.643	121142.294	129692.8172	117897.1468	112691.6152	122055.4261
Q68FX0	Idh3B	Isocitrate dehydrogenase [NAD] subunit beta, mitochondrial	244.908	23.64	42354	9	133338.3249	124174.5256	108974.0111	119454.8263	137507.3494	115246.6086	137564.1254	147745.5052	132960.9011	131551.3914	120107.7093	132180.5744	140609.9675	112329.8345	129093.2882
Q69ZK0	Prex1	Phosphatidylinositol 3,4,5-trisphosphate-dependent Rac exchanger 1 protein	593.73	16.48	184935	20	157238.7576	170047.7006	160722.3506	155749.9092	141292.5837	157126.7851	162070.5613	161704.046	163363.7417	166201.4467	148285.1487	145920.4211	160882.8419	156710.9422	156048.3675

Q69ZN7	Myof	Myoferlin	429.761	10.11	233324	17	116379.3315	118919.1987	104975.9181	105591.7402	107190.0386	106207.7315	113446.1724	116256.9005	112750.8686	110534.0612	130343.5452	121562.2052	123393.0109	113363.072	127315.3164
Q69ZR2	Hectd1	E3 ubiquitin-protein ligase HECTD1	75.525	2.41	290086	4	14073.97953	11489.89107	13601.31338	15416.74673	13961.38291	15708.5812	11202.22773	12244.52747	12894.7395	14596.97012	13660.93776	12019.52987	10540.59991	11870.42845	16095.79566
Q6A009	Ltn1	E3 ubiquitin-protein ligase listerin	124.786	3	198921	3	4073.234978	4835.284767	4521.018328	5049.276064	4289.771042	3957.638021	4685.60843	4752.253764	3203.206179	4638.292215	2845.762582	2389.471398	4276.355185	4549.293967	2677.186052
Q6A028	Swap70	Switch-associated protein 70	515.661	32.82	68996	15	195692.4824	203179.2639	189178.1729	200451.1522	239653.3294	181318.9031	197511.2129	200468.4018	181099.3261	182041.5066	173983.5502	193678.8863	178198.3264	186725.1372	191339.2516
Q6A065	Cep170	Centrosomal protein of 170 kDa	93.838	2.96	175050	4	63088.64177	76492.03499	56784.02624	77747.55419	89207.74513	79565.96989	70153.30517	62923.372	79642.37638	81996.51314	73566.81673	71949.55837	77619.54384	87712.14563	86614.96353
Q6A0A9	FAM120A	Constitutive coactivator of PPAR-gamma-like protein 1	181.776	5.85	121646	4	22661.25664	18112.77588	27174.1612	26411.80863	28409.01324	25597.36232	26466.07703	27906.62521	25628.17486	17593.44273	26758.57779	25840.90915	26717.15628	26696.52095	27068.36529
Q6A0D4	Rftn1	Raftlin	82.367	11.91	61537	4	13083.27066	11771.33866	11162.65077	11826.01204	11105.17942	9049.506602	8823.247147	9092.298721	9228.745649	9352.770904	10645.84932	8707.397986	12705.65729	8749.945742	10628.85048
Q6AXS3	Dek	Protein DEK	232.107	21.16	42892	7	58451.03289	60049.67952	55544.18026	56039.80867	64094.99939	41552.14412	45562.27601	22065.27427	46173.47301	43144.80469	68663.83477	61863.20219	54991.33343	43429.22783	61993.8219
Q6AXS5	Serbp1	Plasminogen activator inhibitor 1 RNA-binding protein	383.707	37.59	44754	13	347869.979	344636.0798	228350.588	248817.1317	413920.7637	382447.0761	302743.378	396978.1497	301309.1002	207455.7572	370665.08	338348.8216	417871.75	247830.2448	282291.6709
Q6AXT7	Rbm42	RNA-binding protein 42	56.837	5.7	49905	2	6824.992477	5997.441382	7719.1768	7547.28582	5038.796315	6990.208057	5508.561681	7932.526499	6719.025405	6853.651156	7748.255192	6584.510173	6750.244626	7447.837897	6855.211845
Q6AXY7	Prpf38b	Pre-mRNA-splicing factor 38B	55.077	3.32	63932	2	4497.297423	10133.29863	4346.090243	4583.147843	4458.10993	3104.011559	3164.276236	4204.082292	4183.648023	3618.641497	7623.090621	7858.499867	8748.179371	5026.748998	4210.387539
Q6AY23	Pycr2	Pyrroline-5-carboxylate reductase 2	364.091	41.56	33673	9	105655.6289	97587.23479	98042.50359	82914.90248	96902.04472	116737.476	104371.8592	97990.70679	118560.6697	102216.8499	100922.5878	92049.96741	100348.5487	100860.3537	98036.75824
Q6AYE2	Sh3glb1	Endophilin-B1	166.687	16.16	40787	5	76146.5712	61159.53965	56685.28477	63403.17315	54829.95102	50822.44034	48558.09918	44518.67576	51390.07656	54367.70486	63263.05295	52227.41253	60507.06111	58592.17051	60739.51216
Q6AYH5	Dctn2	Dynactin subunit 2	233.446	21.14	44148	7	92783.45419	87529.93277	85457.20245	87510.21236	86160.62716	104773.104	83014.71018	81203.06323	96844.70284	77871.03147	91258.82829	81970.40863	91797.6792	89282.10604	88451.77734
Q6AYK3	Isyna1	Inositol-3-phosphate synthase 1	209.782	17.59	60884	7	76605.23053	80361.34499	74439.91552	74765.23051	71491.85204	56019.35359	73558.8044	72137.10346	68585.62424	65717.68628	73062.03256	81632.15996	69254.69226	70574.18968	67850.41002
Q6AYL5	Sf3b4	Splicing factor 3B subunit 4	206.282	18.4	44356	4	22973.13346	24121.6313	18370.02108	24107.73392	18362.86394	19941.65115	19537.90544	20987.36818	19708.62632	16660.94806	23403.29874	25243.7027	21174.23332	19526.19471	18095.41823
Q6AYP5	Cadm1	Cell adhesion molecule 1	48.06	8.19	51854	2	3549.034381	10114.78772	7361.784592	10201.17449	9997.110029	9621.695909	10131.98474	10940.82049	9852.637317	9382.120271	4512.319691	9477.900729	9135.7519	8174.069412	9732.238679
Q6AYT3	Rtcb	tRNA-splicing ligase RtcB homolog	185.449	21.58	55249	6	56855.41175	60818.63729	51469.5848	51679.92562	54735.25734	54802.30695	51171.96861	53302.06894	46233.29182	51538.57375	53901.77851	60200.30772	57912.79327	50678.56179	50957.22231
Q6AYY8	Slc33a1	Acetyl-coenzyme A transporter 1	93.788	7.82	61101	3	1672.888262	1466.834169	9345.181918	1784.993407	1149.478489	4712.934219	11675.03617	9576.40582	1738.336641	6975.672393	1505.987884	8556.165477	5975.89617	9526.659311	6984.098018
Q6BBI8	Ufc1	Ubiquitin-fold modifier-conjugating enzyme 1	74.507	20.36	19492	2	14542.4138	14332.43015	12378.49491	12040.43588	13741.56686	11923.69511	13892.85144	11649.11162	12252.62077	11122.92051	12702.13027	13547.43304	14506.00559	13809.0088	13532.45763
Q6DFV1	Ncapg2	Condensin-2 complex subunit G2	52.338	2.02	130894	2	5612.2646	2618.472485	5364.683523	7498.821673	6244.437968	4141.12571	5540.971795	5221.210844	6162.868673	6396.690888	6290.310114	6976.722583	6315.77088	6251.969095	6624.614198
Q6DFW4	Nop58	Nucleolar protein 58	431.638	23.51	60343	9	114975.9023	107995.206	104111.2384	106375.0533	115293.4321	91931.93844	95285.41976	91936.747	99588.81584	87888.21829	96747.37243	106371.6098	94762.37872	102169.1395	97611.59782
Q6DIC0	Smarca2	Probable global transcription activator SNF2L2	202.857	5.2	180253	6	26216.75281	29902.81073	44002.81605	44629.08026	24977.1092	46042.01964	26297.96799	42433.70483	47425.04908	24949.42271	28101.29007	47523.63565	29096.71955	30098.57151	29821.12281
Q6DVA0	Lemd2	LEM domain-containing protein 2	35.137	4.5	57507	2	4802.982902	4834.416488	3814.431461	3404.114474	6236.116852	6978.263612	6270.817181	6918.967158	5367.613373	6246.541212	6182.947766	4279.587359	5729.85524	5480.635568	5970.536542
Q6GQT9	Nomo1	Nodal modulator 1	253.918	11.2	133420	9	59972.09874	53196.51632	48823.07938	52068.39981	53608.09175	80056.01929	73493.75264	77073.95423	76007.26464	70330.52694	48317.09574	68065.5631	67338.69474	67950.32021	52651.4198
Q6IMY8	Hnrnpu	Heterogeneous nuclear ribonucleoprotein U	1237.314	46.87	87732	34	2193168.122	2242029.028	2325469.408	2462415.804	2240943.403	2007178.846	2165776.163	1981928.788	2056403.768	2054034.001	2022374.555	2184962.196	2026458.215	2160690.696	2141007.958
Q6IRU2	Tpm4	Tropomyosin alpha-4 chain	577.614	50.81	28468	16	341165.1538	325539.1046	291346.2403	298261.5778	344361.7422	338753.4376	333006.1045	332059.7522	329319.4302	327443.7212	322151.889	333802.5623	346749.1419	310268.6964	332732.8097
Q6KCD5	Nipbl	Nipped-B-like protein	110.161	1.29	315450	3	3989.631514	4156.853383	3541.303065	3832.596186	3570.96108	3774.613498	3607.274039	4267.170662	4214.898319	3517.247835	3905.285108	4095.564265	4096.210415	3704.688891	4073.526124
Q6MG14	Nrm	Nurim	51.085	14.5	29382	3	11954.96707	15436.43319	13885.59572	12087.34149	14432.12564	15197.14557	13862.42017	13995.90207	12927.00668	16570.44405	12984.69644	11697.90561	14819.19768	13681.19337	13153.45417
Q6MGA9	Brd2	Bromodomain-containing protein 2	38.5	4.51	88051	2	21559.06344	21241.53065	22108.36844	19241.53946	16449.74742	16495.0862	20975.73552	17668.41716	15423.70931	16356.77993	15710.32093	15508.41788	16644.55328	16421.52002	20385.77741
Q6NS46	Pdcd11	Protein RRP5 homolog	162.864	4.51	207779	7	23624.29726	23503.37167	25210.86014	25041.40042	21017.47967	25210.38374	26963.35626	25517.10733	26754.48245	26088.98968	24828.7584	25625.73369	23966.95774	25898.07728	26213.70862
Q6NSR8	Npepl1	Probable aminopeptidase NPEPL1	194.656	18.89	55940	7	93970.01257	91388.973	82082.29441	74551.59883	80957.40948	57416.25391	65232.66051	58984.96437	62680.92015	67530.67028	76591.55484	76358.7919	83513.1453	82616.02965	74408.40353
Q6NV83	U2surp	U2 snRNP-associated SURP motif-containing protein	168.363	5.05	118261	4	12448.78061	13634.71695	13567.25361	14626.11162	13677.93524	13364.80639	14473.12013	13724.5826	11812.20778	14003.63238	13243.68659	13817.8685	9832.035665	12721.62904	11707.36664
Q6NVF9	Cpsf6	Cleavage and polyadenylation specificity factor subunit 6	174.093	9.98	59153	3	36444.11747	37307.97167	36907.37628	46516.64471	38554.68555	41836.67345	55927.97414	48521.0752	45796.55291	38730.48143	38431.52054	49391.22645	45155.30749	48570.05338	45384.08483
Q6NWW9	Fndc3b	Fibronectin type III domain-containing protein 3B	80.396	3.31	132764	3	1829.547261	2029.734906	1351.132222	1751.092698	1488.20318	1064.874263	1589.332462	1618.890331	1481.548901	1063.205864	1341.58301	1295.948912	1336.376455	1171.661896	1205.386093
Q6NZJ6	Eif4g1	Eukaryotic translation initiation factor 4 gamma 1	725.573	13.88	176077	16	173169.8128	172092.4114	146391.0856	182152.5077	146214.9027	138768.3255	173409.3995	181065.8804	137354.7468	139868.8358	146485.2126	179328.904	158148.6461	129628.6664	147007.9405
Q6P1B1	Xpnpep1	Xaa-Pro aminopeptidase 1	246.517	25.2	69591	12	176433.2494	205061.6853	204064.2369	197525.24	195769.8678	186323.6184	200786.7445	212414.5731	221046.0728	173488.0418	198499.514	212895.2984	206383.277	188475.8839	187658.5892
Q6P2B1	Tnpo3	Transportin-3	168.112	9.97	104170	5	20770.40673	17871.09998	19682.39801	21926.30897	18533.03938	15669.18198	21083.47287	20831.12233	17469.58984	16947.40368	18765.46647	13983.62697	16022.54261	19784.10455	21214.87493
Q6P3A8	Bckdhb	2-oxoisovalerate dehydrogenase subunit beta, mitochondrial	28.169	9.49	42880	2	30313.85444	27061.00238	31866.53517	23046.35064	18503.52819	14706.5382	18248.39134	12306.96839	12666.6686	15730.15172	27707.04562	20851.94496	18736.12429	21393.28986	17928.00296
Q6P3D0	Nudt16	U8 snoRNA-decapping enzyme	43.214	20	21825	3	18655.58455	16991.77443	16108.00281	17222.03034	16153.1436	16561.06963	14011.94167	13476.30491	16238.06917	15811.40819	18708.80357	15865.9334	16374.79587	17098.8067	17494.84819
Q6P4S8	Ints1	Integrator complex subunit 1	109.81	3.28	245168	4	6428.381313	5023.941515	6055.608745	5901.003933	5359.343225	3703.649971	5717.766442	4816.039854	5047.207688	4530.00409	5618.302734	5815.298664	5157.584432	5836.267224	3736.363822
Q6P4Z9	Cops8	COP9 signalosome complex subunit 8	48.672	19.14	23236	2	0.008226335	0.007562416	2922.258839	3350.679039	1532.265788	1198.325995	0.012348474	1826.299705	2570.648064	1185.364166	3722.379662	2639.639199	2707.467542	0.00945634	1736.949285
Q6P542	Abcf1	ATP-binding cassette sub-family F member 1	340.247	20.19	94945	8	56915.41053	58981.39377	50079.08747	50625.49173	48813.06698	48667.115	51446.97839	56185.92907	59330.3903	52442.26851	52571.58042	54299.16983	50537.95076	51611.58099	53399.20877
Q6P549	Inppl1	Phosphatidylinositol 3,4,5-trisphosphate 5-phosphatase 2	207.803	7.4	138973	6	37623.1611	38251.00491	37872.99666	38411.85841	35413.43598	35457.19833	39768.63746	42420.93285	35395.11305	39779.21305	42129.0883	43654.00503	38448.14124	42042.84333	40555.41839
Q6P5D8	Smchd1	Structural maintenance of chromosomes flexible hinge domain-containing protein 1	344.122	5.98	225648	10	47221.74377	52945.22859	41717.32611	49075.15335	52375.03638	48154.74337	53017.85865	47771.82973	51555.43166	43472.21822	47805.52441	48257.41501	38743.14386	49281.09919	52799.60484
Q6P5E4	Uggt1	UDP-glucose:glycoprotein glucosyltransferase 1	1074.363	39.72	176434	37	447522.9497	451805.7952	361406.2397	443994.0464	398176.0878	411516.7879	446536.8887	452205.618	471472.6954	438010.2237	393025.5687	441287.0978	418369.3546	419448.9268	410785.3085
Q6P5F9	Xpo1	Exportin-1	972.08	36.79	123093	28	361309.9682	367385.8832	349655.9759	359370.659	339549.0616	294231.4667	315133.6393	328625.7944	327270.2087	293742.1298	329409.4388	353042.6499	343651.7406	348317.8485	333746.5972
Q6P5H2	Nes	Nestin	1914.467	37.93	207124	50	830531.5473	846787.0467	732291.347	759463.2835	766415.8714	750174.7426	777333.5919	786574.5191	689747.0446	745226.6949	765245.7885	707921.6479	844088.6008	810011.2987	826571.2068
Q6P7P5	Bzw1	Basic leucine zipper and W2 domain-containing protein 1	288.035	27.92	48043	10	99947.20202	83438.96837	102096.3246	116067.791	93250.18824	81781.50091	99574.11273	75051.82255	110005.2331	105439.6958	94118.57454	93541.39174	91678.32121	121437.0366	95816.23086
Q6P7Q1	Babam2	BRISC and BRCA1-A complex member 2	93	9.92	43559	3	16910.56083	19015.50145	12038.765	18504.43607	12275.07743	14401.81396	16032.34807	19236.83981	13745.71354	12158.51509	15240.64732	16918.74554	16013.7318	16773.83605	17441.72774
Q6P7S1	Asah1	Acid ceramidase	149.889	12.44	44443	5	118327.0234	100893.3717	91026.94238	101262.2547	89055.91784	83518.9197	78204.93013	78580.61729	85670.14976	91074.0074	92807.18496	80828.73557	99052.83993	105080.406	97295.52223
Q6P8X1	Snx6	Sorting nexin-6	162.194	18.47	46649	8	77091.5797	79094.70086	57909.91964	76006.39724	74493.0697	37145.19634	30220.59353	55737.13928	43780.50324	64455.71845	52994.14626	49936.34971	79268.26874	85679.26902	75283.80176
Q6P9Q4	Fhod1	FH1/FH2 domain-containing protein 1	74.217	2.01	129600	2	4245.160245	4802.860418	4410.950543	4949.352901	4190.976711	5673.957674	4632.397311	4955.161666	5431.816576	4664.573743	5309.847213	4591.952938	4837.350661	5031.134943	4989.959368

Q6P9Q6	Fkbp15	FK506-binding protein 15	99.335	3.54	132961	3	12330.48488	22438.3907	10013.93239	13221.81733	10295.07771	9656.802255	12437.74504	10569.4741	7956.167144	9105.408373	11125.64734	10628.23244	19113.1837	20518.15291	21668.43325
Q6P9R2	Oxsr1	Serine/threonine-protein kinase OSR1	280.913	19.17	58214	7	55695.80285	66160.63322	61232.91304	62006.9414	59326.25178	61988.84272	59867.27321	63975.00683	56872.93753	64016.82495	55445.10215	66061.64845	68979.21715	61115.67984	56190.76605
Q6PAR5	Gapvd1	GTPase-activating protein and VPS9 domain-containing protein 1	56.369	2.13	162402	2	9152.436627	10834.97264	9050.971224	15467.51108	7321.49282	7573.611548	6955.607616	9477.903832	8250.858294	6462.350685	17586.9595	7076.743603	12885.28911	9008.443627	7260.486738
Q6PB66	Lrpprc	Leucine-rich PPR motif-containing protein, mitochondrial	863.426	30.24	156615	29	167511.4761	169214.5341	144651.7217	154503.3544	147717.3548	186800.1167	193815.574	192004.9434	189287.4037	166204.2572	169091.0688	177317.2908	172916.5905	150688.2184	173639.5779
Q6PB93	Galnt2	Polypeptide N-acetylgalactosaminyltransferase 2	49.663	5.79	64514	3	21215.47615	22634.64673	29184.37231	23206.02872	12027.84352	37281.93589	25452.59763	31524.9592	29309.38472	27759.0897	21449.21738	20219.93864	25215.86524	23171.8193	21019.23918
Q6PCN7	Hltf	Helicase-like transcription factor	55.908	2.69	113317	2	928.4854096	980.3645741	1045.014018	1045.079771	634.7313815	273.8505065	611.9870421	815.0806323	854.8782958	321.4818453	589.5176857	957.1825284	641.9062151	539.3847411	553.8524884
Q6PCT3	Tpd52l2	Tumor protein D54	38.425	9.55	23992	2	24071.38213	26805.63661	22668.24819	19600.4506	20878.83435	16806.60686	17634.737	15330.42809	17075.68955	18629.11058	18920.80463	16553.24387	20929.19621	20007.35927	20299.13713
Q6PD03	Ppp2r5a	Serine/threonine-protein phosphatase 2A 56 kDa regulatory subunit alpha isoform	92.167	8.64	56347	3	8401.411286	7958.838481	7919.195946	7654.356162	7353.833489	7932.649212	7353.620546	9883.281382	1738.532211	8415.374405	6916.545469	8115.161651	2999.511633	7480.118861	9658.620148
Q6PDG5	Smarcc2	SWI/SNF complex subunit SMARCC2	128.747	5.94	132604	5	31836.36813	31442.99689	31836.20329	33770.42247	34127.5039	26551.90924	29208.97875	26447.16188	27701.7928	25411.35847	24173.23057	25638.57674	28284.94291	28797.41228	31725.04103
Q6PDI5	Ecpas	Proteasome adapter and scaffold protein ECM29	311.537	7.34	203703	9	20958.33324	25994.6942	30562.34033	28407.15648	26492.62344	20652.77093	21118.9948	30498.23649	29779.50399	23207.61513	24741.20932	25819.03158	25516.12198	19937.33744	25940.78934
Q6PDL0	Dync1li2	Cytoplasmic dynein 1 light intermediate chain 2	310.413	28.25	54218	8	48093.80935	50579.68983	46713.70836	47162.67101	44216.24924	43587.82509	52199.54997	47352.11798	46042.70056	51854.02242	46886.07599	40833.22971	46056.31072	50300.76684	46682.56102
Q6PDM2	Srsf1	Serine/arginine-rich splicing factor 1	185.744	32.66	27745	7	179761.2134	161808.0252	167848.2649	159093.7302	161052.5163	157545.5168	130243.9499	114491.0346	145099.2311	114620.6879	157802.2139	159293.3804	166351.2434	156294.6458	145383.1578
Q6PDQ2	Chd4	Chromodomain-helicase-DNA-binding protein 4	322.991	9.19	217751	13	89914.71718	91831.26845	91039.55253	101372.739	90248.96232	80565.24668	97494.73466	85610.73261	85886.20072	89950.83592	104524.1819	97710.38411	83523.68295	86538.77464	95046.50371
Q6PDV7	Rpl10	60S ribosomal protein L10	382.021	42.99	24604	10	685493.1607	755627.6913	685608.4555	684804.2635	721631.117	699145.1065	655287.3987	720584.4834	679675.735	664037.0654	657630.1878	687684.4758	624770.3219	669013.7953	625652.2271
Q6PE01	Snrnp40	U5 small nuclear ribonucleoprotein 40 kDa protein	172.828	19.55	39276	4	32158.29754	34956.05661	37070.20747	35110.66922	34312.15699	29279.06308	30685.10609	32362.1817	30441.18119	27913.62052	29203.58134	35135.19182	32590.50171	31352.13226	31770.70457
Q6PEC4	Skp1	S-phase kinase-associated protein 1	164.247	31.9	18672	4	38951.42222	36034.30206	31511.25892	28249.68175	33647.98283	36521.72611	31810.86399	35102.80056	34447.68187	35524.086	33668.99289	35054.82991	30137.52813	29425.87824	32404.5445
Q6PFD9	Nup98	Nuclear pore complex protein Nup98-Nup96	170.461	6	197241	7	28232.28517	30000.51525	26324.15289	27112.28836	26376.28974	28007.9611	36196.36591	31619.77873	23968.17427	31580.32831	27326.37951	26820.7421	28553.97672	24804.20101	27304.92986
Q6PFQ7	Rasa4	Ras GTPase-activating protein 4	83.052	3.87	90060	3	11777.93222	10687.60479	12628.22772	10098.91897	13216.48024	14977.36923	14278.04203	11814.79067	15943.82359	13316.34237	10704.4172	7766.393673	12468.8472	12942.96649	12934.31193
Q6PFR5	Tra2a	Transformer-2 protein homolog alpha	101.025	8.54	32316	2	11582.48272	9054.539877	11833.33533	10066.51173	8005.694664	11480.43842	10971.43166	10299.43912	10817.99118	8865.749905	9975.963658	11659.78402	11524.37249	10691.34701	10278.8439
Q6PGB6	Naa50	N-alpha-acetyltransferase 50	85.827	21.3	19414	3	25593.18425	20010.13683	25055.266	20505.15246	20168.91899	19302.11443	26301.8485	23784.52187	21626.80056	19059.98865	27675.39905	17209.83245	19180.34281	23923.9269	21469.81629
Q6PGH2	Jpt2	Jupiter microtubule associated homolog 2	33.619	14.74	20020	2	19008.48611	17499.99901	19558.72866	18496.14654	19705.68372	8255.798072	8724.779674	17132.90185	16046.34509	18015.40627	16847.64832	17755.05647	18863.59279	17100.79844	16671.11995
Q6PGL7	Washc2	WASH complex subunit 2	178.8	4.87	145311	3	5737.121326	5348.734981	2372.307302	4788.296857	3616.937358	2155.099103	3350.909344	4241.560744	4016.920302	2642.116284	4499.035533	3999.514267	4756.096615	3240.798695	3214.488604
Q6PHZ2	Camk2d	Calcium/calmodulin-dependent protein kinase type II subunit delta	420.693	32.67	56369	13	255914.4726	259301.9322	240789.503	267652.2307	252148.7787	194219.2674	216849.7384	197604.6735	255805.3369	285060.7779	265020.6915	309214.6206	326169.4689	261714.9505	251112.6808
Q6PIP5	Nudcd1	NudC domain-containing protein 1	65.206	7.04	66705	3	9296.169214	10682.64857	11047.38344	11883.17323	10284.08825	10820.28609	13235.32607	10848.53612	10757.97831	9809.6683	10047.13425	9293.863778	11030.55025	10938.83242	12919.94721
Q6PIU9		Uncharacterized protein FLJ45252 homolog	67.619	8.47	37705	2	9576.675938	10142.28088	8593.863965	2952.406667	7528.955547	7688.642298	8575.508973	8737.990897	8424.512453	8297.968613	8469.368913	4590.262103	8984.759524	8136.160128	8326.771754
Q6Q0N3	Nt5dc2	5'-nucleotidase domain-containing protein 2	130.949	10.49	63653	4	17428.09192	14813.517	13750.37064	16002.72981	12754.10772	8313.397487	11309.37779	10144.40396	11134.01785	11016.8835	14032.85742	13407.55654	14527.58741	12343.42628	12195.58314
Q6Q899	Ddx58	Probable ATP-dependent RNA helicase DDX58	113.886	6.59	105975	4	6910.118595	6678.684094	9886.749131	5284.084977	10449.53872	11209.31209	11924.24844	12360.48121	11950.74454	11485.79237	5214.682193	2598.368259	7740.32856	8280.410679	7627.290814
Q6R5N8	Tlr13	Toll-like receptor 13	196.681	11.81	114443	7	26636.5427	25065.47371	23841.31553	26142.74909	22283.79686	20798.71416	26010.20224	21750.27609	25243.24044	20963.32681	22311.152	24202.97168	23471.49942	25525.4053	24574.65703
Q6RJR6	Rtn3	Reticulon-3	37.993	2.02	101517	2	58146.14022	61069.09316	62737.66728	53943.40902	55520.04692	53257.93671	54127.00476	61754.52934	66446.54455	60087.70948	72439.19131	59759.82636	62212.09963	59504.2061	54577.15188
Q6TEK4	Vkorc1	Vitamin K epoxide reductase complex subunit 1	78.781	19.25	17783	2	9639.036701	7472.003697	7909.155851	9487.075672	8542.548233	8115.537207	8234.752089	8193.07536	7727.856865	6337.057677	5274.072343	6792.851469	6423.977365	7837.010007	6930.694577
Q6TUG0	Dnajb11	DnaJ homolog subfamily B member 11	290.148	30.17	40495	7	41934.51763	50756.5407	48985.59436	44220.58096	47946.26355	64048.03454	54344.86889	56014.68595	64336.09644	58726.37971	53033.08691	53933.3421	52965.35779	52758.78156	54670.38527
Q6UPE1	Etfdh	Electron transfer flavoprotein-ubiquinone oxidoreductase, mitochondrial	221.252	16.88	68198	7	53084.83394	45906.35514	43276.19178	48449.60612	42632.15937	43067.20576	27966.21597	24866.25019	46612.89746	38524.00188	34722.81654	30445.31994	37334.36141	52343.17543	47125.42739
Q6URK4	Hnrnpa3	Heterogeneous nuclear ribonucleoprotein A3	923.97	41.42	39652	20	849642.9038	842987.6286	796204.1548	851745.2761	864088.3685	659012.8174	706485.3578	718629.7284	698403.3897	727671.7541	803190.2632	799371.2796	794421.3894	832896.624	758724.8995
Q6URW6	Myh14	Myosin-14	274.465	4.45	228586	9	562380.7876	564578.3229	498433.0047	528397.6941	425968.5506	484064.0185	486022.814	479605.2661	475817.5913	485177.1121	504119.0899	488564.5281	502405.546	514059.945	529314.2767
Q6VN19	Ranbp10	Ran-binding protein 10	93.852	5.97	67188	3	16317.19526	17218.75088	20683.40479	14669.87269	16587.08053	18917.58763	17999.90762	17454.13327	16714.12742	17363.31652	20736.59617	18048.77449	19602.4362	16410.6736	16501.6857
Q6WKZ8	Ubr2	E3 ubiquitin-protein ligase UBR2	80.05	1.82	199155	3	6616.118624	6823.490629	5230.802905	6080.393564	6242.304343	5185.508161	7020.31362	6871.494069	7283.827076	6512.733493	6326.503438	6480.191532	6935.257514	6564.387643	6471.926315
Q6WVG3	Kctd12	BTB/POZ domain-containing protein KCTD12	340.99	40.06	35892	8	113846.1879	109548.2842	97281.32029	106934.94	99049.50661	88811.82107	88697.60762	80090.2937	86039.9739	92854.20376	99507.19823	87916.31606	97712.07632	100246.5994	91856.78007
Q6Y7W8	Gigyf2	GRB10-interacting GYF protein 2	104.926	3.64	149193	2	5127.440879	2940.476629	3407.126165	4104.117577	3104.970224	3671.791671	4440.874824	4535.34619	5220.424187	3230.549926	2943.322418	3419.442241	3485.710904	3145.733215	3659.86121
Q6ZPE2	Sbf1	Myotubularin-related protein 5	109.768	3.64	208693	5	14631.83098	13687.27007	11451.80576	7446.246028	7301.130372	12210.33999	12028.17011	11476.71427	12025.86321	14698.01372	12723.46938	7653.734474	11450.65065	14600.19946	8343.867736
Q6ZPF4	Fmnl3	Formin-like protein 3	410.717	14.4	117169	10	36777.65835	40776.90091	37721.66297	38120.81573	33349.73824	31258.1915	33876.5723	33068.06007	33470.53462	28819.37658	27007.36105	31029.65164	35848.04776	28870.54052	36276.40573
Q6ZPY7	Kdm3b	Lysine-specific demethylase 3B	68.068	2.05	170875	2	694.6007833	576.3008462	6369.000943	6620.547494	6642.750079	5695.364402	252.2559254	2308.387936	213.8306958	125.4338691	6161.716762	296.9863866	2452.066159	215.089929	198.4239428
Q6ZPZ3	Zc3h4	Zinc finger CCCH domain-containing protein 4	69.297	2.99	140967	2	2012.704437	5700.314523	1829.990612	7550.650423	4299.135227	4449.316751	5036.313553	4506.786739	3727.550011	1320.273816	3233.063492	5199.527301	5136.6204	5218.200253	5283.351138
Q6ZQ08	Cnot1	CCR4-NOT transcription complex subunit 1	144.694	4.08	266808	8	43076.4646	39721.40699	41565.31008	40988.82619	33191.35818	36604.09407	34123.4893	34338.60295	33274.53906	39262.83602	40401.35276	31480.748	36198.79004	41025.09533	39291.71693
Q6ZQ38	Cand1	Cullin-associated NEDD8-dissociated protein 1	877.807	33.66	136332	29	359091.7863	367790.5632	366856.906	366396.348	361862.2188	339413.7771	363703.4479	368946.5334	377241.4177	324229.8797	339722.9548	358653.5964	341145.5465	356915.4012	357921.0754
Q6ZQ58	Larp1	La-related protein 1	185.366	7.65	121125	5	8391.227618	8954.38986	6536.752321	8611.813054	7858.547373	7101.988654	9475.293627	9805.786817	9872.241887	6402.09642	8420.224392	8736.107506	7917.235799	7190.742481	7796.324897

Q6ZQ88	Kdm1a	Lysine-specific histone demethylase 1A	41.547	4.69	92851	2	8299.464369	7118.415388	6716.926599	7350.355812	7724.055111	5933.492657	8329.508846	14502.2204	7131.725208	5482.316668	7644.597698	7653.008769	9032.901268	8106.683536	8645.504118
Q6ZQA0	Nbeal2	Neurobeachin-like protein 2	119.261	1.5	301924	3	10257.61804	9512.856299	8616.926703	11264.97436	9024.439543	9033.099384	1113.597434	6902.362127	6180.336357	7817.41956	11669.7433	6506.709763	14216.78567	10995.00663	5395.464107
Q6ZQI3	Mlec	Malectin	104.181	12.37	32342	3	32187.16992	32848.642	29432.20314	29286.58966	32145.57204	38557.60336	36921.17194	36264.87014	37518.40881	34839.71144	26590.50012	30023.19518	30755.66076	34318.97843	29640.36721
Q6ZQK0	Ncapd3	Condensin-2 complex subunit D3	73.851	2.32	169432	3	1076.041022	2571.700608	5372.662962	1036.056276	3894.231631	1870.282461	2537.334759	4259.411242	4297.402175	2292.211624	4623.658499	4763.937935	2725.741628	2737.565398	1987.067718
Q6ZQL4	Wdr43	WD repeat-containing protein 43	66.597	5.61	75381	2	5754.926694	6236.958212	5760.989125	6096.868901	4964.591096	5695.716484	6211.255427	7206.087568	6997.611697	6715.606999	5667.075631	4887.325883	6279.177328	5844.55807	7010.849909
Q6ZQM8	Ugt1a7c	UDP-glucuronosyltransferase 1-7C	815.454	55.74	59758	26	543471.7332	552468.3544	428331.9415	448450.3223	447346.3432	604254.7696	506381.7433	525730.1423	622317.1057	570586.6834	550636.672	559317.0289	534756.8705	550845.5978	489990.3622
Q6ZWM4	Lsm8	U6 snRNA-associated Sm-like protein LSm8	113.322	46.88	10403	3	39146.19305	40849.34507	14531.6698	41819.05869	39277.90275	39252.80206	46201.31572	44606.86803	39455.23375	41285.70287	39469.25135	39570.10467	36238.18112	42792.46629	38243.98354
Q6ZWQ7	Spcs3	Signal peptidase complex subunit 3	55.049	17.78	20313	3	39601.83742	32975.62631	40360.4178	43241.47393	40767.13362	60088.102	52210.29314	54187.39032	51786.6231	53387.62549	41121.48732	44981.20042	46026.17932	46120.73849	44687.67316
Q6ZWR6	Syne1	Nesprin-1	57.737	0.51	1009926	2	1938.768068	4924.679137	2987.806849	5674.336622	5426.188193	4390.376911	4417.823681	6352.125546	5846.183524	5351.554923	4505.096499	4607.624089	6340.031852	5922.658593	6179.759976
Q71LX4	Tln2	Talin-2	478.82	5.35	253621	15	388351.479	424638.936	391868.3693	414193.4622	415444.3268	366577.5248	387276.6356	382420.6625	376665.5718	364216.3616	394222.5864	399644.8147	381872.2407	405467.1235	411159.8997
Q75Q39	Tomm70	Mitochondrial import receptor subunit TOM70	18.207	2.62	67445	2	20512.48643	22529.83921	22828.22402	20914.44506	10107.2154	26214.79126	11000.81955	23923.7361	23627.22036	24728.66392	21237.92843	21909.73967	24141.97443	23323.96235	23571.45012
Q75Q40	Tomm40	Mitochondrial import receptor subunit TOM40 homolog	276.063	20.78	37919	4	34913.7394	38259.21391	39587.60625	38912.42871	35067.14195	37743.79262	28939.88389	45457.97341	44067.80691	34732.05103	38456.11443	37604.83013	30433.58795	22287.27329	36247.63745
Q76MZ3	Ppp2r1a	Serine/threonine-protein phosphatase 2A 65 kDa regulatory subunit A alpha isoform	1194.761	59.76	65323	29	549836.4159	572173.8317	527421.1732	549375.4565	559352.9309	546881.1863	563363.777	603416.492	563367.4811	520437.0854	527755.3869	579019.4664	541531.2633	566023.5146	587884.2814
Q78IK2	Atp5md	Up-regulated during skeletal muscle growth protein 5	166.998	44.83	6382	3	39385.15575	45642.35877	40605.29342	44748.60266	42956.53648	47907.97026	47665.0174	51068.33345	51035.56207	46526.12748	37465.89774	38629.2877	44907.6188	41523.51062	39153.01427
Q78IK4	Apool	MICOS complex subunit Mic27	230.272	26.42	29261	5	63540.69124	67867.99225	58902.98801	62470.29765	61504.61145	60226.38276	59153.39558	56308.39634	57136.85078	55959.06426	61050.72976	55928.68414	60718.69711	58040.77608	59217.81043
Q78P75	Dynll2	Dynein light chain 2, cytoplasmic	86.955	25.84	10350	2	21071.66335	24168.2517	23458.88609	18548.53189	19143.81196	13215.7826	15751.58081	14592.94783	12660.57102	13386.39077	15148.05916	16776.43605	14183.76599	14720.18031	15765.60659
Q78PY7	Snd1	Staphylococcal nuclease domain-containing protein 1	1339.608	56.48	102088	33	538457.9134	569057.9955	516726.0537	575390.7593	513682.341	510982.4797	561845.0134	538980.1733	528831.953	484131.6373	488232.2687	535997.5605	509540.0241	523036.8481	512221.3298
Q78ZA7	Nap1l4	Nucleosome assembly protein 1-like 4	292.049	32.27	42679	8	118628.9381	121078.3213	99050.01278	110246.8273	107313.3404	98472.17285	105220.2488	104428.3814	102972.6052	93876.3949	115609.8486	105985.3039	116754.1849	106874.1223	100915.027
Q791V5	Mtch2	Mitochondrial carrier homolog 2	426.22	46.2	33499	11	163505.8431	160925.3249	155191.1924	149082.6269	148041.9386	164741.6561	147280.7939	152948.3492	156918.1973	141048.6447	137401.6886	136011.8615	131558.7069	154183.6125	150338.4725
Q792H5	Celf2	CUGBP Elav-like family member 2	155.568	10.24	54271	5	42654.41246	44914.68801	45632.10266	51361.59202	46218.28161	18566.97309	45306.76138	39577.93334	36193.64669	26547.4139	36180.47395	41995.59385	38664.69775	40014.44903	36225.49206
Q794E4	Hnrnpf	Heterogeneous nuclear ribonucleoprotein F	906.329	65.78	45730	19	845815.4203	896475.7478	881754.9919	920870.8189	889992.2479	766281.7579	815713.8348	854828.9756	821881.442	797243.6765	843166.0408	848296.1711	808599.6867	805440.785	795361.8193
Q7M6Y3	Picalm	Phosphatidylinositol-binding clathrin assembly protein	553.799	26.52	71543	12	152632.6679	160037.4822	138986.5213	152247.8407	157389.0545	111727.2647	136968.1016	142774.5912	128898.8651	139388.8695	149797.8597	154414.3249	153087.1607	141725.4525	140064.5757
Q7TMB8	Cyfip1	Cytoplasmic FMR1-interacting protein 1	855.917	21.79	145241	24	254985.1928	274934.595	235503.1301	244816.7329	239640.9471	224962.4956	242991.6803	238528.3443	257418.2032	241237.0433	229032.35	253362.5781	254463.8757	255727.7069	250756.4897
Q7TMF3	Ndufa12	NADH dehydrogenase [ubiquinone] 1 alpha subcomplex subunit 12	41.292	17.24	17086	2	1025.700374	5610.369041	738.4982039	1003.19557	915.6415967	4537.419793	1036.022529	4742.633502	5649.85791	891.1082497	955.067833	4478.743246	924.2025894	913.6173952	907.1572004
Q7TMK9	Syncrip	Heterogeneous nuclear ribonucleoprotein Q	603.878	33.71	69633	18	419594.2451	423132.5028	475142.8745	458983.665	477774.9868	444004.7483	470735.9464	482422.864	449595.4334	457368.9841	458415.3228	468029.9913	444333.8834	459217.3739	459650.3379
Q7TMQ7	Wdr91	WD repeat-containing protein 91	201.738	11.1	83421	7	31093.85293	32473.29284	34936.54337	34008.78634	28994.84153	30668.57718	32311.14389	26852.53075	30270.02304	26934.87483	33398.42394	31218.67682	29688.67731	34750.96057	32507.41049
Q7TMR0	Prcp	Lysosomal Pro-X carboxypeptidase	92.213	13.85	55027	3	15648.9649	18035.58442	15373.24519	15386.48668	13130.68785	12105.61527	11087.04351	14218.33031	8837.17667	11126.61853	13640.22829	13174.38789	14384.22781	15741.46263	14031.50984
Q7TMY8	Huwe1	E3 ubiquitin-protein ligase HUWE1	171.937	3.29	482635	8	26108.51891	27376.59522	23786.61211	15961.73613	19048.61199	14937.77372	13229.84335	21942.93718	20020.68281	12568.71025	22693.63258	18064.58489	16275.44616	15229.84156	14236.37672
Q7TN05	Ap1s3	AP-1 complex subunit sigma-3	14.946	12.34	18440	2	3321.827113	2924.374582	1734.440287	1970.416936	1881.150624	1261.190853	2662.714188	1626.912204	1732.145778	861.5683376	1727.892561	2835.088443	3065.648226	1821.826615	2824.869233
Q7TNC4	Luc7l2	Putative RNA-binding protein Luc7-like 2	113.31	8.67	46583	3	33769.26695	41659.19661	44132.33637	43756.46809	44313.6004	37897.02756	42199.05178	43627.8276	36456.33952	43987.77938	40815.49802	45038.98963	41586.21891	43563.01139	38915.38257
Q7TNG5	Eml2	Echinoderm microtubule-associated protein-like 2	276.909	20.03	70734	9	96413.48096	95170.30185	89282.91862	88958.06366	95670.8781	84665.41923	95166.27551	95883.06986	90515.73895	85097.06637	85128.09125	90971.32312	91241.95073	91185.27004	93839.64985
Q7TPD0	Ints3	Integrator complex subunit 3	163.768	6.34	117938	3	8236.075807	7747.919871	8289.342143	9918.45802	6357.777897	6045.747615	7978.725001	8478.256957	7475.394856	8204.579117	8569.046969	7872.207289	6744.091581	7715.894391	8024.594662
Q7TPJ0	Ssr1	Translocon-associated protein subunit alpha	59.898	10.66	35629	3	91777.21708	93956.92738	59995.20203	89004.28602	92953.32819	116210.1102	115008.7113	114894.5028	118619.4071	109221.7031	104006.5959	100796.0949	103145.8821	101682.0907	102047.98
Q7TPR4	Actn1	Alpha-actinin-1	1341.234	44.73	103068	32	709871.1486	687717.5613	615846.4399	662365.5076	643756.2116	508731.8351	563249.924	585402.8842	572247.5621	586496.581	667561.9816	649872.5016	643771.0549	681692.4772	676829.37
Q7TPV4	Mybbp1a	Myb-binding protein 1A	1303.757	42.49	152037	36	359822.0545	395076.1825	358222.6998	403250.2813	357092.1238	357943.2975	390508.1325	425428.301	390223.4219	331320.828	383678.7174	427297.989	374132.7406	355854.2864	390549.5914
Q7TQH0	Atxn2l	Ataxin-2-like protein	125.609	5.72	110649	4	17917.85091	19295.89313	13150.94033	24621.51061	9904.102584	16338.69975	24222.46271	17016.69906	24228.72683	18187.59633	23379.98825	16442.76577	16425.21027	21197.93134	21322.89393
Q7TSI3	Ppp6r1	Serine/threonine-protein phosphatase 6 regulatory subunit 1	80.721	5.26	94527	3	17204.51555	9296.763106	13660.68261	11759.72215	11116.60077	8959.042733	14014.95007	12185.39504	12325.42318	12452.86458	11454.34363	14777.68626	13768.72346	10888.88286	11780.30527
Q7TSS2	Ube2q1	Ubiquitin-conjugating enzyme E2 Q1	36.697	4.5	46173	2	9908.063574	10008.5717	10869.65973	5903.348847	10201.63631	9317.783204	10262.07437	9753.532446	11300.61071	10820.12935	8658.631796	11290.74327	12606.3088	11582.14839	11163.02387
Q7TSV4	Pgm2	Phosphoglucomutase-2	82.058	12.42	68748	4	26805.6597	23241.08819	26796.58182	13964.93581	22753.74607	10023.75771	18232.96964	12903.5359	11087.92879	11580.07099	25181.14738	21813.24016	25409.80113	24846.65525	21053.68886
Q7TT50	Cdc42bpb	Serine/threonine-protein kinase MRCK beta	72.4	2.22	194753	2	3760.938857	4251.540008	2582.293555	3177.740409	3233.448536	2938.959609	3453.411682	3856.588365	4085.558894	2440.572066	3297.522324	4406.025577	3395.137025	3234.19051	3327.252363
Q80SW1	Ahcyl1	S-adenosylhomocysteine hydrolase-like protein 1	265.391	25.28	58951	10	58072.95463	53832.88604	53452.7402	58228.05693	57979.36726	49824.57027	49066.09997	51362.5611	51821.06076	44439.88983	51118.42095	51268.99298	54274.42997	48579.9258	53379.05776
Q80TH2	Erbin	Erbin	56.959	3.64	157248	3	9549.973253	9492.670321	7441.056887	9298.780304	9146.013797	6257.946973	6841.031496	6592.140369	7036.915933	8606.00959	7946.749854	7981.734975	8251.809797	7678.342085	7978.372958
Q80TL7	Mon2	Protein MON2 homolog	84.94	3.32	189078	4	11199.9828	9769.047311	9046.782105	10919.66167	10618.66956	7956.425419	11668.30934	8127.424124	11166.63997	6245.033101	12725.67209	8777.150567	12265.92512	11955.99768	10874.41634
Q80TM9	Nisch	Nischarin	318.819	9.04	175012	10	53861.93882	50413.03687	48516.37028	48454.02201	36689.63087	50125.25332	55803.76084	58664.27278	55275.77357	52665.1219	49692.32964	49001.94068	52663.04308	54707.03338	52817.10876
Q80TY0	Fnbp1	Formin-binding protein 1	347.112	27.11	71344	13	145463.318	156368.8484	134003.0392	150667.4486	145170.7925	142237.5507	138729.7102	132299.0687	142088.2928	147603.6818	148231.9297	149876.4797	165434.0756	161386.3078	150130.3732
Q80U93	Nup214	Nuclear pore complex protein Nup214	170.162	4.22	212979	6	19815.01454	18247.69316	16245.82766	21026.53887	20295.80405	15914.82462	18125.01928	17246.65891	17382.06533	14322.38945	18807.23141	20446.53292	16832.83892	19320.57369	16951.34073
Q80UG5	Sept9	Septin-9	112.081	10.81	65575	6	75560.379	81962.17983	80555.59128	87641.33163	80304.42315	78458.96772	80576.8806	81761.7323	81934.8305	88999.79455	82811.47036	76339.03823	74464.09229	91843.17595	82568.32984
Q80UJ7	Rab3gap1	Rab3 GTPase-activating protein catalytic subunit	70.334	6.01	110198	3	14642.30516	16336.00711	15399.14901	14236.53804	14801.46282	13668.24749	15498.18395	14840.10594	17321.74658	12225.6164	13955.57054	14390.85271	13517.48175	13895.59223	14186.01603

Q80UM3	Naa15	N-alpha-acetyltransferase 15, NatA auxiliary subunit	465.937	25.78	100961	18	120460.7949	148956.9248	125263.7484	148257.3559	124090.4634	140516.0821	135637.998	159385.6072	157009.0281	140975.1839	111280.1258	154997.0987	121735.0779	128818.173	141772.6873
Q80UM7	Mogs	Mannosyl-oligosaccharide glucosidase	495.036	21.82	91831	11	89378.04751	96348.19348	84668.42137	91527.30249	79757.49607	109327.3763	101203.957	93485.72181	94182.46687	112747.3235	93424.82018	93122.95097	107294.0557	97824.86868	96748.43007
Q80UU9	Pgrmc2	Membrane-associated progesterone receptor component 2	189.428	20.28	23334	2	4567.883493	9845.597864	9685.006931	8457.057558	9724.927693	10018.49067	3873.653841	7882.874015	8899.409919	3433.255265	6258.37741	4301.115095	3938.334276	3382.15414	5012.592009
Q80UY2	Kcmf1	E3 ubiquitin-protein ligase KCMF1	89.458	8.14	41791	2	2383.652643	2207.79221	1922.717205	2267.5042	1188.270702	1085.099778	1065.494076	2121.331521	2209.145391	1294.678344	2290.672715	1507.487475	1908.937147	1839.412475	1723.941782
Q80VA0	Galnt7	N-acetylgalactosaminyltransferase 7	30.194	5.02	75419	2	21412.11024	21507.93754	16585.91182	21408.51763	23499.60138	27394.95225	28445.71031	25275.94119	27748.62196	25265.82877	22865.81593	22379.71114	24845.75244	24441.26906	21936.2552
Q80VD1	Fam98b	Protein FAM98B	80.732	6.76	45349	2	20797.90325	18245.25428	17106.01396	17039.3901	19645.27682	14916.93455	12164.15694	14078.19958	9390.045996	14927.73866	16112.64599	17400.40188	16863.18862	16045.16361	16107.21025
Q80VJ3	Dnph1	2'-deoxynucleoside 5'-phosphate N-hydrolase 1	97.056	17.92	18977	2	16976.81882	11263.22184	11636.37186	13157.03724	13156.42257	14619.55109	14812.48553	13143.72313	15268.86071	12657.01476	14315.05747	13794.99012	10101.08664	13833.61302	13345.96884
Q80VL1	Tdrkh	Tudor and KH domain-containing protein	86.022	6.25	62134	2	8225.858205	8975.100481	7907.904726	13233.64805	7941.974687	9033.128463	8473.431855	7423.554068	10733.16357	11957.62836	8285.42572	9013.553207	0.00981159	7541.264168	7595.922063
Q80W54	Zmpste24	CAAX prenyl protease 1 homolog	94.956	13.26	54735	4	15268.76939	16907.09995	21755.72406	16560.48882	18971.56799	23135.70703	25619.4533	20871.26085	23318.45962	22311.31141	17445.64021	12992.78958	24578.67114	20442.59676	18666.69679
Q80WJ7	Mtdh	Protein LYRIC	516.935	27.98	63846	11	77941.63983	82351.92405	77544.4471	89677.72016	77239.61624	63580.66723	90506.83195	74601.95311	77540.76123	67990.61633	76132.16054	87326.91236	89097.31373	61812.62153	85112.05042
Q80WQ2	Vac14	Protein VAC14 homolog	349.148	19.44	88048	9	45301.20295	48925.14671	41911.38073	44063.18189	36946.16593	35251.67463	39960.09437	42725.73497	42903.38227	36357.17045	40385.93582	40850.03074	40739.21377	41259.57	47415.36883
Q80WS3	Fbll1	rRNA/tRNA 2'-O-methyltransferase fibrillarin-like protein 1	101.924	11.46	33339	3	128669.822	122452.1324	118268.0344	108542.4511	123049.2379	115018.4355	126253.5652	121887.1736	123274.4594	119255.6734	126014.8122	128137.1324	125554.5043	125537.5797	117018.9168
Q80X41	Vrk1	Serine/threonine-protein kinase VRK1	196.372	25	49741	8	58969.13571	52539.59573	56328.58203	57992.45128	53397.80417	46957.55082	50205.7746	46540.50193	48817.33015	48960.13855	47202.06296	52164.7868	54535.37411	56653.60198	55869.59314
Q80X50	Ubap2l	Ubiquitin-associated protein 2-like	211.611	9.03	116799	7	72707.55171	57921.31703	69446.7714	80281.96117	66296.15629	76975.10122	69240.47509	68992.28045	74181.97842	65320.53158	84129.51433	74631.41045	78169.16459	76018.74813	73243.18819
Q80X82	Sympk	Symplekin	251.144	12.38	142284	8	25869.20042	25248.54845	20223.7144	23375.32042	20444.43453	18158.54649	19559.40722	20529.03499	22283.58852	19154.6702	20544.12981	20889.52764	20757.78773	22460.70729	19868.92471
Q80XI3	Eif4g3	Eukaryotic translation initiation factor 4 gamma 3	137.247	3.74	174890	5	31490.64816	28630.76136	33473.60423	26371.82491	30339.05824	23421.3014	30617.05673	35473.14976	31136.08469	27207.84708	30001.01785	29517.51359	28179.40945	30818.10676	31197.53006
Q80XQ2	Tbc1d5	TBC1 domain family member 5	48.421	3.07	91837	2	11625.77175	13907.18511	14267.7577	13223.78453	13406.09284	10548.39729	11892.61011	11555.57053	10201.40996	11734.48193	11452.54022	11816.33703	13263.31496	13961.35161	14278.11236
Q80Y81	Elac2	Zinc phosphodiesterase ELAC protein 2	56.003	4.93	92719	2	7093.071264	6321.145368	4695.83139	5597.922987	4968.349995	4607.207631	5957.51787	6771.07486	5397.463145	5034.80674	5927.867132	5321.380603	5383.158384	3764.575889	4935.650064
Q80Y98	Ddhd2	Phospholipase DDHD2	31.513	4.43	79577	2	2336.031951	9426.051111	8256.57634	3027.526627	8448.652209	6855.048336	8266.598001	9435.062677	8339.96723	8168.439785	8078.507984	8685.874235	8211.548526	8888.109341	8284.323832
Q80YW0	Cyth4	Cytohesin-4	40.33	7.89	45285	3	4203.427799	7438.614233	4107.137828	8607.876908	7363.767522	6768.599704	7000.809205	7922.135128	7565.27713	3472.907197	8898.102333	7894.213379	3904.154964	9138.054581	7611.147368
Q80ZJ1	Rap2a	Ras-related protein Rap-2a	136.594	38.25	20642	4	48520.12684	42863.31847	36866.88068	45390.06634	46440.61668	38044.01403	35884.888	32633.39271	45259.70313	38677.71684	50129.56813	40630.34481	50516.19356	42976.72306	43989.64009
Q810A7	Ddx42	ATP-dependent RNA helicase DDX42	69.43	3.55	101965	2	12945.65743	8675.353736	13771.96172	15446.58836	11116.86941	6157.337886	3970.625976	5225.416259	10945.92936	10874.20765	9307.769759	9005.15722	7148.757716	10643.23316	8493.67159
Q810B6	Ankfy1	Rabankyrin-5	529.491	16.6	128653	12	93374.56574	83124.266	87893.30095	88924.28649	86644.77505	72076.03939	85309.98898	76919.45459	79154.274	71223.10601	98894.34258	82999.65517	79935.80027	85777.79462	79831.99876
Q811U4	Mfn1	Mitofusin-1	14.964	2.43	83726	2	4533.683039	5544.90499	4277.958817	1773.64944	4855.229649	7949.795271	7063.216788	6027.877731	8439.63022	7412.798902	5394.146547	6064.321278	1790.554669	5007.271302	4736.943386
Q812A2	Srgap3	SLIT-ROBO Rho GTPase-activating protein 3	64.24	2.37	124420	2	6102.616377	5986.491004	7309.029561	10271.03078	6368.739955	5667.602473	5577.884471	6010.000779	4805.023385	6859.479603	6675.554158	5405.307684	6474.220703	5897.696788	6109.940454
Q812D1	Psip1	PC4 and SFRS1-interacting protein	125.406	10.23	59639	4	25395.86531	39390.51727	28932.76605	41824.20368	40932.66985	20147.89093	26001.92876	23023.01128	30633.19829	26506.14796	39715.75747	28732.48656	39094.99239	40045.17268	31145.34982
Q8BFQ4	Wdr82	WD repeat-containing protein 82	125.611	14.7	35079	3	9700.523416	17085.47843	16603.71082	17771.40466	11012.24636	11340.49831	11632.41695	6048.853078	10753.45006	10477.60408	11755.32503	17142.06905	14714.6233	11989.70953	11176.52091
Q8BFQ8	Gatd1	Glutamine amidotransferase-like class 1 domain-containing protein 1	95.04	10.45	23277	2	13691.24619	13446.45344	11558.15516	11970.77332	11865.0557	8552.679911	9611.275024	11660.04108	12205.52426	7847.212622	12025.38605	13217.31203	13648.34383	12486.92387	11054.6837
Q8BFR4	Gns	N-acetylglucosamine-6-sulfatase	106.863	16.36	61175	7	134064.2167	125010.6262	106646.4873	89725.91431	84441.45249	67752.26654	74146.4868	58072.50904	72215.34075	99693.01416	120493.3582	86781.31813	120536.2487	93249.46604	129995.8566
Q8BFR5	Tufm	Elongation factor Tu, mitochondrial	475.295	41.37	49508	15	363752.104	331155.4083	326764.9579	329883.5799	338885.7429	370523.5682	356397.3004	341986.8329	354631.8783	343723.7602	340418.8736	327521.3498	339550.9669	335588.4428	321464.5927
Q8BFV2	Pcid2	PCI domain-containing protein 2	114.783	10.78	46132	3	4759.090797	5362.703708	4438.025896	5516.669897	3811.65413	3533.913225	4544.992465	3844.823147	4322.383067	3736.140931	4827.8349	3532.251969	5055.549977	4907.347344	4727.361658
Q8BFY9	Tnpo1	Transportin-1	520.106	25.28	102357	18	138793.2243	143620.7633	135793.2431	152615.4552	140661.7169	154820.4821	140440.0603	147311.2093	150550.5061	146796.1189	131204.995	144437.4199	135390.751	149079.6951	142861.681
Q8BFZ3	Actbl2	Beta-actin-like protein 2	877.627	35.9	42004	21	17038360.48	17822354.05	17745924.74	18720221.1	17021821.69	17984067.15	17455294.04	17674553.08	17902872.87	17663693.15	18033342.87	18097326.8	18709223.67	18753409.65	17398280.5
Q8BFZ9	Erlin2	Erlin-2	170.441	17.65	37873	5	32201.5948	35996.16479	29668.42949	30380.4868	34694.06241	34365.49203	30869.85161	34155.5842	34415.57329	35566.67063	29084.75984	29658.51544	33316.50051	31688.35714	32273.01901
Q8BG07	Pld4	Phospholipase D4	660.298	46.52	56154	18	832412.6799	772652.8066	684692.0799	693074.3979	733098.8391	573817.2116	542990.968	519038.2501	553789.7053	626453.4654	721722.5693	662297.6926	755984.6812	763164.668	739245.9072
Q8BG51	Rhot1	Mitochondrial Rho GTPase 1	91.504	4.44	72242	3	8197.825405	11640.26782	12655.72684	12771.03466	13785.64765	17024.50574	12733.87152	14524.82656	14641.10912	10312.55697	10040.31591	12513.22884	11209.7614	12242.66307	6149.177269
Q8BG73	Sh3bgrl2	SH3 domain-binding glutamic acid-rich-like protein 2	73.009	24.3	12255	2	24651.27296	27128.86371	23337.60373	27268.57543	20987.51593	23805.4738	24344.01645	19671.37524	18687.42003	24002.37241	21626.15415	26907.45987	24681.67812	28776.61306	26784.63751
Q8BG81	Poldip3	Polymerase delta-interacting protein 3	192.148	15.95	46132	5	26786.86869	26159.80253	22576.25876	21089.98915	22597.17417	21120.50173	23876.41061	21522.38878	20041.52967	21684.32022	25704.80845	25370.97843	26092.32233	23854.96182	21948.73432
Q8BGD9	Eif4b	Eukaryotic translation initiation factor 4B	406.935	21.6	68840	8	75711.77647	65060.48323	71877.221	66916.38909	72062.51868	58694.0333	66407.25354	73168.56142	60860.11083	58586.63097	70013.24369	67622.0023	68197.72399	58436.29694	61649.87161
Q8BGH2	Samm50	Sorting and assembly machinery component 50 homolog	237.323	22.81	51864	8	110915.1693	116352.9459	120465.0726	103918.6191	120510.0735	146832.0982	125760.9599	128715.0271	136237.831	133569.1333	122719.32	91582.93498	124918.1441	125348.5002	124294.509
Q8BGQ7	Aars	Alanine--tRNA ligase, cytoplasmic	1233.768	47.83	106909	29	385913.4565	426862.2257	391423.6856	412414.4516	391317.8385	385851.163	433474.1295	438659.2799	425297.144	302536.277	402269.6188	438415.616	402052.6409	389963.1899	404137.1121
Q8BH04	Pck2	Phosphoenolpyruvate carboxykinase [GTP], mitochondrial	736.834	42.81	70528	19	242625.2062	225354.1422	213600.4207	228619.5367	208120.7472	236876.9743	231111.2134	232873.666	240795.6195	218659.393	212806.1147	204072.7375	214023.5622	226595.1075	237819.023
Q8BH43	Wasf2	Wiskott-Aldrich syndrome protein family member 2	286.419	16.9	54074	7	124296.9572	120902.7371	119066.7628	115780.4236	116086.1114	119136.4993	116968.0807	105048.9906	104992.0292	105945.3967	77192.04314	118668.4156	126809.8678	131529.2489	118446.0849
Q8BH59	Slc25a12	Calcium-binding mitochondrial carrier protein Aralar1	777.57	48.6	74570	21	227800.9222	212269.4842	219011.5638	205410.4449	214514.5049	236299.4098	216413.2445	216796.7055	220616.1459	230388.5769	212266.2964	211832.8393	217310.9355	228508.8437	217817.8242
Q8BH61	F13a1	Coagulation factor XIII A chain	164.935	14.48	83207	9	78656.12174	55470.83343	39616.09083	35866.47133	50518.43767	13791.46684	19390.04481	12063.10051	15557.43917	26305.93889	30442.3057	21273.88405	33666.83806	32358.51138	28491.49802
Q8BH69	Sephs1	Selenide, water dikinase 1	36.156	6.89	42907	2	12673.79247	14716.28702	13755.40376	7406.976509	10065.88817	8615.99434	11222.79028	9218.839908	10374.92287	6672.728451	7374.640801	7175.25887	13120.94633	6682.912222	13208.27502
Q8BH86	Dglucy	D-glutamate cyclase, mitochondrial	162.102	14.42	66366	5	18206.27445	13547.42304	15127.88694	14337.77556	16208.93918	9718.729078	7858.725617	8664.869632	10619.83046	12374.0499	11477.56359	12743.79695	14381.88039	12831.15591	11977.44198
Q8BH95	Echs1	Enoyl-CoA hydratase, mitochondrial	206.452	23.1	31474	5	52633.39322	53325.20216	44494.53584	50402.29458	45369.97717	42238.65165	38614.57685	39382.85082	38765.26028	41705.47857	42986.47065	41171.8452	41003.89613	45796.50424	44338.34581
Q8BHC4	Dcakd	Dephospho-CoA kinase domain-containing protein	96.641	23.38	26476	4	19451.39817	20228.12535	16973.33397	19158.54097	16193.7951	16519.95289	20421.31991	18590.73874	17260.25875	19319.2228	19370.52915	18661.82304	17538.59151	17906.73035	17822.802

Q8BHD7	Ptbp3	Polypyrimidine tract-binding protein 3	385.747	37.48	56701	12	399023.5697	370692.5041	418331.959	459089.9398	441568.3425	343394.9487	399216.5728	393142.7928	393170.6083	387455.2125	408745.668	390230.5495	369638.6006	398493.3756	380103.2247
Q8BHF7	Pgs1	CDP-diacylglycerol--glycerol-3-phosphate 3-phosphatidyltransferase, mitochondrial	34.03	4.16	62489	2	7450.602194	5859.11769	12296.25196	7406.188941	3543.319303	9830.364785	9764.734945	8932.785276	9474.180383	8145.719742	5950.691328	6867.653416	4105.96868	9678.067696	9358.794871
Q8BHG1	Nrdc	Nardilysin	237.392	13.7	132891	12	52864.40517	70371.21234	60346.68383	62555.98786	65909.40502	71883.85233	70095.82919	67709.42384	61318.82036	65451.95521	68284.01152	69766.40254	60670.20823	65817.35771	70776.44551
Q8BHL5	Elmo2	Engulfment and cell motility protein 2	288.182	21.45	83887	12	125460.9178	66634.80424	149494.2953	147037.7092	144547.7782	88463.62195	88829.48932	65370.33601	86768.43901	66487.23343	87477.17141	74884.90676	90006.58323	77357.09914	85329.83673
Q8BHN3	Ganab	Neutral alpha-glucosidase AB	999.162	43.64	106911	31	583785.054	586531.0477	563682.2602	549605.4678	567588.4376	680867.2409	649421.3302	678890.4668	689753.6663	639805.5674	610811.6696	617685.1684	625006.585	614341.1735	604305.2517
Q8BHZ0	Fam49a	Protein FAM49A	122.261	18.58	37343	4	22979.64461	26824.89052	20710.36303	23806.99968	18171.73042	9603.793781	17297.76721	15515.83529	14037.60436	15833.12342	23556.69771	24004.81462	22735.9613	23304.76125	20435.56309
Q8BIG7	Comtd1	Catechol O-methyltransferase domain-containing protein 1	87.731	21.76	28961	3	20818.75701	9301.248564	3121.392374	9605.410422	2483.097584	3374.971408	4651.642408	4320.102826	4081.157158	3332.844766	11394.90673	15370.8951	3035.618285	6965.21942	3418.968262
Q8BIJ6	Iars2	Isoleucine--tRNA ligase, mitochondrial	409.097	16.9	112804	12	106509.1605	111154.5035	102980.1764	113251.2717	101164.5581	118753.5719	118100.9668	107215.4987	119260.0562	107929.312	103880.9993	101325.2323	112184.6526	114558.1737	114082.8265
Q8BIJ7	Rufy1	RUN and FYVE domain-containing protein 1	133.283	11.24	80376	6	20535.44819	26791.19807	22551.16684	23576.14695	22495.60949	15182.34311	12902.79401	21297.97306	14793.16948	24783.02028	20536.48973	20703.31697	25261.34687	22357.27148	20780.98716
Q8BJ71	Nup93	Nuclear pore complex protein Nup93	194.733	14.65	93281	7	60087.53891	69334.59421	65380.35922	58755.40914	63818.46921	68304.77165	71644.865	64992.89087	68507.84952	57291.25612	57260.57988	60483.22621	61089.60465	53879.84008	63332.35147
Q8BJS4	Sun2	SUN domain-containing protein 2	309.489	16.01	81605	8	56903.53582	79908.4546	78808.08689	89468.46309	90888.69847	77099.28565	62742.76359	66049.08043	74806.23036	76010.18138	85890.05512	77487.26868	89224.62892	84611.27948	84463.90275
Q8BJW5	Nol11	Nucleolar protein 11	42.597	5.67	80818	2	4810.817459	4221.922752	4393.28649	5907.355473	3566.263509	4661.254756	5031.679047	5973.298229	5523.267817	4290.232401	5872.544488	3826.194199	3885.703092	4679.383081	3855.521564
Q8BJW6	Eif2a	Eukaryotic translation initiation factor 2A	368.948	31.84	64403	13	108755.386	120201.0206	108034.2125	100161.2179	116423.127	104693.8185	114208.9377	110557.1678	104424.11	95053.28004	107076.0962	109603.476	110055.9496	102982.8669	109064.2407
Q8BJY1	Psmd5	26S proteasome non-ATPase regulatory subunit 5	133.829	10.91	55972	4	51124.43242	31867.26732	32222.7743	29818.71085	49181.68296	29855.22989	35579.06877	33941.74815	30397.29735	31639.60314	36186.3398	32135.45382	35121.20516	51630.75372	32553.68529
Q8BK64	Ahsa1	Activator of 90 kDa heat shock protein ATPase homolog 1	296.164	32.84	38117	7	105604.8395	109082.8553	82311.43587	113393.057	102798.6274	102264.785	113213.5889	120680.3517	124207.4056	109252.6259	111659.8203	121109.7095	111877.1966	115341.4517	125368.304
Q8BK67	Rcc2	Protein RCC2	650.406	48.27	55983	19	383684.7448	400911.4937	409006.9143	347067.4401	390473.2661	267541.1641	334393.7519	319000.5754	308262.6544	298527.5401	312931.0769	335490.527	335611.4957	335396.1387	339616.4156
Q8BK72	Mrps27	28S ribosomal protein S27, mitochondrial	55.244	7.47	47779	3	13783.26392	15577.98648	12843.88531	15131.86933	15765.24544	13869.37836	18201.80826	13898.02406	14506.10144	14428.53302	13251.93819	18440.43097	13965.65311	16040.81724	17639.89143
Q8BKC5	Ipo5	Importin-5	952.868	31.08	123591	24	278963.3315	278782.3823	217733.2787	280058.7897	234425.8183	223852.2219	268958.1998	283757.6059	251910.1604	219249.0644	271432.7878	290199.2955	255964.9117	229957.0254	240574.0857
Q8BKE6	Cyp20a1	Cytochrome P450 20A1	57.927	9.96	52149	3	9557.685443	9071.657405	13925.0809	8529.550547	15966.77845	8941.726704	13899.80518	9197.860188	13603.96937	9414.86795	8300.821018	12667.57302	8748.261543	13897.52723	8181.304407
Q8BL66	Eea1	Early endosome antigen 1	379.141	9.99	160915	11	89314.03017	84720.92639	70584.02439	75498.27683	85577.11538	55486.64078	76677.52884	75999.08751	76561.51147	86838.0481	94662.0459	85246.66397	85599.14824	85937.40818	88230.34416
Q8BL97	Srsf7	Serine/arginine-rich splicing factor 7	91.117	14.61	30818	4	82013.34677	88626.44523	72166.08475	66254.93241	93634.54242	92151.36582	92733.95335	83210.63506	90134.57455	60411.05035	65303.31951	61892.83269	99817.10394	87981.87826	57156.97851
Q8BLF1	Nceh1	Neutral cholesterol ester hydrolase 1	471.958	49.51	45740	12	195991.4439	192160.6427	175278.5726	197308.0866	158658.4921	223752.6679	230149.613	233834.1438	245617.6884	216724.1031	189807.7541	192631.8062	200006.6853	186785.9225	222132.4819
Q8BLN5	Lss	Lanosterol synthase	44.572	3	83141	2	4568.418205	4862.286864	3467.645188	1990.74753	4488.693071	1463.831892	4240.947225	5251.927472	5502.494179	945.0659292	3864.103526	2202.758056	4111.199778	3639.967906	2074.356455
Q8BMA6	Srp68	Signal recognition particle subunit SRP68	116.462	6.72	70574	3	13767.12591	11146.7682	9572.813679	13195.33691	8344.3169	7607.744236	10962.17574	9568.409916	10439.99341	9121.137849	11406.13651	9237.383875	10680.87499	8422.834974	9708.995084
Q8BMD8	Slc25a24	Calcium-binding mitochondrial carrier protein SCaMC-1	401.143	41.05	52902	12	188018.9733	190006.8667	174534.3455	183568.7462	210962.4504	200616.6483	179580.2654	191538.8061	190630.4117	182124.3049	190089.057	172970.4158	191173.8601	178042.4585	175515.8908
Q8BMF4	Dlat	Dihydrolipoyllysine-residue acetyltransferase component of pyruvate dehydrogenase complex, mitochondrial	477.868	30.53	67942	12	222732.2657	225461.7403	212000.0347	216890.9058	203530.1372	256268.2831	235479.8813	223412.917	259359.641	235744.2511	215742.656	232386.3961	241849.1142	231747.0159	224777.662
Q8BMG7	Rab3gap2	Rab3 GTPase-activating protein non-catalytic subunit	136.472	7.69	152535	8	43004.96541	45945.04068	43516.01288	38790.77861	40090.71772	44382.85768	47348.99525	48308.45768	45767.94247	46101.52987	45498.31381	46726.75053	42396.65136	45580.69639	44577.99779
Q8BMJ2	Lars	Leucine--tRNA ligase, cytoplasmic	855.886	30.14	134192	26	285470.4615	306538.8982	283703.462	296965.1506	286023.5912	324687.3158	328361.0778	344542.2008	330266.6469	291402.5988	305146.3578	314411.7274	298290.0316	294126.8003	301027.3862
Q8BMJ3	Eif1ax	Eukaryotic translation initiation factor 1A, X-chromosomal	158.528	18.75	16460	3	20149.90659	21277.27819	16222.59494	20561.6327	16967.06827	12244.41401	13485.65125	14970.98661	14992.30974	12441.42254	17009.93055	15680.47674	17357.13473	15054.84963	16021.92071
Q8BMK4	Ckap4	Cytoskeleton-associated protein 4	910.017	51.48	63692	24	294827.8192	319749.4099	290988.4582	289078.0749	318473.4688	402396.1752	364484.1185	368682.026	393253.5245	394190.1654	334206.1677	313895.8678	343749.6995	330190.0455	330598.709
Q8BML9	Qars	Glutamine--tRNA ligase	529.013	23.87	87677	15	117885.7792	146804.4496	130412.4843	137973.0546	135470.4185	136205.4677	119465.4608	150567.019	123713.6301	110136.74	135362.2427	138171.7042	143359.2437	132629.4467	133335.1432
Q8BMP6	Acbd3	Golgi resident protein GCP60	221.869	18.86	60181	6	24536.99477	26030.74802	25207.88829	24937.56289	19538.56458	23229.8595	20445.06294	21182.11569	26564.08453	23915.11353	19766.83751	25657.32506	24545.84156	27529.37624	25702.8287
Q8BMS1	Hadha	Trifunctional enzyme subunit alpha, mitochondrial	1127.238	47.44	82670	24	421240.6638	408911.8338	404841.5324	396928.1993	372196.0633	365087.6897	352773.7641	334147.3998	352267.5945	367595.693	403551.5424	384304.4139	367538.9596	375836.9315	386392.4949
Q8BNV1	Trmt2a	tRNA (uracil-5-)-methyltransferase homolog A	34.814	4.36	63301	2	12794.80804	8606.600034	5410.084487	5940.888467	9812.430075	9044.560381	11369.8637	11245.45688	9471.787873	10205.81066	5754.207788	8222.368983	5139.644528	9897.883289	10277.11827
Q8BP40	Acp6	Lysophosphatidic acid phosphatase type 6	157.119	18.9	47625	5	46960.80441	41635.53003	37555.29647	42942.39049	46706.63269	27386.30652	33642.64898	36445.56639	37809.58823	25429.84189	44825.54049	21083.32142	43619.12153	42204.68386	38611.74466
Q8BP47	Nars	Asparagine--tRNA ligase, cytoplasmic	679.96	38.1	64279	18	343189.622	373948.3963	360694.2893	390224.2326	342030.77	387312.849	425720.078	409562.2465	424401.3301	353508.6096	330088.2992	406040.3285	347614.2887	380780.6681	400825.1769
Q8BP48	Metap1	Methionine aminopeptidase 1	116.45	12.95	43221	3	12730.947	9526.42989	10511.61133	11983.35952	8674.444478	8017.584323	12622.7229	11653.86165	12873.53618	8139.785599	7817.692304	8412.175934	3978.269607	8275.70024	4879.995823
Q8BP92	Rcn2	Reticulocalbin-2	252.901	27.81	37271	6	11939.881	12662.3283	9173.873831	13236.96799	10151.85931	11005.18596	13676.79328	12885.53314	11689.97146	15032.06903	10643.52792	15932.12312	16916.28036	11101.6966	14866.41567
Q8BPU7	Elmo1	Engulfment and cell motility protein 1	416.403	28.61	83936	16	232907.6338	187308.2187	249314.6685	249498.618	245108.2838	150856.4966	160008.0327	137525.5195	154849.9323	140344.9525	176194.2239	166412.2172	187007.1917	180006.2188	183537.1326
Q8BPX9	Slc15a3	Solute carrier family 15 member 3	90.052	4.84	64051	2	5651.921171	7279.361593	6155.841469	7728.36009	4563.396804	17078.43931	6093.703758	5561.679662	6713.007879	8962.134777	21404.95603	15355.60209	7407.322552	13036.51924	0.010360417
Q8BQM4	Heatr3	HEAT repeat-containing protein 3	50.04	3.53	74305	2	1110.664269	910.9643608	1086.574402	4886.062518	3265.700347	941.5557539	894.8463761	1169.031508	1397.338328	960.1958633	1118.171118	1421.020042	796.1271442	1023.596989	1023.092335
Q8BQZ4	Ralgapb	Ral GTPase-activating protein subunit beta	89.737	4.25	165200	3	6300.58725	5735.135666	5613.302484	5738.373303	5306.070798	9850.227024	11588.88395	6325.992736	5894.368627	9726.169043	6472.150696	10157.28279	5610.912374	5524.965706	10433.71529
Q8BRH0	Tmtc3	Transmembrane and TPR repeat-containing protein 3	56.312	2.93	104198	2	4820.771324	4969.508192	3863.74276	4330.508563	4008.509942	4506.691672	4707.715285	5554.432718	5160.8285	5576.568245	4974.650418	4099.58338	3983.404454	5166.985332	4477.051402
Q8BRN9	Cc2d1b	Coiled-coil and C2 domain-containing protein 1B	135.932	6.96	93091	4	14915.36808	22572.31163	15196.94677	18072.26501	21383.39684	16128.2507	14005.87703	14222.09615	13491.30236	6983.664105	21526.48113	21801.20093	18078.03519	14559.48283	16859.79868

Q8BSY0	Asph	Aspartyl/asparaginyl beta-hydroxylase	677.992	35.76	83042	22	480257.1503	484722.2683	459677.9228	463956.6043	439473.0795	470617.9319	478243.7199	467542.5188	499507.2163	504799.186	522261.1298	494961.781	540314.5477	496406.4341	499874.324
Q8BT60	Cpne3	Copine-3	129.342	10.32	59585	4	31486.07843	29807.12537	31138.30135	32155.7167	30938.69258	32161.32387	34731.88936	35564.3519	31265.88745	35330.34388	28783.76536	31987.82212	29810.55934	35706.43385	33459.23232
Q8BTI8	Srrm2	Serine/arginine repetitive matrix protein 2	362.852	6.4	294840	11	81824.62641	108723.8145	82488.25377	80198.53164	99899.01373	10121.2989	8460.210529	9875.917237	7268.343001	11369.44387	43393.58736	55118.75671	47630.34488	42965.90501	67847.39514
Q8BTM8	Flna	Filamin-A	2308.771	33.81	281222	61	804175.7509	812288.0927	848734.9608	834253.12	744851.9647	576862.8925	878827.3799	835535.712	675974.9803	584159.048	838075.0381	951888.6277	780876.9994	817627.2077	873837.251
Q8BTX9	Hsdl1	Inactive hydroxysteroid dehydrogenase-like protein 1	95.987	11.52	36868	2	11965.95128	14214.86294	9983.026347	10454.35312	10478.02597	12360.26531	9975.960225	12604.14433	4812.527531	11702.84482	12822.3961	11055.53563	12725.12225	11600.53979	10091.231
Q8BTY2	Slc4a7	Sodium bicarbonate cotransporter 3	98.025	3.87	116514	3	11727.26005	16816.78958	11019.89503	10846.65739	14228.13664	13561.37815	16087.22777	15873.6156	16752.6553	15692.58046	13927.7611	10914.11941	12741.94177	16274.28008	17004.0377
Q8BU30	Iars	Isoleucine--tRNA ligase, cytoplasmic	642.093	25.28	144271	24	217169.3874	248689.6245	265762.8886	254904.415	238075.691	251909.4839	281386.3463	277106.123	269037.1047	261698.8217	247139.6776	281649.8985	257170.0497	244012.3801	260894.9862
Q8BUM3	Ptpn7	Tyrosine-protein phosphatase non-receptor type 7	193.124	21.73	40351	5	63894.37018	62880.6585	54646.19204	55511.87409	58366.47397	36566.29059	44942.40731	37095.65284	39410.35908	43901.36467	55174.29986	53352.74648	44441.78412	54076.86291	54465.5702
Q8BUR4	Dock1	Dedicator of cytokinesis protein 1	77.53	1.88	215085	3	7462.321637	8096.330761	7483.886607	6717.119215	7549.481741	5507.214382	6892.118677	4882.161074	5265.260682	8191.849148	5986.11469	5703.726325	6900.10136	6971.616019	6237.176731
Q8BUV8	Gpr107	Protein GPR107	32.332	3.99	62056	2	10100.11868	2908.921013	11035.44374	7479.083165	10275.99701	10804.53498	4230.308212	7064.881552	7675.632467	9937.833473	9217.29882	8507.332126	6920.537675	0.00945634	3532.498018
Q8BV13	Cops7b	COP9 signalosome complex subunit 7b	92.903	14.02	29689	2	5849.850913	4558.171387	2800.757471	4620.614095	4414.422799	3499.648152	3505.548915	4401.512512	4170.361191	3659.934876	5191.25001	4338.553181	4178.890576	3542.055216	3515.59562
Q8BV66	Ifi44	Interferon-induced protein 44	59.537	5.92	47852	2	3572.228534	4207.632131	5088.488179	4626.646663	4190.974149	10169.55373	5064.824698	9368.21027	5549.840333	5386.793078	4738.593575	3919.878875	4023.411801	4855.695635	4584.264826
Q8BVE3	Atp6v1h	V-type proton ATPase subunit H	372.601	27.33	55855	11	128749.5928	142445.8513	131438.2525	126796.3865	135440.9838	108941.7444	138863.8887	124760.9279	134126.09	118371.7191	122452.7984	133011.5869	117452.044	133413.6609	99107.83883
Q8BVF2	Pdcl3	Phosducin-like protein 3	109.738	18.33	27581	3	16846.92807	16617.94342	17206.7549	16236.03925	15540.33762	14831.25515	19073.50094	15737.40257	15476.09683	11492.25933	10976.87103	12202.28297	14219.17479	14807.89023	14270.13484
Q8BVG4	Dpp9	Dipeptidyl peptidase 9	46.733	3.48	98001	2	12508.90175	5206.130508	11395.38698	11554.48317	8387.540151	8803.40145	9959.325287	10427.42706	12222.27939	9868.876531	10747.9765	11388.23114	9489.630202	10859.58593	10864.96634
Q8BVI4	Qdpr	Dihydropteridine reductase	216.144	31.12	25570	5	56862.94296	60012.16994	49511.25505	58479.56069	62965.0606	46162.98714	44529.02979	50815.44548	44626.53366	47213.1028	48603.19428	47786.63744	45325.77896	52407.27996	54345.51887
Q8BVI5	Stx16	Syntaxin-16	113.23	12.58	37080	3	12142.84937	7525.252103	10812.5396	14031.13627	9273.402048	9581.609682	6966.244221	10492.55235	11246.08673	11467.57369	6775.890532	7870.22436	12329.42328	11675.73187	12017.53679
Q8BVK9	Sp110	Sp110 nuclear body protein	177.181	17.98	50140	5	36975.71971	35942.57373	29376.33475	39674.86383	32650.75654	39294.41133	35841.11266	39792.40816	40683.79097	25818.28185	30740.48997	27967.06148	26316.84095	30785.96466	36615.94249
Q8BVY0	Rsl1d1	Ribosomal L1 domain-containing protein 1	153.633	11.06	50421	5	31353.82569	24900.7246	30332.05768	35595.4698	31837.64193	43307.36689	41517.76794	50188.49024	40785.521	30388.0517	33582.63266	36749.78447	31212.54727	31205.27294	30813.79836
Q8BWT1	Acaa2	3-ketoacyl-CoA thiolase, mitochondrial	15.445	7.05	41830	2	18602.9031	17837.99551	17389.89808	18753.18965	18712.06341	23943.71276	15594.51131	7523.997578	17437.25167	8033.848421	20911.16328	14351.39973	8761.672123	16970.77259	17207.29094
Q8BX70	Vps13c	Vacuolar protein sorting-associated protein 13C	977.048	13.18	420089	31	179066.7132	196490.6853	170489.9678	193807.5314	179382.8685	162483.1891	187353.2347	179852.6708	171987.4132	169238.0364	187077.346	191485.4053	191589.4006	192848.571	180419.0409
Q8BXQ2	Pigt	GPI transamidase component PIG-T	62.931	7.73	65705	3	8360.266242	13464.34801	11195.71438	9932.810797	14057.13049	15707.7179	14156.25829	17133.45037	20714.29339	12388.54806	9170.273525	8999.706485	13253.25072	14468.5944	14180.43694
Q8BY71	Hat1	Histone acetyltransferase type B catalytic subunit	107.581	11.06	49278	3	10006.67883	15097.9111	16200.61752	14993.57711	10792.81157	13267.38159	15127.96589	15978.55309	11676.44712	11332.27722	9823.66201	10262.51339	9812.808039	8874.736895	11871.03273
Q8BY87	Usp47	Ubiquitin carboxyl-terminal hydrolase 47	77.855	2.69	157455	3	6372.045312	11247.22544	7345.607652	6539.839489	6884.774429	8539.613139	8225.165783	8863.288352	9779.489809	9420.295926	8878.955875	8400.334241	9384.440144	8473.063503	8455.058939
Q8BYA0	Tbcd	Tubulin-specific chaperone D	47.165	1.59	133321	2	15775.72964	14036.13092	15547.46212	15248.39737	16598.22394	18395.03451	18532.76435	18765.0438	19571.33639	16063.54934	13423.50398	17375.57394	19198.19868	17049.11127	14937.39488
Q8BYI6	Lpcat2	Lysophosphatidylcholine acyltransferase 2	90.418	10.11	60254	4	16611.11605	15025.58226	12963.58664	11406.71377	12327.41168	25032.64559	19287.8936	24274.51422	23350.63768	19004.62829	9663.025275	16344.89297	18562.72283	16340.95792	17861.48261
Q8BYK6	Ythdf3	YTH domain-containing family protein 3	97.025	4.96	63961	2	5112.996463	7078.953796	6209.531515	7292.584737	5046.775473	4176.395192	6831.918322	6402.618085	6559.091267	5142.461289	6702.943168	6429.111417	6702.706883	5549.15682	6520.834578
Q8BYW1	Arhgap25	Rho GTPase-activating protein 25	346.993	21.91	73383	10	113991.3251	138791.1626	133020.0656	138527.6404	131914.322	100956.6102	116711.9363	118620.4769	105251.3628	110655.3215	116493.5628	122727.6805	108736.6341	121781.3798	126766.3075
Q8BZA9	Tigar	Fructose-2,6-bisphosphatase TIGAR	88.892	19.33	29191	3	12274.22497	5954.554923	8809.599438	10888.26669	9856.405478	8764.852807	11348.90216	14520.44368	9237.634491	11096.67142	10753.16644	10716.58503	11162.16783	8809.073255	12178.76301
Q8BZN6	Dock10	Dedicator of cytokinesis protein 10	425.27	10.65	245758	14	98845.32553	90304.49234	96947.18934	98145.36347	97151.68193	103445.315	138035.2567	126679.5923	94245.12528	108268.8369	68699.86665	79956.81189	87527.23364	76970.74621	108129.9383
Q8BZW8	Nhlrc2	NHL repeat-containing protein 2	86.826	3.17	78430	2	1796.244094	4968.761404	5005.347726	3973.260556	5766.511171	6155.591728	7113.333873	6665.634829	6832.963467	5503.957043	5746.369568	6281.784854	6978.433416	6312.818277	6723.519916
Q8C052	Map1s	Microtubule-associated protein 1S	332.994	17.37	102939	10	89470.88171	84768.6528	76889.53712	80509.29449	55628.0073	45890.1587	66700.53598	63872.63659	61507.3514	68838.93907	76419.10395	78780.86653	81905.9872	63174.98692	77919.81292
Q8C0C7	Farsa	Phenylalanine--tRNA ligase alpha subunit	361.809	27.76	57599	11	119070.1329	130570.9378	119459.0279	132227.1564	123444.8915	114259.6179	121057.105	122404.0516	113574.0372	114508.8916	128223.0961	136107.8946	118181.1139	119364.5096	121281.8552
Q8C0D5	Efl1	Elongation factor-like GTPase 1	45.671	2.93	125777	2	3943.704377	6063.93154	3629.571171	4288.703279	4774.835409	2789.73314	4505.319535	3528.768077	0.012083387	3994.953028	4236.445094	4365.880035	5228.632609	0.00945634	4732.133562
Q8C0E2	Vps26b	Vacuolar protein sorting-associated protein 26B	88.309	10.71	39125	3	21841.89719	25064.00093	22798.3296	25592.54248	24848.70942	13809.80247	26845.5302	22459.02373	14183.30131	16015.12879	21247.60827	12719.23166	19311.77642	26234.52439	14213.38177
Q8C0I1	Agps	Alkyldihydroxyacetonephosphate synthase, peroxisomal	293.425	19.38	71684	8	49446.40397	44764.85765	48454.494	31381.21929	40416.20658	52128.65593	48327.49451	48709.07915	53675.90294	52998.06996	39843.22887	46105.27995	45879.33416	48476.67202	44050.73547
Q8C0L0	Tmx4	Thioredoxin-related transmembrane protein 4	117.178	12.54	37131	3	37555.40077	37215.73289	33991.07213	33068.67484	34747.00937	37654.75815	37079.41146	35258.35556	38340.40235	39071.60809	33713.35281	34239.61763	35040.24237	37526.89743	34553.9787
Q8C0L6	Paox	Peroxisomal N(1)-acetyl-spermine/spermidine oxidase	163.299	11.31	55447	5	32476.06647	20494.72886	21502.57957	30796.70817	26682.78159	16184.81052	20177.39741	17761.37071	16036.99708	26473.95973	20767.05992	18771.33429	30773.10577	21106.63984	19801.80475
Q8C0M9	Asrgl1	Isoaspartyl peptidase/L-asparaginase	45.474	7.06	33950	2	10337.42275	7778.187494	16831.55061	12975.44251	8863.186067	11170.01734	7569.466439	11093.03235	6820.475286	8438.688062	13197.97286	10821.99791	11949.06962	13245.14264	10476.85799
Q8C129	Lnpep	Leucyl-cystinyl aminopeptidase	354.035	14.54	117304	11	93559.96266	98932.56586	92585.94119	95749.78866	96837.38091	94694.87836	95509.97059	99785.51876	106457.3936	99374.36199	108356.3703	103305.6889	98314.18418	104954.5799	96823.83318
Q8C147	Dock8	Dedicator of cytokinesis protein 8	289.793	7.52	238978	12	42690.54253	69636.42535	65566.46355	55182.30131	80137.60986	51257.59109	79099.93518	86824.19908	61637.45323	66122.83308	45339.92001	45942.03824	65393.86587	70270.91068	68800.64672
Q8C156	Ncaph	Condensin complex subunit 2	76.659	5.06	82303	2	8320.950528	10650.61796	10770.70785	10023.22183	11101.30066	0.014539799	9284.397084	8055.66893	8344.546049	8602.735355	952.5828971	8025.527473	8160.97596	9596.202415	8603.736772
Q8C166	Cpne1	Copine-1	129.587	15.11	58887	7	112982.2252	89374.17152	83224.30236	86501.84951	109436.7817	78504.32462	102304.3295	110005.8655	106689.89	85031.51107	107723.1894	92892.21983	109762.4457	93086.98721	87086.02578
Q8C2E7	Washc5	WASH complex subunit 5	200.055	8.54	134110	7	49610.87309	38646.32641	43761.20233	42683.78371	35246.7632	29958.11715	33938.42272	36976.49399	30175.52793	36337.82288	36236.9196	37871.11741	39630.16803	35129.3229	40330.56886
Q8C2Q3	Rbm14	RNA-binding protein 14	241.333	13.75	69449	7	143017.7107	136205.1038	129048.8192	131426.9317	145237.1508	102828.275	114624.9701	121871.5375	104335.3938	110082.5335	122767.9767	138000.859	127378.783	119817.2886	124790.7626
Q8C3J5	Dock2	Dedicator of cytokinesis protein 2	1416.391	27.79	211704	40	459771.8972	472366.975	466468.0419	443805.892	456673.9352	394835.3632	404129.8083	403873.2272	438267.2582	457048.2873	453473.8548	445887.1204	441938.992	479472.4372	447199.3135
Q8C3X8	Lmf2	Lipase maturation factor 2	188.349	7.12	79997	3	15035.62888	16576.59024	13992.45272	18360.7501	15206.16754	14383.37481	16282.56211	16592.65651	10619.52989	16976.82464	15978.0515	15285.86309	16530.01032	15635.18283	15404.07985
Q8C3Y4	Kntc1	Kinetochore-associated protein 1	30.626	0.95	250358	2	2229.843042	2428.133326	3827.434513	4548.633541	3386.019471	4020.785242	3431.646385	2130.001123	4172.617401	3732.601491	3324.849981	4126.018692	4187.062355	3963.016892	3733.049162
Q8C4Y3	Nelfb	Negative elongation factor B	57.729	5.34	65636	2	6424.964299	6160.24174	4748.464361	6534.933545	6079.508252	4895.449122	6626.263747	6083.568143	5843.561429	5199.181975	7627.237545	5971.60599	6794.718749	6582.455162	8003.451641
Q8C6G8	Wdr26	WD repeat-containing protein 26	252.889	19.81	70543	9	44765.30656	37399.51093	29636.56651	41778.88467	44822.10777	53325.53587	48024.91059	38136.94928	48273.83451	43281.21007	27266.86617	32553.05227	37898.16972	48695.6052	39967.54504
Q8C7R4	Uba6	Ubiquitin-like modifier-activating enzyme 6	193.004	10.64	117966	6	10268.89738	14740.45461	11623.89128	11061.81149	9704.049166	17227.21054	11920.30619	13312.07871	9008.273682	10852.1311	9460.151871	9708.935281	11612.60199	7418.961781	6706.297692

Q8C7X2	Emc1	ER membrane protein complex subunit 1	364.763	14.54	111605	8	41762.57489	49706.84337	38504.94019	38023.81755	35304.3597	41766.90059	46596.45763	48784.00995	49812.88342	48790.42938	38829.24708	44937.56303	46021.93581	41926.97481	32828.0084
Q8C854	Myef2	Myelin expression factor 2	124.366	5.25	63295	3	35245.42525	29242.02008	39434.75572	43490.87751	21701.89692	41486.31154	38401.44836	25621.06062	43403.80063	41032.49944	43924.86437	43974.93642	44081.55159	27763.71852	40921.87928
Q8C878	Uba3	NEDD8-activating enzyme E1 catalytic subunit	227.269	24.89	51737	6	24164.72019	26397.65941	19269.14692	24573.08031	19580.93951	15599.75989	22660.52737	17721.86749	19871.56626	12904.37476	17656.81048	19259.72576	17254.24549	19860.56851	19069.69109
Q8C9B9	Dido1	Death-inducer obliterator 1	38.162	1.37	247176	2	4975.462912	4326.181996	4762.192212	4129.429823	4468.212876	3361.280587	3090.552943	3825.319269	3473.452999	4075.00562	4378.258273	3944.537309	3629.190055	4008.630728	3564.919647
Q8CAQ8	Immt	MICOS complex subunit Mic60	1043.474	46.63	83900	27	481437.4978	491525.5687	488323.2017	419938.0948	505033.7727	505375.6423	422914.0602	510318.6162	428165.6434	457220.3308	452381.1187	492024.3268	497225.8898	435930.5324	484290.434
Q8CAY6	Acat2	Acetyl-CoA acetyltransferase, cytosolic	257.487	40.81	41298	9	93500.20656	109389.6852	90993.06806	103933.0727	88033.15005	98131.91247	79859.53304	70184.27166	77221.73565	65675.99409	104918.3921	96491.68897	70265.6687	81983.64639	80752.56322
Q8CB77	Eloa	Elongin-A	63.24	5.43	87161	2	3796.457376	2843.464297	3062.749183	3403.74763	2576.254889	1944.061838	2410.637501	2815.294284	2300.374056	2586.979501	1972.011798	2859.213908	2325.331565	2643.2186	2602.265994
Q8CB96	Rassf4	Ras association domain-containing protein 4	123.874	20.19	36934	5	25779.87887	29372.52658	26926.05697	27631.28341	31010.66754	24150.92853	30588.04708	21405.46216	33566.85432	27526.69573	28955.66105	29379.32624	28446.41961	30795.81344	30031.45222
Q8CBW3	Abi1	Abl interactor 1	106.175	17.88	52288	7	70652.89284	58943.02586	65984.0155	64882.6189	59357.26115	53309.52392	64785.34321	49272.54596	61756.70418	56933.709	58107.15992	60176.92666	55191.04834	68672.30229	61152.53017
Q8CBY8	Dctn4	Dynactin subunit 4	67.87	9.64	53057	4	13486.08653	14744.38991	21786.76919	22000.57427	14822.61102	19206.94781	17925.00641	15980.07712	14881.59571	18357.89825	12508.57262	13170.3133	15453.47529	14268.93797	19250.73808
Q8CCB4	Vps53	Vacuolar protein sorting-associated protein 53 homolog	65.219	5.41	94423	2	2392.726291	2238.147974	1940.457671	2649.843934	1555.008637	993.9566094	2036.120815	2334.795001	1853.713894	1498.035773	2569.87495	1938.047581	1985.42433	1727.107331	1558.802231
Q8CCF0	Prpf31	U4/U6 small nuclear ribonucleoprotein Prp31	53.378	7.62	55430	2	11683.99569	10649.26334	11920.50442	13300.65901	11516.61461	8142.350342	5141.173708	6854.789195	6777.48181	5636.179012	15772.57005	10735.01861	14850.0306	10521.51825	13716.10379
Q8CCH2	Nhlrc3	NHL repeat-containing protein 3	234.33	30.55	38195	6	49770.50513	36033.57228	39758.81567	41520.31916	42374.95477	29333.70364	30735.9818	29822.28322	36820.12801	32224.54916	39257.98854	37112.74107	41728.58999	46839.30209	42283.05672
Q8CCJ3	Ufl1	E3 UFM1-protein ligase 1	95.437	6.81	89520	3	8912.985486	3505.601737	3652.139061	8608.982622	6040.23199	7353.353651	8685.367172	7160.168015	8432.621856	6639.420525	4964.570265	5554.531722	6308.026445	6266.231052	4028.980839
Q8CDG3	Vcpip1	Deubiquitinating protein VCIP135	106.969	6.07	134503	6	30471.78157	26814.84953	25533.50073	33277.59946	29713.5559	19421.03182	33518.41407	31517.57165	24704.6567	19770.98859	34957.32545	28868.37791	33187.61792	19660.72177	22123.27108
Q8CDM1	Atad2	ATPase family AAA domain-containing protein 2	65.863	3.65	117943	2	3150.17895	2885.313987	2017.524377	3048.528133	2729.052639	1286.524054	2015.041785	2260.822155	3012.101373	1932.282125	2460.667155	3104.849733	2054.485513	2046.485462	2160.977309
Q8CDN6	Txnl1	Thioredoxin-like protein 1	250.07	38.75	32237	6	73634.28129	75743.27265	72967.60436	71121.29425	65886.3474	67711.40244	67856.55073	71738.85656	64627.14294	66832.12637	68333.36022	76110.94273	71538.52629	63932	73206.86975
Q8CFE2	Hpf1	Histone PARylation factor 1	88.835	12.14	39291	3	13190.88449	13329.27192	12784.99395	15610.56425	12019.98062	11215.27705	13024.12394	13760.1221	13765.03336	12184.66096	12157.0436	12378.06545	13705.18115	12989.76828	13865.24976
Q8CFE4	Scyl2	SCY1-like protein 2	56.996	2.69	103317	2	5421.266533	4996.217699	4000.980379	5119.617283	4885.090065	3597.500348	5679.37269	3536.665944	5834.853415	4829.068927	4838.881147	5777.92656	5002.508521	4706.923772	5627.662386
Q8CFI7	Polr2b	DNA-directed RNA polymerase II subunit RPB2	67.151	5.79	133911	5	26750.11754	38256.11332	36910.52407	35538.98268	37814.58534	25388.79435	39863.43669	37545.46674	41170.55813	41075.42041	20106.87925	34043.2471	25657.81851	26046.21557	31964.02994
Q8CFQ3	Aqr	RNA helicase aquarius	80.653	5.33	170294	5	21534.96027	23163.42974	14724.18381	21601.24149	22274.49776	23785.40342	16135.91626	22121.73794	24210.21659	20589.04621	23394.56435	16835.51137	22685.93936	22614.25933	20232.33705
Q8CG47	Smc4	Structural maintenance of chromosomes protein 4	385.276	15.86	146895	15	92887.97801	81180.65711	103219.9309	95833.41995	96912.34398	106834.3605	84532.6834	82413.93989	100586.5713	77075.04374	97105.42206	92528.76508	95447.35527	71735.94355	82420.31575
Q8CG48	Smc2	Structural maintenance of chromosomes protein 2	249.947	12.51	134239	11	76598.59187	90402.48613	97341.21375	94576.0433	88502.13009	88372.45741	90005.21224	82956.04254	85284.67764	85037.56509	77478.36698	91183.46896	80597.28297	81318.32671	80996.54585
Q8CGC7	Eprs	Bifunctional glutamate/proline--tRNA ligase	1682.65	42.59	170079	42	705488.4204	728477.288	674987.8558	715873.7615	663928.4783	634408.627	729055.3554	710017.9081	700355.2433	636271.3489	660623.1989	726250.2954	672309.4778	692471.4939	683860.6946
Q8CGK3	Lonp1	Lon protease homolog, mitochondrial	813.421	32.67	105843	22	248898.478	262492.3339	224424.1523	236142.6481	223917.9113	266883.1509	278136.8453	262308.0962	302842.9895	268949.8786	264337.0032	268954.6593	262685.7528	259069.2747	263356.9113
Q8CGY8	Ogt	UDP-N-acetylglucosamine--peptide N-acetylglucosaminyltransferase 110 kDa subunit	158.065	6.41	116952	5	30531.53355	28732.48342	29969.81779	31105.87983	27555.78855	24966.76395	17114.38311	26583.14926	7882.753801	24896.23521	21839.1932	29207.08397	29397.04741	29784.55721	28539.78792
Q8CH18	Ccar1	Cell division cycle and apoptosis regulator protein 1	127.484	5.06	132060	4	26007.32882	26339.03745	16194.45271	24657.13806	33514.59046	23589.31242	21607.05589	23626.0438	22049.29316	20925.85379	28538.85645	26199.9191	23349.25682	20511.77934	25355.99303
Q8CHH9	Sept8	Septin-8	122.925	14.45	49812	5	58332.72996	49967.51606	38389.39231	59642.84182	55560.4959	57505.28474	67949.38038	56730.441	60270.65905	43425.98052	60891.20947	54798.96653	63182.93685	65589.49302	60253.74331
Q8CHP8	Pgp	Glycerol-3-phosphate phosphatase	163.498	22.43	34541	4	19190.3128	20113.15584	16460.03142	19919.89848	13977.75758	9260.0602	10772.09185	9005.213889	14682.17602	9588.825462	16258.00459	15882.96312	12052.28633	16767.54403	16145.28219
Q8CHT3	Ints5	Integrator complex subunit 5	99.327	7.37	108348	3	6696.944919	4245.816658	5357.119573	7016.407315	5818.471393	6349.455454	6216.912634	4701.299087	5742.06847	7202.151801	2333.304141	6101.851339	6195.86589	8283.855718	4248.819815
Q8CI43	Myl6b	Myosin light chain 6B	57.825	11.59	22749	2	340752.5208	302173.6903	303920.3102	296447.6986	267333.0955	270012.8137	245476.5391	209171.5944	239148.2189	216633.4725	333381.6027	287068.4528	290002.201	247703.4542	258333.4768
Q8CI51	Pdlim5	PDZ and LIM domain protein 5	229.892	18.95	63299	7	45987.68241	46746.27484	42550.94646	40912.71979	41676.39464	38788.33181	40958.79883	35517.37782	32010.47179	44257.86615	43210.35829	34590.09348	40233.50479	42601.661	41646.55416
Q8CI75	Dis3l2	DIS3-like exonuclease 2	36.81	2.07	97775	2	11905.50931	7060.283099	13229.22716	13411.50326	13429.73526	7085.213797	8868.153917	0.01388636	11823.38111	6677.338102	13293.91838	5636.141258	0.00981159	13781.27572	5355.796351
Q8CI94	Pygb	Glycogen phosphorylase, brain form	767.631	34.4	96730	24	291358.1484	293755.8444	275524.952	273095.6476	286909.4598	232596.4282	267607.499	239850.3257	254111.9469	243516.2072	283694.3101	270323.7938	297614.8569	294293.6101	275057.9658
Q8CIE6	Copa	Coatomer subunit alpha	1273.274	38.24	138432	36	444278.3514	423586.762	450995.9388	421138.4822	399999.6355	454192.4659	484314.2297	480355.9627	486027.3282	415226.3636	458454.7181	437098.3065	441118.6646	447207.2552	431858.4789
Q8CIG8	Prmt5	Protein arginine N-methyltransferase 5	46.897	5.81	72680	3	16539.53665	15212.29642	14578.54893	15120.16929	14088.48806	13469.59472	15199.7877	17769.17311	20514.82386	12962.0878	12542.62104	13256.25442	15450.19086	15230.4444	18282.82133
Q8CIH5	Plcg2	1-phosphatidylinositol 4,5-bisphosphate phosphodiesterase gamma-2	681.111	21.5	147592	18	144835.7144	143675.1673	149416.2189	160784.6066	135914.2869	127899.7527	138452.7586	135206.2197	141791.6335	131454.5803	136229.1963	136820.684	141946.9144	135632.7423	144404.9687
Q8CIM5	Gpr84	G-protein coupled receptor 84	233.413	22.22	43717	6	28255.63973	35677.23882	34779.64162	32140.74716	29542.90724	64560.18444	70997.71852	62114.65125	64772.64504	51321.33816	21871.47947	36214.06028	28629.42539	33355.66821	30574.21631
Q8CIM8	Ints4	Integrator complex subunit 4	63.958	2.9	108193	2	4918.46317	4251.686076	4071.796598	4395.067996	3694.482232	2758.394856	3429.882837	4149.235546	4103.950171	3482.139152	3669.681359	3548.431181	3923.23733	4130.194338	4097.633467
Q8CIN4	Pak2	Serine/threonine-protein kinase PAK 2	429.749	35.5	57930	12	127795.5847	121085.498	128172.9938	119807.5286	123300.4456	113825.7939	125177.087	113682.4319	116230.7269	108716.3152	115462.5749	119198.6851	126875.0952	126795.1216	126736.5421
Q8CIN7	Impa2	Inositol monophosphatase 2	46.609	11.38	31796	2	33859.15406	29501.70776	35279.03353	28421.80961	32182.07614	23250.63324	25006.67108	23108.70921	27445.49208	19455.60986	32002.8869	26485.92608	26407.43626	30073.18124	28283.62662
Q8CIZ9	Nox1	NADPH oxidase 1	27.213	2.54	68193	2	5800.63427	7028.694435	7187.498142	6506.643533	5208.903559	9594.795463	10118.17297	7607.02676	9629.313731	8022.153297	5035.570877	5142.224374	7381.625997	7952.647423	6416.120552
Q8JZN5	Acad9	Acyl-CoA dehydrogenase family member 9, mitochondrial	307.353	23.04	68722	11	118336.8867	113780.5902	109029.4254	103995.3629	98294.28814	111858.9589	108056.5821	111443.798	117997.2214	119532.9331	107486.6166	95012.7454	109965.8007	113422.751	109995.0516
Q8JZQ2	Afg3l2	AFG3-like protein 2	41.803	3.62	89519	3	5566.285555	14817.39085	5357.486893	0.009171104	20463.19183	6639.79482	9205.142231	15434.85351	15071.29431	17968.17456	12522.03931	6237.945938	6402.17974	10637.61136	5848.128841
Q8JZQ9	Eif3b	Eukaryotic translation initiation factor 3 subunit B	736.872	34	91370	21	209496.6351	222353.0581	224249.9306	227007.8987	198617.0925	200532.2593	232140.7798	246645.7824	207004.8872	196308.9719	202287.9299	231492.5088	204777.5906	209192.8516	207407.574
Q8JZR0	Acsl5	Long-chain-fatty-acid--CoA ligase 5	333.003	24.6	76206	12	120892.513	137210.7992	123231.1937	134771.9726	132255.0573	152219.4429	141430.8895	140741.1005	148474.0727	137370.2484	120803.3948	131367.9479	131084.1889	136587.6908	134819.6355
Q8JZU2	Slc25a1	Tricarboxylate transport protein, mitochondrial	214.73	34.73	33931	7	108786.095	107881.1799	99678.79901	108868.6214	106860.4292	103291.1567	107585.6607	97469.75307	103105.1237	92765.39141	117323.6396	107126.4969	114935.4889	102199.9104	99816.49133
Q8JZV7	Amdhd2	N-acetylglucosamine-6-phosphate deacetylase	362.215	38.39	43501	9	98920.88442	88838.17775	83814.98235	79251.15663	79309.5857	100310.3597	95647.10657	93340.30385	84682.74372	76860.14917	87188.17616	88040.45349	89530.15203	90318.04804	88503.05104
Q8JZX4	Rbm17	Splicing factor 45	45.392	7.41	45304	2	9080.426372	11885.10859	11278.75022	9925.171268	8502.12095	5594.59087	5050.142362	6513.92288	4926.926997	5546.840119	9603.809652	8435.463566	9210.235361	8416.023478	8591.529611

Q8K0F1	Tbc1d23	TBC1 domain family member 23	84.48	7.02	76426	2	2564.347965	2407.923261	2520.918354	2414.169223	1948.094287	1471.698505	165.4972135	2033.787953	1908.446381	1122.350224	1596.14626	1765.917127	1735.664023	1543.044267	1597.440421
Q8K124	Plekho2	Pleckstrin homology domain-containing family O member 2	332.694	29.9	53872	8	116519.4096	123039.6943	99286.47688	107105.3666	96257.58877	96932.19727	103673.059	102655.0655	75761.70001	94471.46759	113371.3343	108487.9963	111402.1194	98695.62734	109214.1559
Q8K183	Pdxk	Pyridoxal kinase	413.942	39.42	35015	9	149139.1243	139854.6803	126631.4172	145781.644	131010.8554	82294.545	109052.7459	97172.96685	88095.24322	90739.55748	138206.5881	131846.4014	131445.1181	125331.9894	126466.4356
Q8K1B8	Fermt3	Fermitin family homolog 3	1135.748	60.6	75635	31	571655.7503	601277.3953	574280.2264	615803.1541	588158.1267	551190.2013	597237.222	596810.6396	571924.839	555531.9417	580120.6892	595209.2113	569096.2588	595136.6716	577521.3368
Q8K1J6	Trnt1	CCA tRNA nucleotidyltransferase 1, mitochondrial	174.989	18.89	49895	6	175615.3871	119279.1016	193650.687	144945.1385	139059.4628	157519.1707	242321.9485	251072.2539	159751.4252	199445.9159	159922.4297	141381.634	153164.0835	180107.9879	139749.5776
Q8K1R7	Nek9	Serine/threonine-protein kinase Nek9	167.947	5.39	107143	4	28379.5448	30614.05404	25944.93067	25521.93415	25090.90115	24278.31171	28502.35893	32237.55162	29385.12943	28554.02513	27110.03941	28046.85732	27359.23655	27421.3943	31378.90987
Q8K1X4	Nckap1l	Nck-associated protein 1-like	570.387	23.99	128905	18	193126.2936	164077.0675	154525.5457	154760.8423	168936.3603	144134.2385	153657.7435	146816.4244	161752.4945	180101.1416	186674.5744	164305.4857	167446.6878	171878.8164	139940.8309
Q8K212	Pacs1	Phosphofurin acidic cluster sorting protein 1	36.415	2.08	104829	2	2829.423605	7100.939629	9248.395921	9413.054548	4475.086434	9421.422894	4392.460656	9615.753723	6016.108025	5926.225683	7784.432947	9546.076232	9082.238261	6605.043993	6687.718875
Q8K224	Nat10	RNA cytidine acetyltransferase	109.584	5.47	115419	4	31258.39403	30224.56883	30517.58666	31820.39593	29815.29209	27793.36638	28841.32146	32186.98791	30013.77065	30485.82187	32518.12739	34627.13046	32931.71202	13973.61531	32009.94213
Q8K297	Colgalt1	Procollagen galactosyltransferase 1	282.896	14.59	71061	9	74653.51602	84526.67818	86467.96403	89227.96924	81973.54805	109640.1055	105960.7621	109650.4233	117292.9774	102650.775	77090.94791	79822.3493	94144.31722	85230.55625	96635.62585
Q8K2B3	Sdha	Succinate dehydrogenase [ubiquinone] flavoprotein subunit, mitochondrial	743.411	50.15	72585	23	505170.7056	514538.7862	467933.9006	477511.0185	496186.1627	536133.134	508382.3936	496023.22	527624.2426	488495.4903	480231.2941	488730.3104	510738.5687	528135.2904	500478.0462
Q8K2C9	Hacd3	Very-long-chain (3R)-3-hydroxyacyl-CoA dehydratase 3	128.075	17.68	43131	4	21485.25264	21246.40652	18919.19028	14610.19288	20270.35141	22901.32183	23855.33331	22669.32396	25329.45843	22406.5291	19499.15521	20002.52379	21118.32688	19268.81796	14824.70722
Q8K2I4	Manba	Beta-mannosidase	232.516	13.54	100831	8	112359.434	61360.88897	103182.6996	78292.89553	96797.63039	57797.70919	40421.56302	33890.04923	58846.52919	64032.74257	64226.55552	80402.0075	77498.86128	82435.65943	92999.63763
Q8K2K6	Agfg1	Arf-GAP domain and FG repeat-containing protein 1	163.311	18.72	58043	6	43169.74302	49913.48638	42428.08783	40372.41797	40912.9847	42935.27162	43163.85967	43909.08887	44802.12943	40878.73786	46548.82636	44557.54726	47099.12086	42198.95332	50079.16664
Q8K2L8	Trappc12	Trafficking protein particle complex subunit 12	43.084	3.51	87694	2	6412.963597	8517.984592	9435.564173	8377.677071	7608.811514	7123.66844	8427.782633	8212.825235	6783.752363	8766.266424	2265.750938	9046.388217	6803.492763	7657.619698	9091.983061
Q8K2Q9	Shtn1	Shootin-1	63.504	5.86	71343	4	53461.02425	32069.29347	46929.49315	46350.7211	45349.43651	45124.08575	44736.47798	43139.60751	34527.48056	48027.60226	57455.26706	42105.98251	48561.32252	54487.43827	42770.03256
Q8K2T1	Nmral1	NmrA-like family domain-containing protein 1	62.407	14.89	34376	4	14387.66204	19658.84749	20791.16642	18177.97115	18115.13821	11273.85513	14742.30761	13984.13859	16843.34716	14477.6279	20010.22364	18984.09602	17266.59092	17528.71081	16543.8526
Q8K2V1	Ppp4r1	Serine/threonine-protein phosphatase 4 regulatory subunit 1	82.607	1.89	106298	2	4521.24737	4611.878718	3049.814173	3990.246311	2760.089906	2517.412729	2957.668297	4058.962892	2777.680103	3167.423096	6342.476589	3160.787881	4004.372705	3089.465961	3796.546498
Q8K2V6	Ipo11	Importin-11	82.276	3.9	112416	2	4661.637515	5932.551583	4698.223753	5116.928407	3721.570237	3307.327753	4656.129164	5974.403097	4549.278502	3815.2029	3301.52743	3577.156822	3830.329813	4205.184577	3434.764996
Q8K2Z4	Ncapd2	Condensin complex subunit 1	476.331	15.52	155665	14	43685.21341	48197.05245	51325.62843	58559.34012	41950.95754	86048.05238	52915.98374	56644.07131	50146.53896	44165.19761	47036.43544	40285.01343	45388.8773	36376.77249	44388.45915
Q8K310	Matr3	Matrin-3	360.405	18.32	94630	9	80427.07898	77580.63717	65754.09898	68606.07781	65263.88276	62371.55205	75626.12419	75860.69605	78663.38641	64259.23237	61352.61538	76516.21439	69170.50391	62905.08407	69917.46337
Q8K337	Inpp5b	Type II inositol 1,4,5-trisphosphate 5-phosphatase	68.502	3.12	112762	2	7373.446901	6318.45882	7115.769589	7942.064551	6307.079251	5693.751866	7039.195919	9185.333025	5453.666361	4475.773414	8303.80274	7620.722849	7074.322087	7500.377934	7679.959287
Q8K363	Ddx18	ATP-dependent RNA helicase DDX18	179.236	14.24	74181	7	33917.9847	36834.2317	32499.24662	38881.49917	41197.15253	31981.78279	40409.59118	38325.66491	42313.57101	39258.74339	33913.77306	41534.66142	37201.73324	21110.75334	35903.09143
Q8K385	FRRS1	Ferric-chelate reductase 1	71.809	4.9	66047	3	56996.97876	61699.80619	64644.43426	69214.64932	64456.36621	53864.42446	62475.70931	60589.71373	61189.81208	49192.97542	69310.54171	69994.56597	60841.75898	68258.39357	67667.57457
Q8K3G9	Appl2	DCC-interacting protein 13-beta	215.632	25.23	73854	10	51863.70027	62890.45561	51895.09179	49758.16212	55630.99954	34938.909	45103.45611	37731.23152	34859.69817	40589.48667	55003.14041	46797.56846	47150.1166	48530.83321	47251.77268
Q8K3H0	Appl1	DCC-interacting protein 13-alpha	49.011	5.09	79328	3	9384.907722	8601.382912	7083.875133	6636.68749	6123.418561	9336.014295	8655.609696	5374.201658	10518.10194	9520.898303	9313.587321	6164.762368	8437.788489	6516.896724	9131.926353
Q8K411	Pitrm1	Presequence protease, mitochondrial	259.001	16.89	117372	11	77759.59926	72832.43835	62780.66161	75758.73617	67601.10811	47146.34649	50959.30319	62133.36312	72672.98694	71057.67595	65555.82827	61618.29207	61523.43883	66607.77531	61947.37097
Q8K482	Emilin2	EMILIN-2	48.044	3.72	117310	2	8384.750366	7328.319235	7349.226176	6917.575466	5991.987271	6070.020501	5601.898363	5559.177965	6369.449277	5251.723852	6820.887083	6530.434992	6846.758197	2755.49963	5471.89031
Q8K4Q0	Rptor	Regulatory-associated protein of mTOR	79.476	2.17	149471	2	7377.323027	5411.639155	3216.056755	5605.488597	4798.043875	5600.259066	7012.852795	6057.934757	6287.270162	6556.0245	5491.17029	6298.918146	5350.322671	6400.888713	5375.914001
Q8K4Z5	Sf3a1	Splicing factor 3A subunit 1	441.401	30.21	88545	17	190245.4145	201180.4267	184943.2164	190131.588	191898.9825	191117.6374	185529.0316	196433.1507	171303.4452	174753.5661	190299.6962	208104.9306	198065.9527	185022.1261	191942.2071
Q8QZT1	Acat1	Acetyl-CoA acetyltransferase, mitochondrial	549.109	47.88	44816	11	258523.9978	263487.2151	238026.0161	237171.4075	242409.2329	226244.8186	211845.1488	191554.5671	218020.9969	214314.0684	242795.6564	208694.6785	226282.3985	229683.1887	219650.1054
Q8QZY1	Eif3l	Eukaryotic translation initiation factor 3 subunit L	688.491	35.64	66613	16	136555.2169	144630.5878	138325.7532	147879.7816	139203.0407	157889.3394	182109.1464	183024.1765	162220.4841	150905.3699	161761.8576	149998.851	170088.3211	152861.0016	148685.2194
Q8R010	Aimp2	Aminoacyl tRNA synthase complex-interacting multifunctional protein 2	133.343	31.56	35378	6	66122.29624	63656.50563	65336.64688	87176.19159	48759.92164	46157.01491	46681.14038	66588.59327	72338.73025	73053.63935	70390.59669	66694.8073	63453.4473	68415.76125	55839.74478
Q8R016	Blmh	Bleomycin hydrolase	195.995	21.98	52511	8	153718.7745	165277.6313	175038.5803	168977.8505	151988.3076	144436.2737	170841.0897	148307.4167	148878.1654	161683.1061	166666.65	157719.862	156235.2486	153130.2614	169605.1799
Q8R050	Gspt1	Eukaryotic peptide chain release factor GTP-binding subunit ERF3A	258.743	20.75	68625	9	56077.003	55649.86985	56804.61997	49982.34975	46693.94046	34143.42567	49371.5086	45547.4467	49185.3448	43449.88026	38205.70176	50941.16909	51393.53614	49378.90611	37555.45874
Q8R059	Gale	UDP-glucose 4-epimerase	79.096	18.73	38225	3	10019.69392	10277.19249	7824.04209	10642.07185	8679.61064	8320.793665	11099.44372	12975.91689	12990.15446	11256.93658	7373.478576	9316.824809	10839.69397	8620.019749	11592.97987
Q8R0G9	Nup133	Nuclear pore complex protein Nup133	140.219	7.53	128620	5	13553.08592	15489.58751	15016.92047	16680.18434	12517.36422	11582.30884	13681.24491	13417.35641	14212.67753	14638.30765	12043.95216	12912.07897	11365.59643	13412.97966	12137.26954
Q8R0H9	Gga1	ADP-ribosylation factor-binding protein GGA1	67.638	9.76	69972	2	6818.885451	4694.150238	4524.694196	4851.76658	4014.258598	2435.286417	4172.202467	3465.699009	3807.261094	2978.703687	5621.207492	2443.063199	2345.612122	3356.16131	3568.517975
Q8R0X7	Sgpl1	Sphingosine-1-phosphate lyase 1	131.613	14.61	63677	7	94893.84648	102514.4209	71698.36323	91391.56098	100743.644	113560.6098	103269.017	111904.0197	99048.42865	76581.684	101432.1194	94428.90279	101052.8933	92107.99129	101831.6234
Q8R146	Apeh	Acylamino-acid-releasing enzyme	216.699	15.71	81581	6	41587.41564	44727.19682	43453.48517	42305.73706	43284.17446	39456.79545	44801.86932	43697.56143	38780.63035	39047.83155	44936.1828	48237.38057	42898.11683	41463.91204	43382.2244
Q8R164	Bphl	Valacyclovir hydrolase	36.1	8.59	32851	2	8573.04349	8056.360558	12111.49566	8226.545307	9120.129259	8956.943549	8754.120389	11267.51362	9860.489283	8787.835137	7414.094897	7084.096884	8205.577585	8869.188955	9165.968092
Q8R180	Ero1a	ERO1-like protein alpha	524.829	53.88	54084	17	189975.0642	236595.4009	196446.812	193284.7602	181054.3244	290282.9516	290523.7973	291765.7306	309303.1307	241104.2943	202418.6521	191573.2597	218040.0937	211001.2064	224973.619
Q8R1A4	Dock7	Dedicator of cytokinesis protein 7	31.519	1.22	241438	2	10727.95568	10584.85141	11100.43214	12311.61854	11017.6326	2504.95801	10240.07476	14543.5862	9345.091325	11813.27083	9992.409536	10381.07274	9749.182967	9985.407622	13355.90836
Q8R1B4	Eif3c	Eukaryotic translation initiation factor 3 subunit C	573.956	22.39	105531	18	283038.3948	310194.0557	306077.3878	297600.3413	296748.7085	300554.2439	330193.1715	344189.9872	314147.5055	297478.7619	288070.9837	282323.0628	289163.2512	315744.0305	302300.9507
Q8R1F1	Fam129b	Niban-like protein 1	362.851	22.16	84819	12	109475.3056	100460.8393	91240.16424	95042.96253	77326.6888	65228.95705	85255.01974	79370.35539	87819.82054	78342.89243	95643.74143	97849.21225	90916.43161	91772.31014	91141.09285
Q8R1F5	Hyi	Putative hydroxypyruvate isomerase	61.211	14.08	30449	2	8560.788841	6971.688945	6429.153548	5929.019362	4586.095349	3606.332549	5451.435726	3939.67344	4613.528468	5060.813139	6111.892318	6732.042832	6563.617192	4945.455778	5241.834358
Q8R1I1	Uqcr10	Cytochrome b-c1 complex subunit 9	85.36	37.5	7446	2	25391.08992	28198.18362	30041.94576	28178.2251	28201.44306	37484.54072	29807.54016	34703.93571	35285.83392	33827.3839	28722.07546	25939.00725	31597.48293	32364.28448	28222.46649
Q8R1Q8	Dync1li1	Cytoplasmic dynein 1 light intermediate chain 1	350.415	34.42	56614	11	105295.266	84293.63478	92822.04391	85219.02305	78862.89869	96823.87576	83796.75139	77117.94621	83312.60244	61421.73285	105868.6342	91554.09698	67290.77984	90941.51397	79605.1419

Q8R1S4	Mtss1	Metastasis suppressor protein 1	214.905	13.31	82408	7	100059.3023	97358.44146	87147.31449	91569.53542	91931.43658	74756.02976	74765.98505	80694.12121	70163.26144	81696.54919	94061.58979	83487.22924	91192.93203	81916.13759	100672.9455
Q8R2E9	Ero1b	ERO1-like protein beta	79.667	7.28	53518	3	52470.13337	58413.07067	41372.93872	47635.73029	56678.16815	77127.03485	66593.78959	71285.22391	65436.96549	49450.35473	56217.289	52551.21957	53242.51069	54917.82465	57131.64729
Q8R2U0	Seh1l	Nucleoporin SEH1	78.308	14.17	39775	4	20948.22308	22548.30852	22172.43438	19565.74943	21432.76468	23024.36306	22689.31938	22111.13397	24253.97507	21155.28735	23743.70056	25779.77785	22343.76751	25304.7558	23538.30456
Q8R2Y8	Ptrh2	Peptidyl-tRNA hydrolase 2, mitochondrial	212.469	27.62	19527	3	27514.39962	22660.61418	24407.86886	25005.78903	18007.10344	24621.16564	25210.1693	19864.229	22796.9497	31544.99318	27883.77721	24120.61267	24655.89081	23339.96957	24267.11583
Q8R323	Rfc3	Replication factor C subunit 3	124.109	12.36	40526	3	13113.49627	11271.64826	14056.70886	11281.12926	13336.05828	12091.44987	12036.5856	11573.60544	11666.01765	11810.27299	11103.75604	13143.03427	12834.44912	13885.27063	9265.202752
Q8R395	Commd5	COMM domain-containing protein 5	63.053	16.52	24493	2	5593.635529	8291.6897	7208.166759	6012.036192	4561.928044	4034.26589	7386.262132	5808.867693	4862.347698	4788.630904	5027.246854	8606.506347	8583.759196	5071.222163	7258.722877
Q8R3F5	Mcat	Malonyl-CoA-acyl carrier protein transacylase, mitochondrial	70.672	6.82	41928	2	10485.81144	11284.49681	8215.505955	4943.293094	6752.95361	7123.779233	10765.36576	10607.56439	6659.014835	10137.2458	5665.89329	10870.86468	10694.69584	10369.69081	11228.11967
Q8R404	Mic13	MICOS complex subunit MIC13	98.273	22.69	13373	2	24023.67967	19569.22152	22021.83533	22823.77941	20753.1251	24522.42222	21672.3114	21547.55954	22539.19142	22967.12915	22874.67101	19643.41421	22007.79659	20810.78407	22417.38
Q8R480	Nup85	Nuclear pore complex protein Nup85	169.923	10.37	74776	5	11449.45876	15633.50974	15442.73891	13793.40178	13571.2757	14561.77909	14963.5722	15206.79436	16332.38302	10569.8872	11093.45662	8383.979276	15476.46262	11738.75365	16568.57903
Q8R491	Ehd3	EH domain-containing protein 3	259.129	23.55	60791	13	215506.7792	237170.3563	218514.5374	220474.1477	226824.6998	240314.2119	245482.6639	252397.9291	261071.2527	246890.024	224968.6983	239976.6761	236766.9264	238889.5405	242334.507
Q8R4N0	Clybl	Citramalyl-CoA lyase, mitochondrial	100.364	9.76	37549	2	13116.48654	13398.45196	11989.72354	13456.28462	9837.783531	11271.23615	11704.74888	11431.55145	13619.33641	14595.13193	12792.68671	11494.73654	12466.83828	12320.85755	10082.74323
Q8R4R6	Nup35	Nucleoporin NUP35 G	70.765	10.46	34786	2	5165.576648	5476.26616	9784.367737	5566.920071	3946.683631	5067.368147	7431.089439	5672.018939	3601.516903	8763.658132	4177.513107	5269.587237	8131.089218	4371.73937	3627.734542
Q8R4X3	Rbm12	RNA-binding protein 12	49.964	2.72	102796	2	5557.678752	4932.463208	5327.71196	5905.393407	4694.231382	5271.334343	5912.607211	5626.949024	4538.10439	4103.545386	4755.623342	6619.057033	5307.517156	5506.820787	4962.936449
Q8R5A3	Apbb1ip	Amyloid beta A4 precursor protein-binding family B member 1-interacting protein	44.92	5.22	74319	3	19354.64413	18555.50617	18304.78423	20365.48343	16284.73918	12570.59805	16867.6359	8830.310886	14856.39127	13598.00105	15888.27758	17714.68871	17967.77008	16967.2241	17518.98019
Q8R5C5	Actr1b	Beta-centractin	280.734	31.38	42281	8	99966.82183	101290.4666	107129.8764	99247.39079	101482.3307	94324.65872	102359.0456	107097.9646	110216.9098	108670.96	101541.5587	100283.6318	112851.8604	119879.0006	84957.32795
Q8R5G7	Arap3	Arf-GAP with Rho-GAP domain, ANK repeat and PH domain-containing protein 3	60.89	2.02	169741	2	8870.23425	7651.204136	9075.189417	7667.99529	7288.628798	8645.01578	7460.668665	8105.753557	10963.90792	8109.089228	6715.305488	8130.24635	6414.671709	6526.674579	7721.494198
Q8R5H1	Usp15	Ubiquitin carboxyl-terminal hydrolase 15	150.196	5.1	112325	5	16830.58645	17890.98536	14591.34691	20915.62583	16809.23686	17417.21482	18945.43491	21419.57244	21677.46318	18944.39274	7480.454736	20436.44777	12987.23889	16137.99413	5697.91057
Q8R5J9	Arl6ip5	PRA1 family protein 3	153.183	23.94	21558	5	64947.26296	60086.62192	63081.78364	68424.98978	63326.78699	98176.09892	78383.56315	94706.11063	84001.25278	77528.74929	72079.43583	51932.86918	70715.48105	96592.85893	65172.73962
Q8R5L3	Vps39	Vam6/Vps39-like protein	58.742	4.06	101693	3	13603.37865	14138.14223	17462.47895	13552.39434	13916.53649	13092.99036	7813.359608	15554.86637	13506.14378	14070.84937	13885.32025	14139.81769	16687.49861	15137.02167	14699.28143
Q8VBT0	Tmx1	Thioredoxin-related transmembrane protein 1	168.028	16.91	31396	4	38522.16793	39918.85788	41903.18794	41661.53123	38315.64931	48834.18456	50577.25992	48006.01971	46269.28823	44702.86695	37216.9455	43722.24167	35414.74341	39105.24089	44111.60291
Q8VBT6	Apobr	Apolipoprotein B receptor	330.318	11.36	102705	10	68354.80437	73788.94025	57884.42355	73065.45817	79784.97178	69928.58372	75879.00859	74711.30124	71360.01216	54006.75444	78138.88962	72413.9778	73654.13571	63952.09472	73739.49877
Q8VBT9	Aspscr1	Tether containing UBX domain for GLUT4	60.7	6.91	59796	2	10794.81008	10816.41825	9985.806809	11256.28588	9561.931154	6454.509576	8011.799586	8915.378725	8614.066779	7440.553176	9432.353499	9905.431487	8630.976247	9282.321113	10632.5077
Q8VBW6	Nae1	NEDD8-activating enzyme E1 regulatory subunit	205.366	17.6	60274	5	19213.36299	20978.96169	18205.82046	17601.08237	17564.71467	15361.76336	19543.95619	18669.0283	18467.664	17110.96891	19370.64381	19311.7116	20259.10385	16853.79412	7061.568034
Q8VC04	Tmem106a	Transmembrane protein 106A	102.21	26.82	29109	5	123115.3099	142602.9075	126563.8017	128252.6844	133602.5245	129954.9534	130766.1299	111378.1016	123014.9807	130505.6501	120859.6071	132162.4329	147178.886	137914.2828	111626.5789
Q8VC30	Tkfc	Triokinase/FMN cyclase	39.395	5.71	59691	2	8325.918741	7500.780579	10025.14458	6276.293473	7128.58627	5197.430288	7059.577075	6249.384676	9004.257467	5605.438951	6542.060012	5801.287239	7212.908345	7954.522757	9645.5984
Q8VCF0	Mavs	Mitochondrial antiviral-signaling protein	233.55	24.85	53399	6	27131.31358	27453.95873	30920.80716	32870.78994	24043.26139	21467.14873	31229.86519	27322.29415	22416.3351	21522.59484	22944.21623	29104.04973	26942.38624	27644.25422	39644.87246
Q8VCH8	Ubxn4	UBX domain-containing protein 4	78.25	10.08	56472	3	10585.5136	9606.891157	4783.688192	10359.72911	10881.10438	12042.41257	13660.99032	5156.563384	12510.8503	10765.59151	4439.989891	8634.365581	10146.03049	10516.06903	5727.750538
Q8VCN5	Cth	Cystathionine gamma-lyase	349.244	29.9	43567	8	91735.9291	99126.88213	84041.22329	91819.34711	78444.5801	137130.2717	163010.5519	146473.0703	166316.0635	129930.9773	113641.8228	123248.0596	117383.3629	117843.4668	120894.9766
Q8VCT3	Rnpep	Aminopeptidase B	1034.075	55.85	72416	27	640507.8737	704821.0836	682337.8047	692513.5666	698034.9332	556694.2133	608786.7992	569254.7144	564944.8464	520171.5342	568713.8899	661660.7931	633850.7523	640361.7671	636777.8227
Q8VCW4	Unc93b1	Protein unc-93 homolog B1	192.292	13.88	66981	6	52044.65908	59820.69714	49175.69761	51608.76244	59921.59614	64656.72144	53229.99308	63070.73404	49887.16	49560.84568	49657.52083	56190.033	65028.28111	48108.50835	60275.69703
Q8VCW8	Acsf2	Acyl-CoA synthetase family member 2, mitochondrial	80.641	5.04	67951	2	15390.35257	16060.10939	10743.15048	13167.55879	13096.65834	17409.67957	15026.92141	14964.69089	16650.37094	16216.54001	14364.60632	13993.26945	12763.79709	11943.46567	14745.17419
Q8VDD5	Myh9	Myosin-9	6108.517	65.77	226372	143	5815748.475	5900396.63	5343489.037	5595955.647	5567632.364	5027692.829	5122633.499	5154454.032	5065190.692	5154755.489	5727510.874	5682539.518	5787184.843	5793794.283	5678934.586
Q8VDF2	Uhrf1	E3 ubiquitin-protein ligase UHRF1	38.961	4.73	88304	3	9955.045203	11782.49984	10171.87072	10136.68672	10805.65775	9029.645236	9172.036713	10234.36847	11103.57756	8520.85197	9768.207083	10315.88157	9926.534181	9209.783896	10227.68416
Q8VDJ3	Hdlbp	Vigilin	1039.386	32.1	141743	27	364317.3519	339093.2223	356721.5783	322299.4807	345478.3606	295170.3888	357097.1872	372378.686	335606.4648	333214.9852	346819.2221	325438.4205	342308.6517	327902.4435	354053.3247
Q8VDK1	Nit1	Deaminated glutathione amidase	137.33	25.08	35705	4	15294.38208	14517.18942	10768.29589	12183.86048	9829.685056	7034.771161	5813.054615	7210.324713	9290.101282	8233.897648	11647.2274	12134.60561	7820.635788	10255.77501	9148.214844
Q8VDL4	Adpgk	ADP-dependent glucokinase	153.351	11.69	53902	4	28832.55263	31222.89845	28535.15878	23800.40336	26078.77255	41807.12131	37612.19255	34666.11621	37476.59123	33635.89622	30914.12569	31680.00777	32573.11802	31242.66331	31902.04357
Q8VDM4	Psmd2	26S proteasome non-ATPase regulatory subunit 2	1167.44	45.04	100203	29	410161.3038	426491.1229	359918.6984	442954.5568	390059.6756	405904.9196	419166.6934	482382.9824	448156.592	351141.9854	401920.726	458942.1462	452695.1243	372442.2948	434551.7313
Q8VDM6	Hnrnpul1	Heterogeneous nuclear ribonucleoprotein U-like protein 1	336.085	17.23	96002	11	106264.5175	110186.726	106383.966	115024.1286	98302.17377	89707.55268	98332.46749	73446.76074	92041.0853	65215.49525	70871.07262	76901.00781	96842.89699	103414.407	93789.76445
Q8VDP3	Mical1	[F-actin]-monooxygenase MICAL1	108.756	8.11	116785	5	13321.00763	9291.755842	10427.23697	13975.56397	8994.72378	12539.29205	7960.141289	13397.28111	7418.727578	9457.712331	8526.162563	16622.60473	10233.26475	12743.23441	9825.405347
Q8VDP4	Ccar2	Cell cycle and apoptosis regulator protein 2	138.216	9.76	103002	6	43281.45254	37315.92355	38589.49524	34467.71065	34147.60027	36402.86688	37762.30369	31161.27197	36472.19897	31985.13878	32368.91657	37620.68922	36681.76066	36222.24881	39235.28374
Q8VDP6	Cdipt	CDP-diacylglycerol--inositol 3-phosphatidyltransferase	87.182	17.37	23599	3	13935.77401	14678.13275	14693.53335	13686.07006	12555.14333	15688.65077	12890.3236	10471.82606	15379.12614	17096.54315	15093.92486	16693.3819	14084.44571	16663.88955	8239.454162
Q8VDQ8	Sirt2	NAD-dependent protein deacetylase sirtuin-2	92.204	9.51	43256	2	10266.69169	9879.20713	9286.741668	10023.47174	10552.41456	9733.59057	9755.694318	7429.684896	8335.895129	7591.684017	11067.31078	10004.75117	10509.87614	8605.916801	9925.702624
Q8VDV3	Rab3il1	Guanine nucleotide exchange factor for Rab-3A	81.456	10.97	42713	2	8257.278693	5722.751265	4572.938626	5716.477843	5561.416612	4076.421349	3405.258437	5272.098728	1948.812989	3521.888189	5892.214487	2671.06727	4659.368415	4341.235299	5598.047135
Q8VE22	Mrps23	28S ribosomal protein S23, mitochondrial	43.686	11.3	20348	2	5616.961837	6094.049806	540.0150523	5887.725872	4765.561115	6405.279197	6508.442325	3846.74245	5542.990925	5578.64453	4655.506233	5588.944943	6094.146044	5537.47999	5787.731497
Q8VE37	Rcc1	Regulator of chromosome condensation	276.016	30.64	44931	7	113393.5996	109650.5508	103199.6748	112417.9669	108996.7074	87044.45029	98383.14562	87414.27215	88349.76164	96902.62149	111328.5331	107600.9109	107665.0123	113347.2421	106021.0651
Q8VE88	Fam114a2	Protein FAM114A2	174.493	18.11	54045	5	11246.9922	26107.23618	21800.41116	27312.53941	8386.159215	7397.114813	23900.29565	8355.544031	8807.645658	22882.51099	12958.18147	5802.086533	9524.319079	8893.019545	24337.46306
Q8VE99	Ccdc115	Coiled-coil domain-containing protein 115	70.489	22.78	19743	2	6546.624106	6735.040161	7775.720392	6797.808878	5620.833673	4345.832491	5963.874618	5736.383951	5004.015622	4727.860482	5770.658696	5388.043017	5891.629328	4471.940258	4970.112643
Q8VEB4	Pla2g15	Group XV phospholipase A2	146.52	21.84	47307	6	76389.42907	62606.85367	57731.5754	55634.46423	62118.23162	39556.14226	49915.68733	44086.05317	43475.66355	45364.49063	57590.20956	59836.97977	61341.04137	63890.88856	56652.67487
Q8VEE4	Rpa1	Replication protein A 70 kDa DNA-binding subunit	195.433	14.13	69037	7	48836.88599	56645.82865	58123.07493	44528.15685	50385.13416	46081.86596	54980.32754	50616.43312	49617.64005	50808.1946	48062.06443	47051.46328	51618.43251	53511.53929	41693.75881
Q8VEH3	Arl8a	ADP-ribosylation factor-like protein 8A	273.771	44.09	21390	7	204705.3541	205716.7115	164133.3868	182416.1953	173270.365	124023.6748	142311.1628	146403.7912	154140.7825	151549.3743	178863.7366	194669.7214	192469.0881	190443.7482	182386.6705

Q8VEM8	Slc25a3	Phosphate carrier protein, mitochondrial	445.343	35.29	39632	11	222565.2546	220412.2246	217267.502	220429.0075	213086.3959	208858.6354	203141.0305	197093.1278	231865.6099	219107.6307	193457.5945	219377.3082	214615.1619	235261.3321	204946.3949
Q8VH51	Rbm39	RNA-binding protein 39	289.043	17.55	59407	6	38010.18549	37581.69331	30996.69868	38713.15439	34087.79265	31008.25961	27064.29577	34947.64132	38127.47495	38232.41633	34310.38114	37763.4459	29922.88242	34855.43421	37211.87106
Q8VHR5	Gatad2b	Transcriptional repressor p66-beta	34.843	5.89	65411	2	12980.54224	11292.56496	9430.550069	9743.309429	12195.64872	8482.914319	5599.32488	8110.248433	8393.513249	9999.898884	10956.42306	8985.426741	9781.392554	4793.804176	12680.81066
Q8VI93	Oas3	2'-5'-oligoadenylate synthase 3	642.936	28.3	126333	19	110048.8292	152840.316	123344.9514	112773.2371	126124.6086	147212.125	123344.0918	134738.4716	150053.891	132551.138	149769.2949	130305.9235	141442.9519	124916.6197	99429.98563
Q8VIJ6	Sfpq	Splicing factor, proline- and glutamine-rich	665.644	31.9	75442	18	484663.5046	578361.2188	496568.9848	492127.3366	505937.5287	472512.933	449686.4411	431817.6094	396878.4439	459627.3952	484769.9582	454272.7458	478907.6509	465341.2616	459002.9777
Q8WTY4	Ciapin1	Anamorsin	142.093	29.77	33429	5	30967.0666	31553.38925	30061.94274	30158.02334	24414.46045	12561.48436	11243.36828	10897.89428	17152.47645	9948.725191	29212.3801	15726.77694	24416.35555	11308.31289	22526.14106
Q91V04	Tram1	Translocating chain-associated membrane protein 1	171.159	19.25	43039	6	43309.14649	51637.36173	36312.97316	48399.25676	52182.85516	55001.72393	53961.62165	55101.30371	58026.36931	57301.83234	55723.85431	51338.23496	52855.58572	54119.2084	22906.89414
Q91V61	Sfxn3	Sideroflexin-3	244.941	22.12	35406	4	15639.80385	13694.49501	11902.00406	10524.11885	10202.54969	10297.53487	7094.999476	10638.52229	7509.680681	10168.88877	13144.96304	12715.67673	12761.37608	14398.21559	10612.1139
Q91V64	Isoc1	Isochorismatase domain-containing protein 1	205.121	30.98	32033	6	31520.4131	44445.45146	49376.3426	38872.74993	32465.93194	34910.53822	42960.31977	39947.30703	39030.26708	30916.29368	42748.4012	42586.98561	43859.28473	42194.69324	41830.97735
Q91V76		Ester hydrolase C11orf54 homolog	97.997	18.1	34996	3	16210.41332	17359.69918	13076.32671	12611.28549	13223.38223	7740.826511	10611.13023	5841.508067	8734.590286	8794.396994	9213.538124	11258.18278	12177.10815	10170.49183	13374.65295
Q91V92	Acly	ATP-citrate synthase	784.041	24.47	119728	21	256067.4989	284528.3663	261292.1242	280850.5494	243809.9984	238421.6971	277335.38	235185.8143	248353.9713	236476.1421	244338.5093	282797.4225	266899.5561	225296.1469	257102.4106
Q91VD9	Ndufs1	NADH-ubiquinone oxidoreductase 75 kDa subunit, mitochondrial	429.426	23.11	79777	13	149972.3698	163988.2696	155511.5815	157896.2959	150453.3376	168709.3638	147124.6474	154516.5219	166670.7472	146222.1254	150564.9733	151968.8918	161768.0926	151437.4159	139426.5709
Q91VH2	Snx9	Sorting nexin-9	142.806	14.45	66546	5	21668.98579	18019.5502	6613.086051	24295.64754	21521.22591	9501.333632	25355.43057	10708.9487	20537.13734	17652.08985	19088.9346	20729.50122	15642.02339	24732.9073	8429.313977
Q91VI7	Rnh1	Ribonuclease inhibitor	1644.278	88.6	49816	30	835420.8204	852669.3356	745567.6483	820085.0122	765490.4258	743641.0802	764718.7859	770095.5762	760593.3437	611310.7572	690917.3297	800917.3005	710815.3382	782729.1538	816918.2823
Q91VJ1	Aim2	Interferon-inducible protein AIM2	135.597	14.12	40155	4	27569.1356	22174.47373	21525.49371	18432.52545	30699.30917	16970.28683	15875.89136	21236.79496	24777.33525	23588.19785	14700.16118	22354.61828	20607.18964	25422.86312	21235.9001
Q91VJ4	Stk38	Serine/threonine-protein kinase 38	101.51	12.26	54174	3	7285.611193	6603.847827	4407.402441	7359.873721	5747.970353	5007.226157	4560.683918	3980.994941	2641.545645	5288.383138	1042.33863	6216.381384	6071.277091	5952.72616	6789.103943
Q91VM9	Ppa2	Inorganic pyrophosphatase 2, mitochondrial	59.856	10	38115	2	7903.557585	8560.614874	8099.727464	7533.917194	5935.551086	6172.131186	7054.076633	6800.915744	6526.86711	8060.842645	8063.905259	7642.241482	7768.18548	7873.892095	6741.32626
Q91VN4	Chchd6	MICOS complex subunit Mic25	76.019	10.26	29852	2	17475.96508	10594.55116	9573.432395	5197.145207	10755.19055	14350.74568	14610.04156	13119.54517	15437.13395	10371.72358	10407.0235	10152.64779	16237.869	13828.17798	11348.26683
Q91VR2	Atp5f1c	ATP synthase subunit gamma, mitochondrial	288.365	41.95	32886	10	135202.7498	139961.7036	122152.636	145634.0168	142498.3411	147636.4109	134682.4608	101604.861	142382.7287	138717.9317	107849.4619	119597.9428	124380.3316	140920.2641	119084.4834
Q91VR5	Ddx1	ATP-dependent RNA helicase DDX1	342.974	25.81	82500	13	131437.7124	121487.0623	116037.6925	121269.5401	118043.8305	117343.5312	114228.473	118412.5842	118964.2186	121337.7784	103696.9776	127844.1151	115667.7867	127519.5623	129395.688
Q91VU0	Fam3c	Protein FAM3C	136.48	20.26	24753	4	47771.46862	46440.80996	36097.63534	42888.86793	56353.54307	56269.47085	45414.39068	41723.30298	48601.07378	50329.05416	53746.39006	42217.98787	49599.9284	50177.44671	49524.96205
Q91VW3	Sh3bgrl3	SH3 domain-binding glutamic acid-rich-like protein 3	104.967	55.91	10477	4	25272.3366	21576.28098	15851.9673	19087.62655	17666.94268	13072.61821	14785.99497	17339.98566	16698.08522	11509.11356	66123.94984	21822.99925	18879.55012	17901.83197	17710.52357
Q91W50	Csde1	Cold shock domain-containing protein E1	192.314	13.41	88791	8	59906.59243	40774.07257	69259.07891	59146.53837	61993.79325	79618.66212	57163.30932	84717.61751	91035.4273	88382.30399	46132.27119	62507.93609	65175.20477	65960.73946	59148.9292
Q91W86	Vps11	Vacuolar protein sorting-associated protein 11 homolog	39.991	4.68	107719	2	8616.449748	10164.22134	6209.744299	8503.386535	7375.706522	7924.635965	7884.176563	9521.639824	8474.390378	8202.6044	8617.44619	9009.728232	8250.230131	6430.214005	8274.12079
Q91W89	Man2c1	Alpha-mannosidase 2C1	128.331	5.29	115688	4	17037.39035	17082.85427	16688.82815	16341.37052	14913.63879	15814.62177	12308.27364	15154.43063	14789.34811	14158.62108	15044.64153	16752.21947	16922.28873	15606.07503	16387.8299
Q91W90	Txndc5	Thioredoxin domain-containing protein 5	155.332	17.75	46415	6	50856.94901	50711.36283	43424.29924	54773.40766	41235.82435	44580.75889	50653.99025	46696.17843	49628.85646	57372.89554	43356.04679	44750.6142	47928.63685	46661.39525	55985.83701
Q91WC0	Setd3	Histone-lysine N-methyltransferase setd3	73.032	6.57	67176	2	8477.908513	8182.110297	6777.470347	8412.442432	7137.906522	5647.226253	6966.563799	7158.59844	6838.712138	5757.566275	8112.595641	6843.378554	7014.608111	7011.417185	5062.029638
Q91WD5	Ndufs2	NADH dehydrogenase [ubiquinone] iron-sulfur protein 2, mitochondrial	32.247	6.91	52626	2	5270.618724	0.007562416	0.009509917	0.009171104	7162.417935	7449.042761	6624.644554	8218.605432	1199.168607	7608.007107	6941.474286	1463.42213	8201.057483	4703.532492	730.1357028
Q91WK2	Eif3h	Eukaryotic translation initiation factor 3 subunit H	308.963	37.22	39832	10	128389.0901	138556.9697	132473.1693	134047.6021	138261.609	124158.9386	134000.1458	136266.9432	137677.4216	119250.5185	140507.9128	135957.0657	133360.4877	127041.4876	123097.166
Q91WM3	Rrp9	U3 small nucleolar RNA-interacting protein 2	57.29	11.58	52107	2	1215.872434	1661.366763	3200.82453	1927.03945	1331.166987	1312.870534	1781.963904	3350.104856	1862.420941	2886.144439	1797.89582	1611.748287	1319.390924	1524.712869	1297.239527
Q91WQ3	Yars	Tyrosine--tRNA ligase, cytoplasmic	323.828	27.46	59105	12	128930.2678	132015.1323	146873.4857	161121.7547	138053.673	110989.2826	148425.8679	157454.4451	108454.7412	102905.4636	148358.0815	150612.7543	143670.124	130738.661	142568.3051
Q91WU5	As3mt	Arsenite methyltransferase	106.388	12.23	41794	3	16237.18181	15607.43642	11186.51591	14745.52856	14755.53771	12356.37504	13464.26215	16181.4085	15026.96791	13226.11808	15261.8436	15156.31967	15935.30776	13394.2632	16465.54857
Q91X20	Ash2l	Set1/Ash2 histone methyltransferase complex subunit ASH2	44.107	3.69	68250	2	7110.010851	7680.936227	10831.95172	11817.91739	9582.629898	7950.817564	7875.875301	6719.472662	6667.115175	10902.56886	12975.3304	7815.517441	6578.497057	9211.58826	7752.304108
Q91X91	Qprt	Nicotinate-nucleotide pyrophosphorylase [carboxylating]	118.312	18.39	31530	3	70924.39481	62467.87538	69190.15103	69866.87986	72658.33005	66575.3861	61769.71	74113.88923	76565.69836	50281.4578	64776.29906	69454.08451	71135.30918	74648.54343	44023.30626
Q91XD6	Vps36	Vacuolar protein-sorting-associated protein 36	85.835	17.1	43736	4	6030.251855	25205.05889	21469.98433	22380.14333	24191.26779	9052.40642	22935.97861	22415.78334	15045.97357	20417.48886	20585.91487	22264.18707	20094.57084	20896.5838	24017.05423
Q91XD7	Creld1	Cysteine-rich with EGF-like domain protein 1	71.479	6.43	45718	2	9105.784051	1956.588612	1287.385629	1906.829639	1639.858216	997.6670936	1658.2388	2203.05726	8828.306739	1463.891472	3119.337874	7551.22894	7319.247586	7063.807103	8715.452471
Q91XU1	Qki	Protein quaking	203.341	23.75	37643	5	64714.09982	64286.28092	71090.73181	62294.99201	56941.54734	48053.27738	51447.84587	37111.99014	42920.42891	37215.69312	59678.57361	57610.55336	51186.73726	49218.60225	48850.41086
Q91XV3	Basp1	Brain acid soluble protein 1	801.696	84.07	22087	14	1144827.639	747578.5585	935338.4316	820983.2668	915820.6104	914920.9642	798046.4778	758037.1615	754324.9176	740907.278	1013588.527	785056.4754	802164.6964	776361.1034	689080.9356
Q91YE6	Ipo9	Importin-9	480.2	15.75	116052	9	50550.24655	40744.75686	53106.29866	66193.85286	53768.40395	29574.00291	57953.80306	62876.06117	55007.74593	42416.8365	53430.24618	58584.45613	56109.48187	51775.98419	50549.2291
Q91YH5	Atl3	Atlastin-3	275.605	21.44	60575	7	52953.18378	55283.47459	52825.26158	46761.23804	39919.66403	60710.05282	58183.35502	50079.85724	63307.45584	60005.12432	55921.32957	54904.35111	57044.74717	65178.02877	56766.72752
Q91YI0	Asl	Argininosuccinate lyase	410.107	29.53	51739	11	84232.96012	95804.48604	86366.66439	96649.99668	78709.53394	71896.85091	97095.74928	86344.28649	81025.45292	84529.24678	95541.46338	96074.86943	95880.84113	95003.61704	93727.19789
Q91YP3	Dera	Deoxyribose-phosphate aldolase	188.828	20.13	34975	5	27632.2028	31869.06339	32716.06561	35382.85611	28862.91635	31284.54852	33327.76598	32072.40115	29383.89994	32161.29159	28188.70236	29435.29496	31133.30641	32454.72964	32752.3126
Q91YQ5	Rpn1	Dolichyl-diphosphooligosaccharide--protein glycosyltransferase subunit 1	874.429	46.71	68528	22	671092.0064	727922.3881	696097.2859	713179.5114	702450.4228	876697.8089	856536.7047	836062.0606	865630.5526	847938.0701	721615.1335	757072.3887	760671.9525	758274.53	776139.1022
Q91YR1	Twf1	Twinfilin-1	234.228	29.71	40079	7	48790.0329	48709.69349	39869.93906	46716.41886	39172.07082	47872.24234	57119.33023	48167.7958	46741.87553	49083.54675	52807.02674	58417.55986	48787.32269	53162.8415	48068.58792
Q91YR9	Ptgr1	Prostaglandin reductase 1	329.123	36.47	35560	9	115224.2471	111227.7379	110233.5331	115409.5259	103698.7051	82115.43648	92258.58649	87925.26935	77504.9944	83848.26555	90731.84405	84423.56744	89706.02478	65716.94557	85353.12177
Q91YT0	Ndufv1	NADH dehydrogenase [ubiquinone] flavoprotein 1, mitochondrial	157.716	21.98	50834	6	44219.39872	43737.598	38269.58162	32720.89742	38600.42882	40806.56865	27990.7987	21967.97081	26144.44058	35672.06661	27416.68449	24739.16005	41557.96153	41677.16195	27790.8075
Q91YT7	Ythdf2	YTH domain-containing family protein 2	64.795	4.15	62280	2	15480.29418	12578.51878	10011.66546	9233.409823	13190.98006	15555.11361	11917.11257	14829.73456	11004.71956	8019.303643	13161.89069	9634.734502	12616.48097	10746.07322	10557.27103
Q91YT8	Tmem63a	CSC1-like protein 1	29.435	2.74	91860	2	0.008226335	7813.967174	7008.181516	0.009171104	7488.53653	8974.284078	7471.661894	5884.064968	7194.475746	6879.888594	7640.298898	0.009057159	14798.59089	7613.666017	13198.71235

Q91YX0	Themis2	Protein THEMIS2	64.675	7.24	74378	2	3441.614113	3178.438173	897.1429323	3289.55079	2450.319321	1208.894684	2228.880866	2055.220716	2253.884131	1565.306047	2769.620307	2809.983446	2528.542304	2613.517703	1953.705208
Q91Z53	Grhpr	Glyoxylate reductase/hydroxypyruvate reductase	105.315	12.5	35329	2	3354.105689	3851.391305	2671.920675	4101.696405	2850.472403	2019.650402	2226.189825	2946.718054	3130.759084	2457.792403	3169.536718	3689.252756	3674.737321	3323.93174	2838.172681
Q91Z67	Srgap2	SLIT-ROBO Rho GTPase-activating protein 2	226.916	8.87	120798	8	62568.92247	60554.00946	61683.05547	23767.85282	27395.44329	64612.57133	59082.38568	53316.54547	34272.87755	57096.52164	57109.89054	30937.81884	26023.47195	61094.25178	50487.9576
Q91ZA3	Pcca	Propionyl-CoA carboxylase alpha chain, mitochondrial	104.067	7.18	79922	3	6652.941758	9948.599856	8307.75572	6799.652269	8647.746249	6524.572798	7488.063755	7669.463304	9013.413653	7339.798483	8040.991547	7944.794806	7645.302671	8426.826731	6072.065361
Q91ZJ5	Ugp2	UTP--glucose-1-phosphate uridylyltransferase	356.313	26.77	56979	10	133143.5417	132088.1247	114298.8801	153684.3015	128363.6314	133348.8915	128246.7695	144231.1037	137761.6065	163686.7819	111994.7445	136607.0256	133582.1217	117135.7071	159990.8687
Q91ZR2	Snx18	Sorting nexin-18	249.209	13.19	67904	7	91016.68415	83523.01706	75975.99067	79027.19828	83603.06295	86134.8259	89316.69837	88545.87853	74428.90243	86213.02497	77356.64072	86639.63698	77363.88814	71229.26815	84168.85362
Q91ZV7	Plxdc1	Plexin domain-containing protein 1	90.334	7.6	55635	3	30367.60326	31240.35062	26490.84986	27345.47742	26554.18343	23509.47802	24894.76766	14992.07826	25485.17401	31173.58308	30579.48712	25280.61968	29322.92821	29224.8341	29719.47417
Q91ZW3	Smarca5	SWI/SNF-related matrix-associated actin-dependent regulator of chromatin subfamily A member 5	195.545	7.33	121627	7	59721.38061	64475.37913	60087.49103	54631.02169	60601.32665	62876.65014	59153.47584	61667.09388	65933.43561	55233.00751	60595.99031	62104.13169	68465.8355	62622.6043	62449.70614
Q91ZX7	Lrp1	Prolow-density lipoprotein receptor-related protein 1	2764.038	22.4	504742	73	1259187.125	1216806.73	1090362.792	1117435.157	1215509.377	879684.749	910759.7436	835685.9624	837641.8519	958483.3668	996298.8925	987516.015	1069052.116	1108972.038	1063142.038
Q920A5	Scpep1	Retinoid-inducible serine carboxypeptidase	437.629	27.43	50965	14	339462.0391	338661.0454	261158.9474	327055.1748	308566.6054	200291.6838	268283.8991	197175.1961	267690.8462	234539.307	341791.7862	336347.769	350227.2721	287752.4898	306609.5994
Q920B9	Supt16h	FACT complex subunit SPT16	441.24	19.29	119825	15	115849.0866	134425.5918	140623.3112	144611.1727	139180.9378	137604.8362	121563.6395	121170.9707	122616.374	126172.6999	124488.6395	136960.4722	119428.0198	126487.261	138555.5811
Q920E5	Fdps	Farnesyl pyrophosphate synthase	786.464	56.94	40582	18	522514.5187	539572.8626	611624.0746	782305.4945	769250.0029	454848.3272	565369.7125	557862.845	718635.0394	479121.5953	631200.3988	740808.9184	651415.5396	672793.9859	461525.4905
Q920L1	Fads1	Fatty acid desaturase 1	86.641	8.72	52323	2	13093.90628	14891.15654	12648.7418	12893.21668	14083.83335	11860.25252	13955.69592	15798.1849	13376.0782	13393.7218	11064.8355	12310.54093	12611.43536	13673.11293	12648.82935
Q920Q4	Vps16	Vacuolar protein sorting-associated protein 16 homolog	284.336	10.61	94928	5	22211.42623	18509.31115	22158.0435	22995.01309	19577.46578	20501.43342	16880.29772	23056.37152	21398.87163	23638.66637	24189.04581	20790.80234	20744.78983	19537.76099	19851.84816
Q921E2	Rab31	Ras-related protein Rab-31	169.472	30.41	21331	5	42328.79766	60119.58271	54820.87021	56664.63138	63695.80386	55142.76362	47890.00968	54523.56867	57570.32416	53686.53048	52825.99216	48527.79839	59556.60256	57008.99013	59927.8564
Q921F2	Tardbp	TAR DNA-binding protein 43	665.188	42.75	44548	13	331267.0626	345280.6396	313251.6124	319530.0642	330180.9007	285997.4911	276894.8111	341686.0822	315480.3031	291188.4368	315286.1034	323475.2268	319158.813	308063.7049	313361.6487
Q921F4	Hnrnpll	Heterogeneous nuclear ribonucleoprotein L-like	394.855	33.84	64125	13	137540.4522	148710.3144	131358.7496	145173.2238	132750.0086	107904.9259	115885.4159	108062.2889	113145.7174	112099.8558	135891.8903	128678.7958	116945.5697	123098.2223	136130.9535
Q921H8	Acaa1a	3-ketoacyl-CoA thiolase A, peroxisomal	269.331	29.25	43953	8	92779.22586	95624.64423	75389.48078	74903.01718	83485.5059	95470.87473	79111.339	81742.14559	102598.6123	97233.2152	85220.64119	78617.87389	90092.53276	70473.85319	71233.7025
Q921M3	Sf3b3	Splicing factor 3B subunit 3	700.526	23.66	135550	19	214959.3495	235506.5946	223103.5671	248174.971	239567.5628	201337.4152	232318.746	208108.7879	214397.3341	219833.4476	220763.1559	244360.3051	213453.8621	206772.7663	208762.3642
Q921M7	Fam49b	Protein FAM49B	581.935	58.64	36776	15	245539.7969	285304.1794	280562.2222	282940.0203	250766.1155	224577.7289	236261.7619	228537.4558	220253.4509	222238.3674	242502.9758	261257.0157	253071.4933	266091.6092	250333.2459
Q921S7	Mrpl37	39S ribosomal protein L37, mitochondrial	115.989	11.11	48341	3	9838.010159	11265.07463	9987.667186	9891.331042	9699.484345	10579.37252	10391.83214	8709.726045	10363.28505	9346.898366	9528.722464	11233.56542	11098.92722	9409.7364	10340.59261
Q921T2	Tor1aip1	Torsin-1A-interacting protein 1	343.201	25.38	66781	9	109716.5264	98128.56763	94308.11116	87521.00675	99173.27958	113154.9857	114404.7351	91905.50964	102848.9559	94577.35642	91834.80719	52893.8021	113183.8061	93400.36085	93186.95396
Q921W4	Cryzl1	Quinone oxidoreductase-like protein 1	37.166	11.78	38725	2	10780.9086	11332.02281	9922.597954	11383.73671	8903.339544	8579.182331	12977.81871	10123.59782	12561.13381	11181.27792	11077.63736	12239.88263	10194.07785	9595.562362	10049.4047
Q921Z5	Tnfaip8	Tumor necrosis factor alpha-induced protein 8	85.739	21.72	22960	2	5902.843443	2671.832663	4615.965962	5058.864453	5071.25531	3372.752053	4111.345729	5115.307843	5168.894584	4146.272416	4650.386698	179.6792802	4208.413308	4093.386435	5384.821369
Q922B2	Dars	Aspartate--tRNA ligase, cytoplasmic	547.159	46.51	57147	22	375620.8302	402113.0708	432394.9206	378575.0601	417896.741	522507.8879	455131.723	545266.6949	495647.7826	472366.6088	386408.9002	405348.108	399895.1115	388752.2489	387283.3664
Q922D4	Ppp6r3	Serine/threonine-protein phosphatase 6 regulatory subunit 3	123.538	4.15	94653	4	9161.455775	10063.08065	7162.64635	6789.201201	10151.35509	10273.37881	11156.08074	11647.03047	12218.65135	11685.27716	7553.189834	10064.17066	9770.706505	10294.87106	10438.3464
Q922D8	Mthfd1	C-1-tetrahydrofolate synthase, cytoplasmic	571.914	23.85	101200	18	146826.1091	168510.2766	225030.2384	218614.6881	158885.6875	228001.7789	222635.5912	173328.0121	186996.8527	216244.5497	148592.4898	177693.978	147558.4964	203795.1541	207995.1339
Q922F4	Tubb6	Tubulin beta-6 chain	1199.02	64.65	50090	27	3021368.976	2979401.827	2969736.95	3039797.38	2903157.996	2748006.518	2812769.623	2500315.254	2818368.999	2815131.366	3212375.058	3027462.935	2720448.158	3028860.441	2715511.792
Q922H2	Pdk3	[Pyruvate dehydrogenase (acetyl-transferring)] kinase isozyme 3, mitochondrial	102.409	10.6	47923	3	8995.024672	8434.756426	6389.292729	8298.387744	7615.0977	7324.65296	7155.713836	9504.744429	9300.341952	5704.399215	7777.483333	6448.681674	6387.359921	7320.342183	6370.920645
Q922K7	Nop2	Probable 28S rRNA (cytosine-C(5))-methyltransferase	44.331	3.78	86752	2	6977.573022	11399.56842	4197.097463	11107.86074	11781.39487	12345.21117	9368.413015	13614.64165	6067.303462	9959.860867	3071.551428	7018.536047	10316.44064	9214.929279	10798.63315
Q922L6	Nelfcd	Negative elongation factor D	77.402	5.25	66276	2	8115.508086	6471.312711	4697.716161	6674.371555	5298.791676	4724.494741	6525.176051	5664.161898	5326.875382	6228.718802	6645.564141	6813.130449	6168.640542	6797.618236	6295.051935
Q922Q1	Marc2	Mitochondrial amidoxime reducing component 2	233.604	36.39	38194	9	108705.6661	110152.9296	88426.28427	79917.59322	113678.4981	81361.67147	78250.2552	74139.75952	83740.58392	80353.99423	63827.01049	77424.02253	112939.3699	70845.90341	95155.3606
Q922Q8	Lrrc59	Leucine-rich repeat-containing protein 59	337.106	39.74	34877	9	232561.8973	259211.274	239914.2672	272390.7199	246184.9781	287053.3423	301788.5693	307002.8163	307845.2942	259009.3961	211778.1523	248025.2484	246578.4577	263742.1816	263785.0658
Q922R8	Pdia6	Protein disulfide-isomerase A6	791.765	48.64	48100	16	689651.8035	712232.7955	681308.7556	671463.8294	636505.0034	774412.879	769355.1934	812105.702	745237.679	743566.6266	677425.2843	651225.9311	662772.5337	674251.9265	664527.204
Q922U1	Prpf3	U4/U6 small nuclear ribonucleoprotein Prp3	89.173	12.3	77455	5	34470.51885	33609.56849	35410.42493	40999.71687	30238.38834	24217.2391	31435.58151	30905.04781	29626.18695	27080.90903	27919.31222	30571.95941	32411.23169	31063.28837	36099.75545
Q922W5	Pycr1	Pyrroline-5-carboxylate reductase 1, mitochondrial	64.77	10.68	32373	3	64747.6098	55850.22471	59230.79487	53983.63348	59961.14414	73827.95437	66409.11816	57939.68948	72020.59884	61968.44661	61739.13325	57022.02367	60807.85013	61652.02451	62825.753
Q922Y1	Ubxn1	UBX domain-containing protein 1	53.654	24.24	33573	4	36520.6265	35314.45839	26972.18244	35879.16414	33275.67408	23838.25378	29178.5799	35410.99789	23762.83674	25870.57025	42183.22462	40492.63976	36830.11926	22567.26605	25747.64786
Q923D2	Blvrb	Flavin reductase (NADPH)	485.078	63.11	22197	9	89878.23338	95459.91969	73116.74365	89279.60255	74082.96737	66359.68817	49200.18279	59764.78066	77377.81071	78656.52955	100378.6417	92243.78156	92406.55705	88767.1327	71200.8289
Q923G2	Polr2h	DNA-directed RNA polymerases I, II, and III subunit RPABC3	69.717	12.67	17143	2	23521.55445	21762.88359	0.009509917	18416.94833	663.5936945	8147.423859	14685.8226	21476.91442	11838.75196	8762.664179	17691.67965	19919.97004	18346.30491	5119.99853	8581.536315
Q923V8	Selenof	Selenoprotein F	105.064	15.43	17822	2	7922.454505	8203.360685	8537.043396	2170.02243	6951.686061	7792.550975	8485.344243	4643.42199	9283.270422	7364.254347	10317.82691	6866.052564	7162.091667	7256.175623	7708.263324
Q924C1	Xpo5	Exportin-5	97.908	5.15	136973	4	20559.81254	20420.32415	19712.01902	19334.21346	19488.88677	17554.38147	22290.8758	20454.22917	20497.03409	19238.87044	20621.06768	17755.93162	17314.00298	18084.27313	17481.71118
Q924K8	Mta3	Metastasis-associated protein MTA3	69.652	4.91	67077	2	7862.095827	5132.237653	7481.122787	5442.341541	5942.14877	5886.784933	4302.231529	8237.28085	3781.284833	6501.688127	7714.724268	7357.260813	7816.216893	7306.577346	8235.765854
Q924N4	Slc12a6	Solute carrier family 12 member 6	36.913	2.52	127527	3	16568.42343	18847.64796	14819.44822	17475.63084	11725.91649	16150.40572	13837.53491	12884.5906	13396.81027	17298.48051	12774.44547	16071.57734	14882.07158	14843.32313	16718.4826
Q924T2	Mrps2	28S ribosomal protein S2, mitochondrial	72.702	13.06	32313	2	9386.225992	8413.056074	8604.725479	4205.308141	6678.514384	6053.393805	8111.139171	7627.018744	9916.151405	6764.932517	7934.949295	7870.402106	4032.134011	7234.865762	7425.510045
Q924Z4	Cers2	Ceramide synthase 2	118.544	11.84	45024	3	54530.28743	58329.9446	55874.5548	49288.72084	27177.54904	87955.77584	79036.14296	81425.28664	87807.68278	77139.287	59668.38282	64598.07942	61490.96247	61995.68208	65716.38176
Q93092	Taldo1	Transaldolase	956.355	77.74	37387	28	2809041.864	2894525.387	2862094.422	2908448.687	2985974.232	2380057.701	2491772.125	2451000.903	2462836.703	2390288.586	2844678.343	2811830.952	2833517.865	2848763.924	2688607.034

Q99020	Hnrnpab	Heterogeneous nuclear ribonucleoprotein A/B	444.007	33.33	30831	11	434787.036	411036.4189	395974.2571	406162.9871	399551.0638	353045.537	434021.809	420258.687	436492.2087	394999.793	422400.573	420001.6862	424233.0356	435942.4852	410123.8995
Q99104	Myo5a	Unconventional myosin-Va	75.721	2.48	215538	4	13427.75462	13259.27987	12952.27103	6973.751319	13446.4354	16855.91677	8312.275007	11075.88186	8165.924167	6740.682251	10871.46492	11099.9927	12111.357	10773.52261	10796.94373
Q99J09	Wdr77	Methylosome protein 50	103.063	11.4	36943	2	7211.539213	7177.484018	7981.696313	7018.461642	6961.564258	7210.205182	7186.554952	8617.253305	6881.968428	7944.32764	6241.804277	7754.937453	7531.064512	6719.436719	6691.474526
Q99J36	Thumpd1	THUMP domain-containing protein 1	159.824	20	38885	4	18689.71973	15953.15036	19190.30138	15449.93237	14687.23072	12510.01798	17526.17694	14668.14941	14765.27649	18323.54382	17789.08358	17278.03739	17053.54819	15255.168	15419.57256
Q99J47	Dhrs7b	Dehydrogenase/reductase SDR family member 7B	54.128	12.07	34987	2	4382.670638	6425.153162	3756.244127	6940.33356	6599.858537	8566.599879	7415.020987	5634.305739	7836.46005	6899.887589	5159.810109	7555.228264	7223.268747	4125.20905	7591.675587
Q99J56	Derl1	Derlin-1	63.347	8.76	28835	2	9251.579392	9774.876043	6997.441871	9977.745766	8177.023685	6587.881339	9757.731816	8920.029753	11528.57517	8477.132653	7389.554319	7037.256652	8315.896652	10278.85202	9608.524944
Q99J77	Nans	Sialic acid synthase	327.599	35.38	40024	7	77576.14375	92563.64304	96097.00272	85245.13318	96046.08197	79148.47409	86978.99639	80388.89903	73601.90939	87996.93766	89411.58731	87665.47803	87874.61299	85028.4459	82637.35094
Q99J99	Mpst	3-mercaptopyruvate sulfurtransferase	187.533	30.3	33097	6	65142.56027	60698.20204	60453.17588	60081.7204	55989.13441	49794.30931	58746.63067	54770.21818	56279.31698	59994.26836	58126.79721	54901.15846	56509.31888	59913.79799	52377.72349
Q99JB2	Stoml2	Stomatin-like protein 2, mitochondrial	288.499	29.75	38385	5	14693.69919	25223.39586	14177.76892	18026.65941	11071.81118	16576.10015	18767.82836	19151.38141	21461.41524	19296.54518	21126.24813	12265.64912	19588.63619	13352.03001	12935.59662
Q99JF5	Mvd	Diphosphomevalonate decarboxylase	73.384	6.23	44072	2	21556.07728	24693.05688	24687.79566	25553.85418	21952.63762	23356.62474	24522.93041	28409.26629	22630.86959	21205.50446	21989.96301	23927.89443	22073.01913	22155.64104	25353.02995
Q99JI4	Psmd6	26S proteasome non-ATPase regulatory subunit 6	393.691	33.68	45536	12	113254.2701	129962.8742	116236.0313	102879.3771	112143.6191	111582.9208	117165.2365	139423.3405	132595.7049	99272.23612	102577.7527	114629.6652	115409.84	119649.3723	103304.854
Q99JI6	Rap1b	Ras-related protein Rap-1b	626.584	80.43	20825	13	509211.7448	512683.6953	453774.7747	480620.2428	490902.295	479173.1208	499710.3566	506360.6704	520948.993	501809.177	466011.0798	460353.6864	498722.0003	506074.2931	515564.4705
Q99JR1	Sfxn1	Sideroflexin-1	273.499	30.75	35649	6	74732.2585	74998.20077	71264.9535	74092.53006	65795.31963	77858.88839	64524.83363	68540.19613	66621.57241	69183.56561	72946.84	73343.21972	75146.18423	74248.23294	76489.32948
Q99JW2	Acy1	Aminoacylase-1	89.298	19.85	45781	5	33682.68477	38804.21831	38384.74196	39237.20501	32233.60964	30820.72165	19988.17557	24989.8978	32478.31335	34372.02303	39240.99048	34844.56343	36662.48333	32940.9037	34401.16773
Q99JW4	Lims1	LIM and senescent cell antigen-like-containing domain protein 1	34.969	7.69	37240	2	24398.78617	24173.75336	22941.98403	22582.62983	25361.73822	22362.97121	24284.18038	22087.84307	22162.95554	18647.61564	20881.8039	21986.85912	24160.94024	22842.81433	22739.86092
Q99JX3	Gorasp2	Golgi reassembly-stacking protein 2	96.524	9.98	47038	3	6191.860804	6041.790678	3955.724156	6177.279395	4914.907622	4829.001803	4824.298402	4733.058857	3303.055736	3271.995488	4282.398726	5458.944182	4812.312709	3754.214105	4619.110845
Q99JX4	Eif3m	Eukaryotic translation initiation factor 3 subunit M	280.026	29.14	42517	7	51058.67109	55935.68	53597.60952	55846.39927	45049.92725	28053.96321	40582.58095	41461.81936	41937.51788	31246.76752	25883.91172	51104.59648	50025.30989	38037.12133	25489.5932
Q99JX7	Nxf1	Nuclear RNA export factor 1	65.96	10.68	70300	4	18531.4656	19246.83196	13444.7742	19424.92255	18437.9572	16950.55269	24065.29905	22410.418	16583.20997	17491.46338	10165.26878	20238.74583	26215.66138	20284.51513	21110.88613
Q99JY0	Hadhb	Trifunctional enzyme subunit beta, mitochondrial	258.273	27.58	51386	10	166500.6405	169990.9976	158834.7556	172069.3745	136314.6024	152056.0155	155067.9886	135265.8478	144188.5183	164761.0416	161557.3498	167554.7054	158639.8633	158630.6926	136407.2036
Q99K01	Pdxdc1	Pyridoxal-dependent decarboxylase domain-containing protein 1	79.299	4.96	87335	2	5005.206791	4527.456635	3869.508766	4917.833514	3397.790343	2090.927698	3484.980556	4099.846973	4376.517788	3099.981924	3683.405294	4177.401407	3470.873965	3335.798548	3497.467481
Q99K23	Ufsp2	Ufm1-specific protease 2	49.549	6.29	52515	3	16229.62181	13462.91115	16262.15144	12496.97571	10007.45785	18608.37153	17178.98261	17897.70526	13558.13104	16437.46863	13479.21739	12202.76526	13512.94757	15950.61322	14410.3061
Q99K51	Pls3	Plastin-3	1198.189	64.6	70742	32	2458405.179	2475906.111	2347985.393	2353383.561	2565008.717	2528828.696	2547802.585	2578784.961	2561919.702	2399089.713	2492664.038	2687388.77	2597054.146	2686812.079	2507147.237
Q99K70	Rragc	Ras-related GTP-binding protein C	126.702	14.32	44121	6	40207.97437	45762.14365	39300.82756	43317.26852	47978.67812	44997.26599	45393.33962	50668.66666	55818.24514	51587.81848	44895.78574	46724.78966	48729.38034	54034.91459	50967.58273
Q99K85	Psat1	Phosphoserine aminotransferase	655.923	51.08	40473	17	511328.0847	539010.5819	603198.3638	611697.4712	554838.7139	579406.7177	658472.8111	701289.7201	647546.0576	545907.4673	546030.0639	642459.8325	537532.9226	585443.8101	576908.5389
Q99KB8	Hagh	Hydroxyacylglutathione hydrolase, mitochondrial	59.517	6.47	34084	2	22202.20039	25521.82013	24418.60556	25122.12218	18519.77887	20014.59368	23712.45992	24258.10922	23531.21936	19404.75172	22118.70977	22267.98202	24192.89413	29004.44465	20282.99689
Q99KC8	Vwa5a	von Willebrand factor A domain-containing protein 5A	1382.493	55.23	87143	31	717709.2009	720349.3851	657710.1617	690039.8631	655780.7881	551110.5814	590959.2084	552164.7162	580819.7101	609276.6581	649041.6313	679081.8408	673884.6698	707318.0227	685712.4741
Q99KD5	Unc45a	Protein unc-45 homolog A	74.061	5.83	103447	3	6155.230455	2346.157231	5950.641843	7248.355393	5820.435568	5157.32276	6224.970754	6861.490613	6948.26538	5631.215178	6490.016768	7832.670524	7220.37006	6093.519236	5539.764612
Q99KE1	Me2	NAD-dependent malic enzyme, mitochondrial	499.073	36.16	65799	12	85927.41244	111949.2605	98919.98368	99305.54476	103528.8665	103595.1185	92741.07842	94352.31396	105700.889	96971.50017	97393.94258	87358.85696	101328.3832	89576.83178	94026.62429
Q99KH8	Stk24	Serine/threonine-protein kinase 24	344.392	27.15	47954	10	139273.3461	131298.4875	132188.0524	115453.2628	132914.5075	91511.98541	103310.3103	96646.42387	97519.90438	108391.669	117413.8963	111806.9199	120699.8177	132716.4639	114348.8716
Q99KI0	Aco2	Aconitate hydratase, mitochondrial	1271.939	51.54	85464	31	940083.1028	988989.3201	872352.9549	875675.4735	925361.3954	961124.4919	942790.8951	961146.1657	951453.9197	974113.3807	926720.7832	885634.8543	961801.3879	906300.0607	857913.705
Q99KK2	Cmas	N-acylneuraminate cytidylyltransferase	187.57	18.06	48058	7	77929.58003	77548.38347	72290.65515	69720.73833	78904.26931	77408.08918	75014.32521	77435.52031	86314.1097	72866.13108	68257.94917	72062.637	76849.41741	67582.09508	72530.41225
Q99KK7	Dpp3	Dipeptidyl peptidase 3	518.188	34.01	82898	15	124733.9895	118377.2457	129852.645	143107.0934	106460.1137	92571.03532	132793.1072	126541.1731	95514.65424	96632.60132	125852.886	124455.2702	100175.0798	125787.5108	109658.5349
Q99KN9	Clint1	Clathrin interactor 1	207.204	12.68	68513	6	72615.98438	73984.84463	69434.62248	72239.43062	84223.58925	69019.51911	65116.82565	49428.00723	71206.96398	66902.86989	71750.49814	70278.58943	66171.194	70887.79027	51797.02214
Q99KP3	Cryl1	Lambda-crystallin homolog	80.371	14.42	35209	3	24193.85787	23467.25925	23765.35031	24435.02998	22797.20795	18629.25614	12953.148	14135.78201	12766.41696	17568.50401	20028.67408	22425.957	18486.00601	22143.91518	21839.77382
Q99KP6	Prpf19	Pre-mRNA-processing factor 19	539.892	46.23	55239	14	285090.3061	323675.3927	325526.0439	327174.4358	324355.7846	279633.0138	287707.9747	305676.4746	275339.0743	270524.9108	266881.024	289509.1399	284170.859	284216.2916	301170.3806
Q99KQ4	Nampt	Nicotinamide phosphoribosyltransferase	728.798	53.36	55447	17	252051.1223	277601.8892	257464.1542	249521.344	245476.2302	250097.4759	273822.2885	275361.6075	244265.5574	248184.24	240407.9453	279535.7039	267531.2066	254260.9908	276353.9296
Q99KR3	Lactb2	Endoribonuclease LACTB2	75.062	16.32	32754	3	23652.61025	28213.68279	22116.24028	29499.71694	24835.59976	22023.2179	22623.27666	27911.28582	28218.43009	25405.00641	21464.83146	25901.63288	23271.57601	24153.82707	25812.86151
Q99L04	Dhrs1	Dehydrogenase/reductase SDR family member 1	130.991	18.85	34005	3	11455.43519	7961.940962	10737.01778	10087.20977	3149.19695	9668.337768	6422.077838	6516.919765	5787.446905	4621.604085	5395.838307	6077.158126	9538.884385	3411.373047	11405.02766
Q99L13	Hibadh	3-hydroxyisobutyrate dehydrogenase, mitochondrial	131.632	21.79	35440	5	34142.14411	37265.00581	35100.03073	34098.88096	29588.80755	36387.34565	35059.62095	29998.34143	32260.77311	36033.1569	36959.2468	33177.92608	31679.32386	32612.5654	32293.30471
Q99L20	Gstt3	Glutathione S-transferase theta-3	18.425	9.13	27403	2	10323.5151	10368.88584	10438.02835	9516.671969	10549.69096	9071.177301	8772.522703	8717.683762	8214.833911	7693.024798	9470.213921	8767.296378	8948.558386	11865.88113	10579.97521
Q99L27	Gmpr2	GMP reductase 2	77.828	12.93	38019	4	29816.48403	26110.07587	25664.62823	23616.77035	33714.04357	26890.58115	16352.71379	30153.2403	26135.62575	27624.50586	25655.94052	26023.52413	25307.06397	23952.69545	27149.48217
Q99L45	Eif2s2	Eukaryotic translation initiation factor 2 subunit 2	291.272	34.44	38092	10	228361.6786	231838.2028	211261.1332	244179.4329	246424.6384	222897.9022	242547.9996	244578.9644	252520.589	229366.2792	212662.3574	236444.6194	209645.3952	235630.4509	237485.5419
Q99L47	St13	Hsc70-interacting protein	256.881	18.87	41656	7	169446.4912	171326.2933	168164.2604	169779.1298	170954.6681	173191.2861	169545.1421	160684.8844	150627.3444	158014.7799	160469.6668	169985.5745	169112.0679	170428.9893	163871.0623
Q99LC3	Ndufa10	NADH dehydrogenase [ubiquinone] 1 alpha subcomplex subunit 10, mitochondrial	161.675	23.38	40603	4	19454.32469	23725.07145	15569.96972	23797.89736	19127.11906	19524.25712	19219.43984	22276.9562	24350.18148	20452.46434	17080.4876	20952.9504	22750.87737	19515.0315	22361.19028
Q99LC5	Etfa	Electron transfer flavoprotein subunit alpha, mitochondrial	501.239	49.55	35009	12	260841.2412	192195.2937	226782.0222	239808.2465	156053.3083	235570.6751	171777.3387	152130.2065	218708.4208	168130.1298	232891.7836	156956.9594	159094.7973	161777.9894	172658.6951
Q99LC8	Eif2b1	Translation initiation factor eIF-2B subunit alpha	92.162	13.11	33816	2	3512.798937	3179.959882	2461.984492	2837.929513	2080.997396	1292.416526	2237.034873	2292.772933	2667.838128	1627.407889	2629.029701	2930.443938	2356.91581	2030.256775	1759.706406
Q99LE6	Abcf2	ATP-binding cassette sub-family F member 2	50.613	3.82	71782	2	6521.321956	4265.196332	5457.900923	3683.899901	4530.064728	11766.38633	11075.31555	10454.9099	5431.470689	6027.57692	5102.395396	7473.353807	7558.881547	5302.851656	7561.443891
Q99LG2	Tnpo2	Transportin-2	168.214	7.22	100456	7	87194.59714	84010.84581	90662.40823	85229.47811	83601.23619	94278.70568	90269.14235	83561.34201	85555.39384	87078.7572	83062.8325	92808.50451	91450.72175	95005.76363	90875.55537

Q99LI2	Clcc1	Chloride channel CLIC-like protein 1	184.009	12.8	60621	4	7733.453423	11044.07154	7725.123494	8829.433025	10905.72837	9741.413928	11869.66973	11226.12359	12429.85234	10489.85952	10230.78274	9863.58175	10726.90606	8024.144443	9703.090942
Q99LI7	Cstf3	Cleavage stimulation factor subunit 3	153.709	9.48	82877	4	8653.557719	8556.729683	10232.54756	10217.92207	14198.91063	6701.201773	10129.46411	9705.430097	9852.585238	6382.674721	9829.197947	9257.956126	11488.02668	9743.902733	7426.775984
Q99LQ7	Tmem189	Transmembrane protein 189	104.394	21.77	31134	3	19567.56226	21677.33376	13567.29997	14117.01907	14586.7158	9483.706888	10776.64609	10662.06835	10503.17219	10108.16117	14959.00247	11171.86013	12854.65242	11397.37506	10792.03357
Q99LR1	Abhd12	Monoacylglycerol lipase ABHD12	549.044	55.03	45270	17	374591.2879	364582.1629	348633.888	360253.0659	317193.7154	353670.6611	321500.1421	302407.1256	356070.5747	379737.3364	361798.1173	364681.8968	389359.7791	393733.9242	370369.2818
Q99LX0	Park7	Protein/nucleic acid deglycase DJ-1	481.906	74.6	20021	12	573078.3799	470588.1436	426510.2978	459596.038	425496.7685	324779.1782	331276.108	319643.7082	327876.9155	306214.4868	456219.3902	436258.5266	430644.3211	411650.8887	379036.7234
Q99M31	Hspa14	Heat shock 70 kDa protein 14	123.625	13.36	54650	4	17012.28666	18290.51912	20795.44826	16746.37563	17288.80236	19484.98694	16698.03578	21370.64558	23323.67813	18664.84697	23228.49628	16445.27574	15698.05247	17051.03564	17711.78236
Q99MD9	Nasp	Nuclear autoantigenic sperm protein	209.463	13.45	83954	7	101019.9326	110625.5806	118951.9306	125881.7706	120195.4667	105976.4687	117968.9863	126806.9996	108968.5389	107001.9606	92105.97962	85579.9131	112581.6197	98975.88488	89287.31386
Q99MN1	Kars	Lysine--tRNA ligase	513.808	35.63	67840	14	111457.0462	122532.6646	109003.3872	114330.8573	114660.9781	107824.5353	127384.8213	118146.5216	116260.9233	104054.8759	107346.4559	117567.7171	111806.6709	109123.7761	116356.1713
Q99MN9	Pccb	Propionyl-CoA carboxylase beta chain, mitochondrial	169.033	11.65	58409	4	12358.78553	15073.0402	13106.10464	13387.43256	12785.83208	13950.81846	15296.34505	12089.06537	13083.06664	14923.93408	13474.02343	13407.52031	12710.72007	13254.18053	12829.03326
Q99MR3	Slc12a9	Solute carrier family 12 member 9	175.845	8.42	96330	6	28549.67775	28856.64694	26037.73795	25228.61704	26753.09925	20452.63786	20315.61543	19949.1979	22278.52256	20193.88621	26352.86719	19055.47097	29987.21456	25485.91326	27363.3134
Q99MR6	Srrt	Serrate RNA effector molecule homolog	64.149	4.8	100452	4	31338.33139	28050.43103	36858.39783	34240.05176	31193.83898	26261.02419	32389.46718	29655.95935	29043.67708	31549.10912	31660.49158	30713.76056	28502.75776	33356.77933	36862.27435
Q99MZ7	Pecr	Peroxisomal trans-2-enoyl-CoA reductase	75.716	16.83	32410	3	17014.98182	16037.68305	18614.96089	8897.07849	15503.94304	17862.71246	16329.04177	16755.71708	18574.30291	20333.20371	20484.48773	17178.18674	18237.72985	20253.80094	15637.5609
Q99N69	Lpxn	Leupaxin	478.931	30.57	43478	11	99649.40868	90968.52538	87268.79418	84657.23792	83898.04666	74983.5122	68773.81391	64378.19034	68716.70482	73206.24208	91816.98437	93845.04206	86093.03425	85561.61324	81387.16464
Q99NB9	Sf3b1	Splicing factor 3B subunit 1	1219.249	29.6	145816	30	323856.7468	343430.5702	339384.8846	344451.5111	341145.9925	300631.6538	328514.841	324518.8758	315960.4969	297502.7382	296203.5979	327587.2497	322500.9546	312183.9077	322210.9985
Q99P69	Nuf2	Kinetochore protein Nuf2	53.86	4.97	54594	2	4133.179255	1494.940832	4474.485113	1915.952044	3596.901701	3539.497889	3511.051642	3736.717352	3946.322389	2582.892269	2545.600668	3570.22411	4059.344003	4589.28634	3462.046201
Q99P72	Rtn4	Reticulon-4	446.038	17.13	126613	11	374268.8485	380447.3246	258865.0221	368173.8547	359360.3416	271339.9193	401321.8641	419156.7489	270504.8495	362248.7568	366683.5822	368390.1515	263008.2794	348396.8657	362609.0396
Q99P75	Rab9a	Ras-related protein Rab-9A	81.889	23.38	22896	3	21995.55073	24661.74092	20612.38235	19658.46241	17092.917	21278.23311	20377.4643	18229.40525	19577.54574	22053.76893	22362.45396	22931.71331	22836.8735	23881.21498	25205.95607
Q99P88	Nup155	Nuclear pore complex protein Nup155	424.053	11.36	155118	10	65013.47673	73014.12539	70157.91351	71011.94718	68783.33258	66070.34616	67601.34481	71324.50147	70472.33031	61928.61259	65161.20363	65538.756	70641.8745	68702.52002	65203.09564
Q99P91	Gpnmb	Transmembrane glycoprotein NMB	122.195	7.67	63676	5	64259.76755	57019.7712	49166.14965	50335.48309	43277.227	58265.46907	53546.00906	54398.68864	61373.20164	58498.99746	65384.24639	58527.1696	65485.02535	70067.00363	63149.53156
Q99PD4	Arpc1a	Actin-related protein 2/3 complex subunit 1A	94.403	9.46	41599	3	126147.932	105096.0632	109377.8497	96947.15878	89745.76163	55596.61619	88283.96461	87018.1568	95804.59511	43750.91982	64057.26285	89043.55201	70425.09232	76764.91952	84223.36496
Q99PL5	Rrbp1	Ribosome-binding protein 1	1019.678	26.48	172879	33	501413.146	511903.6775	483778.9066	482342.1725	494348.2655	429330.1068	418403.3602	456756.1224	420112.9844	401703.8964	492492.6555	436836.6632	469057.717	490035.509	474241.7415
Q99PV0	Prpf8	Pre-mRNA-processing-splicing factor 8	1095.879	22.96	273616	36	264276.7892	259784.1421	250333.9414	244245.2631	247363.5573	247009.5133	260656.1231	259987.5745	257841.4359	250794.1175	242153.037	257028.0287	254408.8916	256006.9337	240657.2798
Q9CPN8	Igf2bp3	Insulin-like growth factor 2 mRNA-binding protein 3	217.889	15.72	63575	6	42357.79138	42273.69204	40665.61008	41025.00619	39269.48397	39419.74077	46167.11045	37794.96296	39866.69422	36999.90943	43574.16916	40783.92706	38436.04109	37917.52937	44087.344
Q9CPQ1	Cox6c	Cytochrome c oxidase subunit 6C	35.183	19.74	8469	2	100355.2447	98210.77865	112952.6182	107750.9206	106395.3837	135597.8641	127919.0904	124755.6511	119428.3898	129961.4006	103648.7313	109448.2816	114148.1088	113833.7046	110586.8489
Q9CPQ3	Tomm22	Mitochondrial import receptor subunit TOM22 homolog	166.177	30.99	15537	3	27935.13143	29393.94145	25002.11745	25732.14731	22281.81305	20025.87293	20461.22092	21586.37365	23880.10148	24786.53514	29222.49646	28619.10903	28218.87412	29483.67539	24239.96636
Q9CPQ8	Atp5mg	ATP synthase subunit g, mitochondrial	123.324	41.75	11425	4	72786.12967	74369.77915	77051.64317	70736.2638	66382.71637	69352.87309	55352.61552	57404.10648	71174.38717	66478.98158	71283.33904	77016.5681	65921.80791	82992.42033	74867.03846
Q9CPT4	Mydgf	Myeloid-derived growth factor	56.41	15.66	17982	2	56882.23783	48746.09896	53473.79991	65702.25419	58610.3912	68228.57583	46448.75136	65188.58439	62996.19384	54761.18651	57592.33382	54851.37125	63991.51981	55718.02011	36686.44512
Q9CPU0	Glo1	Lactoylglutathione lyase	134.222	36.96	20810	6	178627.9534	167736.1575	164935.6815	166512.401	160438.9581	132832.9031	131441.8137	119843.6569	128584.3708	133723.1679	159058.4372	146695.1343	152242.1571	154688.9309	153484.4752
Q9CPU4	Mgst3	Microsomal glutathione S-transferase 3	204.663	49.67	16958	4	50027.70973	52003.84395	44488.04532	53510.26697	44316.77862	35679.02841	40186.73518	46024.51953	41468.64924	39186.91095	41950.68306	39625.73374	43043.71101	48971.09674	43873.67077
Q9CPV4	Glod4	Glyoxalase domain-containing protein 4	452.641	66.78	33317	15	198983.1153	199681.6315	178733.1072	186490.8433	200372.2649	167532.6288	149271.7754	149347.3106	169196.5859	146394.7949	187546.0179	181508.437	189451.3177	192369.9478	186914.3176
Q9CPW4	Arpc5	Actin-related protein 2/3 complex subunit 5	182.71	39.07	16288	5	442979.4131	412087.5644	391948.7091	413590.2604	440089.7787	372011.0478	335402.0542	349301.5839	375372.6494	345941.9709	436215.4729	396618.4554	400424.5449	410548.6199	394048.014
Q9CPY7	Lap3	Cytosol aminopeptidase	132.337	16.76	56141	6	35557.21981	38276.87215	37672.37544	36708.13561	34151.20625	29639.4211	36315.51016	33404.01624	36683.24136	35175.10376	31910.82176	37279.80264	36636.52187	35576.10185	34236.17551
Q9CQ22	Lamtor1	Ragulator complex protein LAMTOR1	139.49	52.8	17749	6	58931.75525	48294.85339	43024.01255	51479.50949	50998.72954	34829.04628	41706.6502	38523.67051	40918.35972	44919.71294	49638.93359	47541.21107	51068.78232	43009.21032	51269.52153
Q9CQ60	Pgls	6-phosphogluconolactonase	418.295	71.6	27254	9	261334.9694	155916.616	225142.075	227043.721	120274.356	152244.335	125528.1912	126062.6908	136705.9052	108387.9793	224898.8392	140980.9543	140869.9942	107401.3133	131007.572
Q9CQ65	Mtap	S-methyl-5'-thioadenosine phosphorylase	143.254	19.43	31062	3	19320.21075	41790.03018	39175.53439	46495.98221	38410.96293	42465.31622	40623.19508	40308.41487	43253.68872	42483.99022	41150.35787	50869.7081	42422.48527	41717.4365	39681.31263
Q9CQ69	Uqcrq	Cytochrome b-c1 complex subunit 8	7.134	18.29	9768	2	55513.9268	50841.41747	56158.71113	51839.23227	55326.34891	67675.94713	56518.07685	49149.64474	57435.63669	60304.42226	60707.10736	53470.9243	58595.23843	58329.38356	56747.27583
Q9CQ71	Rpa3	Replication protein A 14 kDa subunit	90.716	33.06	13584	2	9296.02114	7037.357182	7896.559395	8358.313432	5719.65772	4399.438841	5307.540871	5816.908798	6528.74227	6938.950303	8459.47785	6346.706573	6685.683774	6696.926517	7025.032025
Q9CQA1	Trappc5	Trafficking protein particle complex subunit 5	84.532	19.15	20795	2	5412.64846	5116.649169	5741.183533	6583.482478	5249.139694	4934.411714	5547.194253	4708.812024	5466.498555	5232.193528	4679.537875	6141.072825	6575.496574	5343.499117	5606.948702
Q9CQA3	Sdhb	Succinate dehydrogenase [ubiquinone] iron-sulfur subunit, mitochondrial	263.81	30.5	31814	8	160705.2695	148422.0049	141238.8121	155954.1863	135227.7412	134021.2118	156850.9724	127442.1617	154305.0078	165786.2831	149220.4647	132617.3921	159406.9727	167627.0948	148347.3766
Q9CQB5	Cisd2	CDGSH iron-sulfur domain-containing protein 2	60.147	20.74	15242	3	34489.6677	30626.86855	35864.02897	32761.86016	34461.56033	46185.65105	35907.62771	38215.17115	42258.70035	32106.57559	31462.11109	29094.50801	31114.25721	31856.30882	39800.76306
Q9CQD1	Rab5a	Ras-related protein Rab-5A	262.131	42.33	23599	7	203906.2644	203565.2042	194684.1488	187307.7135	212200.0643	205884.8248	209746.4021	220872.3936	209380.2152	195142.3899	214098.6918	201103.8188	217339.5658	211056.942	214440.8879
Q9CQE8	RTRAF	RNA transcription, translation and transport factor protein	265.853	27.46	28152	5	20743.09735	18397.85627	16525.97913	19873.75866	15388.33054	12279.07689	13196.64756	12633.13814	15676.17204	14185.045	18414.51578	16675.80535	18096.2271	15972.56493	16659.75717
Q9CQF9	Pcyox1	Prenylcysteine oxidase	322.256	24.16	56495	7	52687.5842	53158.43199	43054.88649	49148.34334	43824.21609	45059.77986	47902.96015	47536.9003	51490.58617	52726.40475	51380.44058	50056.38877	54013.36318	47805.42321	49072.6936
Q9CQI6	Cotl1	Coactosin-like protein	340.221	71.83	15944	12	897628.1707	889892.6952	824975.9156	873258.0439	886003.7395	705962.7021	755068.0088	731979.3523	743377.659	796632.5463	940624.2903	891602.0008	932798.7983	917097.1579	832501.2607
Q9CQI7	Snrpb2	U2 small nuclear ribonucleoprotein B''	176.405	27.56	25323	5	98521.33131	61916.79836	109933.8756	105094.7397	100236.889	106854.469	105987.1137	119099.4313	105159.9882	113702.8737	64859.20938	70044.88302	109526.9479	110328.8636	104706.2661
Q9CQM5	Txndc17	Thioredoxin domain-containing protein 17	67.76	18.7	14015	2	46324.97861	42006.62155	43406.92937	35760.71475	37039.80158	32985.19252	30134.68829	31044.60919	33196.08465	29255.83248	48276.00669	43181.00394	43021.96362	39541.72662	36401.40157
Q9CQM9	Glrx3	Glutaredoxin-3	341.766	33.53	37778	8	73473.88421	81754.24365	45215.17361	73504.10288	67889.56179	63029.43435	67022.51009	73328.01848	71766.79334	59528.51471	75107.60977	70314.6641	77782.9804	72418.23737	72888.55113

Q9CQN1	Trap1	Heat shock protein 75 kDa, mitochondrial	264.616	17.28	80209	10	57564.62058	44904.73209	48852.24154	46881.73259	62683.74036	71606.72375	71453.44513	81072.50368	81846.6399	69904.1411	65207.8608	60875.17478	74344.66071	50004.60374	82997.66551
Q9CQN6	Tmem14c	Transmembrane protein 14C	81.653	35.09	11642	2	17562.39919	18744.83503	17179.31878	21778.86284	15018.73402	22460.15887	23618.75816	24421.74608	22855.41517	21308.24972	16470.1525	17437.57246	18778.34602	21101.99914	19760.87981
Q9CQQ7	Atp5pb	ATP synthase F(0) complex subunit B1, mitochondrial	285.389	38.28	28949	8	237900.1802	228282.7331	211247.0014	234304.9057	219122.9361	236991.5624	222896.5145	225139.3109	239884.4596	239751.8691	251364.8049	187823.8493	231970.8053	237125.8954	174873.3585
Q9CQS8	Sec61b	Protein transport protein Sec61 subunit beta	94.284	26.04	9958	2	80802.15051	70203.60653	75585.09978	71483.32356	71364.27677	96883.35081	82466.67255	85527.55331	80376.55088	74913.95664	74093.91362	73017.94089	79219.24513	67034.65812	74582.40673
Q9CQT1	Mri1	Methylthioribose-1-phosphate isomerase	295.974	42.82	39411	8	45829.65862	51339.22483	41555.76212	46690.61137	42792.89727	37142.09937	39259.31847	47487.12464	43087.48777	35931.06766	37630.49934	48152.41536	40322.02986	41402.14323	39323.20224
Q9CQV7	Dnajc19	Mitochondrial import inner membrane translocase subunit TIM14	122.275	28.45	12437	2	11380.00587	22225.84144	17549.46855	24589.36131	8372.546379	10739.07855	21436.9217	8861.42758	17689.86092	9052.47728	23620.21214	16593.35237	20616.34385	10382.20272	10918.79389
Q9CQV8	Ywhab	14-3-3 protein beta/alpha	836.75	74.8	28086	23	1454088.621	1458895.024	1459901.371	1385537.771	1574156.268	1471727.119	1487412.936	1461221.523	1482145.542	1429387.885	1449995.561	1496980.944	1624872.498	1629617.671	1506563.56
Q9CQW1	Ykt6	Synaptobrevin homolog YKT6	149.569	30.3	22314	4	38373.59208	41883.89488	40418.31418	37762.77814	40160.05588	35896.1476	43273.65921	39450.68221	36569.74513	37209.71569	34827.23494	45520.85315	41919.16542	41104.76972	45376.19537
Q9CQW9	Ifitm3	Interferon-induced transmembrane protein 3	116.3	36.5	14954	3	46912.86955	56796.58919	60836.42556	59776.02917	53222.55556	84614.13736	77676.84146	82074.21011	95289.31115	65780.07803	75155.14802	79096.49948	69284.15156	65166.81355	61069.93175
Q9CQX2	Cyb5b	Cytochrome b5 type B	139.1	58.22	16318	4	56081.06681	57676.25695	60218.78495	53721.05561	48768.03045	58352.03904	63961.10109	64612.66152	57286.32832	53153.37002	57980.35235	57809.70218	49948.9561	52689.52806	58238.70961
Q9CR16	Ppid	Peptidyl-prolyl cis-trans isomerase D	333.03	35.41	40743	10	109370.7488	122389.6593	87333.93711	124479.2796	127957.0503	93071.71331	122742.2766	131045.3804	125828.3315	95853.75001	113893.9493	123542.0278	114828.0127	99529.92236	111893.7533
Q9CR51	Atp6v1g1	V-type proton ATPase subunit G 1	167.366	22.88	13724	3	23783.66194	22817.31688	22551.88484	17999.63675	14747.94345	14885.68853	14350.52448	13876.21818	12094.84044	13721.63816	15319.10595	14009.51686	16050.4664	13940.93538	17459.42334
Q9CR57	Rpl14	60S ribosomal protein L14	119.827	21.66	23564	4	149394.9633	158637.9513	163372.3842	170182.2732	156496.6559	226240.3695	255937.7959	236207.6004	213911.7995	220199.8061	161554.4692	185512.0818	168787.1865	159943.5352	191739.2465
Q9CR60	Golt1b	Vesicle transport protein GOT1B	127.103	10.87	15422	2	21504.44263	20602.43846	17125.07659	18884.62991	15166.38293	16434.79874	18582.28945	16556.41137	22636.02164	21974.56043	19991.50565	20403.08572	18658.39442	19774.33142	19207.88869
Q9CR62	Slc25a11	Mitochondrial 2-oxoglutarate/malate carrier protein	437.786	57.01	34155	15	288463.7617	287284.7155	262733.2762	238642.7643	269426.0275	315060.8926	295406.137	288389.5678	313653.5608	316507.2883	296772.3511	283749.2394	321181.433	312972.018	294270.9648
Q9CR68	Uqcrfs1	Cytochrome b-c1 complex subunit Rieske, mitochondrial	123.906	31.02	29368	5	36550.85006	41817.13388	34639.90051	14590.26751	51679.05932	31689.61279	57234.90577	23563.66801	7500.240535	33828.20309	44310.60576	59073.50198	23284.90996	24276.67674	5712.755752
Q9CRA5	Golph3	Golgi phosphoprotein 3	148.018	22.82	33752	4	14821.99403	13133.79766	6206.538268	12132.90468	9872.188102	9204.26372	10978.73887	5419.583114	9630.123318	8810.61179	11119.76339	10741.53184	10300.65625	8670.992966	9590.633126
Q9CRB9	Chchd3	MICOS complex subunit Mic19	147.242	15.42	26335	3	43563.57471	56217.02834	57571.92766	59528.06087	55401.65089	66257.30618	62873.07086	25334.45484	24720.86656	22469.94318	22490.93318	57562.2787	61908.31808	23218.83858	24168.8421
Q9CRC8	Lrrc40	Leucine-rich repeat-containing protein 40	175.109	14.45	68076	6	28761.75268	33309.70736	30987.65237	33453.36596	27769.77717	24952.75667	27635.68437	31144.8201	27639.38815	26526.01669	25593.23477	32016.2616	25888.16031	27167.7209	31446.02983
Q9CRT8	Xpot	Exportin-T	73.759	4.57	109734	4	11206.6811	12736.18002	15270.96127	11705.44642	5888.80972	12187.66154	15637.75412	16420.93952	10521.21268	12671.95835	6065.557003	6812.943056	6472.855077	15417.3064	14735.35769
Q9CSH3	Dis3	Exosome complex exonuclease RRP44	54.478	6.05	108838	3	17628.19136	18680.11587	11353.47797	12092.2572	5759.737795	12759.68996	14913.87576	14397.91044	14300.39839	13450.73726	13902.18555	17067.80034	14275.81833	13970.88597	13866.54092
Q9CSN1	Snw1	SNW domain-containing protein 1	136.612	9.33	61475	3	18008.39818	17489.69522	16613.98034	19004.03538	17643.49657	16134.83177	15172.84025	13297.86685	14586.26716	13253.49773	17161.75042	16217.39647	15862.70568	13091.5835	16259.21051
Q9CSU0	Rprd1b	Regulation of nuclear pre-mRNA domain-containing protein 1B	106.769	18.4	36884	4	12106.37894	12629.67474	11656.0978	11965.11246	11262.90234	13237.7622	11556.21761	16120.33455	8605.782832	16546.00317	10466.01181	12353.48545	10544.12104	26162.85952	12188.07702
Q9CT10	Ranbp3	Ran-binding protein 3	155.084	15.07	52573	5	63550.54639	61371.43097	68640.73457	62793.51486	67739.76376	49042.82341	58934.9449	57802.75609	59150.08804	52048.86012	56451.10592	63905.69581	55439.52687	62166.53449	68044.3363
Q9CU62	Smc1a	Structural maintenance of chromosomes protein 1A	329.71	14.19	143235	15	151149.5913	135590.3551	139845.5806	135132.1035	139233.5087	116288.6833	132371.555	129955.2456	121814.5325	131022.5666	135804.0878	140375.8364	139596.911	133997.0793	147690.1843
Q9CVB6	Arpc2	Actin-related protein 2/3 complex subunit 2	836.237	77	34357	23	882359.171	883647.3498	729382.4535	845571.5826	847358.7329	734101.8667	753311.6607	849160.7303	840236.6867	767184.5435	834524.1847	866164.8598	817140.6367	842919.8901	795281.1738
Q9CW03	Smc3	Structural maintenance of chromosomes protein 3	732.012	24.08	141556	22	145127.5848	146684.313	140743.6116	145067.0867	153468.4089	129049.2111	116146.0305	138672.4495	122065.9394	121357.479	145409.8568	142646.3214	146072.0797	140312.7888	144375.7627
Q9CWI3	Bccip	BRCA2 and CDKN1A-interacting protein	57.039	14.87	35942	3	21269.50672	19175.28206	23441.53524	25833.81588	22148.28636	20533.87535	19319.73106	19238.9297	19090.259	16430.05261	15763.35086	17241.26759	15754.01165	20080.63408	22106.8291
Q9CWJ9	Atic	Bifunctional purine biosynthesis protein PURH	1231.219	73.14	64217	30	600864.6372	636206.9835	601211.0573	621975.2335	605254.1713	529522.5851	584192.2446	594374.6945	554541.147	553297.88	602452.2107	626068.5476	583257.4446	616298.7871	610294.277
Q9CWK8	Snx2	Sorting nexin-2	876.156	52.22	58471	23	905432.857	893499.181	825001.7065	824161.3843	832571.7726	630772.8047	683982.5359	662358.2003	651469.3883	706675.037	843386.0621	784342.9886	821934.1872	862975.349	834006.1733
Q9CWM4	Pfdn1	Prefoldin subunit 1	39.52	14.75	14255	2	10170.79524	16481.2849	18839.36998	9845.179755	14910.78397	14227.34458	12908.24278	12403.28938	13287.6988	15935.34753	18555.34022	15317.85298	9669.551464	15874.26392	14388.55829
Q9CWP6	Mospd2	Motile sperm domain-containing protein 2	60.307	8.11	59855	4	28423.41173	29344.74604	32853.89284	28912.26648	29434.10374	30539.7582	31750.79175	32744.30307	31943.68087	31372.60998	29840.02909	26463.55037	27657.82777	28851.81696	35791.84646
Q9CWU9	Nup37	Nucleoporin Nup37	59.059	10.12	36732	2	1866.59958	0.007562416	1680.816858	1891.524213	2080.476522	1430.558797	1704.538601	1657.119479	2026.31263	0.013320187	1504.720452	1594.799353	2109.817976	1719.040932	1616.465254
Q9CWZ7	Napg	Gamma-soluble NSF attachment protein	82.443	15.38	34732	4	14265.37962	15062.08226	14128.59789	14843.73847	11183.73504	16606.96632	18063.865	16234.33809	11446.90507	15791.42291	11914.5285	11686.06677	15013.12989	12963.30353	15186.22878
Q9CX34	Sugt1	Protein SGT1 homolog	133.637	15.18	38159	3	13286.65651	19803.67721	19297.96673	17329.04221	17895.67966	17711.10779	21539.22109	21909.40335	19709.14591	16323.91903	17363.37562	22317.33675	17998.29885	14059.20582	19053.13774
Q9CX56	Psmd8	26S proteasome non-ATPase regulatory subunit 8	192.496	34.28	39930	10	169637.3093	131002.3736	164979.8075	180143.5041	153895.3735	197544.7667	188677.1763	196609.7991	201039.0049	172924.9043	173842.1263	170970.2327	173349.9488	130346.4877	152564.1594
Q9CX86	Hnrnpa0	Heterogeneous nuclear ribonucleoprotein A0	472.942	32.79	30530	10	330478.1899	296219.6492	288942.951	297231.186	320041.0393	231355.151	234226.7581	251538.1691	211104.1762	231149.1595	281109.1882	287148.174	292945.5407	290237.0107	277016.9756
Q9CXF4	Tbc1d15	TBC1 domain family member 15	177.005	8.64	76527	5	20123.47332	20864.19446	16885.48135	19406.62391	19672.32478	16421.62568	17664.11094	21157.14281	18761.37337	15690.3843	18735.1898	14327.96093	16780.45852	18780.65689	16744.73719
Q9CXR1	Dhrs7	Dehydrogenase/reductase SDR family member 7	85.807	12.13	38167	3	16834.62764	32060.60426	17095.01099	17410.19616	30170.41819	25672.66394	36684.55357	31997.89389	23899.65693	20330.66455	28276.83682	29588.36095	23789.6525	25718.05931	17527.66222
Q9CXT8	Pmpcb	Mitochondrial-processing peptidase subunit beta	57.009	6.34	54614	3	47811.61314	46585.41091	48062.83756	48500.73961	55850.91272	63387.19339	55529.40862	60455.54373	60356.71089	57968.24796	54373.64477	20319.79156	66006.31514	15298.25581	57447.03391
Q9CXW2	Mrps22	28S ribosomal protein S22, mitochondrial	91.193	5.85	41192	2	12494.32983	6778.261257	7276.079457	6969.689713	13339.16625	15947.76453	9738.640273	17614.3593	16748.92456	9189.377166	15780.08742	14223.62245	7782.922488	14004.47725	9428.768503
Q9CXW3	Cacybp	Calcyclin-binding protein	319.498	41.92	26510	9	144181.984	157075.2234	165668.1163	152672.9489	164778.4514	157187.387	186435.4837	177662.2226	171570.488	163416.4354	158043.172	166055.6368	162106.043	163569.039	160779.6797
Q9CXY6	Ilf2	Interleukin enhancer-binding factor 2	309.961	25.38	43062	6	62226.10228	67973.69969	61724.36655	64797.53399	63570.5389	59068.88023	62469.69561	59643.55968	66078.39396	62234.37748	60728.64763	65188.80547	63697.27513	64454.90721	71074.08474
Q9CXY9	Pigk	GPI-anchor transamidase	60.615	5.82	44895	2	8298.818602	8395.90357	3547.958818	8287.601334	7447.796178	9352.356085	9605.603987	8525.454054	10064.51576	9843.053684	5372.526337	8505.56598	9001.017917	8675.79915	9604.844406
Q9CY64	Blvra	Biliverdin reductase A	693.431	62.37	33525	17	593219.2786	577915.6412	580480.0459	559772.5166	569257.4137	489428.274	540475.1887	502775.379	528682.3123	519917.6514	557048.6395	580503.4638	579040.8941	587149.5068	577200.5369
Q9CY73	Mrpl44	39S ribosomal protein L44, mitochondrial	48.684	11.71	37527	2	2712.518414	2540.138374	2697.452426	3429.194875	2810.215356	1736.7661	2194.818156	2477.789788	3152.097198	2002.4897	2805.43042	2317.933152	2102.621616	2042.230392	2348.222725
Q9CYG7	Tomm34	Mitochondrial import receptor subunit TOM34	221.466	24.27	34278	5	22796.29604	25736.61731	19423.05543	20661.21025	21654.86005	18039.44554	19556.68593	16942.82884	17175.78077	19892.4304	19433.49843	16240.87716	20050.63577	19977.06589	22438.15781

Q9CYI4	Luc7l	Putative RNA-binding protein Luc7-like 1	34.3	5.12	43934	2	17203.95102	18460.17625	18299.54546	16349.03642	18480.78261	15451.8665	16219.53396	18304.04444	15307.30702	15938.45946	18030.46816	18508.91555	15416.32738	15339.39916	15492.00094
Q9CYN2	Spcs2	Signal peptidase complex subunit 2	140.809	32.3	24978	6	78188.3394	74753.21631	84048.77417	91444.21229	89211.93592	109575.6288	65862.67966	91863.78113	100951.5923	103450.0062	88763.44006	86861.24756	90441.83518	91130.60294	81557.43291
Q9CYR6	Pgm3	Phosphoacetylglucosamine mutase	60.972	8.3	59453	3	8896.763153	5344.815759	5073.714522	5351.439855	4510.939101	7317.812856	1906.373854	4958.209722	2716.460892	7724.82761	0.008046421	10570.95269	9211.789271	8248.413357	6012.823204
Q9CZ04	Cops7a	COP9 signalosome complex subunit 7a	72.7	16.36	30224	2	15153.7025	18985.84165	13034.49139	8593.595207	13737.46183	11983.67637	14132.71284	15551.95371	9591.452674	614.8411795	12421.48821	11558.04722	11733.95419	11576.36702	14248.02571
Q9CZ13	Uqcrc1	Cytochrome b-c1 complex subunit 1, mitochondrial	452.613	36.25	52852	15	266877.1174	265201.1912	268892.1269	248631.6736	293467.8568	331607.2716	287737.1418	278864.9971	314058.7168	277281.9219	273024.0801	233185.3282	302277.7366	255513.7477	281356.9541
Q9CZ42	Naxd	ATP-dependent (S)-NAD(P)H-hydrate dehydratase	51.309	11.37	36717	2	16115.39503	10064.28686	12846.95582	14035.35613	8885.33237	12928.42978	12071.12274	11009.37661	12167.72664	9136.363621	16226.51291	9931.870467	11532.88895	9746.120244	9631.249223
Q9CZB0	Sdhc	Succinate dehydrogenase cytochrome b560 subunit, mitochondrial	155.813	38.46	18382	5	73187.82162	84720.94908	71623.95764	79583.2423	75503.87679	74303.06412	72684.76088	69506.00632	72767.98049	85405.29549	81009.34566	64288.31551	79277.47201	76668.70126	76459.75049
Q9CZD3	Gars	Glycine--tRNA ligase	803.059	36.63	81878	22	467091.7891	512203.5727	507391.1568	498378.2141	503981.1321	508063.7022	598408.944	605507.1112	628609.5536	549708.8888	550306.1251	555454.0055	526172.3571	521049.5038	485792.6529
Q9CZN7	Shmt2	Serine hydroxymethyltransferase, mitochondrial	867.461	60.52	55759	23	772784.7134	782844.9464	835945.9477	826641.2508	760690.1439	892420.043	917350.2724	957290.979	954362.826	824391.8691	784334.023	876321.2685	802219.9553	850189.2431	801237.9989
Q9CZR8	Tsfm	Elongation factor Ts, mitochondrial	138.169	13.58	35334	2	6656.118152	5461.141783	5947.490494	5271.855861	5094.663727	6168.23452	16537.06416	16643.3261	17707.89537	4899.971016	6312.222047	5847.392165	4744.612443	5175.811643	5302.75195
Q9CZU3	Mtrex	Exosome RNA helicase MTR4	272.901	13.56	117636	10	45845.29277	53744.49274	51751.82964	43949.6849	49814.83536	44911.18674	51071.49525	52756.25863	49345.91187	43257.29367	46120.32226	53854.14177	49643.93338	46052.58192	50509.98902
Q9CZU6	Cs	Citrate synthase, mitochondrial	529.968	37.93	51737	16	812094.0265	813946.6818	807543.9325	826981.9756	832829.6674	989433.1324	937590.5081	934490.0211	997085.332	911721.0924	836375.5696	867638.4234	858893.113	867259.6004	897497.2101
Q9CZW4	Acsl3	Long-chain-fatty-acid--CoA ligase 3	370.069	22.64	80492	10	53535.97851	62615.14586	50641.9462	50100.6203	46810.46014	42084.26443	52266.4787	43058.3723	48687.27968	40333.59922	38789.52592	53566.04711	44500.9431	40121.58936	44498.50749
Q9CZX9	Emc4	ER membrane protein complex subunit 4	49.036	15.85	20117	2	7122.162136	6559.671068	20810.96131	17763.56452	7361.298634	21147.64936	25802.75868	23558.92755	13215.11993	20077.71753	16723.17321	32020.7132	34003.36579	29267.20796	27391.99362
Q9CZY3	Ube2v1	Ubiquitin-conjugating enzyme E2 variant 1	106.734	28.57	16355	4	96485.03362	103324.28	98687.10482	98098.3799	100579.5088	96331.68901	87473.5713	91078.52138	93090.13474	83945.18989	99141.58496	112084.2592	105850.5549	103252.5901	95605.85224
Q9D051	Pdhb	Pyruvate dehydrogenase E1 component subunit beta, mitochondrial	81.491	12.81	38937	4	38771.0228	43805.45178	36226.67066	40680.32402	35606.22061	41335.36298	36764.98227	37748.76652	33806.30778	37600.9429	37666.13694	36988.83008	36455.06632	38249.35705	40011.30226
Q9D071	Mms19	MMS19 nucleotide excision repair protein homolog	349.104	14.55	113089	9	33712.37156	33838.59625	32941.43876	36716.93987	29411.43669	30982.49509	31757.63898	35246.61464	35355.80579	30015.57265	29402.59145	31876.38736	29105.60639	33297.54009	28627.45258
Q9D0B6	Pbdc1	Protein PBDC1	191.829	34.34	22223	6	114967.6101	132104.2024	124272.3966	114426.0534	125723.2929	129777.5096	149950.6822	134525.1771	109141.7301	108985.1099	101324.1042	106004.9307	112978.8223	92838.43678	134375.5881
Q9D0E1	Hnrnpm	Heterogeneous nuclear ribonucleoprotein M	1288.2	49.25	77649	37	752409.5286	917294.4128	921088.6952	957201.2289	955067.3054	761513.169	789749.4392	655025.3138	796806.9159	794551.2937	879126.9762	903887.1318	900280.5197	918104.6742	831162.2804
Q9D0F3	Lman1	Protein ERGIC-53	302.23	32.3	57789	9	190105.3529	93559.86274	204831.4209	192838.4392	202323.6689	219154.3982	221923.9734	221020.1445	225648.659	222002.72	199669.1418	104674.588	169220.8195	202832.1583	99396.41788
Q9D0F6	Rfc5	Replication factor C subunit 5	98.822	15.63	38096	3	16532.58334	18708.40309	19297.61605	17395.64621	16804.89521	15920.62963	15660.80409	27694.91551	17363.62911	14540.29272	13394.62537	16397.05542	14034.70463	15713.63971	16014.31875
Q9D0F9	Pgm1	Phosphoglucomutase-1	661.559	44.48	61418	17	176870.1501	172879.3413	160053.1757	161825.7103	165271.7744	153971.3142	191907.0309	189968.9393	169068.3086	137799.8911	176082.5073	186107.4451	178918.3598	141420.7692	157103.8971
Q9D0G0	Mrps30	28S ribosomal protein S30, mitochondrial	101.961	9.05	49939	2	3102.694486	4169.40272	3313.108411	3117.938531	3079.477777	2876.125866	3180.495154	3772.717739	3224.748562	2971.976793	2298.503371	2932.827103	2549.059713	2440.171366	2723.34233
Q9D0I9	Rars	Arginine--tRNA ligase, cytoplasmic	833.074	41.21	75674	22	256000.2897	266886.8234	240672.7022	262968.8232	249834.9003	248708.9251	295801.6586	286474.1111	287392.3736	240855.5265	224537.6676	264844.5928	225454.3591	250546.9566	256074.2429
Q9D0K2	Oxct1	Succinyl-CoA:3-ketoacid coenzyme A transferase 1, mitochondrial	775.214	54.42	55989	19	424365.0918	446587.6981	395768.957	438856.3208	371537.9357	406768.0603	357362.8522	416212.8406	401169.6412	397239.023	408481.5845	386500.3765	386810.0608	408159.3812	382495.1134
Q9D0L8	Rnmt	mRNA cap guanine-N7 methyltransferase	87.993	11.18	53291	4	13957.63447	15322.58858	16068.6056	15830.93899	14317.2395	13389.95937	15933.70691	12532.5046	13980.61458	13786.4109	14402.63873	15316.70046	14609.09597	16089.06467	16391.00019
Q9D0M1	Prpsap1	Phosphoribosyl pyrophosphate synthase-associated protein 1	237.635	23.6	39432	6	40023.96359	44008.61229	38522.80694	32699.58378	52338.26984	46129.71644	60094.54688	57719.07689	47588.51314	44284.97606	46319.1815	44459.01895	50831.46448	40782.60641	39554.91555
Q9D0M3	Cyc1	Cytochrome c1, heme protein, mitochondrial	290.737	37.54	35328	8	139998.0205	136890.1831	132550.1711	127368.3332	131719.752	134961.8496	131558.7414	143909.0234	137161.7872	132591.5515	142961.5723	137505.6588	146949.8736	155856.9875	129079.4052
Q9D0R2	Tars	Threonine--tRNA ligase, cytoplasmic	643.285	39.47	83356	22	309372.8027	327070.7661	319719.5926	315266.0879	289338.2006	284391.832	315854.5926	329017.0287	296602.911	284789.9785	288633.0423	330251.5762	277401.7252	308012.2246	299113.3821
Q9D172	Gatd3a	Glutamine amidotransferase-like class 1 domain-containing protein 3A, mitochondrial	48.883	12.41	28090	3	34894.60084	37032.07584	35745.48785	35795.81256	35466.24242	32915.22701	29618.13001	25467.82638	29725.63625	27893.89665	26238.30426	26983.90456	31702.11618	35867.59352	34081.85451
Q9D1A2	Cndp2	Cytosolic non-specific dipeptidase	1008.466	62.53	52767	23	520077.4833	522946.5589	482522.4944	482973.9515	458316.9565	455893.5061	474047.2144	463065.862	503672.2148	444319.57	512172.6882	503315.7064	511001.0876	509087.1964	503901.7081
Q9D1E6	Tbcb	Tubulin-folding cofactor B	49.756	11.89	27386	2	12402.50644	15886.38179	14822.02897	17028.95109	15727.55622	14440.57707	15749.42292	18301.17343	14597.48356	13851.20948	14822.26562	16281.42832	16930.50103	15579.81123	15531.59586
Q9D1G1	Rab1b	Ras-related protein Rab-1B	440.286	61.19	22187	11	233958.7291	197048.3076	191668.63	184045.1667	198168.2233	217117.605	228715.1649	251410.8034	221902.2774	209793.7297	206593.0387	202316.0652	226196.1742	209020.1032	198108.064
Q9D1J3	Sarnp	SAP domain-containing ribonucleoprotein	233.338	31.9	23533	6	129393.4681	125507.1744	122914.6847	110463.4671	118590.6832	115097.2267	115435.9809	109368.3635	115863.839	105835.1921	121620.5571	124042.4449	117677.2004	119929.384	116618.9529
Q9D1M4	Eef1e1	Eukaryotic translation elongation factor 1 epsilon-1	117.73	31.61	19859	3	29622.12247	21389.57817	26165.30161	23115.73003	22411.45685	21766.79218	18573.85544	27235.13641	24286.70745	24449.98397	20457.84804	18986.64222	22181.36852	25456.80665	20247.1887
Q9D1Q6	Erp44	Endoplasmic reticulum resident protein 44	96.402	11.58	46853	4	2192.074661	7303.973929	2470.636425	6386.521564	6150.621007	3877.711362	0.012348474	2548.400815	6828.8211	7172.937301	7295.395102	0.009057159	7056.760567	2273.091274	2902.792517
Q9D1R9	Rpl34	60S ribosomal protein L34	137.924	36.75	13293	6	309643.7124	283130.0756	297747.6701	308576.4282	294596.5433	337791.4263	361886.0242	340989.6257	328630.4839	291886.3347	289946.3791	289015.0719	283143.3108	323234.1134	295148.9894
Q9D2C2	Saal1	Protein SAAL1	24.228	5.91	52769	2	1853.090004	1465.299679	1031.272092	1692.176107	1328.239467	829.9210641	1067.450322	1500.176747	1335.615481	846.0014348	1156.97977	1380.571628	1335.939692	1177.219623	1004.652192
Q9D2N9	Vps33a	Vacuolar protein sorting-associated protein 33A	222.192	20.07	67555	7	20163.45742	18745.84272	13081.6273	16214.95603	15841.94228	14517.62259	9752.445867	13543.76047	14560.1323	10657.24764	15795.39775	15959.11006	14410.74241	15116.51915	16548.21045
Q9D2R0	Aacs	Acetoacetyl-CoA synthetase	103.35	6.4	75200	4	37257.81309	42265.84225	40038.18376	43137.7029	34863.55798	32762.2247	38066.90659	30629.15709	37190.95809	37899.32841	35468.47031	45380.06866	38558.04331	40358.41866	41115.69678
Q9D2V7	Coro7	Coronin-7	315.235	17.25	100812	9	118437.8403	121102.0295	125695.318	123258.5415	119417.6328	95824.11188	92243.65718	79608.26344	95687.16875	103900.8545	106738.7259	108213.0753	106979.8297	111279.0272	111385.2641
Q9D2Y4	Mlkl	Mixed lineage kinase domain-like protein	49.704	5.3	54317	2	5529.441856	5616.508777	6068.866141	6034.471555	4996.998966	5239.986972	6761.118346	0.01388636	3556.945034	6518.733771	6001.419988	5243.979296	5975.568708	5132.204915	5810.659772
Q9D3E6	Stag1	Cohesin subunit SA-1	69.939	3.42	144440	4	5503.708316	2947.097751	3816.927814	6124.120239	8376.155459	10250.39953	4636.419086	3664.22757	6761.012154	10537.92701	4173.278678	4024.39202	2842.593056	6168.053538	4556.919854
Q9D4H1	Exoc2	Exocyst complex component 2	32.172	2.06	103959	2	7730.081654	8123.241617	8376.953446	7252.54888	7694.043844	8968.366379	8255.285996	9882.053342	8827.781837	10155.83918	7929.764343	8298.991963	8232.970956	8794.434841	8405.08806
Q9D554	Sf3a3	Splicing factor 3A subunit 3	215.947	30.34	58842	10	92167.07956	99937.35378	100599.4636	113992.196	96919.56834	93932.44036	91470.77854	86494.59939	90389.75756	100387.3489	88756.18219	92754.58724	106141.9787	94642.89551	84974.17917

Q9D5V5	Cul5	Cullin-5	146.259	9.62	90974	5	24450.05063	22490.42957	20335.33468	19927.45776	21249.90942	22355.107	22804.05832	22818.03125	21207.28651	18043.10394	21216.53702	23284.43306	23967.83343	21929.68654	23597.83033
Q9D662	Sec23b	Protein transport protein Sec23B	641.768	33.25	86437	15	125500.4618	145992.5184	124902.1433	108550.4758	124647.4996	105646.1099	115536.2875	97499.75455	113257.3196	98421.98195	117130.5738	98474.1472	124903.7113	105356.1434	112573.1687
Q9D6F9	Tubb4a	Tubulin beta-4A chain	1728.708	70.95	49586	37	4121653.567	4659553.525	4460502.482	4525831.949	4061514.043	3421584.245	3651790.745	3257100.297	3871426.694	3729111.016	4256910.433	4426066.308	4298850.757	4397755.018	3874891.647
Q9D6K5	Synj2bp	Synaptojanin-2-binding protein	154.513	33.79	15815	4	24173.46479	29400.18422	31614.04686	27562.07826	27900.71044	33033.34834	29883.66851	36791.02083	38528.6434	40519.73552	29489.3198	26665.2669	29959.05039	31650.10861	33074.94375
Q9D6K8	Fundc2	FUN14 domain-containing protein 2	56.334	22.52	16564	2	3048.212248	2756.062131	2735.62105	5961.584209	5717.937884	6106.6267	3188.704419	2280.862324	4345.635672	6870.285205	3634.258278	2419.343359	3740.957264	2767.605541	6165.61925
Q9D6K9	Cers5	Ceramide synthase 5	94.804	12.56	48167	4	30907.70742	31845.73334	32422.32327	32311.12793	32721.96898	38846.89448	35765.72831	38267.30401	41143.48228	40217.19744	32694.33194	32151.52122	33980.28893	33492.93171	33240.1898
Q9D6N1	Ca13	Carbonic anhydrase 13	39.131	13.36	29522	2	12410.83664	14204.66881	14263.54243	12819.07404	12989.07612	15587.38652	15493.15195	13900.93937	13545.58093	14107.88116	14794.57688	12197.15322	12002.91195	3494.915363	12960.06404
Q9D6R2	Idh3a	Isocitrate dehydrogenase [NAD] subunit alpha, mitochondrial	322.657	26.23	39639	10	318644.8698	306392.0361	299074.8742	295934.4837	302773.0318	324588.1543	297762.3246	315752.9725	322394.8762	308177.8291	323754.382	306612.4625	308220.4598	311332.3833	282492.1657
Q9D6U8	Fam162a	Protein FAM162A	163.641	36.13	17725	6	95026.91569	92933.70229	69260.98565	84135.83317	94296.44761	133105.4226	107182.1867	124704.6604	111861.2292	110151.6053	102759.5615	91621.27393	102492.9993	101871.1324	96035.88205
Q9D6Y9	Gbe1	1,4-alpha-glucan-branching enzyme	78.516	5.7	80364	3	12982.4672	15306.39556	12896.00916	11480.66496	10653.17332	13331.27492	12856.84299	13309.03586	15859.79572	13412.7397	13746.56375	12104.80869	12511.06647	13841.55634	11005.23602
Q9D6Z1	Nop56	Nucleolar protein 56	516.266	34.48	64464	14	203796.3112	179579.1576	202598.3021	201164.7375	186112.1704	182913.701	190008.9346	189348.4551	199960.7076	188980.5247	188350.4025	183737.8024	188100.0851	200572.6226	202149.8076
Q9D706	Rpap3	RNA polymerase II-associated protein 3	57.972	4.39	74096	2	5337.970281	3435.605908	2778.422526	7368.340942	6327.237614	5000.898581	4964.328926	5016.776346	5247.050655	6433.998267	5646.965091	6092.158458	6213.03004	5840.461111	2798.512696
Q9D753	Exosc8	Exosome complex component RRP43	65.434	15.22	29949	2	4953.026117	4006.435498	0.009509917	5547.433768	2279.086143	708.9311039	2917.872067	1977.039193	2440.309282	2439.828466	5908.389805	2204.859905	4141.91712	2706.092052	5181.232033
Q9D771	Tmem206	Transmembrane protein 206	38.178	8.86	40178	2	8883.763487	11757.72537	11800.43577	4202.386823	10069.56648	11091.31886	10474.23968	6209.345094	10086.40179	8172.891192	7954.05351	11811.97147	8338.9637	9232.954859	8487.280508
Q9D7H3	RtcA	RNA 3'-terminal phosphate cyclase	36.038	5.46	39254	2	15157.98944	16995.90918	19080.59141	16425.26549	16669.53557	19484.47441	18774.27117	18218.20417	18222.33651	19631.50126	18427.71996	19010.43424	15669.08989	20389.31974	19831.27884
Q9D7J6	Dnase1l1	Deoxyribonuclease-1-like 1	69.847	15.29	35604	3	10409.75178	6806.17886	6249.972439	7030.376006	7942.755273	4531.518379	7547.793324	5245.441709	1911.674095	5336.652964	7087.922163	5479.306352	9494.350804	6095.217878	6128.429913
Q9D7N3	Mrps9	28S ribosomal protein S9, mitochondrial	58.88	10.26	44929	3	12224.01348	12815.41806	8731.817205	10361.62753	10753.6593	10599.63097	12014.53894	10398.08949	12499.70718	14004.29173	12414.2404	13060.61865	11641.37816	9912.527613	11785.98148
Q9D7N9	Apmap	Adipocyte plasma membrane-associated protein	144.322	16.39	46434	4	31443.43722	29273.83138	26421.88869	11892.8178	26737.21021	36212.83171	29712.38282	30622.18961	33369.04021	34983.17652	27838.76956	30665.35684	31542.42029	30236.22272	28268.52113
Q9D7S7	Rpl22l1	60S ribosomal protein L22-like 1	159.879	45.08	14467	4	139328.9233	171224.9418	159173.4418	164198.66	174055.1257	191749.9878	185939.5814	211690.58	195176.0852	183766.5107	158370.6775	171814.9396	151295.2405	157193.1966	164624.4303
Q9D7X3	Dusp3	Dual specificity protein phosphatase 3	374.275	50.81	20472	7	148291.3346	148317.1293	141640.4729	152753.3703	133051.6464	149219.417	139241.9743	133530.9138	139663.2779	143008.0975	162657.199	165191.2034	155718.688	140916.6045	142555.0748
Q9D819	Ppa1	Inorganic pyrophosphatase	187.747	12.46	32667	3	20471.47815	18887.51134	21594.70214	20302.19593	14030.22512	19661.86179	22252.32078	20292.42184	18175.99974	8994.816655	7669.884099	10874.13658	17990.82119	19875.18801	17482.38461
Q9D880	Timm50	Mitochondrial import inner membrane translocase subunit TIM50	274.972	18.41	39776	5	68079.99953	66519.02366	70995.93695	72879.48653	67219.12623	61140.06012	67467.59849	65018.70561	65882.04496	64791.44044	67393.08766	66883.64455	68467.46423	65401.54354	67337.03066
Q9D883	U2af1	Splicing factor U2AF 35 kDa subunit	181.985	22.59	27815	5	84749.36009	87296.8818	95142.88221	99152.24976	89433.36175	81234.56454	90598.33414	83250.50279	77687.90063	84685.96544	92210.1003	91570.68064	88432.33321	88220.03617	89383.53105
Q9D8B3	Chmp4b	Charged multivesicular body protein 4b	104.393	10.71	24936	2	14504.93359	15369.27515	25240.22201	22220.4767	24625.10553	28091.85211	25564.35441	24145.78246	25010.96452	23764.49219	14376.29173	27548.16106	25689.79448	22962.51977	22692.73656
Q9D8C4	Ifi35	Interferon-induced 35 kDa protein homolog	95.421	11.54	31876	2	5421.568358	2601.522767	3424.247298	4108.694553	2552.909748	2630.29268	2874.808122	3695.877152	3729.042672	2424.354738	3493.323688	3519.197795	3250.703991	2643.661582	2733.390691
Q9D8E6	Rpl4	60S ribosomal protein L4	384.919	37.47	47154	12	109170.6515	133289.1271	119445.8947	124354.0574	106323.2062	226246.9124	314981.6049	242831.1439	260499.9743	261649.7768	124665.9102	143168.4213	145259.6997	107633.9108	183453.3997
Q9D8M4	Rpl7l1	60S ribosomal protein L7-like 1	68.976	20.73	28544	3	12183.67976	7610.918694	7249.820197	6565.662428	7556.468458	7499.564929	6064.422439	6199.001076	6691.544763	5501.142687	5935.481058	7633.397755	6189.226534	5422.894544	6187.334683
Q9D8N0	Eef1g	Elongation factor 1-gamma	743.63	64.76	50061	22	1091622.334	1208240.994	1186566.03	1207654.385	1163414.496	1233733.052	1423566.089	1414674.557	1326436.989	1156501.265	1020499.115	1289647.058	1105908.765	1174696.019	1204234.253
Q9D8N2	Fam45a	Protein FAM45A	98.163	11.2	40421	4	24655.0797	23267.53396	25762.34025	20140.3122	21300.74032	21281.30283	24390.95918	23530.08037	24658.74436	21842.25934	23515.88023	22369.64864	23921.925	26021.21064	17953.31087
Q9D8T2	Gsdmdc1	Gasdermin-D	125.849	10.47	53238	5	73452.02684	73776.22783	69398.57038	80269.65355	69351.74677	70770.07461	83044.2539	71821.19572	85015.49603	67489.73068	61969.87644	71860.31817	71252.44485	77237.71286	65863.09562
Q9D8U8	Snx5	Sorting nexin-5	756.615	58.66	46797	23	691269.1011	665168.7971	604437.2017	674641.6535	658027.4485	512366.9594	533513.8105	518518.2893	516594.0921	574943.9296	640394.9474	582502.5601	665874.9585	680936.4995	632299.5947
Q9D8V0	Hm13	Minor histocompatibility antigen H13	110.963	20.63	41748	5	73272.04284	78469.61426	66669.98011	77236.03589	74023.46146	89203.52327	85995.36016	73513.50553	90043.19394	95876.50755	67106.07183	81688.26906	79807.13599	81327.036	78809.78825
Q9D8W5	Psmd12	26S proteasome non-ATPase regulatory subunit 12	597.035	43.42	52895	17	227030.3778	239097.8043	215559.1023	228923.834	232975.0278	225277.2095	243718.0423	255438.7919	245552.2206	219719.6	227598.333	229763.4428	245459.8775	225657.6249	219849.7713
Q9D8Y7	Tnfaip8l2	Tumor necrosis factor alpha-induced protein 8-like protein 2	144.152	29.89	20615	4	62608.36775	57430.05872	60561.8362	66412.57451	40425.73298	30146.53296	44606.63377	39973.58349	49555.48915	46978.60424	53032.70471	54529.43452	43237.3084	59871.92532	62593.38957
Q9D906	Atg7	Ubiquitin-like modifier-activating enzyme ATG7	601.598	29.94	77520	16	150148.3147	151390.4043	124714.2654	129224.1152	122592.169	134211.3924	146328.4302	142420.003	150050.9306	133656.5137	132981.5482	123664.1636	134993.6367	121991.9441	138565.6099
Q9D964	Gatm	Glycine amidinotransferase, mitochondrial	445.082	51.54	48297	15	339687.3749	308252.7836	301052.2333	309414.2268	321063.1954	260286.5893	265087.1508	222447.2457	261917.3315	274804.7135	277046.9044	233637.371	282168.9218	318138.1676	262472.9398
Q9DAR7	Dcps	m7GpppX diphosphatase	396.432	47.93	38988	11	205009.0869	226877.3338	206731.7117	202685.1964	200822.3575	150851.3205	160544.7345	158106.5902	150253.8227	171939.7299	199841.9467	186550.3402	185933.0581	191239.6694	190632.0703
Q9DAS9	Gng12	Guanine nucleotide-binding protein G(I)/G(S)/G(O) subunit gamma-12	95.198	37.5	7997	2	23526.54578	23889.26284	19300.63545	21074.82702	24268.37173	16959.32927	17927.23685	18272.19433	18305.71188	23142.65591	27170.2025	22793.04141	15765.09262	24100.5335	20162.44827
Q9DAW6	Prpf4	U4/U6 small nuclear ribonucleoprotein Prp4	26.097	5.18	58370	2	3648.408265	4608.55031	3390.313535	4944.36382	5685.112091	2937.290949	3030.670006	15487.94801	2997.488571	0.013320187	2297.587085	3223.624733	2851.398664	3512.021598	3802.024257
Q9DB16	Cab39l	Calcium-binding protein 39-like	28.824	5.34	39106	2	10922.07869	12518.21891	10870.96377	11816.95901	10773.31241	10827.95038	13276.7305	9860.958156	10551.37125	9840.161538	11094.93515	11503.36122	11593.69873	11284.14249	10101.8569
Q9DB20	Atp5po	ATP synthase subunit O, mitochondrial	460.684	53.52	23364	10	333479.4861	288904.464	272034.3748	305733.6978	278941.2049	328426.894	186765.4226	201878.3672	292112.5795	217447.962	310913.0989	230599.3642	227395.5234	223121.2455	233862.1312
Q9DB25	Alg5	Dolichyl-phosphate beta-glucosyltransferase	65.493	7.72	36791	2	21050.5011	17219.55061	21381.66097	16433.27415	18519.4627	25269.1318	21130.47117	20877.67461	22067.8185	20739.54101	17833.35499	19180.15635	20356.54275	20778.7649	19057.2029
Q9DB29	Iah1	Isoamyl acetate-hydrolyzing esterase 1 homolog	43.156	9.24	27974	2	13271.95605	13303.57483	12636.57862	10960.77344	10358.72379	11306.77869	11099.59956	12167.58662	12622.56647	11195.25246	12492.53912	11641.34478	12039.38409	11897.20407	10983.82234
Q9DB34	Chmp2a	Charged multivesicular body protein 2a	25.324	10.81	25134	3	21500.65851	21844.01507	24110.44144	25634.9061	24552.20686	24530.60086	22311.47614	19922.53088	21732.5604	26421.82618	27484.38909	25517.15193	23406.32103	28695.08523	2348.513801
Q9DB73	Cyb5r1	NADH-cytochrome b5 reductase 1	238.949	34.75	34135	7	50833.83301	40691.89558	29619.86709	35560.63337	36321.41165	36068.16797	35187.6808	26658.01584	38562.69438	43858.24722	41040.80988	41179.2449	44541.9997	47144.44399	35775.74896
Q9DB77	Uqcrc2	Cytochrome b-c1 complex subunit 2, mitochondrial	615.105	35.32	48235	12	298432.5006	316951.5279	295837.5841	315179.1826	311608.6729	342051.2386	326376.4063	308345.8772	345487.2677	337778.5878	323892.2011	303790.3784	312955.5136	315323.8286	307131.5157
Q9DBB8	Dhdh	Trans-1,2-dihydrobenzene-1,2-diol dehydrogenase	222.785	20.12	36301	4	28410.24342	27982.03087	23396.61277	24996.20064	21239.1762	15873.54613	19958.25213	19763.09985	19844.62333	19340.87809	23906.93629	24712.11537	20663.59647	22917.80783	24428.80568

Q9DBC7	Prkar1a	cAMP-dependent protein kinase type I-alpha regulatory subunit	390.797	36.48	43185	11	182486.7792	184421.2665	163018.349	177498.0442	166918.9286	144450.6972	161187.5837	161366.8852	143138.617	152472.1169	166624.5672	163264.2565	153662.5614	162931.0576	167681.437
Q9DBD5	Pelp1	Proline-, glutamic acid- and leucine-rich protein 1	106.259	4.1	118069	4	16466.57831	19628.50141	22728.88342	20850.13957	20192.25144	23760.49675	23832.49326	24153.26547	23788.61816	23408.35368	17710.47005	23961.70707	22585.71887	20880.37327	21047.80026
Q9DBG1	Cyp27a1	Sterol 26-hydroxylase, mitochondrial	84.312	9.76	60720	3	3882.680393	9873.679004	6742.637279	8620.384596	7486.227255	6355.399033	9884.458834	7128.080943	8330.252187	8466.239204	9098.498445	8393.628547	8978.568999	8614.152705	8630.183652
Q9DBG5	Plin3	Perilipin-3	782.967	66.82	47262	19	315108.1379	269660.3242	265687.1897	243775.6109	171803.7537	171791.4814	193872.9203	162083.1714	191440.8565	171356.692	280040.4626	219768.6318	189281.8519	201163.4737	203304.9526
Q9DBG6	Rpn2	Dolichyl-diphosphooligosaccharide--protein glycosyltransferase subunit 2	1215.901	54.36	69063	23	428064.6722	438585.6035	399526.1733	429523.3288	413754.0248	467918.6472	492544.5865	503378.8802	517681.6452	485466.1601	425454.2862	430167.5869	463780.7691	379817.2184	404797.3192
Q9DBG7	Srpra	Signal recognition particle receptor subunit alpha	79.018	6.92	69623	3	12148.17695	10597.70846	2557.543994	9666.234328	10342.9298	9703.5504	3525.267082	10900.69329	11906.9657	10181.3207	2950.333868	12384.48245	10650.60378	9071.033389	9246.467234
Q9DBL1	Acadsb	Short/branched chain specific acyl-CoA dehydrogenase, mitochondrial	184.214	10.42	47874	3	19535.63791	27819.58262	22676.20799	24857.05665	24922.52354	27266.98021	26945.28427	28843.61426	27762.38796	24991.78257	17305.32069	17265.26453	11643.45576	11562.80308	26004.39712
Q9DBL7	Coasy	Bifunctional coenzyme A synthase	140.069	17.41	62023	7	67641.66336	62754.66865	62494.27996	61465.18597	65058.4679	60434.10197	64135.12813	53234.73398	68685.07655	58727.34543	38348.65448	59846.73433	66268.49064	68198.27962	67596.13432
Q9DBP5	Cmpk1	UMP-CMP kinase	274.558	42.86	22165	8	126093.4819	117765.5597	119006.9645	115942.9723	110115.8468	89070.44775	107713.0599	92997.40797	97209.61508	96887.50973	114948.8592	119060.2645	120724.6607	112875.5315	117061.1769
Q9DBQ7	Scyl3	Protein-associating with the carboxyl-terminal domain of ezrin	45.049	3.95	81333	2	3088.971683	2865.277139	2612.054556	1012.756446	679.2952721	720.1925421	3034.718947	3255.821266	3321.682639	3326.195129	3020.770747	2930.379134	2194.354195	2523.245121	790.2845521
Q9DBR1	Xrn2	5'-3' exoribonuclease 2	356.657	18.72	108687	13	142853.7105	165816.7256	154540.9613	155094.5421	151548.5805	122122.3343	112818.2155	120142.047	138246.0295	120679.3616	156956.4224	153431.9672	139537.492	147670.5113	149244.4333
Q9DBR3	Armc8	Armadillo repeat-containing protein 8	79.415	9.81	75364	4	12679.92418	13185.189	11790.07352	12071.21869	10903.21968	10080.94005	11770.47215	10870.42796	12360.64474	10059.40349	9426.488664	10562.36424	10819.90032	10367.848	10453.00509
Q9DBR7	Ppp1r12a	Protein phosphatase 1 regulatory subunit 12A	35.473	2.04	114996	2	10206.41836	10503.66132	9578.511309	8267.247812	10290.47466	10293.85993	10827.97178	6639.047242	10812.92601	9661.334699	10092.96465	9364.4445	10111.00557	9959.071717	10937.19658
Q9DBS1	Tmem43	Transmembrane protein 43	323.038	39	44783	12	153865.664	137669.8076	120801.7046	131034.115	122269.1475	183788.7207	163469.7546	142555.9782	167571.8053	162111.6032	159009.7564	137798.0964	152078.1858	146368.0613	132779.224
Q9DBZ5	Eif3k	Eukaryotic translation initiation factor 3 subunit K	122.338	27.98	25087	4	21685.47136	25908.25201	22568.33462	24240.06378	24083.94388	21233.09612	28929.06663	30032.43592	25482.8963	23293.04415	23785.41522	21353.23836	20010.73571	25460.99581	25843.3289
Q9DC16	Ergic1	Endoplasmic reticulum-Golgi intermediate compartment protein 1	128.824	17.59	32562	4	44697.59148	51058.30756	41656.63382	41641.53822	44038.70686	65963.71128	54767.60656	49662.29789	59286.35843	62206.93789	49203.47337	51518.07392	50805.59622	49419.69604	50264.75278
Q9DC23	Dnajc10	DnaJ homolog subfamily C member 10	66.229	6.81	90583	4	13134.92381	11979.61894	15606.26551	9435.89862	11149.91218	11355.92685	14743.95304	11342.49128	8616.895802	6983.24039	7880.779261	14010.04444	4443.801647	13606.30625	10754.10926
Q9DC50	Crot	Peroxisomal carnitine O-octanoyltransferase	63.466	6.54	70264	2	2658.638961	2408.318397	1852.484372	2331.643047	1564.835338	1069.345106	1533.74204	1640.714273	1068.803423	1127.148355	1490.486575	1825.328379	1415.018018	1121.53209	1294.305146
Q9DC53	Cpne8	Copine-8	25.264	5.2	64667	2	12733.5568	12822.00398	13883.24796	14853.36125	13616.11592	16920.20813	15440.73267	14954.75693	15531.72421	19596.92705	12785.9951	14138.12287	15934.99747	15681.52362	14941.55459
Q9DC61	Pmpca	Mitochondrial-processing peptidase subunit alpha	145.997	10.5	58279	3	22199.05382	21733.88929	20849.15552	19602.72274	17634.00736	17355.64604	20887.50876	24853.59624	23174.53742	23786.86677	18561.18594	20407.91319	18964.47679	21043.41238	18029.9404
Q9DC69	Ndufa9	NADH dehydrogenase [ubiquinone] 1 alpha subcomplex subunit 9, mitochondrial	175.187	22.02	42525	5	22647.90735	31271.9048	28770.25345	20264.89247	32936.04233	18005.02801	17903.32866	35648.83651	19551.80964	18034.03289	33730.70491	33603.07594	34220.97459	32062.5114	17966.82474
Q9DC70	Ndufs7	NADH dehydrogenase [ubiquinone] iron-sulfur protein 7, mitochondrial	121.819	26.34	24683	4	35733.87213	36724.13049	35412.33642	30270.88294	40367.62813	40372.19941	36891.68687	38238.81267	30401.86185	35484.97127	30183.29141	33707.85822	33536.39542	37480.71976	36255.80405
Q9DCD0	Pgd	6-phosphogluconate dehydrogenase, decarboxylating	927.981	65.63	53247	26	1240975.335	1300887.846	1194093.395	1199735.613	1298398.353	1085546.674	1231885.863	1171810.127	1141856.197	1079662.329	1182057.213	1256914.339	1216214.351	1223287.155	1200731.189
Q9DCG9	Trmt112	Multifunctional methyltransferase subunit TRM112-like protein	42.929	20	14141	2	20419.14221	23479.07741	18968.24125	19881.37755	19688.8193	18004.50458	18226.65957	8422.582167	17219.16768	16621.16899	20081.31578	16468.73265	16671.20401	16655.53369	16764.64862
Q9DCH4	Eif3f	Eukaryotic translation initiation factor 3 subunit F	457.119	33.24	37984	9	134323.6836	163335.134	176201.1867	193391.6402	148879.2536	163862.638	181375.5606	185293.0271	189025.9554	157776.6149	143432.3201	209690.2595	143212.7567	179304.9934	127670.3471
Q9DCJ5	Ndufa8	NADH dehydrogenase [ubiquinone] 1 alpha subcomplex subunit 8	35.209	18.02	19992	2	21063.68998	21067.44195	16418.93194	16663.44707	16673.49595	18684.76727	20454.8985	10923.25168	20615.87118	20123.99519	16577.26846	19480.07966	18787.22428	19174.79476	20932.82165
Q9DCL9	Paics	Multifunctional protein ADE2	428.915	45.41	47006	13	180300.8774	178414.349	190146.4536	181035.0638	186874.8282	151138.1325	157589.9669	148399.1917	165641.7984	141420.9306	167594.8851	175248.2189	137899.4961	176051.7288	153330.2915
Q9DCN2	Cyb5r3	NADH-cytochrome b5 reductase 3	497.727	52.82	34128	14	283453.2653	294919.0044	278342.0178	270390.9974	282047.4155	368462.4353	338930.0133	359832.9712	331704.0625	306692.6282	308968.8905	276042.593	313825.1148	302521.3254	316822.8567
Q9DCS3	Mecr	Enoyl-[acyl-carrier-protein] reductase, mitochondrial	76.994	8.58	40343	2	5608.593063	6161.214456	5579.835278	5509.469985	4457.45639	3686.862173	5527.403106	4250.92626	5408.13918	4482.433973	4789.179894	5370.261006	4769.815671	4898.041407	4357.815283
Q9DCS9	Ndufb10	NADH dehydrogenase [ubiquinone] 1 beta subcomplex subunit 10	76.028	18.75	21024	2	3320.594437	3747.540204	714.2450138	695.3834342	3542.687709	2803.860373	4121.447151	3287.144727	3660.227421	3726.557056	3572.407694	4898.169567	3509.164873	2915.86175	2742.065571
Q9DCT2	Ndufs3	NADH dehydrogenase [ubiquinone] iron-sulfur protein 3, mitochondrial	139.65	23.95	30149	5	44412.19935	46316.00364	43172.52417	52824.48394	40107.07999	49220.51067	48015.09355	50636.72109	44612.58037	55215.53809	44196.31038	44935.25345	54265.57501	48962.92174	49045.368
Q9DCW4	Etfb	Electron transfer flavoprotein subunit beta	409.696	48.24	27623	11	358763.4239	361350.3797	360130.5033	383215.3084	367634.9624	418180.756	380020.2771	361685.4671	376775.9414	418531.4217	365967.1289	368034.35	374743.4925	392506.8696	367464.9254
Q9DCX2	Atp5pd	ATP synthase subunit d, mitochondrial	201.244	54.04	18749	6	124171.9416	136593.1616	133547.9992	130142.8396	133051.1917	137383.5841	120433.964	119822.1191	114113.4275	122074.4647	116898.7967	107086.8265	119856.6587	123263.453	119692.6398
Q9EP69	Sacm1l	Phosphatidylinositide phosphatase SAC1	204.235	15.67	66944	9	75397.61273	66983.19717	65187.14147	65539.49003	56249.32712	66031.29953	91975.42363	73503.25045	94070.472	92704.39828	63167.4494	62832.63073	63949.26129	71829.92538	54315.61353
Q9EP89	Lactb	Serine beta-lactamase-like protein LACTB, mitochondrial	133.758	8.89	60705	3	19403.87258	19688.14807	17869.54384	20970.14346	17101.42464	18582.21625	21456.70859	23451.99911	22154.02591	21368.6934	22734.56673	18049.14357	22881.52605	19129.34523	23245.65128
Q9EP97	Senp3	Sentrin-specific protease 3	71.384	5.28	64403	2	6781.59391	4201.335966	6215.820518	0.009171104	6073.190036	4044.494639	6800.506089	8339.627624	8029.736845	6693.202445	6032.833215	5742.458724	3043.980212	5950.455504	5790.641479
Q9EPB4	Pycard	Apoptosis-associated speck-like protein containing a CARD	197.84	36.79	21459	6	121435.846	113066.2141	120104.6372	121097.3618	114661.8874	99588.12427	109085.4199	108018.9774	106599.748	98520.19168	111106.5887	86345.09816	91535.02293	94924.2027	112737.2363
Q9EPE9	Atp13a1	Manganese-transporting ATPase 13A1	104.953	4.08	132388	3	7144.243183	11186.44253	9200.744721	6460.426903	5113.055759	11571.79016	13700.81724	5514.590114	6442.753143	12432.80105	11636.84651	6666.64276	12567.46594	11474.70191	11533.37898

Q9EPK6	Sil1	Nucleotide exchange factor SIL1	144.397	13.98	52430	4	18492.75864	17384.66839	13199.51224	11469.47048	10678.47925	17843.40906	19053.71405	20450.87388	20563.09095	20053.05188	16030.98287	15871.60318	17278.33049	16558.28942	15264.24272
Q9EPK7	Xpo7	Exportin-7	133.268	3.96	123810	3	4893.946099	4738.025484	3599.363203	6413.403215	5510.040166	4442.534807	4641.935767	6132.099164	8016.231364	5822.794831	5826.71811	6507.419617	6380.322558	6683.161065	5779.634831
Q9EPL8	Ipo7	Importin-7	529.681	25.34	119486	17	129005.5223	144715.1205	137630.6923	143130.4247	130686.4783	134511.7701	157027.8396	163401.8756	144853.7813	128478.3976	122952.3283	143005.4106	130020.1416	132409.218	138102.7583
Q9EPL9	Acox3	Peroxisomal acyl-coenzyme A oxidase 3	28.909	3.71	78404	2	3898.683207	3186.13036	3187.82628	3058.212544	3379.513206	3341.867265	4680.865072	2859.535115	2274.019947	4034.204289	1926.965278	3548.468542	3332.030152	4056.117771	4146.226773
Q9EPU0	Upf1	Regulator of nonsense transcripts 1	301.157	12.63	123967	11	88860.30665	90498.8313	101673.6088	90088.72317	91775.17051	99077.34839	98214.68157	89345.28153	98238.7934	95312.29107	88679.95845	97053.2872	95763.62207	94297.73911	91826.82811
Q9EPU4	Cpsf1	Cleavage and polyadenylation specificity factor subunit 1	85.87	2.15	160818	3	11118.45365	11677.73675	13688.79274	10617.79365	11907.15165	6645.070441	11568.68883	8269.272384	14626.52145	12303.1257	11727.55281	8644.916039	12859.49811	14812.0652	10245.04045
Q9EQ06	Hsd17b11	Estradiol 17-beta-dehydrogenase 11	105.857	25.17	32881	5	25052.31298	22278.56093	25368.69862	57019.64021	23411.23935	25368.28414	22060.28966	26871.0864	27261.68564	26884.2115	54012.0869	26339.79108	23456.10258	24766.46076	23883.25204
Q9EQ32	Pik3ap1	Phosphoinositide 3-kinase adapter protein 1	749.784	26.63	90928	18	266975.6689	270043.9807	263223.2842	283160.512	302728.7929	178296.6168	179752.9588	195094.6584	181336.6559	171775.4654	245921.707	235744.6097	233561.0089	242611.8206	233880.3655
Q9EQ80	Nif3l1	NIF3-like protein 1	100.567	21.81	41746	6	47152.91813	45981.43102	45272.27591	38118.24324	48915.21233	34017.11117	38433.15616	49812.12823	41899.5398	41585.0874	34013.45614	47565.6654	37907.79244	27532.58667	34798.88859
Q9EQH2	Erap1	Endoplasmic reticulum aminopeptidase 1	221.718	10.32	106599	7	60844.84711	68020.6623	60538.69857	63905.74504	60246.6841	61530.461	61986.72209	60871.03749	68995.9096	61706.61835	52738.0488	57602.78232	61235.80225	64220.9621	57646.07306
Q9EQH3	Vps35	Vacuolar protein sorting-associated protein 35	1279.333	44.1	91713	28	346348.6664	358819.8744	328192.1302	361310.1273	353724.7451	314518.529	356612.9788	344909.134	327825.1745	333804.1903	304512.5895	350149.2497	348958.8298	354959.225	348245.5237
Q9EQK5	Mvp	Major vault protein	1063.413	46.34	95924	28	367148.0339	390699.0563	356962.2173	400198.5048	400059.6787	527935.3043	568944.2994	598058.3568	596757.4558	511775.554	410423.1214	452915.4761	452119.8119	382386.8463	450431.1419
Q9EQP2	Ehd4	EH domain-containing protein 4	669.126	44.92	61481	20	420168.3446	462979.1434	417262.5497	432866.1594	426592.2593	378158.824	392368.9981	377937.3958	394674.7	379204.6888	445849.9998	440088.4733	443998.4055	466167.6322	450421.6932
Q9EQV6	Tpp1	Tripeptidyl-peptidase 1	73.575	6.22	61332	2	12148.68595	16234.89761	9425.062847	11115.7284	7988.109546	3880.292613	5557.651126	4377.200968	0.012083387	4596.311581	10086.56171	8186.427321	9518.221765	7454.961499	8307.199631
Q9ER00	Stx12	Syntaxin-12	150.501	23.72	31195	5	74993.54336	77268.74802	67915.8839	72511.94996	58871.53862	42799.9497	49006.61119	42273.75827	39427.00696	42717.2833	80666.31065	69348.15197	74849.0255	51333.6308	67825.93872
Q9ER38	Tor3a	Torsin-3A	116.091	12.21	43814	3	13544.34441	12739.17379	13034.38203	13423.26062	12664.23097	19318.53168	17093.67472	9113.696768	18823.95021	18661.66511	13362.67806	13108.12118	14596.49543	15922.53616	14288.58804
Q9ER41	Tor1b	Torsin-1B	59.065	10.71	37818	3	6755.249606	6598.787172	7178.696143	7946.37098	7103.92508	8170.605752	8513.028781	6997.798285	11610.97027	8051.272091	7326.581541	2906.149512	8499.762172	8286.734606	3307.948627
Q9ER72	Cars	Cysteine--tRNA ligase, cytoplasmic	413.681	19.61	94860	13	112955.7035	126208.2709	135721.7761	121912.1868	114197.2469	115599.6931	138712.3729	134216.2612	105330.074	104262.0114	125289.1055	138644.2343	130320.965	122690.4082	115601.2595
Q9ER88	Dap3	28S ribosomal protein S29, mitochondrial	158.728	15.09	44699	3	8458.774057	7422.498725	6807.773081	6689.06137	5472.082846	4368.757174	6985.827419	6726.493952	7084.180887	5543.623294	5821.669987	7937.1579	3075.023495	5125.17058	5503.757657
Q9ERD8	Parvg	Gamma-parvin	69.736	17.22	37603	3	7429.898564	5866.702793	8177.644596	8245.77138	7387.134072	4727.436797	4956.583716	8431.341891	7211.913584	7690.170948	8147.680047	9095.29407	8679.427743	7732.427619	7595.018116
Q9ERK4	Cse1l	Exportin-2	1083.246	42.22	110455	27	331266.1741	338343.4542	332614.6983	339120.2019	300358.6075	252620.451	316737.0887	342834.8729	319075.739	246367.7928	303023.6154	347782.0343	325336.3864	314453.5806	288894.3644
Q9ERL7	Gmfg	Glia maturation factor gamma	94.209	30.28	16748	4	89190.80789	90764.18134	92031.17065	89812.80135	92360.33547	67325.77061	75566.38845	60387.14646	69690.3358	75794.6874	92221.44576	83309.53683	85202.44602	83972.17277	63232.65318
Q9ERN0	Scamp2	Secretory carrier-associated membrane protein 2	138.647	9.73	36465	2	45216.41	43337.51974	41166.91111	44002.15737	46007.9527	47335.29845	39986.162	41507.58533	42942.69557	44511.51248	46174.66376	41867.02747	42772.76395	44062.80731	46552.41865
Q9ERS2	Ndufa13	NADH dehydrogenase [ubiquinone] 1 alpha subcomplex subunit 13	74.502	22.92	16860	3	22856.24958	25886.89953	24634.33804	22753.73282	21909.72954	22014.60852	21313.08503	23191.23584	25552.30931	25045.11161	25102.80944	24961.72163	24374.54837	23324.77796	22840.58748
Q9ERS5	Plekha2	Pleckstrin homology domain-containing family A member 2	61.286	8.71	47380	3	22137.80258	16435.26382	18090.21553	16679.2913	25949.79209	20212.42038	17170.53934	23108.94007	24484.69224	21622.34139	22053.85964	23396.45201	20168.0989	23747.58981	21977.43786
Q9ERU9	Ranbp2	E3 SUMO-protein ligase RanBP2	381.301	5.6	341121	11	42171.82273	35116.02439	38467.89192	37732.862	32382.56868	29737.74286	40943.86952	34160.15976	40191.3416	30669.28746	32290.02946	44243.91141	34270.63841	35663.76449	40522.50594
Q9ES52	Inpp5d	Phosphatidylinositol 3,4,5-trisphosphate 5-phosphatase 1	932.081	33.59	133542	29	297443.5635	293256.2107	281663.813	289638.0258	294955.0832	266663.5709	280236.3576	260818.7842	285778.6372	274969.1379	281647.8478	307796.7042	298778.0013	316982.5651	297671.7301
Q9ESL4	Map3k20	Mitogen-activated protein kinase kinase kinase 20	250.059	14.84	91720	9	40954.07831	51817.28294	43699.036	45915.66687	39805.87209	41650.19689	48322.27728	53644.33298	75605.16577	44739.10785	40073.76285	43048.69695	38653.30894	70899.97476	50615.33892
Q9EST5	Anp32b	Acidic leucine-rich nuclear phosphoprotein 32 family member B	188.821	27.57	31079	7	435539.647	364419.7222	457289.4119	432698.6951	423061.3819	326716.2285	420580.5443	352709.7965	372726.726	390191.1523	382436.2535	390436.4369	360165.7065	424602.7075	416448.6024
Q9ESU6	Brd4	Bromodomain-containing protein 4	39.623	2.36	155895	3	11625.33575	14784.66733	18651.80231	12868.79977	13171.01801	10157.84738	12441.85326	8813.3973	11169.14576	9119.605228	14227.22173	13165.16657	8706.059441	11229.9943	15915.69017
Q9ESV0	Ddx24	ATP-dependent RNA helicase DDX24	61.412	4.08	96429	2	5352.615215	4436.050662	5672.826246	4586.238505	4320.294256	4292.470482	5893.02142	5611.220986	4199.752157	4996.059914	4262.855982	4119.694259	4534.472416	3934.980027	4915.295057
Q9ESW0	Ddb1	DNA damage-binding protein 1	311.36	11.75	126862	11	174594.6305	191701.5134	177126.9962	188426.2579	186982.7307	168574.7107	191866.1327	187938.7952	188749.9588	170747.7597	174694.5319	181656.5034	169129.768	191358.0816	182001.118
Q9ESW4	Agk	Acylglycerol kinase, mitochondrial	97.478	11.88	46976	2	19158.91699	17068.70121	16613.35269	18842.92661	5913.666191	3062.909654	7880.199614	14729.2303	13966.72775	6585.706072	0.008046421	16981.15956	18288.97456	14598.12143	15001.149
Q9ESX5	Dkc1	H/ACA ribonucleoprotein complex subunit DKC1	330.471	24.95	57402	8	83180.927	96142.7907	84650.01017	83292.90786	86299.01417	67901.50304	86170.76406	71692.49694	84236.65528	79120.51163	75415.52215	87956.33059	87009.59376	81744.80762	83908.96257
Q9ESY9	Ifi30	Gamma-interferon-inducible lysosomal thiol reductase	195.149	20.97	27784	6	57069.81062	56635.61183	52554.76624	59401.0686	51208.97166	20314.94959	26142.32165	21668.51494	20710.46899	23279.28273	32245.56092	39293.1005	35525.96269	40213.24493	40433.51255
Q9ESZ8	Gtf2i	General transcription factor II-I	186.548	14.43	112265	8	43692.66236	39294.1305	44433.71992	43303.08082	32009.53874	36574.78911	37585.30066	38157.32057	34674.36923	38123.71362	38100.01605	42263.78993	33669.9189	31463.06458	36749.46754
Q9ET01	Pygl	Glycogen phosphorylase, liver form	203.488	11.76	97463	7	72803.52014	69524.06298	56791.8006	63253.61079	67253.89244	56547.33006	52484.19464	62915.44983	60527.57602	65875.95008	67057.17776	67028.55004	70612.54766	74851.6136	67884.2316
Q9ET22	Dpp7	Dipeptidyl peptidase 2	42.274	7.71	56254	2	5386.850672	6900.424521	3164.54448	5620.799204	3938.23956	2868.050388	2379.674073	4795.374382	2982.872204	3751.549057	5684.753095	6150.483757	6520.24074	4476.350411	6001.387272
Q9ET30	Tm9sf3	Transmembrane 9 superfamily member 3	118.637	11.07	67545	5	61463.15081	72640.24712	58288.7044	65394.92592	57597.36473	71563.29337	66364.76244	65351.98519	70963.45353	72308.04189	60951.73062	56523.73499	60609.21448	61540.84633	62258.05397
Q9JHF7	Hpgds	Hematopoietic prostaglandin D synthase	113.844	22.11	23227	3	21300.33491	18833.48733	19053.44416	21793.40821	17159.38587	15062.57973	11265.58855	12104.80035	13742.68665	14233.51883	19494.22075	17040.8485	18068.30331	20023.12535	18263.85789
Q9JHI5	Ivd	Isovaleryl-CoA dehydrogenase, mitochondrial	187.424	20.99	46325	4	17074.70295	11029.4619	11142.93315	16861.49935	6330.494363	12264.17454	15308.87567	12656.27191	15756.6474	11623.02114	18867.16518	16771.93464	21793.74203	14843.33377	12307.92243
Q9JHJ0	Tmod3	Tropomodulin-3	151.149	21.59	39503	5	71614.37046	65429.46862	62942.17805	65569.29153	53626.65274	68421.83885	51618.40608	46576.0649	55965.81351	47604.10689	73664.3313	39424.06703	60363.60095	57722.85394	41622.22013
Q9JHJ3	Glmp	Glycosylated lysosomal membrane protein	116.49	13.12	43804	3	38668.29232	38879.3925	37520.72792	41449.47697	33885.00427	28613.00032	31259.50153	28861.57453	28180.40971	18049.56423	26744.76409	30473.34732	32478.64467	34169.87323	30931.03683
Q9JHK5	Plek	Pleckstrin	492.602	47.14	39901	10	149805.4739	164389.8339	134451.7932	156665.0752	149310.9959	146421.1308	158185.4597	175575.1722	154523.6566	149260.3135	138382.4829	139682.1124	137847.3377	138877.7042	143894.6871
Q9JHP7	Kdelc1	KDEL motif-containing protein 1	36.999	3.39	57985	2	4011.583201	0.007562416	4099.330471	0.009171104	5045.208555	0.014539799	5437.742811	5693.785808	5166.203734	0.013320187	0.008046421	0.009057159	0.00981159	4537.933688	0.010360417
Q9JHR7	Ide	Insulin-degrading enzyme	144.95	10.7	117772	7	46306.31717	49719.33648	44091.01102	49964.59449	41877.63524	38232.10451	41874.71911	33556.07882	36454.67806	42136.05029	45855.02372	42404.4929	40903.58243	38286.91527	42771.18516
Q9JHS3	Lamtor2	Ragulator complex protein LAMTOR2	122.42	21.6	13480	2	63019.51176	67790.34136	48120.43913	52760.78603	59629.61707	37186.65658	38524.87445	36012.35711	32379.18426	36442.16323	34775.99533	33020.07338	30344.28041	31049.36154	38933.21544
Q9JHU4	Dync1h1	Cytoplasmic dynein 1 heavy chain 1	4542.941	39.49	532045	139	1803241.012	1864631.21	1698489.68	1762221.557	1796253.417	1842543.883	1872244.24	1796590.214	1744799.9	1787623.976	1856760.288	1839307.806	1910504.842	1894511.833	1862623.231
Q9JHW2	Nit2	Omega-amidase NIT2	293.059	41.67	30502	8	43193.01532	48090.42995	37808.67871	43851.37984	42203.07864	40405.28472	44623.1869	41186.70628	41727.73518	37675.39941	37038.40346	39103.50246	41361.00367	39258.34376	41313.78794

Q9JHW4	Eefsec	Selenocysteine-specific elongation factor	64.403	8.06	63539	2	3667.691289	4153.789622	1042.572537	3257.748521	2618.496239	1854.469266	3554.067552	4119.257951	3250.082953	3186.004291	3607.882352	3165.476682	2654.46072	2371.35711	3284.332368
Q9JI10	Stk3	Serine/threonine-protein kinase 3	163.787	16.1	56855	7	49409.94896	52822.8082	51585.43937	57668.50038	53522.26734	57773.58403	55659.91964	64174.60242	57130.61575	55879.1831	59360.27732	56320.92707	56713.54713	54020.6497	52448.99798
Q9JI11	Stk4	Serine/threonine-protein kinase 4	145.787	17.45	55541	7	69056.54779	69363.37677	65069.64169	73245.0513	66749.98501	70384.58091	64119.45792	75570.21424	65065.90884	69153.24886	73388.16204	65252.55882	70171.28611	79613.49998	66622.34322
Q9JI75	Nqo2	Ribosyldihydronicotinamide dehydrogenase [quinone]	90.129	22.08	26248	3	0.008226335	0.007562416	6165.689606	6425.011539	500.4094793	4811.230097	4697.702957	4972.067892	4337.794279	7384.048944	14116.42553	4831.44262	12095.70629	6668.021466	6709.912855
Q9JIF7	Copb1	Coatomer subunit beta	993.059	34.63	107066	24	174936.3361	210407.92	192515.4882	220996.8089	177319.8093	175942.6814	194129.5216	191454.9881	202802.3577	192729.3582	200669.9878	201982.0734	209831.1286	172368.7304	181864.3605
Q9JIG7	Ccdc22	Coiled-coil domain-containing protein 22	144.224	15.15	70844	6	23914.57996	22741.42993	21830.50292	23684.91395	22343.59415	13352.90287	25208.03919	25791.19803	22879.51548	20012.37036	22148.31658	20234.53878	21954.50984	24519.61011	24849.33499
Q9JII5	Dazap1	DAZ-associated protein 1	130.901	15.27	43214	4	25111.60635	29185.15449	26707.39543	27967.5786	21730.51126	27005.34379	27162.60815	26693.97804	23369.78682	23751.62488	28528.12856	29822.2553	25922.38559	25950.07533	24760.26908
Q9JII6	Akr1a1	Alcohol dehydrogenase [NADP(+)]	477.043	65.85	36587	17	361531.6186	337829.5124	312500.9566	350020.1686	346631.0511	299708.1148	327939.5997	298495.2826	302492.1363	291572.7242	359933.4076	334611.9101	332551.9279	325210.8666	328010.967
Q9JIK5	Ddx21	Nucleolar RNA helicase 2	222.972	12.69	93551	7	61109.28266	56614.16482	50670.66139	67505.71962	57781.36275	61052.23246	75313.04098	74660.90765	72934.79164	65085.93645	57546.96005	52510.04572	58476.4495	56119.36607	49915.54976
Q9JIK9	Mrps34	28S ribosomal protein S34, mitochondrial	30.133	10.55	25827	3	19680.56331	19382.67185	22423.78863	22906.8421	20331.80187	23554.28423	22458.80887	22894.69784	25628.90288	24585.83156	19088.23858	20727.45204	20635.38324	21201.79425	21871.51296
Q9JIX8	Acin1	Apoptotic chromatin condensation inducer in the nucleus	139.162	4.19	150719	5	27774.60066	32323.18267	39848.13757	39456.3256	34946.62159	26987.69429	34747.51327	30813.98106	27270.95964	30392.02444	37485.34593	34630.55407	30348.15108	35247.83448	32598.87483
Q9JIY5	Htra2	Serine protease HTRA2, mitochondrial	152.61	10.7	49348	4	31558.35707	29811.67039	20969.42031	29332.55752	26435.61381	28775.31719	33548.47026	24204.64847	32258.1631	31426.6966	18851.51288	23694.75635	34615.06938	32682.24444	29051.42414
Q9JJ28	Flii	Protein flightless-1 homolog	992.811	29.5	144803	26	196079.6467	199870.3592	174378.7062	197125.0681	153355.3319	159148.7187	180744.4548	186870.712	177964.9318	175879.0686	166312.8328	174672.9263	158331.75	180575.4337	170908.4582
Q9JJA4	Wdr12	Ribosome biogenesis protein WDR12	33.697	8.04	47347	2	4055.733203	5227.738712	5899.246683	4827.798497	7901.136674	7105.441856	6678.111903	10072.43005	8791.195637	3556.335457	4914.339476	4995.620663	6165.153797	7393.053775	5082.519641
Q9JJK2	Lancl2	LanC-like protein 2	141.872	18.44	50777	5	21679.40444	17073.84743	16633.09052	16566.98655	15187.81217	12107.28821	19753.98213	18591.26816	21513.6049	16212.62554	17768.702	21712.33661	23123.78893	20741.11212	19272.8485
Q9JJU8	Sh3bgrl	SH3 domain-binding glutamic acid-rich-like protein	216.988	70.18	12811	6	113699.3313	109711.1484	95537.68643	93171.5438	95131.66306	82456.52563	84742.32345	83181.99444	66018.96786	75597.18899	83456.64043	91840.34851	96079.43753	82149.36875	92021.44853
Q9JJZ4	Ube2j1	Ubiquitin-conjugating enzyme E2 J1	38.103	6.92	34990	2	8976.457833	9181.522289	11141.26297	10220.63213	9432.207797	15221.25619	15199.39564	15515.86306	16475.72371	15182.7653	10106.7019	8348.454288	11882.69422	12972.84261	11413.98295
Q9JK23	Psmg1	Proteasome assembly chaperone 1	110.485	12.8	33104	3	22412.38943	22414.85457	22919.61908	21303.42825	21716.24025	22417.69902	23564.98364	24317.76502	24171.82465	22920.02564	26476.10818	21451.47231	25137.54913	23969.94382	23933.2799
Q9JK81	Myg1	UPF0160 protein MYG1, mitochondrial	343.682	30.53	42723	8	89074.84124	96717.60992	85283.23753	90289.69874	87285.81721	73399.88086	76043.49644	74149.02866	66064.6914	73129.17147	88945.32941	83498.85863	84859.04037	84959.56592	82379.51643
Q9JKF1	Iqgap1	Ras GTPase-activating-like protein IQGAP1	3899.36	65.42	188742	92	2961205.639	3027587.362	2763973.072	2808023.782	2632196.263	2787018.778	3089547.728	3020743.126	3081746.233	2942142.864	3117751.981	3103882.464	3126958.822	3165606.072	3133180.303
Q9JKP5	Mbnl1	Muscleblind-like protein 1	28.027	6.45	36976	2	58187.8971	54004.03485	49679.72324	49618.44953	48126.10385	40890.56144	45078.56776	35025.76598	36354.86324	37364.76933	49519.79024	44657.32546	47642.68786	46784.24728	45826.14826
Q9JKR6	Hyou1	Hypoxia up-regulated protein 1	1066.388	39.14	111181	29	447097.2533	472642.6099	412562.7105	444746.9206	394031.2224	614597.2779	557270.0026	560681.3872	604805.7164	520967.8682	487379.1228	481126.4265	470362.2661	469901.2598	443340.5556
Q9JKV1	Adrm1	Proteasomal ubiquitin receptor ADRM1	72.636	10.81	42060	3	35201.91204	31209.7096	38147.63357	42132.24729	23640.19744	30655.43321	34367.42106	32211.27169	32669.43326	25107.91296	25819.44782	32138.0804	34140.5122	34648.56733	23583.47338
Q9JL26	Fmnl1	Formin-like protein 1	999.021	31.9	122060	29	438853.0833	437525.9881	422320.7988	467524.7872	452210.0738	401627.7178	404440.002	389448.8545	388764.0629	417268.5614	434558.7471	408427.2161	409279.4265	445006.0084	428129.2676
Q9JL35	Hmgn5	High mobility group nucleosome-binding domain-containing protein 5	59.391	5.67	45344	2	4005.673361	245.3484736	1733.518443	5108.582702	10926.01899	8457.736766	3940.301643	7847.523718	3689.885369	1443.154738	13753.73814	400.9657923	7514.141334	4269.450759	11041.33689
Q9JLB0	Mpp6	MAGUK p55 subfamily member 6	112.07	9.04	62631	4	49786.9907	50667.30798	48833.95873	57781.07109	54571.84958	46903.84808	48017.94914	48065.03674	50643.96064	50903.17753	56663.17739	50647.20087	53094.43907	53669.3656	50273.84405
Q9JLI6	Scly	Selenocysteine lyase	111.144	11.11	47174	3	28892.1442	26316.54305	32939.81732	26503.7329	27196.20715	25686.52037	21326.41212	3771.714033	27643.88015	20667.1358	29410.67006	24779.38063	25854.96279	23691.10946	24458.14638
Q9JLI8	Sart3	Squamous cell carcinoma antigen recognized by T-cells 3	85.073	3.33	109619	2	6305.941566	7520.191901	1136.708927	7404.859681	6644.600598	6541.661134	5486.956111	6527.555397	5373.587158	6355.745899	7289.639377	6351.499485	5988.129359	6753.734295	6843.143255
Q9JLJ2	Aldh9a1	4-trimethylaminobutyraldehyde dehydrogenase	687.374	69.64	53515	21	375545.0163	379463.5148	376394.0598	370053.1239	365879.0956	348239.1155	352816.6627	338898.0067	353191.0311	306403.0207	353907.8292	362469.595	350675.5833	365334.0021	358072.0888
Q9JLQ0	Cd2ap	CD2-associated protein	115.993	12.87	70450	6	34345.84463	33689.45785	23821.49211	39097.02927	23564.05648	28692.32765	31709.20517	19641.14984	21796.80475	23095.83046	22207.64284	26803.51312	23572.23256	20095.1141	28572.80657
Q9JLQ2	Git2	ARF GTPase-activating protein GIT2	146.227	15.82	78766	6	20883.39544	22547.99468	11279.09971	19503.89722	20959.65378	13213.25719	15016.67372	10654.36919	16091.97498	20781.95581	25632.42888	19830.39021	18327.9462	11427.52542	13888.23952
Q9JLV6	Pnkp	Bifunctional polynucleotide phosphatase/kinase	136.344	20.31	57223	4	17166.04407	14138.64514	11791.88159	13983.9498	11832.01086	8803.04886	12111.59795	9288.21012	12152.01449	8340.842099	12795.85901	12125.83941	10639.23951	2152.474905	8734.900941
Q9JM14	Nt5c	5'(3')-deoxyribonucleotidase, cytosolic type	114.573	27.5	23076	3	17106.82782	15518.21031	12824.4506	14354.32023	14378.53705	9837.651915	12037.0533	11408.12169	12804.1579	11084.39187	13685.98024	13465.21781	12350.32196	11723.39301	11013.20189
Q9JM76	Arpc3	Actin-related protein 2/3 complex subunit 3	357.381	69.1	20525	13	410221.9812	412733.304	404344.7343	402993.4537	382225.3606	327790.2834	353030.9828	316105.9915	359071.1696	335325.0093	421885.2158	433102.6862	471331.1017	420452.8874	392389.7257
Q9JM90	Stap1	Signal-transducing adaptor protein 1	483.469	61.28	34628	14	247220.7662	251380.5135	199260.9868	219211.6352	189881.4186	268852.5667	291281.9442	354426.3449	352945.5691	255132.0229	238888.1535	268204.6178	233242.9957	222549.5152	220879.5346
Q9JMA1	Usp14	Ubiquitin carboxyl-terminal hydrolase 14	521.642	42.19	56002	15	130956.2661	137082.4499	120723.2859	136934.7572	142035.4695	140207.0386	138882.041	151911.7047	143468.0464	129590.0738	137478.8859	145688.4039	124157.3044	122887.6486	134961.6458
Q9JMD0	Znf207	BUB3-interacting and GLEBS motif-containing protein ZNF207	61.803	5.05	52793	2	10757.63116	9145.103586	0.009509917	8741.47696	11635.88682	11638.12887	8800.164021	8271.968143	9087.723462	9501.309303	9079.997712	12748.73423	10897.86444	0.00945634	9568.13486
Q9JMH6	Txnrd1	Thioredoxin reductase 1, cytoplasmic	304.448	20.23	67084	7	40906.31621	40884.56324	32759.05756	32467.54339	30314.44045	42827.02281	49608.21033	45125.10002	51583.10262	41486.56796	32997.90127	39574.95025	34947.93003	41278.29828	36211.28793
Q9JMH9	Myo18a	Unconventional myosin-XVIIIa	392.202	9.22	232755	11	44995.70565	42401.88633	35262.05595	50531.73554	42495.60325	36007.18078	34942.96183	38465.64636	39597.24074	41831.69401	40476.39369	39962.55139	36255.10611	45702.81556	43845.91521
Q9QUJ7	Acsl4	Long-chain-fatty-acid--CoA ligase 4	768.031	38.4	79077	19	183965.3149	210348.8877	192080.5236	207226.6352	200552.2458	196540.8372	216624.9221	231896.9133	216643.2974	179472.602	169709.0822	194208.712	179563.2756	178978.3146	195352.5869
Q9QUN7	Tlr2	Toll-like receptor 2	67.488	2.93	89449	2	7652.299103	6749.405423	6581.194543	8945.061154	6631.009997	9371.926655	10002.57427	8113.402372	9592.810544	10370.7229	6550.601771	7366.861402	8246.509086	6882.727042	7665.579651
Q9QUR6	Prep	Prolyl endopeptidase	678.986	36.76	80752	18	139761.283	153826.9844	153106.6565	146971.448	151608.1112	155670.5951	163716.1684	131158.0266	128599.2213	148131.4942	131445.3658	149629.5453	152702.1539	149694.2342	155244.2541
Q9QVP9	Ptk2b	Protein-tyrosine kinase 2-beta	535.731	23.09	115794	18	128360.191	133971.6048	135913.8099	133656.2528	140048.6216	140946.6637	145535.8434	134406.0183	139382.7741	122648.1385	129248.4676	139419.3098	141860.3565	133278.0192	128980.2561
Q9QWR8	Naga	Alpha-N-acetylgalactosaminidase	268.096	27.47	47235	10	95748.93291	92650.34613	79697.34025	89297.50455	63406.75091	52250.00873	56281.7888	50102.40175	55548.85207	52704.48971	72471.73908	70915.03603	81944.72336	72696.22065	76343.51179
Q9QWT9	Kifc1	Kinesin-like protein KIFC1	85.723	8.46	74153	4	22302.1298	24088.63459	26409.34987	26189.2099	26047.84584	13420.05493	18702.38762	19826.90247	23665.53678	23424.32125	21900.64372	20550.63591	22941.36203	20120.32707	21881.58328
Q9QXB9	Drg2	Developmentally-regulated GTP-binding protein 2	78.998	8.24	40718	2	8938.543682	7477.008617	6297.534484	8814.982255	9165.96758	8049.304786	8390.135223	9359.607671	10027.23549	6309.46411	5919.472221	7230.651913	7802.990869	9568.877707	8316.939718
Q9QXE7	Tbl1x	F-box-like/WD repeat-containing protein TBL1X	104.011	9.68	56802	4	15012.10361	22285.85677	21388.71257	20625.59427	23876.79112	22247.98321	22937.27211	21255.65958	23924.13032	19696.16411	19154.84886	20812.29724	21964.5103	24560.34565	22112.3253

Q9QXG4	Acss2	Acetyl-coenzyme A synthetase, cytoplasmic	116.082	7.42	78862	3	15682.11703	17344.55167	14667.98258	13599.34122	13164.46525	11339.66503	14687.59615	15717.00871	13824.76305	10815.88687	5609.334998	8957.332565	10990.20254	8141.504142	13500.27041
Q9QXK3	Copg2	Coatomer subunit gamma-2	433.918	19.63	97681	12	130047.5933	140072.9921	125815.3617	141278.8064	128934.1162	130818.9082	167900.1401	141382.6086	147967.1868	124757.9895	129451.5914	130973.8075	133809.3875	135091.4331	136357.8673
Q9QXK7	Cpsf3	Cleavage and polyadenylation specificity factor subunit 3	97.847	13.01	77505	5	27076.94573	23840.85014	19091.76081	21959.09796	19969.30263	20143.1764	22626.03808	21787.51063	25972.9018	23638.18518	21160.58623	22129.13351	17318.41329	22750.28641	23622.23429
Q9QXS1	Plec	Plectin	5678.135	44	534188	171	3204091.02	3104901.628	2971521.163	2964316.996	2970063.578	2841388.787	2957741.696	2962009.824	3023665.162	2980586.416	3129732.522	2974072.647	3068865.731	3240870.062	3055979.782
Q9QXT0	Cnpy2	Protein canopy homolog 2	95.44	14.29	20767	2	19595.50917	7337.681506	19323.20486	21397.62465	18289.5499	7454.229835	16475.7509	16816.38485	10434.86855	11273.25547	8012.497159	12033.63866	18333.93985	16014.29457	6521.479514
Q9QXW9	Slc7a8	Large neutral amino acids transporter small subunit 2	148.128	14.88	57873	6	56115.60508	58669.1265	48472.17769	49983.92259	48191.98274	46826.19101	48736.06847	44417.98229	48343.29284	50403.90695	53656.5236	48113.93149	60119.24329	52534.24078	54714.15803
Q9QXX4	Slc25a13	Calcium-binding mitochondrial carrier protein Aralar2	855.463	64.64	74467	27	477578.491	482304.7455	496813.8462	499673.6508	488879.9369	502307.2793	474889.9731	467923.7827	491030.8171	482822.3162	461160.6329	455090.7884	474658.6044	493846.6408	470510.2924
Q9QXZ0	Macf1	Microtubule-actin cross-linking factor 1	698.777	4.58	831878	24	57608.80422	54728.13993	42435.57689	48790.76659	53412.76124	39596.75192	51507.41799	40609.55099	57613.79414	58792.97399	55356.50301	46206.54352	56405.0413	57624.8768	50304.31921
Q9QY06	Myo9b	Unconventional myosin-IXb	209.275	4.4	238834	6	21777.65362	16333.16364	11261.51469	17903.8996	17644.38721	11997.18835	21335.79542	17101.66622	20078.98515	18927.04986	19977.66782	20710.12796	20969.52185	23004.31916	19401.30213
Q9QY17	Pacsin2	Protein kinase C and casein kinase substrate in neurons 2 protein	45.468	4.1	55978	2	22853.19349	17490.00528	21009.77802	18734.82452	21303.11469	17345.93709	18664.96557	8304.9161	8906.397922	8985.514236	21455.46744	18424.71227	18706.06404	19891.19523	21007.94115
Q9QY76	Vapb	Vesicle-associated membrane protein-associated protein B	278.886	37.86	26946	8	119339.1094	113438.5194	108877.4664	100931.343	103975.537	91673.18817	113038.1418	105031.0147	103272.2853	104492.2642	110331.3562	108652.6192	102583.5407	150322.5512	106031.0836
Q9QYB1	Clic4	Chloride intracellular channel protein 4	894.001	77.08	28729	20	536635.7143	552719.3661	509766.9624	528163.831	534202.6664	507396.2673	485914.1474	548282.2012	525107.2248	501996.6187	561493.0318	566526.4916	575114.4134	552917.4439	511704.8838
Q9QYF1	Rdh11	Retinol dehydrogenase 11	165.498	15.19	35148	4	67659.60088	69628.18231	76878.81944	62066.17297	74371.03256	78232.63393	81583.1454	75073.65885	86115.12049	66538.76258	77009.17213	67449.43624	69265.72539	68814.44526	75073.82719
Q9QYI3	Dnajc7	DnaJ homolog subfamily C member 7	41.484	4.86	56476	2	6505.687805	7231.690923	7956.973523	9859.757225	7450.636037	7165.083677	7877.164976	8209.105426	7948.154348	6400.247378	6813.480352	8185.863513	7041.799756	7842.978139	8029.887539
Q9QYJ0	Dnaja2	DnaJ homolog subfamily A member 2	275.12	37.86	45746	9	46050.72904	55714.57766	83814.53539	79398.79306	45445.63871	46182.78671	95159.53324	98471.16789	90879.07431	58576.66081	42792.73295	78818.5262	36419.59251	35852.47993	39066.20175
Q9QYJ3	Dnajb1	DnaJ homolog subfamily B member 1	54.184	9.41	38167	3	22173.14703	18066.14225	20617.803	15218.79878	23677.90009	20175.02583	20904.57744	19941.1091	21751.40293	18919.91024	21775.69688	21654.38438	19844.53718	21130.40125	22545.28711
Q9QYL8	Lypla2	Acyl-protein thioesterase 2	65.824	31.17	24807	3	35067.33742	25482.8113	26120.49563	26073.13583	20643.67686	18785.15732	12993.54912	12938.31866	12651.41634	11530.96865	26080.9223	18624.6819	17652.31151	3857.338751	16937.69736
Q9QYR9	Acot2	Acyl-coenzyme A thioesterase 2, mitochondrial	162.512	13.25	49657	4	39719.66733	39950.18141	32971.5638	34829.34791	32206.55962	32001.95313	26260.03039	26193.08143	31462.59895	33076.49499	33151.05282	28333.83342	34214.81046	33412.22657	35102.97718
Q9QZ08	Nagk	N-acetyl-D-glucosamine kinase	284.72	37.32	37268	9	113205.307	94353.51558	105634.1471	120382.0616	93172.65449	75165.63045	105334.2005	83361.30206	99287.33534	101377.0921	99650.85903	89987.24463	92798.51046	112088.4047	100377.3939
Q9QZ88	Vps29	Vacuolar protein sorting-associated protein 29	189.181	40.66	20496	6	69708.65762	77560.80852	74033.8991	74835.09598	71382.25502	56520.4496	63415.05774	59277.28612	62573.22092	62793.93189	73885.5435	72088.62652	66936.18407	63233.34199	62100.45131
Q9QZB7	Actr10	Actin-related protein 10	143.584	15.59	46208	4	15298.2279	12148.59544	18653.29655	16239.60108	17531.33299	13958.11381	17177.40663	14339.60509	15562.08221	14507.64494	13377.43822	18099.99046	16799.68555	15573.45775	14927.99798
Q9QZD8	Slc25a10	Mitochondrial dicarboxylate carrier	192.678	28.22	31715	5	36743.42034	36926.30789	32178.15614	32503.36343	38215.26045	39804.18397	32416.23867	31637.04989	37480.22229	35210.84848	45956.83508	26790.99612	32770.42665	43424.43819	44181.50983
Q9QZD9	Eif3i	Eukaryotic translation initiation factor 3 subunit I	303.528	40.62	36461	10	79138.37419	100582.8362	102263.1096	109151.5958	95687.33542	96883.56891	106457.0966	137974.0489	99893.69781	96685.60234	88093.14102	92199.84528	88998.72687	91168.15406	95850.95898
Q9QZE5	Copg1	Coatomer subunit gamma-1	519.389	27.92	97513	16	191334.9762	208751.9475	196101.64	201000.0245	193036.3482	196495.822	242135.2395	209408.0096	226533.1146	193136.13	203531.5525	202992.9611	203371.4131	208819.2316	207689.7917
Q9QZE7	Tsnax	Translin-associated protein X	164.886	34.83	32926	7	29881.12246	30132.61364	35083.46921	32391.62041	28718.62959	26689.27399	32385.04025	25889.8936	36311.27242	31632.17048	34908.97048	36454.3988	33697.63173	29887.79443	701169.3869
Q9QZL0	Ripk3	Receptor-interacting serine/threonine-protein kinase 3	225.918	25.72	53322	8	43545.43564	55541.35296	62719.16098	52696.85427	51739.68526	57852.50606	64749.53264	67197.89201	49881.87352	52772.86556	48315.00367	45844.78246	49256.72388	52851.5057	50313.70057
Q9QZM0	Ubqln2	Ubiquilin-2	60.757	5.17	67351	2	2313.757338	2402.154537	2663.195897	0.009171104	0.008265859	0.014539799	0.012348474	0.01388636	0.012083387	0.013320187	0.008046421	0.009057159	0.00981159	0.00945634	0.010360417
Q9QZN4	Fbxo6	F-box only protein 6	44.32	11.86	34493	2	2935.286505	1167.970676	2167.634098	2367.663978	1347.321823	1598.306062	2300.476331	2599.297794	1995.91971	1954.629003	2374.86994	1774.856679	2119.182354	1655.78393	2142.866524
Q9QZQ8	H2afy	Core histone macro-H2A.1	155.609	18.01	39735	5	77007.6464	75849.61534	70909.42048	73313.52276	74677.92737	78687.96229	79555.29751	65170.71959	70503.10066	77059.49242	77447.05031	77260.05172	79411.25304	80209.00351	86095.65283
Q9R002	Ifi202	Interferon-activable protein 202	33.455	2.7	50466	2	6090.589968	6669.685765	6205.69664	8290.540122	6394.358951	7796.957407	5464.188983	7251.730365	8668.213946	8239.408076	6902.794135	6611.635823	7267.213682	10027.74956	6091.868003
Q9R059	Fhl3	Four and a half LIM domains protein 3	68.107	9.69	31795	2	12216.36093	13993.85607	14383.99148	13820.16076	14657.33414	12713.14537	3155.66762	14353.00196	1868.080498	12271.29046	11972.47681	14322.80741	17208.18375	14551.64588	14119.99816
Q9R062	Gyg1	Glycogenin-1	32.38	17.72	37402	3	27806.42218	27913.82545	28250.90021	26661.41168	28830.63817	28521.09425	27247.39895	26058.97217	27371.61727	25396.20176	29867.33462	28702.56623	27405.72632	29563.55782	30169.26907
Q9R0C8	Vav3	Guanine nucleotide exchange factor VAV3	179.81	17.47	97968	9	69293.23591	78222.79214	69229.0989	67508.59935	69649.82191	72095.90076	69131.74678	65474.41428	75815.10858	68822.86161	64458.67062	62212.4372	65271.08853	72746.52365	74499.0779
Q9R0E1	Plod3	Multifunctional procollagen lysine hydroxylase and glycosyltransferase LH3	256.913	9.99	84922	5	25698.2037	22941.79803	24527.35384	25754.99941	18931.84434	18797.49979	24906.35207	21932.68036	23474.72802	23673.34548	21854.02879	20515.05938	23464.97226	20505.75801	21623.89511
Q9R0E2	Plod1	Procollagen-lysine,2-oxoglutarate 5-dioxygenase 1	73.614	8.65	83595	4	13493.64551	16086.60809	8821.733474	15013.83493	14708.36445	15100.19055	16612.85601	9656.015486	13812.27487	12210.27647	13076.95972	9940.910645	14966.05756	14298.44175	14902.08658
Q9R0N0	Galk1	Galactokinase	333.628	33.93	42295	9	189188.824	187252.8467	153929.7779	157739.3416	178744.0003	201388.8134	200877.123	212474.9232	215150.5641	177439.5818	180530.4722	179368.3932	175613.6985	194141.9713	173431.6651
Q9R0P3	Esd	S-formylglutathione hydrolase	370.137	64.89	31320	11	200833.0865	211877.0856	189485.8377	177121.2219	177750.6094	210007.5849	215872.0603	239967.3878	212246.3463	192745.7953	174555.007	180801.5619	185161.7101	184290.1865	198485.7426
Q9R0P5	Dstn	Destrin	436.859	71.52	18522	11	535084.4907	418301.6619	310498.5484	316225.5321	360596.848	327208.8369	263101.7878	326634.9626	340386.3385	276406.8389	520313.8959	437129.5718	409383.9003	419688.2478	379164.4052
Q9R0Q8	Clec4e	C-type lectin domain family 4 member E	43.575	12.15	24431	2	3453.607616	0.007562416	5299.737919	3763.06661	1932.177342	21983.50062	10088.36758	18583.85805	16542.06151	12638.85016	6374.77794	4170.323509	3513.606187	6290.35356	3124.0072
Q9R0U0	Srsf10	Serine/arginine-rich splicing factor 10	137.952	14.89	31301	4	23039.44389	24453.39069	18577.69033	21218.95321	19243.67593	17433.95922	20509.24876	20395.39267	17101.14924	15272.05051	22327.01753	20541.33647	32110.83759	19489.6625	11117.2386
Q9R0X4	Acot9	Acyl-coenzyme A thioesterase 9, mitochondrial	118.054	18.22	50560	6	26262.63519	31974.41919	36251.91235	32808.83914	27411.62164	38209.64415	41482.41735	37771.05065	36578.54486	37181.51019	33973.7028	40980.86141	41816.53128	39942.43901	37569.13708
Q9R112	Sqor	Sulfide:quinone oxidoreductase, mitochondrial	328.27	24.44	50282	8	93534.46102	79702.45439	60700.69526	87352.25844	72121.37159	97781.87407	108620.327	106500.4484	107213.4028	69486.2602	72188.18321	55292.43227	74311.22771	68263.87352	61660.27347
Q9R190	Mta2	Metastasis-associated protein MTA2	229.831	14.97	75030	8	91220.24482	92632.2039	89814.77977	89056.52463	87328.36158	86999.60228	94781.78724	91830.5719	88254.70164	81800.44665	88192.28097	89914.74207	88269.68648	87370.19493	91122.67203
Q9R1C7	Prpf40a	Pre-mRNA-processing factor 40 homolog A	121.702	6.09	108481	5	28900.99574	47817.78974	14053.00474	54857.30491	24386.61484	17201.74408	29648.66161	34072.13065	33608.13117	26397.37698	25014.56032	38663.60981	29551.86204	31543.36072	29510.66774
Q9R1J0	Nsdhl	Sterol-4-alpha-carboxylate 3-dehydrogenase, decarboxylating	90.186	13.81	40686	4	25715.38029	31644.31974	33826.34847	36389.87997	31766.38196	35696.7597	38723.24034	32763.48013	37610.78027	35770.85578	30614.18731	39035.5964	36910.92506	32255.95265	26826.73447
Q9R1P1	Psmb3	Proteasome subunit beta type-3	292.76	43.9	22965	8	106387.9866	112469.8042	121021.7451	126546.1346	101140.3722	102171.7812	112042.2991	98718.87971	124447.285	102288.0397	105144.0218	134405.3388	120308.5708	127825.9571	109931.2107
Q9R1P3	Psmb2	Proteasome subunit beta type-2	225.423	55.22	22906	7	221356.0162	217703.3673	185007.5415	227967.2879	203153.462	166154.8228	168880.6583	182506.4791	204059.8033	157045.5498	185712.7213	213548.8268	210077.2622	211018.5115	180549.2506

Q9R1P4	Psma1	Proteasome subunit alpha type-1	404.874	72.24	29547	15	585719.8223	579801.4052	534847.8862	534904.849	566035.2496	492976.0432	493599.3932	510146.4809	487805.3745	484124.1779	597387.0854	540163.8176	557857.9064	558688.9128	543309.5834
Q9R1Q6	Tmem176b	Transmembrane protein 176B	40.262	10.27	28367	2	25442.97959	24338.20187	36452.89971	20041.37662	15093.6382	23938.88555	38504.30806	42079.84568	23784.40408	27973.71121	25998.06227	11723.51133	25077.2054	31643.6523	24126.13388
Q9R1T2	Sae1	SUMO-activating enzyme subunit 1	585.544	59.71	38620	16	219299.1363	228124.9509	232350.6305	238780.8995	248300.9718	181859.4783	194478.2919	200926.4018	205296.8038	192365.2242	226353.4873	232004.4376	220380.1775	234566.3857	215102.0482
Q9R1T4	Sept6	Septin-6	90.798	11.52	49620	4	46572.42678	54701.7471	55624.15391	49398.27884	49128.06234	53033.25682	53172.66529	51041.5536	49893.22284	53362.32046	53716.65048	53819.74268	47598.67307	54774.07883	46474.73625
Q9R233	Tapbp	Tapasin	125.12	16.56	49736	6	114049.148	96370.46479	117525.2623	105519.4902	99296.9203	175493.1108	141491.6193	148811.686	153991.9272	137235.5014	132206.8227	114484.2796	125841.1597	129920.8579	119027.7912
Q9WTI7	Myo1c	Unconventional myosin-Ic	1374.976	35.84	121944	31	364137.9191	345903.7826	351800.0819	369291.6288	352050.9748	345314.6093	368776.7444	360182.5742	331628.1063	373121.2795	353663.379	340439.1053	350782.2549	333706.5556	370761.6929
Q9WTK5	Nfkb2	Nuclear factor NF-kappa-B p100 subunit	69.018	2.89	96832	2	7230.348235	8974.680767	9724.162827	8491.145404	7064.710812	12677.58735	14513.73118	13234.47562	14828.39104	12786.71262	9770.070835	8243.811219	8493.349071	9966.992583	9974.08059
Q9WTM5	Ruvbl2	RuvB-like 2	332.26	30.24	51113	12	177540.0699	192068.8804	165681.8486	178923.1053	201608.358	181638.0226	187625.1728	179276.8591	188426.1119	185440.6451	181075.7299	168327.5347	165672.9289	182341.9157	181448.1825
Q9WTP6	Ak2	Adenylate kinase 2, mitochondrial	334.009	47.28	26469	8	210552.7156	192785.9638	173446.7533	177230.9083	181871.4046	159255.0628	177316.2055	166646.8679	168155.3121	153792.0275	172231.7317	173278.178	164407.4104	165799.8085	170770.4678
Q9WTP7	Ak3	GTP:AMP phosphotransferase AK3, mitochondrial	230.256	42.73	25426	6	39277.97071	36013.71716	26729.06616	30179.04351	31388.53509	26296.62671	23875.48756	30968.37308	24162.39357	32248.56545	33486.08366	29753.94847	32766.49465	26338.77817	37023.62229
Q9WTR1	Trpv2	Transient receptor potential cation channel subfamily V member 2	299.97	9.66	85965	5	26026.13628	29525.13046	15265.36349	25676.22192	25340.54869	23370.27943	28943.78292	26199.21052	28178.90835	23061.28955	18222.98281	26616.34012	30343.32378	28066.43959	33909.14637
Q9WTX6	Cul1	Cullin-1	217.197	8.12	89692	5	16812.38569	22122.21933	8329.331964	23033.04566	5787.993105	4533.429054	18722.72555	18437.36217	9877.599359	9135.375463	9542.752405	19960.89935	10227.09508	9214.324074	9551.453967
Q9WU28	Pfdn5	Prefoldin subunit 5	128.072	21.43	17356	2	12882.21079	9668.658133	9719.138595	9086.345444	9440.790858	9860.006856	8398.027441	7896.29782	8122.340901	7305.617152	9523.096004	8913.21573	7436.118342	7226.129286	7729.553307
Q9WU78	Pdcd6ip	Programmed cell death 6-interacting protein	1224.259	53.39	96024	35	690145.7785	697809.2723	652313.1694	669418.5999	644850.8759	524275.1133	584867.8049	575161.6385	569634.1672	543271.722	632398.35	640817.4434	622081.3182	627895.6637	658879.658
Q9WU81	Slc37a2	Glucose-6-phosphate exchanger SLC37A2	189.923	12.38	55073	4	144053.719	143711.8602	131288.2431	129623.7735	117957.5349	90748.04981	103616.9105	92056.37105	97515.66311	110851.6411	127302.2636	128759.3773	139410.1866	129921.5387	135314.0553
Q9WUA2	Farsb	Phenylalanine--tRNA ligase beta subunit	390.165	25.81	65697	13	246351.7362	270022.1707	261095.4021	263642.6242	264676.5476	249485.5248	280619.7777	273780.5899	255271.2995	256600.2804	264064.7124	270159.1709	257010.5917	263359.2377	266453.8056
Q9WUA3	Pfkp	ATP-dependent 6-phosphofructokinase, platelet type	1753.233	65.31	85455	44	1310137.888	1409160.101	1314621.481	1351441.956	1326130.772	1471971.737	1440983.958	1431499.936	1463862.025	1444233.607	1414355.838	1405264.525	1402977.499	1463378.701	1364086.033
Q9WUB3	Pygm	Glycogen phosphorylase, muscle form	193.969	9.26	97286	5	75116.23915	71159.59418	66385.51946	63793.93094	66279.00463	55134.71948	61030.63531	64176.75481	65518.05106	65967.87269	67613.88145	61130.57762	72175.90191	74899.71327	65230.01201
Q9WUL7	Arl3	ADP-ribosylation factor-like protein 3	124.646	32.42	20487	4	35940.057	36684.98365	32928.00838	34936.96622	31840.03903	31589.8625	29140.04648	32005.62859	33650.07562	31303.65471	37185.36329	35078.93101	32918.00768	32549.43724	31492.93144
Q9WUM3	Coro1b	Coronin-1B	170.612	30.17	53912	7	127743.1582	114185.9356	101870.9111	99212.87076	109173.7786	92641.26982	88602.78369	88641.47917	98994.65758	80096.55498	103553.1076	105825.8707	105622.5335	105706.4913	99308.72731
Q9WUM4	Coro1c	Coronin-1C	654.857	39.87	53121	16	455333.888	421010.1259	409205.5575	438327.8084	400118.3332	359136.6661	417711.5492	367556.8143	383985.3009	367722.9008	403225.1796	405382.6339	400407.041	425469.0973	403345.6177
Q9WUM5	Suclg1	Succinate--CoA ligase [ADP/GDP-forming] subunit alpha, mitochondrial	257.113	25.72	36155	7	111191.023	115820.3779	110414.8777	112917.1684	113397.3267	107335.8818	96317.60401	98143.53312	99810.84203	99436.90027	103952.2423	96258.75694	95557.38244	88494.0998	101153.1405
Q9WUN2	Tbk1	Serine/threonine-protein kinase TBK1	31.136	5.62	83425	3	16584.07609	18742.37157	17095.98576	15656.50804	16659.26111	17118.58915	16857.77719	17866.26307	17567.05443	15118.70019	16203.44382	12689.38606	18577.50154	18516.71627	15177.82907
Q9WUP7	Uchl5	Ubiquitin carboxyl-terminal hydrolase isozyme L5	303.691	52.89	37617	14	152318.1751	179440.3721	159392.6739	168056.0629	158087.0071	166656.097	169877.7359	167029.3399	168905.4004	163416.0224	163461.8571	163992.108	153604.0254	159853.5865	161451.5527
Q9WUR2	Eci2	Enoyl-CoA delta isomerase 2, mitochondrial	34.15	4.86	43268	2	10634.16209	9772.734934	10683.46743	11482.94971	14309.25572	12381.70243	10858.95719	11064.15136	14396.02934	13917.46822	10859.38021	10875.84046	12351.30067	13327.16338	16285.12191
Q9WUT3	Rps6ka2	Ribosomal protein S6 kinase alpha-2	109.358	6.96	83157	5	36831.26527	35823.11782	31669.55149	34055.72404	34882.26362	33494.97243	33954.45104	36470.3223	35160.95816	34200.34913	31091.3461	24541.78643	37834.6225	28509.36508	38567.86311
Q9WUU7	Ctsz	Cathepsin Z	406.55	49.67	33996	11	1261812.972	1262562.612	1102909.948	1266830.527	1247926.357	1000741.723	1010848.769	1017789.959	868577.9324	1012053.003	1198487.747	1250978.205	1175343.191	1215839.04	1158755.422
Q9WV32	Arpc1b	Actin-related protein 2/3 complex subunit 1B	836.032	70.7	41064	22	1893889.304	1737714.757	1808893.126	1777751.39	1763437.56	1589770.982	1780959.183	1739684.69	1685662.451	1563053.403	1609996.258	1691531.047	1667150.876	1809515.53	1748550.403
Q9WV54	Asah1	Acid ceramidase	610.897	45.18	44670	17	351943.1996	320076.3785	265283.7599	286804.0263	269999.5459	208872.4191	209983.2459	204007.3545	230132.8764	235988.5699	289281.616	230818.8192	289832.4801	279092.6766	270488.3797
Q9WV55	Vapa	Vesicle-associated membrane protein-associated protein A	257.742	42.57	27855	7	113335.2173	102930.9814	98185.4852	112619.9696	102308.3794	105105.6583	101430.8602	122582.2969	110442.2408	105959.6892	110275.7957	111987.9635	111965.9915	104779.5998	92646.83436
Q9WV80	Snx1	Sorting nexin-1	300.524	20.88	58952	9	70033.91869	76431.82304	63484.02937	68735.22987	74625.65408	73914.80059	70639.02622	70109.24366	70550.28024	65649.23384	64667.05683	72555.26043	71438.35486	68015.71555	76070.39049
Q9WVA3	Bub3	Mitotic checkpoint protein BUB3	206.598	23.62	36955	6	78797.85327	81044.59412	83952.21046	84155.28508	92302.97041	76880.30173	76641.89115	81326.91567	76077.14891	76187.51969	76993.68277	77832.60005	74344.16522	77678.8842	85993.82548
Q9WVA4	Tagln2	Transgelin-2	924.244	84.42	22395	24	1146786.56	1119708.58	957532.2966	990102.9667	1017330.119	858507.9383	908464.7056	772943.5019	928466.7745	949925.946	1033027.843	1034081.698	1103513.952	1036317.316	967108.518
Q9WVJ2	Psmd13	26S proteasome non-ATPase regulatory subunit 13	718.896	59.31	42809	18	409674.1731	392482.6067	433081.4225	411740.4855	382934.0092	430049.0999	483360.6806	495696.6129	467172.653	427553.5839	406137.3724	459229.1495	402434.9397	437810.7553	358972.9892
Q9WVQ5	Apip	Methylthioribulose-1-phosphate dehydratase	113.666	9.13	26949	2	15659.99539	18947.80459	16886.63919	16133.59229	17914.34603	2786.560701	13570.24138	12105.81752	12950.00287	11856.92941	15368.08854	14059.77617	15132.11014	13009.15023	15248.94945
Q9Z0F8	Adam17	Disintegrin and metalloproteinase domain-containing protein 17	43.267	2.78	93056	2	7415.592474	9583.354084	19118.2697	15718.26855	15364.79661	18106.62668	23536.29659	19422.80941	9076.837116	7966.141112	8189.687393	7802.524357	17280.3713	17230.72028	17189.62255
Q9Z0J0	Npc2	NPC intracellular cholesterol transporter 2	140.289	41.61	16442	5	148860.8603	124912.9274	110178.2044	109680.7134	108067.9885	87267.87612	88934.98851	76808.34288	83077.53223	70873.33788	88793.34861	91576.18739	89583.30141	97650.27628	103709.4075
Q9Z0M5	Lipa	Lysosomal acid lipase/cholesteryl ester hydrolase	246.757	32.75	45325	11	138840.9042	168603.8993	127271.349	145809.7535	114750.1254	213546.0486	251590.4662	268659.9115	270924.9889	166806.2631	167484.1664	215060.1769	152931.3526	153549.0449	170640.7346
Q9Z0P5	Twf2	Twinfilin-2	458.829	52.44	39471	11	105772.1384	104010.5692	104623.2904	97996.58065	103798.6641	78118.62737	89882.15352	91012.88751	91020.89098	86924.92236	96943.14185	99161.7758	103876.0312	99905.51871	104971.2027
Q9Z0R9	Fads2	Fatty acid desaturase 2	26.472	4.95	52387	2	2161.440816	1865.04591	1910.255647	0.009171104	1875.532692	1665.574226	1739.2083	1396.767527	2395.723276	1709.509458	1691.097687	2191.794636	2567.891245	1667.440665	2295.228727
Q9Z0S1	Bpnt1	3'(2'),5'-bisphosphate nucleotidase 1	96.983	13.64	33196	3	20836.58348	24326.02827	23152.95442	23725.08567	23087.12882	18008.83744	23126.19142	19542.45775	19436.07194	15762.59969	16900.61388	19920.46366	18144.23153	21701.75093	20724.25869
Q9Z0W3	Nup160	Nuclear pore complex protein Nup160	206.661	8.2	158232	6	13971.96475	12968.71957	11425.80327	14025.23811	11293.83835	9389.44624	11768.41453	12571.14159	14668.49008	12593.89453	11360.37048	12248.56392	12028.80106	11041.36279	12273.14899
Q9Z0X1	Aifm1	Apoptosis-inducing factor 1, mitochondrial	474.576	27.45	66765	13	70233.5554	76193.38007	81231.347	87465.98012	59972.13773	79062.24581	73302.19076	95343.66811	82182.65472	87162.40131	72791.84984	80431.85084	76696.75397	80158.61068	72486.68611
Q9Z110	Aldh18a1	Delta-1-pyrroline-5-carboxylate synthase	631.966	28.43	87266	17	105407.4239	104913.5897	93911.96604	110551.3538	97810.99164	130241.7364	131075.0887	128456.6575	134633.4956	124223.5836	116754.0657	104182.9473	95189.57536	114741.8442	106550.8761
Q9Z1B5	Mad2l1	Mitotic spindle assembly checkpoint protein MAD2A	68.328	23.41	23598	4	34840.44687	41979.61994	40269.7027	17288.26175	49861.80197	41029.47104	40996.31658	43675.3016	45341.81881	46110.178	35942.77464	27580.53588	45831.16416	34092.36907	40514.57504
Q9Z1F9	Uba2	SUMO-activating enzyme subunit 2	577.522	38.09	70569	16	157157.284	178355.1806	142075.6658	171486.1657	146551.8026	125772.1148	126785.3029	130795.9953	132881.924	116187.395	141489.7371	154313.1564	132383.9303	143059.2786	122319.0883

Q9Z1G3	Atp6v1c1	V-type proton ATPase subunit C 1	536.893	54.71	43888	16	231747.6547	231552.888	197843.971	221378.6203	218160.476	199840.5284	213112.8185	228607.1098	216870.2234	197551.399	237121.4008	256881.6831	224403.7144	222878.5769	237288.6318
Q9Z1Q5	Clic1	Chloride intracellular channel protein 1	697.267	82.99	27013	16	1466844.442	1411935.205	1368937.565	1446912.411	1406327.458	1380380.422	1336528.413	1298901.311	1347960.787	1278786.229	1370944.304	1391726.172	1423905.857	1407922.64	1421554.767
Q9Z1Q9	Vars	Valine--tRNA ligase	1585.913	49.56	140215	42	760587.9538	788702.8541	795669.3931	799443.4992	766977.2885	719910.5572	788623.0608	795480.8968	759931.8991	675189.5784	739181.6933	831733.4523	759587.0261	727549.067	728223.8329
Q9Z1R2	Bag6	Large proline-rich protein BAG6	133.391	4.68	121037	3	26210.75581	23750.36206	25905.87106	25830.22997	25259.82431	24387.20209	29126.10197	29939.94929	28144.9933	26435.02815	25314.38404	27916.33912	24957.10663	23943.97671	26406.35538
Q9Z1T1	Ap3b1	AP-3 complex subunit beta-1	129.11	7.15	122740	6	24563.0702	35348.56111	23257.80839	26384.11419	25014.37996	21909.49123	21802.82088	29350.03938	24854.29434	23611.56478	29573.41495	23077.20299	24424.83767	25932.80332	24014.9407
Q9Z1W8	Atp12a	Potassium-transporting ATPase alpha chain 2	91.022	2.71	114727	5	123549.3314	128740.8332	120077.5054	112112.8443	128795.2911	134062.0396	112799.0506	116756.8025	130902.6653	119130.0773	127587.7748	126588.8109	139749.8835	129460.8637	137541.5864
Q9Z1X4	Ilf3	Interleukin enhancer-binding factor 3	135.001	6.57	96021	5	35659.01248	42492.93403	42159.23296	37478.91872	38947.11134	31455.88188	36368.3678	39248.09758	37132.72221	37003.18619	38070.32878	39058.70122	30490.62027	36511.62699	37382.51231
Q9Z1Z0	Uso1	General vesicular transport factor p115	76.005	5.21	106983	4	31116.17304	28898.1041	28935.53106	20147.81645	31507.13776	23605.10991	33999.66498	23591.62645	30069.36631	26492.74785	28229.45749	28994.76831	30818.56777	31112.36677	30868.52467
Q9Z204	Hnrnpc	Heterogeneous nuclear ribonucleoproteins C1/C2	536.73	32.91	34385	13	270156.3485	277721.5266	303275.1004	304417.3877	246393.4926	290279.2876	300666.2659	316757.1785	276586.3215	260095.3909	265135.9324	273016.2337	310590.3513	328553.607	301388.1152
Q9Z277	Baz1b	Tyrosine-protein kinase BAZ1B	36.765	1.89	170651	2	14092.45279	16806.10767	13652.22116	18488.29952	12777.96815	10876.85231	12545.68157	11050.42816	11340.09699	11442.39434	13911.39066	15567.18979	13253.59903	12953.7148	10144.2278
Q9Z2I0	Letm1	Mitochondrial proton/calcium exchanger protein	193.315	13.69	82989	6	25228.59512	16992.29056	16040.80692	14474.11089	14653.35826	20148.51978	15000.56205	14342.24093	19727.69693	18416.88503	16403.25455	16498.99941	14662.73593	16937.05365	21648.24209
Q9Z2I8	Suclg2	Succinate--CoA ligase [GDP-forming] subunit beta, mitochondrial	666.326	45.96	46840	16	270662.9262	263768.8394	274797.5384	265124.6929	279832.2975	280132.5431	256868.9696	252201.4649	273159.8355	268497.4718	281135.9023	255949.8825	284164.0498	288174.5641	268723.7107
Q9Z2I9	Sucla2	Succinate--CoA ligase [ADP-forming] subunit beta, mitochondrial	139.405	13.17	50114	5	95377.66194	95227.31491	99481.71548	91313.11136	97710.12336	99400.56086	98038.42562	102029.2767	101471.8486	91476.72327	72903.09965	98035.75351	97306.23952	104578.681	100482.3139
Q9Z2L0	Vdac1	Voltage-dependent anion-selective channel protein 1	927.384	69.61	30756	16	582097.9972	588944.3053	628100.5391	587331.8664	597040.6849	752395.0281	701629.7402	749488.8297	727244.9842	668743.3005	563672.0026	606288.2186	606241.4085	607004.7561	592585.8385
Q9Z2L7	Crlf3	Cytokine receptor-like factor 3	120.444	20.81	49559	4	12060.71249	12930.60784	15233.03096	14266.27878	12511.37767	12413.55913	12812.28124	13628.40304	13144.7765	9535.932598	12442.18864	9490.50431	9457.137895	10428.51042	11696.25121
Q9Z2N8	Actl6a	Actin-like protein 6A	214.878	23.08	47448	6	61167.21663	72677.32564	87867.31034	65320.69501	64764.30665	75315.21591	87233.86272	82335.97493	75028.40155	79228.16538	80904.7261	84241.60911	68907.96047	79848.67914	82310.52641
Q9Z2U0	Psma7	Proteasome subunit alpha type-7	505.459	55.24	27855	11	344010.7748	367159.8879	350295.1565	370689.4884	356109.214	326438.8091	365718.6942	366362.4485	343149.5432	322172.6834	348779.0084	341083.1418	369822.8623	348911.1771	361970.2578
Q9Z2U1	Psma5	Proteasome subunit alpha type-5	463.579	49.38	26411	10	240078.7112	262830.4949	238075.2394	267316.3299	244283.4833	209176.4463	235651.0311	260757.9064	248900.2129	226611.558	240004.8357	276362.6187	276595.6442	253573.3447	262621.5703
Q9Z2W0	Dnpep	Aspartyl aminopeptidase	583.343	48.2	52207	13	164357.5979	176846.2518	145878.4345	158189.4778	146651.803	133409.5952	139238.3809	139647.1886	146890.0132	134500.7072	164843.4598	157738.1394	157482.7138	149179.4595	151149.0301
Q9Z2Y8	Plpbp	Pyridoxal phosphate homeostasis protein	53.737	10.58	30049	2	71406.86938	63483.07675	79575.92714	58337.39483	62262.7271	59697.61024	69016.09097	69232.8894	68540.70425	81162.23654	61365.204	67698.01451	57778.87488	62085.15796	64882.47701
Q9Z329	Itpr2	Inositol 1,4,5-trisphosphate receptor type 2	59.577	1.59	307475	5	19988.04316	24841.71885	19977.74514	27624.07033	22542.79928	25872.80246	21248.51949	25387.28896	25725.97779	23361.67808	25324.92083	23316.45465	23656.156	27867.31745	21060.60574
