# Supplementary material for: Repeated Exposure of Macrophages to Synthetic Amorphous Silica Induces Adaptive Proteome Changes and a Moderate Cell Activation
Source: Nanomaterials (Basel). 2022 Apr 22;12(9):1424. doi: 10.3390/nano12091424 (PMC9105884; doi:10.3390/nano12091424)
Supplement: Supplementary file 1 [file nanomaterials-12-01424-s001.zip › Table S2.pdf]

Table S2: list of proteins modulated in response to treatment with precipitated silica

Coverage: % coverage of the protein sequence by the peptides identified in the mass spectrometry analysis
#peptides: number of peptides used for protein identification
U PR vs ctrl: result of the Mann Whitney U test in the comparison control vs treated with precipitated silica
B-H: evaluation of the false positive rate by the Benjamini-Hochberg method
SGoF+ : evaluation of the false positive rate by the Sequential Goodness of Fit method
Sfisher : : evaluation of the false positive rate by the Sequential Fisher test method

accession number	gene_name	description	protein_set _score	coverage	MW	#peptides	U pr vs ctrl	ratio PR/ctrl	B-H	SGoF+	Sfisher
A0A0B4J1G0	Fcgr4	Low affinity immunoglobulin gamma Fc region receptor IV	72.487	18.07	28398	4	0	0.70647232	0.002224	0	0
A1L314	Mpeg1	Macrophage-expressed gene 1 protein	674.945	37.87	78390	18	0	1.72939303	0.011752	1.4863942E-232	0
A2ADY9	Ddi2	Protein DDI1 homolog 2	137.299	18.8	44591	4	0	0.58794931	0.010214	7.9209309E-261	0
A2APV2	Fmnl2	Formin-like protein 2	199.494	4.88	123101	6	0	0.88410624	0.008997	2.7730879E-283	0
A9UMV8	H2afj	Histone H2A.J	423.071	64.34	14045	9	0	1.10804837	0.029274	1.8244111E-131	6.0622739E-202
B0BNM9	GLTP	Glycolipid transfer protein	119.801	32.54	23704	4	0	0.67720267	0.012682	9.9453868E-225	0
B9EJ86	Osbpl8	Oxysterol-binding protein-related protein 8	668.143	27.11	101269	18	0	1.26530957	0.008063	4.4363205E-294	0
E9PVA8	Gcn1	eIF-2-alpha kinase activator GCN1	950.724	15.46	293021	30	0	1.24035538	0.025521	1.9862693E-148	4.5504231E-228
E9Q555	Rnf213	E3 ubiquitin-protein ligase RNF213	1509.702	11.59	584784	40	0	1.24941177	0.0087	5.3527353E-284	0
E9Q7G0	Numa1	Nuclear mitotic apparatus protein 1	1707.656	34.62	235630	48	0	0.81143072	0.005135	0	0
F1LMZ8	Psmd11	26S proteasome non-ATPase regulatory subunit 11	596.643	43.13	47464	16	0	1.15881201	0.007835	5.7646359e-311	0
F1LQ48	Hnrnpl	Heterogeneous nuclear ribonucleoprotein L	894.689	60.67	67903	25	0	0.82745035	0.004489	0	0

G5E829	Atp2b1	Plasma membrane calcium-transporting ATPase 1	383.056	12.54	134747	11	0	1.24452434	0.03984	6.1296177E-86	2.9142912E-135
O08528	Hk2	Hexokinase-2	826.505	39.04	102535	30	0	0.83772963	0.07505	1.6194543E-28	5.2690089E-57
O08529	Capn2	Calpain-2 catalytic subunit	654.542	36.43	79872	20	0	0.87928496	0.061276	5.3020797E-46	4.4413436E-80
O08553	Dpysl2	Dihydropyrimidinase-related protein 2	1630.64	79.2	62278	33	0	0.74141408	0.001188	0	0
O08583	Alyref	THO complex subunit 4	246.457	41.96	26940	5	0	0.57738177	0.006432	3.4912655e-319	0
O08709	Prdx6	Peroxiredoxin-6	499.923	80.36	24871	13	0	0.82868378	0.013727	3.2377391E-220	0
O08739	Ampd3	AMP deaminase 3	178.866	10.05	88652	8	0	1.29438732	0.02368	7.6061336E-163	7.8468903E-251
O08807	Prdx4	Peroxiredoxin-4	341.044	54.74	31053	12	0	1.2954477	0.005367	0	0
O08997	Atox1	Copper transport protein ATOX1	124.127	58.82	7338	4	0	0.67163739	0.055562	1.0031745E-51	9.5481778E-88
O09106	Hdac1	Histone deacetylase 1	262.786	23.24	55075	7	0	0.85799916	0.051736	6.1718233E-60	6.4309689E-99
O09131	Gsto1	Glutathione S-transferase omega-1	126.438	29.17	27498	6	0	0.86504129	0.057716	1.1356506E-49	5.5428854E-85
O09159	Man2b1	Lysosomal alpha-mannosidase	557.178	24.38	114648	17	0	0.54320371	0.001188	0	0
O35129	Phb2	Prohibitin-2	503.598	58.19	33296	15	0	1.12983593	0.018274	1.6378345E-188	2.9667428E-293
O35295	Purb	Transcriptional activator protein Pur-beta	243.206	24.38	33901	4	0	0.79872641	0.037205	2.3005038E-98	4.3390717E-153
O35344	Kpna3	Importin subunit alpha-4	211.239	15.16	57773	5	0	1.3347877	0.035734	2.9524072E-106	1.7282975E-164
O35601	Fyb1	FYN-binding protein 1	254	13.92	90055	9	0	1.16785176	0.052965	3.1392057E-55	1.6193787E-92
O35609	Scamp3	Secretory carrier-associated membrane protein 3	184.82	16.33	38458	3	0	0.81661913	0.030798	8.2495148E-123	7.1832476E-189
O35658	C1qbp	Complement component 1 Q subcomponent-binding protein, mitochondrial	176.214	24.82	31013	5	0	1.24658249	0.028622	1.2647574E-135	2.8751219E-208
O35682	Myadm	Myeloid-associated differentiation marker	99.873	13.12	35285	3	0	1.23427099	0.010103	2.3124309E-273	0
O35691	Pnn	Pinin	55.561	3.17	82436	2	0	0.29052094	0.001188	0	0

O35737	Hnrnph1	Heterogeneous nuclear ribonucleoprotein H	631.314	41.2	49199	14	0	0.86956008	0.011191	7.9390655E-240	0
O35964	Sh3gl1	Endophilin-A2	113.857	13.59	41492	4	0	0.72367191	0.014603	1.8005191E-214	0
O35987	Nsfl1c	NSFL1 cofactor p47	378.771	31.08	40680	8	0	0.82960963	0.020084	8.0409777E-178	2.9573354E-275
O54734	Ddost	Dolichyl-diphosphooligosaccharide--protein glycosyltransferase 48 kDa subunit	568.017	50.57	49028	15	0	1.27255907	0.001188	0	0
O54782	Man2b2	Epididymis-specific alpha-mannosidase	109.642	6.39	115610	3	0	0.52966468	0.029274	5.986259E-131	3.6796444E-201
O54988	Slk	STE20-like serine/threonine-protein kinase	256.181	9	141457	9	0	0.72742318	0.005135	0	0
O55023	Impa1	Inositol monophosphatase 1	249.78	32.85	30436	7	0	0.80024582	0.014748	6.5439262E-207	0
O55126	Nipsnap2	Protein NipSnap homolog 2	84.772	21.71	32933	4	0	0.64663038	0.164185	1	0.014553036
O55173	Pdpk1	3-phosphoinositide-dependent protein kinase 1	52.966	4.47	63593	2	0	0.49361961	0.033895	6.8107435E-115	3.984154E-177
O70370	Ctss	Cathepsin S	545.861	51.76	38475	11	0	0.73787218	0.001905	0	0
O70439	Stx7	Syntaxin-7	430.805	44.83	29821	8	0	0.81522052	0.006381	0	0
O70492	Snx3	Sorting nexin-3	216.177	62.35	18757	8	0	0.8155763	0.02442	4.0279998E-156	3.5271212E-240
O70503	Hsd17b12	Very-long-chain 3-oxoacyl-CoA reductase	331.683	42.31	34742	10	0	1.18862654	0.008078	1.8396375E-290	0
O70593	Sgta	Small glutamine-rich tetratricopeptide repeat-containing protein alpha	62.997	7.96	34157	2	0	0.62947562	0.009488	7.4036663E-282	0
O88339	Epn1	Epsin-1	34.239	5.74	60158	2	0	0.67453201	0.02707	1.686759E-142	7.5745291E-219
O88384	Vti1b	Vesicle transport through interaction with t-SNAREs homolog 1B	104.755	15.09	26713	3	0	0.61389224	0.020981	3.7350406E-173	1.5017292E-267
O88569	Hnrnpa2b1	Heterogeneous nuclear ribonucleoproteins A2/B1	1252.754	68.84	37403	26	0	0.80569954	0.00966	1.3506458E-277	0
O88746	Tom1	Target of Myb protein 1	200.291	21.14	54325	6	0	0.8650813	0.065553	2.8580516E-40	1.7756947E-72

O88844	Idh1	Isocitrate dehydrogenase [NADP] cytoplasmic	570.313	39.13	46674	14	0	0.83330349	0.00381	0	0
O88958	Gnpda1	Glucosamine-6-phosphate isomerase 1	167.953	39.1	32549	6	0	0.66236905	0.005367	0	0
O89001	Cpd	Carboxypeptidase D	105.263	3.49	152406	5	0	1.19493637	0.003303	0	0
O89017	Lgmn	Legumain	154.612	13.1	49373	3	0	0.52723812	0.01245	3.0494471E-231	0
O89023	Tpp1	Tripeptidyl-peptidase 1	205.19	24.56	61342	5	0	0.63687121	0.007393	1.9484499e-315	0
P00493	Hprt1	Hypoxanthine-guanine phosphoribosyltransferase	409.853	59.17	24570	10	0	0.78872128	0.005367	0	0
P01899	H2-D1	H-2 class I histocompatibility antigen, D-B alpha chain	255.44	28.45	40836	9	0	1.30979099	0.010109	1.4827993E-270	0
P01900	H2-D1	H-2 class I histocompatibility antigen, D-D alpha chain	393.694	33.42	41110	12	0	1.27314072	0.01032	9.1968549E-259	0
P01901	H2-K1	H-2 class I histocompatibility antigen, K-B alpha chain	85.959	9.21	41302	4	0	1.31820789	0.010247	1.888527E-259	0
P01902	H2-K1	H-2 class I histocompatibility antigen, K-D alpha chain	345.838	33.15	41490	11	0	1.42907365	0.001905	0	0
P02262		Histone H2A type 1	466.815	63.85	14077	10	0	1.09853146	0.037286	3.4314231E-95	1.6464505E-148
P04441	Cd74	H-2 class II histocompatibility antigen gamma chain	251.246	21.51	31557	6	0	1.09325279	0.048897	1.9817453E-64	4.6972975E-105
P05063	Aldoc	Fructose-bisphosphate aldolase C	1174.329	80.17	39395	30	0	0.81567825	0.004996	0	0
P05064	Aldoa	Fructose-bisphosphate aldolase A	1657.605	92.31	39356	40	0	0.85526035	0.004841	0	0
P05555	Itgam	Integrin alpha-M CD11b	968.769	29.31	127481	23	0	0.88841505	0.009994	6.887236E-277	0

P06151	Ldha	L-lactate dehydrogenase A chain	1682.741	84.64	36499	35	0	0.90082785	0.008063	1.5653415E-295	0
P06339	H2-T23	H-2 class I histocompatibility antigen, D-37 alpha chain	147.539	8.68	40875	3	0	1.47266368	0.00272	0	0
P06745	Gpi	Glucose-6-phosphate isomerase	1529.065	69.53	62767	39	0	0.88373383	0.002581	0	0
P06761	Hspa5	Endoplasmic reticulum chaperone BiP	1805.756	54.59	72347	39	0	1.2330325	0.018526	6.4139631E-185	3.6683803E-287
P07091	S100a4	Protein S100-A4	293.402	51.49	11721	8	0	0.74607229	0.041148	1.1211458E-81	3.3760221E-129
P07901	Hsp90aa1	Heat shock protein HSP 90-alpha	1880.892	60.3	84788	51	0	1.12848265	0.038678	1.2818026E-91	2.3060817E-143
P08003	Pdia4	Protein disulfide-isomerase A4	868.491	44.04	71982	24	0	1.18278331	0.009488	3.8154148E-281	0
P08101	Fcgr2	Low affinity immunoglobulin gamma Fc region receptor II	293.703	39.7	36695	9	0	0.77335257	0.014276	5.1273742E-217	0
P08113	Hsp90b1	Endoplasmin	1785.373	54.49	92476	49	0	1.17504432	0.001754	0	0
P08228	Sod1	Superoxide dismutase [Cu-Zn]	173.22	34.42	15943	4	0	0.75623393	0.004489	0	0
P08508	Fcgr3	Low affinity immunoglobulin gamma Fc region receptor III	55.362	8.05	30036	2	0	0.721257	0.008063	5.4846854E-297	0
P08752	Gnai2	Guanine nucleotide-binding protein G(i) subunit alpha-2	1256.755	69.86	40489	26	0	0.83641181	0.006731	1.9671421e-318	0
P08775	Polr2a	DNA-directed RNA polymerase II subunit RPB1	350.364	7.41	217176	10	0	0.8373587	0.005977	0	0
P09103	P4hb	Protein disulfide-isomerase	1539.052	66.6	57058	38	0	1.33211212	0.005367	0	0
P09528	Fth1	Ferritin heavy chain	222.436	41.21	21067	6	0	1.97155513	0.007657	1.0410799e-311	0
P09581	Csf1r	Macrophage colony-stimulating factor 1 receptor	258.036	13	109179	11	0	0.70849846	0.005135	0	0
P09671	Sod2	Superoxide dismutase [Mn], mitochondrial	338.067	51.35	24603	8	0	1.3323457	0.016728	5.2250241E-197	7.40343e-308
P0C0S6	H2afz	Histone H2A.Z	301.957	53.91	13553	6	0	1.11299771	0.04608	3.034856E-70	4.3544226E-113

P0CC09	Hist2h2aa3	Histone H2A type 2-A	539.504	63.85	14095	11	0	1.08375735	0.042765	6.3904228E-78	7.0531385E-124
P10126	Eef1a1	Elongation factor 1-alpha 1	1368.036	67.53	50114	40	0	1.09470744	0.019838	5.3753383E-179	3.2752178E-277
P10605	Ctsb	Cathepsin B	1041.968	69.32	37280	22	0	0.73515917	0.016096	4.4218731E-200	3.2756004e-313
P10711	Tcea1	Transcription elongation factor A protein 1	175.251	20.93	33880	4	0	0.58802858	0.097041	5.2105243E-10	3.9952221E-31
P11030	Dbi	Acyl-CoA-binding protein	123.494	39.08	10027	2	0	0.63898258	0.058177	1.1703906E-48	1.2663725E-83
P11103	Parp1	Poly [ADP-ribose] polymerase 1	499.094	19.94	113100	14	0	0.72860564	0.012682	5.025984E-226	0
P11499	Hsp90ab1	Heat shock protein HSP 90-beta	1898.325	58.01	83281	49	0	1.11023126	0.036697	1.9280103E-103	2.0578361E-160
P11506	Atp2b2	Plasma membrane calcium-transporting ATPase 2	135.354	5.63	136811	6	0	1.53222942	0.017223	9.6802208E-193	1.7463861E-300
P11598	Pdia3	Protein disulfide-isomerase A3	945.818	52.28	56623	26	0	1.13792224	0.007943	5.3253421e-308	0
P11688	Itga5	Integrin alpha-5	144.39	8.64	115043	8	0	1.37739716	0.039706	2.0452251E-89	3.2164616E-140
P11730	Camk2g	Calcium/calmodulin-dependent protein kinase type II subunit gamma	105.492	8.92	59038	4	0	0.87805961	0.041279	5.3499752E-80	8.1445146E-127
P11835	Itgb2	Integrin beta-2	1205.242	41.76	85026	25	0	0.8640331	0.008574	7.30108E-287	0
P12001	Rpl18	60S ribosomal protein L18	125.06	22.34	21659	5	0	1.55549103	0.010109	9.2429203E-268	0
P12265	Gusb	Beta-glucuronidase	922.641	47.69	74195	24	0	0.69189001	0.014663	1.1217639E-210	0
P12815	Pdcd6	Programmed cell death protein 6	164.125	42.41	21867	4	0	0.81598601	0.014254	1.1790799E-217	0
P13020	Gsn	Gelsolin	1819.541	61.03	85942	44	0	0.73387199	0.001754	0	0
P14069	S100a6	Protein S100-A6	189.18	78.65	10051	6	0	0.63064077	0.005135	0	0
P14115	Rpl27a	60S ribosomal protein L27a	165.384	22.3	16605	4	0	1.17762372	0.016096	2.5763983E-201	2.2744158e-315
P14131	Rps16	40S ribosomal protein S16	263.487	52.05	16445	10	0	1.14364855	0.008078	9.6825185E-290	0
P14148	Rpl7	60S ribosomal protein L7	220.793	31.48	31420	8	0	2.19264458	0.002645	0	0
P14152	Mdh1	Malate dehydrogenase, cytoplasmic	618.435	47.6	36511	13	0	0.8610661	0.001754	0	0
P14206	Rpsa	40S ribosomal protein SA	721.327	57.63	32838	16	0	0.77006492	0.006427	6.1857019e-320	0
P14211	Calr	Calreticulin	725.785	58.41	47995	23	0	1.13393898	0.010214	2.766416E-264	0

P14685	Psmd3	26S proteasome non-ATPase regulatory subunit 3	410.713	25.66	60718	11	0	1.12571745	0.033915	1.9615411E-113	5.5276351E-175
P14733	Lmnb1	Lamin-B1	1089.586	57.99	66786	32	0	0.87205419	0.002232	0	0
P15702	Spn	Leukosialin	103.556	9.62	40038	2	0	2.15663744	0.059338	5.4773405E-48	9.9830246E-83
P15864	Hist1h1c	Histone H1.2	41.584	16.04	21267	3	0	1.63581744	0.127722	0.17942049	6.7073405E-15
P16045	Lgals1	Galectin-1	739.544	94.81	14866	15	0	0.74552845	0.04527	4.9017272E-71	3.4216834E-114
P16110	Lgals3	Galectin-3	576.284	30.68	27515	12	0	0.67572795	0.0147	2.0341904E-209	0
P16446	Pitpna	Phosphatidylinositol transfer protein alpha isoform	125.049	40.96	31907	8	0	0.71887062	0.002897	0	0
P16460	Ass1	Argininosuccinate synthase	229.08	26.21	46584	10	0	6.50898018	0.00966	2.6440654E-278	0
P16546	Sptan1	Spectrin alpha chain, non-erythrocytic 1	408.587	7.2	284597	13	0	0.75374462	0.024136	2.7928663E-162	6.254734E-250
P16675	Ctsa	Lysosomal protective protein	740.128	35.65	53844	18	0	0.79336391	0.012716	1.9537882E-223	0
P17078	Rpl35	60S ribosomal protein L35	42.539	18.7	14553	2	0	2.04972731	0.0034	0	0
P17182	Eno1	Alpha-enolase	1915.87	79.26	47141	40	0	0.88935395	0.010593	2.7287213E-253	0
P17225	Ptbp1	Polypyrimidine tract-binding protein 1	1128.374	66.98	56478	21	0	0.83076123	0.042795	1.0977251E-76	3.8685187E-122
P17742	Ppia	Peptidyl-prolyl cis-trans isomerase A	1008.804	71.34	17971	20	0	0.7955285	0.001905	0	0
P18242	Ctsd	Cathepsin D	859.885	54.39	44954	24	0	0.71539651	0.033979	1.6831324E-111	3.8133636E-172
P18572	Bsg	Basigin	59.597	9.77	42445	3	0	1.2433021	0.02442	8.616213E-158	8.0294103E-243
P18760	Cfl1	Cofilin-1	690.857	73.49	18560	14	0	0.79858187	0.02063	6.7344247E-175	2.0354326E-270
P19096	Fasn	Fatty acid synthase	3301.881	53.55	272428	87	0	0.8832528	0.022455	5.7549382E-168	4.558205E-259
P19253	Rpl13a	60S ribosomal protein L13a	144.874	27.59	23464	6	0	1.38421736	0.001628	0	0
P19783	Cox4i1	Cytochrome c oxidase subunit 4 isoform 1, mitochondrial	181.422	30.77	19530	5	0	1.10709563	0.018274	6.5328044E-188	3.1068274E-292
P19973	Lsp1	Lymphocyte-specific protein 1	598.308	46.06	36714	12	0	0.73542697	0.014663	4.7812723E-210	0
P20060	Hexb	Beta-hexosaminidase subunit beta	453.303	38.06	61116	16	0	0.76276347	0.033979	5.547066E-112	7.471955E-173
P20108	Prdx3	Thioredoxin-dependent peroxide reductase, mitochondrial Prx3	201.247	42.8	28127	8	0	1.15747552	0.018046	1.0237401E-189	2.6565419E-295
P21575	Dnm1	Dynamin-1	42.89	3.94	97295	4	0	1.12425483	0.004809	0	0

P23198	Cbx3	Chromobox protein homolog 3	366.566	42.62	20855	7	0	0.82238848	0.028962	1.4025549E-134	1.1303804E-206
P24051	Rps27l	40S ribosomal protein S27-like	146.25	25	9477	3	0	0.85140594	0.018984	1.5352435E-182	3.8127704E-283
P24270	Cat	Catalase	342.177	24.1	59795	9	0	1.20464178	0.010474	1.1817281E-254	0
P24369	Ppib	Peptidyl-prolyl cis-trans isomerase B	393.827	43.06	23713	11	0	1.09790244	0.042049	1.3985353E-79	3.1879681E-126
P24452	Capg	Macrophage-capping protein	858.851	52.56	39240	17	0	0.83609508	0.003961	0	0
P25113	Pgam1	Phosphoglycerate mutase 1	1015.485	74.02	28832	21	0	0.83022014	0.039706	1.5327178E-88	5.6623196E-139
P25976	Ubtf	Nucleolar transcription factor 1	64.217	6.54	89509	4	0	0.80211235	0.020325	4.587242E-176	2.400759E-272
P26039	Tln1	Talin-1	5661.689	64.62	269821	120	0	0.92799632	0.081312	1.1775164E-20	2.3681193E-46
P26883	Fkbp1a	Peptidyl-prolyl cis-trans isomerase FKBP1A	125.15	25	11923	2	0	0.73125356	0.010816	6.7766544E-250	0
P27008	Parp1	Poly [ADP-ribose] polymerase 1	679.782	24.85	112660	16	0	0.75362268	0.024636	1.4454916E-155	2.6702606E-239
P27659	Rpl3	60S ribosomal protein L3	722.272	44.42	46110	23	0	1.23045443	0.007943	9.6847911e-309	0
P27661	H2afx	Histone H2AX	358.821	52.45	15143	7	0	1.11648757	0.046927	6.8285766E-68	7.9071518E-110
P27773	Pdia3	Protein disulfide-isomerase A3	1310.895	67.33	56678	34	0	1.09864325	0.005541	0	0
P29066	Arrb1	Beta-arrestin-1	207.183	21.77	47020	7	0	0.84166995	0.010878	1.5262062E-248	0
P29351	Ptpn6	Tyrosine-protein phosphatase non-receptor type 6	1259.199	70.42	67559	36	0	1.21026869	0.018291	2.6008886E-187	3.2429935E-291
P29391	Ftl1	Ferritin light chain 1	365.894	55.74	20802	7	0	1.62070185	0.018701	2.5299075E-184	3.7383743E-286
P29758	Oat	Ornithine aminotransferase, mitochondrial	625.5	51.94	48355	17	0	1.26763319	0.002581	0	0
P30681	Hmgb2	High mobility group protein B2	399.936	29.05	24162	10	0	0.72365943	0.000398	0	0
P30993	C5ar1	C5a anaphylatoxin chemotactic receptor 1	103.201	11.68	39023	4	0	0.74919896	0.006427	0	0
P31938	Map2k1	Dual specificity mitogen-activated protein kinase kinase 1	480.979	39.95	43474	11	0	0.84707131	0.039396	9.8117821E-91	4.2463805E-142
P32020	Scp2	Non-specific lipid-transfer protein	78.683	5.3	59126	3	0	0.71911348	0.036655	6.5794893E-104	4.3580767E-161

P32577	Csk	Tyrosine-protein kinase CSK	379.565	33.56	50746	12	0	0.90721345	0.017745	6.3516139E-191	2.3061935E-297
P34884	Mif	Macrophage migration inhibitory factor	364.546	71.3	12504	8	0	0.71538462	0.01119	7.9435728E-242	0
P34960	Mmp12	Macrophage metalloelastase	124.013	12.26	54971	5	0	1.74335443	0.008524	1.3969525E-287	0
P35293	Rab18	Ras-related protein Rab-18	80.318	18.93	23035	3	0	1.4985006	0.005465	0	0
P35564	Canx	Calnexin	771.865	29.1	67278	17	0	1.23001742	0.001188	0	0
P35700	Prdx1	Peroxiredoxin-1	593.265	64.82	22176	20	0	1.15915024	0.001754	0	0
P36552	Cpox	Oxygen-dependent coproporphyrinogen-III oxidase, mitochondrial	58.206	7.45	49715	2	0	0.67622473	0.032052	2.5968408E-120	3.86946E-185
P38060	Hmgcl	Hydroxymethylglutaryl-CoA lyase, mitochondrial	443.427	42.46	34239	10	0	0.81981376	0.026623	3.5050503E-145	5.3404203E-223
P38647	Hspa9	Stress-70 protein, mitochondrial	1481.185	53.31	73461	32	0	1.26373687	0.027703	8.8470931E-139	4.2233638E-213
P40124	Cap1	Adenylyl cyclase-associated protein 1	1729.781	73.42	51565	40	0	0.80410376	0.002646	0	0
P41105	Rpl28	60S ribosomal protein L28	142.453	39.42	15733	6	0	1.20822692	0.01968	3.5665409E-180	3.5661251E-279
P41123	Rpl13	60S ribosomal protein L13	166.598	28.44	24309	6	0	1.4788861	0.010214	1.1318334E-265	0
P41216	Acsl1	Long-chain-fatty-acid--CoA ligase 1	279.735	22.46	77951	14	0	1.19771273	0.033979	5.9882813E-113	2.8464704E-174
P42082	Cd86	T-lymphocyte activation antigen CD86	79.02	10.68	34666	3	0	1.22754843	0.011436	7.5548813E-236	0
P42667	Sec11a	Signal peptidase complex catalytic subunit SEC11A	70.665	16.2	20599	4	0	1.2643091	0.001754	0	0
P43883	Plin2	Perilipin-2	41.644	6.35	46646	2	0	3.39576324	0.083273	5.5821718E-20	2.0220204E-45
P45376	Akr1b1	Aldose reductase	575.159	56.65	35732	20	0	0.88181047	0.008576	3.8091621E-286	0
P46978	Stt3a	Dolichyl-diphosphooligosaccharide--protein glycosyltransferase subunit STT3A	246.339	18.44	80598	11	0	1.20627168	0.010807	6.2561212E-252	0

P47753	Capza1	F-actin-capping protein subunit alpha-1	366.653	55.94	32940	11	0	0.90437112	0.015006	4.8760759E-205	5.9781943e-322
P47754	Capza2	F-actin-capping protein subunit alpha-2	660.331	70.28	32967	14	0	0.89958584	0.040894	1.6020949E-82	2.133256E-130
P47757	Capzb	F-actin-capping protein subunit beta	867.592	66.79	31345	21	0	0.76325169	0.008063	1.9083243E-298	0
P47911	Rpl6	60S ribosomal protein L6	224.616	24.32	33510	7	0	2.34611915	0.00277	0	0
P48036	Anxa5	Annexin A5	1571.408	83.39	35752	38	0	1.09533771	0.026404	1.0131883E-145	7.7507253E-224
P48441	Idua	Alpha-L-iduronidase	73.994	3.31	71254	2	0	0.75673042	0.024638	5.177361E-155	2.0068537E-238
P48758	Cbr1	Carbonyl reductase [NADPH] 1	110.481	25.99	30641	4	0	0.65104702	0.052965	6.0782462E-56	1.7340564E-93
P48962	Slc25a4	ADP/ATP translocase 1	705.604	58.39	32904	22	0	1.17674934	0.010549	5.6836222E-254	0
P49717	Mcm4	DNA replication licensing factor MCM4	823.128	32.48	96736	22	0	0.80365756	0.001905	0	0
P50247	Ahcy	Adenosylhomocysteinase	813.817	58.33	47688	23	0	0.87297604	0.00318	0	0
P50396	Gdi1	Rab GDP dissociation inhibitor alpha	659.795	46.31	50522	16	0	0.71990621	0.122148	0.043881312	1.2164954E-16
P50399	Gdi2	Rab GDP dissociation inhibitor beta	1482.039	80	50537	37	0	0.83150433	0.021369	7.6941925E-171	9.3650834E-264
P50429	Arsb	Arylsulfatase B	484.17	37.83	59647	14	0	0.53141643	0.002645	0	0
P50543	S100a11	Protein S100-A11	164.439	71.43	11083	4	0	0.69434345	0.0147	3.6618863E-208	0
P51174	Acadl	Long-chain specific acyl-CoA dehydrogenase, mitochondrial	859.062	52.09	47908	20	0	1.11623109	0.026044	2.4189286E-147	2.2914015E-226
P51569	Gla	Alpha-galactosidase A	333.553	26.25	47643	9	0	0.68351814	0.008063	4.1792951E-302	0
P51859	Hdgf	Hepatoma-derived growth factor	165.045	31.22	26269	7	0	0.71447577	0.010807	1.3077335E-252	0
P51881	Slc25a5	ADP/ATP translocase 2	804.91	58.72	32931	23	0	1.21865417	0.004574	0	0
P52293	Kpna2	Importin subunit alpha-1	562.347	37.05	57928	10	0	1.23022806	0.019505	9.1611116E-181	3.6776048E-280
P52875	Tmem165	Transmembrane protein 165	69.156	13.62	34791	2	0	1.30791907	0.029029	4.6568713E-134	7.0190038E-206

P53811	Pitpnb	Phosphatidylinositol transfer protein beta isoform	114.69	34.69	31487	7	0	0.80690335	0.001974	0	0
P53986	Slc16a1	Monocarboxylate transporter 1	98.015	6.49	53267	3	0	1.57531638	0.147048	1	4.4458464E-07
P54116	Stom	Erythrocyte band 7 integral membrane protein	430.834	47.18	31375	11	0	0.61535642	0.227397	1	0.99976338
P54822	Adsl	Adenylosuccinate lyase	453.506	35.12	54866	11	0	0.82958046	0.01382	2.7064606E-218	0
P55264	Adk	Adenosine kinase	112.439	13.57	40149	4	0	0.77204533	0.080352	6.9714041E-21	1.1489478E-46
P55302	Lrpap1	Alpha-2-macroglobulin receptor-associated protein	230.143	22.5	42215	7	0	1.30585562	0.004996	0	0
P56135	Atp5mf	ATP synthase subunit f, mitochondrial	50.03	34.09	10344	4	0	1.22139303	0.008063	2.9326536E-296	0
P56959	Fus	RNA-binding protein FUS	350.781	22.2	52673	10	0	0.87144626	0.006261	0	0
P57716	Ncstn	Nicastrin	174.822	12.01	78492	6	0	1.26846882	0.006427	1.0938613e-320	0
P58389	Ptpa	Serine/threonine-protein phosphatase 2A activator	250.352	36.53	36710	8	0	0.7189622	0.02442	4.9810084E-160	2.3144257E-246
P59017	Bcl2l13	Bcl-2-like protein 13	170.758	15.9	46719	4	0	0.33969098	0.070372	1.8252184E-35	3.8344508E-66
P59325	Eif5	Eukaryotic translation initiation factor 5	111.347	7.46	48968	4	0	0.81931174	0.010913	1.6702475E-244	0
P60335	Pcbp1	Poly(rC)-binding protein 1	636.305	63.48	37498	14	0	0.8725778	0.04313	4.7187444E-75	7.7383729E-120
P60710	Actb	Actin, cytoplasmic 1	3245.62	90.93	41737	62	0	1.06002161	0.054418	2.03509E-52	1.0969394E-88
P60766	Cdc42	Cell division control protein 42 homolog	384.278	58.12	21259	9	0	0.82556444	0.039706	2.2703259E-86	7.0557667E-136
P60843	Eif4a1	Eukaryotic initiation factor 4A-I	1018.101	54.93	46154	24	0	1.25857338	0.008078	5.0872379E-289	0
P60867	Rps20	40S ribosomal protein S20	130.94	28.57	13373	4	0	0.85008882	0.00274	0	0
P61161	Actr2	Actin-related protein 2	1171.859	58.38	44761	26	0	0.86873201	0.007943	1.6017244E-306	0
P61211	Arl1	ADP-ribosylation factor-like protein 1	127.795	19.89	20412	3	0	1.29382878	0.008063	3.5502754E-299	0
P61314	Rpl15	60S ribosomal protein L15	216.757	38.73	24146	8	0	1.53441236	0.001974	0	0
P61354	Rpl27	60S ribosomal protein L27	150.104	36.03	15798	5	0	1.2454984	0.006115	0	0

P61971	Nutf2	Nuclear transport factor 2	149.849	71.65	14478	4	0	0.5349008	0.014663	2.6270356E-211	0
P61979	Hnrnpk	Heterogeneous nuclear ribonucleoprotein K	846.419	50.76	50976	21	0	0.88000571	0.037005	4.0385336E-101	4.6122606E-157
P62242	Rps8	40S ribosomal protein S8	343.425	45.67	24205	8	0	1.57592642	0.008063	1.023958E-297	0
P62267	Rps23	40S ribosomal protein S23	229.807	42.66	15808	5	0	1.17465397	0.010099	9.0356385E-275	0
P62702	Rps4x	40S ribosomal protein S4, X isoform	648.212	53.23	29598	17	0	1.10950181	0.008052	7.7075176E-303	0
P62751	Rpl23a	60S ribosomal protein L23a	84.407	24.36	17695	3	0	1.42881343	0.019086	2.3487487E-181	3.7385879E-281
P62774	Mtpn	Myotrophin	279.736	75.42	12861	5	0	0.69862956	0.039706	3.0946763E-87	4.1075381E-137
P62830	Rpl23	60S ribosomal protein L23	191.96	50	14865	7	0	0.92280147	0.01319	3.8105351E-222	0
P62855	Rps26	40S ribosomal protein S26	110.364	31.3	13015	3	0	1.10491249	0.010214	1.6178783E-261	0
P62908	Rps3	40S ribosomal protein S3	525.947	70.78	26674	19	0	1.12332808	0.018291	1.0335605E-186	3.3702047E-290
P62918	Rpl8	60S ribosomal protein L8	246.047	45.14	28025	11	0	1.24937443	0.005465	0	0
P63001	Rac1	Ras-related C3 botulinum toxin substrate 1	404.337	54.17	21450	9	0	0.76942349	0.01119	1.7138145E-240	0
P63024	Vamp3	Vesicle-associated membrane protein 3	185.691	54.37	11480	3	0	0.69577243	0.012552	1.2560175E-228	0
P63038	Hspd1	60 kDa heat shock protein, mitochondrial	2093.763	71.38	60955	46	0	1.12524427	0.006313	0	0
P63087	Ppp1cc	Serine/threonine-protein phosphatase PP1-gamma catalytic subunit	538.526	44.58	36984	11	0	0.84074336	0.030596	8.1495432E-124	2.2113395E-190
P63158	Hmgb1	High mobility group protein B1	259.891	35.81	24894	9	0	0.73703062	0.006427	9.8813129e-324	0
P63213	Gng2	Guanine nucleotide-binding protein G(I)/G(S)/G(O) subunit gamma-2	99.828	49.3	7850	3	0	0.72270714	0.010913	3.6555619E-243	0
P63259	Actg1	Actin, cytoplasmic 2	3229.127	90.93	41793	61	0	1.06162827	0.052867	5.0895425E-57	5.9729905E-95

P63328	Ppp3ca	Serine/threonine-protein phosphatase 2B catalytic subunit alpha isoform	225.344	23.22	58644	9	0	0.8474695	0.016394	3.1009702E-198	5.482952e-310
P67778	Phb	Prohibitin	743.077	79.04	29820	18	0	1.12395878	0.04313	7.2287157E-76	5.5170974E-121
P68037	Ube2l3	Ubiquitin-conjugating enzyme E2 L3	192.778	51.95	17862	4	0	0.83694926	0.009488	1.9627815E-280	0
P68101	Eif2s1	Eukaryotic translation initiation factor 2 subunit 1	535.04	54.6	36108	15	0	0.88157769	0.092764	2.3137217E-12	1.1284038E-34
P68372	Tubb4b	Tubulin beta-4B chain	2065.138	82.92	49831	45	0	0.86134606	0.010816	1.4241426E-250	0
P69897	Tubb5	Tubulin beta-5 chain	2252.156	83.11	49671	46	0	0.86381007	0.010214	6.7138646E-263	0
P70158	Smpdl3a	Acid sphingomyelinase-like phosphodiesterase 3a	75.152	8.99	49858	2	0	0.51911478	0.021369	2.9012205E-170	8.1976948E-263
P70168	Kpnb1	Importin subunit beta-1	1005.834	39.84	97184	26	0	0.87036258	0.032457	2.555936E-119	1.1596862E-183
P70206	Plxna1	Plexin-A1	150.334	3.59	211099	6	0	1.25898111	0.025399	5.6752531E-149	6.3688984E-229
P70315	Was	Wiskott-Aldrich syndrome protein homolog	167.146	14.04	54192	4	0	0.75906982	0.037867	2.1328609E-93	6.3909443E-146
P70340	Smad1	Mothers against decapentaplegic homolog 1	42.288	8.17	52157	2	0	0.66080207	0.008034	1.4189492E-303	0
P70665	Siae	Sialate O-acetylesterase	82.931	7.39	60775	3	0	0.67495208	0.001905	0	0
P70670	Naca	Nascent polypeptide-associated complex subunit alpha, muscle-specific form	297.931	3.2	220499	6	0	1.18116453	0.037231	1.8755128E-97	8.9387935E-152
P80317	Cct6a	T-complex protein 1 subunit zeta	846.636	56.12	58004	24	0	1.1009306	0.024871	6.6037752E-154	1.1218333E-236
P81155	Vdac2	Voltage-dependent anion-selective channel protein 2	562.666	50.51	31746	12	0	1.31038855	0.003833	0	0
P83732	Rpl24	60S ribosomal protein L24	113.715	24.2	17779	4	0	1.52791466	0.007992	2.6077153E-304	0
P83870	Phf5a	PHD finger-like domain-containing protein 5A	32.466	21.82	12405	2	0	0.43978365	0.100378	3.13338E-09	6.3291479E-30
P83917	Cbx1	Chromobox protein homolog 1	135.822	18.92	21418	3	0	0.79687447	0.007992	8.7613389E-306	0
P83940	Eloc	Elongin-C	181.167	37.5	12473	3	0	0.74995964	0.005367	0	0

P84099	Rpl19	60S ribosomal protein L19	89.065	13.27	23466	2	0	2.09790193	0.003833	0	0
P85108	Tubb2a	Tubulin beta-2A chain	1611.259	58.2	49907	36	0	0.85574711	0.01245	1.3774954E-230	0
P97429	Anxa4	Annexin A4	866.33	67.71	35916	20	0	1.37702407	0.003303	0	0
P97821	Ctsc	Dipeptidyl peptidase 1	238.605	18.18	52376	7	0	0.6424995	0.002645	0	0
P99026	Psmb4	Proteasome subunit beta type-4	360.035	40.91	29116	9	0	0.88372202	0.025363	1.0498757E-151	3.2901466E-233
P99029	Prdx5	Peroxiredoxin-5, mitochondrial	406.2	56.67	21897	11	0	0.75888946	0.02442	1.8115994E-159	1.7821644E-245
Q00519	Xdh	Xanthine dehydrogenase/oxidase	800.625	23.52	146562	19	0	0.87936125	0.078196	9.5452171E-23	3.2019321E-49
Q00612	G6pdx	Glucose-6-phosphate 1-dehydrogenase X	931.64	55.92	59263	28	0	1.15374874	0.03319	7.4991131E-117	5.1994323E-180
Q01405	Sec23a	Protein transport protein Sec23A	135.088	7.32	86162	4	0	0.56969317	0.137788	1	1.2669836E-10
Q01730	Rsu1	Ras suppressor protein 1	225.544	37.18	31550	7	0	0.83681699	0.017018	1.4509335E-194	1.2496147E-303
Q03265	Atp5f1a	ATP synthase subunit alpha, mitochondrial	1612.934	62.39	59753	35	0	1.1011114	0.020325	1.1938586E-176	2.5903655E-273
Q04447	Ckb	Creatine kinase B-type	1299.978	64.04	42713	23	0	0.87382301	0.006381	0	0
Q04750	Top1	DNA topoisomerase 1	324.79	22.69	90876	14	0	0.64629325	0.002565	0	0
Q04899	Cdk18	Cyclin-dependent kinase 18	112.94	10.64	51848	4	0	1.81107392	0.001851	0	0
Q06138	Cab39	Calcium-binding protein 39	276.569	24.63	39843	9	0	1.21865246	0.01245	2.7956175E-229	0
Q09014	Ncf1	Neutrophil cytosol factor 1	176.654	15.9	44667	6	0	0.9276363	0.031688	8.2524725E-121	7.0004459E-186
Q11136	Pepd	Xaa-Pro dipeptidase	322.341	29.21	55029	11	0	0.78464705	0.027121	6.7037659E-141	2.2484049E-216
Q14C51	Ptcd3	Pentatricopeptide repeat domain-containing protein 3, mitochondrial	73.369	6.13	77796	4	0	1.35400099	0.102141	4.7293022E-08	4.5745683E-28
Q1AAU6	Asap1	Arf-GAP with SH3 domain, ANK repeat and PH domain-containing protein 1	376.54	17.92	127088	13	0	0.85051363	0.010913	3.5606395E-245	0
Q3KRE8	Tubb2b	Tubulin beta-2B chain	1629.306	64.27	49953	36	0	0.85578097	0.012617	1.1267883E-226	0
Q3TBD2	Arhgap45	Rho GTPase-activating protein 45	339.323	15.77	122902	11	0	0.763648	0.030558	7.7648706E-126	2.0270493E-193

Q3TCN2	Plbd2	Putative phospholipase B-like 2	263.875	17	66289	8	0	0.57279741	0.02057	1.7592775E-175	2.2208533E-271
Q3U0V1	Khsrp	Far upstream element-binding protein 2	709.8	30.48	76775	19	0	0.88023471	0.014633	7.7588194E-214	0
Q3U4I7	Pyroxd2	Pyridine nucleotide-disulfide oxidoreductase domain-containing protein 2	48.518	3.79	62940	2	0	0.80994137	0.13158	0.39584153	1.0343647E-13
Q3U9G9	Lbr	Lamin-B receptor	489.882	25.56	71440	14	0	1.36741542	0.010214	1.3640495E-263	0
Q3UFY7	Nt5c3b	7-methylguanosine phosphate-specific 5'-nucleotidase	19.393	6.67	34425	2	0	0.79922275	0.052965	1.3829367E-55	5.304216E-93
Q3UGP9	Lrrc58	Leucine-rich repeat-containing protein 58	61.816	12.84	40139	2	0	0.76261216	0.074415	9.1347569E-32	2.7529402E-61
Q3URQ0	Tex10	Testis-expressed protein 10	104.337	5.71	105209	4	0	1.18378932	0.037113	3.3757367E-100	9.8583747E-156
Q3USZ8		Deleted in autism protein 1 homolog	77.072	5.35	49463	2	0	1.51547194	0.006069	0	0
Q3UW53	Fam129a	Protein Niban	737.731	34.99	102649	25	0	0.84527425	0.004489	0	0
Q3UZA1	Rcsd1	CapZ-interacting protein	48.015	7.28	44114	2	0	0.38494613	0.022243	1.5378297E-168	5.3743896E-260
Q3V0K9	Pls1	Plastin-1	323.882	11.59	70408	9	0	1.12962005	0.024898	2.3517016E-153	8.3171126E-236
Q4FZY0	Efhd2	EF-hand domain-containing protein D2	624.41	58.16	26759	16	0	0.86521885	0.01119	3.6929991E-241	0
Q4G009	Mcts1	Malignant T-cell-amplified sequence 1	100.927	20.33	20550	2	0	0.63446034	0.013313	7.3781004E-221	0
Q4KLL4	Tm9sf4	Transmembrane 9 superfamily member 4	85.689	4.35	74675	2	0	1.41173671	0.005465	0	0
Q4KM65	Nudt21	Cleavage and polyadenylation specificity factor subunit 5	267.979	46.26	26240	7	0	0.77381383	0.004489	0	0
Q4QRB4	Tubb3	Tubulin beta-3 chain	1113.19	33.11	50419	24	0	0.83473383	0.009488	1.0079459E-279	0
Q4V7C7	Actr3	Actin-related protein 3	974.506	76.56	47357	29	0	0.84660212	0.00741	6.0684493e-314	0

Q501J6	Ddx17	Probable ATP-dependent RNA helicase DDX17	518.414	21.23	72399	11	0	0.87588988	0.010913	1.7055879E-242	0
Q568Z6	Ist1	IST1 homolog	30.577	4.37	39942	2	0	0.78161266	0.002036	0	0
Q571I9	Aldh16a1	Aldehyde dehydrogenase family 16 member A1	241.958	13.47	84756	7	0	0.8099287	0.001628	0	0
Q5FWK3	Arhgap1	Rho GTPase-activating protein 1	222.831	31.89	50411	9	0	0.68468955	0.045336	1.2210269E-70	1.2224753E-113
Q5I0H4	Tmco1	Calcium load-activated calcium channel	107.898	19.68	21175	3	0	1.13118411	0.082036	3.3320407E-20	9.92958E-46
Q5RK09	Eif3g	Eukaryotic translation initiation factor 3 subunit G	45.846	9.69	35651	3	0	1.55542866	0.116754	0.008764224	2.3554255E-18
Q5SSL4	Abr	Active breakpoint cluster region-related protein	138.765	8.5	97667	5	0	0.66174964	0.045063	1.9634089E-71	9.5367115E-115
Q5SUR0	Pfas	Phosphoribosylformylglycinamidine synthase	587.731	26.78	144629	19	0	0.72356688	0.008063	1.248509E-292	0
Q5U2R0	Mat2b	Methionine adenosyltransferase 2 subunit beta	219.535	23.65	37375	5	0	0.87970741	0.0147	8.6386429E-209	0
Q5XI73	Arhgdia	Rho GDP-dissociation inhibitor 1	574.961	77.94	23407	15	0	0.78308913	0.004708	0	0
Q5XIP6	Fen1	Flap endonuclease 1	128.983	21.32	42609	5	0	0.83539885	0.014806	2.758747E-206	4.9406565e-324
Q60591	Nfatc2	Nuclear factor of activated T-cells, cytoplasmic 2	116.59	6.15	100020	3	0	0.71169797	0.026991	1.210194E-144	3.6545373E-222
Q60597	Ogdh	2-oxoglutarate dehydrogenase, mitochondrial	708.109	30.6	116449	22	0	1.14091725	0.003961	0	0
Q60605	Myl6	Myosin light polypeptide 6	651.916	66.23	16930	13	0	0.78613413	0.0109	7.5770144E-246	0
Q60668	Hnrnpd	Heterogeneous nuclear ribonucleoprotein D0	240.764	22.54	38354	7	0	0.79696227	0.011481	3.4559873E-235	0
Q60710	Samhd1	Deoxynucleoside triphosphate triphosphohydrolase SAMHD1	668.224	44.38	75893	21	0	0.69007046	0.017063	5.8956328E-194	1.4056842E-302
Q60854	Serpinb6	Serpin B6	1109.272	64.81	42599	24	0	0.64178853	0.003961	0	0

Q60855	Ripk1	Receptor-interacting serine/threonine-protein kinase 1	56.794	3.2	74854	2	0	0.78071621	0.064173	7.9110117E-42	1.5471527E-74
Q60872	Eif1a	Eukaryotic translation initiation factor 1A	127.34	18.06	16502	2	0	0.58219867	0.004421	0	0
Q60931	Vdac3	Voltage-dependent anion-selective channel protein 3	444.29	43.11	30753	9	0	1.15267527	0.01245	6.2112042E-230	0
Q61036	Pak3	Serine/threonine-protein kinase PAK 3	120.297	10.38	62398	5	0	0.89673424	0.011436	1.64855E-236	0
Q61093	Cybb	Cytochrome b-245 heavy chain	329.147	37.89	65305	13	0	1.38097561	0.01089	1.6095016E-246	0
Q61191	Hcfc1	Host cell factor 1	498.608	10.12	210437	13	0	0.83301378	0.006955	1.1064531e-317	0
Q61207	Psap	Prosaposin	294.177	14.54	61422	7	0	0.48399718	0.010214	3.2987132E-262	0
Q61210	Arhgef1	Rho guanine nucleotide exchange factor 1	557.637	26.09	102805	17	0	0.8861993	0.037439	7.6200873E-94	1.4456544E-146
Q61263	Soat1	Sterol O-acyltransferase 1	202.63	14.26	63799	6	0	1.21137853	0.010447	5.0813884E-256	0
Q61545	Ewsr1	RNA-binding protein EWS	59.664	4.43	68462	2	0	0.85119539	0.032545	2.4955126E-118	3.4005702E-182
Q61686	Cbx5	Chromobox protein homolog 5	139.648	18.85	22186	3	0	0.68645357	0.029274	1.6845441E-132	1.6360212E-203
Q61879	Myh10	Myosin-10	1972.797	32.44	228996	54	0	0.91831557	0.10173	2.4432757E-08	1.5925377E-28
Q61990	Pcbp2	Poly(rC)-binding protein 2	555.288	49.17	38222	13	0	0.89903175	0.025399	1.6184257E-149	8.8953856E-230
Q62188	Dpysl3	Dihydropyrimidinase-related protein 3	260.334	8.95	61936	6	0	0.76514525	0.010099	3.5057626E-276	0
Q62193	Rpa2	Replication protein A 32 kDa subunit	79.469	13.7	29718	2	0	0.70363933	0.003679	0	0
Q62318	Trim28	Transcription intermediary factor 1-beta	935.768	42.21	88847	22	0	0.89467382	0.018235	4.0985781E-189	2.8197188E-294
Q62418	Dbnl	Drebrin-like protein	465.119	31.19	48700	10	0	0.81996042	0.10458	1.0702299E-06	7.7509027E-26
Q62433	Ndrg1	Protein NDRG1	888.864	60.91	43009	18	0	0.60950211	0.003688	0	0
Q62465	Vat1	Synaptic vesicle membrane protein VAT-1 homolog	373.432	35.47	43097	9	0	0.732685	0.015006	2.044348E-204	7.6283736e-321
Q62523	Zyx	Zyxin	273.581	18.62	60546	8	0	0.83470364	0.038313	1.6604424E-92	1.2255549E-144
Q63799	Tcea2	Transcription elongation factor A protein 2	53.729	7.69	33528	2	0	0.7452502	0.007943	2.9231207E-307	0

Q63921	Ptgs1	Prostaglandin G/H synthase 1	208.36	22.43	69032	9	0	0.76632108	0.030596	2.5954644E-123	1.2615957E-189
Q63945	Set	Protein SET	264.432	39.79	33406	8	0	0.87616703	0.065132	6.8567908E-41	2.692681E-73
Q64012	Raly	RNA-binding protein Raly	231.157	32.05	33188	10	0	0.86909378	0.012946	8.6362518E-223	0
Q640N3	Arhgap30	Rho GTPase-activating protein 30	48.964	5.18	120114	3	0	0.71962334	0.00391	0	0
Q641Z6	Ehd1	EH domain-containing protein 1	626.153	65.73	60603	27	0	1.15473907	0.010103	2.9544748E-271	0
Q64232	Tecr	Very-long-chain enoyl-CoA reductase	127.618	13.96	36123	6	0	1.27307619	0.008997	1.4341263E-282	0
Q64430	Atp7a	Copper-transporting ATPase 1	60.924	2.88	161959	3	0	1.27813191	0.026991	4.9234707E-143	1.1271584E-219
Q64514	Tpp2	Tripeptidyl-peptidase 2	740.218	25.91	139879	24	0	0.89905788	0.014911	1.1608854E-205	4.4465908e-323
Q64522	Hist2h2ab	Histone H2A type 2-B	380.051	57.69	14013	7	0	1.11488153	0.053703	4.089987E-53	1.2362474E-89
Q66H50	Far1	Fatty acyl-CoA reductase 1	225.411	18.45	59268	6	0	1.25181431	0.019068	6.0105301E-182	3.7860781E-282
Q66HA6	Arl8b	ADP-ribosylation factor-like protein 8B	360.396	53.76	21539	11	0	0.82833398	0.036235	7.6149946E-105	1.9249292E-162
Q68FD5	Cltc	Clathrin heavy chain 1	4079.117	65.73	191557	96	0	0.88023033	0.010447	2.16948E-257	0
Q6A0D4	Rftn1	Raftlin	82.367	11.91	61537	4	0	0.77265081	0.011223	3.6710954E-239	0
Q6AXS3	Dek	Protein DEK	232.107	21.16	42892	7	0	0.67475074	0.043308	1.9584821E-73	1.4778192E-117
Q6AXY7	Prpf38b	Pre-mRNA-splicing factor 38B	55.077	3.32	63932	2	0	0.65224841	0.303439	1	1
Q6AYE2	Sh3glb1	Endophilin-B1	166.687	16.16	40787	5	0	0.79960727	0.081312	1.9834942E-20	4.8538761E-46
Q6DFW4	Nop58	Nucleolar protein 58	431.638	23.51	60343	9	0	0.85035158	0.008623	1.9838484E-285	0
Q6GQT9	Nomo1	Nodal modulator 1	253.918	11.2	133420	9	0	1.40831648	0.001905	0	0
Q6IMY8	Hnrnpu	Heterogeneous nuclear ribonucleoprotein U	1237.314	46.87	87732	34	0	0.89543776	0.024703	1.8508342E-154	1.503669E-237
Q6NSR8	Npepl1	Probable aminopeptidase NPEPL1	194.656	18.89	55940	7	0	0.73730998	0.012682	4.4120745E-224	0
Q6P3A8	Bckdhb	2-oxoisovalerate dehydrogenase subunit beta, mitochondrial	28.169	9.49	42880	2	0	0.56317763	0.032486	7.994514E-119	6.2895623E-183
Q6P5F9	Xpo1	Exportin-1	972.08	36.79	123093	28	0	0.87718911	0.016096	1.0683395E-200	2.7345965e-314
Q6PCT3	Tpd52l2	Tumor protein D54	38.425	9.55	23992	2	0	0.74963305	0.035732	9.9515848E-107	3.5636396E-165

Q6PDG5	Smarcc2	SWI/SNF complex subunit SMARCC2	128.747	5.94	132604	5	0	0.83012269	0.005135	0	0
Q6PDM2	Srsf1	Serine/arginine-rich splicing factor 1	185.744	32.66	27745	7	0	0.7980103	0.051273	2.6387651E-60	2.0185921E-99
Q6Q0N3	Nt5dc2	5'-nucleotidase domain-containing protein 2	130.949	10.49	63653	4	0	0.69456725	0.017933	2.5523478E-190	2.4862031E-296
Q6Q899	Ddx58	Probable ATP-dependent RNA helicase DDX58	113.886	6.59	105975	4	0	1.50297929	0.057716	2.3698281E-50	6.7981065E-86
Q6TUG0	Dnajb11	DnaJ homolog subfamily B member 11	290.148	30.17	40495	7	0	1.27209039	0.012556	2.521612E-227	0
Q6URK4	Hnrnpa3	Heterogeneous nuclear ribonucleoprotein A3	923.97	41.42	39652	20	0	0.83483471	0.002283	0	0
Q6WVG3	Kctd12	BTB/POZ domain-containing protein KCTD12	340.99	40.06	35892	8	0	0.828796	0.01585	1.4901293E-202	1.5399833e-317
Q6ZQI3	Mlec	Malectin	104.181	12.37	32342	3	0	1.18089517	0.007882	3.1864146e-310	0
Q6ZWQ7	Spcs3	Signal peptidase complex subunit 3	55.049	17.78	20313	3	0	1.37935962	0.004996	0	0
Q71LX4	Tln2	Talin-2	478.82	5.35	253621	15	0	0.922664	0.03692	1.3925139E-101	9.9257345E-158
Q78IK2	Atp5md	Up-regulated during skeletal muscle growth protein 5	166.998	44.83	6382	3	0	1.14467686	0.02442	2.382661E-158	1.0501669E-243
Q78P75	Dynll2	Dynein light chain 2, cytoplasmic	86.955	25.84	10350	2	0	0.65425814	0.011223	1.6945031E-238	0
Q7TPJ0	Ssr1	Translocon-associated protein subunit alpha	59.898	10.66	35629	3	0	1.34199657	0.040089	4.4395281E-85	4.91991E-134
Q7TPR4	Actn1	Alpha-actinin-1	1341.234	44.73	103068	32	0	0.84834479	0.014633	1.4328996E-212	0
Q80SW1	Ahcyl1	S-adenosylhomocysteine hydrolase-like protein 1	265.391	25.28	58951	10	0	0.87551117	0.023429	2.0675259E-163	9.7627754E-252
Q80VA0	Galnt7	N-acetylgalactosaminyltransferase 7	30.194	5.02	75419	2	0	1.28460698	0.022823	8.0137836E-167	3.1945228E-257
Q80VD1	Fam98b	Protein FAM98B	80.732	6.76	45349	2	0	0.70531475	0.022929	1.5189608E-164	1.4749478E-253
Q80X41	Vrk1	Serine/threonine-protein kinase VRK1	196.372	25	49741	8	0	0.86481896	0.014276	2.225649E-216	0

Q811U4	Mfn1	Mitofusin-1	14.964	2.43	83726	2	0	1.7580448	0.026075	8.4169356E-147	1.6032954E-225
Q8BG07	Pld4	Phospholipase D4	660.298	46.52	56154	18	0	0.75784231	0.010214	3.8711092E-260	0
Q8BGH2	Samm50	Sorting and assembly machinery component 50 homolog	237.323	22.81	51864	8	0	1.17294611	0.022921	1.1074926E-165	2.1889831E-255
Q8BH61	F13a1	Coagulation factor XIII A chain	164.935	14.48	83207	9	0	0.33486593	0.037005	1.1688182E-100	2.1346473E-156
Q8BH86	Dglucy	D-glutamate cyclase, mitochondrial	162.102	14.42	66366	5	0	0.63589418	0.010863	3.2188595E-249	0
Q8BH95	Echs1	Enoyl-CoA hydratase, mitochondrial	206.452	23.1	31474	5	0	0.81513449	0.028103	1.0044837E-137	1.7647169E-211
Q8BHN3	Ganab	Neutral alpha-glucosidase AB	999.162	43.64	106911	31	0	1.17099724	0.003128	0	0
Q8BHZ0	Fam49a	Protein FAM49A	122.261	18.58	37343	4	0	0.6425975	0.022921	4.1054063E-165	1.7985393E-254
Q8BK67	Rcc2	Protein RCC2	650.406	48.27	55983	19	0	0.79109885	0.010099	1.7813689E-275	0
Q8BLF1	Nceh1	Neutral cholesterol ester hydrolase 1	471.958	49.51	45740	12	0	1.25090458	0.012164	6.7385886E-232	0
Q8BMJ3	Eif1ax	Eukaryotic translation initiation factor 1A, X-chromosomal	158.528	18.75	16460	3	0	0.71586333	0.020981	9.8125402E-174	1.6738047E-268
Q8BMK4	Ckap4	Cytoskeleton-associated protein 4	910.017	51.48	63692	24	0	1.2708903	0.002645	0	0
Q8BMS1	Hadha	Trifunctional enzyme subunit alpha, mitochondrial	1127.238	47.44	82670	24	0	0.88411555	0.016728	1.2740708E-197	6.4235631e-309
Q8BPU7	Elmo1	Engulfment and cell motility protein 1	416.403	28.61	83936	16	0	0.63874326	0.01089	3.4127792E-247	0
Q8BTI8	Srrm2	Serine/arginine repetitive matrix protein 2	362.852	6.4	294840	11	0	0.10393214	0.004071	0	0
Q8BUM3	Ptpn7	Tyrosine-protein phosphatase non-receptor type 7	193.124	21.73	40351	5	0	0.68376691	0.0034	0	0
Q8BYI6	Lpcat2	Lysophosphatidylcholine acyltransferase 2	90.418	10.11	60254	4	0	1.62363762	0.010109	7.4287632E-270	0
Q8C2Q3	Rbm14	RNA-binding protein 14	241.333	13.75	69449	7	0	0.80845939	0.008063	2.2622063E-301	0
Q8C9B9	Dido1	Death-inducer obliterator 1	38.162	1.37	247176	2	0	0.78660403	0.020981	2.5730587E-174	1.8567014E-269
Q8CCF0	Prpf31	U4/U6 small nuclear ribonucleoprotein Prp31	53.378	7.62	55430	2	0	0.55106488	0.002916	0	0

Q8CGY8	Ogt	UDP-N-acetylglucosamine--peptide N-acetylglucosaminyltransferase 110 kDa subunit	158.065	6.41	116952	5	0	0.6859119	0.1436	1	8.8880854E-08
Q8CI94	Pygb	Glycogen phosphorylase, brain form	767.631	34.4	96730	24	0	0.87121218	0.013735	1.4182431E-219	0
Q8CIM5	Gpr84	G-protein coupled receptor 84	233.413	22.22	43717	6	0	1.95619714	0.004996	0	0
Q8CIN7	Impa2	Inositol monophosphatase 2	46.609	11.38	31796	2	0	0.74267965	0.016096	1.8268529E-199	3.9119852e-312
Q8CIZ9	Nox1	NADPH oxidase 1	27.213	2.54	68193	2	0	1.41721077	0.02126	2.0366913E-171	1.0625714E-264
Q8JZR0	Acsl5	Long-chain-fatty-acid--CoA ligase 5	333.003	24.6	76206	12	0	1.11085515	0.042052	3.6479845E-79	1.2354719E-125
Q8JZX4	Rbm17	Splicing factor 45	45.392	7.41	45304	2	0	0.54532392	0.010103	5.8763887E-272	0
Q8K183	Pdxk	Pyridoxal kinase	413.942	39.42	35015	9	0	0.67496115	0.003679	0	0
Q8K297	Colgalt1	Procollagen galactosyltransferase 1	282.896	14.59	71061	9	0	1.30789365	0.003303	0	0
Q8K2C9	Hacd3	Very-long-chain (3R)-3-hydroxyacyl-CoA dehydratase 3	128.075	17.68	43131	4	0	1.21371879	0.085409	3.1376933E-18	5.1951958E-43
Q8K3G9	Appl2	DCC-interacting protein 13-beta	215.632	25.23	73854	10	0	0.71027757	0.010214	2.2832875E-266	0
Q8QZT1	Acat1	Acetyl-CoA acetyltransferase, mitochondrial	549.109	47.88	44816	11	0	0.85669917	0.016096	6.2018E-202	1.8830886e-316
Q8QZY1	Eif3l	Eukaryotic translation initiation factor 3 subunit L	688.491	35.64	66613	16	0	1.18335008	0.053031	1.6063142E-54	1.498696E-91
Q8R180	Ero1a	ERO1-like protein alpha	524.829	53.88	54084	17	0	1.42675172	0.007992	4.7840385E-305	0
Q8R1S4	Mtss1	Metastasis suppressor protein 1	214.905	13.31	82408	7	0	0.81628643	0.008063	3.4891046E-291	0
Q8R491	Ehd3	EH domain-containing protein 3	259.129	23.55	60791	13	0	1.11414094	0.011481	1.5781029E-234	0
Q8R5J9	Arl6ip5	PRA1 family protein 3	153.183	23.94	21558	5	0	1.35304727	0.025326	2.9652105E-152	4.517346E-234
Q8VBT0	Tmx1	Thioredoxin-related transmembrane protein 1	168.028	16.91	31396	4	0	1.19003574	0.00741	3.3778227e-313	0

Q8VBT9	Aspscr1	Tether containing UBX domain for GLUT4	60.7	6.91	59796	2	0	0.7523823	0.014602	4.1706945E-215	0
Q8VCN5	Cth	Cystathionine gamma-lyase	349.244	29.9	43567	8	0	1.66872057	0.006261	0	0
Q8VCT3	Rnpep	Aminopeptidase B	1034.075	55.85	72416	27	0	0.82494866	0.005135	0	0
Q8VDD5	Myh9	Myosin-9	6108.517	65.77	226372	143	0	0.9043874	0.025399	3.7100801E-151	2.3875547E-232
Q8VDK1	Nit1	Deaminated glutathione amidase	137.33	25.08	35705	4	0	0.600417	0.029465	2.0897637E-129	8.066379E-199
Q8VDL4	Adpgk	ADP-dependent glucokinase	153.351	11.69	53902	4	0	1.33746085	0.011481	7.1931128E-234	0
Q8VE37	Rcc1	Regulator of chromosome condensation	276.016	30.64	44931	7	0	0.83645968	0.008063	1.2223677E-300	0
Q8VEB4	Pla2g15	Group XV phospholipase A2	146.52	21.84	47307	6	0	0.7071917	0.025399	1.3085528E-150	1.7241376E-231
Q8VEH3	Arl8a	ADP-ribosylation factor-like protein 8A	273.771	44.09	21390	7	0	0.7723031	0.02442	1.3669201E-160	2.9917284E-247
Q8VIJ6	Sfpq	Splicing factor, proline- and glutamine-rich	665.644	31.9	75442	18	0	0.86427596	0.052133	7.7891519E-59	2.0230343E-97
Q8WTY4	Ciapin1	Anamorsin	142.093	29.77	33429	5	0	0.41999251	0.001754	0	0
Q91V04	Tram1	Translocating chain-associated membrane protein 1	171.159	19.25	43039	6	0	1.20510236	0.093025	2.530448E-11	3.9977584E-33
Q91V76		Ester hydrolase C11orf54 homolog	97.997	18.1	34996	3	0	0.5756321	0.011436	3.5908387E-237	0
Q91XU1	Qki	Protein quaking	203.341	23.75	37643	5	0	0.67876751	0.008239	2.6681682E-288	0
Q91YQ5	Rpn1	Dolichyl-diphosphooligosaccharide--protein glycosyltransferase subunit 1	874.429	46.71	68528	22	0	1.21993176	0.001188	0	0
Q91YR9	Ptgr1	Prostaglandin reductase 1	329.123	36.47	35560	9	0	0.76224778	0.002654	0	0
Q91ZX7	Lrp1	Prolow-density lipoprotein receptor-related protein 1	2764.038	22.4	504742	73	0	0.74962365	0.003961	0	0
Q921F4	Hnrnpll	Heterogeneous nuclear ribonucleoprotein L-like	394.855	33.84	64125	13	0	0.80096617	0.00741	1.8768908e-312	0
Q921M7	Fam49b	Protein FAM49B	581.935	58.64	36776	15	0	0.84146784	0.030363	2.4139024E-126	3.4893544E-194

Q922B2	Dars	Aspartate--tRNA ligase, cytoplasmic	547.159	46.51	57147	22	0	1.24136346	0.014276	9.6433497E-216	0
Q922D4	Ppp6r3	Serine/threonine-protein phosphatase 6 regulatory subunit 3	123.538	4.15	94653	4	0	1.31510251	0.055914	4.8988124E-51	8.1521557E-87
Q922F4	Tubb6	Tubulin beta-6 chain	1199.02	64.65	50090	27	0	0.9182704	0.051189	1.1256316E-60	6.3173719E-100
Q922R8	Pdia6	Protein disulfide-isomerase A6	791.765	48.64	48100	16	0	1.13373465	0.010109	3.7152028E-269	0
Q924Z4	Cers2	Ceramide synthase 2	118.544	11.84	45024	3	0	1.68581727	0.017223	2.3911665E-193	1.5735532E-301
Q93092	Taldo1	Transaldolase	956.355	77.74	37387	28	0	0.84203906	0.001188	0	0
Q99JX4	Eif3m	Eukaryotic translation initiation factor 3 subunit M	280.026	29.14	42517	7	0	0.70092107	0.020285	3.1012621E-177	2.7792793E-274
Q99KC8	Vwa5a	von Willebrand factor A domain-containing protein 5A	1382.493	55.23	87143	31	0	0.83808105	0.006193	0	0
Q99KH8	Stk24	Serine/threonine-protein kinase 24	344.392	27.15	47954	10	0	0.76387524	0.006427	0	0
Q99KP3	Cryl1	Lambda-crystallin homolog	80.371	14.42	35209	3	0	0.64093997	0.01378	6.2011288E-219	0
Q99L20	Gstt3	Glutathione S-transferase theta-3	18.425	9.13	27403	2	0	0.82952936	0.008063	2.3555266E-293	0
Q99LQ7	Tmem189	Transmembrane protein 189	104.394	21.77	31134	3	0	0.61705299	0.062191	4.7080998E-44	1.7206606E-77
Q99LX0	Park7	Protein/nucleic acid deglycase DJ-1	481.906	74.6	20021	12	0	0.68348455	0.027136	2.2788329E-140	1.4901236E-215
Q99MR3	Slc12a9	Solute carrier family 12 member 9	175.845	8.42	96330	6	0	0.76196615	0.004489	0	0
Q99N69	Lpxn	Leupaxin	478.931	30.57	43478	11	0	0.78410735	0.010103	1.1667378E-272	0
Q99PL5	Rrbp1	Ribosome-binding protein 1	1019.678	26.48	172879	33	0	0.85953527	0.007057	6.2126191e-317	0
Q9CPQ1	Cox6c	Cytochrome c oxidase subunit 6C	35.183	19.74	8469	2	0	1.21305862	0.006427	5.9287878e-323	0
Q9CPU0	Glo1	Lactoylglutathione lyase	134.222	36.96	20810	6	0	0.77116018	0.001754	0	0
Q9CPV4	Glod4	Glyoxalase domain-containing protein 4	452.641	66.78	33317	15	0	0.81071735	0.008063	6.6059266E-292	0
Q9CPW4	Arpc5	Actin-related protein 2/3 complex subunit 5	182.71	39.07	16288	5	0	0.84640021	0.010214	4.5980049E-267	0
Q9CQI6	Cotl1	Coactosin-like protein	340.221	71.83	15944	12	0	0.85389443	0.005135	0	0
Q9CQM5	Txndc17	Thioredoxin domain-containing protein 17	67.76	18.7	14015	2	0	0.7657042	0.030558	2.4927773E-125	1.1695525E-192

Q9CQN1	Trap1	Heat shock protein 75 kDa, mitochondrial	264.616	17.28	80209	10	0	1.44078991	0.010099	4.5750644E-274	0
Q9CQW9	Ifitm3	Interferon-induced transmembrane protein 3	116.3	36.5	14954	3	0	1.46079142	0.021219	5.3810579E-172	1.2005988E-265
Q9CR57	Rpl14	60S ribosomal protein L14	119.827	21.66	23564	4	0	1.44407987	0.004686	0	0
Q9CSN1	Snw1	SNW domain-containing protein 1	136.612	9.33	61475	3	0	0.81619677	0.014633	3.3373501E-213	0
Q9CT10	Ranbp3	Ran-binding protein 3	155.084	15.07	52573	5	0	0.85462172	0.033895	6.4122543E-114	1.0702965E-175
Q9CU62	Smc1a	Structural maintenance of chromosomes protein 1A	329.71	14.19	143235	15	0	0.90085107	0.046927	2.7843134E-68	2.2836832E-110
Q9CW03	Smc3	Structural maintenance of chromosomes protein 3	732.012	24.08	141556	22	0	0.85802055	0.020981	1.4190278E-172	1.344572E-266
Q9CWJ9	Atic	Bifunctional purine biosynthesis protein PURH	1231.219	73.14	64217	30	0	0.91858341	0.039849	1.6513909E-85	1.1989933E-134
Q9CWK8	Snx2	Sorting nexin-2	876.156	52.22	58471	23	0	0.77914448	0.002645	0	0
Q9CX86	Hnrnpa0	Heterogeneous nuclear ribonucleoprotein A0	472.942	32.79	30530	10	0	0.75632042	0.00381	0	0
Q9CXY9	Pigk	GPI-anchor transamidase	60.615	5.82	44895	2	0	1.3172183	0.162736	1	0.0073283356
Q9CY64	Blvra	Biliverdin reductase A	693.431	62.37	33525	17	0	0.89607671	0.010447	4.4707827E-258	0
Q9CZU6	Cs	Citrate synthase, mitochondrial	529.968	37.93	51737	16	0	1.16536972	0.009571	5.1669872E-279	0
Q9CZY3	Ube2v1	Ubiquitin-conjugating enzyme E2 variant 1	106.734	28.57	16355	4	0	0.90897518	0.04219	9.4948799E-79	4.7759037E-125
Q9D0F3	Lman1	Protein ERGIC-53	302.23	32.3	57789	9	0	1.25585799	0.209901	1	0.96966329
Q9D172	Gatd3a	Glutamine amidotransferase-like class 1 domain-containing protein 3A, mitochondrial	48.883	12.41	28090	3	0	0.81382263	0.024994	8.3586628E-153	6.1444858E-235
Q9D2V7	Coro7	Coronin-7	315.235	17.25	100812	9	0	0.76863846	0.011422	7.80745E-238	0
Q9D6F9	Tubb4a	Tubulin beta-4A chain	1728.708	70.95	49586	37	0	0.82142871	0.011741	3.2727779E-233	0
Q9D6K9	Cers5	Ceramide synthase 5	94.804	12.56	48167	4	0	1.21242112	0.010447	1.0508854E-256	0
Q9D6U8	Fam162a	Protein FAM162A	163.641	36.13	17725	6	0	1.34741162	0.017648	1.5776941E-191	2.1258185E-298

Q9D7S7	Rpl22l1	60S ribosomal protein L22-like 1	159.879	45.08	14467	4	0	1.19844728	0.02442	1.1202902E-156	4.6491615E-241
Q9D8E6	Rpl4	60S ribosomal protein L4	384.919	37.47	47154	12	0	2.2042643	0.006261	0	0
Q9D8U8	Snx5	Sorting nexin-5	756.615	58.66	46797	23	0	0.80640699	0.004721	0	0
Q9D964	Gatm	Glycine amidinotransferase, mitochondrial	445.082	51.54	48297	15	0	0.81327482	0.010447	2.4526555E-255	0
Q9DAR7	Dcps	m7GpppX diphosphatase	396.432	47.93	38988	11	0	0.75969358	0.002645	0	0
Q9DBB8	Dhdh	Trans-1,2-dihydrobenzene-1,2-diol dehydrogenase	222.785	20.12	36301	4	0	0.75208056	0.033025	7.7741413E-118	1.8321203E-181
Q9DBC7	Prkar1a	cAMP-dependent protein kinase type I-alpha regulatory subunit	390.797	36.48	43185	11	0	0.87221557	0.027279	7.7313923E-140	9.8446908E-215
Q9DBD5	Pelp1	Proline-, glutamic acid- and leucine-rich protein 1	106.259	4.1	118069	4	0	1.19102403	0.069628	1.2186769E-36	1.1055574E-67
Q9DBG6	Rpn2	Dolichyl-diphosphooligosaccharide--protein glycosyltransferase subunit 2	1215.901	54.36	69063	23	0	1.16949227	0.005033	0	0
Q9DBP5	Cmpk1	UMP-CMP kinase	274.558	42.86	22165	8	0	0.82162955	0.010214	5.600604E-265	0
Q9DBR1	Xrn2	5'-3' exoribonuclease 2	356.657	18.72	108687	13	0	0.79756366	0.008063	6.5934373E-300	0
Q9DC53	Cpne8	Copine-8	25.264	5.2	64667	2	0	1.21405434	0.07505	8.8065801E-29	2.3450457E-57
Q9DCG9	Trmt112	Multifunctional methyltransferase subunit TRM112-like protein	42.929	20	14141	2	0	0.76626947	0.147285	1	8.6020227E-07
Q9DCL9	Paics	Multifunctional protein ADE2	428.915	45.41	47006	13	0	0.83356645	0.008063	8.3405856E-295	0
Q9DCN2	Cyb5r3	NADH-cytochrome b5 reductase 3	497.727	52.82	34128	14	0	1.21038842	0.023223	5.6093389E-164	1.2056291E-252
Q9EPB4	Pycard	Apoptosis-associated speck-like protein containing a CARD	197.84	36.79	21459	6	0	0.88387968	0.012682	2.2377642E-225	0
Q9EQ32	Pik3ap1	Phosphoinositide 3-kinase adapter protein 1	749.784	26.63	90928	18	0	0.65380223	0.001905	0	0

Q9EQK5	Mvp	Major vault protein	1063.413	46.34	95924	28	0	1.46390192	0.003961	0	0
Q9EQP2	Ehd4	EH domain-containing protein 4	669.126	44.92	61481	20	0	0.89002856	0.017285	3.9116176E-192	1.9319502E-299
Q9EQV6	Tpp1	Tripeptidyl-peptidase 1	73.575	6.22	61332	2	0	0.32350491	0.018701	3.9140869E-183	3.7976652E-284
Q9ER00	Stx12	Syntaxin-12	150.501	23.72	31195	5	0	0.61504035	0.006427	1.9317967e-321	0
Q9ERL7	Gmfg	Glia maturation factor gamma	94.209	30.28	16748	4	0	0.76793392	0.012556	5.6328643E-228	0
Q9ESY9	Ifi30	Gamma-interferon-inducible lysosomal thiol reductase	195.149	20.97	27784	6	0	0.40493894	0.000398	0	0
Q9JHF7	Hpgds	Hematopoietic prostaglandin D synthase	113.844	22.11	23227	3	0	0.67667753	0.007943	1.7582277e-309	0
Q9JHJ3	Glmp	Glycosylated lysosomal membrane protein	116.49	13.12	43804	3	0	0.70883403	0.028622	4.2159035E-135	1.8052967E-207
Q9JHS3	Lamtor2	Ragulator complex protein LAMTOR2	122.42	21.6	13480	2	0	0.61974737	0.017008	8.7392192E-196	9.7680014E-306
Q9JJU8	Sh3bgrl	SH3 domain-binding glutamic acid-rich-like protein	216.988	70.18	12811	6	0	0.77278647	0.021472	1.0918873E-169	7.1608017E-262
Q9JJZ4	Ube2j1	Ubiquitin-conjugating enzyme E2 J1	38.103	6.92	34990	2	0	1.58512159	0.001628	0	0
Q9JK81	Myg1	UPF0160 protein MYG1, mitochondrial	343.682	30.53	42723	8	0	0.80861539	0.004722	0	0
Q9JKP5	Mbnl1	Muscleblind-like protein 1	28.027	6.45	36976	2	0	0.75000913	0.010807	2.9875601E-251	0
Q9JKR6	Hyou1	Hypoxia up-regulated protein 1	1066.388	39.14	111181	29	0	1.31654352	0.006069	0	0
Q9JL26	Fmnl1	Formin-like protein 1	999.021	31.9	122060	29	0	0.90223488	0.016373	7.5335696E-199	4.6603188e-311
Q9JLJ2	Aldh9a1	4-trimethylaminobutyraldehyde dehydrogenase	687.374	69.64	53515	21	0	0.91014628	0.060842	2.4892117E-46	1.6253582E-80
Q9JM14	Nt5c	5'(3')-deoxyribonucleotidase, cytosolic type	114.573	27.5	23076	3	0	0.77068709	0.029539	7.1662314E-128	1.7172618E-196
Q9JM76	Arpc3	Actin-related protein 2/3 complex subunit 3	357.381	69.1	20525	13	0	0.8404013	0.005793	0	0
Q9JM90	Stap1	Signal-transducing adaptor protein 1	483.469	61.28	34628	14	0	1.37551934	0.049928	6.4655396E-63	5.5779284E-103

Q9JMH6	Txnrd1	Thioredoxin reductase 1, cytoplasmic	304.448	20.23	67084	7	0	1.30055549	0.036213	2.5826507E-105	4.0236621E-163
Q9QWR8	Naga	Alpha-N-acetylgalactosaminidase	268.096	27.47	47235	10	0	0.63423713	0.029468	2.2101489E-128	2.8834339E-197
Q9QYL8	Lypla2	Acyl-protein thioesterase 2	65.824	31.17	24807	3	0	0.5165359	0.018526	1.6230753E-185	3.5876897E-288
Q9QZ88	Vps29	Vacuolar protein sorting-associated protein 29	189.181	40.66	20496	6	0	0.82874225	0.004686	0	0
Q9R0Q8	Clec4e	C-type lectin domain family 4 member E	43.575	12.15	24431	2	0	5.52556332	0.014633	6.1409741E-212	0
Q9R0X4	Acot9	Acyl-coenzyme A thioesterase 9, mitochondrial	118.054	18.22	50560	6	0	1.23601496	0.049875	2.7144944E-63	1.7004053E-103
Q9R1P4	Psma1	Proteasome subunit alpha type-1	404.874	72.24	29547	15	0	0.88124919	0.015112	8.5554355E-204	9.7074018e-320
Q9R1T2	Sae1	SUMO-activating enzyme subunit 1	585.544	59.71	38620	16	0	0.83551501	0.006427	3.409053e-322	0
Q9R233	Tapbp	Tapasin	125.12	16.56	49736	6	0	1.4209438	0.010109	1.8547277E-268	0
Q9WTK5	Nfkb2	Nuclear factor NF-kappa-B p100 subunit	69.018	2.89	96832	2	0	1.64013063	0.00274	0	0
Q9WTP6	Ak2	Adenylate kinase 2, mitochondrial	334.009	47.28	26469	8	0	0.87741877	0.075898	5.9293603E-27	6.43545E-55
Q9WU78	Pdcd6ip	Programmed cell death 6-interacting protein	1224.259	53.39	96024	35	0	0.8338587	0.0034	0	0
Q9WU81	Slc37a2	Glucose-6-phosphate exchanger SLC37A2	189.923	12.38	55073	4	0	0.74221806	0.008623	1.0313925E-284	0
Q9WUA3	Pfkp	ATP-dependent 6-phosphofructokinase, platelet type	1753.233	65.31	85455	44	0	1.08061681	0.016728	2.1388555E-196	8.5158207E-307
Q9WUM3	Coro1b	Coronin-1B	170.612	30.17	53912	7	0	0.81308873	0.046786	4.5981909E-69	1.8856168E-111
Q9WUM5	Suclg1	Succinate--CoA ligase [ADP/GDP-forming] subunit alpha, mitochondrial	257.113	25.72	36155	7	0	0.88878574	0.01089	7.2235172E-248	0
Q9WUU7	Ctsz	Cathepsin Z	406.55	49.67	33996	11	0	0.7994102	0.007368	3.4822518e-316	0
Q9WV54	Asah1	Acid ceramidase	610.897	45.18	44670	17	0	0.72885311	0.029269	5.1035937E-133	2.670168E-204
Q9WVA4	Tagln2	Transgelin-2	924.244	84.42	22395	24	0	0.84456508	0.044248	1.2451795E-72	1.9939039E-116

Q9WVQ5	Apip	Methylthioribulose-1-phosphate dehydratase	113.666	9.13	26949	2	0	0.62272704	0.090111	5.8101445E-15	1.9631592E-38
Q9Z0J0	Npc2	NPC intracellular cholesterol transporter 2	140.289	41.61	16442	5	0	0.67635301	0.026991	1.4343135E-143	1.6726267E-220
Q9Z0P5	Twf2	Twinfilin-2	458.829	52.44	39471	11	0	0.84649057	0.010913	7.8208921E-244	0
Q9Z110	Aldh18a1	Delta-1-pyrroline-5-carboxylate synthase	631.966	28.43	87266	17	0	1.26538525	0.004489	0	0
Q9Z1F9	Uba2	SUMO-activating enzyme subunit 2	577.522	38.09	70569	16	0	0.79487429	0.033468	7.1756142E-116	1.4542307E-178
Q9Z277	Baz1b	Tyrosine-protein kinase BAZ1B	36.765	1.89	170651	2	0	0.75517913	0.076797	3.4126496E-24	3.4312438E-51
Q9Z2L0	Vdac1	Voltage-dependent anion-selective channel protein 1	927.384	69.61	30756	16	0	1.20646332	0.007399	1.088333e-314	0
A2AGT5	Ckap5	Cytoskeleton-associated protein 5	272.831	6.55	225635	12	1	1.07867675	0.10644	6.000189E-06	1.4953944E-24
A2RSY6	Trmt1l	TRMT1-like protein	90.625	4.4	80861	2	1	0.71207349	0.209969	1	0.97229942
B0BNG0	Emc2	ER membrane protein complex subunit 2	111.856	8.42	34870	2	1	1.24121831	0.04313	3.0533869E-74	1.0743056E-118
B2RX12	Abcc3	Canalicular multispecific organic anion transporter 2	272.445	7.88	169124	7	1	0.77565525	0.104211	7.9432841E-07	4.6905056E-26
E9Q1P8	Irf2bp2	Interferon regulatory factor 2-binding protein 2	130.928	11.58	59292	4	1	0.86138203	0.027937	2.9839883E-138	2.7393445E-212
O35130	Emg1	Ribosomal RNA small subunit methyltransferase NEP1	98.513	18.85	26974	3	1	1.81666168	0.018701	9.9603082E-184	3.7734571E-285
O35239	Ptpn9	Tyrosine-protein phosphatase non-receptor type 9	99.563	10.29	67970	4	1	0.48086682	0.015112	3.573814E-203	1.223855e-318
O35264	Pafah1b2	Platelet-activating factor acetylhydrolase IB subunit beta	163.641	36.68	25581	4	1	0.70881927	0.04313	1.201658E-74	2.8869889E-119
O35286	Dhx15	Pre-mRNA-splicing factor ATP-dependent RNA helicase DHX15	433.044	24.91	91007	16	1	1.03264499	0.051189	4.7907242E-61	1.9735819E-100
O35639	Anxa3	Annexin A3	1159.261	73.99	36384	27	1	0.9106291	0.047765	9.9389397E-67	3.1990613E-108

O35643	Ap1b1	AP-1 complex subunit beta-1	982.506	39.45	103935	25	1	0.89244327	0.02442	6.5762352E-159	1.3699479E-244
O35685	Nudc	Nuclear migration protein nudC	347.061	32.83	38358	9	1	1.16864366	0.055167	4.5236527E-52	3.2480443E-88
O54924	Exoc8	Exocyst complex component 8	44.609	5.03	81043	2	1	0.86180525	0.084476	2.581931E-19	1.6586653E-44
O70251	Eef1b	Elongation factor 1-beta	229.088	40.89	24694	6	1	1.17876384	0.053405	3.6209404E-54	4.5423729E-91
O70435	Psma3	Proteasome subunit alpha type-3	399.733	37.25	28405	8	1	0.94088869	0.066408	2.3878162E-39	2.9312176E-71
O88342	Wdr1	WD repeat-containing protein 1	1015.99	62.38	66407	24	1	0.89773927	0.036757	1.6453248E-102	4.5463082E-159
O88531	Ppt1	Palmitoyl-protein thioesterase 1	424.343	35.29	34490	10	1	0.73692126	0.08022	2.4237391E-21	2.6854219E-47
O89044	Prim2	DNA primase large subunit	90.342	4.93	58603	2	1	0.8141836	0.089294	1.4974319E-15	2.8701841E-39
P01896		H-2 class I histocompatibility antigen, alpha chain	107.51	27.03	20454	4	1	1.21040437	0.042364	2.4659371E-78	1.8391694E-124
P04256	Hnrnpa1	Heterogeneous nuclear ribonucleoprotein A1	841.217	49.38	34212	18	1	0.87995225	0.076016	1.108623E-25	3.2980694E-53
P09527	Rab7a	Ras-related protein Rab-7a	710.488	74.88	23504	17	1	0.93000795	0.027077	1.9682367E-141	3.3801467E-217
P10810	Cd14	Monocyte differentiation antigen CD14	612.498	50.55	39204	14	1	1.11535345	0.061285	1.1266516E-45	1.207931E-79
P16332	Mut	Methylmalonyl-CoA mutase, mitochondrial	107.713	7.35	82844	4	1	1.17406184	0.029207	1.5431679E-133	4.3414561E-205
P21708	Mapk3	Mitogen-activated protein kinase 3	616.377	62.37	43081	17	1	0.82574926	0.037117	9.7294378E-100	4.5354224E-155
P22437	Ptgs1	Prostaglandin G/H synthase 1	696.456	49.17	69042	19	1	0.80679997	0.033979	1.8244182E-112	1.4603972E-173
P23492	Pnp	Purine nucleoside phosphorylase	251.547	31.49	32277	7	1	0.85494088	0.055562	2.2194434E-51	2.7930813E-87
P24668	M6pr	Cation-dependent mannose-6-phosphate receptor	311.197	29.86	31172	8	1	1.0702246	0.054383	9.133995E-53	3.6951805E-89
P26151	Fcgr1	High affinity immunoglobulin gamma Fc receptor I	103.29	10.4	44888	3	1	0.80731859	0.02442	3.1098494E-157	6.1158854E-242

P27867	Sord	Sorbitol dehydrogenase	47.078	7.28	38235	2	1	0.79666368	0.032106	8.1551917E-120	2.1220275E-184
P27870	Vav1	Proto-oncogene vav	714.634	30.77	98137	20	1	1.13311384	0.061997	1.0655178E-44	2.3959637E-78
P28271	Aco1	Cytoplasmic aconitate hydratase	290.564	15.86	98126	8	1	0.81973226	0.018481	4.0996018E-186	3.4923688E-289
P28667	Marcksl1	MARCKS-related protein	68.906	17.5	20165	3	1	1.55010246	0.029274	5.5492139E-132	9.9698887E-203
P29314	Rps9	40S ribosomal protein S9	270.149	44.85	22591	13	1	1.20400384	0.040894	2.2697149E-83	1.3326255E-131
P29418	Atp5f1e	ATP synthase subunit epsilon, mitochondrial	2.148	29.41	5767	2	1	0.62587349	0.042765	1.6524615E-77	2.6851238E-123
P30204	Msr1	Macrophage scavenger receptor types I and II CD204	167.547	11.79	50170	6	1	1.38884586	0.019838	1.3859043E-179	3.4309988E-278
P32067	Ssb	Lupus La protein homolog	467.172	32.29	47756	13	1	0.92837705	0.115695	0.0042094699	4.542893E-19
P35285	Rab22a	Ras-related protein Rab-22A	73.206	22.16	21802	3	1	0.87045196	0.074341	4.8165308E-32	1.191176E-61
P36876	Ppp2r2a	Serine/threonine-protein phosphatase 2A 55 kDa regulatory subunit B alpha isoform	291.297	21.92	51678	8	1	1.12963964	0.034563	4.644855E-110	4.9189659E-170
P42932	Cct8	T-complex protein 1 subunit theta	1318.426	61.86	59555	32	1	1.10871898	0.071286	2.6262049E-34	1.2733295E-64
P46413	Gss	Glutathione synthetase	90.551	11.6	52345	4	1	0.70146263	0.078828	1.6475141E-22	6.7628213E-49
P50408	Atp6v1f	V-type proton ATPase subunit F	178.96	57.98	13370	5	1	0.67647276	0.143349	1	3.389151E-08
P50428	Arsa	Arylsulfatase A	16.653	8.89	53748	2	1	0.47864197	0.065553	5.8136309E-40	4.535092E-72
P52019	Sqle	Squalene monooxygenase	95.834	4.9	63770	2	1	1.27542822	0.037233	4.2979393E-96	8.1983737E-150
P52431	Pold1	DNA polymerase delta catalytic subunit	339.844	14.03	123790	12	1	0.87357979	0.030301	7.4892881E-127	5.984354E-195
P58044	Idi1	Isopentenyl-diphosphate Delta-isomerase 1	119.261	24.67	26289	4	1	0.8109032	0.034907	4.1986387E-109	1.2309982E-168
P61028	Rab8b	Ras-related protein Rab-8B	303.135	44.44	23603	9	1	1.17323971	0.017016	3.5641949E-195	1.1069035E-304
P61107	Rab14	Ras-related protein Rab-14	457.453	73.02	23927	13	1	0.84012067	0.041148	4.2427115E-82	8.5094746E-130

P61255	Rpl26	60S ribosomal protein L26	205.138	39.31	17258	8	1	1.12379508	0.037231	5.3383653E-97	4.0387835E-151
P61804	Dad1	Dolichyl-diphosphooligosaccharide--protein glycosyltransferase subunit DAD1	80.636	19.47	12497	3	1	1.21269414	0.03144	2.6168126E-122	4.0665034E-188
P62141	Ppp1cb	Serine/threonine-protein phosphatase PP1-beta catalytic subunit	490.999	33.94	37187	9	1	0.86342144	0.05209	3.3531989E-59	6.4326053E-98
P62309	Snrpg	Small nuclear ribonucleoprotein G	56.483	26.32	8496	2	1	0.54437178	0.040894	6.0366668E-83	5.3371172E-131
P62317	Snrpd2	Small nuclear ribonucleoprotein Sm D2	180.707	52.54	13527	7	1	0.83642344	0.102192	9.0437144E-08	1.3022141E-27
P62843	Rps15	40S ribosomal protein S15	111.328	28.28	17040	3	1	0.4124668	0.086871	8.3573121E-18	2.0198981E-42
P62869	Elob	Elongin-B	274.104	59.32	13170	7	1	0.86264413	0.042765	4.2636969E-77	1.020146E-122
P62962	Pfn1	Profilin-1	700.956	91.43	14957	16	1	0.90043415	0.066408	1.9512214E-38	4.6978742E-70
P63081	Atp6v0c	V-type proton ATPase 16 kDa proteolipid subunit c	144.756	31.61	15808	2	1	0.32211682	0.034563	1.5401868E-110	9.7912577E-171
P63085	Mapk1	Mitogen-activated protein kinase 1	445.944	63.69	41276	15	1	0.8443241	0.04762	1.6709824E-67	2.7322453E-109
P63276	Rps17	40S ribosomal protein S17	395.75	59.26	15524	10	1	0.82002543	0.024382	1.02355E-161	4.9238776E-249
P63280	Ube2i	SUMO-conjugating enzyme UBC9	104.874	34.18	18007	4	1	0.84553714	0.049632	4.7523609E-64	1.5636067E-104
P84096	Rhog	Rho-related GTP-binding protein RhoG	411.779	51.83	21309	9	1	0.86901807	0.046522	7.5263892E-70	1.5374681E-112
P97315	Csrp1	Cysteine and glycine-rich protein 1	254.274	50.78	20583	6	1	0.87918395	0.07505	4.7766156E-29	1.0417191E-57
Q01205	Dlst	Dihydrolipoyllysine-residue succinyltransferase component of 2-oxoglutarate dehydrogenase complex, mitochondrial	264.324	19.16	48925	8	1	1.1986916	0.066408	9.7112801E-39	1.8668166E-70
Q02765	Ctss	Cathepsin S	37.975	6.97	36833	2	1	0.71999641	0.078053	3.1783484E-23	7.1316289E-50

Q3U7R1	Esyt1	Extended synaptotagmin-1	422.369	14.65	121554	11	1	1.14656726	0.035732	3.3474674E-107	7.3307636E-166
Q4V8K5	Brox	BRO1 domain-containing protein BROX	159.807	18.98	46192	6	1	0.77399809	0.030558	7.98669E-125	6.7256401E-192
Q4VA53	Pds5b	Sister chromatid cohesion protein PDS5 homolog B	468.027	10.44	164419	11	1	0.85796872	0.022784	2.1495652E-167	3.8356876E-258
Q571E4	Galns	N-acetylgalactosamine-6-sulfatase	163.699	25.77	57673	7	1	0.56485818	0.085321	1.1650402E-18	1.3204172E-43
Q5BJS0	Dhx30	ATP-dependent RNA helicase DHX30	86.006	3.02	133997	3	1	1.29813725	0.232579	1	0.99995601
Q5SF07	Igf2bp2	Insulin-like growth factor 2 mRNA-binding protein 2	92.866	4.56	65584	2	1	0.82227161	0.101221	1.2470658E-08	5.4927386E-29
Q5XI72	Eif4h	Eukaryotic translation initiation factor 4H	172.757	27.02	27324	5	1	0.82820952	0.038013	5.9573176E-93	2.8045227E-145
Q5XI81	Fxr1	Fragile X mental retardation syndrome-related protein 1	163.162	6.16	63947	3	1	1.13223172	0.096954	3.6072061E-10	2.2743422E-31
Q60770	Stxbp3	Syntaxin-binding protein 3	185.306	17.74	67942	8	1	0.81712725	0.024416	3.7440378E-161	3.8452293E-248
Q61187	Tsg101	Tumor susceptibility gene 101 protein	84.606	12.28	44124	4	1	0.82053754	0.04313	1.8489261E-75	2.0684639E-120
Q62048	Pea15	Astrocytic phosphoprotein PEA-15	224.718	70.77	15054	6	1	0.61998911	0.026991	4.170343E-144	2.475601E-221
Q62261	Sptbn1	Spectrin beta chain, non-erythrocytic 1	232.782	5.46	274223	9	1	0.8169848	0.040257	1.1909494E-84	2.008736E-133
Q63060	Gk	Glycerol kinase	23.058	3.82	57477	2	1	1.47420195	0.037231	1.5163122E-96	1.82167E-150
Q63584	Tmed10	Transmembrane emp24 domain-containing protein 10	373.401	35.16	24858	7	1	1.13917231	0.044248	3.1293287E-72	7.2578626E-116
Q642C0	Dnajc8	DnaJ homolog subfamily C member 8	90.595	27.67	29813	4	1	0.84375189	0.029996	2.318982E-127	1.0187497E-195
Q64578	Atp2a1	Sarcoplasmic/ endoplasmic reticulum calcium ATPase 1	254.77	9.76	109409	6	1	1.15304747	0.062616	4.2944043E-43	3.2531806E-76
Q68FH4	Galk2	N-acetylgalactosamine kinase	137.369	16.38	50503	4	1	0.72804811	0.037233	1.2156888E-95	3.6774616E-149

Q6DVA0	Lemd2	LEM domain-containing protein 2	35.137	4.5	57507	2	1	1.37632587	0.071651	5.0827666E-34	3.0326748E-64
Q6NS46	Pdcd11	Protein RRP5 homolog	162.864	4.51	207779	7	1	1.10250993	0.089079	9.4756112E-16	1.5040482E-39
Q6P8X1	Snx6	Sorting nexin-6	162.194	18.47	46649	8	1	0.63450878	0.039706	5.6048372E-89	1.3515347E-139
Q6PE01	Snrnp40	U5 small nuclear ribonucleoprotein 40 kDa protein	172.828	19.55	39276	4	1	0.86794205	0.019838	2.0809636E-178	3.116988E-276
Q6PGH2	Jpt2	Jupiter microtubule associated homolog 2	33.619	14.74	20020	2	1	0.7231985	0.163964	1	0.013036864
Q794E4	Hnrnpf	Heterogeneous nuclear ribonucleoprotein F	906.329	65.78	45730	19	1	0.91455078	0.025399	4.606396E-150	1.2401414E-230
Q7TMR0	Prcp	Lysosomal Pro-X carboxypeptidase	92.213	13.85	55027	3	1	0.73960435	0.039645	2.7060308E-90	1.8048063E-141
Q7TSV4	Pgm2	Phosphoglucomutase -2	82.058	12.42	68748	4	1	0.56205647	0.041187	2.9562635E-81	1.3365445E-128
Q80TH2	Erbin	Erbin	56.959	3.64	157248	3	1	0.78645066	0.041187	7.7783181E-81	5.2720122E-128
Q80W54	Zmpste24	CAAX prenyl protease 1 homolog	94.956	13.26	54735	4	1	1.28830192	0.03467	1.3979257E-109	2.4656611E-169
Q810B6	Ankfy1	Rabankyrin-5	529.491	16.6	128653	12	1	0.87435635	0.039706	4.1825185E-88	2.3675902E-138
Q8BMF4	Dlat	Dihydrolipoyllysine-residue acetyltransferase component of pyruvate dehydrogenase complex, mitochondrial	477.868	30.53	67942	12	1	1.11997786	0.057492	1.0787368E-50	2.3685473E-86
Q8BP40	Acp6	Lysophosphatidic acid phosphatase type 6	157.119	18.9	47625	5	1	0.74473339	0.036801	4.7915418E-102	2.1275822E-158
Q8BQZ4	Ralgapb	Ral GTPase-activating protein subunit beta	89.737	4.25	165200	3	1	1.51203888	0.141964	1	1.2516375E-08
Q8BV66	Ifi44	Interferon-induced protein 44	59.537	5.92	47852	2	1	1.63881176	0.155368	1	0.00025260713
Q8BVI4	Qdpr	Dihydropteridine reductase	216.144	31.12	25570	5	1	0.81070874	0.028622	3.7867806E-136	4.5685089E-209
Q8BYA0	Tbcd	Tubulin-specific chaperone D	47.165	1.59	133321	2	1	1.18291059	0.0294	1.9603303E-130	2.229177E-200
Q8BZW8	Nhlrc2	NHL repeat-containing protein 2	86.826	3.17	78430	2	1	1.50029258	0.095966	1.7134936E-10	7.2983064E-32

Q8CCH2	Nhlrc3	NHL repeat-containing protein 3	234.33	30.55	38195	6	1	0.75879899	0.035202	1.1236972E-107	1.4938511E-166
Q8CGK3	Lonp1	Lon protease homolog, mitochondrial	813.421	32.67	105843	22	1	1.1532312	0.036734	5.6380564E-103	9.6870684E-160
Q8CHP8	Pgp	Glycerol-3-phosphate phosphatase	163.498	22.43	34541	4	1	0.59455365	0.0147	1.5494204E-207	0
Q8CI43	Myl6b	Myosin light chain 6B	57.825	11.59	22749	2	1	0.78142546	0.021565	4.1016046E-169	6.2202077E-261
Q8K0F1	Tbc1d23	TBC1 domain family member 23	84.48	7.02	76426	2	1	0.56529094	0.101721	1.7481843E-08	9.3671433E-29
Q8K2I4	Manba	Beta-mannosidase	232.516	13.54	100831	8	1	0.56414211	0.039706	8.3910285E-87	1.7047363E-136
Q8R0H9	Gga1	ADP-ribosylation factor-binding protein GGA1	67.638	9.76	69972	2	1	0.67697231	0.08503	4.2783188E-19	3.3227057E-44
Q8R1I1	Uqcr10	Cytochrome b-c1 complex subunit 9	85.36	37.5	7446	2	1	1.22211378	0.026222	2.9230969E-146	1.1178919E-224
Q8R5A3	Apbb1ip	Amyloid beta A4 precursor protein-binding family B member 1-interacting protein	44.92	5.22	74319	3	1	0.7184927	0.052133	9.6294393E-58	6.1928177E-96
Q8VDM6	Hnrnpul1	Heterogeneous nuclear ribonucleoprotein U-like protein 1	336.085	17.23	96002	11	1	0.78100228	0.057717	2.477244E-49	1.5779496E-84
Q8VDV3	Rab3il1	Guanine nucleotide exchange factor for Rab-3A	81.456	10.97	42713	2	1	0.610927	0.074914	6.1366358E-31	3.3399401E-60
Q99KN9	Clint1	Clathrin interactor 1	207.204	12.68	68513	6	1	0.86355841	0.156618	1	0.00064660191
Q99PD4	Arpc1a	Actin-related protein 2/3 complex subunit 1A	94.403	9.46	41599	3	1	0.70252964	0.105953	3.4196473E-06	5.635273E-25
Q9CQ22	Lamtor1	Ragulator complex protein LAMTOR1	139.49	52.8	17749	6	1	0.79491293	0.049666	1.1370777E-63	5.163113E-104
Q9CQE8	RTRAF	RNA transcription, translation and transport factor protein	265.853	27.46	28152	5	1	0.74750699	0.033122	2.4169594E-117	9.7796671E-181
Q9CQN6	Tmem14c	Transmembrane protein 14C	81.653	35.09	11642	2	1	1.2700383	0.037439	2.7167135E-94	3.2636762E-147
Q9CR51	Atp6v1g1	V-type proton ATPase subunit G 1	167.366	22.88	13724	3	1	0.67643385	0.061315	2.3883102E-45	3.2778334E-79

Q9CR62	Slc25a11	Mitochondrial 2-oxoglutarate/malate carrier protein	437.786	57.01	34155	15	1	1.13550691	0.050448	1.536514E-62	1.8247058E-102
Q9CX56	Psmd8	26S proteasome non-ATPase regulatory subunit 8	192.496	34.28	39930	10	1	1.19650552	0.059966	1.1658366E-46	5.90501E-81
Q9CXT8	Pmpcb	Mitochondrial-processing peptidase subunit beta	57.009	6.34	54614	3	1	1.20617187	0.013202	1.6782597E-221	0
Q9CZD3	Gars	Glycine--tRNA ligase	803.059	36.63	81878	22	1	1.16120729	0.063908	1.852161E-42	2.2652086E-75
Q9D4H1	Exoc2	Exocyst complex component 2	32.172	2.06	103959	2	1	1.17644231	0.051189	2.0343073E-61	6.1550633E-101
Q9D6K5	Synj2bp	Synaptojanin-2-binding protein	154.513	33.79	15815	4	1	1.27092642	0.051011	3.6432011E-62	5.9317907E-102
Q9DBS1	Tmem43	Transmembrane protein 43	323.038	39	44783	12	1	1.23114194	0.039706	1.1389094E-87	9.8732656E-138
Q9DC16	Ergic1	Endoplasmic reticulum-Golgi intermediate compartment protein 1	128.824	17.59	32562	4	1	1.30836558	0.028103	3.3747173E-137	1.1301291E-210
Q9DC50	Crot	Peroxisomal carnitine O-octanoyltransferase	63.466	6.54	70264	2	1	0.59539578	0.037174	8.0318525E-99	9.5115734E-154
Q9EPK6	Sil1	Nucleotide exchange factor SIL1	144.397	13.98	52430	4	1	1.37542004	0.079257	2.8359867E-22	1.4220649E-48
Q9ES52	Inpp5d	Phosphatidylinositol 3,4,5-trisphosphate 5-phosphatase 1	932.081	33.59	133542	29	1	0.93926367	0.052133	1.8051979E-58	6.3435121E-97
Q9ESU6	Brd4	Bromodomain-containing protein 4	39.623	2.36	155895	3	1	0.72715427	0.092764	1.0183332E-12	3.3843039E-35
Q9JHR7	Ide	Insulin-degrading enzyme	144.95	10.7	117772	7	1	0.82882629	0.037439	9.6652927E-95	7.3479639E-148
Q9JII6	Akr1a1	Alcohol dehydrogenase [NADP(+)]	477.043	65.85	36587	17	1	0.88978403	0.035927	8.7411313E-106	8.3598418E-164
Q9JK23	Psmg1	Proteasome assembly chaperone 1	110.485	12.8	33104	3	1	1.0598174	0.07505	2.5841E-29	4.6173682E-58
Q9QUN7	Tlr2	Toll-like receptor 2	67.488	2.93	89449	2	1	1.29794238	0.033707	2.2129233E-115	7.6340487E-178
Q9QY17	Pacsin2	Protein kinase C and casein kinase substrate in neurons 2 protein	45.468	4.1	55978	2	1	0.61354344	0.075971	1.9281528E-26	3.1284536E-54

Q9Z0M5	Lipa	Lysosomal acid lipase/cholesteryl ester hydrolase	246.757	32.75	45325	11	1	1.68498212	0.029468	6.8028377E-129	4.8312654E-198
Q9Z1Q5	Clic1	Chloride intracellular channel protein 1	697.267	82.99	27013	16	1	0.93544533	0.031688	8.2841334E-122	2.2743116E-187
A2AAY5	Sh3pxd2b	SH3 and PX domain-containing protein 2B	31.872	3.96	101517	3	2	1.07477241	0.074901	3.2605928E-31	1.4575771E-60
B0BNM1	Naxe	NAD(P)H-hydrate epimerase	199.691	29.43	30891	4	2	0.58233722	0.040593	8.515525E-84	3.3056619E-132
B2RQC6	Cad	CAD protein	670.669	12.18	243238	18	2	1.13239778	0.092764	1.1534038E-11	1.2259433E-33
B2RXR6	Ankrd44	Serine/threonine-protein phosphatase 6 regulatory ankyrin repeat subunit B	292.332	9.87	107385	6	2	0.78993584	0.091781	1.905273E-13	2.9502505E-36
D3ZLZ7	Impdh1	Inosine-5'-monophosphate dehydrogenase 1	75.457	5.06	55293	3	2	1.08493037	0.23935	1	0.99999763
D4AE41	Rbmxl1	RNA binding motif protein, X-linked-like-1	209.356	15.98	42250	7	2	0.91005714	0.088194	2.3606721E-16	2.1321576E-40
F1M775	Diaph1	Protein diaphanous homolog 1	461.72	12.65	140409	12	2	1.162121	0.066408	4.8214107E-39	7.4047901E-71
O08788	Dctn1	Dynactin subunit 1	292.672	10.62	141676	9	2	0.86197908	0.078053	5.985596E-24	7.3773737E-51
O08992	Sdcbp	Syntenin-1	99.91	21.07	32379	3	2	1.35209256	0.107451	1.3624285E-05	6.3534826E-24
O35381	Anp32a	Acidic leucine-rich nuclear phosphoprotein 32 family member A	234.908	25.51	28538	8	2	0.78141831	0.039038	3.5501259E-91	9.9275776E-143
O35593	Psmd14	26S proteasome non-ATPase regulatory subunit 14	213.025	36.13	34577	5	2	1.06662868	0.093739	5.4867101E-11	1.2902186E-32
O55125	Nipsnap1	Protein NipSnap homolog 1	182.332	33.45	33363	5	2	0.85452189	0.047747	4.0798311E-67	9.3651469E-109
O55142	Rpl35a	60S ribosomal protein L35a	50.285	27.27	12554	5	2	1.14233122	0.052965	1.1660451E-56	1.8422428E-94
O55143	Atp2a2	Sarcoplasmic/ endoplasmic reticulum calcium ATPase 2	625.662	20.69	114858	16	2	1.17598738	0.070794	3.5682429E-35	9.248163E-66
O70133	Dhx9	ATP-dependent RNA helicase A	1082.594	27.17	149475	30	2	0.8657253	0.027491	2.6179053E-139	6.4692055E-214

O70145	Ncf2	Neutrophil cytosol factor 2	441.898	43.62	59485	17	2	0.85629811	0.067443	1.5593683E-37	7.3528153E-69
O70172	Pip4k2a	Phosphatidylinositol 5-phosphate 4-kinase type-2 alpha	73.489	8.64	46152	3	2	0.66663557	0.289103	1	1
O70200	Aif1	Allograft inflammatory factor 1	194.268	21.77	16911	2	2	0.75021553	0.139945	1	3.4439099E-09
O70311	Nmt2	Glycylpeptide N-tetradecanoyltransfer ase 2	138.266	12.1	60484	5	2	1.08690138	0.107451	2.3172592E-05	1.6443972E-23
O70404	Vamp8	Vesicle-associated membrane protein 8	106.163	30.69	11451	3	2	0.73822574	0.189836	1	0.48689244
O88543	Cops3	COP9 signalosome complex subunit 3	188.945	25.3	47832	6	2	0.89932029	0.103945	4.3354917E-07	1.7060041E-26
O89086	Rbm3	RNA-binding protein 3	157.678	43.79	16605	5	2	0.65429422	0.047986	2.4158194E-66	1.0899968E-107
P00173	Cyb5a	Cytochrome b5	126.291	41.04	15355	4	2	1.16188844	0.027077	5.7675125E-142	5.0683403E-218
P00405	Mtco2	Cytochrome c oxidase subunit 2	186.311	40.53	25976	7	2	0.73539259	0.058177	5.3909408E-49	4.4819358E-84
P08207	S100a10	Protein S100-A10	70.036	27.84	11186	2	2	0.7634433	0.224396	1	0.99927289
P08905	Lyz2	Lysozyme C-2	74.868	16.22	16689	2	2	1.49982537	0.10076	8.8691289E-09	3.2112091E-29
P10649	Gstm1	Glutathione S-transferase Mu 1	172.837	32.57	25970	6	2	0.80917902	0.126248	0.11230398	1.6139892E-15
P11497	Acaca	Acetyl-CoA carboxylase 1	257.096	4.65	265194	7	2	1.13145975	0.051856	1.4402351E-59	2.0371644E-98
P11928	Oas1a	2'-5'-oligoadenylate synthase 1A	199.408	26.43	42429	6	2	1.28544556	0.087632	1.4770698E-16	1.1035589E-40
P17439	Gba	Glucosylceramidase	172.323	19.61	57622	5	2	0.73599209	0.093488	3.7315858E-11	7.1935142E-33
P21956	Mfge8	Lactadherin	107.297	12.31	51241	4	2	1.26826029	0.090111	9.0782127E-15	3.7042701E-38
P23780	Glb1	Beta-galactosidase	558.879	27.36	73121	14	2	0.81655468	0.052965	2.6653173E-56	5.6573634E-94
P25322	Ccnd1	G1/S-specific cyclin-D1	26.402	7.46	33429	2	2	0.8954447	0.136431	1	2.1943979E-11
P25799	Nfkb1	Nuclear factor NF-kappa-B p105 subunit	127.435	8.34	105615	7	2	1.14646492	0.059212	2.5349319E-48	3.5706725E-83
P26638	Sars	Serine--tRNA ligase, cytoplasmic	962.88	54.3	58389	23	2	0.91515324	0.153223	1	7.7586462E-05
P27546	Map4	Microtubule-associated protein 4	360.388	16.8	117429	13	2	0.90454064	0.044381	7.8471348E-72	2.6356981E-115
P28352	Apex1	DNA-(apurinic or apyrimidinic site) lyase	249.51	38.8	35490	8	2	0.81942804	0.092764	3.4723885E-12	2.0546485E-34

P28650	Adssl1	Adenylosuccinate synthetase isozyme 1	216.936	24.07	50254	7	2	0.72875248	0.07025	2.4064253E-36	2.6983753E-67
P28798	Grn	Granulins	274.292	20.54	63458	9	2	0.68462203	0.070807	6.9583173E-35	2.2215254E-65
P29416	Hexa	Beta-hexosaminidase subunit alpha	457.711	32.95	60613	14	2	0.82144242	0.025528	6.9382656E-148	3.2340587E-227
P40142	Tkt	Transketolase	1581.91	65.17	67630	37	2	0.8932322	0.03319	2.3220596E-116	2.7524967E-179
P42346	Mtor	Serine/threonine-protein kinase mTOR	86.655	2.28	288794	4	2	0.79122841	0.208456	1	0.96048335
P46462	Vcp	Transitional endoplasmic reticulum ATPase	1627.367	63.28	89349	43	2	0.906469	0.072513	3.6293945E-33	4.0160666E-63
P49300	Clec10a	C-type lectin domain family 10 member A	363.302	46.05	34596	10	2	0.86066962	0.062191	2.2424717E-44	6.4329304E-78
P50518	Atp6v1e1	V-type proton ATPase subunit E 1	214.308	36.73	26157	7	2	0.62141552	0.037231	6.5754376E-98	1.973636E-152
P51660	Hsd17b4	Peroxisomal multifunctional enzyme type 2	224.389	12.11	79482	6	2	1.32952612	0.095694	1.1757327E-10	4.1171246E-32
P51863	Atp6v0d1	V-type proton ATPase subunit d 1	379.805	36.75	40301	11	2	1.05751005	0.092341	2.9095214E-13	5.4546895E-36
P52480	Pkm	Pyruvate kinase PKM	3430.515	82.86	57845	65	2	0.93706156	0.06328	8.9293114E-43	8.6092819E-76
P56399	Usp5	Ubiquitin carboxyl-terminal hydrolase 5	937.149	46.39	95833	25	2	0.88043176	0.046786	1.1327569E-68	6.5695095E-111
P56480	Atp5f1b	ATP synthase subunit beta, mitochondrial	1842.881	74.86	56300	37	2	1.10401651	0.087495	9.216109E-17	5.6979792E-41
P57780	Actn4	Alpha-actinin-4	2225.24	66.01	104977	51	2	0.90060905	0.064741	3.3462001E-41	1.0426725E-73
P58021	Tm9sf2	Transmembrane 9 superfamily member 2	163.342	11.18	75330	5	2	1.16830776	0.07036	4.7399345E-36	6.5530337E-67
P58242	Smpdl3b	Acid sphingomyelinase-like phosphodiesterase 3b	120.72	14.69	51600	4	2	0.78076173	0.146939	1	3.5547603E-07
P61021	Rab5b	Ras-related protein Rab-5B	133.178	21.4	23707	4	2	1.0698975	0.059777	2.5390744E-47	7.7378331E-82
P61078	Ube2d3	Ubiquitin-conjugating enzyme E2 D3	106.186	40.82	16687	4	2	0.80895027	0.090385	1.4144161E-14	6.9777465E-38
P61589	Rhoa	Transforming protein RhoA	508.556	64.77	21782	11	2	0.79270649	0.033895	2.0919094E-114	2.0670387E-176
P61924	Copz1	Coatomer subunit zeta-1	149.625	21.47	20198	3	2	0.76061284	0.11809	0.017341406	1.1764219E-17

P62305	Snrpe	Small nuclear ribonucleoprotein E	145.962	39.13	10804	3	2	0.75332173	0.053425	8.1433112E-54	1.3700268E-90
P62307	Snrpf	Small nuclear ribonucleoprotein F	87.878	33.72	9725	2	2	0.5365205	0.147285	1	1.6383435E-06
P62329	Tmsb4x	Thymosin beta-4	113.415	79.55	5053	5	2	0.67167925	0.11002	0.00010575655	2.6902806E-22
P62331	Arf6	ADP-ribosylation factor 6	304.954	65.71	20082	7	2	0.86317027	0.029465	6.4068323E-130	1.3440194E-199
P62482	Kcnab2	Voltage-gated potassium channel subunit beta-2	87.277	15.8	41021	4	2	0.85491305	0.091781	1.2440624E-13	1.5926019E-36
P62804	Hist1h4b	Histone H4	791.118	58.25	11367	16	2	1.11630314	0.061315	5.0506607E-45	8.8701775E-79
P62849	Rps24	40S ribosomal protein S24	122.541	20.3	15423	3	2	1.32480292	0.059966	5.4472227E-47	2.1417385E-81
P62959	Hint1	Histidine triad nucleotide-binding protein 1	96.715	58.73	13777	5	2	0.80781883	0.037174	2.7983609E-99	2.081069E-154
P63036	Dnaja1	DnaJ homolog subfamily A member 1	114.421	14.36	44868	3	2	1.44678892	0.039645	7.4472755E-90	7.6261736E-141
P63323	Rps12	40S ribosomal protein S12	225.662	45.45	14525	6	2	0.87063972	0.044023	4.9437267E-73	5.4525947E-117
P68033	Actc1	Actin, alpha cardiac muscle 1	1232.137	40.58	42019	30	2	1.06671008	0.085321	1.9145929E-18	2.6216228E-43
P68181	Prkacb	cAMP-dependent protein kinase catalytic subunit beta	264.71	23.65	40708	8	2	1.08359375	0.036444	2.2406741E-104	9.182561E-162
P70195	Psmb7	Proteasome subunit beta type-7	53.644	29.96	29891	3	2	0.68504037	0.076598	1.1005108E-24	7.3677029E-52
P70296	Pebp1	Phosphatidylethanolamine-binding protein 1	253.167	62.03	20830	6	2	0.82429493	0.087462	5.7342593E-17	2.9358681E-41
P70333	Hnrnph2	Heterogeneous nuclear ribonucleoprotein H2	330.103	25.17	49280	8	2	0.90987908	0.092569	4.4303E-13	1.0049399E-35
P80315	Cct4	T-complex protein 1 subunit delta	953.883	60.48	58066	27	2	1.0939328	0.076598	6.2246662E-25	3.4029384E-52
P81117	Nucb2	Nucleobindin-2	156.708	12.38	50304	4	2	1.34450548	0.092764	7.7529233E-12	6.7717256E-34
P83868	Ptges3	Prostaglandin E synthase 3	136.366	26.25	18721	3	2	0.78520099	0.165359	1	0.018045591
P98078	Dab2	Disabled homolog 2	586.553	34.6	82312	19	2	0.94979695	0.123806	0.058303623	2.5830734E-16
Q05921	Rnasel	2-5A-dependent ribonuclease	45.813	4.63	83275	2	2	0.68372141	0.04817	5.8588766E-66	3.6984078E-107

Q07984	Ssr4	Translocon-associated protein subunit delta	153.742	30.64	18980	4	2	1.24763676	0.07505	2.9703175E-28	1.1818335E-56
Q2PQA9	Kif5b	Kinesin-1 heavy chain	506.963	21.39	109531	16	2	0.89517316	0.152364	1	1.8166859E-05
Q3THG9	Aarsd1	Alanyl-tRNA editing protein Aarsd1	47.018	6.55	44971	2	2	1.20956357	0.118235	0.020404482	1.7476749E-17
Q3TL44	Nlrx1	NLR family member X1	96.774	7.18	107831	4	2	0.84141849	0.192235	1	0.60045065
Q3TLH4	Prrc2c	Protein PRRC2C	39.129	0.91	310892	2	2	0.78473813	0.189019	1	0.47052648
Q4VSI4	Usp7	Ubiquitin carboxyl-terminal hydrolase 7	111.74	7.62	128431	5	2	0.83259159	0.067378	7.8188716E-38	2.9548286E-69
Q5M7W1	Txnip	Thioredoxin-interacting protein	103.107	7.11	44018	3	2	0.63245578	0.065265	1.4016107E-40	6.9246368E-73
Q5PPJ9	Sh3glb2	Endophilin-B2	17.669	5.69	44853	2	2	0.82383189	0.048897	8.2453004E-65	1.4082176E-105
Q5RJQ8	Calhm2	Calcium homeostasis modulator protein 2	80.793	9.6	35931	3	2	0.58292436	0.062232	2.0603214E-43	1.223984E-76
Q5XI31	Pigs	GPI transamidase component PIG-S	251.113	17.84	61724	6	2	1.14419964	0.075848	1.8043702E-27	1.3120875E-55
Q5XIG8	Strap	Serine-threonine kinase receptor-associated protein	433.385	52.86	38456	12	2	1.09582414	0.053547	1.8271251E-53	4.1219577E-90
Q60631	Grb2	Growth factor receptor-bound protein 2	219.982	46.54	25238	8	2	1.06697543	0.096385	2.4898353E-10	1.2903167E-31
Q61029	Tmpo	Lamina-associated polypeptide 2, isoforms beta/delta/epsilon/gamma	868.001	53.98	50373	17	2	0.90564175	0.038313	4.6182866E-92	5.3218676E-144
Q61164	Ctcf	Transcriptional repressor CTCF	76.872	4.76	83745	3	2	0.83445233	0.076598	3.511455E-25	1.5689124E-52
Q61390	Cct6b	T-complex protein 1 subunit zeta-2	159.704	11.11	58185	5	2	1.13789346	0.064561	1.6290028E-41	4.0249258E-74
Q61599	Arhgdib	Rho GDP-dissociation inhibitor 2	626.533	76.5	22851	15	2	0.79328312	0.042823	2.8200199E-76	1.4629318E-121
Q61820	Rasl2-9	GTP-binding nuclear protein Ran, testis-specific isoform	154.659	25	24452	6	2	0.93928663	0.138271	1	1.1784911E-09
Q62159	Rhoc	Rho-related GTP-binding protein RhoC	544.859	65.8	22006	11	2	0.80086951	0.040441	3.1880011E-84	8.1666616E-133
Q64105	Spr	Sepiapterin reductase	408.648	41.76	27883	8	2	0.8595088	0.052133	4.1740932E-58	1.9842493E-96

Q64378	Fkbp5	Peptidyl-prolyl cis-trans isomerase FKBP5	145.766	17.11	50966	6	2	1.18442696	0.030558	2.5537809E-124	3.860205E-191
Q6AYK3	Isyna1	Inositol-3-phosphate synthase 1	209.782	17.59	60884	7	2	0.8897299	0.139945	1	4.4768134E-09
Q6P7S1	Asah1	Acid ceramidase	149.889	12.44	44443	5	2	0.83315493	0.089604	2.3597082E-15	5.4628121E-39
Q6PB66	Lrpprc	Leucine-rich PPR motif-containing protein, mitochondrial	863.426	30.24	156615	29	2	1.18442336	0.022823	2.9819556E-166	2.6470113E-256
Q6PB93	Galnt2	Polypeptide N-acetylgalactosaminyltr ansferase 2	49.663	5.79	64514	3	2	1.39771173	0.110141	0.00021653683	1.0513923E-21
Q6PCN7	Hltf	Helicase-like transcription factor	55.908	2.69	113317	2	2	0.62094951	0.123607	0.050660747	1.7762081E-16
Q6PFQ7	Rasa4	Ras GTPase-activating protein 4	83.052	3.87	90060	3	2	1.20409818	0.097788	7.5041231E-10	7.003024E-31
Q6ZPF4	Fmnl3	Formin-like protein 3	410.717	14.4	117169	10	2	0.85941368	0.041279	2.0421558E-80	2.0758825E-127
Q6ZQA0	Nbeal2	Neurobeachin-like protein 2	119.261	1.5	301924	3	2	0.63781525	0.147285	1	2.0244086E-06
Q78IK4	Apool	MICOS complex subunit Mic27	230.272	26.42	29261	5	2	0.91885593	0.074914	7.5045031E-30	9.0018381E-59
Q792H5	Celf2	CUGBP Elav-like family member 2	155.568	10.24	54271	5	2	0.72013152	0.136169	1	1.2050884E-11
Q7M6Y3	Picalm	Phosphatidylinositol-binding clathrin assembly protein	553.799	26.52	71543	12	2	0.86662717	0.066497	3.9107755E-38	1.1797312E-69
Q80UM7	Mogs	Mannosyl-oligosaccharide glucosidase	495.036	21.82	91831	11	2	1.15682727	0.07613	1.9756461E-25	7.2024719E-53
Q80XQ2	Tbc1d5	TBC1 domain family member 5	48.421	3.07	91837	2	2	0.84196856	0.034493	5.0966938E-111	1.9412948E-171
Q810A7	Ddx42	ATP-dependent RNA helicase DDX42	69.43	3.55	101965	2	2	0.59999449	0.092764	1.5372021E-12	6.1849248E-35
Q8BFR4	Gns	N-acetylglucosamine-6-sulfatase	106.863	16.36	61175	7	2	0.68880793	0.079773	4.8686571E-22	2.9793769E-48
Q8BMG7	Rab3gap2	Rab3 GTPase-activating protein non-catalytic subunit	136.472	7.69	152535	8	2	1.09729127	0.089671	3.7079931E-15	1.0367139E-38
Q8BMJ2	Lars	Leucine--tRNA ligase, cytoplasmic	855.886	30.14	134192	26	2	1.11006931	0.063945	3.8325151E-42	5.9270325E-75

Q8BRN9	Cc2d1b	Coiled-coil and C2 domain-containing protein 1B	135.932	6.96	93091	4	2	0.70361393	0.110118	0.00013471571	4.2462474E-22
Q8BT60	Cpne3	Copine-3	129.342	10.32	59585	4	2	1.08698153	0.100256	2.2018264E-09	3.6676415E-30
Q8BXQ2	Pigt	GPI transamidase component PIG-T	62.931	7.73	65705	3	2	1.40501471	0.099964	1.5425983E-09	2.1197883E-30
Q8BYW1	Arhgap25	Rho GTPase-activating protein 25	346.993	21.91	73383	10	2	0.84144811	0.034907	1.2584752E-108	6.1111531E-168
Q8C0L6	Paox	Peroxisomal N(1)-acetyl-spermine/spermidine oxidase	163.299	11.31	55447	5	2	0.73234132	0.137931	1	2.2370707E-10
Q8C2E7	Washc5	WASH complex subunit 5	200.055	8.54	134110	7	2	0.79727184	0.074876	1.7280313E-31	6.3472524E-61
Q8C3J5	Dock2	Dedicator of cytokinesis protein 2	1416.391	27.79	211704	40	2	0.91260321	0.079773	8.335705E-22	6.2159761E-48
Q8CB77	Eloa	Elongin-A	63.24	5.43	87161	2	2	0.76883239	0.084156	1.553897E-19	8.2544104E-45
Q8CIH5	Plcg2	1-phosphatidylinositol 4,5-bisphosphate phosphodiesterase gamma-2	681.111	21.5	147592	18	2	0.91856938	0.118444	0.027981648	3.8338037E-17
Q8K482	Emilin2	EMILIN-2	48.044	3.72	117310	2	2	0.80207893	0.062191	9.8608747E-44	4.5939435E-77
Q8R050	Gspt1	Eukaryotic peptide chain release factor GTP-binding subunit ERF3A	258.743	20.75	68625	9	2	0.83593929	0.108133	3.8922651E-05	4.222301E-23
Q8VDQ8	Sirt2	NAD-dependent protein deacetylase sirtuin-2	92.204	9.51	43256	2	2	0.85678487	0.121235	0.032612376	5.6610485E-17
Q8VE99	Ccdc115	Coiled-coil domain-containing protein 115	70.489	22.78	19743	2	2	0.7700426	0.04313	7.7415587E-74	3.9888646E-118
Q8VHR5	Gatad2b	Transcriptional repressor p66-beta	34.843	5.89	65411	2	2	0.72940316	0.059338	1.1807009E-47	2.782128E-82
Q91VW3	Sh3bgrl3	SH3 domain-binding glutamic acid-rich-like protein 3	104.967	55.91	10477	4	2	0.73807937	0.099964	1.0775178E-09	1.2228496E-30
Q91W89	Man2c1	Alpha-mannosidase 2C1	128.331	5.29	115688	4	2	0.88010849	0.090385	2.1974319E-14	1.3102678E-37
Q920A5	Scpep1	Retinoid-inducible serine carboxypeptidase	437.629	27.43	50965	14	2	0.74162049	0.02849	1.1315663E-136	7.2187505E-210
Q922Q8	Lrrc59	Leucine-rich repeat-containing protein 59	337.106	39.74	34877	9	2	1.16991326	0.034931	3.7643659E-108	3.0258852E-167

Q922U1	Prpf3	U4/U6 small nuclear ribonucleoprotein Prp3	89.173	12.3	77455	5	2	0.81992845	0.075971	3.4633657E-26	6.8724141E-54
Q99K23	Ufsp2	Ufm1-specific protease 2	49.549	6.29	52515	3	2	1.22234498	0.174751	1	0.13218977
Q99KP6	Prpf19	Pre-mRNA-processing factor 19	539.892	46.23	55239	14	2	0.89472935	0.048708	3.4228386E-65	4.204158E-106
Q99NB9	Sf3b1	Splicing factor 3B subunit 1	1219.249	29.6	145816	30	2	0.92605133	0.048496	1.4177142E-65	1.2500461E-106
Q9CPQ3	Tomm22	Mitochondrial import receptor subunit TOM22 homolog	166.177	30.99	15537	3	2	0.84959129	0.106549	7.911033E-06	2.4269756E-24
Q9CPU4	Mgst3	Microsomal glutathione S-transferase 3	204.663	49.67	16958	4	2	0.82892828	0.046721	1.8623872E-69	5.3952927E-112
Q9CYG7	Tomm34	Mitochondrial import receptor subunit TOM34	221.466	24.27	34278	5	2	0.83073798	0.074914	2.1570391E-30	1.7409822E-59
Q9CZN7	Shmt2	Serine hydroxymethyltransferase, mitochondrial	867.461	60.52	55759	23	2	1.14247857	0.031688	2.6172856E-121	1.2630835E-186
Q9D662	Sec23b	Protein transport protein Sec23B	641.768	33.25	86437	15	2	0.84238765	0.08503	7.0697795E-19	6.6301025E-44
Q9D7N9	Apmap	Adipocyte plasma membrane-associated protein	144.322	16.39	46434	4	2	1.31112896	0.190704	1	0.56842729
Q9D880	Timm50	Mitochondrial import inner membrane translocase subunit TIM50	274.972	18.41	39776	5	2	0.93811362	0.089002	5.9791677E-16	7.864149E-40
Q9DAS9	Gng12	Guanine nucleotide-binding protein G(I)/G(S)/G(O) subunit gamma-12	95.198	37.5	7997	2	2	0.84425691	0.118235	0.023932505	2.5907287E-17
Q9DBG5	Plin3	Perilipin-3	782.967	66.82	47262	19	2	0.70341271	0.092764	5.1961352E-12	3.7342867E-34
Q9DCD0	Pgd	6-phosphogluconate dehydrogenase, decarboxylating	927.981	65.63	53247	26	2	0.91605362	0.074914	1.151997E-30	7.6328387E-60
Q9JIK9	Mrps34	28S ribosomal protein S34, mitochondrial	30.133	10.55	25827	3	2	1.1374721	0.057716	5.1939012E-50	1.9428047E-85
Q9QWT9	Kifc1	Kinesin-like protein KIFC1	85.723	8.46	74153	4	2	0.79207809	0.12864	0.20017377	9.5272872E-15

Q9QYR9	Acot2	Acyl-coenzyme A thioesterase 2, mitochondrial	162.512	13.25	49657	4	2	0.82923186	0.08022	1.4233176E-21	1.2945045E-47
Q9R1P3	Psmb2	Proteasome subunit beta type-2	225.423	55.22	22906	7	2	0.83269293	0.053031	7.1093446E-55	4.9333942E-92
Q9WUM4	Coro1c	Coronin-1C	654.857	39.87	53121	16	2	0.89271048	0.052632	2.2163597E-57	1.9289042E-95
Q9WVA3	Bub3	Mitotic checkpoint protein BUB3	206.598	23.62	36955	6	2	0.92114478	0.115695	0.0050806949	6.8741582E-19
Q9WVJ2	Psmd13	26S proteasome non-ATPase regulatory subunit 13	718.896	59.31	42809	18	2	1.13494173	0.051189	8.6187346E-62	1.9157601E-101
Q9Z0S1	Bpnt1	3'(2'),5'-bisphosphate nucleotidase 1	96.983	13.64	33196	3	2	0.83278039	0.090385	3.4041842E-14	2.4558208E-37
Q9Z2W0	Dnpep	Aspartyl aminopeptidase	583.343	48.2	52207	13	2	0.87595055	0.078053	1.0470452E-23	1.5751407E-50
