# Supplementary material for: Repeated Exposure of Macrophages to Synthetic Amorphous Silica Induces Adaptive Proteome Changes and a Moderate Cell Activation
Source: Nanomaterials (Basel). 2022 Apr 22;12(9):1424. doi: 10.3390/nano12091424 (PMC9105884; doi:10.3390/nano12091424)
Supplement: Supplementary file 1 [file nanomaterials-12-01424-s001.zip › Table S3.pdf]

Table S3: list of proteins modulated in response to treatment with fumed silica

Coverage: % coverage of the protein sequence by the peptides identified in the mass spectrometry analysis
#peptides: number of peptides used for protein identification
U Fu vs ctrl: result of the Mann Whitney U test in the comparison control vs treated with fumed silica
B-H: evaluation of the false positive rate by the Benjamini-Hochberg method
SGoF+ : evaluation of the false positive rate by the Sequential Goodness of Fit method
Sfisher : : evaluation of the false positive rate by the Sequential Fisher test method

accession number	gene_name	description	protein_set_score	coverage	MW	#peptides	U Fu vs ctrl	ratio Fu/ctrl	B-H	SGoF+	Sfisher
A2RSY6	Trmt1l	TRMT1-like protein	90.625	4.4	80861	2	0	0.825740987	0.23128573	1.61254E-24	4.1071E-32
D3ZCL3	Snrpc	U1 small nuclear ribonucleoprotein C	61.28	16.98	17364	2	0	0.625453666	0.13358441	1.07272E-52	1.50246E-86
O08528	Hk2	Hexokinase-2	826.505	39.04	102535	30	0	0.908565234	0.28507094	3.80878E-10	1.98838E-12
O08553	Dpysl2	Dihydropyrimidinase-related protein 2	1630.64	79.2	62278	33	0	0.893579019	0.079171354	1.45742E-53	4.19836E-89
O08997	Atox1	Copper transport protein ATOX1	124.127	58.82	7338	4	0	0.746070005	0.30513796	0.00367379	0.000359576
O09159	Man2b1	Lysosomal alpha-mannosidase	557.178	24.38	114648	17	0	0.874440359	0.20737785	5.12043E-39	1.96722E-56
O35691	Pnn	Pinin	55.561	3.17	82436	2	0	0.541080378	0.0235938	6.57984E-56	1.48736E-97
O35855	Bcat2	Branched-chain-amino-acid aminotransferase, mitochondrial	337.251	36.64	44127	10	0	0.914033063	0.25788439	1.89868E-16	1.48269E-20
O54734	Ddost	Dolichyl-diphosphooligosaccharide--protein glycosyltransferase 48 kDa subunit	568.017	50.57	49028	15	0	1.08875869	0.21730432	2.0596E-28	3.38859E-38
O55143	Atp2a2	Sarcoplasmic/endoplasmic reticulum calcium ATPase 2	625.662	20.69	114858	16	0	0.913351432	0.23103754	1.02063E-25	6.06881E-34
O70152	Dpm1	Dolichol-phosphate mannosyltransferase subunit 1	34.675	7.31	29175	2	0	1.584323185	0.3363215	1	0.91986959
P01899	H2-D1	H-2 class I histocompatibility antigen, D-B alpha chain	255.44	28.45	40836	9	0	1.126852296	0.26627283	4.97035E-15	1.13625E-18
P01902	H2-K1	H-2 class I histocompatibility antigen, K-D alpha chain	345.838	33.15	41490	11	0	1.164794807	0.21730432	9.72367E-34	6.03285E-47
P05063	Aldoc	Fructose-bisphosphate aldolase C	1174.329	80.17	39395	30	0	0.897673154	0.054065425	9.93139E-55	5.86034E-93
P05064	Aldoa	Fructose-bisphosphate aldolase A	1657.605	92.31	39356	40	0	0.917053025	0.19007698	8.4882E-42	8.62523E-62
P06745	Gpi	Glucose-6-phosphate isomerase	1529.065	69.53	62767	39	0	0.956501712	0.31817293	0.61475177	0.16266973
P08113	Hsp90b1	Endoplasmin	1785.373	54.49	92476	49	0	1.033435631	0.21730432	3.33874E-34	9.53284E-48
P08752	Gnai2	Guanine nucleotide-binding protein G(i) subunit alpha-2	1256.755	69.86	40489	26	0	0.89378071	0.19007698	7.84297E-43	7.17829E-64
P08775	Polr2a	DNA-directed RNA polymerase II subunit RPB1	350.364	7.41	217176	10	0	0.865504602	0.25788439	4.23775E-17	1.94236E-21
P09528	Fth1	Ferritin heavy chain	222.436	41.21	21067	6	0	1.285977137	0.075061067	1.94894E-54	6.31071E-92
P10711	Tcea1	Transcription elongation factor A protein 1	175.251	20.93	33880	4	0	0.448016733	0.21319152	2.23498E-35	8.4609E-50
P11598	Pdia3	Protein disulfide-isomerase A3	945.818	52.28	56623	26	0	1.078688558	0.19007698	2.2946E-47	2.43873E-73
P11835	Itgb2	Integrin beta-2	1205.242	41.76	85026	25	0	0.871278553	0.19007698	1.29006E-43	1.82672E-65
P13020	Gsn	Gelsolin	1819.541	61.03	85942	44	0	0.906421704	0.19007698	3.83853E-44	1.50279E-66
P14069	S100a6	Protein S100-A6	189.18	78.65	10051	6	0	0.766567122	0.19007698	1.22202E-47	6.22557E-74
P14152	Mdh1	Malate dehydrogenase, cytoplasmic	618.435	47.6	36511	13	0	0.929614466	0.19007698	4.69237E-42	2.63898E-62
P16460	Ass1	Argininosuccinate synthase	229.08	26.21	46584	10	0	1.408366188	0.19007698	1.42635E-42	2.40972E-63

P17742	Ppia	Peptidyl-prolyl cis-trans isomerase A	1008.804	71.34	17971	20	0	0.899337986	0.0235938	3.3236E-56	7.62619E-99
P21575	Dnm1	Dynamin-1	42.89	3.94	97295	4	0	1.097507022	0.20548165	1.6246E-39	2.25866E-57
P24051	Rps27l	40S ribosomal protein S27-like	146.25	25	9477	3	0	0.826258334	0.048095791	5.05192E-55	4.98947E-94
P27867	Sord	Sorbitol dehydrogenase	47.078	7.28	38235	2	0	0.891187628	0.13937684	1.49845E-51	1.91913E-83
P28798	Grn	Granulins	274.292	20.54	63458	9	0	0.61930017	0.19007698	1.50305E-46	1.36729E-71
P29391	Ftl1	Ferritin light chain 1	365.894	55.74	20802	7	0	1.33053224	0.26575971	5.73344E-16	6.54364E-20
P30681	Hmgb2	High mobility group protein B2	399.936	29.05	24162	10	0	0.852623927	0.14885417	1.06284E-50	3.21073E-81
P32020	Scp2	Non-specific lipid-transfer protein	78.683	5.3	59126	3	0	0.788356734	0.19007698	2.76273E-41	9.06199E-61
P34884	Mif	Macrophage migration inhibitory factor	364.546	71.3	12504	8	0	0.721635942	0.19007698	2.3587E-43	6.28867E-65
P34960	Mmp12	Macrophage metalloelastase	124.013	12.26	54971	5	0	1.539224247	0.19007698	1.82635E-48	8.92547E-76
P35700	Prdx1	Peroxiredoxin-1	593.265	64.82	22176	20	0	1.112919429	0.19007698	2.80234E-46	5.09651E-71
P42667	Sec11a	Signal peptidase complex catalytic subunit SEC11A	70.665	16.2	20599	4	0	1.138299919	0.19007698	6.49656E-48	1.53694E-74
P47791	Gsr	Glutathione reductase, mitochondrial	264.542	25.8	53663	7	0	0.934307942	0.19007698	2.08826E-44	4.24836E-67
P48036	Anxa5	Annexin A5	1571.408	83.39	35752	38	0	1.129761056	0.13141194	2.83999E-53	3.42738E-88
P50543	S100a11	Protein S100-A11	164.439	71.43	11083	4	0	0.773037747	0.20737785	2.4875E-36	1.66111E-51
P51859	Hdgf	Hepatoma-derived growth factor	165.045	31.22	26269	7	0	0.846730763	0.25546543	4.2253E-18	8.25101E-23
P54822	Adsl	Adenylosuccinate lyase	453.506	35.12	54866	11	0	0.896985899	0.30059583	8.3726E-06	3.18165E-07
P55302	Lrpap1	Alpha-2-macroglobulin receptor-associated protein	230.143	22.5	42215	7	0	1.182569906	0.14885417	7.4198E-50	4.10885E-79
P59325	Eif5	Eukaryotic translation initiation factor 5	111.347	7.46	48968	4	0	0.888638808	0.21730432	3.87268E-32	3.07881E-44
P60335	Pcbp1	Poly(rC)-binding protein 1	636.305	63.48	37498	14	0	0.8335091	0.19007698	2.58938E-42	8.01417E-63
P60766	Cdc42	Cell division control protein 42 homolog	384.278	58.12	21259	9	0	0.821026933	0.21730432	1.95112E-34	3.75513E-48
P60843	Eif4a1	Eukaryotic initiation factor 4A-I	1018.101	54.93	46154	24	0	1.085676355	0.3759886	1	0.99998262
P60867	Rps20	40S ribosomal protein S20	130.94	28.57	13373	4	0	0.772099227	0.022124067	4.2385E-57	5.3311E-103
P62774	Mtpn	Myotrophin	279.736	75.42	12861	5	0	0.749382254	0.26627283	7.07726E-15	1.80937E-18
P62830	Rpl23	60S ribosomal protein L23	191.96	50	14865	7	0	0.915340671	0.14885417	1.41308E-49	2.02197E-78
P62869	Elob	Elongin-B	274.104	59.32	13170	7	0	0.851529511	0.20548165	5.11777E-40	2.53701E-58
P63323	Rps12	40S ribosomal protein S12	225.662	45.45	14525	6	0	0.8063337	0.079171354	7.46598E-54	5.07238E-90
P68033	Actc1	Actin, alpha cardiac muscle 1	1232.137	40.58	42019	30	0	1.1038074	0.19007698	6.14763E-45	3.33583E-68
P70340	Smad1	Mothers against decapentaplegic homolog 1	42.288	8.17	52157	2	0	0.635624247	0.30513796	0.000231415	1.48166E-05
P97429	Anxa4	Annexin A4	866.33	67.71	35916	20	0	1.185125351	0.20548165	8.92814E-41	8.9825E-60
P97821	Ctsc	Dipeptidyl peptidase 1	238.605	18.18	52376	7	0	0.798001626	0.18417611	5.09813E-49	4.71151E-77
P97874	Gak	Cyclin-G-associated kinase	63.416	2.91	143703	2	0	1.267689151	0.20737785	1.53577E-37	1.06403E-53
Q01205	Dlst	Dihydrolipoyllysine-residue succinyltransferase component of 2-oxoglutarate dehydrogenase complex, mitochondrial	264.324	19.16	48925	8	0	1.153323641	0.20737785	2.69012E-37	2.96102E-53
Q04750	Top1	DNA topoisomerase 1	324.79	22.69	90876	14	0	0.836048306	0.25788439	1.3087E-16	8.96325E-21
Q04899	Cdk18	Cyclin-dependent kinase 18	112.94	10.64	51848	4	0	1.404300606	0.13937684	4.02343E-52	5.76702E-85
Q04931	Ssrp1	FACT complex subunit SSRP1	364.482	25.81	80914	12	0	0.89580918	0.24413627	2.27351E-20	5.21678E-26
Q3UQ44	Iqgap2	Ras GTPase-activating-like protein IQGAP2	68.925	2.16	180528	4	0	1.161738739	0.13937684	2.88415E-51	1.08847E-82
Q3UW53	Fam129a	Protein Niban	737.731	34.99	102649	25	0	0.936900574	0.14885417	3.88912E-50	8.26178E-80
Q501J6	Ddx17	Probable ATP-dependent RNA helicase DDX17	518.414	21.23	72399	11	0	0.886215268	0.047137948	2.56531E-55	3.99693E-95
Q5FWK3	Arhgap1	Rho GTPase-activating protein 1	222.831	31.89	50411	9	0	0.698145128	0.22950591	6.40192E-26	2.94971E-34

Q60631	Grb2	Growth factor receptor-bound protein 2	219.982	46.54	25238	8	0	1.093551069	0.15367011	2.68641E-49	9.88764E-78
Q61191	Hcfc1	Host cell factor 1	498.608	10.12	210437	13	0	0.88861105	0.21730432	2.8683E-29	1.43925E-39
Q61207	Psap	Prosaposin	294.177	14.54	61422	7	0	0.787122054	0.30547343	0.10598378	0.018694137
Q61210	Arhgef1	Rho guanine nucleotide exchange factor 1	557.637	26.09	102805	17	0	0.915925148	0.20737785	8.20969E-37	2.25147E-52
Q61749	Eif2b4	Translation initiation factor eIF-2B subunit delta	64.619	7.25	57624	2	0	0.431785992	0.32376711	1	0.53526425
Q62193	Rpa2	Replication protein A 32 kDa subunit	79.469	13.7	29718	2	0	0.656031223	0.022124067	8.43542E-57	1.4591E-101
Q64514	Tpp2	Tripeptidyl-peptidase 2	740.218	25.91	139879	24	0	0.931966117	0.24413627	1.83456E-21	1.40837E-27
Q64620	Ppp6c	Serine/threonine-protein phosphatase 6 catalytic subunit	117.876	19.02	35159	5	0	0.884688172	0.20737785	4.31829E-36	4.4772E-51
Q68FD5	Cltc	Clathrin heavy chain 1	4079.117	65.73	191557	96	0	0.921387776	0.21730432	8.86351E-28	3.50657E-37
Q6IMY8	Hnrnpu	Heterogeneous nuclear ribonucleoprotein U	1237.314	46.87	87732	34	0	0.919004705	0.24163074	2.14046E-22	6.14911E-29
Q6NWW9	Fndc3b	Fibronectin type III domain-containing protein 3B	80.396	3.31	132764	3	0	0.751618241	0.2700068	4.24535E-13	3.71908E-16
Q6P549	Inppl1	Phosphatidylinositol 3,4,5-trisphosphate 5-phosphatase 2	207.803	7.4	138973	6	0	1.102664536	0.21730432	1.13816E-34	1.47371E-48
Q6TUG0	Dnajb11	DnaJ homolog subfamily B member 11	290.148	30.17	40495	7	0	1.143332857	0.23103754	1.62416E-25	1.2399E-33
Q6ZQ38	Cand1	Cullin-associated NEDD8-dissociated protein 1	877.807	33.66	136332	29	0	0.962876329	0.29414201	5.00468E-08	7.47404E-10
Q78P75	Dynll2	Dynein light chain 2, cytoplasmic	86.955	25.84	10350	2	0	0.719928788	0.19007698	3.32669E-45	9.25185E-69
Q792H5	Celf2	CUGBP Elav-like family member 2	155.568	10.24	54271	5	0	0.836640115	0.19007698	5.21555E-46	1.89112E-70
Q7TPJ0	Ssr1	Translocon-associated protein subunit alpha	59.898	10.66	35629	3	0	1.196385887	0.33798703	1	0.95198661
Q8BH61	F13a1	Coagulation factor XIII A chain	164.935	14.48	83207	9	0	0.562158101	0.30513796	0.000161183	9.77704E-06
Q8BHN3	Ganab	Neutral alpha-glucosidase AB	999.162	43.64	106911	31	0	1.077496556	0.13937684	7.77146E-52	3.35941E-84
Q8BK67	Rcc2	Protein RCC2	650.406	48.27	55983	19	0	0.859099982	0.19007698	4.3049E-43	2.13036E-64
Q8BP48	Metap1	Methionine aminopeptidase 1	116.45	12.95	43221	3	0	0.62447758	0.21730432	1.05879E-29	2.8999E-40
Q8BPU7	Elmo1	Engulfment and cell motility protein 1	416.403	28.61	83936	16	0	0.767226417	0.21730432	6.6274E-35	5.75061E-49
Q8BSY0	Asph	Aspartyl/asparaginyl beta-hydroxylase	677.992	35.76	83042	22	0	1.096959946	0.20548165	2.86473E-40	8.41248E-59
Q8BTI8	Srrm2	Serine/arginine repetitive matrix protein 2	362.852	6.4	294840	11	0	0.567063723	0.13141194	5.52438E-53	2.29869E-87
Q8BVF2	Pdcl3	Phosducin-like protein 3	109.738	18.33	27581	3	0	0.806282156	0.21730432	2.81154E-33	3.695E-46
Q8K0F1	Tbc1d23	TBC1 domain family member 23	84.48	7.02	76426	2	0	0.694888001	0.14885417	5.54148E-51	6.06013E-82
Q8QZY1	Eif3l	Eukaryotic translation initiation factor 3 subunit L	688.491	35.64	66613	16	0	1.108691595	0.24163074	5.08299E-22	2.1795E-28
Q8VCN5	Cth	Cystathionine gamma-lyase	349.244	29.9	43567	8	0	1.332107743	0.079171354	3.81791E-54	5.84577E-91
Q91YR9	Ptgr1	Prostaglandin reductase 1	329.123	36.47	35560	9	0	0.74835585	0.14885417	2.0349E-50	1.64629E-80
Q924Z4	Cers2	Ceramide synthase 2	118.544	11.84	45024	3	0	1.27841818	0.36932998	1	0.99986935
Q99KP3	Cryl1	Lambda-crystallin homolog	80.371	14.42	35209	3	0	0.884253083	0.25425995	2.85856E-18	4.80724E-23
Q99PD4	Arpc1a	Actin-related protein 2/3 complex subunit 1A	94.403	9.46	41599	3	0	0.729192917	0.20737785	4.7037E-37	8.18201E-53
Q9CPU0	Glo1	Lactoylglutathione lyase	134.222	36.96	20810	6	0	0.914009045	0.20548165	4.97092E-41	2.90438E-60
Q9CQW9	Ifitm3	Interferon-induced transmembrane protein 3	116.3	36.5	14954	3	0	1.260239649	0.21730432	4.7679E-33	9.03159E-46
Q9CZU6	Cs	Citrate synthase, mitochondrial	529.968	37.93	51737	16	0	1.057230626	0.20548165	1.6007E-40	2.76753E-59
Q9D0F6	Rfc5	Replication factor C subunit 5	98.822	15.63	38096	3	0	0.851420698	0.21730432	3.35621E-28	7.40925E-38
Q9D0G0	Mrps30	28S ribosomal protein S30, mitochondrial	101.961	9.05	49939	2	0	0.771268276	0.24413627	1.76023E-19	9.57315E-25
Q9D2V7	Coro7	Coronin-7	315.235	17.25	100812	9	0	0.895847579	0.033029055	1.30035E-55	2.67466E-96
Q9DAR7	Dcps	m7GpppX diphosphatase	396.432	47.93	38988	11	0	0.915625723	0.24413627	1.50145E-20	2.88368E-26
Q9DBR3	Armc8	Armadillo repeat-containing protein 8	79.415	9.81	75364	4	0	0.851557407	0.20737785	2.82694E-38	4.73503E-55
Q9DCL9	Paics	Multifunctional protein ADE2	428.915	45.41	47006	13	0	0.883671184	0.30513796	0.016393867	0.002052137

Q9DCX2	Atp5pd	ATP synthase subunit d, mitochondrial	201.244	54.04	18749	6	0	0.892459328	0.19007698	7.04325E-44	5.26919E-66
Q9EPB4	Pycard	Apoptosis-associated speck-like protein containing a CARD	197.84	36.79	21459	6	0	0.841254737	0.26627283	3.48412E-15	7.11934E-19
Q9EQ32	Pik3ap1	Phosphoinositide 3-kinase adapter protein 1	749.784	26.63	90928	18	0	0.859744459	0.19007698	1.13405E-44	1.19358E-67
Q9ESY9	Ifi30	Gamma-interferon-inducible lysosomal thiol reductase	195.149	20.97	27784	6	0	0.677976041	0.0235938	1.67586E-56	3.8105E-100
Q9JHS3	Lamtor2	Ragulator complex protein LAMTOR2	122.42	21.6	13480	2	0	0.577106017	0.13358441	2.07933E-52	9.34414E-86
Q9JKF1	Iqgap1	Ras GTPase-activating-like protein IQGAP1	3899.36	65.42	188742	92	0	1.102472694	0.23342411	9.78772E-24	6.26599E-31
Q9JLJ2	Aldh9a1	4-trimethylaminobutyraldehyde dehydrogenase	687.374	69.64	53515	21	0	0.958831319	0.19007698	3.44761E-48	3.75412E-75
Q9QZQ8	H2afy	Core histone macro-H2A.1	155.609	18.01	39735	5	0	1.07710626	0.26575971	8.25615E-16	1.06256E-19
Q9WTP6	Ak2	Adenylate kinase 2, mitochondrial	334.009	47.28	26469	8	0	0.904475564	0.32961405	1	0.63841845
Q9WUM5	Suclg1	Succinate--CoA ligase [ADP/GDP-forming] subunit alpha, mitochondrial	257.113	25.72	36155	7	0	0.861061759	0.19007698	9.65787E-49	2.09158E-76
Q9WVQ5	Apip	Methylthioribulose-1-phosphate dehydratase	113.666	9.13	26949	2	0	0.851251469	0.21730432	3.88007E-30	5.73343E-41
Q9Z0J0	Npc2	NPC intracellular cholesterol transporter 2	140.289	41.61	16442	5	0	0.783300611	0.27534881	1.04594E-11	2.23698E-14
E9Q7G0	Numa1	Nuclear mitotic apparatus protein 1	1707.656	34.62	235630	48	1	0.929641415	0.30513796	5.20087E-05	2.65604E-06
O08807	Prdx4	Peroxiredoxin-4	341.044	54.74	31053	12	1	1.173508932	0.19007698	1.53272E-41	2.80432E-61
O35737	Hnrnph1	Heterogeneous nuclear ribonucleoprotein H	631.314	41.2	49199	14	1	0.904594749	0.21003578	1.2944E-35	3.20056E-50
O55173	Pdpk1	3-phosphoinositide-dependent protein kinase 1	52.966	4.47	63593	2	1	0.596221732	0.25788439	2.89921E-17	1.1584E-21
O70439	Stx7	Syntaxin-7	430.805	44.83	29821	8	1	0.927133043	0.28507094	6.17947E-09	5.94154E-11
P01901	H2-K1	H-2 class I histocompatibility antigen, K-B alpha chain	85.959	9.21	41302	4	1	1.117417985	0.32376711	1	0.49984662
P06151	Ldha	L-lactate dehydrogenase A chain	1682.741	84.64	36499	35	1	0.929243565	0.29433913	2.82228E-07	5.85466E-09
P06339	H2-T23	H-2 class I histocompatibility antigen, D-37 alpha chain	147.539	8.68	40875	3	1	1.182345467	0.27894049	1.42609E-11	3.3096E-14
P08753	Gnai3	Guanine nucleotide-binding protein G(k) subunit alpha	420.479	29.94	40522	10	1	0.878366576	0.21730432	5.70293E-34	2.4042E-47
P19783	Cox4i1	Cytochrome c oxidase subunit 4 isoform 1, mitochondrial	181.422	30.77	19530	5	1	1.065006288	0.24163074	7.81132E-22	4.07853E-28
P25976	Ubtf	Nucleolar transcription factor 1	64.217	6.54	89509	4	1	0.739498768	0.20737785	9.0661E-39	5.72539E-56
P26039	Tln1	Talin-1	5661.689	64.62	269821	120	1	0.976991675	0.25785466	1.97977E-17	6.88355E-22
P27008	Parp1	Poly [ADP-ribose] polymerase 1	679.782	24.85	112660	16	1	0.919689781	0.20737785	1.60235E-38	1.65416E-55
P27773	Pdia3	Protein disulfide-isomerase A3	1310.895	67.33	56678	34	1	1.046527292	0.21730432	5.09857E-31	2.12707E-42
P32921	Wars	Tryptophan--tRNA ligase, cytoplasmic	342.969	29.73	54358	10	1	1.157309994	0.30513796	0.012704669	0.001522726
P36536	Sar1a	GTP-binding protein SAR1a	121.468	27.27	22371	3	1	0.637932946	0.30513796	0.001071466	8.63757E-05
P43883	Plin2	Perilipin-2	41.644	6.35	46646	2	1	1.86808436	0.32135255	0.85951591	0.2518881
P47911	Rpl6	60S ribosomal protein L6	224.616	24.32	33510	7	1	1.182681073	0.40804158	1	0.99999997
P50429	Arsb	Arylsulfatase B	484.17	37.83	59647	14	1	0.844875141	0.2682679	4.02736E-14	1.76379E-17
P60710	Actb	Actin, cytoplasmic 1	3245.62	90.93	41737	62	1	1.067602774	0.21730432	1.26162E-28	1.54549E-38
P61205	Arf3	ADP-ribosylation factor 3	661.188	58.56	20601	14	1	1.092594191	0.21730432	2.34246E-30	2.53209E-41
P62245	Rps15a	40S ribosomal protein S15a	335.051	71.54	14840	13	1	0.93388564	0.23342411	5.76886E-23	8.85177E-30
P62317	Snrpd2	Small nuclear ribonucleoprotein Sm D2	180.707	52.54	13527	7	1	0.873362345	0.19007698	9.68966E-46	6.97624E-70
P63001	Rac1	Ras-related C3 botulinum toxin substrate 1	404.337	54.17	21450	9	1	0.781259368	0.21730432	1.65493E-33	1.50393E-46

P63213	Gng2	Guanine nucleotide-binding protein G(I)/G(S)/G(O) subunit gamma-2	99.828	49.3	7850	3	1	0.839095217	0.23342411	2.38497E-23	2.36888E-30
P63245	Rack1	Receptor of activated protein C kinase 1	898.447	82.02	35077	20	1	0.905584905	0.25788439	9.00359E-17	5.40094E-21
P63259	Actg1	Actin, cytoplasmic 2	3229.127	90.93	41793	61	1	1.068615526	0.21730432	6.41531E-30	1.29356E-40
P83868	Ptges3	Prostaglandin E synthase 3	136.366	26.25	18721	3	1	0.701389709	0.36311283	1	0.99966826
P83870	Phf5a	PHD finger-like domain-containing protein 5A	32.466	21.82	12405	2	1	0.567198852	0.30547343	0.042172257	0.006243256
Q07984	Ssr4	Translocon-associated protein subunit delta	153.742	30.64	18980	4	1	1.268839368	0.24413627	6.51219E-21	8.71359E-27
Q3U1J4	Ddb1	DNA damage-binding protein 1	492.801	19.56	126853	16	1	0.901877493	0.20548165	2.88679E-39	6.67813E-57
Q3V0K9	Pls1	Plastin-1	323.882	11.59	70408	9	1	1.089921318	0.26244782	2.74948E-16	2.44559E-20
Q571I9	Aldh16a1	Aldehyde dehydrogenase family 16 member A1	241.958	13.47	84756	7	1	0.88151529	0.21730432	8.07095E-33	2.20012E-45
Q60973	Rbbp7	Histone-binding protein RBBP7	381.971	45.18	47790	13	1	0.941652781	0.28507094	1.7857E-08	2.15388E-10
Q61686	Cbx5	Chromobox protein homolog 5	139.648	18.85	22186	3	1	0.874662241	0.20737785	4.9785E-38	1.35093E-54
Q61990	Pcbp2	Poly(rC)-binding protein 2	555.288	49.17	38222	13	1	0.897887996	0.21730432	1.36376E-32	5.32141E-45
Q63584	Tmed10	Transmembrane emp24 domain-containing protein 10	373.401	35.16	24858	7	1	1.084705903	0.2558024	9.18024E-18	2.40069E-22
Q68FL6	Mars	Methionine--tRNA ligase, cytoplasmic	594.832	28.49	101431	15	1	0.860164429	0.22884711	2.50504E-26	6.87386E-35
Q6DFW4	Nop58	Nucleolar protein 58	431.638	23.51	60343	9	1	0.906899943	0.21730432	2.3238E-27	1.63923E-36
Q6PDG5	Smarcc2	SWI/SNF complex subunit SMARCC2	128.747	5.94	132604	5	1	0.850354161	0.24413627	7.80611E-20	3.01974E-25
Q6TEK4	Vkorc1	Vitamin K epoxide reductase complex subunit 1	78.781	19.25	17783	2	1	0.772560853	0.21730432	1.43647E-27	7.59311E-37
Q6ZWQ7	Spcs3	Signal peptidase complex subunit 3	55.049	17.78	20313	3	1	1.131968789	0.30513796	7.6412E-05	4.14E-06
Q794E4	Hnrnpf	Heterogeneous nuclear ribonucleoprotein F	906.329	65.78	45730	19	1	0.92467834	0.19007698	4.30096E-47	9.5017E-73
Q80UJ7	Rab3gap1	Rab3 GTPase-activating protein catalytic subunit	70.334	6.01	110198	3	1	0.927469132	0.30513796	0.000134131	7.91449E-06
Q8BUM3	Ptpn7	Tyrosine-protein phosphatase non-receptor type 7	193.124	21.73	40351	5	1	0.885579565	0.30513796	0.00273087	0.000254891
Q8BZW8	Nhlrc2	NHL repeat-containing protein 2	86.826	3.17	78430	2	1	1.489667127	0.30468754	2.35443E-05	1.06141E-06
Q8CGK3	Lonp1	Lon protease homolog, mitochondrial	813.421	32.67	105843	22	1	1.102458889	0.28507094	1.57116E-09	1.12367E-11
Q8R2U0	Seh1l	Nucleoporin SEH1	78.308	14.17	39775	4	1	1.131650492	0.21730432	2.30021E-32	1.28304E-44
Q8VCT3	Rnpep	Aminopeptidase B	1034.075	55.85	72416	27	1	0.919007372	0.2700068	5.89883E-13	5.67835E-16
Q8VDL4	Adpgk	ADP-dependent glucokinase	153.351	11.69	53902	4	1	1.143296045	0.30044021	5.46268E-06	1.93092E-07
Q8VDM6	Hnrnpul1	Heterogeneous nuclear ribonucleoprotein U-like protein 1	336.085	17.23	96002	11	1	0.82404115	0.30351064	1.92199E-05	8.38378E-07
Q8VIJ6	Sfpq	Splicing factor, proline- and glutamine-rich	665.644	31.9	75442	18	1	0.915796431	0.34568264	1	0.99161318
Q91YH5	Atl3	Atlastin-3	275.605	21.44	60575	7	1	1.169822729	0.30513796	0.018570175	0.002375411
Q91YQ5	Rpn1	Dolichyl-diphosphooligosaccharide--protein glycosyltransferase subunit 1	874.429	46.71	68528	22	1	1.074921917	0.19007698	1.79699E-45	2.54773E-69
Q91ZX7	Lrp1	Prolow-density lipoprotein receptor-related protein 1	2764.038	22.4	504742	73	1	0.885694922	0.21730432	7.71407E-29	7.02882E-39
Q99NB9	Sf3b1	Splicing factor 3B subunit 1	1219.249	29.6	145816	30	1	0.934063113	0.23103754	2.57986E-25	2.52113E-33
Q9CSN1	Snw1	SNW domain-containing protein 1	136.612	9.33	61475	3	1	0.885455112	0.30547343	0.026689309	0.003639243
Q9DC50	Crot	Peroxisomal carnitine O-octanoyltransferase	63.466	6.54	70264	2	1	0.660754714	0.24657069	3.93986E-19	2.99843E-24
Q9JHJ3	Glmp	Glycosylated lysosomal membrane protein	116.49	13.12	43804	3	1	0.813000595	0.19007698	8.04737E-47	3.62111E-72
Q9JKP5	Mbnl1	Muscleblind-like protein 1	28.027	6.45	36976	2	1	0.902987531	0.32376711	1	0.39461287
Q9QWT9	Kifc1	Kinesin-like protein KIFC1	85.723	8.46	74153	4	1	0.858901013	0.21319152	3.85211E-35	2.21105E-49
Q9QXG4	Acss2	Acetyl-coenzyme A synthetase, cytoplasmic	116.082	7.42	78862	3	1	0.633892322	0.21730432	4.70813E-29	3.18766E-39
Q9R0X4	Acot9	Acyl-coenzyme A thioesterase 9, mitochondrial	118.054	18.22	50560	6	1	1.255790773	0.21730432	1.82823E-31	3.97487E-43
Q9R233	Tapbp	Tapasin	125.12	16.56	49736	6	1	1.166527914	0.21730432	1.41162E-30	1.11432E-41

Q9Z0S1	Bpnt1	3'(2'),5'-bisphosphate nucleotidase 1	96.983	13.64	33196	3	1	0.845941076	0.22752045	3.75238E-27	3.52451E-36
Q9Z2L7	Crlf3	Cytokine receptor-like factor 3	120.444	20.81	49559	4	1	0.798701327	0.22884711	4.0083E-26	1.42643E-34
A6H5Z3	Exoc6b	Exocyst complex component 6B	77.214	5.56	94130	3	2	1.273485682	0.34568264	1	0.9882078
B0BNM1	Naxe	NAD(P)H-hydrate epimerase	199.691	29.43	30891	4	2	0.712774135	0.34335689	1	0.95999631
B9EJ86	Osbpl8	Oxysterol-binding protein-related protein 8	668.143	27.11	101269	18	2	1.107375456	0.24413627	5.18394E-20	1.68871E-25
E9Q1P8	Irf2bp2	Interferon regulatory factor 2-binding protein 2	130.928	11.58	59292	4	2	0.925494688	0.44788539	1	1
F1M775	Diaph1	Protein diaphanous homolog 1	461.72	12.65	140409	12	2	1.123745239	0.2682679	1.11767E-13	6.62068E-17
O35114	Scarb2	Lysosome membrane protein 2	48.249	12.97	54044	4	2	1.174255995	0.24163074	3.30152E-22	1.15979E-28
O35239	Ptpn9	Tyrosine-protein phosphatase non-receptor type 9	99.563	10.29	67970	4	2	0.549169256	0.21730432	3.05586E-31	9.22423E-43
O54774	Ap3d1	AP-3 complex subunit delta-1	96.886	5.34	135081	4	2	0.840313903	0.27894049	3.57377E-11	1.04728E-13
O54988	Slk	STE20-like serine/threonine-protein kinase	256.181	9	141457	9	2	0.869774668	0.20981848	7.48307E-36	1.20273E-50
O70145	Ncf2	Neutrophil cytosol factor 2	441.898	43.62	59485	17	2	0.919604096	0.2682679	1.42684E-14	4.55048E-18
O88838	Spsb2	SPRY domain-containing SOCS box protein 2	11.784	6.82	28938	2	2	1.84341756	0.24825716	8.75323E-19	9.19938E-24
P01900	H2-D1	H-2 class I histocompatibility antigen, D-D alpha chain	393.694	33.42	41110	12	2	1.103110134	0.30970752	0.16596425	0.032119244
P04441	Cd74	H-2 class II histocompatibility antigen gamma chain	251.246	21.51	31557	6	2	1.062924886	0.27894049	2.63607E-11	7.15305E-14
P05555	Itgam	Integrin alpha-M CD11b	968.769	29.31	127481	23	2	0.937863358	0.2682679	2.8551E-14	1.12632E-17
P08228	Sod1	Superoxide dismutase [Cu-Zn]	173.22	34.42	15943	4	2	0.88267918	0.23342411	1.52926E-23	1.22014E-30
P08508	Fcgr3	Low affinity immunoglobulin gamma Fc region receptor III	55.362	8.05	30036	2	2	0.88097937	0.32961405	1	0.71700353
P09055	Itgb1	Integrin beta-1	467.858	21.55	88231	13	2	0.896515266	0.28507094	5.07607E-10	2.82839E-12
P09103	P4hb	Protein disulfide-isomerase	1539.052	66.6	57058	38	2	1.052306347	0.29414201	6.44452E-08	1.00983E-09
P09411	Pgk1	Phosphoglycerate kinase 1	2028.486	82.97	44550	48	2	0.944655104	0.23128573	1.02265E-24	2.05754E-32
P13471	Rps14	40S ribosomal protein S14	311.078	66.89	16259	9	2	0.936865748	0.27894049	1.94072E-11	4.8724E-14
P14733	Lmnb1	Lamin-B1	1089.586	57.99	66786	32	2	0.955287605	0.23342411	6.25294E-24	3.20019E-31
P17047	Lamp2	Lysosome-associated membrane glycoprotein 2	207.745	17.11	45681	7	2	1.09499716	0.29732277	2.27238E-06	6.89483E-08
P17182	Eno1	Alpha-enolase	1915.87	79.26	47141	40	2	0.943830323	0.27894049	8.80416E-11	3.22616E-13
P17225	Ptbp1	Polypyrimidine tract-binding protein 1	1128.374	66.98	56478	21	2	0.906433418	0.29936128	3.53681E-06	1.16053E-07
P19253	Rpl13a	60S ribosomal protein L13a	144.874	27.59	23464	6	2	1.107453316	0.29686941	1.44881E-06	4.05691E-08
P21300	Akr1b7	Aldose reductase-related protein 1	111.066	15.82	35988	6	2	1.076573729	0.22829718	9.73076E-27	1.57137E-35
P23198	Cbx3	Chromobox protein homolog 3	366.566	42.62	20855	7	2	0.888633541	0.26244782	3.97411E-16	4.0056E-20
P24369	Ppib	Peptidyl-prolyl cis-trans isomerase B	393.827	43.06	23713	11	2	1.065995295	0.2682679	2.1865E-13	1.57844E-16
P28740	Kif2a	Kinesin-like protein KIF2A	100.667	5.53	79756	4	2	0.863815653	0.37616256	1	0.99999152
P29418	Atp5f1e	ATP synthase subunit epsilon, mitochondrial	2.148	29.41	5767	2	2	0.635655679	0.22829718	1.56271E-26	3.29266E-35
P35564	Canx	Calnexin	771.865	29.1	67278	17	2	1.07457164	0.24413627	9.89738E-21	1.58905E-26
P40124	Cap1	Adenylyl cyclase-associated protein 1	1729.781	73.42	51565	40	2	0.944986989	0.20737785	1.43033E-36	6.13662E-52
P41565	Idh3g	Isocitrate dehydrogenase [NAD] subunit gamma 1, mitochondrial	188.881	21.63	42851	6	2	0.884012134	0.23128573	6.47358E-25	1.02712E-32
P49242	Rps3a	40S ribosomal protein S3a	727.985	57.58	29945	20	2	0.887662227	0.34568264	1	0.98683272
P49717	Mcm4	DNA replication licensing factor MCM4	823.128	32.48	96736	22	2	0.918095248	0.30513796	9.23533E-05	5.14943E-06
P56135	Atp5mf	ATP synthase subunit f, mitochondrial	50.03	34.09	10344	4	2	1.109296296	0.25546543	6.2339E-18	1.40963E-22
P57716	Ncstn	Nicastrin	174.822	12.01	78492	6	2	1.112639054	0.27166054	4.07994E-12	6.75132E-15
P60892	Prps1	Ribose-phosphate pyrophosphokinase 1	338.338	27.04	34834	8	2	1.123564546	0.25788439	6.18274E-17	3.24303E-21
P61028	Rab8b	Ras-related protein Rab-8B	303.135	44.44	23603	9	2	1.114711399	0.25681693	1.34939E-17	4.07355E-22
P61354	Rpl27	60S ribosomal protein L27	150.104	36.03	15798	5	2	1.105769465	0.26627283	1.00584E-14	2.87338E-18
P61589	Rhoa	Transforming protein RhoA	508.556	64.77	21782	11	2	0.827756774	0.21730432	1.0918E-31	1.70673E-43
P62270	Rps18	40S ribosomal protein S18	304.374	54.61	17719	13	2	1.072471959	0.24413627	3.43622E-20	9.40887E-26

P63158	Hmgb1	High mobility group protein B1	259.891	35.81	24894	9	2	0.8578287	0.29686941	1.15352E-06	3.10133E-08
P68037	Ube2l3	Ubiquitin-conjugating enzyme E2 L3	192.778	51.95	17862	4	2	0.890058672	0.31014969	0.19611539	0.039315603
P70333	Hnrnph2	Heterogeneous nuclear ribonucleoprotein H2	330.103	25.17	49280	8	2	0.900568955	0.28124977	2.13218E-10	9.69885E-13
P84082	Arf2	ADP-ribosylation factor 2	569.006	58.56	20746	13	2	1.090046349	0.24453979	2.63589E-19	1.69759E-24
P84096	Rhog	Rho-related GTP-binding protein RhoG	411.779	51.83	21309	9	2	0.845645683	0.24067098	1.38516E-22	3.24205E-29
Q04692	Smarcad1	SWI/SNF-related matrix-associated actin-dependent regulator of chromatin subfamily A containing DEAD/H box 1	102	4.51	116451	3	2	0.803274931	0.29433913	2.21707E-07	4.39489E-09
Q2TBE6	Pi4k2a	Phosphatidylinositol 4-kinase type 2-alpha	55.402	3.34	54258	2	2	1.191827363	0.27166054	2.96978E-12	4.50636E-15
Q3T1J9	Mob1a	MOB kinase activator 1A	118.443	33.8	25080	6	2	1.062070226	0.30513796	0.003170452	0.000303084
Q3TBD2	Arhgap45	Rho GTPase-activating protein 45	339.323	15.77	122902	11	2	0.907713491	0.29433913	7.27028E-07	1.79505E-08
Q3TCN2	Plbd2	Putative phospholipase B-like 2	263.875	17	66289	8	2	0.788186964	0.32376711	1	0.55289498
Q3THK7	Gmps	GMP synthase [glutamine-hydrolyzing]	511.269	31.17	76723	17	2	0.925830686	0.27166054	1.5646E-12	1.98744E-15
Q3TRM8	Hk3	Hexokinase-3	1391.424	50.65	100101	33	2	1.23300809	0.32376711	0.93335884	0.28132664
Q4FZY0	Efhd2	EF-hand domain-containing protein D2	624.41	58.16	26759	16	2	1.05405137	0.30166128	1.27343E-05	5.1889E-07
Q4V7C7	Actr3	Actin-related protein 3	974.506	76.56	47357	29	2	0.919608436	0.24413627	2.8037E-21	2.59348E-27
Q4V8H8	Ehd2	EH domain-containing protein 2	30.884	3.5	61237	2	2	1.104120752	0.20737785	8.75188E-38	3.80668E-54
Q5XI73	Arhgdia	Rho GDP-dissociation inhibitor 1	574.961	77.94	23407	15	2	0.917503881	0.29414201	1.36032E-07	2.4568E-09
Q61183	Papola	Poly(A) polymerase alpha	33.647	3.38	82309	2	2	0.703755546	0.29414201	1.06249E-07	1.83097E-09
Q61316	Hspa4	Heat shock 70 kDa protein 4	1248.721	50.3	94133	30	2	0.945588414	0.30547343	0.058290321	0.009157355
Q61699	Hsph1	Heat shock protein 105 kDa	136.096	5.48	96407	3	2	0.729615566	0.2682679	5.67024E-14	2.74864E-17
Q61820	Rasl2-9	GTP-binding nuclear protein Ran, testis-specific isoform	154.659	25	24452	6	2	0.934013098	0.31168245	0.35665743	0.081811092
Q62159	Rhoc	Rho-related GTP-binding protein RhoC	544.859	65.8	22006	11	2	0.827359531	0.21730432	6.50834E-32	7.30752E-44
Q640N3	Arhgap30	Rho GTPase-activating protein 30	48.964	5.18	120114	3	2	0.832564347	0.21730432	8.49135E-31	4.88691E-42
Q69ZN7	Myof	Myoferlin	429.761	10.11	233324	17	2	1.113769486	0.24413627	1.1982E-21	7.60908E-28
Q6A028	Swap70	Switch-associated protein 70	515.661	32.82	68996	15	2	0.898624906	0.38229794	1	0.99999413
Q6ZPF4	Fmnl3	Formin-like protein 3	410.717	14.4	117169	10	2	0.851591711	0.30547343	0.030006931	0.004178273
Q80SW1	Ahcyl1	S-adenosylhomocysteine hydrolase-like protein 1	265.391	25.28	58951	10	2	0.918508708	0.24067098	8.94737E-23	1.70315E-29
Q80UM7	Mogs	Mannosyl-oligosaccharide glucosidase	495.036	21.82	91831	11	2	1.105813534	0.30547343	0.033674166	0.004787065
Q80UU9	Pgrmc2	Membrane-associated progesterone receptor component 2	189.428	20.28	23334	2	2	0.541445519	0.23342411	3.71266E-23	4.58538E-30
Q80VD1	Fam98b	Protein FAM98B	80.732	6.76	45349	2	2	0.888992761	0.31168245	0.33295811	0.075118817
Q8BH86	Dglucy	D-glutamate cyclase, mitochondrial	162.102	14.42	66366	5	2	0.818974968	0.2682679	7.9683E-14	4.27291E-17
Q8BH95	Echs1	Enoyl-CoA hydratase, mitochondrial	206.452	23.1	31474	5	2	0.87439013	0.27166054	2.15762E-12	2.99749E-15
Q8BK64	Ahsa1	Activator of 90 kDa heat shock protein ATPase homolog 1	296.164	32.84	38117	7	2	1.14062151	0.32961405	1	0.68655037
Q8BMK4	Ckap4	Cytoskeleton-associated protein 4	910.017	51.48	63692	24	2	1.092209153	0.22752045	6.04816E-27	7.45331E-36
Q8BUR4	Dock1	Dedicator of cytokinesis protein 1	77.53	1.88	215085	3	2	0.852304158	0.21730432	5.45912E-28	1.61421E-37
Q8BVI4	Qdpr	Dihydropteridine reductase	216.144	31.12	25570	5	2	0.863244122	0.27166054	5.59449E-12	1.0091E-14
Q8C156	Ncaph	Condensin complex subunit 2	76.659	5.06	82303	2	2	0.694736573	0.46500925	1	1
Q8C878	Uba3	NEDD8-activating enzyme E1 catalytic subunit	227.269	24.89	51737	6	2	0.816779358	0.30513796	0.011152637	0.001307915
Q8C9B9	Dido1	Death-inducer obliterator 1	38.162	1.37	247176	2	2	0.861617872	0.24825716	1.93032E-18	2.77861E-23
Q8CFI7	Polr2b	DNA-directed RNA polymerase II subunit RPB2	67.151	5.79	133911	5	2	0.786317889	0.32376711	1	0.51757375
Q8K2V6	Ipo11	Importin-11	82.276	3.9	112416	2	2	0.76039248	0.28507094	3.59373E-09	3.07451E-11
Q8K411	Pitrm1	Presequence protease, mitochondrial	259.001	16.89	117372	11	2	0.889329306	0.30547343	0.064692459	0.010363383
Q8R0G9	Nup133	Nuclear pore complex protein Nup133	140.219	7.53	128620	5	2	0.844584906	0.29686941	1.8162E-06	5.29426E-08
Q8VE99	Ccdc115	Coiled-coil domain-containing protein 115	70.489	22.78	19743	2	2	0.79138375	0.23128573	4.09042E-25	5.11191E-33

Q8WTY4	Ciapin1	Anamorsin	142.093	29.77	33429	5	2	0.701233726	0.31926343	0.71619676	0.19815419
Q91XU1	Qki	Protein quaking	203.341	23.75	37643	5	2	0.834706533	0.21730432	1.74426E-29	6.47029E-40
Q99K51	Pls3	Plastin-3	1198.189	64.6	70742	32	2	1.063142115	0.29247805	3.8791E-08	5.50721E-10
Q99KP6	Prpf19	Pre-mRNA-processing factor 19	539.892	46.23	55239	14	2	0.899185235	0.23128573	2.53804E-24	8.16907E-32
Q99MD9	Nasp	Nuclear autoantigenic sperm protein	209.463	13.45	83954	7	2	0.82981051	0.24413627	4.27691E-21	4.7626E-27
Q99P91	Gpnmb	Transmembrane glycoprotein NMB	122.195	7.67	63676	5	2	1.221748592	0.28507094	6.75214E-10	4.01019E-12
Q9CPU4	Mgst3	Microsomal glutathione S-transferase 3	204.663	49.67	16958	4	2	0.889985192	0.34389346	1	0.96989572
Q9CR62	Slc25a11	Mitochondrial 2-oxoglutarate/malate carrier protein	437.786	57.01	34155	15	2	1.120601088	0.2700068	3.04959E-13	2.42886E-16
Q9CX86	Hnrnpa0	Heterogeneous nuclear ribonucleoprotein A0	472.942	32.79	30530	10	2	0.93185776	0.33140787	1	0.83374379
Q9CXF4	Tbc1d15	TBC1 domain family member 15	177.005	8.64	76527	5	2	0.880527655	0.34389346	1	0.97983101
Q9CZX9	Emc4	ER membrane protein complex subunit 4	49.036	15.85	20117	2	2	2.33834168	0.20548165	9.12645E-40	7.61364E-58
Q9JI75	Nqo2	Ribosyldihydronicotinamide dehydrogenase [quinone]	90.129	22.08	26248	3	2	3.393253366	0.28507094	4.71697E-09	4.27917E-11
Q9JK81	Myg1	UPF0160 protein MYG1, mitochondrial	343.682	30.53	42723	8	2	0.946486505	0.36932998	1	0.99990523
Q9JM14	Nt5c	5'(3')-deoxyribonucleotidase, cytosolic type	114.573	27.5	23076	3	2	0.838988226	0.28507094	2.07407E-09	1.5761E-11
Q9WU28	Pfdn5	Prefoldin subunit 5	128.072	21.43	17356	2	2	0.803748195	0.3139939	0.46177628	0.11303125
Q9WU78	Pdcd6ip	Programmed cell death 6-interacting protein	1224.259	53.39	96024	35	2	0.948587472	0.27933403	1.18457E-10	4.67111E-13
