# Supplementary material for: Repeated Exposure of Macrophages to Synthetic Amorphous Silica Induces Adaptive Proteome Changes and a Moderate Cell Activation
Source: Nanomaterials (Basel). 2022 Apr 22;12(9):1424. doi: 10.3390/nano12091424 (PMC9105884; doi:10.3390/nano12091424)
Supplement: Supplementary file 1 [file nanomaterials-12-01424-s001.zip › Table S4.pdf]

Table S4: results of the pathway analysis by the DAVID tool using proteins modulated by precipitated silica
cutoff value for cluster selection: FDR≤0.1

Annotation Cluster 1 Enrichment Score: 19.09662544270277

Category	Term	Count	PValue	Genes	Fold Enrichment	FDR
GOTERM_MF_DIRECT	GO:0098641~cadherin binding involved in cell-cell adhesion	53	2.10611223169521E-22	Q9D8U8, O08709, P19096, Q9Z0P5, Q99KN9, O35685, O08992, P60335, P47753, Q5FWK3, P52480, P47911, P47757, Q9CR57, P24452, Q99LX0, Q62418, Q9CPV4, P11499, P18572, Q7M6Y3, P16546, Q9DBG5, P42932, Q9CWK8, P06151, E9PVA8, O88844, P05064, Q922Q8, P62962, Q9WVA4, Q62261, P59325, Q9DCL9, Q9CWJ9, A2AGT5, Q8VDD5, A2APV2, P62855, O54988, Q99KH8, P26039, P61979, Q61029, Q9Z1Q5, Q9WUM3, Q9EQP2, P35700, Q62433, Q61191, P17182, Q9WUA3	5.11437452984645	1.0330480496465E-19
GOTERM_CC_DIRECT	GO:0005913~cell-cell adherens junction	54	2.7573349787343E-22	Q62523, Q9D8U8, O08709, P19096, Q9Z0P5, Q99KN9, O35685, O08992, P60335, P47753, Q5FWK3, P52480, P47911, P47757, Q9CR57, P24452, Q99LX0, Q62418, Q9CPV4, P11499, P18572, Q7M6Y3, P16546, Q9DBG5, P42932, Q9CWK8, P06151, E9PVA8, O88844, P05064, Q922Q8, P62962, Q9WVA4, Q62261, P59325, Q9DCL9, Q9CWJ9, A2AGT5, Q8VDD5, A2APV2, P62855, O54988, Q99KH8, P26039, P61979, Q61029, Q9Z1Q5, Q9WUM3, Q9EQP2, P35700, Q62433, Q61191, P17182, Q9WUA3	5.0223647613101	1.90256113532667E-20

GOTERM_BP_DIRECT	GO:0098609~cell-cell adhesion	35	8.83390310365848E-15	O08709, Q9D8U8, Q9Z0P5, P59325, Q9DCL9, Q9CWJ9, A2AGT5, Q99KN9, O35685, A2APV2, P62855, P47753, Q5FWK3, O54988, Q99KH8, P47757, P26039, Q9CR57, Q61029, P24452, Q62418, Q9CPV4, P18572, Q9EQP2, Q62433, P35700, Q9DBG5, Q61191, P06151, O88844, P05064, P17182, Q9WUA3, Q922Q8, Q9WVA4	5.08893391872115	1.21775354283932E-11

Annotation Cluster 2 Enrichment Score: 16.781319002796423

Category	Term	Count	PValue	Genes	Fold Enrichment	FDR
UP_KEYWORDS	Lysosome	43	7.05284865873525E-20	Q8BFR4, Q9Z0J0, Q9WV54, Q9CQW9, O08709, P20060, O70370, P51569, P97821, P18242, Q9ESY9, Q9CQ22, P70665, P52875, Q571E4, Q9JHJ3, Q8VEH3, Q9QWR8, O88531, Q8VEB4, O89023, P10605, P23780, Q8VE99, Q9Z0M5, Q61207, P48441, Q8K2I4, P50428, P17439, Q9WUU7, P12265, O09159, P16675, P50429, O89017, Q9JHS3, P24668, Q7TMR0, Q3TCN2, P29416, O70404, O88384	5.73444583605285	3.09149866207895E-18

GOTERM_CC_DIRECT	GO:0005764~lysosome	50	2.37199097867177E-18	Q8BFR4, O08709, O70370, P97821, P18242, P70665, P52875, Q9JHJ3, Q8VEH3, P16460, O88531, P04441, O89023, P10605, Q9Z0M5, Q61207, P48441, P50428, P17439, P12265, P16675, P50429, O89017, Q7TMR0, O70404, P29416, Q9Z0J0, Q9WV54, Q9CQW9, P20060, O08529, P51569, Q9ESY9, Q9CQ22, Q571E4, O54782, Q9QWR8, Q8VEB4, O70439, P08228, P23780, Q8VE99, P24270, Q8K2I4, Q9WUU7, O09159, Q9JHS3, P24668, Q3TCN2, O88384	4.43959736089849	1.27296849188719E-16
KEGG_PATHWAY	mmu04142:Lysosome	33	2.70748085207608E-14	Q8BFR4, Q9Z0J0, Q9WV54, P20060, Q68FD5, O70370, P51569, P97821, P18242, P51863, Q571E4, O35643, Q9QWR8, O88531, Q8VEB4, O89023, P10605, P23780, Q9Z0M5, Q61207, Q8R0H9, P48441, Q8K2I4, P50428, P17439, Q9WUU7, P12265, O09159, P16675, P50429, O89017, P24668, P29416	4.97691975841242	6.41672961942032E-12

Annotation Cluster 3 Enrichment Score: 10.497346398991686

Category	Term	Count	PValue	Genes	Fold Enrichment	FDR

UP_KEYWORDS	Ribonucleoprotein	46	1.12050450387712E-18	Q9CX86, P62918, Q9EQK5, Q9D7S7, Q60668, Q921F4, P19253, Q8VDM6, P14115, Q61990, P63323, P62751, Q6DFW4, P62855, P60335, P62317, P47911, P62830, P62908, Q9CR57, P61979, P62309, P62849, P70333, Q9JIK9, P14206, Q64012, P14148, P62242, P63276, O55142, P14131, P62267, P61255, P84099, Q9D8E6, O88569, P62305, P62702, P62307, P27659, O35737, P60867, Q8CCF0, P41105, P62843	4.9594037002529	4.20989549313831E-17
GOTERM_CC_DIRECT	GO:0030529~intracellular ribonucleoprotein complex	49	3.07572462802306E-18	P62918, Q9EQK5, Q9D7S7, O70133, Q60668, P19253, Q6P5F9, P32067, P14115, Q61990, P62751, Q6DFW4, P60335, P62317, P47911, P62830, Q9CR57, P62849, Q60591, P14206, P70333, Q9JIK9, P62242, O55142, P14131, P57780, P84099, O88569, P60867, P62843, Q9CX86, Q921F4, Q8VDM6, P60710, P62908, Q6PB66, P61979, Q64012, P14148, P63276, P62267, Q9D8E6, P62305, P62702, P62307, P27659, O35737, Q8CCF0, P41105	4.50036434977578	1.48557499533514E-16

GOTERM_BP_DIRECT	GO:0006412~translation	47	5.07021685167495E-12	P62918, P51881, Q9D7S7, Q9CR62, P19253, O70251, P59325, Q922B2, P14115, P63323, P62751, Q9CZD3, Q8BMJ2, Q8BMJ3, P62855, P26638, Q8QZY1, P48962, P47911, P62830, Q99PL5, O89086, P05555, Q60872, P62908, Q9CR57, Q99JX4, P62849, Q3THG9, P10126, P14206, P14148, P62242, P63276, O55142, P14131, Q8R050, P62267, P61255, P84099, Q9D8E6, P62702, P27659, P60867, P41105, P60843, P62843	3.22087638047738	4.65952928668928E-09
GOTERM_CC_DIRECT	GO:0005840~ribosome	29	4.80108085276811E-11	P62918, Q9D7S7, P19253, P14115, P62751, P62855, P47911, P62830, Q99PL5, P05555, P62908, Q9CR57, P62849, P35564, Q9JIK9, P14206, P62242, P63276, O55142, P14131, P62267, P84099, Q9D8E6, P62702, P27659, P60867, P41105, P62843	4.53358458162389	1.54594803459133E-09
UP_KEYWORDS	Ribosomal protein	28	1.01886353827528E-10	P62918, Q9D7S7, P19253, P14115, P63323, P62751, P62855, P47911, P62830, P62908, Q9CR57, P62849, Q9JIK9, P14206, P14148, P62242, P63276, O55142, P14131, P62267, P61255, P84099, Q9D8E6, P62702, P27659, P60867, P41105, P62843	4.58019891957389	2.23300925471999E-09
GOTERM_MF_DIRECT	GO:0003735~structural constituent of ribosome	32	1.4593378627042E-08	P62918, P51881, Q9D7S7, Q9CR62, P19253, P14115, P63323, P62751, P62855, P48962, P47911, P62830, P05555, P62908, Q9CR57, P62849, Q9JIK9, P14206, P14148, P62242, P63276, O55142, P14131, P62267, P61255, P84099, Q9D8E6, P62702, P27659, P60867, P41105, P62843	3.26337448559671	1.78951305414103E-06

KEGG_PATHWAY	mmu03010:Ribosome	27	5.06775372155885E-08	P62918, Q9D7S7, P19253, P14115, P63323, P62751, P62855, P47911, P62830, P62908, Q9CR57, P62849, P14206, P14148, P62242, P63276, O55142, P14131, P62267, P61255, P84099, Q9D8E6, P62702, P27659, P60867, P41105, P62843	3.42611780234285	2.00176272001575E-06
GOTERM_CC_DIRECT	GO:0022625~cytosolic large ribosomal subunit	16	3.9684178608124E-07	P62918, Q9CR57, Q9D7S7, P19253, P14115, P14148, O55142, P62751, P63323, P61255, P84099, Q9D8E6, P27659, P47911, P62830, P41105	5.1674961809491	8.33367750770603E-06
GOTERM_CC_DIRECT	GO:0022627~cytosolic small ribosomal subunit	12	1.33183686026453E-06	P63276, P62242, P62908, P14131, P62267, P63323, P62849, P62855, P62702, P14206, P60867, P62843	6.65437008207124	2.38250816113989E-05

Annotation Cluster 4 Enrichment Score: 7.890100737654368

Category	Term	Count	PValue	Genes	Fold Enrichment	FDR
GOTERM_BP_DIRECT	GO:0006457~protein folding	25	3.19788636032583E-11	Q8BYA0, P27773, P26883, P07901, P38647, O35685, P08003, P04441, Q922R8, P80315, Q9CQN1, P11499, P35564, Q64378, Q8R180, P17742, P14211, P42932, Q61390, P24369, P08113, P09103, Q8VBT0, P63038, P80317	5.36723499240122	1.76331453908367E-08
UP_KEYWORDS	Chaperone	25	3.55453489094184E-09	Q8BYA0, P07901, P38647, Q9JKR6, P04441, Q922R8, P80315, Q9CQN1, Q99LX0, P70670, P11499, P35564, Q64378, O08997, P14211, P42932, Q9JK23, O08583, Q9CYG7, Q61390, Q9CQI6, P08113, P09103, P63038, P80317	4.34639295378412	5.49907456657473E-08
GOTERM_MF_DIRECT	GO:0051082~unfolded protein binding	13	1.87954175748902E-05	P80315, Q9CQN1, P07901, Q8VE99, P38647, P11499, P35564, P14211, P42932, O35685, Q61390, P08113, P80317	4.66662551440329	0.000970437086367

Annotation Cluster 5 Enrichment Score: 7.845354276928301

Category	Term	Count	PValue	Genes	Fold Enrichment	FDR
UP_KEYWORDS	Actin-binding	28	1.25850830844516E-08	Q7TPR4, Q3V0K9, Q61879, Q9Z0P5, Q62261, Q9CPW4, P18760, P40124, P61161, Q8VDD5, Q8R1S4, Q9JM76, P47753, P47754, P47757, P24452, O70200, Q62418, Q9WUM3, Q9WUM4, Q9D2V7, P16546, P13020, P57780, Q9CQI6, O88342, P62962, P28667	3.6896046852123	1.74204044800567E-07
GOTERM_MF_DIRECT	GO:0003779~actin binding	37	1.35988946778766E-08	Q7TPR4, Q3V0K9, Q61879, Q9Z0P5, Q62261, Q9CPW4, Q71LX4, P18760, P19973, P07091, P40124, P61161, Q8VDD5, Q8R1S4, Q9JM76, A2APV2, P47753, P47754, Q9ERL7, P47757, P26039, P24452, O70200, Q62418, Q9WUM3, Q9WUM4, Q9D2V7, P16546, P70315, Q9JL26, P13020, Q6ZPF4, P57780, Q9CQI6, O88342, P62962, P28667	2.94717473884141	1.78951305414103E-06
GOTERM_MF_DIRECT	GO:0051015~actin filament binding	22	1.70050327220895E-08	Q7TPR4, Q3V0K9, P28650, Q61879, O70200, Q62418, Q9WUM3, Q9WUM4, Q9CPW4, Q71LX4, P18760, Q9JL26, P61161, Q8VDD5, Q8R1S4, Q9JM76, P57780, Q3UZA1, P47753, O88342, P47757, P26039	4.48713991769547	1.85354856670775E-06

Annotation Cluster 6 Enrichment Score: 6.9741174880617525

Category	Term	Count	PValue	Genes	Fold Enrichment	FDR

UP_KEYWORDS	Oxidoreductase	48	1.82212176991372E-08	P09528, P51660, Q91YR9, O08709, O08807, P19096, P99029, Q9DBB8, Q9CY64, Q9JMH6, Q9DCD0, Q9ESY9, Q8BVI4, Q9JLJ2, P52019, Q64105, Q62465, Q9DCN2, Q8C0L6, P22437, P36552, Q00612, P09671, P00405, P45376, Q00519, Q60597, Q3U4I7, Q8CIZ9, P08228, P24270, P51174, Q6P3A8, Q9JII6, Q99KP3, Q8R180, P35700, P20108, P14152, Q61093, P62482, P06151, O88844, O09131, Q9Z110, O70503, P48758, Q8BMS1	2.4943806322562	2.3031695603651E-07
GOTERM_BP_DIRECT	GO:0055114~oxidation-reduction process	55	4.91978151291858E-08	P09528, P51660, Q91YR9, O08709, P19096, Q9JMH6, Q8BVI4, Q9JLJ2, Q64105, Q9DCN2, Q8C0L6, P36552, P45376, Q8R1I1, Q00519, Q60597, Q8R180, Q61093, P06151, P62482, O88844, O09131, Q8BMS1, P48758, P50396, O08807, P99029, Q9DBB8, Q9CY64, Q9DCD0, Q9ESY9, P52019, Q62465, P22437, Q571I9, Q00612, P09671, P00405, Q3U4I7, Q8CIZ9, P08228, P24270, O70145, P51174, A2AAY5, Q6P3A8, Q9JII6, Q99KP3, P35700, P20108, P14152, Q3U9G9, Q8VBT0, Q9Z110, O70503	2.23581860038488	1.69547970388956E-05

GOTERM_MF_DIRECT	GO:0016491~oxidoreductase activity	51	9.63973960124413E-08	P09528, P51660, Q91YR9, O08709, P19096, Q9JMH6, Q8BVI4, Q9JLJ2, Q64105, Q9DCN2, Q8C0L6, P36552, P45376, Q00519, Q60597, Q8R180, Q61093, P06151, P62482, O88844, O09131, Q8BMS1, P48758, P50396, O08807, P99029, Q9DBB8, Q9CY64, Q9DCD0, Q9ESY9, P52019, Q62465, P22437, Q571I9, Q00612, P09671, P00405, Q3U4I7, Q8CIZ9, P08228, P28352, P24270, P51174, Q6P3A8, Q9JII6, Q99KP3, P35700, P20108, P14152, Q9Z110, O70503	2.27328611724307	8.59689504438227E-06
UP_KEYWORDS	NADP	20	1.46872853362636E-06	Q8CIZ9, Q91YR9, P24270, P19096, Q9DBB8, Q9CY64, Q9JMH6, Q9DCD0, Q8BVI4, Q9JII6, P52019, Q64105, Q00612, Q61093, P62482, O88844, P45376, Q9Z110, P48758, O70503	3.79502196193265	1.37955572979904E-05
**Annotation Cluster 7**	Enrichment Score: 6.272864013280407					
Category	Term	Count	PValue	Genes	Fold Enrichment	FDR

UP_KEYWORDS	Mitochondrion	76	4.59025732476503E-12	Q9D964, P11928, Q9R0X4, Q8BH95, Q8JZR0, Q9D6U8, Q9CR62, P38647, Q8VDK1, Q9D880, Q9WTP6, Q9DCN2, O35129, P36552, P63087, Q05921, Q8BGH2, P48962, P56480, P41216, Q8R1I1, Q60597, Q99LX0, Q8BH86, Q9CPV4, Q03265, P19783, Q9JIK9, Q8BMF4, P67778, Q9CYG7, P32020, Q9CZU6, Q8BMS1, Q8BP40, Q8QZT1, Q9D6K5, P51881, Q811U4, Q78IK2, P56135, P99029, Q9CPQ3, Q9CPQ1, Q62465, Q3TL44, Q9CZD3, P09671, Q9QYR9, P00405, Q9EPB4, Q9CXT8, Q9JM90, Q8CGK3, P62908, Q6PB66, Q9CQN1, P28352, Q60931, Q9CQN6, P51174, Q14C51, Q6P3A8, Q8WTY4, Q9WUM5, Q9D172, P59017, P20108, P16332, P38060, Q78IK4, Q9Z110, P63038, Q9JK81, P29758, O35658	2.38533989100682	1.34137519601467E-10
UP_KEYWORDS	Transit peptide	37	2.2336412207831E-06	Q9D964, Q9R0X4, Q8BH95, P99029, P38647, Q9D880, Q3TL44, P36552, P09671, Q9QYR9, Q9CXT8, P56480, Q8CGK3, Q60597, Q6PB66, Q9CQN1, P51174, Q8BH86, Q14C51, Q6P3A8, Q03265, Q9WUM5, P19783, Q9D172, Q8BMF4, P20108, P16332, P38060, Q78IK4, Q9CZU6, P29758, P63038, Q9JK81, Q8BMS1, Q8BP40, O35658, Q8QZT1	2.40909482387391	2.02568152091709E-05

GOTERM_CC_DIRECT	GO:0005759~mitochondrial matrix	19	7.72778301956294E-05	Q60597, P62908, Q9CQN1, Q99LX0, Q8BH95, P51174, Q9WUM5, P35700, P16332, Q9CZN7, Q9QYR9, P38060, Q9CXT8, Q9CZU6, P29758, P63038, O35658, Q8QZT1, Q8CGK3	2.97027955347772	0.001008788972554
UP_SEQ_FEATURE	transit peptide:Mitochondrion	37	0.000102244176038	Q9D964, Q9R0X4, Q8BH95, P99029, Q921F4, P38647, Q9D880, Q3TL44, P36552, P09671, Q9QYR9, Q9CXT8, P56480, Q8CGK3, Q60597, Q6PB66, Q9CQN1, P51174, Q8BH86, Q14C51, Q6P3A8, Q03265, Q9WUM5, P19783, Q9D172, Q8BMF4, P20108, P16332, P38060, Q9CZU6, P29758, P63038, Q9JK81, Q8BMS1, O35658, Q8QZT1, Q9D6K5	2.00842615362361	0.049179448674241

**Annotation Cluster 8**	Enrichment Score: 6.165766723021288					
Category	Term	Count	PValue	Genes	Fold Enrichment	FDR
UP_KEYWORDS	Spliceosome	21	3.06737202152557E-10	Q9CSN1, Q99NB9, Q8JZX4, P61979, P62309, Q8BTI8, Q6PDM2, O35691, Q64012, Q6PE01, O08583, P16110, Q99KP6, P83870, O88569, Q922U1, P62305, P62317, P62307, O35737, Q8CCF0	5.96013064534294	5.37812561107483E-09
UP_KEYWORDS	mRNA splicing	28	4.39847371952314E-09	Q99NB9, Q8JZX4, Q8BTI8, O35691, Q9JKP5, Q9DAR7, Q8VIJ6, Q99KP6, P83870, P62317, Q9CSN1, P61979, P62309, Q6PDM2, O35286, Q64012, Q6PE01, P17225, P16110, O08583, Q3U0V1, O88569, Q922U1, P62305, P62307, O35737, Q8CCF0, O35658	3.87408491947291	6.42665882352547E-08

GOTERM_CC_DIRECT	GO:0005681~spliceosomal complex	21	3.4997401111078E-08	Q9CSN1, Q99NB9, Q8JZX4, P61979, Q8BTI8, Q6PDM2, O35286, O35691, Q64012, Q6PE01, O08583, P16110, Q99KP6, P83870, O88569, Q922U1, P62305, P62317, P62307, O35737, Q8CCF0	4.53818253758903	8.45187236832533E-07
UP_KEYWORDS	mRNA processing	29	2.14786621573615E-07	Q99NB9, Q8JZX4, Q8BTI8, O35691, Q9JKP5, Q9DAR7, Q8VIJ6, Q99KP6, P83870, P62317, Q9DBR1, Q9CSN1, P61979, P62309, Q6PDM2, O35286, Q64012, Q6PE01, P17225, P16110, O08583, Q3U0V1, O88569, Q922U1, P62305, P62307, O35737, Q8CCF0, O35658	3.13676489524563	2.25955525895443E-06
GOTERM_BP_DIRECT	GO:0008380~RNA splicing	27	7.64473833071557E-07	Q99NB9, Q8JZX4, Q8BTI8, O35691, Q9JKP5, Q9DAR7, Q8VIJ6, Q99KP6, P83870, P62317, Q9CSN1, P61979, Q6PDM2, O35286, Q64012, Q6PE01, P17225, P16110, O08583, Q3U0V1, O88569, Q922U1, P62305, P62307, O35737, Q8CCF0, O35658	3.07869944128441	0.000191604941616
GOTERM_CC_DIRECT	GO:0071013~catalytic step 2 spliceosome	16	8.09129785892731E-07	Q9CSN1, Q99NB9, P61979, P62309, Q8BTI8, Q6PDM2, O35691, Q64012, Q6PE01, Q99KP6, P83870, O88569, P62305, P62317, P62307, O35737	4.898355754858	1.50311417917765E-05

GOTERM_BP_DIRECT	GO:0006397~mRNA processing	32	8.39246876744904E-07	Q99NB9, Q8JZX4, Q8BTI8, Q921F4, O35691, Q9JKP5, Q9DAR7, Q8VIJ6, Q99KP6, P83870, Q05921, P60335, P62317, Q6NS46, Q9DBR1, Q9CSN1, P61979, Q6PDM2, O35286, Q64012, Q6PE01, P17225, P16110, O08583, Q3U0V1, O88569, Q922U1, P62305, P62307, O35737, Q8CCF0, O35658	2.73095584209632	0.000192816969932
KEGG_PATHWAY	mmu03040:Spliceosome	18	0.00080170833149	Q9CSN1, Q99NB9, Q8JZX4, P61979, P62309, Q6PDM2, O35286, Q6PE01, O08583, Q99KP6, P83870, Q922U1, P62305, Q810A7, P60335, P62317, P62307, Q8CCF0	2.49016080872037	0.010555826364615

**Annotation Cluster 9**	Enrichment Score: 6.142197552205322					
Category	Term	Count	PValue	Genes	Fold Enrichment	FDR

UP_KEYWORDS	Nucleotide-binding	105	1.54867158953322E-11	P07901, Q9CW03, O70133, P55264, P38647, Q922B2, P68181, Q61036, Q9WTP6, Q8VEH3, P16460, P61021, P61028, Q9JJZ4, P56480, Q9DBC7, P58389, Q03265, P10126, Q64430, P84096, O55143, Q6PCN7, Q8K183, Q811U4, Q80X41, Q5SUR0, Q9DCL9, P61161, Q8VDD5, Q9JKR6, Q9CZD3, Q8BMJ2, P26638, Q8CGK3, Q61820, Q60855, P68372, Q9WUM5, P61211, Q61390, Q04447, P60766, Q3UFY7, P11928, B2RQC6, Q922F4, Q8JZR0, Q9Z277, Q61879, O70172, Q9DBP5, B2RX12, P63001, Q9QWT9, P63085, Q9JHR7, Q9Z1F9, Q05921, Q810A7, P31938, P52480, P41216, P28650, P70296, P68033, O35286, P11499, P35293, P42932, Q8R050, Q68FH4, P09581, Q6Q899, P00493, Q501J6, O08528, P59325, Q9CU62, Q9JM14, P62331, P49717, Q3TL44, P68037, Q04899, P35285, Q9D6F9, O54988, G5E829, Q99KH8, P60710, Q62159, P80315, Q9CQN1, Q60931, P63280, Q9EQP2, P51859, P08113, Q9Z110, Q9WUA3, P63038, P08752, P60843, P80317	1.98784288912521	3.7027329822476E-10

GOTERM_MF_DIRECT	GO:0000166~nucleotide binding	126	2.79720723520736E-10	P07901, P52431, Q9CW03, O70133, P55264, P38647, Q922B2, P68181, Q61036, Q9WTP6, P62751, Q8VEH3, P16460, P61021, P61028, Q9JJZ4, P56480, Q9DBC7, P58389, Q03265, P10126, Q64430, P84096, O55143, Q6PCN7, O88569, Q8K183, Q9CX86, Q811U4, Q921F4, Q80X41, Q5SUR0, Q9DCL9, P61161, Q8VDD5, Q9JKR6, Q9CZD3, Q8BMJ2, P26638, Q8CGK3, O89086, Q61545, Q61820, Q60855, Q3THG9, P68372, Q9WUM5, Q64012, P61211, O08583, Q8C2Q3, Q61390, Q04447, P60766, Q3UFY7, B2RQC6, Q922F4, Q8JZR0, Q9Z277, Q60668, Q61879, O70172, P32067, P56959, Q9DBP5, B2RX12, P63001, P63085, Q9JHR7, Q9Z1F9, Q05921, Q810A7, P31938, P52480, P41216, P28650, P62849, P70296, P68033, Q6PDM2, O35286, P11499, P70333, P35293, P42932, Q8R050, Q68FH4, P09581, Q6Q899, P00493, Q8JZX4, Q9CY64, Q501J6, O08528, P59325, Q9CU62, Q9JM14, P62331, P49717, Q8VIJ6, Q5SF07, Q3TL44, P68037, Q04899, P35285, Q9D6F9, O54988, G5E829, Q99KH8, P60710, Q62159, P80315, Q9CQN1, Q60931, P63280, Q9EQP2, P17225, P51859, P08113, Q9Z110, Q9WUA3, O35737, P63038, P08752, P60843, P80317	1.7522095959596	4.57343382956403E-08

UP_KEYWORDS	ATP-binding	77	1.48283301634006E-07	P07901, P11928, Q9CW03, B2RQC6, O70133, Q8JZR0, Q9Z277, Q61879, P55264, O70172, P38647, Q922B2, P68181, Q61036, Q9WTP6, Q9DBP5, B2RX12, Q9QWT9, P63085, Q9JHR7, P16460, Q9Z1F9, Q05921, Q810A7, P31938, P52480, Q9JJZ4, P56480, P41216, P70296, P68033, P58389, O35286, P11499, Q03265, Q64430, P42932, O55143, Q68FH4, P09581, Q6PCN7, Q8K183, Q6Q899, Q80X41, Q501J6, O08528, Q9CU62, Q5SUR0, Q9DCL9, P61161, P49717, Q8VDD5, Q9JKR6, P68037, Q3TL44, Q9CZD3, Q8BMJ2, Q04899, O54988, P26638, G5E829, Q99KH8, Q8CGK3, P60710, P80315, Q9CQN1, Q60855, P63280, Q9EQP2, Q61390, Q04447, P08113, Q9Z110, P63038, Q9WUA3, P80317, P60843	1.87593253620845	1.62493784707265E-06

GOTERM_MF_DIRECT	GO:0005524~ATP binding	80	0.001027120190324	P07901, P11928, Q9CW03, B2RQC6, O70133, Q8JZR0, Q9Z277, Q61879, Q9Z0P5, P55264, O70172, P38647, Q922B2, P68181, Q61036, Q9WTP6, Q9DBP5, B2RX12, Q9QWT9, P63085, Q9JHR7, P16460, Q9Z1F9, Q05921, Q810A7, P31938, P52480, Q9JJZ4, P56480, P41216, P70296, P68033, P58389, O35286, P11499, Q03265, Q64430, P42932, O55143, Q68FH4, P09581, Q6PCN7, Q8K183, Q6Q899, Q80X41, Q501J6, O08528, Q9CU62, Q5SUR0, Q9DCL9, P61161, P49717, Q8VDD5, Q9JKR6, P68037, Q3TL44, Q9CZD3, Q8BMJ2, Q04899, O54988, P26638, G5E829, Q99KH8, Q8CGK3, P60710, P80315, Q9CQN1, Q8BH61, Q60855, Q3THG9, P63280, Q9EQP2, Q61390, Q04447, P08113, Q9Z110, P63038, Q9WUA3, P80317, P60843	1.42921510318104	0.029635438432579
UP_SEQ_FEATURE	nucleotide phosphate-binding region:ATP	40	0.294859246329712	Q9CW03, O70133, Q61879, Q80X41, Q501J6, Q9CU62, Q5SUR0, P68181, Q61036, Q9WTP6, Q9DBP5, Q9QWT9, P61161, P49717, Q8VDD5, Q3TL44, P63085, Q9CZD3, P16460, Q04899, Q9Z1F9, Q810A7, O54988, P31938, P26638, Q99KH8, P56480, Q8CGK3, Q60855, O35286, Q03265, Q9EQP2, Q68FH4, Q04447, Q6PCN7, P09581, Q8K183, Q6Q899, Q9WUA3, P60843	1.1183288397152	0.998961578400831

Annotation Cluster 10 Enrichment Score: 5.7152123414759926

Category	Term	Count	PValue	Genes	Fold Enrichment	FDR
UP_KEYWORDS	Redox-active center	14	8.05373772455301E-10	P27773, O08709, O08807, Q9CQM5, P99029, Q9JMH6, Q9ESY9, Q8R180, P35700, P20108, P08003, P09103, Q8VBT0, Q922R8	9.89128064546276	1.3238331384734E-08
GOTERM_BP_DIRECT	GO:0045454~cell redox homeostasis	15	6.68826112118315E-08	P27773, O08709, P28352, O08807, P99029, Q9JMH6, Q8BZW8, Q8R180, P35700, P20108, P08003, P09103, Q8VBT0, Q91VW3, Q922R8	6.34159457563713	2.04883732345577E-05
INTERPRO	IPR013766:Thioredoxin domain	11	4.41513040257316E-07	P27773, P20108, O08709, O08807, P99029, P08003, P09103, Q8VBT0, Q8BZW8, Q922R8, P35700	8.42760416666667	0.000296205197614
INTERPRO	IPR012336:Thioredoxin-like fold	19	4.61020075691933E-07	P27773, P10649, O08709, O08807, Q9CQM5, Q9Z1Q5, P99029, Q9JHF7, Q8BZW8, P35700, Q99L20, P20108, Q9JJU8, P08003, P09103, Q8VBT0, O09131, Q91VW3, Q922R8	4.28140318627451	0.000296205197614
GOTERM_CC_DIRECT	GO:0005623~cell	16	0.012793976700128	P27773, O08709, O08807, P99029, Q9Z0P5, Q9JMH6, O08528, P29391, P30681, Q9CPQ3, Q8BZW8, P35700, O55143, P09103, Q8VBT0, Q922R8	2.03568031370722	0.066446137055502

Annotation Cluster 11 Enrichment Score: 4.905648953988063

Category	Term	Count	PValue	Genes	Fold Enrichment	FDR

UP_KEYWORDS	Protein transport	45	1.83903272880863E-08	Q9EPK6, Q9WU78, P98078, P61924, Q8C2E7, Q9D8U8, Q9ER00, O70492, P70168, Q6P5F9, Q9CPQ3, Q9D662, Q9D880, Q6P8X1, P62331, P63024, O35643, Q01405, O35344, Q80XQ2, P35285, P61021, O35609, P61028, Q9D0F3, Q9D4H1, Q99PL5, Q91V04, Q61820, Q9CT10, Q8R0H9, Q8VDV3, P70670, Q61187, Q9D2V7, Q60770, Q9QZ88, P35293, Q9CWK8, O88746, P57780, P52293, P61971, O70404, O88384	2.58975718806063	2.3031695603651E-07
GOTERM_BP_DIRECT	GO:0015031~protein transport	46	2.60176315029035E-06	P50396, Q9EPK6, Q9WU78, P98078, P61924, Q8C2E7, Q9D8U8, Q9ER00, O70492, P70168, Q6P5F9, Q9CPQ3, Q9D662, Q9D880, Q6P8X1, P62331, P63024, O35643, Q8VDD5, Q01405, O35344, Q80XQ2, P35285, P61021, O35609, P61028, Q9D0F3, Q9D4H1, O88531, Q99PL5, Q91V04, Q61820, Q9CT10, Q8R0H9, Q8VDV3, P70670, Q61187, Q9D2V7, Q60770, Q9QZ88, P35293, Q9CWK8, O88746, P57780, O70404, O88384	2.13528916454448	0.000478204067023

UP_KEYWORDS	Transport	88	5.2812100515102E-05	P98078, Q9D8U8, Q9CR62, Q9ER00, O70492, Q6P5F9, P51863, O08788, Q3U7R1, Q62048, Q9D880, Q6P8X1, B2RX12, O35643, Q01405, Q9DC16, P61021, P61028, O35609, Q9DC50, Q9D4H1, P48962, P56480, Q8R1I1, Q99PL5, Q9CT10, Q8VDV3, Q62418, Q6PDM2, Q9CR51, Q03265, Q64430, Q99MR3, P53811, O08997, Q8R180, Q9DBG5, P35293, Q9CWK8, O55143, P57780, Q61093, P32020, P62482, O88569, P52293, O70404, P51881, Q9EPK6, Q9WU78, P61924, Q8C2E7, P56135, P70168, Q9CPQ3, Q9D662, P53986, P63024, P62331, O35344, Q5SF07, Q80XQ2, P50518, P35285, P00405, Q9D0F3, G5E829, Q9WU81, Q6PB66, Q8CIZ9, Q91V04, Q61820, Q60931, Q8R0H9, Q9Z1Q5, P70670, Q9D2V7, Q61187, Q60770, Q9QZ88, O08583, O88746, Q3U0V1, B9EJ86, Q8VBT0, P24668, P61971, O88384	1.53717354586436	0.000396845212442

Annotation Cluster 12 Enrichment Score: 4.691157205682

Category	Term	Count	PValue	Genes	Fold Enrichment	FDR
GOTERM_BP_DIRECT	GO:0005975~carbohydrate metabolic process	31	8.70903739332816E-11	Q7TSV4, P20060, Q9DCD0, P51569, O08528, Q9CPU0, P70665, Q93092, O54782, O88958, Q9QWR8, Q00612, Q6ZQI3, Q8BHN3, P23780, P06745, Q8CI94, P48441, Q8VDL4, Q8K2I4, P17439, Q8BMF4, P12265, O09159, P14152, P62141, P06151, Q8CHP8, Q9CZU6, Q91W89, P29416	4.13537639802874	4.00180268223429E-08

UP_KEYWORDS	Glycosidase	16	3.26209419155019E-08	P23780, P08905, P20060, P48441, P51569, Q8K2I4, P17439, P12265, O09159, O54782, Q80UM7, Q9QWR8, P58242, P29416, Q91W89, Q8BHN3	6.25062440788907	3.89968532898955E-07
GOTERM_MF_DIRECT	GO:0004553~hydrolase activity, hydrolyzing O-glycosyl compounds	12	8.09413410679671E-08	O09159, O54782, P23780, Q9QWR8, P20060, P48441, P51569, Q8K2I4, Q91W89, P29416, P12265, Q8BHN3	8.50194931773879	7.94034555876757E-06
GOTERM_MF_DIRECT	GO:0016798~hydrolase activity, acting on glycosyl bonds	16	1.46782698249589E-07	P23780, P08905, P20060, P48441, P51569, Q8K2I4, P17439, P12265, O09159, O54782, Q80UM7, Q9QWR8, P58242, P29416, Q91W89, Q8BHN3	5.52263374485597	1.19994855819039E-05
UP_SEQ_FEATURE	active site:Proton donor	23	8.06202178233286E-06	P23780, P06745, P20060, Q8VCT3, P48441, P51569, Q80W54, Q8K2I4, Q9JII6, P17439, P12265, P54822, P36552, Q9QWR8, P62141, P63087, P63328, P45376, P17182, P29416, Q8BP40, Q8BHN3, Q3UFY7	3.02071530132342	0.007755664954604
KEGG_PATHWAY	mmu00511:Other glycan degradation	8	2.47408093657801E-05	O09159, O54782, P23780, P20060, Q8K2I4, P17439, Q91W89, P29416	8.17756512493355	0.000651507979966
KEGG_PATHWAY	mmu00531:Glycosaminoglycan degradation	8	7.84163994165305E-05	Q571E4, Q8BFR4, P23780, P50429, P20060, P48441, P29416, P12265	7.00934153565733	0.001858468666172
INTERPRO	IPR017853:Glycoside hydrolase, superfamily	10	8.7114481131456E-05	P23780, Q9QWR8, P20060, P48441, P51569, Q8K2I4, P17439, P29416, P12265, Q8BHN3	5.37646198830409	0.031426549068173

Annotation Cluster 13 Enrichment Score: 4.5856942876524345

Category	Term	Count	PValue	Genes	Fold Enrichment	FDR
GOTERM_BP_DIRECT	GO:0051693~actin filament capping	8	1.81633611951342E-06	P13020, P24452, Q8VDD5, P47753, Q62261, P47754, P47757, P16546	12.2134414049308	0.00035768847725
UP_KEYWORDS	Actin capping	7	6.74206601508248E-06	P13020, P24452, P47753, Q62261, P47754, P47757, P16546	13.6732408922573	5.91054453988897E-05
GOTERM_BP_DIRECT	GO:0051016~barbed-end actin filament capping	7	1.31845834187184E-05	P13020, P24452, Q9Z0P5, Q62418, P47753, P47754, P47757	12.0226063829787	0.002184525827756

GOTERM_CC_DIRECT	GO:0008290~F-actin capping protein complex	4	0.002812983709333	P47753, P47754, P47757, P62774	13.0622820129547	0.017877251731681

Annotation Cluster 14 Enrichment Score: 3.914066305663755

Category	Term	Count	PValue	Genes	Fold Enrichment	FDR
INTERPRO	IPR013766:Thioredoxin domain	11	4.41513040257316E-07	P27773, P20108, O08709, O08807, P99029, P08003, P09103, Q8VBT0, Q8BZW8, Q922R8, P35700	8.42760416666667	0.000296205197614
GOTERM_MF_DIRECT	GO:0051920~peroxiredoxin activity	6	1.49083398402693E-05	P20108, O08709, O08807, Q99LX0, P99029, P35700	16.1537037037037	0.0008602989049
UP_KEYWORDS	Peroxidase	7	3.57439860805704E-05	P20108, P22437, O08709, O08807, P24270, P99029, P35700	10.565686144017	0.00027649024527
UP_KEYWORDS	Antioxidant	6	5.66238627812033E-05	P20108, P08228, O08709, O08807, P99029, P35700	13.2825768667643	0.000413668775318
UP_SEQ_FEATURE	active site:Cysteine sulfenic acid (-SOH) intermediate	5	6.00068577930158E-05	P20108, O08709, O08807, P99029, P35700	19.2312620115311	0.038484398131254
GOTERM_BP_DIRECT	GO:0042744~hydrogen peroxide catabolic process	6	9.50174591800331E-05	P20108, O08709, P24270, P99029, Q9JMH6, P35700	11.7772470690404	0.010478525398374
GOTERM_MF_DIRECT	GO:0004601~peroxidase activity	8	0.00011285302676	P20108, P22437, O08709, O08807, P24270, Q9CQM5, P99029, P35700	6.94782954998009	0.004813426923994
GOTERM_MF_DIRECT	GO:0016209~antioxidant activity	7	0.000193075381498	P20108, P08228, O08709, O08807, P24270, P99029, P35700	7.85249485596708	0.007576277969975
INTERPRO	IPR019479:Peroxiredoxin, C-terminal	4	0.000639815767172	P20108, O08709, O08807, P35700	20.4305555555556	0.083932195638973
INTERPRO	IPR000866:Alkyl hydroperoxide reductase subunit C/ Thiol specific antioxidant	4	0.001092568191176	P20108, O08709, O08807, P35700	17.5119047619048	0.103204087847055

Annotation Cluster 15 Enrichment Score: 3.814452000898943

Category	Term	Count	PValue	Genes	Fold Enrichment	FDR
GOTERM_BP_DIRECT	GO:0051603~proteolysis involved in cellular protein catabolic process	15	1.10392272473365E-08	P10605, O70435, P99026, P70195, O08529, O70370, P97821, Q920A5, Q9R1P4, Q9R1P3, Q9WUU7, P16675, Q9JHR7, O89017, Q8CGK3	7.23164293713006	4.34787850298668E-06

UP_KEYWORDS	Protease	32	0.000525737656081	Q11136, O70435, P56399, P99026, Q9CXY9, O35593, P34960, O08529, O70370, P97821, P18242, Q9R1P4, Q9R1P3, Q99K23, Q9JHR7, Q9CXT8, Q9Z2W0, Q8CGK3, O89001, O89023, Q64514, P10605, Q99LX0, P70195, Q6NSR8, Q8VCT3, Q80W54, Q920A5, Q9WUU7, P16675, O89017, Q7TMR0	1.96052795081392	0.003215558222078
GOTERM_MF_DIRECT	GO:0008233~peptidase activity	35	0.000891216029373	P27773, Q11136, O70435, P56399, P99026, Q9CXY9, O35593, P34960, O08529, O70370, P97821, P18242, Q9R1P4, Q9R1P3, Q99K23, Q6ZWQ7, Q9JHR7, Q9CXT8, Q9Z2W0, Q8CGK3, O89001, O89023, Q64514, P10605, P57716, Q99LX0, P70195, Q6NSR8, Q8VCT3, Q80W54, Q920A5, Q9WUU7, P16675, O89017, Q7TMR0	1.82616159441095	0.026493421964082
UP_KEYWORDS	Zymogen	16	0.002049034876484	P10605, P23780, Q8BH61, P99026, Q99LX0, P70195, P34960, O70370, P97821, P18242, Q9WUU7, P16675, O89017, Q7TMR0, P29416, O89023	2.4943806322562	0.010015147180821
GOTERM_BP_DIRECT	GO:0006508~proteolysis	34	0.007989137534611	Q11136, O70435, P56399, P99026, Q9CXY9, O35593, P34960, O08529, O70370, P97821, P18242, Q9R1P4, Q9R1P3, Q99K23, Q6ZWQ7, Q9JHR7, Q9CXT8, Q9Z2W0, Q8CGK3, O89001, O89023, Q64514, P10605, Q9CX56, Q99LX0, P70195, Q6NSR8, Q8VCT3, Q80W54, Q920A5, Q9WUU7, P16675, O89017, Q7TMR0	1.60537503002956	0.310226087083418

Annotation Cluster 16 Enrichment Score: 3.545308532416807

Category	Term	Count	PValue	Genes	Fold Enrichment	FDR
GOTERM_BP_DIRECT	GO:0006164~purine nucleotide biosynthetic process	7	5.63456530307712E-05	Q9CWJ9, P00493, P11928, P28650, Q5SUR0, P54822, Q9DCL9	9.61808510638298	0.00739737930504
UP_KEYWORDS	Purine biosynthesis	5	0.000466564322483	Q9CWJ9, P28650, Q5SUR0, P54822, Q9DCL9	12.7717085257349	0.002921581352693
GOTERM_BP_DIRECT	GO:0006189~'de novo' IMP biosynthetic process	4	0.000879634917006	Q9CWJ9, Q5SUR0, P54822, Q9DCL9	18.3201621073962	0.057741749194867

Annotation Cluster 17 Enrichment Score: 3.460460715819797

Category	Term	Count	PValue	Genes	Fold Enrichment	FDR
INTERPRO	IPR013766:Thioredoxin domain	11	4.41513040257316E-07	P27773, P20108, O08709, O08807, P99029, P08003, P09103, Q8VBT0, Q8BZW8, Q922R8, P35700	8.42760416666667	0.000296205197614
GOTERM_CC_DIRECT	GO:0005790~smooth endoplasmic reticulum	9	3.87986651946568E-06	P27773, P14211, Q9JKR6, O08807, P24369, P08003, P08113, P35564, Q922R8	9.12107623318386	6.46198458242044E-05
GOTERM_CC_DIRECT	GO:0034663~endoplasmic reticulum chaperone complex	6	2.89542023404383E-05	Q9JKR6, P24369, P08003, P09103, P08113, Q922R8	14.695067264574	0.000437027491576
INTERPRO	IPR005788:Disulphide isomerase	4	0.000639815767172	P27773, P08003, P09103, Q922R8	20.4305555555556	0.083932195638973
GOTERM_MF_DIRECT	GO:0003756~protein disulfide isomerase activity	6	0.000673473631979	P27773, P08003, P09103, Q8VBT0, Q922R8, Q8R180	8.07685185185185	0.022781987343851
GOTERM_CC_DIRECT	GO:0005788~endoplasmic reticulum lumen	12	0.000815209842803	P27773, P14211, Q9EPK6, Q9JKR6, P08905, P24369, P08003, P09103, P08113, Q8K297, Q922R8, Q9DBS1	3.39116936874784	0.006907830773226

Annotation Cluster 18 Enrichment Score: 3.2542349881549804

Category	Term	Count	PValue	Genes	Fold Enrichment	FDR
SMART	SM00102:ADF	5	0.000171828436358	Q9Z0P5, Q62418, Q9CQI6, P18760, Q9ERL7	15.8917682926829	0.042613452216732
INTERPRO	IPR002108:Actin-binding, cofilin/tropomyosin type	5	0.000200516155534	Q9Z0P5, Q62418, Q9CQI6, P18760, Q9ERL7	15.3229166666667	0.041334973205176

Annotation Cluster 19 Enrichment Score: 3.198537748986291

Category	Term	Count	PValue	Genes	Fold Enrichment	FDR

UP_KEYWORDS	Lipid metabolism	29	7.08962305624729E-05	P51660, Q9Z0J0, O08709, P19096, Q8JZR0, Q8BH95, Q8BYI6, Q9JHF7, Q8BLF1, Q9DCN2, P22437, Q8K2C9, P58044, P58242, Q924Z4, Q9DC50, P41216, Q8VEB4, Q9Z0M5, P51174, P17439, Q61263, Q9D6K9, Q8CIH5, Q3TCN2, O70503, Q8BMS1, Q8BP40, Q8BG07	2.30932091808252	0.000503938071295
GOTERM_BP_DIRECT	GO:0006629~lipid metabolic process	34	0.000160978360042	P51660, Q9Z0J0, Q9WV54, O08709, P19096, Q8JZR0, Q8BH95, Q8BYI6, Q9JHF7, P53986, Q8BLF1, Q9DCN2, P22437, Q8K2C9, P58242, Q924Z4, Q9DC50, P56480, P41216, Q8VEB4, Q9Z0M5, Q61207, P51174, Q03265, P17439, Q61263, Q9D6K9, Q8CIH5, P38060, Q3TCN2, O70503, Q8BMS1, Q8BP40, Q8BG07	2.03557356748846	0.015850619237021
UP_KEYWORDS	Fatty acid metabolism	12	0.001311750192557	P51660, P22437, Q8K2C9, Q8BH95, P19096, Q8JZR0, P51174, Q9DC50, Q9JHF7, Q8BMS1, P41216, Q8VEB4	3.21352666131394	0.007113486216652
KEGG_PATHWAY	mmu01212:Fatty acid metabolism	10	0.001478650610093	Q8K2C9, Q8BH95, P19096, Q8JZR0, P51174, O88531, O70503, Q8BMS1, P41216, Q8QZT1	3.60774931982362	0.017522009729601

Annotation Cluster 20 Enrichment Score: 3.1326555888652114

Category	Term	Count	PValue	Genes	Fold Enrichment	FDR
UP_KEYWORDS	Glycolysis	9	7.35811897119128E-06	Q60597, P06745, Q8VDL4, P05063, P52480, P05064, O08528, P17182, Q9WUA3	8.53879941434846	6.24253319168809E-05
GOTERM_BP_DIRECT	GO:0006096~glycolytic process	9	3.57378297498019E-05	Q60597, P06745, Q8VDL4, P05063, P52480, P05064, O08528, P17182, Q9WUA3	6.87006079027356	0.005185747190537

KEGG_PATHWAY	mmu00010:Glycolysis / Gluconeogenesis	13	0.000187533369165	P06745, Q7TSV4, Q8VDL4, O08528, Q9JII6, Q8BMF4, Q9JLJ2, P06151, P05063, P52480, P05064, P17182, Q9WUA3	3.62414818036828	0.003534988602134

Annotation Cluster 21 Enrichment Score: 2.8938082139705914

Category	Term	Count	PValue	Genes	Fold Enrichment	FDR
GOTERM_BP_DIRECT	GO:0042554~superoxide anion generation	6	2.40159822513707E-05	Q8CIZ9, P08228, P09671, Q61093, O70145, Q09014	14.989223542415	0.003678447948168
GOTERM_BP_DIRECT	GO:0006801~superoxide metabolic process	6	0.00047448046668	Q8CIZ9, P08228, P09671, Q61093, O70145, A2AAY5	8.67797152455607	0.032703566165926
GOTERM_MF_DIRECT	GO:0016175~superoxide-generating NADPH oxidase activity	4	0.002472439390293	Q8CIZ9, Q61093, O70145, Q09014	13.4614197530864	0.06382797478626

Annotation Cluster 22 Enrichment Score: 2.7308178848835514

Category	Term	Count	PValue	Genes	Fold Enrichment	FDR
UP_KEYWORDS	RNA-binding	50	2.97848894347024E-10	P62918, P11928, O70133, Q60668, Q6P5F9, P56959, O35130, P32067, Q61990, Q9JKP5, P62751, Q05921, P60335, Q810A7, Q99LX0, Q6PDM2, P70333, O88569, Q6Q899, Q80SW1, Q9CX86, Q8JZX4, Q921F4, Q501J6, Q8VDM6, Q8VIJ6, Q5SF07, A2RSY6, O89086, P62908, Q6PB66, Q61545, P61979, P28352, P62309, P28271, Q14C51, Q64012, P14148, P17225, Q9CQE8, O08583, Q8C2Q3, Q3U0V1, P62305, P62702, P62307, O35737, Q8CCF0, P60843	2.76259918193933	5.37812561107483E-09

GOTERM_MF_DIRECT	GO:0003723~RNA binding	58	7.81979743308571E-07	P62918, O70133, Q60668, Q6P5F9, O35130, P32067, O35295, Q61990, Q9JKP5, P62751, Q05921, P60335, Q810A7, Q60872, Q60710, Q99LX0, Q6PDM2, P70333, P14131, O88569, Q6Q899, P60867, P62843, Q80SW1, Q9CX86, Q8JZX4, Q921F4, Q501J6, Q8VDM6, Q8VIJ6, Q5SF07, Q8BMJ3, Q6NS46, A2RSY6, O89086, P62908, Q6PB66, Q61545, P61979, P28352, P62309, P28271, Q14C51, Q64012, P14148, P17225, Q9CQE8, O08583, Q8C2Q3, Q3U0V1, P62305, P62702, P08113, P17182, P62307, O35737, Q8CCF0, P60843	2.00195473251029	5.90093944758237E-05

Annotation Cluster 23 Enrichment Score: 2.4938811585669423

Category	Term	Count	PValue	Genes	Fold Enrichment	FDR
KEGG_PATHWAY	mmu04146:Peroxisome	14	0.000475292060846	P51660, P08228, P24270, Q8JZR0, P99029, P35700, Q8C0L6, P09671, P32020, O88844, Q9DC50, P38060, P41216, Q00519	3.10353375223382	0.007040263651284
GOTERM_CC_DIRECT	GO:0005777~peroxisome	14	0.000526243044691	P51660, P08228, P24270, P99029, Q8C0L6, P40142, P58044, Q9JHR7, P32020, O88844, Q9DC50, P38060, P41216, Q00519	3.1409304076952	0.004538846260456
UP_KEYWORDS	Peroxisome	10	0.004997766324026	P51660, Q8C0L6, P58044, P24270, P32020, P99029, Q9DC50, P38060, P41216, Q00519	3.10340580999165	0.020537695987794

Annotation Cluster 24 Enrichment Score: 2.4570448232814757

Category	Term	Count	PValue	Genes	Fold Enrichment	FDR

GOTERM_BP_DIRECT	GO:0002479~antigen processing and presentation of exogenous peptide antigen via MHC class I, TAP-dependent	10	7.53599642198257E-07	P06339, O70435, P99026, P70195, Q9R233, Q9R1P4, Q9R1P3, P14211, P01902, P01901, P01900, P01899, P01896	9.16008105369808	0.000191604941616
UP_KEYWORDS	Proteasome	9	0.000254831682552	O70435, Q9CX56, P99026, P70195, O35593, Q9R1P4, Q9WVJ2, Q9R1P3, P14685	5.33674963396779	0.001675518312782
GOTERM_CC_DIRECT	GO:0000502~proteasome complex	9	0.001720850171175	O70435, Q9CX56, P99026, P70195, O35593, Q9R1P4, Q9WVJ2, Q9R1P3, P14685	4.00774561761109	0.012405531831011
KEGG_PATHWAY	mmu03050:Proteasome	9	0.002560176992409	O70435, Q9CX56, P99026, P70195, O35593, Q9R1P4, Q9WVJ2, Q9R1P3, P14685	3.6799043062201	0.027056953748162
GOTERM_CC_DIRECT	GO:0008541~proteasome regulatory particle, lid subcomplex	4	0.002812983709333	Q9CX56, O35593, Q9WVJ2, P14685	13.0622820129547	0.017877251731681
UP_KEYWORDS	Threonine protease	5	0.003849988704127	O70435, P99026, P70195, Q9R1P4, Q9R1P3	7.54691867429788	0.016801834184912
GOTERM_CC_DIRECT	GO:0005839~proteasome core complex	5	0.004144035343441	O70435, P99026, P70195, Q9R1P4, Q9R1P3	7.347533632287	0.024710729270147
GOTERM_CC_DIRECT	GO:0022624~proteasome accessory complex	4	0.018604184992103	Q9CX56, O35593, Q9WVJ2, P14685	6.91532577156423	0.087240983992095

Annotation Cluster 25 Enrichment Score: 2.3999624174705922

Category	Term	Count	PValue	Genes	Fold Enrichment	FDR
UP_KEYWORDS	Immunity	27	0.000222186741649	P06339, P11928, Q9CQW9, P10810, P34884, Q9R1P4, Q9ESY9, P30681, Q61990, Q3TL44, Q9QUN7, P58242, P01899, Q9EPB4, P01896, P04441, Q60710, P26151, Q62418, P42082, Q9R0Q8, Q9ES52, P63158, P16110, P01902, P01901, P09581, P01900, Q6Q899, O35658	2.23584523318351	0.001498336232144
UP_KEYWORDS	Innate immunity	17	0.0027085079929	P11928, Q60710, Q9CQW9, P26151, P10810, P34884, P30681, Q61990, P63158, Q3TL44, P16110, Q9QUN7, P58242, P09581, Q9EPB4, Q6Q899, O35658	2.34236314040449	0.012281682795393

Annotation Cluster 26 Enrichment Score: 2.3865683902433714

Category	Term	Count	PValue	Genes	Fold Enrichment	FDR
INTERPRO	IPR020575:Heat shock protein Hsp90, N-terminal	4	0.000134408604693	Q9CQN1, P07901, P08113, P11499	30.6458333333333	0.032325269428778
INTERPRO	IPR001404:Heat shock protein Hsp90	4	0.000134408604693	Q9CQN1, P07901, P08113, P11499	30.6458333333333	0.032325269428778
PIR_SUPERFAMILY	PIRSF002583:heat shock protein, HSP90/HTPG types	4	0.00095913543333	Q9CQN1, P07901, P08113, P11499	15.5775862068966	0.098790949632966
UP_KEYWORDS	Stress response	8	0.007305942439949	O89086, P07901, Q9JKR6, Q99LX0, E9PVA8, P08113, P11499, Q3UW53	3.54202049780381	0.028256806789803

Annotation Cluster 27 Enrichment Score: 2.281301357330428

Category	Term	Count	PValue	Genes	Fold Enrichment	FDR
GOTERM_BP_DIRECT	GO:0006099~tricarboxylic acid cycle	7	0.000522502974117	Q60597, P14152, O88844, P28271, Q9WUM5, Q9CZU6, Q8BMF4	6.63316214233309	0.035135139015643
UP_KEYWORDS	Tricarboxylic acid cycle	6	0.000780759226329	P14152, O88844, P28271, Q9WUM5, Q9CZU6, Q8BMF4	7.96954612005857	0.004666810830105
KEGG_PATHWAY	mmu00020:Citrate cycle (TCA cycle)	7	0.006667977218735	Q60597, P14152, O88844, P28271, Q9WUM5, Q9CZU6, Q8BMF4	4.02489533492823	0.060405137629932

Annotation Cluster 28 Enrichment Score: 2.2424899481164733

Category	Term	Count	PValue	Genes	Fold Enrichment	FDR
UP_KEYWORDS	Viral nucleoprotein	5	0.003849988704127	P61979, Q60668, O88569, P62317, O35737	7.54691867429788	0.016801834184912
GOTERM_CC_DIRECT	GO:0019013~viral nucleocapsid	5	0.005929469948427	P61979, Q60668, O88569, P62317, O35737	6.67957602935181	0.033693341001062
UP_KEYWORDS	Virion	5	0.008204551171474	P61979, Q60668, O88569, P62317, O35737	6.1493411420205	0.030825670829966

Annotation Cluster 29 Enrichment Score: 2.1662029833598906

Category	Term	Count	PValue	Genes	Fold Enrichment	FDR
KEGG_PATHWAY	mmu00511:Other glycan degradation	8	2.47408093657801E-05	O09159, O54782, P23780, P20060, Q8K2I4, P17439, Q91W89, P29416	8.17756512493355	0.000651507979966

Annotation Cluster 30 Enrichment Score: 2.07072484761341

Category	Term	Count	PValue	Genes	Fold Enrichment	FDR
INTERPRO	IPR017998:Chaperone tailless complex polypeptide 1 (TCP-1)	5	0.000306984068067	P80315, P42932, Q61390, P63038, P80317	13.9299242424242	0.055372251277508
INTERPRO	IPR027409:GroEL-like apical domain	5	0.000861248576412	P80315, P42932, Q61390, P63038, P80317	10.9449404761905	0.095598591981778
INTERPRO	IPR027413:GroEL-like equatorial domain	5	0.000861248576412	P80315, P42932, Q61390, P63038, P80317	10.9449404761905	0.095598591981778
GOTERM_CC_DIRECT	GO:0005832~chaperonin-containing T-complex	4	0.002812983709333	P80315, P42932, Q61390, P80317	13.0622820129547	0.017877251731681

Annotation Cluster 31 Enrichment Score: 2.003328340425609

Category	Term	Count	PValue	Genes	Fold Enrichment	FDR
KEGG_PATHWAY	mmu00531:Glycosaminoglycan degradation	8	7.84163994165305E-05	Q571E4, Q8BFR4, P23780, P50429, P20060, P48441, P29416, P12265	7.00934153565733	0.001858468666172

Annotation Cluster 32 Enrichment Score: 1.9787696369460814

Category	Term	Count	PValue	Genes	Fold Enrichment	FDR
GOTERM_CC_DIRECT	GO:0005687~U4 snRNP	5	0.000235429343554	P62309, P62305, P62317, P62307, Q8CCF0	14.695067264574	0.00236900776951
GOTERM_CC_DIRECT	GO:0005689~U12-type spliceosomal complex	7	0.000241666320848	Q99NB9, P62309, P83870, P62305, O35286, P62317, P62307	7.61966450755689	0.002382139448356
GOTERM_CC_DIRECT	GO:0071011~precatalytic spliceosome	6	0.002289483983356	P62309, P83870, P62305, P62317, Q8CCF0, Q6PE01	6.29788597053171	0.015574940337477
GOTERM_CC_DIRECT	GO:0005686~U2 snRNP	5	0.004144035343441	Q99NB9, P62309, P83870, P62305, P62317	7.347533632287	0.024710729270147
GOTERM_CC_DIRECT	GO:0034709~methylosome	4	0.00682715253876	P62309, P62305, P62317, P62307	9.79671150971599	0.037902467542772
GOTERM_CC_DIRECT	GO:0034719~SMN-Sm protein complex	4	0.015710570298598	P62309, P62305, P62317, P62307	7.347533632287	0.07624255617666
GOTERM_CC_DIRECT	GO:0034715~pICln-Sm protein complex	3	0.015785208318149	P62305, P62317, P62307	14.695067264574	0.07624255617666
GOTERM_CC_DIRECT	GO:0005682~U5 snRNP	4	0.018604184992103	P62309, P62305, P62317, Q6PE01	6.91532577156423	0.087240983992095
GOTERM_CC_DIRECT	GO:0005683~U7 snRNP	3	0.021605933643224	P62309, P62305, P62307	12.5957719410634	0.098276508655177

Annotation Cluster 33 Enrichment Score: 1.8660316202385918

Category	Term	Count	PValue	Genes	Fold Enrichment	FDR
GOTERM_CC_DIRECT	GO:0030904~retromer complex	9	5.30986390685791E-07	Q9QZ88, Q6P8X1, Q9CWK8, Q9D8U8, Q80XQ2, O70492, P24668, O08788, Q810B6	11.5004874244492	1.06861011125515E-05

GOTERM_CC_DIRECT	GO:0019898~extrinsic component of membrane	9	0.008239295415784	Q6P8X1, Q6ZQA0, Q9CWK8, Q9D8U8, P32020, O70311, O70492, Q8VBT9, Q09014	3.1118965972039	0.044714378492402

Annotation Cluster 34 Enrichment Score: 1.781958596890396

Category	Term	Count	PValue	Genes	Fold Enrichment	FDR
GOTERM_MF_DIRECT	GO:0004579~dolichyl-diphosphooligosaccharide-protein glycotransferase activity	5	0.000203458720665	P46978, P61804, Q91YQ5, O54734, Q9DBG6	14.9571330589849	0.007676654037381
GOTERM_CC_DIRECT	GO:0008250~oligosaccharyltransferase complex	5	0.000235429343554	P46978, P61804, Q91YQ5, O54734, Q9DBG6	14.695067264574	0.00236900776951

Annotation Cluster 35 Enrichment Score: 1.781875783533012

Category	Term	Count	PValue	Genes	Fold Enrichment	FDR
GOTERM_CC_DIRECT	GO:0030863~cortical cytoskeleton	10	5.89128343535373E-07	Q7TPR4, Q8VDD5, P57780, P47753, Q62261, P47754, P47757, P16546, P60710, Q9ES52	9.48068855778967	1.13819595971034E-05
GOTERM_BP_DIRECT	GO:0051017~actin filament bundle assembly	8	0.000224036025549	Q7TPR4, P57780, Q3V0K9, O70200, Q62418, Q9WUM3, Q62188, P60766	6.28119843682154	0.018166685954084

Annotation Cluster 36 Enrichment Score: 1.6023945208006458

Category	Term	Count	PValue	Genes	Fold Enrichment	FDR
UP_KEYWORDS	GTP-binding	22	0.001189061298016	Q62159, Q61820, Q811U4, P28650, Q922F4, Q9WUM5, P10126, P68372, P59325, P84096, P63001, P35293, P62331, Q8R050, P61211, Q8VEH3, P35285, Q9D6F9, P61021, P61028, P08752, P60766	2.20042689057842	0.006688565786357
GOTERM_MF_DIRECT	GO:0003924~GTPase activity	18	0.00207486459923	Q61599, Q61820, Q811U4, P28650, Q922F4, P10126, P68372, P84096, P63001, P62331, Q8R050, P63213, P61211, P35285, Q9D6F9, P61021, P08752, P60766	2.3187134502924	0.058155490624123
UP_KEYWORDS	Prenylation	11	0.020566549153629	P84096, P63001, Q9DAS9, Q62159, P35293, P63213, P35285, P61021, P61028, P14733, P60766	2.32656601169438	0.067612530342554

Annotation Cluster 37 Enrichment Score: 1.5747501440414309

Category	Term	Count	PValue	Genes	Fold Enrichment	FDR
GOTERM_CC_DIRECT	GO:0070688~MLL5-L complex	5	0.000145157927238	Q61191, P62141, P63087, Q8CGY8, P60710	16.3278525161933	0.00163049485712

Annotation Cluster 38 Enrichment Score: 1.5134485538870548

Category	Term	Count	PValue	Genes	Fold Enrichment	FDR
UP_KEYWORDS	Protein biosynthesis	15	0.000145305560279	Q60872, Q99JX4, P10711, Q3THG9, O70251, P10126, Q922B2, P59325, Q8R050, Q9CZD3, Q8BMJ2, Q8BMJ3, P26638, Q8QZY1, P60843	3.38841246601129	0.001005667430349

Annotation Cluster 39 Enrichment Score: 1.4601464293271709

Category	Term	Count	PValue	Genes	Fold Enrichment	FDR
UP_KEYWORDS	Fatty acid metabolism	12	0.001311750192557	P51660, P22437, Q8K2C9, Q8BH95, P19096, Q8JZR0, P51174, Q9DC50, Q9JHF7, Q8BMS1, P41216, Q8VEB4	3.21352666131394	0.007113486216652
KEGG_PATHWAY	mmu01212:Fatty acid metabolism	10	0.001478650610093	Q8K2C9, Q8BH95, P19096, Q8JZR0, P51174, O88531, O70503, Q8BMS1, P41216, Q8QZT1	3.60774931982362	0.017522009729601

Annotation Cluster 40 Enrichment Score: 1.4463498546439604

Category	Term	Count	PValue	Genes	Fold Enrichment	FDR
UP_KEYWORDS	Cell division	23	0.002139167221713	P62908, Q9WU78, Q9CW03, Q68FD5, Q80X41, P63280, Q8VDQ8, Q61187, Q9CU62, Q8VE37, P62331, Q4VA53, Q9QWT9, A2AGT5, O35685, Q8VEH3, P62141, P63087, Q9WVA3, Q8BK67, P25322, P08752, P60766	2.05308647806168	0.010015147180821

UP_KEYWORDS	Mitosis	15	0.025587137840184	P62908, Q9CW03, Q68FD5, Q80X41, P63280, Q8VDQ8, Q9CU62, Q8VE37, Q4VA53, Q9QWT9, A2AGT5, O35685, Q8VEH3, Q9WVA3, Q8BK67	1.92315302124965	0.083079225332944

Annotation Cluster 41 Enrichment Score: 1.4455353973543603

Category	Term	Count	PValue	Genes	Fold Enrichment	FDR
GOTERM_CC_DIRECT	GO:0030136~clathrin-coated vesicle	7	0.014734248736542	P63024, Q99KN9, Q91ZX7, P98078, Q68FD5, O70492, Q7M6Y3	3.48696511362773	0.072873607387452

Annotation Cluster 42 Enrichment Score: 1.4368617249451718

Category	Term	Count	PValue	Genes	Fold Enrichment	FDR
UP_KEYWORDS	FAD	10	0.009341318687005	Q9DCN2, Q3U4I7, Q8CIZ9, Q8C0L6, Q61093, P51174, Q9JMH6, P52019, Q8R180, Q00519	2.81410526838226	0.034602349502568
UP_KEYWORDS	Flavoprotein	10	0.016821360514084	Q9DCN2, Q3U4I7, Q8CIZ9, Q8C0L6, Q61093, P51174, Q9JMH6, P52019, Q8R180, Q00519	2.55434170514698	0.056718177118001

Annotation Cluster 43 Enrichment Score: 1.4259918037079657

Category	Term	Count	PValue	Genes	Fold Enrichment	FDR
GOTERM_BP_DIRECT	GO:0006754~ATP biosynthetic process	6	0.001217322206711	O55126, P56135, P52480, P05064, Q03265, P56480	7.16875908550284	0.076276302815929
UP_KEYWORDS	Hydrogen ion transport	6	0.010970477085582	P50518, P56135, Q9CR51, Q03265, P51863, P56480	4.42752562225476	0.039523773609701
UP_KEYWORDS	ATP synthesis	3	0.05656345916408	P56135, Q03265, P56480	7.66302511544093	0.152777262783986

Annotation Cluster 44 Enrichment Score: 1.3856913094370495

Category	Term	Count	PValue	Genes	Fold Enrichment	FDR
BIOCARTA	m_eradPathway:ERï¿½associated degradation (ERAD) Pathway	7	0.000353960558584	O09159, Q80UM7, Q9R1T2, Q9Z1F9, P63280, P35564, Q8BHN3	6.39929078014184	0.063004979427878

Annotation Cluster 45 Enrichment Score: 1.2670232088698443

Category	Term	Count	PValue	Genes	Fold Enrichment	FDR
UP_KEYWORDS	Hydrogen ion transport	6	0.010970477085582	P50518, P56135, Q9CR51, Q03265, P51863, P56480	4.42752562225476	0.039523773609701

Annotation Cluster 46 Enrichment Score: 1.2388107932329662

Category	Term	Count	PValue	Genes	Fold Enrichment	FDR
GOTERM_CC_DIRECT	GO:0030136~clathrin-coated vesicle	7	0.014734248736542	P63024, Q99KN9, Q91ZX7, P98078, Q68FD5, O70492, Q7M6Y3	3.48696511362773	0.072873607387452

Annotation Cluster 47 Enrichment Score: 1.2069771182148312

Category	Term	Count	PValue	Genes	Fold Enrichment	FDR
UP_KEYWORDS	Biological rhythms	10	0.01396167124391	O09106, P23198, Q8VIJ6, Q60668, O70133, P62141, P63087, Q8CGY8, Q04750, Q9WTK5	2.63543191800878	0.048314730751952

Annotation Cluster 48 Enrichment Score: 1.1885792630607002

Category	Term	Count	PValue	Genes	Fold Enrichment	FDR
UP_KEYWORDS	GTPase activation	13	0.003897003366082	Q8BYA0, P50396, Q61599, Q5SSL4, Q61210, Q8BMG7, Q640N3, Q3TBD2, Q80XQ2, Q8BYW1, Q6PFQ7, Q5FWK3, Q8BQZ4	2.6483665531892	0.016801834184912

Annotation Cluster 49 Enrichment Score: 1.1209470651979352

Category	Term	Count	PValue	Genes	Fold Enrichment	FDR
KEGG_PATHWAY	mmu04662:B cell receptor signaling pathway	13	0.000332881259704	Q60631, P27870, P29351, Q60591, Q9EQ32, Q9ES52, P63001, P63085, Q8CIH5, P63328, P08101, P25799, P31938	3.41705399863295	0.005635204182133

Annotation Cluster 50 Enrichment Score: 1.062168927358502

Category	Term	Count	PValue	Genes	Fold Enrichment	FDR

UP_KEYWORDS	Metal-binding	131	0.001834345626021	Q7TSV4, P12815, P55264, P34960, P11688, Q3U7R1, Q61036, P63328, O89023, Q60597, Q8VCT3, P50428, Q64430, Q61164, P50543, O55023, P14211, O55143, P50429, O88844, Q6PCN7, Q8K183, E9Q555, Q3V0K9, Q8BYI6, Q6PB93, Q5SUR0, O54782, P22437, P40142, P58044, Q6PFQ7, Q80VA0, Q9Z0S1, Q9D0F3, Q9Z2W0, A2RSY6, Q9DBR1, O89001, P05555, Q61545, Q8BH61, Q3THG9, Q6NSR8, P11835, P28271, Q8VDL4, Q8WTY4, P70158, O08739, O09159, Q3TBD2, Q9QZ88, P16332, P61211, P62141, P38060, E9Q1P8, Q810B6, Q3UFY7, P81117, Q8BFR4, Q11136, P11928, Q91ZX7, B2RQC6, P10711, Q9Z277, P56399, P70340, P56959, Q9JKP5, Q9JHR7, P63087, Q9Z1F9, Q05921, P52480, Q91V76, Q00519, Q60710, P28650, O70200, Q8VHR5, P97315, P35564, P16546, O08997, Q9WVQ5, P57780, Q61093, Q99N69, Q6Q899, Q91W89, Q8QZT1, P08775, P00493, Q7TPR4, Q9CY64, O08529, O35593, P11103, Q8VDQ8, P29391, Q9CPU0, Q9JM14, Q571E4, P07091, P09671, P58242, P00405, Q9CXT8, G5E829, Q99KH8, Q8CIZ9, P08228, P28352, P24270, Q62318, Q80W54, Q9EQP2, P14069, P13020, Q8CHP8, Q8C9B9,	1.28130896137417	0.009472941329794

GOTERM_MF_DIRECT GO:0046872~metal ion binding	128	0.418953973596052	Q7TSV4, P12815, P55264, P34960, P11688, Q3U7R1, Q61036, P63328, O89023, Q60597, Q8VCT3, P50428, Q64430, Q61164, P50543, O55023, P14211, O55143, P50429, O88844, Q6PCN7, Q8K183, E9Q555, Q3V0K9, Q8BYI6, Q6PB93, Q5SUR0, O54782, P22437, P40142, P58044, Q6PFQ7, Q80VA0, Q9Z0S1, Q9D0F3, Q9Z2W0, A2RSY6, Q9DBR1, O89001, P05555, Q61545, Q8BH61, Q3THG9, Q6NSR8, P11835, P28271, Q8VDL4, Q8WTY4, P70158, O08739, O09159, Q3TBD2, Q9QZ88, P16332, P61211, P62141, P38060, E9Q1P8, Q810B6, Q3UFY7, P81117, Q8BFR4, Q11136, P11928, Q91ZX7, B2RQC6, P10711, Q9Z277, P56399, P70340, Q9JKP5, Q9JHR7, P63087, Q9Z1F9, Q05921, P52480, Q91V76, Q00519, Q60710, P28650, O70200, Q8VHR5, P97315, P35564, P16546, O08997, Q9WVQ5, P57780, Q61093, Q99N69, Q6Q899, Q91W89, Q8QZT1, P08775, P00493, Q7TPR4, Q9CY64, O08529, O35593, P11103, Q8VDQ8, Q9CPU0, Q9JM14, Q571E4, P07091, P09671, P58242, P00405, Q9CXT8, G5E829, Q99KH8, Q8CIZ9, P08228, P28352, P24270, Q62318, Q80W54, Q9EQP2, P14069, P13020, Q8CHP8, Q8C9B9, Q9WUA3,	1.02716049382716	0.970326409495549

UP_KEYWORDS	Zinc	57	0.846926526495965	Q62523, P27870, P52431, P10711, B2RQC6, P56399, Q9Z277, P70340, P34960, P56959, Q9JKP5, Q9JHR7, Q9Z1F9, Q05921, P63328, Q91V76, Q60710, Q8VCT3, Q8VHR5, P97315, Q61164, Q9WVQ5, P14211, Q99N69, Q6PCN7, Q8K183, Q6Q899, Q91W89, P08775, E9Q555, Q9CY64, O35593, P11103, Q8VDQ8, Q9CPU0, O54782, P58242, Q6PFQ7, Q9CXT8, Q9Z2W0, A2RSY6, Q9DBR1, O89001, P08228, Q61545, Q62318, Q3THG9, Q6NSR8, Q80W54, P70158, O08739, O09159, Q3TBD2, Q9QZ88, Q8C9B9, E9Q1P8, Q810B6	0.901747119349171	0.846926526495965

Annotation Cluster 57 Enrichment Score: 1.0227297981554944

Category	Term	Count	PValue	Genes	Fold Enrichment	FDR
GOTERM_BP_DIRECT	GO:1903076~regulation of protein localization to plasma membrane	4	0.001497825732521	Q62261, O70404, O70439, O88384	15.7029960920538	0.089771859664376

Annotation Cluster 58 Enrichment Score: 0.9341811400985971

Category	Term	Count	PValue	Genes	Fold Enrichment	FDR
UP_KEYWORDS	Hydrogen ion transport	6	0.010970477085582	P50518, P56135, Q9CR51, Q03265, P51863, P56480	4.42752562225476	0.039523773609701

Annotation Cluster 59 Enrichment Score: 0.868076369880284

Category	Term	Count	PValue	Genes	Fold Enrichment	FDR
UP_KEYWORDS	Carbohydrate metabolism	9	0.006514340407497	O88958, Q7TSV4, Q00612, Q8CI94, P62141, P63087, Q8CHP8, Q8BMF4, Q6ZQI3	3.24845629893692	0.02557121682346

Annotation Cluster 61 Enrichment Score: 0.7602670794175364

Category	Term	Count	PValue	Genes	Fold Enrichment	FDR

GOTERM_BP_DIRECT	GO:0006911~phagocytosis, engulfment	9	0.000190838852699	P21956, P63001, P13020, Q8VDD5, P26151, O70200, P08508, P08101, Q8BPU7	5.49604863221885	0.016269619470188
GOTERM_BP_DIRECT	GO:0050766~positive regulation of phagocytosis	8	0.001630951317362	P21956, P14211, P26151, Q5SSL4, P08508, P08101, Q9EPB4, Q8C3J5	4.58004052684904	0.095670910254638
UP_KEYWORDS	IgG-binding protein	3	0.005203542334819	P26151, P08508, P08101	24.904831625183	0.02105433283165

**Annotation Cluster 62**	Enrichment Score: 0.7283051462650213					
Category	Term	Count	PValue	Genes	Fold Enrichment	FDR
GOTERM_CC_DIRECT	GO:0030670~phagocytic vesicle membrane	8	0.000975832789768	P06339, Q9QUN7, P01902, P35285, P01901, P01900, P61028, P01899, Q9R233, P01896, O35639	5.00257609006774	0.007726676023901
GOTERM_CC_DIRECT	GO:0071556~integral component of lumenal side of endoplasmic reticulum membrane	4	0.013089951786648	P06339, P01902, P01901, P01900, P01899, P35564, P01896	7.8373692077728	0.06726007141437

**Annotation Cluster 67**	Enrichment Score: 0.5142599282072734					
Category	Term	Count	PValue	Genes	Fold Enrichment	FDR
KEGG_PATHWAY	mmu04662:B cell receptor signaling pathway	13	0.000332881259704	Q60631, P27870, P29351, Q60591, Q9EQ32, Q9ES52, P63001, P63085, Q8CIH5, P63328, P08101, P25799, P31938	3.41705399863295	0.005635204182133
KEGG_PATHWAY	mmu04380:Osteoclast differentiation	17	0.00121651023727	Q60631, Q8CIZ9, P26151, O70145, P08508, Q60591, Q9WTK5, Q09014, P63001, P63085, Q61093, P09581, Q8CIH5, P63328, P08101, P25799, P31938	2.48247512721197	0.01517436453858

**Annotation Cluster 68**	Enrichment Score: 0.27288716146073455					
Category	Term	Count	PValue	Genes	Fold Enrichment	FDR

UP_KEYWORDS	Transferase	68	0.007909661753991	Q9D964, P11928, P52431, B2RQC6, P19096, Q9Z277, P55264, Q8CGY8, O70172, Q9JHF7, O35130, P68181, Q61036, Q9WTP6, Q9DBP5, Q9CZN7, P63085, Q9DC50, P31938, P52480, Q9JJZ4, P10649, Q9DBC7, Q91YQ5, Q8BMF4, Q9DBG6, P46978, Q9D6K9, Q68FH4, P32020, P09581, O54734, Q8K183, O09131, Q9CZU6, Q8QZT1, P08775, P23492, P00493, P61804, Q8BYI6, O70311, Q6PB93, Q80X41, P11103, O08528, Q93092, Q9CPU4, Q99L20, Q9CWJ9, P68037, P40142, Q04899, Q80VA0, O54988, Q99KH8, Q8K297, Q8VEB4, A2RSY6, Q8BH61, Q60855, Q8CI94, Q8VDL4, Q61263, Q04447, Q9Z110, P29758, Q9WUA3	1.36519834785364	0.030148420888399
