# Supplementary material for: Repeated Exposure of Macrophages to Synthetic Amorphous Silica Induces Adaptive Proteome Changes and a Moderate Cell Activation
Source: Nanomaterials (Basel). 2022 Apr 22;12(9):1424. doi: 10.3390/nano12091424 (PMC9105884; doi:10.3390/nano12091424)
Supplement: Supplementary file 1 [file nanomaterials-12-01424-s001.zip › Table S5.pdf]

Table S5: results of the pathway analysis by the DAVID tool using proteins modulated by fumed silica
cutoff value for cluster selection: FDR≤0.1

Annotation Cluster 1 Enrichment Score: 4.729766471127018

Category	Term	Count	PValue	Genes	Fold Enrichment	FDR
GOTERM_MF_DIRECT	GO:0098641~cadherin binding involved in cell-cell adhesion	16	4.65662105374771E-06	Q8BK64, Q9JKF1, P59325, P35700, Q9DCL9, Q6A028, Q61191, P09055, P06151, P60335, Q5FWK3, O54988, P05064, P17182, P47911, P26039	4.33111452466291	0.001145528779222
GOTERM_CC_DIRECT	GO:0005913~cell-cell adherens junction	16	7.68227002605324E-06	Q8BK64, Q9JKF1, P59325, P35700, Q9DCL9, Q6A028, Q61191, P09055, P06151, P60335, Q5FWK3, O54988, P05064, P17182, P47911, P26039	4.16545733806472	0.000187118148492
GOTERM_BP_DIRECT	GO:0098609~cell-cell adhesion	11	0.000180775728776	Q8BK64, Q61191, P06151, Q5FWK3, O54988, P05064, P17182, P59325, P35700, P26039, Q9DCL9	4.47826184847461	0.052200172511695

Annotation Cluster 2 Enrichment Score: 4.34692497528786

Category	Term	Count	PValue	Genes	Fold Enrichment	FDR
KEGG_PATHWAY	mmu01130:Biosynthesis of antibiotics	22	4.63260550456338E-10	P06745, Q3TRM8, Q8BH95, Q8VDL4, O08528, Q9WUM5, Q9JLJ2, P54822, Q8VCN5, Q9DCL9, Q9WTP6, O35855, P14152, P16460, P06151, P05063, Q9Z0S1, P05064, P09411, P17182, Q9CZU6, Q9QXG4	5.3786636149787	9.07990678894422E-08
UP_KEYWORDS	Glycolysis	8	7.52692254316195E-08	P06745, Q3TRM8, P05063, Q8VDL4, P05064, O08528, P09411, P17182	21.3333333333333	2.60933314829614E-06
GOTERM_BP_DIRECT	GO:0006096~glycolytic process	8	3.36980156341036E-07	P06745, Q3TRM8, P05063, Q8VDL4, P05064, O08528, P09411, P17182	17.0988179669031	0.000446835687308
KEGG_PATHWAY	mmu00010:Glycolysis / Gluconeogenesis	11	3.79518606084664E-07	P06745, Q3TRM8, P06151, P05063, Q8VDL4, P05064, O08528, P09411, P17182, Q9JLJ2, Q9QXG4	8.71995464852608	3.7192823396297E-05
KEGG_PATHWAY	mmu01200:Carbon metabolism	13	1.76165161540288E-06	P06745, Q3TRM8, Q8BH95, Q8VDL4, O08528, Q9WUM5, P14152, P05063, P05064, P09411, P17182, Q9CZU6, Q9QXG4	5.86341778090547	0.000115094572206
GOTERM_BP_DIRECT	GO:0005975~carbohydrate metabolic process	11	0.000360575512936	O09159, P06745, Q3TRM8, P14152, P06151, Q8VDL4, O08528, P09411, Q9CPU0, Q9CZU6, Q8BHN3	4.10869655029953	0.053124792239309
KEGG_PATHWAY	mmu00051:Fructose and mannose metabolism	5	0.003712513945938	Q3TRM8, P05063, P05064, O08528, P21300	7.69407763105242	0.066150248491267

Annotation Cluster 3 Enrichment Score: 3.40900541367469

Category	Term	Count	PValue	Genes	Fold Enrichment	FDR
UP_KEYWORDS	Nucleotide-binding	44	2.32385735814229E-07	P32921, Q61699, P28740, Q61316, Q501J6, O08528, P59325, Q91YH5, Q9DCL9, Q9WTP6, Q9JM14, P63001, Q8C878, Q9QWT9, P49717, P68037, P16460, Q04899, P61028, O54988, P09411, Q8CGK3, P61205, P60710, Q9D0F6, Q62159, Q2TBE6, Q61820, Q3TRM8, P68033, Q61183, Q9WUM5, Q3THK7, P36536, Q04692, P84096, Q68FL6, O55143, P51859, P08113, Q9QXG4, P08752, P60843, P60766	2.34131508931965	6.90517614990853E-06
GOTERM_MF_DIRECT	GO:0000166~nucleotide binding	47	5.1276111626207E-05	Q9CX86, P32921, Q61699, P28740, Q61316, Q501J6, O08528, P59325, Q91YH5, Q9DCL9, Q9WTP6, Q9JM14, P63001, Q8C878, P49717, Q8VIJ6, P68037, P16460, Q04899, P61028, O54988, Q8CGK3, P61205, P60710, Q9D0F6, Q62159, Q2TBE6, Q61820, Q3TRM8, P68033, Q61183, Q9WUM5, P70333, Q3THK7, P36536, Q04692, P17225, P84096, Q68FL6, O55143, P51859, P08113, O35737, Q9QXG4, P08752, P60843, P60766	1.83348091302637	0.006306961730023
UP_KEYWORDS	ATP-binding	30	0.000313616958195	P32921, Q61699, P28740, Q61316, Q501J6, O08528, Q9DCL9, Q9WTP6, Q8C878, Q9QWT9, P49717, P68037, P16460, Q04899, O54988, P09411, Q8CGK3, P60710, Q9D0F6, Q2TBE6, Q3TRM8, P68033, Q61183, Q3THK7, Q04692, Q68FL6, O55143, P08113, Q9QXG4, P60843	2.0542920029347	0.004077020456537

Annotation Cluster 4 Enrichment Score: 2.7113522154185365

Category	Term	Count	PValue	Genes	Fold Enrichment	FDR
GOTERM_CC_DIRECT	GO:0005623~cell	12	0.000123828148036	P27773, O55143, Q3TRM8, O08807, P47791, P09103, O08528, P29391, P30681, Q8BZW8, Q8K2V6, P35700	4.27365103515731	0.002483846969423
UP_KEYWORDS	Redox-active center	6	0.000141239183041	P27773, O08807, P47791, P09103, Q9ESY9, P35700	11.9148936170213	0.00225982692866

Annotation Cluster 5 Enrichment Score: 2.5374635408989508

Category	Term	Count	PValue	Genes	Fold Enrichment	FDR
UP_KEYWORDS	mRNA splicing	12	5.75080583517719E-05	Q9CSN1, P17225, Q9JKP5, Q9DAR7, Q99NB9, Q8VIJ6, Q8BTI8, Q99KP6, P83870, P62317, O35691, O35737	4.66666666666667	0.001087425103379
UP_KEYWORDS	mRNA processing	13	0.000122590951395	Q9CSN1, Q99NB9, Q8BTI8, Q61183, O35691, P17225, Q9JKP5, Q9DAR7, Q8VIJ6, Q99KP6, P83870, P62317, O35737	3.95222584147666	0.002124909824183
GOTERM_CC_DIRECT	GO:0071013~catalytic step 2 spliceosome	8	0.000166506607858	Q9CSN1, Q99NB9, Q8BTI8, Q99KP6, P83870, P62317, O35691, O35737	6.85564853556485	0.003154375182189
UP_KEYWORDS	Spliceosome	8	0.000264570776243	Q9CSN1, Q99NB9, Q8BTI8, Q99KP6, P83870, P62317, O35691, O35737	6.38176638176638	0.003668714763897
GOTERM_BP_DIRECT	GO:0006397~mRNA processing	14	0.000304163224326	Q9CSN1, Q99NB9, Q8BTI8, Q61183, O35691, P17225, Q9JKP5, Q9DAR7, Q8VIJ6, Q99KP6, P83870, P60335, P62317, O35737	3.34542090656799	0.052200172511695
GOTERM_BP_DIRECT	GO:0008380~RNA splicing	12	0.000314933167491	Q9CSN1, P17225, Q9JKP5, Q9DAR7, Q99NB9, Q8VIJ6, Q8BTI8, Q99KP6, P83870, P62317, O35691, O35737	3.83127041582061	0.052200172511695
GOTERM_CC_DIRECT	GO:0005681~spliceosomal complex	8	0.001356831203338	Q9CSN1, Q99NB9, Q8BTI8, Q99KP6, P83870, P62317, O35691, O35737	4.83928131922225	0.019278310014091

Annotation Cluster 6 Enrichment Score: 2.5373357739990734

Category	Term	Count	PValue	Genes	Fold Enrichment	FDR
UP_KEYWORDS	Ribonucleoprotein	16	1.23943400251192E-06	Q9CX86, Q9D0G0, P19253, P70333, P62270, Q8VDM6, Q61990, P63323, P62245, Q6DFW4, P60335, P62317, P60867, O35737, P47911, P62830	4.84848484848485	2.86446969469422E-05
GOTERM_BP_DIRECT	GO:0006412~translation	16	0.000241783752088	P32921, P05555, Q9D0G0, P19253, Q9CR62, P59325, P62270, Q68FL6, P63323, P62245, Q61749, Q8QZY1, P60867, P47911, P62830, P60843	3.07011195415716	0.052200172511695
GOTERM_CC_DIRECT	GO:0005840~ribosome	9	0.002115115799313	P05555, P62245, Q9D0G0, P19253, P35564, P62270, P60867, P47911, P62830	3.93835128638832	0.028850179502623
UP_KEYWORDS	Ribosomal protein	8	0.006255720337146	P63323, P62245, Q9D0G0, P19253, P62270, P60867, P47911, P62830	3.67816091954023	0.048192215930604

Annotation Cluster 7 Enrichment Score: 2.400163403951474

Category	Term	Count	PValue	Genes	Fold Enrichment	FDR

GOTERM_BP_DIRECT	GO:0006457~protein folding	9	0.000263164484311	P27773, Q8BK64, Q9WU28, P24369, P09103, P08113, P35564, P04441, P17742	5.41017287234043	0.052200172511695
GOTERM_CC_DIRECT	GO:0005790~smooth endoplasmic reticulum	5	0.000391902521897	P27773, O08807, P24369, P08113, P35564	14.18410041841	0.006074489089406
GOTERM_CC_DIRECT	GO:0034663~endoplasmic reticulum chaperone complex	3	0.008889852004786	P24369, P09103, P08113	20.5669456066946	0.091861804049457

Annotation Cluster 8 Enrichment Score: 2.294322822731369

Category	Term	Count	PValue	Genes	Fold Enrichment	FDR
UP_KEYWORDS	Lyase	9	0.000178055014714	Q8BH95, P05063, P05064, P17182, Q9CPU0, P54822, Q8VCN5, Q9WVQ5, Q9DCL9	5.75342465753425	0.002645388790031

Annotation Cluster 9 Enrichment Score: 1.9848897071203018

Category	Term	Count	PValue	Genes	Fold Enrichment	FDR
UP_KEYWORDS	Isomerase	7	0.002002568759485	P27773, P06745, Q04750, P24369, P09103, P34884, P17742	5.3551912568306	0.019834966760612

Annotation Cluster 10 Enrichment Score: 1.9641236439846836

Category	Term	Count	PValue	Genes	Fold Enrichment	FDR
UP_KEYWORDS	GTP-binding	12	0.000932756438286	P84096, P63001, Q62159, Q61820, P61028, Q9WUM5, P59325, P36536, Q91YH5, P08752, P60766, P61205	3.37349397590361	0.009700666958174
UP_KEYWORDS	Prenylation	7	0.006852619317892	P84096, P63001, Q62159, P63213, P61028, P14733, P60766	4.16135881104034	0.050905172075767

Annotation Cluster 11 Enrichment Score: 1.903782710156112

Category	Term	Count	PValue	Genes	Fold Enrichment	FDR
UP_KEYWORDS	Oxidoreductase	15	0.009007246765578	P09528, P08228, Q8BSY0, Q91YR9, O08807, P47791, Q9ESY9, Q8BVI4, Q99KP3, Q9JLJ2, P35700, P14152, P06151, Q9JI75, P21300	2.19092331768388	0.058547103976259

Annotation Cluster 12 Enrichment Score: 1.6829498804963494

Category	Term	Count	PValue	Genes	Fold Enrichment	FDR
KEGG_PATHWAY	mmu05100:Bacterial invasion of epithelial cells	8	0.00065931075651	Q8BUR4, P84096, P63001, P09055, Q68FD5, Q8BPU7, P60766, P60710	5.36612593755451	0.016153113534495

Annotation Cluster 13 Enrichment Score: 1.6151610292545608

Category	Term	Count	PValue	Genes	Fold Enrichment	FDR

UP_KEYWORDS	RNA-binding	16	0.002098302718874	Q80SW1, Q9CX86, Q501J6, Q61183, P70333, P62270, Q8VDM6, Q61990, P17225, Q9JKP5, Q68FL6, Q8VIJ6, P60335, O35737, A2RSY6, P60843	2.48474764281753	0.019838498432995

**Annotation Cluster 14**	Enrichment Score: 1.5187892501433913					
Category	Term	Count	PValue	Genes	Fold Enrichment	FDR
UP_KEYWORDS	Purine biosynthesis	3	0.008184301375907	Q3THK7, P54822, Q9DCL9	21.5384615384615	0.056744489539622

**Annotation Cluster 15**	Enrichment Score: 1.4432081195169078					
Category	Term	Count	PValue	Genes	Fold Enrichment	FDR
UP_KEYWORDS	Protein biosynthesis	7	0.005029622450828	P32921, Q68FL6, P10711, Q61749, Q8QZY1, P59325, P60843	4.44444444444444	0.040236979606625

**Annotation Cluster 16**	Enrichment Score: 1.4185799608286274					
Category	Term	Count	PValue	Genes	Fold Enrichment	FDR
GOTERM_CC_DIRECT	GO:0036464~cytoplasmic ribonucleoprotein granule	5	0.000341066169004	P63001, Q9JKF1, P47911, P60766, P60710	14.6906754333533	0.005538264934785
KEGG_PATHWAY	mmu04670:Leukocyte transendothelial migration	8	0.007024367631151	P05555, P63001, P09055, O70145, P11835, P08752, P60766, P60710	3.54710019601061	0.093891157632156
KEGG_PATHWAY	mmu04810:Regulation of actin cytoskeleton	11	0.007185547777971	Q8BUR4, P05555, P63001, Q3UQ44, P13020, P09055, P11835, Q9JKF1, Q61210, P60766, P60710	2.70195777841653	0.093891157632156

**Annotation Cluster 17**	Enrichment Score: 1.4097499508569125					
Category	Term	Count	PValue	Genes	Fold Enrichment	FDR
UP_KEYWORDS	DNA replication	5	0.018912063937907	Q60973, P49717, Q62193, Q99MD9, Q9D0F6	4.91228070175439	0.103909960698105

**Annotation Cluster 18**	Enrichment Score: 1.292265533056753					
Category	Term	Count	PValue	Genes	Fold Enrichment	FDR
KEGG_PATHWAY	mmu04612:Antigen processing and presentation	9	0.000151506589805	P27773, P06339, Q61316, P01902, P01901, P01900, P01899, Q9R233, Q9ESY9, P35564, P04441	5.74240915878547	0.004949215266978
GOTERM_CC_DIRECT	GO:0030670~phagocytic vesicle membrane	6	0.000252507931086	P06339, P17047, P01902, P01901, P01900, P61028, P01899, Q9R233	10.5022700970355	0.00430526022501
GOTERM_CC_DIRECT	GO:0071556~integral component of lumenal side of endoplasmic reticulum membrane	4	0.00071555813496	P06339, P01902, P01901, P01900, P01899, P35564	21.9380753138075	0.010608927131367
UP_KEYWORDS	MHC I	3	0.008184301375907	P06339, P01902, P01901, P01900, P01899	21.5384615384615	0.056744489539622

Annotation Cluster 19 Enrichment Score: 1.270307085208816

Category	Term	Count	PValue	Genes	Fold Enrichment	FDR
UP_KEYWORDS	Mitochondrion	20	0.018964113955634	Q8K411, Q2TBE6, Q9R0X4, P56135, Q8BH95, Q9D0G0, P47791, Q8BH86, Q9CR62, Q8WTY4, Q9WUM5, P19783, Q9WTP6, O35855, P32020, Q9EPB4, Q9DCX2, Q9CZU6, Q9JK81, Q8CGK3	1.7643352236925	0.103909960698105

Annotation Cluster 20 Enrichment Score: 1.2182827387595705

Category	Term	Count	PValue	Genes	Fold Enrichment	FDR
KEGG_PATHWAY	mmu04145:Phagosome	13	9.21276543953499E-05	P05555, P06339, O70145, P08508, P11835, P35564, P63001, P17047, P09055, P01902, P01901, P01900, P01899, O70439, P60710	3.97752317301189	0.004514255065372

Annotation Cluster 21 Enrichment Score: 1.1657249375558902

Category	Term	Count	PValue	Genes	Fold Enrichment	FDR
KEGG_PATHWAY	mmu00620:Pyruvate metabolism	5	0.00612016870271	P14152, P06151, Q9CPU0, Q9JLJ2, Q9QXG4	6.70765742194314	0.093891157632156

Annotation Cluster 32 Enrichment Score: 0.8933160367706657

Category	Term	Count	PValue	Genes	Fold Enrichment	FDR
UP_KEYWORDS	Immunity	12	0.004031995046468	P06339, Q9CQW9, P34884, Q9ESY9, P30681, Q61990, P63158, P01902, P01901, P01900, P01899, Q9EPB4, Q6P549, P04441	2.7930174563591	0.034943957069393
