# Supplementary material for: Repeated Exposure of Macrophages to Synthetic Amorphous Silica Induces Adaptive Proteome Changes and a Moderate Cell Activation
Source: Nanomaterials (Basel). 2022 Apr 22;12(9):1424. doi: 10.3390/nano12091424 (PMC9105884; doi:10.3390/nano12091424)
Supplement: Supplementary file 1 [file nanomaterials-12-01424-s001.zip › Table S6.pdf]

Table S6: proteins linked to mitochondria

accession number	gene_name	description	ratio Pr/ctrl	ratio Fu/ctrl	T test Pr vs ctrl	T test Py vs ctrl
O35129	Phb2	Prohibitin-2	1.129835927	1.034913261	0.002533893	0.193535478
O35658	C1qbp	Complement component 1 Q subcomponent-binding protein, mitochondrial	1.246582495	1.009048216	0.005126001	0.886624753
P00405	Mtco2	Cytochrome c oxidase subunit 2	0.735392594	0.998870793	0.015434415	0.986849666
P09671	Sod2	Superoxide dismutase [Mn], mitochondrial	1.332345703	0.993033623	0.00222107	0.87326313
P11928	Oas1a	2'-5'-oligoadenylate synthase 1A	1.285445563	1.096041935	0.027726763	0.193590535
P16332	Mut	Methylmalonyl-CoA mutase, mitochondrial	1.17406184	1.017172881	0.005280587	0.665421768
P19783	Cox4i1	Cytochrome c oxidase subunit 4 isoform 1, mitochondrial	1.107095629	1.065006288	0.002540912	0.015186435
P20108	Prdx3	Thioredoxin-dependent peroxide reductase, mitochondrial Prx3	1.157475522	1.069655803	0.002485883	0.069271673
P28352	Apex1	DNA-(apurinic or apyrimidinic site) lyase	0.819428042	0.879554978	0.030341022	0.054971364
P29758	Oat	Ornithine aminotransferase, mitochondrial	1.267633192	1.042378612	3.99937E-05	0.042302865
P32020	Scp2	Non-specific lipid-transfer protein	0.719113476	0.788356734	0.007574101	0.004745788
P36552	Cpox	Oxygen-dependent coproporphyrinogen-III oxidase, mitochondrial	0.676224731	0.968451308	0.006153699	0.742848346
P38060	Hmgcl	Hydroxymethylglutaryl-CoA lyase, mitochondrial	0.819813757	0.959369323	0.004561241	0.410133949
P38647	Hspa9	Stress-70 protein, mitochondrial	1.263736865	1.073657338	0.004889481	0.302685727
P41216	Acsl1	Long-chain-fatty-acid--CoA ligase 1	1.197712734	1.013246654	0.006746039	0.725914863
P48962	Slc25a4	ADP/ATP translocase 1	1.176749339	1.034227196	0.000999067	0.317209232
P51174	Acadl	Long-chain specific acyl-CoA dehydrogenase, mitochondrial	1.116231094	1.050999123	0.004417197	0.175955813
P51881	Slc25a5	ADP/ATP translocase 2	1.218654174	1.026985015	0.000157509	0.425442585
P56135	Atp5mf	ATP synthase subunit f, mitochondrial	1.221393033	1.109296296	0.000560756	0.01847533
P56480	Atp5f1b	ATP synthase subunit beta, mitochondrial	1.104016508	1.05561257	0.02764585	0.076372893
P59017	Bcl2l13	Bcl-2-like protein 13	0.339690983	0.884244902	0.019963606	0.616853809
P62908	Rps3	40S ribosomal protein S3	1.123328077	1.008923814	0.00255899	0.747024505
P63038	Hspd1	60 kDa heat shock protein, mitochondrial	1.125244274	1.038345336	0.000320689	0.044564605
P63087	Ppp1cc	Serine/threonine-protein phosphatase PP1-gamma catalytic subunit	0.840743359	0.914840159	0.005791729	0.057155038
P67778	Phb	Prohibitin	1.123958779	1.002925161	0.010113763	0.926671094

P99029	Prdx5	Peroxiredoxin-5, mitochondrial	0.758889457	0.857463329	0.003933749	0.060601088
Q03265	Atp5f1a	ATP synthase subunit alpha, mitochondrial	1.101111398	1.032742313	0.003000464	0.060770771
Q05921	Rnasel	2-5A-dependent ribonuclease	0.683721407	0.750508144	0.011798925	0.066830648
Q14C51	Ptcd3	Pentatricopeptide repeat domain-containing protein 3, mitochondrial	1.354000989	1.095789987	0.034473285	0.127217175
Q3TL44	Nlrx1	NLR family member X1	0.841418495	0.953612312	0.085814762	0.289174857
Q60597	Ogdh	2-oxoglutarate dehydrogenase, mitochondrial	1.140917251	1.060496406	0.000120067	0.038505047
Q60931	Vdac3	Voltage-dependent anion-selective channel protein 3	1.152675268	1.018637496	0.001376895	0.563536334
Q62465	Vat1	Synaptic vesicle membrane protein VAT-1 homolog	0.732685002	0.989629175	0.001912047	0.842823689
Q6P3A8	Bckdhb	2-oxoisovalerate dehydrogenase subunit beta, mitochondrial	0.563177632	0.815164552	0.006279063	0.148122198
Q6PB66	Lrpprc	Leucine-rich PPR motif-containing protein, mitochondrial	1.184423355	1.076639133	0.00353685	0.118913828
Q78IK2	Atp5md	Up-regulated during skeletal muscle growth protein 5	1.144676857	0.94535141	0.003949396	0.226226199
Q78IK4	Apool	MICOS complex subunit Mic27	0.918855935	0.938495996	0.021896988	0.063203722
Q811U4	Mfn1	Mitofusin-1	1.758044799	1.095676459	0.004433715	0.692082075
Q8BGH2	Samm50	Sorting and assembly machinery component 50 homolog	1.172946106	1.029190215	0.003570799	0.663343489
Q8BH86	Dglucy	D-glutamate cyclase, mitochondrial	0.635894178	0.818974968	0.001061496	0.02270373
Q8BH95	Echs1	Enoyl-CoA hydratase, mitochondrial	0.81513449	0.87439013	0.004987222	0.023936642
Q8BMF4	Dlat	Dihydrolipoyllysine-residue acetyltransferase component of pyruvate dehydrogenase complex, mitochondrial	1.119977864	1.060972461	0.01512153	0.054667904
Q8BMS1	Hadha	Trifunctional enzyme subunit alpha, mitochondrial	0.884115547	0.956841894	0.002220491	0.1297981
Q8BP40	Acp6	Lysophosphatidic acid phosphatase type 6	0.744733387	0.88203816	0.007667602	0.326682704
Q8CGK3	Lonp1	Lon protease homolog, mitochondrial	1.153231196	1.102458889	0.007621942	0.028061524
Q8JZR0	Acsl5	Long-chain-fatty-acid--CoA ligase 5	1.110855155	1.00971884	0.009702883	0.773577546
Q8QZT1	Acat1	Acetyl-CoA acetyltransferase, mitochondrial	0.85669917	0.909236673	0.002081238	0.020956994
Q8R1I1	Uqcr10	Cytochrome b-c1 complex subunit 9	1.222113776	1.048813555	0.004470015	0.358517296

Q8VDK1	Nit1	Deaminated glutathione amidase	0.600417003	0.814885405	0.005428763	0.119433048
Q8WTY4	Ciapin1	Anamorsin	0.419992511	0.701233726	1.28693E-05	0.04810254
Q99LX0	Park7	Protein/nucleic acid deglycase DJ-1	0.683484548	0.897481046	0.00475433	0.160566324
Q9CPQ1	Cox6c	Cytochrome c oxidase subunit 6C	1.213058624	1.049462551	0.000347832	0.153293215
Q9CPQ3	Tomm22	Mitochondrial import receptor subunit TOM22 homolog	0.849591285	1.072415204	0.036739609	0.261491408
Q9CPV4	Glod4	Glyoxalase domain-containing protein 4	0.810717354	0.972547966	0.000578651	0.30961383
Q9CQN1	Trap1	Heat shock protein 75 kDa, mitochondrial	1.440789908	1.278062455	0.000830321	0.066341924
Q9CQN6	Tmem14c	Transmembrane protein 14C	1.270038298	1.036161387	0.008086005	0.649579907
Q9CR62	Slc25a11	Mitochondrial 2-oxoglutarate/malate carrier protein	1.135506908	1.120601088	0.012552281	0.023143678
Q9CXT8	Pmpcb	Mitochondrial-processing peptidase subunit beta	1.206171867	0.864809902	0.001528758	0.557885593
Q9CYG7	Tomm34	Mitochondrial import receptor subunit TOM34	0.830737984	0.889982954	0.021865182	0.136182571
Q9CZD3	Gars	Glycine--tRNA ligase	1.161207289	1.060155091	0.017496975	0.083260838
Q9CZU6	Cs	Citrate synthase, mitochondrial	1.165369721	1.057230626	0.000758099	0.005533219
Q9D172	Gatd3a	Glutamine amidotransferase-like class 1 domain-containing protein 3A, mitochondrial	0.813822625	0.865534683	0.004131675	0.063834172
Q9D6K5	Synj2bp	Synaptojanin-2-binding protein	1.270926418	1.07243633	0.012714228	0.247341673
Q9D6U8	Fam162a	Protein FAM162A	1.347411615	1.135720045	0.002408274	0.072679044
Q9D880	Timm50	Mitochondrial import inner membrane translocase subunit TIM50	0.938113622	0.970462852	0.028275219	0.174855024
Q9D964	Gatm	Glycine amidinotransferase, mitochondrial	0.813274821	0.869573	0.000980394	0.03719697
Q9DCN2	Cyb5r3	NADH-cytochrome b5 reductase 3	1.21038842	1.077371373	0.003638862	0.038532839
Q9EPB4	Pycard	Apoptosis-associated speck-like protein containing a CARD	0.883879676	0.841254737	0.001444539	0.021412344
Q9JIK9	Mrps34	28S ribosomal protein S34, mitochondrial	1.137472101	0.988529205	0.015233536	0.787494993
Q9JK81	Myg1	UPF0160 protein MYG1, mitochondrial	0.80861539	0.946486505	0.000172796	0.072743279
Q9JM90	Stap1	Signal-transducing adaptor protein 1	1.375519337	1.069388073	0.012401315	0.340023646
Q9QYR9	Acot2	Acyl-coenzyme A thioesterase 2, mitochondrial	0.829231863	0.913943398	0.024604635	0.169339435

Q9R0X4	Acot9	Acyl-coenzyme A thioesterase 9, mitochondrial	1.236014962	1.255790773	0.012366826	0.009974659
Q9WTP6	Ak2	Adenylate kinase 2, mitochondrial	0.877418771	0.904475564	0.022543927	0.05362444
Q9WUM5	Suclg1	Succinate--CoA ligase [ADP/GDP-forming] subunit alpha, mitochondrial	0.888785741	0.861061759	0.001077851	0.002579821
Q9Z110	Aldh18a1	Delta-1-pyrroline-5-carboxylate synthase	1.265385246	1.048428033	0.000150112	0.340822589
