# Supplementary material for: Repeated Exposure of Macrophages to Synthetic Amorphous Silica Induces Adaptive Proteome Changes and a Moderate Cell Activation
Source: Nanomaterials (Basel). 2022 Apr 22;12(9):1424. doi: 10.3390/nano12091424 (PMC9105884; doi:10.3390/nano12091424)
Supplement: Supplementary file 1 [file nanomaterials-12-01424-s001.zip › Table S7.pdf]

Table S7: proteins linked to cell redox homeostasis

accession number	gene_name	description	ratio rPr/ctrl	ratio Fu/ctrl	T test Pr vs. ctrl	T test Fu vs ctrl
P28352	Apex1	DNA-(apurinic or apyrimidinic site) lyase	0.819428	0.879555	0.030341	0.0549714
Q8R180	Ero1a	ERO1-like protein alpha	1.4267517	1.0507847	0.0005103	0.4003907
Q8BZW8	Nhlrc2	NHL repeat-containing protein 2	1.5002926	1.4896671	0.0317271	0.0348889
P09103	P4hb	Protein disulfide-isomerase	1.3321121	1.0523063	0.0002386	0.0305743
P27773	Pdia3	Protein disulfide-isomerase A3	1.0986432	1.0465273	0.0002576	0.0101618
P08003	Pdia4	Protein disulfide-isomerase A4	1.1827833	1.0572835	0.000747	0.1560396
Q922R8	Pdia6	Protein disulfide-isomerase A6	1.1337347	0.9820241	0.0008646	0.3990531
P35700	Prdx1	Peroxiredoxin-1	1.1591502	1.1129194	1.406E-05	0.0036429
P20108	Prdx3	Thioredoxin-dependent peroxide reductase, mitochondrial Prx3	1.1574755	1.0696558	0.0024859	0.0692717
O08807	Prdx4	Peroxiredoxin-4	1.2954477	1.1735089	0.0002375	0.0046681
P99029	Prdx5	Peroxiredoxin-5, mitochondrial	0.7588895	0.8574633	0.0039337	0.0606011
O08709	Prdx6	Peroxiredoxin-6	0.8286838	0.9322557	0.0016014	0.0868954
Q91VW3	Sh3bgrl3	SH3 domain-binding glutamic acid-rich-like protein 3	0.7380794	1.4321918	0.0332647	0.4175075
Q8VBT0	Tmx1	Thioredoxin-related transmembrane protein 1	1.1900357	0.9962529	0.0004372	0.9396225
Q9JMH6	Txnrd1	Thioredoxin reductase 1, cytoplasmic	1.3005555	1.0432999	0.0074358	0.5895408
P46413	Gss	Glutathione synthetase	0.7014626	0.8743287	0.0240252	0.1522542
P47791	Gsr	Glutathione reductase, mitochondrial	0.9701236	0.9343079	0.2482618	0.0039174
