# Supplementary material for: Repeated Exposure of Macrophages to Synthetic Amorphous Silica Induces Adaptive Proteome Changes and a Moderate Cell Activation
Source: Nanomaterials (Basel). 2022 Apr 22;12(9):1424. doi: 10.3390/nano12091424 (PMC9105884; doi:10.3390/nano12091424)
Supplement: Supplementary file 1 [file nanomaterials-12-01424-s001.zip › Table S8.pdf]

Table S8: proteins linked to lysosomes

accession number	gene_name	description	ratio Pr/ctrl	ratio Fu/ctrl	T test Pr vs ctrl	T test Py vs ctrl
O70439	Stx7	Syntaxin-7	0.8152205	0.927133	0.0003277	0.0285204
O08709	Prdx6	Peroxiredoxin-6	0.8286838	0.9322557	0.0016014	0.0868954
O09159	Man2b1	Lysosomal alpha-mannosidase	0.5432037	0.8744404	4.3E-06	0.0060299
O70370	Ctss	Cathepsin S	0.7378722	0.9307412	1.821E-05	0.1781141
O70404	Vamp8	Vesicle-associated membrane protein 8	0.7382257	1.0473583	0.0842288	0.3784841
O88384	Vti1b	Vesicle transport through interaction with t-SNAREs homolog 1B	0.6138922	0.980002	0.0031462	0.7655956
O88531	Ppt1	Palmitoyl-protein thioesterase 1	0.7369213	0.9803523	0.024622	0.8372305
O89017	Lgmn	Legumain	0.5272381	0.81788	0.0013673	0.0728018
O89023	Tpp1	Tripeptidyl-peptidase 1	0.6368712	0.9176056	0.0004264	0.1401913
P10605	Ctsb	Cathepsin B	0.7351592	0.9233382	0.0021039	0.1549488
P12265	Gusb	Beta-glucuronidase	0.69189	0.9424831	0.0018083	0.3413921
P16675	Ctsa	Lysosomal protective protein	0.7933639	1.0047681	0.0014561	0.9139159
P17047	Lamp2	Lysosome-associated membrane glycoprotein 2	1.0085785	1.0949972	0.8304094	0.0326377
P17439	Gba	Glucosylceramidase	0.7359921	0.9187196	0.0307469	0.1293887
P18242	Ctsd	Cathepsin D	0.7153965	1.0005894	0.0067871	0.9927962
P20060	Hexb	Beta-hexosaminidase subunit beta	0.7627635	0.9610381	0.0067747	0.4284235
P23780	Glb1	Beta-galactosidase	0.8165547	0.955713	0.0135993	0.5097627
P24668	M6pr	Cation-dependent mannose-6-phosphate receptor	1.0702246	0.9801959	0.0141635	0.5424002
P29416	Hexa	Beta-hexosaminidase subunit alpha	0.8214424	0.9676633	0.0043188	0.430075
P48441	Idua	Alpha-L-iduronidase	0.7567304	0.9821068	0.0040303	0.8492658
P50428	Arsa	Arylsulfatase A	0.478642	0.8143551	0.018173	0.1476579
P50429	Arsb	Arylsulfatase B	0.5314164	0.8448751	4.783E-05	0.0225149
P51569	Gla	Alpha-galactosidase A	0.6835181	0.9681942	0.000531	0.5062944
P52875	Tmem165	Transmembrane protein 165	1.3079191	0.9691447	0.005236	0.6632996
P70665	Siae	Sialate O-acetylesterase	0.6749521	0.9238752	2.13E-05	0.0342826
P97821	Ctsc	Dipeptidyl peptidase 1	0.6424995	0.7980016	4.772E-05	0.0022992
Q3TCN2	Plbd2	Putative phospholipase B-like 2	0.5727974	0.788187	0.0030461	0.0518813
Q571E4	Galns	N-acetylgalactosamine-6-sulfatase	0.5648582	0.8264042	0.0266381	0.2571584
Q61207	Psap	Prosaposin	0.4839972	0.7871221	0.0009209	0.0425497
Q7TMR0	Prcp	Lysosomal Pro-X carboxypeptidase	0.7396043	0.9148804	0.0087178	0.1867965
Q8BFR4	Gns	N-acetylglucosamine-6-sulfatase	0.6888079	1.0206849	0.0244023	0.8663465

Q8K2I4	Manba	Beta-mannosidase	0.5641421	0.8795761	0.0088641	0.3300241
Q8VE99	Ccdc115	Coiled-coil domain-containing protein 115	0.7700426	0.7913837	0.0101929	0.0131147
Q8VEB4	Pla2g15	Group XV phospholipase A2	0.7071917	0.9517657	0.0042617	0.4659972
Q8VEH3	Arl8a	ADP-ribosylation factor-like protein 8A	0.7723031	1.0092352	0.0039101	0.8541104
Q9CQ22	Lamtor1	Ragulator complex protein LAMTOR1	0.7949129	0.9596358	0.0122936	0.5195798
Q9CQW9	Ifitm3	Interferon-induced transmembrane protein 3	1.4607914	1.2602396	0.0031971	0.0088772
Q9ESY9	Ifi30	Gamma-interferon-inducible lysosomal thiol reductase	0.4049389	0.677976	3.43E-07	4.007E-05
Q9JHJ3	Glmp	Glycosylated lysosomal membrane protein	0.708834	0.8130006	0.005138	0.0035245
Q9JHS3	Lamtor2	Ragulator complex protein LAMTOR2	0.6197474	0.577106	0.0022696	0.0009776
Q9QWR8	Naga	Alpha-N-acetylgalactosaminidase	0.6342371	0.8896636	0.0054548	0.1938527
Q9WUU7	Ctsz	Cathepsin Z	0.7994102	0.9767766	0.0004218	0.4512307
Q9WV54	Asah1	Acid ceramidase	0.7288531	0.9099175	0.0053045	0.2137605
Q9Z0J0	Npc2	NPC intracellular cholesterol transporter 2	0.676353	0.7833006	0.0046602	0.0247731
Q9Z0M5	Lipa	Lysosomal acid lipase/cholesteryl ester hydrolase	1.6849821	1.2364377	0.0054501	0.0551958
P28798	Grn	Granulins	0.684622	0.6193002	0.0201477	0.003634
Q09014	Ncf1	Neutrophil cytosol factor 1	0.9276363	0.973097	0.0060703	0.2079595
O70145	Ncf2	Neutrophil cytosol factor 2	0.8562981	0.9196041	0.0189294	0.0218527
P97369	Ncf4	Neutrophil cytosol factor 4	1.0789373	1.0575496	0.0873325	0.2493613
Q8CIZ9	Nox1	NADPH oxidase 1	1.4172108	1.0061708	0.0032124	0.9565631
P50408	Atp6v1f	V-type proton ATPase sub. F	0.6764728	0.8520792	0.0558461	0.4287312
P50518	Atp6v1e1	V-type proton ATPase sub. E 1	0.6214155	0.7445003	0.0079272	0.0175893
P51863	Atp6v0d1	V-type proton ATPase sub. d 1	1.0575101	1.0365863	0.0298925	0.2672908
P63081	Atp6v0c	V-type proton ATPase 16 kDa proteolipid subunit	0.3221168	0.8576433	0.0069397	0.5426731
Q9CR51	Atp6v1g1	V-type proton ATPase sub. G 1	0.6764339	0.7534751	0.0165677	0.040652
