# Supplementary material for: Repeated Exposure of Macrophages to Synthetic Amorphous Silica Induces Adaptive Proteome Changes and a Moderate Cell Activation
Source: Nanomaterials (Basel). 2022 Apr 22;12(9):1424. doi: 10.3390/nano12091424 (PMC9105884; doi:10.3390/nano12091424)
Supplement: Supplementary file 1 [file nanomaterials-12-01424-s001.zip › Table S9.pdf]

Table S9: proteins linked to immunity

accession number	gene_name	description	ratio Pr/ctrl	ratio Fu/ctrl	T test Pr/ vs ctrl	T test Fu vs ctrl
O35658	C1qbp	Complement component 1 Q subcomponent-binding protein, mitochondrial	1.2465825	1.0090482	0.005126	0.8866248
P01896		H-2 class I histocompatibility antigen, alpha chain	1.2104044	1.0837322	0.0098115	0.1721726
P01899	H2-D1	H-2 class I histocompatibility antigen, D-B alpha chain	1.309791	1.1268523	0.0008594	0.0214163
P01900	H2-D1	H-2 class I histocompatibility antigen, D-D alpha chain	1.2731407	1.1031101	0.0009463	0.0437297
P01901	H2-K1	H-2 class I histocompatibility antigen, K-B alpha chain	1.3182079	1.117418	0.0009352	0.0516113
P01902	H2-K1	H-2 class I histocompatibility antigen, K-D alpha chain	1.4290736	1.1647948	2.138E-05	0.0083836
P04441	Cd74	H-2 class II histocompatibility antigen gamma chain	1.0932528	1.0629249	0.0120611	0.0254623
P06339	H2-T23	H-2 class I histocompatibility antigen, D-37 alpha chain	1.4726637	1.1823455	5.269E-05	0.0252435
P09581	Csf1r	Macrophage colony-stimulating factor 1 receptor	0.7084985	0.9251445	0.0002168	0.0970605
P10810	Cd14	Monocyte differentiation antigen CD14	1.1153535	1.0394854	0.0165151	0.449006
P11928	Oas1a	2'-5'-oligoadenylate synthase 1A	1.2854456	1.0960419	0.0277268	0.1935905
P16110	Lgals3	Galectin-3	0.6757279	0.9169138	0.0018308	0.2550878
P26151	Fcgr1	High affinity immunoglobulin gamma Fc receptor I	0.8073186	0.9297252	0.0039684	0.2308725
P30681	Hmgb2	High mobility group protein B2	0.7236594	0.8526239	2.813E-07	0.0015849
P34884	Mif	Macrophage migration inhibitory factor	0.7153846	0.7216359	0.0011537	0.0043229
P42082	Cd86	T-lymphocyte activation antigen CD86	1.2275484	1.0905935	0.0012159	0.2079109
P58242	Smpdl3b	Acid sphingomyelinase-like phosphodiesterase 3b	0.7807617	0.8441364	0.0578773	0.1415048
P63158	Hmgb1	High mobility group protein B1	0.7370306	0.8578287	0.0003459	0.0323128
Q3TL44	Nlrx1	NLR family member X1	0.8414185	0.9536123	0.0858148	0.2891749
Q60710	Samhd1	Deoxynucleoside triphosphate triphosphohydrolase SAMHD1	0.6900705	0.8403139	0.002299	0.0575615
Q61990	Pcbp2	Poly(rC)-binding protein 2	0.8990317	0.897888	0.0042718	0.00904
Q62418	Dbnl	Drebrin-like protein	0.8199604	1.0097015	0.0357454	0.8921289
Q6Q899	Ddx58	Probable ATP-dependent RNA helicase DDX58	1.5029793	0.8023908	0.015233	0.3183146
Q9CQW9	Ifitm3	Interferon-induced transmembrane protein 3	1.4607914	1.2602396	0.0031971	0.0088772

Q9EPB4	Pycard	Apoptosis-associated speck-like protein containing a CARD	0.8838797	0.8412547	0.0014445	0.0214123
Q9ES52	Inpp5d	Phosphatidylinositol 3,4,5-trisphosphate 5-phosphatase 1	0.9392637	1.0315179	0.0132339	0.2094514
Q9ESY9	Ifi30	Gamma-interferon-inducible lysosomal thiol reductase	0.4049389	0.677976	3.43E-07	4.007E-05
Q9QUN7	Tlr2	Toll-like receptor 2	1.2979424	1.0041935	0.0066166	0.9564243
Q9R0Q8	Clec4e	C-type lectin domain family 4 member E	5.5255633	1.6245915	0.001789	0.1522657
Q9R1P4	Psma1	Proteasome subunit alpha type-1	0.8812492	0.9986071	0.0019385	0.9594586
P05555	Itgam	Integrin alpha-M CD11b	0.888415	0.9378634	0.0008045	0.0221523
P11835	Itgb2	Integrin beta-2	0.8640331	0.8712786	0.0006385	0.0041396
P30204	Msr1	Macrophage scavenger receptor types I and II CD204	1.3888459	1.1784492	0.0028806	0.0426059
P42082	Cd86	T-lymphocyte activation antigen CD86	1.2275484	1.0905935	0.0012159	0.2079109
P34960	Mmp12	Macrophage metalloelastase	1.7433544	1.5392242	0.0006312	0.0026677
P26151	Fcgr1	High affinity immunoglobulin gamma Fc receptor I	0.8073186	0.9297252	0.0039684	0.2308725
P08101	Fcgr2	Low affinity immunoglobulin gamma Fc region receptor II	0.7733526	0.8721087	0.0017031	0.0425047
P08508	Fcgr3	Low affinity immunoglobulin gamma Fc region receptor III	0.721257	0.8809794	0.0005607	0.0544209
A0A0B4J1G0	Fcgr4	Low affinity immunoglobulin gamma Fc region receptor IV	0.7064723	0.9184123	2.968E-05	0.0334368
